# Abstracts Presented at the 32nd Annual Conference of the International Society for Quality of Life Research

**DOI:** 10.1186/s41687-026-01004-5

**Published:** 2026-04-23

## 101.1 Exploring Variability in EQ VAS scores: A Systematic Review and Meta-regression of Population Health Studies

Ling Jie Cheng^1^, Feng You-Shan^2^, Jing Ying Cheng^3^, Le Ann Chen^4^, Nan Luo^4^

^1^National Perinatal Epidemiology Unit, Nuffield Department of Population Health, University of Oxford, Oxford, UK, ^2^Institute for Clinical Epidemiology and Applied Biometrics, Medical University of Tübingen, Tübingen, Germany, ^3^Khoo Teck Puat Hospital, Yishun Health, National Healthcare Group, Singapore, Singapore, ^4^Saw Swee Hock School of Public Health, National University of Singapore, Singapore, Singapore

*Journal of Patient-Reported Outcomes 2026*, **10(Suppl 1)**:101.1

### Aims

The EQ Visual Analogue Scale (VAS), included in all EQ-5D questionnaires, is widely used in patient and general population surveys to assess overall health status. While its measurement properties are well established in general populations, qualitative studies have highlighted substantial interpretative variability among respondents. This systematic review aimed to identify contributors to global variability in mean EQ VAS scores.

### Methods

We searched eight databases for English-language observational studies reporting EQ VAS scores in general population surveys from inception to 24 January 2025. We extracted ten pre-specified variables: (1) comorbidity prevalence, (2) mean age, (3) gender ratio, (4) publication year, (5) EQ-5D version (3L/5L), (6) region (Europe, Americas, Asia, Oceania, Middle East), (7) sampling method, (8) administration mode, (9) survey venue, and (10) language. Regions were classified according to the UN Statistics Division. We conducted random-effects meta-analysis, subgroup analysis, and meta-regression using R (metafor package).

### Results

Of 16,525 records, 55 estimates from 51 articles were included, comprising 24 language versions and 496,531 adults. Most data were from Europe (45.5%), South-East/East Asia (23.6%), and the Americas (18.2%). The mean age was 46.3 years (SD 6.4). Probabilistic sampling (72.7%) and interviewer-administered surveys (65.5%), especially household-based (87.3%), were commonly used. Univariate analyses identified comorbidity prevalence, age, region, administration mode, and language as significant contributors. In adjusted models, only comorbidity prevalence (β = –10.0, 95% CI –18.1 to –1.9) and the South-East/East/South Asia region (β = 7.6, 95% CI 1.4 to 13.9) remained significant, explaining 51.6% of the variability.

### Conclusion

Variability in mean EQ VAS scores is largely explained by region and comorbidity prevalence. The observed difference between Asian and European populations—exceeding the commonly cited 5-point minimally important difference (MID) for EQ VAS—aligns with larger disparities seen in EQ-5D descriptive systems, possibly reflecting ceiling effects. These findings support the EQ VAS as a promising instrument for cross-regional health comparisons, given its independence from value sets. Further data from underrepresented and absent regions are needed to enhance the generalisability of these results.

## 101.2 Holistic Health in Crisis: Identifying Key Health Issues and Behaviors Across 29 Nations

Daniel Fong^1^, Jiaying Li^2^, Kris Lok^1^, Mandy Ho^1^, Vinciya Pandian^3^, Patricia Davidson^4^, Wenjie Duan^5^, Marie Tarrant^6^, Jung Jae Lee^1^, Chia-Chin Lin^1^

^1^The University of Hong Kong, Hong Kong, Hong Kong, ^2^Johns Hopkins University, Baltimore, Maryland, USA, ^3^The Pennsylvania State University, Washington, District Of Columbia, USA, ^4^University of New South Wales, Sydney Australia, ^5^East China University of Science and Technology, Shanghai, China, ^6^University of British Columbia, Kelowna, British Columbia, Canada

*Journal of Patient-Reported Outcomes 2026*, **10(Suppl 1)**:101.2

### Aims

Pandemics can profoundly influence diverse aspects of health, with effects varying across countries. While prioritization of health issues often relies on their severity or local burden, targeting central health issues—those most interconnected with other health domains—may yield more systemic benefits. This study aimed to (1) identify globally prevalent central health issues during a pandemic and (2) determine bridging lifestyles, i.e., the behaviors most effective in addressing these central health issues.

### Methods

A multinational cross-sectional study was conducted across all six World Health Organization regions, involving community-dwelling adults. Participants reported pandemic-related changes in 13 health domains (e.g., quality of life, emotional distress, social support, physical health) and 18 lifestyle behaviors. Country-specific network analyses were performed to identify central health issues (based on strength centrality) and bridging lifestyles.

### Results

After removing incomplete and inconsistent responses, 16,461 responses (63% females) from 29 countries were analyzed. The top five most influenced health issues were mental burden, emotional distress, economic burden, weight lost, and appetite. In contrast, six central health issues were identified, with the most prevalent one across the countries were emotional distress (in 13 countries), followed by quality of life (in 7 countries), sleep quality (in 4 countries), social support (in 3 countries), mental burden (in 3 countries), and physical health (in 1 country). The most prevalent bridging lifestyles were exercise (in 17 countries), followed by cooking at home (in one country) and food types in daily meals (in 1 country).

### Conclusion

Emotional distress and quality of life were identified as the most prevalent central health issues across countries, suggesting their critical role in holistic health improvement during pandemics. Exercise stood out as a key bridging lifestyle with widespread influence, underscoring its potential as a scalable intervention. These findings advocate for a network-informed approach to public health, emphasizing the prioritization of central health issues and bridging behaviors, particularly in resource-constrained settings. Future research should explore longitudinal dynamics and cultural mediators of these relationships to further refine intervention strategies.

## 101.3 Measuring What Matters in Primary Care: Health-Related Quality of Life and Its Predictors Among Users of Public Primary Care Clinics in Singapore Using the EQ-5D-5L

Ling Jie Cheng^1^, Qin Xiang Ng^2^, Guang Jie Justin Lee^2^, Gerald Choon Huat Koh^3^, Nan Luo^3^

^1^National Perinatal Epidemiology Unit, Nuffield Department of Population Health, University of Oxford, Oxford, UK, ^2^Saw Swee Hock School of Public Health, National University of Singapore and National University Health System, Singapore, Singapore, ^3^Saw Swee Hock School of Public Health, National University of Singapore, Singapore, Singapore

*Journal of Patient-Reported Outcomes 2026*, **10(Suppl 1)**:101.3

### Aims

Health-related quality of life (HRQoL) is increasingly recognised as a key outcome in healthcare delivery, particularly in primary care settings where multimorbidity is rising. However, in high-income Asian contexts like Singapore, population-level data on HRQoL among primary care users remain limited. Existing studies often focus on hospital-based or disease-specific populations. Few have explored the combined impact of sociodemographic, clinical, and patient-reported factors on both EQ-5D-5L index (preference-based) and EQ VAS (subjective) scores. This study assessed HRQoL among public primary care users in Singapore using the EQ-5D-5L and identified associated sociodemographic and health-related factors.

### Methods

This is a secondary analysis of data collected as part of a larger study on the validation of the Consumer Health Activation Index (CHAI) in Singapore. A cross-sectional survey was conducted among 572 adult patients recruited from public polyclinics. Participants completed the EQ-5D-5L, CHAI, and a structured questionnaire covering demographics, chronic conditions, and self-rated health. Univariate analyses assessed subgroup differences. Multivariable regressions identified independent predictors. Due to ceiling effects in EQ index scores, a two-part model was used: logistic regression for full health (index = 1) and generalised linear regression for scores <1. EQ VAS was analysed using standard linear regression.

### Results

Mean EQ index and EQ VAS scores were 0.89 (SD = 0.16) and 77.0 (SD = 12.7), respectively. Participants with poor/fair self-rated health had lower EQ index (0.83) and EQ VAS (68.6) scores than those reporting very good/excellent health (EQ index: 0.95; EQ VAS: 84.3), exceeding typical minimally important difference thresholds. Multivariable analyses showed that absence of comorbidities, higher health activation, and better self-rated health were associated with higher EQ index scores; Malay ethnicity was associated with lower scores. For EQ VAS, younger age, higher activation, and better self-rated health were positively associated; female gender was associated to slightly lower scores.

### Conclusion

This study provides new insights into HRQoL in Singapore’s primary care population, highlighting disparities by age, comorbidity, and ethnicity. Health activation and self-rated health were strong correlates, underscoring the importance of patient engagement. Findings support integrating EQ-5D-5L into routine care for personalised service delivery and population health planning.

## 101.4 Exploring the Use of Health-Related Quality of Life Measures in Africa: A systematic scoping review

Begashaw Gebresillasie^1^, Lucky Gift Ngwira^2^, Jermaine Dambi^3^, Adeladlew Netere^4^, Eyayew Belashew^1^

^1^University of Gondar, Gondar, Ethiopia, ^2^Health Economics and Policy Unit, Kamuzu University of Health Sciences, Blantyre, Malawi, ^3^University of Zimbabwe, Harare, Zimbabwe, ^4^Monash University, Melbourne, Australia

*Journal of Patient-Reported Outcomes 2026*, **10(Suppl 1)**:101.4

### Aims

Health-related quality of life (HRQoL) is an important measure in healthcare that captures the multidimensional effects of health on individuals’ lives. While HRQoL measures are widely used globally, there is limited consolidated evidence of their application in Africa. This review aimed to explore and map the available evidence on the utilization of HRQoL measures in Africa, with a focus on understanding their application across diverse populations, health conditions, and geographical regions while identifying gaps, methodological inconsistencies, and opportunities for future research.

### Methods

The scoping review was conducted following the Arksey and O’Malley framework. Electronic databases, including Medline, Embase, CINAHL, and Cochrane library, and other sources, were searched from the inception up until September 2024. Results were synthesized narratively with descriptive statistics, presented using tables and graphs.

### Results

Out of 20,242 records identified, 731 studies met the inclusion criteria. The geographical distribution revealed significant disparities, with over half of the studies originated from Nigeria (21.8%), Ethiopia (14.8%), and South Africa (14.5%). Most studies (74.2%) employed descriptive designs and were conducted in hospital settings (70.3%). HRQoL instruments were used across a wide range of health conditions, with HIV/AIDS (20.5%), cancer (10.1%), and diabetes (7.1%) being the most frequently studied. Generic HRQoL tools such as WHOQOL-BREF (19.7%), SF-36 (16.4%), and EQ-5D (13.1%) were the most commonly utilized, accounting for nearly half of all studies. The analysis of information reporting practices for EQ-5D instruments revealed inconsistencies, with only 20.3% of studies reporting all three components (descriptive profile, index scores, and EQ-VAS).

### Conclusion

This review highlighted the increasing use of HRQoL measures in African health research over the past two decades while identifying significant gaps. Most studies were descriptive, concentrated in a few countries, and primarily conducted in healthcare settings, limiting broader applicability. The frequent use of generic HRQoL instruments underscores their importance and cultural adaptation. Additionally, inconsistencies in reporting practices and methodological limitations hinder the comparability and utility of findings. To advance HRQoL research and its application, it is essential to address these gaps through standardized guidelines, broader geographical representation, and improved methodological rigor.

## 101.5 Financial toxicity and health: Results from a U.S. nationally representative survey

Oyiza Usman^1^, Joanna Balza^1^, Sarah Reed-Thryselius^1^, Miranda Kapfhammer^1^, Idayat Akinola^1^, Kathryn Flynn^1^, Rachel Cusatis^1^

^1^Medical College of Wisconsin, Milwaukee, Wisconsin, USA

*Journal of Patient-Reported Outcomes 2026*, **10(Suppl 1)**:101.5

### Aims

Financial toxicity (FT) is harmful financial sequelae that patients and their families experience due to medical care or treatment, which can result from the cost of treatment or indirect reasons, such as job loss due to the health condition. We evaluated the association between FT, health, quality of life, socioeconomic, and demographic factors in a large U.S. sample.

### Methods

A nationally representative cross-sectional electronic survey was collected through an online panel (YouGov) in 2024. FT was assessed using the COST-FACIT, a 12-item measure that was validated in a cancer population. The COST-FACIT wording was adapted for the general population, in collaboration with measure developers. COST-FACIT scores range from 0-44, with higher scores indicating greater levels of financial wellness; a cut-off score of <13 represents FT (grades 2 and 3). Weighted bivariate analyses of FT scores, general health, and quality of life were performed using linear regression, and multiple regression was performed to assess the relationship between FT and health conditions, socioeconomic, and demographic characteristics. Results were considered significant at p<0.05.

### Results

Sample (n=2470) demographics generally reflected the U.S. general population. The mean COST-FACIT score was 26.10 (± 0.23), and 11.6% of U.S. adults reported a FT grade 2 or 3. Higher global quality of life and general health ratings were significantly associated with higher financial wellness and demonstrated a dose relationship (Figure 1). After adjusting for socioeconomic and demographic characteristics, the health conditions significantly associated with FT included migraines (1.92), diabetes (1.46), depression or anxiety (3.83), and sleep disorders (1.69). Cardiac conditions (0.43), cancer (-0.88), and alcohol or drugs (1.47) were not significantly associated with FT after adjusting for other variables (Table 1).

### Conclusion

In a large, nationally representative U.S. sample, 1 in 10 participants reported FT. FT was associated with worse self-reported health status and quality of life. As expected, socioeconomic characteristics and diagnosed health conditions were significant predictors of FT, though cancer was not a significant predictor when adjusting for other characteristics. Strategies that protect patients from the high costs of medical treatments are likely to ameliorate FT.

**Table 1 (abstract 101.5):**
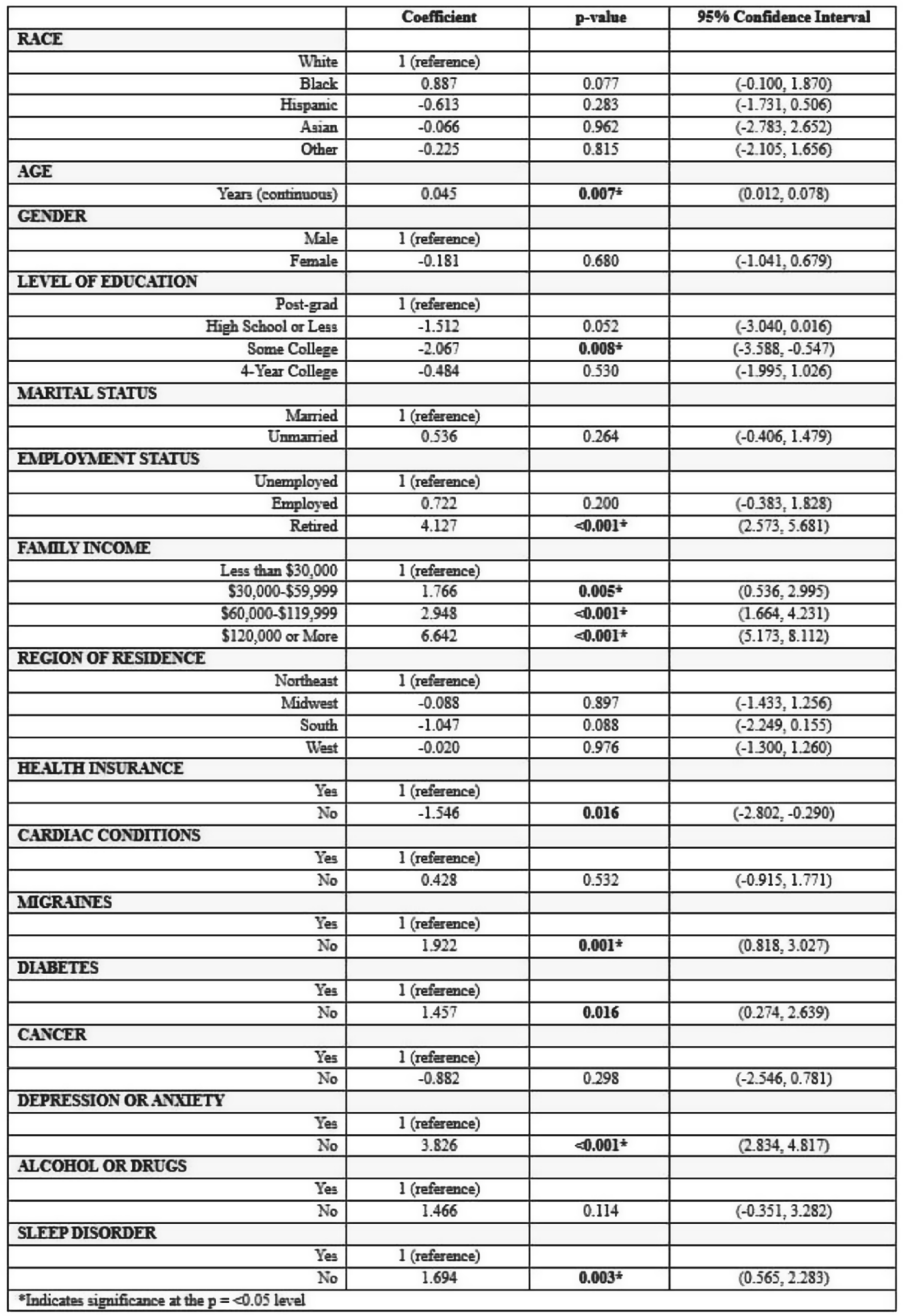
Multiple Regression Model of Characteristics that Predict Financial Toxicity in a US Nationally Representative Sample (n = 2,470)

**Fig. 1 (abstract 101.5):**
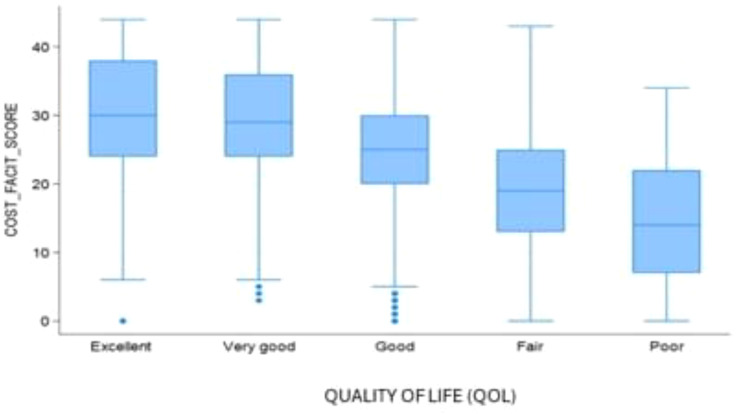
Financial Toxicity by Quality of Life

**Fig. 2 (abstract 101.5):**
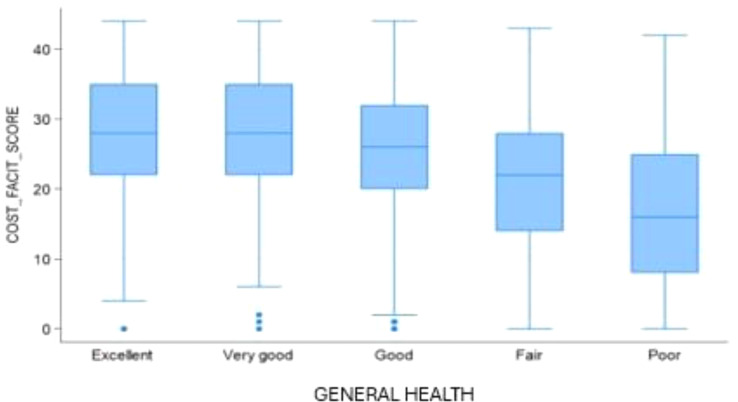
Financial Toxicity by General Health

## 102.2 Association between patient-reported adverse events (AEs) and health-related quality of life (HRQoL) in chronic myeloid leukemia (CML) – Analysis of PRO-CTCAE and PROMIS Global Health measures

Kelly Schoenbeck^1^, David Wei^2^, Nisha Hazra^3^, Cristina Constantinescu^4^, Yan Meng^3^, Dominick Latremouille-Viau^5^, Gabriel Marquez^6^, Daisy Yang^2^, Andrea Damon^2^, Islam Sadek^2^, Annie Guérin^5^, Kathryn Flynn^7^

^1^University of California San Francisco, Division of Hematology and Oncology, San Francisco, California, USA, ^2^Novartis Pharmaceuticals Corporation, East Hanover, New Jersey, USA, ^3^Analysis Group, Ltd., London, UK, ^4^Ipsos, Basel, Switzerland, ^5^Analysis Group, Inc., Montreal, Quebec, Canada, ^6^Novartis Pharmaceuticals Corporation, Dublin, Ireland, ^7^Medical College of Wisconsin, Department of Medicine, Milwaukee, USA

*Journal of Patient-Reported Outcomes 2026*, **10(Suppl 1)**:102.2

### Aims

To evaluate the impact of patient-reported tyrosine kinase inhibitor (TKI)-related AEs on patients’ HRQoL in CML.

### Methods

The cross-sectional online SHIFT survey (06/2024-12/2024) included adult patients with CML on first/second TKI in the US. Data on 18 TKI-related AEs were collected using the PRO-CTCAE questionnaire. HRQoL was assessed via the PROMIS Global Health-10 (Global Physical Health [GPH] and Global Mental Health [GMH] T-scores) and the Work Productivity and Activity Impairment Specific Health Problem (percent activity impairment) instruments. For each HRQoL measure, two generalized linear models (with log-link) were developed to analyze the association with: (1) AE presence and persistence, and (2) AE types.

### Results

A total of 271 patients (female: 59%, white: 80%, median age: 46 years, first/second TKI: 60%/40%) reported currently experiencing a median of 3 AEs (range: 0-14) and 1 persistent AE (range: 0-9) in the last 7 days. AE types are reported in Figure 1. Mean±SD GPH and GMH T-scores were 42.6±7.0 and 44.4±7.7, respectively, indicating worse health than the general population (50±10), and activity impairment (%) was 37.3±24.6.Patients experiencing AEs had significantly lower GPH T-scores (by 2.6 points from the CML mean) and higher activity impairment (by 17.5 percentage-points from the CML mean), with each persistent AE further reducing GPH T-scores by 1.3 points and increasing activity impairment by 4.5 percentage-points. GMH T-scores declined by 1.3 points with each additional persistent AE (Figure 2; all p<0.05).Lower GPH T-scores were associated with shortness of breath (by 3.8 points), fatigue and pain (each by 2.1 points), and gastrointestinal (by 1.7 points) AEs. Mental health AEs (3.1 points lower), fatigue and cognitive AEs (each 2.2 points lower) had the greatest impact on GMH T-scores. AEs leading to greater activity impairment were shortness of breath (15.7 percentage-points higher), fatigue (11.9 percentage-points higher) and cognitive AEs (9.7 percentage-points higher) (Figure 3; all p<0.05).

### Conclusion

The SHIFT study highlights the significant impact of TKI-related AEs on patients’ HRQoL in CML, with persistent AEs, shortness of breath, and fatigue exacerbating these effects. Findings emphasize the importance of timely interventions and better-tolerated treatments to minimize AEs and preserve HRQoL.

**Fig. 1 (abstract 102.2):**
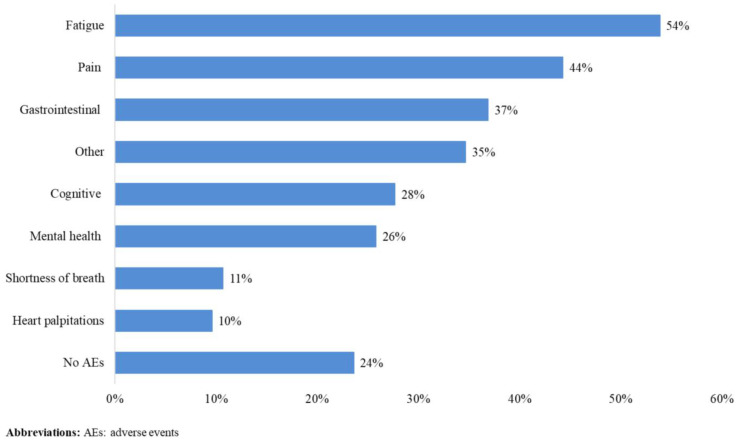
TKI-related AE types in patients with CML

**Fig. 2 (abstract 102.2):**
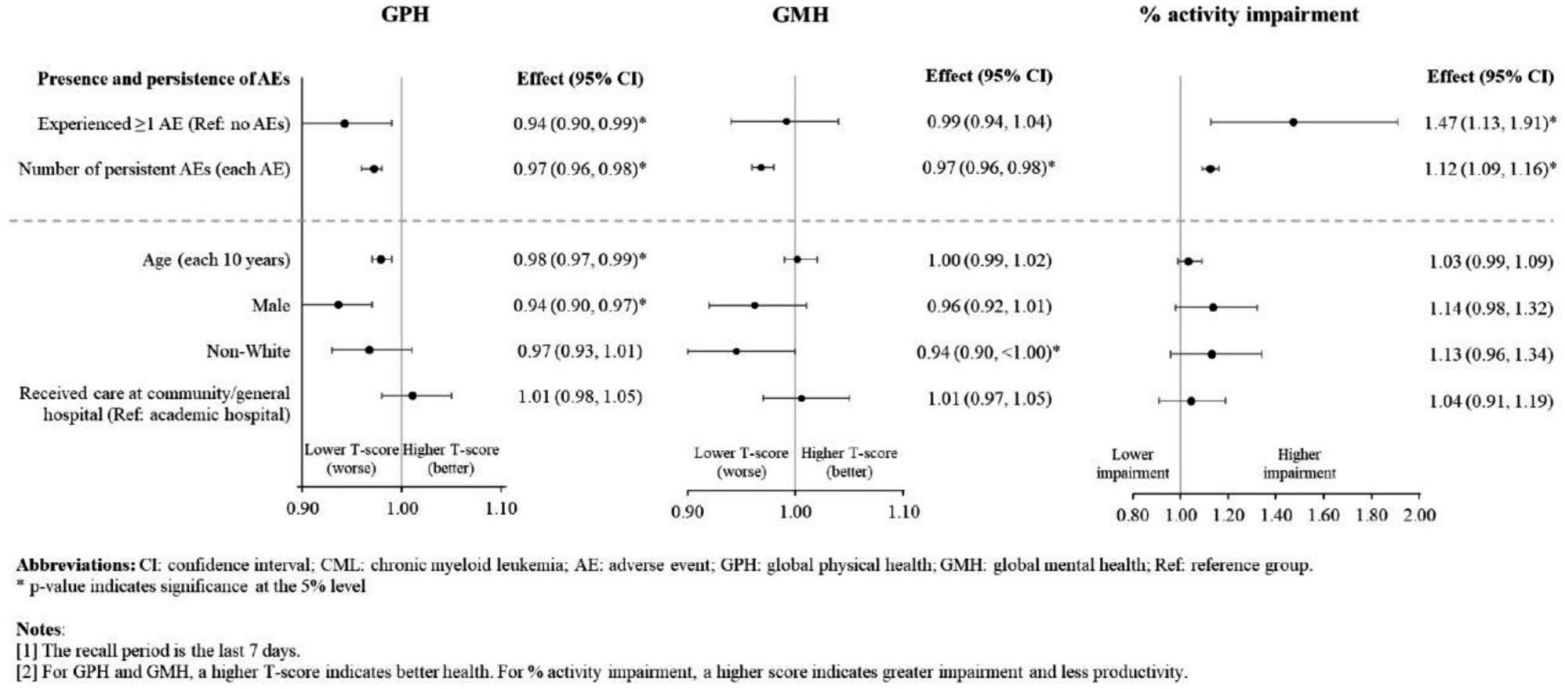
Impact of presence and persistence of TKI-related AEs on patients’ QoL in CML

**Fig. 3 (abstract 102.2):**
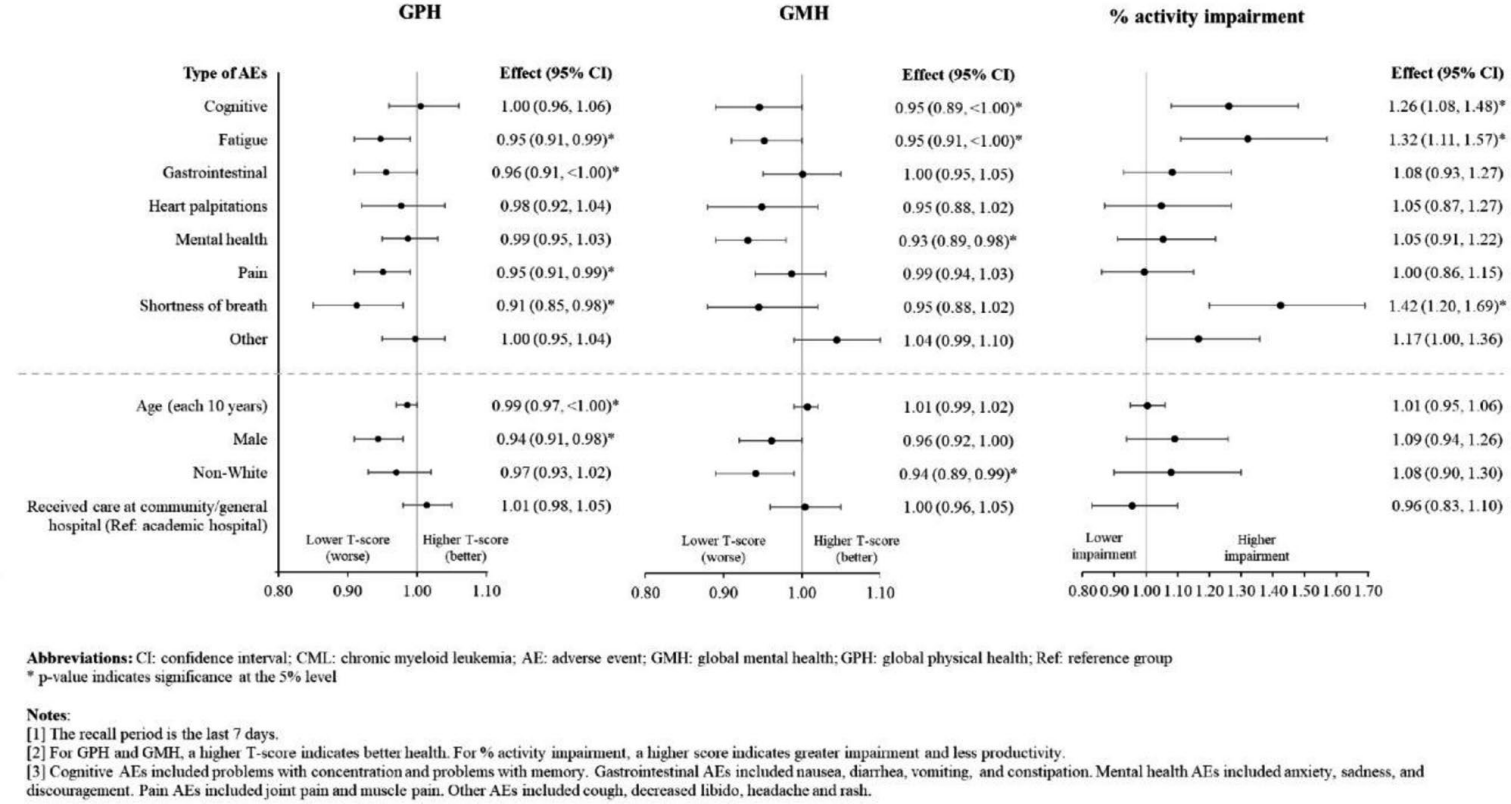
Impact of type of TKI-related AEs on patients’ QoL in CML

## 102.1 Update of the EORTC Multiple Myeloma module (QLQ-MY20): Results from qualitative interviews with patients and healthcare professionals

Katie Forde^1^, Eleanor Scouler^1^, Kim Cocks^1^, Duska Petranoviae^2^, Jens Lehmann^3^, Simone Oerlemans^4^, Ioannis Ntanasis Stathopoulos^5^, Varnavas Constantinou^6^, Lene Kongsgaard Nielsen^7^, Waleed Alrjoub^8^, Omar Shamieh^8^, Evangelos Terpos^5^, Jo Hargroves^9^, Charalampia Kyriakou^9^

^1^Adelphi Values Ltd, Bollington, UK, ^2^University of Rijeka, Rijeka, Croatia, ^3^University of Innsbruck, Innsbruck, Austria, ^4^Netherlands Comprehensive Cancer Organization, Utrecht, Netherlands, ^5^University of Athens, Athens, Greece, ^6^German Medical Institute, Limassol, Cyprus, ^7^Research Unit of Hematology, Odense, Denmark, ^8^King Hussein Cancer Center, Amman, Jordan, ^9^University College London, London, UK

*Journal of Patient-Reported Outcomes 2026*, **10(Suppl 1)**:102.1

### Aims

The European Organization for the Research and Treatment of Cancer Myeloma module (EORTC QLQ-MY20), used alongside the EORTC QLQ-C30 core questionnaire, was published in 1999 to assess health-related quality of life in patients with multiple myeloma (MM). Since its development, treatment and survival rates for MM have advanced dramatically with patients undergoing multiple lines of therapy and relapses. To ensure the QLQ-MY20 remains relevant and fully captures the disease experience, and to address limitations of the original module, qualitative interviews with patients and healthcare professionals were conducted to inform module modification.

### Methods

The module update is being conducted in accordance with the EORTC Quality of Life Group module development guidelines. Following the conduct of two literature reviews, collating a list of concepts and identifying methodological problems with the original module, and a review of side effects of MM treatments, qualitative interviews took place with n=93 patients and n=20 healthcare professionals across Europe (7 countries) and the Middle East (1 country), to assess the relevance/importance of the concepts and to elicit any further concepts for consideration. Concepts rated as sufficiently relevant/important according to a priori criteria were used to create a conceptual framework.

### Results

20 issues met the criteria for importance and relevance from the patient and healthcare professional interviews and were deemed to be specific to MM and/or MM treatment; 11 items were from the original QLQ-MY20 and 9 were new concepts. 9 items from the original module were removed as they were not deemed sufficiently relevant/important and/or were not specific to MM. Items were refined based on discussions with collaborators and EORTC feedback, resulting in 24 items (QLQ-MY24); a conceptual framework (Figure 1) was developed to illustrate the groups of items hypothesized to measure similar concept/symptom domains. These groups of items included: pain, neuropathy, infection, edema, tiredness, weight loss, ocular, oral, restlessness/agitation, and hair loss.

### Conclusion

Next steps will include debriefing interviews with patients with MM to identify and rectify potential problems in administration and to identify missing/redundant concepts. Preliminary evaluation of psychometric properties and international validation field testing of the updated module is then expected.

**Fig. 1 (abstract 102.1):**
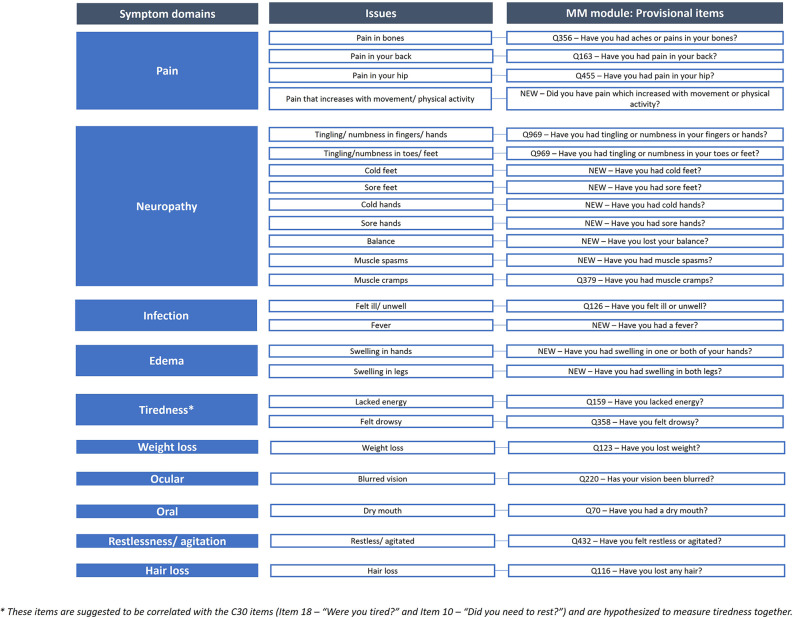
Update of the EORTC Multiple Myeloma module (QLQ-MY20): Results from qualitative interviews with patients and healthcare professionals

## 102.3 Steroid-toxicity in newly diagnosed patients with multiple myeloma treated with a limited dexamethasone regimen; results from the NMSG REST study

Lene Kongsgaard Nielsen^1^, Tine Rosenberg^2^, Andreas Kristian Pedersen^3^, Claudia Rutherford^4^, Tracy King^5,6^, Margaret-Ann Tait^4^, Einar Haukås^7^, Tobias Slørdahl^8^, Anja Klostergaard^9^, Emil Hermansen^10^, Fredrik Schjesvold^11^, Frida Bugge Askeland^12^

^1^Quality of Life Research Center, Odense University Hospital, Odense, Denmark, ^2^Quality of Life Research Center, Department of Hematology, Odense University Hospital, Odense, Denmark, ^3^Open Patient data Explorative Network, Odense University Hospital, Odense, Denmark, ^4^Sydney Quality of Life Office, Susan Wakil School of Nursing and Midwifery, Faculty of Medicine and Health, University of Sydney, Sydney, Australia, ^5^Department of Hematology, Royal Prince Alfred Hospital, Sydney, Australia, ^6^Cancer Care Research Unit, Susan Wakil School of Nursing, University of Sydney, Sydney, Australia, ^7^Department of Cancer and Blood Diseases, Stavanger University Hospital, Stavanger, Norway, ^8^Department of Hematology, St. Olavs Hospital and Department of Clinical and Molecular Medicine, Norwegian University of Science and Technology, Trondheim, Norway, ^9^Department of Hematology, Aarhus University Hospital, Aarhus, Denmark, ^10^Department of Hematology, Copenhagen University Hospital, Copenhagen, Denmark, ^11^Oslo Myeloma Center, Department of Hematology, Oslo University Hospital, Oslo, Norway, ^12^Oslo Myeloma Center, Department of Hematology, Oslo University Hospital and Institute of Clinical Medicine, University of Oslo, Oslo, Norway

*Journal of Patient-Reported Outcomes 2026*, **10(Suppl 1)**:102.3

### Aims

For decades, corticosteroids (steroids) have been essential in treating patients with multiple myeloma (MM). However, the role of steroids’ in the era of novel therapies is uncertain. Considering the unfavourable toxicity profile of steroids, the phase II REST study investigated the efficacy of a quadruplet regimen with limited steroids (dexamethasone) in newly diagnosed MM patients ineligible for autologous transplant. Patients were treated with isatuximab-bortezomib-lenalidomide-dexamethasone; dexamethasone was omitted after 2 cycles, bortezomib after 8 cycles and isatuximab after 18 cycles. Efficacy was comparable with full-dose steroid regimens. This study aimed to investigate the impact of omitting dexamethasone on patient-reported steroid-toxicity.

### Methods

Patients completed the Steroid-Symptom Questionnaire for patients with MM (SSQ-MM) and EORTC QLQ-C30 at day 1 and 22 of cycle 1 and 2 (with dexamethasone) and cycle 4 and 5 (without dexamethasone). The SSQ-MM Total score assessed steroid-toxicity. Mean change from baseline and between groups mean score differences between cycles with and without dexamethasone were estimated using linear mixed model of repeated measures (LMRM). Statistically significant estimates (p-value <0.05) were interpreted using minimal important difference and Benjamini-Hoechberg procedure to avoid type I errors. The proportion of patients developing steroid-toxicity during cycles with and without dexamethasone was compared using mixed effect logistic regression. To investigate the impact of steroid-toxicities on health-related quality of life (HRQL), we employed again LMRM including a Gaussian random intercept for each patient.

### Results

51 patients were included with a median age of 77 years (range 70-88). Questionnaire completion rate was 88%. At cycle 1 day 22 (C1D22) and C2D22, patients reported clinically meaningful greater steroid-toxicity compared to baseline (p-values <0.01), but only at C1D22 significantly more patients reported steroid-toxicity compared to C4D22 (42% vs 7%, p-value 0.004). Mean score differences in steroid-toxicity between cycles with and without dexamethasone were not statistically significant. Steroid-toxicity was associated with impaired physical, emotional and social functioning and greater fatigue, pain, appetite loss and insomnia.

### Conclusion

Steroids are included in most myeloma-targeted therapies. However, growing evidence shows similar responses with steroid-limited treatments. In this steroid-limited study, patients reported steroid-toxicity affecting several HRQL domains in cycles with dexamethasone.

## 102.5 Qualitative research to understand the burden of disease and concept relevance of the PROMIS-Fatigue for patients with sickle cell disease

Phoebe Wright^1^, Jungyoon Moon^2^, Julie Whyte^3^, Jason Hyman^3^, Sara Loniewski^3^, Caroline Rawls^3^, Amber Yates^2^

^1^Agios, Seattle, Washington, USA, ^2^Agios Pharmaceuticals Inc., Cambridge, Massachusetts, USA, ^3^Lumanity, Boston, Massachusetts, USA

*Journal of Patient-Reported Outcomes 2026*, **10(Suppl 1)**:102.5

### Aims

Sickle cell disease (SCD) is characterized by acute, painful episodes (i.e., vaso-occlusive events), general chronic pain, and fatigue; however, less is known about how these symptoms—particularly fatigue—affect patients’ daily lives. The study objectives were to better understand the disease-related symptoms and impacts of SCD from the patient perspective, explore the relevance of concepts in the PROMIS-Fatigue Short Form 13a (SF13a), and capture treatment experiences of adult patients with SCD through qualitative interviews.

### Methods

Adult patients with a confirmed diagnosis of SCD (e.g., a prescription for SCD medication; medical records) were recruited through patient advocacy groups. Eligible participants completed a 90-minute interview, which used a semi-structured interview guide including questions and probes designed to understand symptoms and impacts associated with SCD, with an emphasis on patient experience of fatigue. The relevance of the PROMIS-Fatigue SF13a to patients was assessed by examining the proportion of patients who mentioned concepts in PROMIS-Fatigue items during their interview, either spontaneously or following interviewer probes.

### Results

Twenty participants aged 27–53 completed interviews; 95% were female and 100% identified as Black or African American. All participants reported experiencing SCD-related pain and fatigue, with fatigue and general pain (i.e. not associated with pain crises) identified as the most bothersome symptoms (Figure). Participants reported (either spontaneously or when probed) fatigue-related impacts such as needing to sleep during the day, being unable to do usual activities, and feeling frustrated by being too tired to do things. All PROMIS-Fatigue SF13a concepts were relevant to the majority of participants; 8/13 concepts were relevant to ≥75% of participants, with concepts in items HI7 (fatigue), HI12 (weak all over), and AN2 (tired) relevant to all participants (Table). Regarding improvement of treatment experience, fatigue and pain were the most frequently-reported symptoms that patients want new SCD medications to better address.

### Conclusion

This qualitative research demonstrates that the burden of disease is high in patients with SCD, with both symptoms of pain and fatigue most salient to their experience. Further, these results support the PROMIS-Fatigue SF13a as a content-relevant instrument to capture fatigue-related symptoms and impacts among adult patients with SCD.

**Table 1 (abstract 102.5):**
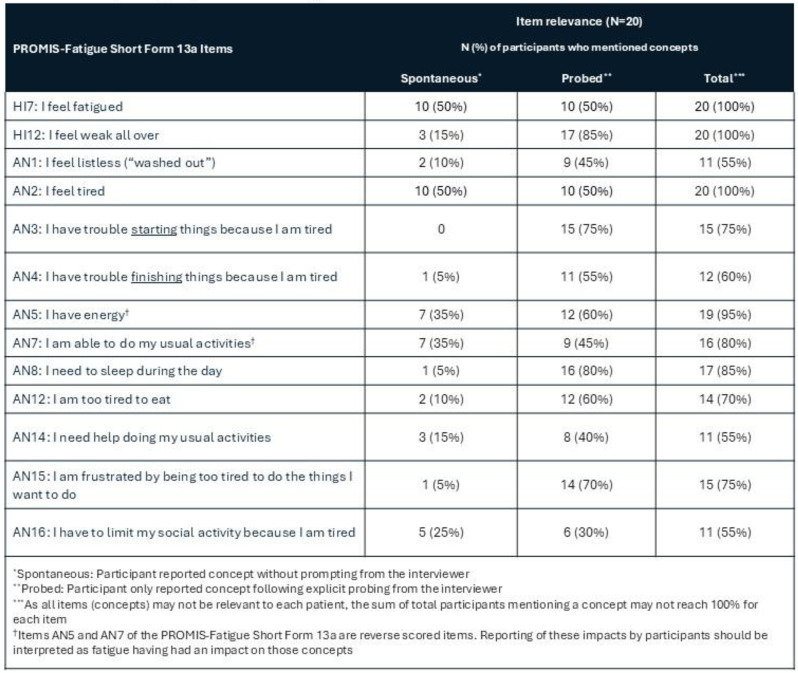
Concept mapping to PROMIS-Fatigue Short Form 13a

**Fig. 1 (abstract 102.5):**
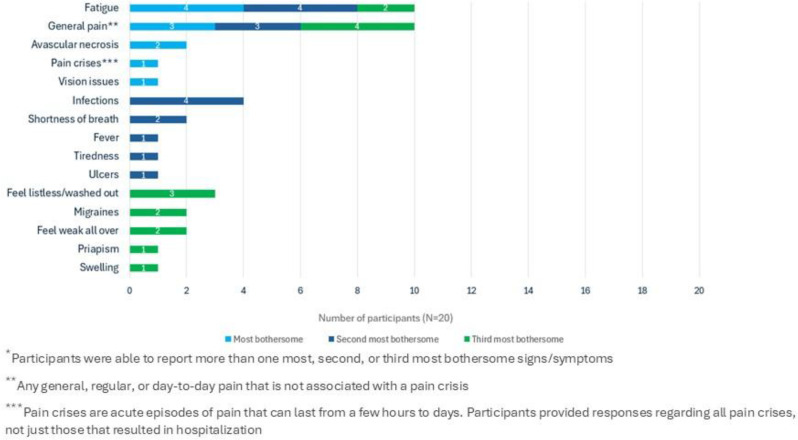
Most bothersome signs and symptoms to treat *

## 102.4 Patient Reported Outcome Data for Daratumumab Treatment Readiness in Multiple Myeloma; Assessing the Feasibility

Tine Rosenberg^1^, Jannie Kirkegaard^2^, Michael Tveden Gundesen^2^, Anne Mette Ølholm^3^, Karin Brochstedt Dieperink^4^, Thomas Lund^5^

^1^Quality of Life Research Center, Department of Hematology, Odense University Hospital, Odense, Denmark, ^2^Department of Hematology, Odense University Hospital, Odense, Denmark, ^3^Centre for Innovative Medical Technology, Odense University Hospital, Odense, Denmark, ^4^Research Unit of Oncology, Odense University Hospital, Odense, Denmark, ^5^Department of Medicine, Hematological Section, Vejle Hospital, Vejle, Denmark

*Journal of Patient-Reported Outcomes 2026*, **10(Suppl 1)**:102.4

### Aims

To test if Patient Reported Outcome (PRO) data can replace clinical evaluation in determining if patients with multiple myeloma are ready to receive their next planned dose of daratumumab, and to assess the feasible of this strategy for all patients.

### Methods

We developed an electronic questionnaire addressing common side effects to daratumumab and an algorithm stratifying patients according to their responses. Applying a mixed-method study design, we tested its usability, defined in this study as reliability, learnability, and user satisfaction. Quantitative data were descriptively analyzed, and positive predictive value and negative predictive value were calculated using the standard clinical evaluation – conducted independently and without knowledge of the algorithm’s output – as the reference standard. Qualitative data were obtained from individual, semi-structured interviews with patients (n=19) and a focus group interview with healthcare professionals (n=4); data were analyzed using a hermeneutic approach.

### Results

With a positive predictive value of 100%, we found the questionnaire able to identify patients physically fit for treatment without need for further consultation. Of 179 completed questionnaires, the algorithm recommended treatment in 142 cases, thus demonstrating the potential of PRO data to replace standard clinical evaluations in 79% of cases. However, with a patient response rate of 77%, we also found that some patients were unable to report side effects digitally using a smartphone themselves. With respect to gender and age we found no statistical difference between patients being able or unable to complete the questionnaires. Qualitative findings were confirmative suggesting that patients had very different perceptions of registering their side effects themselves.

### Conclusion

Self-reporting of side effects prior to treatment is advantageous and flexible for the majority of patients. For patients able to complete the questionnaires, it is reliable and capable of replacing the usual clinical evaluation in determining treatment readiness. It thus holds potential to release resources at the hospital. However, further studies are needed to better understand which patients benefit from this approach and which require additional support. Identifying key factors influencing usability, such as digital literacy, cognitive function, and patient preferences, will be essential for optimizing implementation in a clinical setting.

## 103.1 Linking the Physical-Performance Test to the PROMIS physical function metric: results from a prospective multicenter study

Audrey Yuki Brinker^1^, Felix H. Fischer^1^, Steven Bindseil^1^, Lisa M. Reinhold^1^, Julia Winkler^1^, Volkan Aykac^1^, Frank Buttgereit^1^, Andreas Heißel^2^, Volker Köllner^1^, Ursula Müller-Werdan^1^, Udo Schneider^1^, Matthias Rose^1^, Gregor Liegl^1^

^1^Center for Patient-Centered Outcomes Research (CPCOR), Department of Psychosomatic Medicine, Charité – Universitätsmedizin Berlin, Corporate Member of Freie Universität Berlin, Humboldt-Universität zu Berlin, and Berlin Institute of Health, Berlin, Germany, ^2^Sport- und Gesundheitspark Berlin e.V., Berlin, Germany

*Journal of Patient-Reported Outcomes 2026*, **10(Suppl 1)**:103.1

### Aims

Physical function (PF) is a relevant clinical outcome, commonly assessed via patient-reported (PRO) or performance-based (PerfO) outcome measures. Although various PRO measures have been calibrated to the standardized PROMIS-PF T-score metric, a current challenge lies in the inability to compare scores across different assessment types. To overcome this gap, the ‘StandardizingPF’ project was initiated, aiming to link PerfO assessments onto a common scale with PRO measures. This contribution aims to presents the linking of a comprehensive PerfO battery - the Physical Performance Test (PPT) - to the PROMIS-PF metric across diverse populations, allowing for comparisons between assessment types.

### Methods

Adult participants from different settings completed both a generic 20-item PROMIS-PF short form (PROMIS-PF20a) and the PPT, including 9 timed physical performance tasks. Assumptions of unidimensional item response theory (IRT) modeling were evaluated for the combined set of 29 items from PROMIS-PF20a and the PPT. Next to correlation analysis, non-parametric IRT as well as confirmatory and exploratory factor analysis was applied. We then linked the PPT items to the PROMIS-PF T-score metric applying unidimensional graded response modeling with PROMIS-PF20a item parameters fixed to the original PROMIS-PF metric.

### Results

Data from N=810 patients from geriatrics (n=224), rheumatology (n=204), psychocardiological rehabilitation (n=201; of which long-COVID n=77), as well es non-clinical elderly individuals (age ≥60; n=181) were analyzed (64.6% women, median age=65.5). The mean PROMIS T-score was 40.7 and the mean PPT score was 24.1, indicating moderate functional impairment on average. The correlation between PROMIS-PF20a and PPT was high (Pearsons´s r=0.85). Confirmatory factor analysis supported essential unidimensionality of items from both measures (CFI=0.98; SRMR=0.06). After linking the PPT to the standardized PROMIS T-scale, we found satisfactory overall agreement between observed and PPT-based T-scores (RMSE=6.55, MAE=5.06, SMD<0.02). Nonetheless, population-specific linking algorithms may enhance score accuracy.

### Conclusion

This study demonstrates the feasibility of linking a performance-based physical function measure (PPT) to the standardized PROMIS-PF metric across diverse populations. The resulting linkage enables direct comparisons between PRO and PerfO assessments, facilitating the aggregation, comparison and interpretation of scores derived from different PF assessment types. Future research may explore the use of subgroup-specific linking functions to further improve score precision.

## 103.3 Can patient-reported and performance-based physical function measures similarly predict mortality?

Audrey Yuki Brinker^1^, Felix H. Fischer^1^, Mark Woodward^2^, Marietta Török^3^, Giovanni F.M. Strippoli^4^, Jörgen Hegbrant^5^, Andrew Davenport^6^, Krister Cromm^7^, Bernard Canaud^7^, Michiel L. Bots^8^, Peter J. Blankestijn^9^, Kathrin Fischer^1^, Matthias Rose^1^, Gregor Liegl^1^

^1^Center for Patient-Centered Outcomes Research (CPCOR), Department of Psychosomatic Medicine, Charité – Universitätsmedizin Berlin, Corporate Member of Freie Universität Berlin, Humboldt-Universität zu Berlin, and Berlin Institute of Health, Berlin, Germany, ^2^The George Institute for Global Health, School of Public Health, Imperial College London, London, UK, ^3^Diaverum, Malmö, Sweden, ^4^Department of Precision and Regenerative Medicine and Ionian Area (DiMePRe-J) University of Bari, Bari, Italy, ^5^Division of Nephrology, Department of Clinical Sciences, Lund University, Lund, Sweden, ^6^UCL Department of Renal Medicine, Royal Free Hospital & University College London, London, UK, ^7^Fresenius Medical Care Deutschland GmbH, Global Medical Office, Bad Homburg, Germany, ^8^Julius Center for Health Sciences and Primary Care, University Medical Center Utrecht, Utrecht University, Utrecht, Netherlands, ^9^Department of Nephrology & Hypertension, University Medical Center Utrecht, Utrecht, Netherlands

*Journal of Patient-Reported Outcomes 2026*, **10(Suppl 1)**:103.3

### Aims

Limitations in physical function (PF) do not only adversely affect quality of life, but it is well-known that PF is highly predictive of mortality, making its assessment relevant for evidence-based healthcare decisions. Currently, PF is commonly assessed through performance-based outcomes (PerfO), based on an individual’s actual quantifiable performance to standardized tests, and patient-reported outcomes (PRO), relying on patients’ direct response to standardized questions. However, the predictive value for mortality may vary depending on the specific type of PF measurement employed. We aimed to evaluate the most accurate PF assessment type for mortality prediction in a direct comparison using a standardized metric based on item-response theory (IRT).

### Methods

A survival analysis was conducted with data from the CONVINCE study, an international trial in end-stage kidney disease (ESKD) hemodialysis patients. Hazard ratios (HRs) were estimated using Cox proportional hazards regression, fitted independently for the:1. Patient-Reported Outcome Measurement Information System PF 4-item short form (PROMIS-PF4a),2. Performance-based Physical Performance Test (PPT),A third score was estimated for comparison purposes, derived from the combination of both measures. We applied a previously established common PROMIS T-score metric for the measures, utilizing the psychometrically linked PROMIS-PF T-score for the PPT. Predictive values were compared in unadjusted models, as well as in adjusted models including potential confounding sociodemographic, health-related and dialysis-related predictor variables.

### Results

Data from N=1360 ESKD hemodialysis patients were evaluated (37.1% women, median age 62.4 years). All three proposed PF measures showed highly significant associations with mortality (p<0.01), remaining robust in the adjusted model (p<0.01). HRs for all three PF measures were comparable in the unadjusted model (PPT:0.94[95%CI:0.93-0.95], PROMIS:0.94[95%CI:0.93-0.96], Combined score:0.94[95%CI:0.93-0.95]), and in the adjusted model (PPT:0.96[95%CI:0.94-0.97], PROMIS:0.96[95%CI:0.95-0.98], Combined score:0.96[95%CI:0.94-0.97]), reflecting a 4-5% decrease in the hazard of mortality for each unit increase in PF scores (p<0.01).

### Conclusion

PF is a highly significant and robust predictor of mortality in ESKD hemodialysis patients. PRO and PerfO measures, as well as both measures combined, can be equally utilized as a predictive marker. The non-invasive, easily administered short-form PRO measures provide a simpler cost and time-effective alternative to performance-based measures for clinical practice.

## 103.2 Embedded cognitive debriefing to assess content validity of physical function performance outcome measures in a multi-condition validation study

Courtney Hurt^1^, George Greene^1^, Chelsea Perschon^1^, Xiaodan Tang^1^, Sara Shaunfield^1^, Jin-Shei Lai^1^, John (Devin) Peipert^2^, Maja Kuharic^1^, Emilie Jaeger^1^, Sofia Guzman^1^, Mauricio Andrade^1^, Jack Guralnik^3^, Edward Neilan^4^, Allison Seebald^4^, David Cella^1^

^1^Northwestern University Feinberg School of Medicine, Chicago, Illinois, USA, ^2^University of Birmingham, Edgbaston Birmingham, Birmingham, UK, ^3^University of Maryland School of Medicine, Baltimore, USA, ^4^National Organization for Rare Disorders (NORD), Danbury, USA

*Journal of Patient-Reported Outcomes 2026*, **10(Suppl 1)**:103.2

### Aims

We aimed to evaluate the content validity of seven performance outcome measures (PerfOs) assessing physical function (PF) limitations in individuals with rare diseases and older adults with sarcopenia using a novel, mixed-methods approach. This work contributes to the development of clinical outcome assessments for regulatory use.

### Methods

Cognitive debriefing interviews were conducted with 79 participants enrolled in a psychometric validation study of PROMIS-based PF patient reported outcome items and PF PerfOs, including Balance Tests, Repeated Chair Stand, 4-Meter Gait Speed, Grip Strength, Timed Up and Go, 9-Hole Pegboard, and 6-Minute Walk. Participants included individuals with systemic sclerosis (n=10), myositis (n=10), idiopathic pulmonary fibrosis (n=6), and sarcopenia (n=53). Participants completed each PerfO and were queried using a semi-structured interview guide. Interview data were analyzed to assess clarity and participant comprehension of PerfO instructions, relevance to daily life, and comprehensiveness of the PerfO set. Instruction clarity and participant comprehension was assessed using both participant self-report and test administrator ratings. Relevance was evaluated by asking participants to describe daily activities that reflect the construct measure by each PerfO task, and to indicate whether the task’s perceived difficulty aligned with those real-life activities. Thematic analysis was used to examine open-ended feedback.

### Results

Cognitive debriefing results support the clarity, participant comprehension, relevance, and comprehensiveness of the selected PerfOs across the four conditions. Participants were generally able to link each task to meaningful daily activities affected by their condition. Some variation in perceived difficulty was identified, reflecting the goal of identifying measures that capture a wide range of functional abilities. No additions or changes to the proposed PerfOs were required, supporting the strength of our prior concept elicitation findings and the PerfO selection process conducted in collaboration with the FDA and patient, caregiver, technical expert, and clinician advisors and stakeholders.

### Conclusion

By embedding cognitive debriefing into an early phase of a validation study, we pragmatically and rigorously established the content validity of the candidate PerfOs. Moreover, linking performance tasks to real-world patient experience strengthens the case for these measures as fit-for-purpose core outcome assessments and lays the groundwork for future regulatory qualification.

## 103.5 Identifying patients with delayed physical function recovery after solid organ transplantation using supervised cluster analysis of longitudinal PROMIS® Physical Function scores

Jad Fadlallah^1^, Ana Samudio^1^, Sara Macanovic^1^, Nathaniel Edwards^1^, Istvan Mucsi^1^

^1^University Health Network, Toronto, Ontario, Canada

*Journal of Patient-Reported Outcomes 2026*, **10(Suppl 1)**:103.5

### Aims

Previously we characterized average trajectory of physical function (PF) recovery in solid organ transplant recipients (SOTr). Clinically it is important to identify patients with delayed recovery. We aimed to identify patterns of PF recovery using supervised cluster analysis of longitudinally obtained PROMIS PF scores.

### Methods

Longitudinal convenience sample of adult kidney, kidney-pancreas, and liver transplant recipients who completed PROMIS-PF Computer Adaptive Test (CAT) (higher=better PF) within ~1 week post-transplant and biweekly over 2 months. Based on visual inspection of individual recovery patterns, we identified baseline PROMIS-PF and change between week 0-week 2 as features potentially associated with PF recovery. We stratified participants by baseline PROMIS-PF (>30 vs. ≤30) and by T-score change between baseline and week 2(≥1 vs <1; improved vs. non-responder). Four clusters (EXPOSURE) were identified: CL1(poor baseline– non-responder), CL2(poor baseline–improvement), CL3(better baseline–non-responder),CL4 (better baseline–improvement). We used linear mixed-effects models with random effects for participants and modeled time-by-cluster interactions to assess trajectories. We fit parametric survival models using interval regression to estimate time-to-recovery(T-score≥45 - OUTCOME) followed by Wald-test to evaluate significance of group differences.

### Results

Of 104 participants, 8(8%) were in CL1, 28(27%) in CL2, 56(54%) in CL3, and 22(21%) in CL4. Age, organ type, socioeconomic status, and ethnicity were similar across clusters. CL1 had a higher proportion of females and had significantly worse baseline PROMIS fatigue, pain interference and shortness of breath scores, compared to CL2. Baseline PROMIS scores showed a qualitatively similar pattern for CL3 vs CL4.At 2 months, mean(95% CI) PROMIS-PF scores were: CL1: 29(25–33) vs CL2: 41(39–43)(p<0.001); CL3: 43(41–45) vs CL4: 49(46–52)(p<0.001). The mean(SD) change in PROMIS PF between baseline and week 8 was 3(3) vs 14(8) for CL1 vs CL2; 3(6) vs 11(6) for CL3 vs CL4; p< 0.001 for both. The proportion of individuals who reached recovery event were 0%, 34%, 36%, and 75% in CL1, CL2, CL3 and CL4, respectively(p=0.007).

### Conclusion

Baseline and early PROMIS-PF change identified four distinct recovery clusters among SOTr. No improvement at week 2 predicted delayed PF recovery. This information can help identify patients who might benefit from tailored post-transplant rehabilitation.

**Fig. 1 (abstract 103.5):**
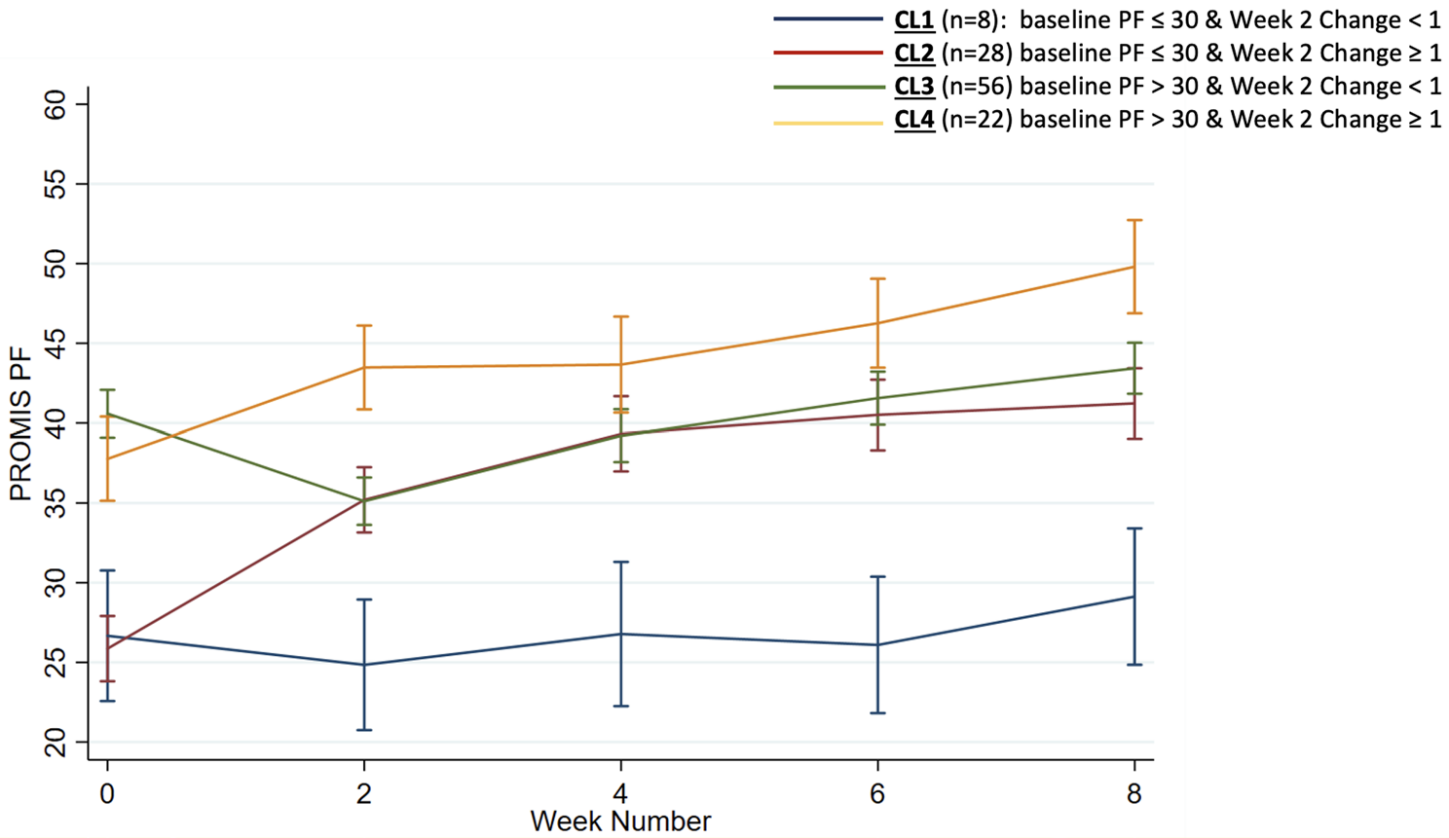
Identifying patients with delayed physical function recovery after solid organ transplantation using supervised cluster analysis of longitudinal PROMIS® Physical Function scores

## 103.4 The Northwestern University Clinical Outcome Assessment Team (NUCOAT) Project: Overview of Development of Cross-Cutting Physical Function Measures for Regulatory Use in Sarcopenia and Rare Disorders

Maja Kuharic^1^, Xiaodan Tang^1^, Courtney N. Hurt^1^, John Devin Peipert^2^, Sara Shaunfield^1^, George Jesus Greene^1^, Jin-Shei Lai^1^, Edward Neilan^3^, Allison Seebald^3^, Chelsea Perschon^1^, Emilie Jaeger^1^, Mauricio Andrade^1^, Sofia Guzman^1^, Jack Guralnik^4^, David Cella^1^

^1^Northwestern University, Chicago, Illinois, USA, ^2^University of Birmingham, Birminingham, UK, ^3^National Organization for Rare Disorders (NORD), Danbury, USA, ^4^University of Maryland School of Medicine, Baltimore, USA

*Journal of Patient-Reported Outcomes 2026*, **10(Suppl 1)**:103.4

### Aims

This abstract provides an overview of the methodological approach of the Northwestern University Clinical Outcome Assessment Team (NUCOAT) project. Through a cooperative agreement with the U.S. Food and Drug Administration (FDA) and in collaboration with the National Organization for Rare Disorders, this project aimed to develop and validate clinical outcome assessments (COAs) of physical function (PF) for regulatory use in age-related sarcopenia and three rare disorders: idiopathic pulmonary fibrosis, systemic sclerosis, and myositis. These COAs include both patient-reported outcome measures (PROs) and performance outcome measures (PerfOs) designed to serve as fit-for-purpose tools in drug development.

### Methods

This initiative followed a sequential, mixed-methods approach. The UG3 Planning Stage (involved identification of target conditions affecting physical function that lack fit-for-purpose measurement tools. The conditions were refined through FDA collaboration, stakeholder input, literature reviews, and preliminary patient interviews. The UH3 Research Stage comprised: (1) Qualitative Phase 1, where 60 participants described PF limitations through concept elicitation interviews; (2) Qualitative Phase 2, where 30 participants evaluated comprehension and relevance of draft items through cognitive interviews; and (3) Validation Phase, where 247 participants (121 with sarcopenia, 126 with rare disorder) completed PROs and PerfOs derived from PROMIS, NIH Toolbox and SPPB across two timepoints with a subset (n=45 from each group) completing additional test-retest visit. Psychometric evaluation assessed reliability, validity and responsiveness.

### Results

The Planning Stage identified conditions representing diverse PF limitations and impacts. The Research Stage yielded data on PF limitations across conditions and informed the selection of 31 PROMIS items from an initial item pool of 165. Cognitive interviews confirmed item clarity, comprehension, and relevance across all four conditions. Preliminary results from the Validation Phase indicate adequate data quality and acceptable test-retest reliability for PROs and PerfOs. Final PRO item selection for the development of overlapping short forms is currently underway.

### Conclusion

Using a structured, multi-phase approach, the NUCOAT project developed cross-cutting COAs that show promise for capturing PF in these populations. Once finalized, these COAs, including overlapping short forms will be made available on HealthMeasures.net to support drug development and broader clinical research and practice.

## 104.2 Developing a person-centered measure for contraceptive side effects

Amelia Mackenzie^1^

^1^FHI 360, Annapolis, Maryland, USA

*Journal of Patient-Reported Outcomes 2026*, **10(Suppl 1)**:104.2

### Aims

We sought to develop a novel patient-reported outcome measure for contraceptive clinical trials to assess the many ways contraception impacts the menstrual cycle, a set of side effects known to impact the satisfaction, wellbeing, and quality of life of contraceptive users. Despite the importance of these outcomes and although regulatory authorities require collection of these data, there is no standard measure used across trials and contraceptive products with evidence of validation in line with best practices and regulator methodological guidance.

### Methods

Prior to initiating measure development, we undertook an iterative approach of convening and consensus-building among global, multidisciplinary experts and stakeholders over five years, including technical consultations, a task force, a community of practice, key informant interviews, formal consensus-building methodologies, and a transdisciplinary systematic review. Building upon that foundation, our investigator team is beginning a multi-phase, multi-site study to develop a patient-reported outcome measure simultaneously in three global regions and in three languages, beginning with concept elucidation and cognitive debriefing along with continued engagement with global experts and stakeholders. In this abstract, we present results from focus group discussions—including abbreviated body mapping (Figure 1), ideation card activities, and other interactive activities—to inform revision of a conceptual model initially developed by a global task force and subsequent item drafting.

### Results

We are finishing data collection and beginning analysis, including high-level transcript coding and interactive affinity mapping exercises with the entire study team. Initial findings include an array of diverse conceptualizations of the impact of contraception on the menstrual cycle using varied terminologies, based on different lived experiences, and including a variety of social, economic, and health impacts of these changes on the lives, wellbeing, and quality of life of participants.

### Conclusion

Standardized and person-centered measurement of data on menstrual cycle side effects in contraceptive clinical trials using a measure with evidence of validation aligned with best practices and regulatory authority guidance can improve future product labeling and permit greater comparability and data synthesis across trials to inform clinical guidance, permitting providers to offer relevant counseling and contraceptive users to make informed decisions about their health, wellbeing, and quality of life.

**Fig. 1 (abstract 104.2):**
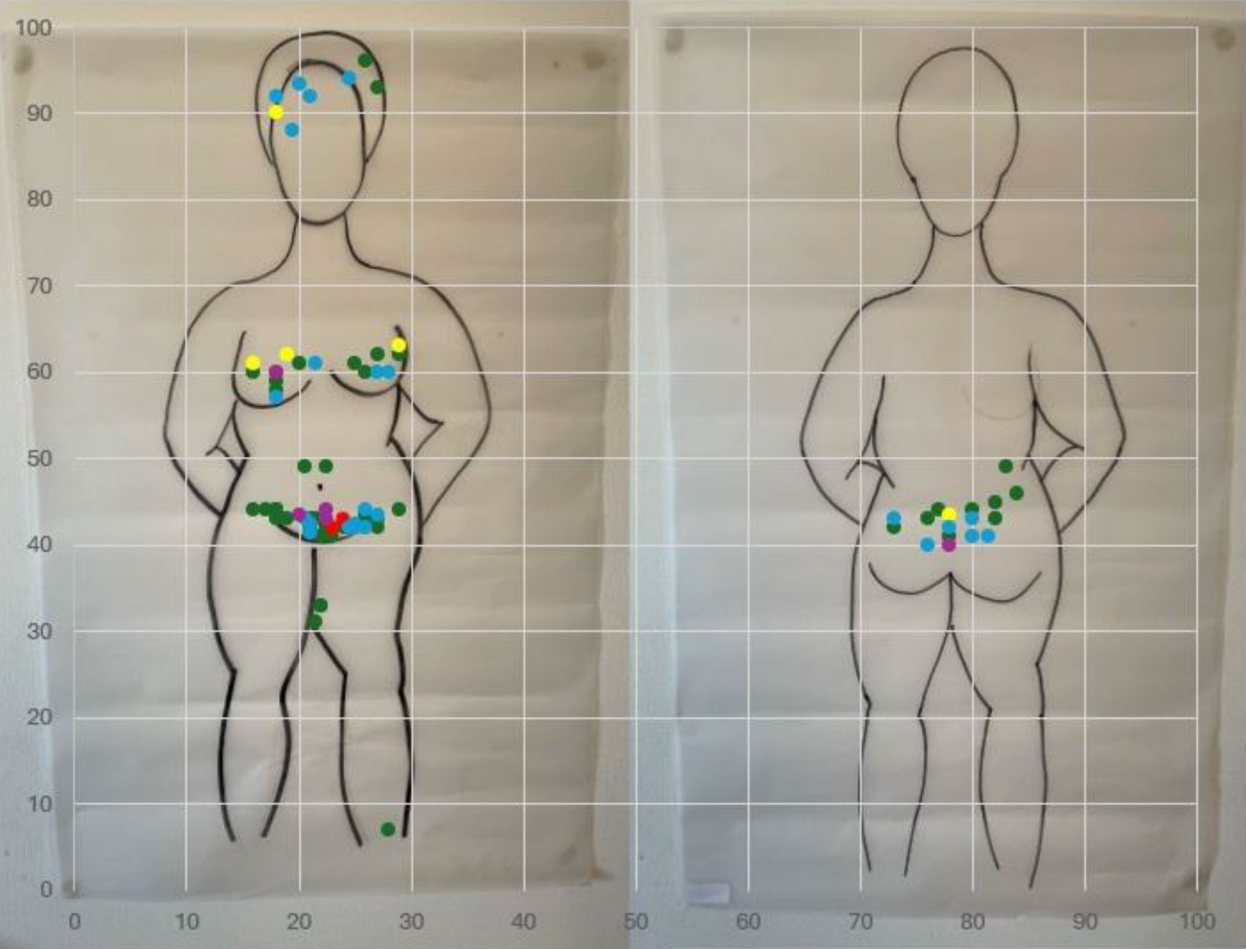
Compiled results across focus group discussions of participant body mapping of their menstrual-associated pain with contraceptive use

## 104.3 Validation of a novel patient-reported outcome instrument assessing atopic dermatitis-associated xerosis in patients with skin of color

Christopher Hartford^1^, Carl Cooper^2^, Donald M. Bushnell^2^, Jason Wang^1^, Brad Shumel^1^, Chien-Chia Chuang^3^, Andrew Alexis Weill^4^, Diana Rofail^1^

^1^Regeneron Pharmaceutical Inc., Tarrytown, New York, USA, ^2^Evidera, Wilmington, North Carolina, USA, ^3^Sanofi, Cambridge, Massachusetts, USA, ^4^Cornell Medical College, New York, USA

*Journal of Patient-Reported Outcomes 2026*, **10(Suppl 1)**:104.3

### Aims

Xerosis in Atopic Dermatitis (X-AD), a newly developed patient-reported outcome instrument, evaluates improvements in the severity and bother of xerosis (dry skin) in patients with AD. We evaluated psychometric properties (reliability, validity, and responsiveness) and generated meaningful score differences (MSDs) for X-AD in patients with skin of color (SoC) and atopic dermatitis (AD).

### Methods

Interim data were analyzed from the phase 4 DISCOVER trial (NCT05590585), an open-label, single-arm study evaluating the efficacy and safety of 24 weeks of dupilumab treatment for moderate-to-severe AD in adolescent/adult patients (≥12 years old) with SoC (Fitzpatrick skin type ≥4), using classical test theory. Psychometric evaluation was only performed for X-AD item 1 (xerosis severity) which consists of a 0–10 NRS assessing worst skin dryness over the past 7 days.

### Results

Data for 123 patients (9.8%/90.2%, adolescents/adults; mean [SD] age 38.1 [17.1] years) were included in the interim dataset, 84 of whom completed X-AD baseline assessments. X-AD item 1 had a test-retest reliability intraclass correlation coefficient (ICC) value of 0.655 for Weeks 2 vs 4 on Patient Global Impression of Disease [PGI-D]. A correlation matrix was generated for baseline convergent validity (PGI-D [r=0.66] and Patient-Oriented Eczema Measure [r=0.63]) and divergent validity (Hospital Anxiety and Depression Scale anxiety [r=0.30], and Hospital Anxiety and Depression Scale depression [r=0.09]). Responsiveness was demonstrated across all anchors tested, with significant improvements from baseline to Week 24. Triangulation of MSD estimates derived from standardized methods indicated that a 4-point change as meaningful MSD is optimal in patients with SoC and moderate-to-severe AD.

### Conclusion

Psychometric analyses confirmed X-AD item 1 was a valid and responsive measure of self-reported xerosis severity in patients with SoC and moderate-to-severe AD. This evidence supports the X-AD as a fit-for-purpose assessment of xerosis in patients with AD and SOC, fulfilling a key gap.

## 104.5 Development of a Nontuberculous Mycobacterial Symptom Scale (NTM-SS): Psychometric Analysis and Proposed Scale

Emily Henkle^1^, Nathan Dieckmann^2^, Heather Franklin^2^, David Cella^3^, Charles Daley^4^, Patrick Flume^5^, Pamela McShane^6^, Kevin Winthrop^1^, Alexandra Quittner^7^

^1^OHSU-PSU School of Public Health, Portland, Oregon, USA, ^2^OHSU School of Nursing, Portland, Oregon, USA, ^3^Northwestern Feinberg School of Medicine, Chicago, Illinois, USA, ^4^National Jewish Health, Denver, Colorado, USA, ^5^Medical University of South Carolina, Charleston, South Carolina, USA, ^6^University of Texas Health Science Center, Northeast, Tyler, USA, ^7^Nicklaus Childrens Research Institute, Miami, USA

*Journal of Patient-Reported Outcomes 2026*, **10(Suppl 1)**:104.5

### Aims

Nontuberculous mycobacterial pulmonary disease (NTM-PD) is a rare, chronic infection that often necessitates long-term, multidrug treatment. No fully validated patient-reported outcome measure (PROM) exists for use as a clinical trial endpoint, despite active drug development programs. Here we report preliminary psychometric analyses of a novel disease-specific PROM.

### Methods

NTM Symptom Scale (NTM-SS) items were selected using a detailed conceptual framework developed from literature review, input from patients and experts, and cognitive testing. Eligible patients met NTM-PD disease criteria (clinic-enrolled) or self-reported NTM-PD (remote-enrolled) with symptoms. Electronically via REDCap patients completed the draft NTM-SS (n=44 items, 5-item Likert frequency scale), GAD-7/PHQ-8, QOL-Bronchiectasis Respiratory Symptoms scale, PROMIS Fatigue SF7a/Cognitive Function/Sleep Disturbance 4a, PRO-CTCAE Appetite items, and global impression of severity. We used exploratory factor analysis and item response theory (comparing Bayesian Information Criteria [BIC] of the one-parameter graded response model vs. two-parameter model), in combination with prior qualitative findings, to refine the NTM-SS. We evaluated item quality using item characteristic curves, total test information function, and theoretical relevance/face validity. We then evaluated validity and reliability.

### Results

Overall, 230 people living with NTM-PD participated; 45 repeated the NTM-SS 10–14 days later. Eight factors with eigenvalues > 1 were extracted (65% of variability, RMSEA=.08, RMSR=.04). We dropped 9 items due to low factor loading or a 2-item factor that lacked face validity. Correlations between the retained 7 factors were .09-.59 justifying an oblique rotation. IRT modeling suggested good fit for all 7 subscales after 3 additional items were dropped in the iterative process. The proposed NTM-SS subscales (Figure) included 4–7 items each (32 total items). Scale reliability ranged from 0.95 (Cognitive Functioning, Fatigue) to 0.85 (Cough and Congestion). Each item was averaged and domain scores were scaled 0-100, 0=best/least frequent and 100=worst/most frequent. Test-retest reliability was high, with correlations between r=0.82-0.88, except Sleep Quality (r=0.73). The NTM-SS subscale showed strong convergent validity with existing scales (correlations 0.64-0.87).

### Conclusion

We identified a disease-specific PROM with a highly reliable set of 7 correlated NTM-SS subscales. Subscales are scored separately, allowing for selection of appropriate clinical trial endpoints. Further validation including responsiveness to treatment is needed.

**Fig. (abstract 104.5):**
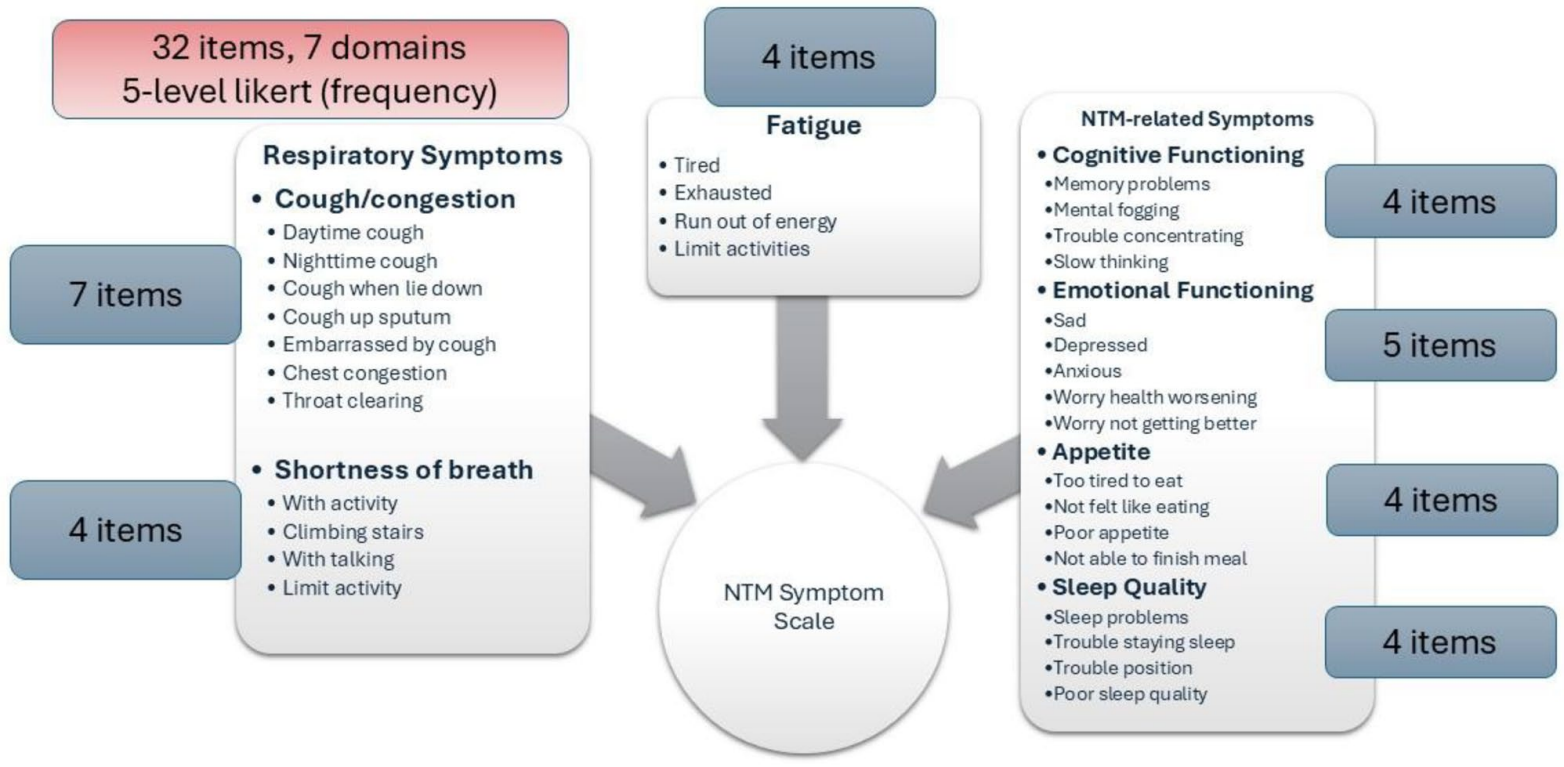
NTM-SS conceptual framework with 7 relevant and reliable domains based on exploratory factor analysis and item response theory

## 104.4 Psychometric properties of cognition bolt-ons for the EQ-5D-3L and EQ-5D-5L: a systematic review

Fanni Rencz^1^, Stevanus Pangestu^2^, Brendan Mulhern^3^, Aureliano Paolo Finch^4^, Mathieu F. Janssen^5^

^1^Corvinus University of Budapest, Department of Health Policy & EuroQol Research Foundation, Budapest, Hungary, ^2^Department of Health Policy and Doctoral School of Business and Management, Corvinus University of Budapest, Budapest, Hungary, ^3^Centre for Health Economics Research and Evaluation, University of Technology Sydney, Sydney, Australia, ^4^EuroQol Research Foundation, Rotterdam, Netherlands, ^5^Erasmus MC, Department of Psychiatry, Section Medical Psychology and Psychotherapy, Rotterdam, Netherlands

*Journal of Patient-Reported Outcomes 2026*, **10(Suppl 1)**:104.4

### Aims

Bolt-ons are additional dimensions for the EQ-5D to capture aspects of health-related quality of life not addressed by the five core dimensions. Cognition is the most commonly used EQ-5D bolt-on, with 52 different versions varying in the number of levels (3L vs. 5L), descriptors and examples provided for the construct. We aimed to systematically review the psychometric properties of cognition bolt-ons for the EQ-5D-3L and EQ-5D-5L.

### Methods

A systematic review was conducted in PubMed, Web of Science and Google Scholar following PRISMA 2020 guidelines (PROSPERO: CRD42023445567). Data were extracted on characteristics of the study population, mode of administration and psychometric properties. A checklist based on existing criteria was developed to rate the psychometric properties of each identified bolt-on version in each publication as positive (+) or negative (-), with the scores summed to determine overall performance.

### Results

A total of 101 publications from 72 studies met the inclusion criteria, assessing the psychometric properties of 15 three-level and 13 five-level bolt-ons. The included studies tested bolt-ons across more than 20 different populations, including patients, the general public and caregivers. The most commonly reported psychometric properties were item-level ceiling (n=75) and known-groups validity (n=54). Few studies explored convergent validity (n=8), divergent validity (n=8), responsiveness (n=3), patient-proxy agreement (n=2) and test-retest reliability (n=1), and no studies reported on content validity or differential item functioning. Five-level bolt-ons outperformed three-level bolt-ons in terms of overall performance (3L: 55+/57-; 5L: 45+/28-). The level of supportive psychometric evidence varied across patient populations, e.g. head/brain injury (3L: 11+/11-; 5L: 1+/3-) and dementia (3L: 9+/8-; 5L: 4+/4-). The most tested bolt-ons were those developed by Janssen 2013 (5L: 18+/15-) and Haagsma 2005 (3L: 8+/12-), with fewer than 10 assessments for all other bolt-ons. Several bolt-ons showed good divergent validity from the core EQ-5D items and known-groups validity for relevant clinical and sociodemographic characteristics.

### Conclusion

Despite the large number of publications, the psychometric evidence base remains insufficient to identify a single preferred descriptor for the EuroQol Group to recommend. Future research should focus on testing the psychometric properties of the most promising bolt-on items, both qualitatively and quantitatively, across diverse populations.

## 104.1 The caregiver experience in amyotrophic lateral sclerosis (ALS): a mixed-methods study to examine the lived experience of caring for people living with ALS and to develop a novel ALS Caregiver Impact Questionnaire (ALS-CIQ)

William Nowell^1^, Nadine McGale^2^, Oren Levy^1^, Sarah Wilding^2^, Phoebe Heinrich^2^, Nick Patel^1^, Jinsy Andrews^3^, Diana Rofail^1^

^1^Regeneron Pharmaceuticals, Inc., Tarrytown, New York, USA, ^2^Modus Outcomes, Cambridge, Massachusetts, USA, ^3^Department of Neurology, Colombia University, New York, USA

*Journal of Patient-Reported Outcomes 2026*, **10(Suppl 1)**:104.1

### Aims

This study aimed to develop a conceptualization of the personal experience of caregivers of people living with amyotrophic lateral sclerosis (pALS), and a novel instrument to assess caregiver impact.

### Methods

This mixed-methods study comprised two stages. First, targeted literature reviews were conducted to identify caregiver concepts of interest and impact measures. Draft items were developed to capture the impacts of caring, informed by a preliminary conceptualization of the caregiver experience and conceptual review of general caregiver impact measures. Second, quantitative data collection of caregiver responses to draft items was conducted, followed by semi-structured concept elicitation and cognitive debriefing interviews with caregivers. Conceptualization of the caregiver experience was refined, and mixed-methods analysis of draft items was conducted.

### Results

Based on the caregiver interviews (n=20; Table 1) and targeted literature review, the caregiver experience included concepts relevant to responsibilities associated with providing care for, offering broader assistance to, and advocating for pALS. Impacts included loss of independence (i.e., loss of freedom and/or privacy, inability to leave the pALS or their home), and affected interpersonal relationships, activities, and professional/family life (Figure). An initial set of 38 items to assess caregiver impact was developed based on the preliminary conceptualization, which was reduced and refined through team review. The resulting 22-item draft set was completed by caregivers prior to interviews. This set was named the ALS Caregiver Impact Questionnaire (ALS-CIQ) and was found to be meaningful and relevant. Qualitative analysis of debriefing interviews identified the most/least relevant items (Table 2), overlap between items, and items that were not conceptually relevant to caregivers. Exploratory Rasch measurement theory analysis conducted on all caregivers completing draft items (n=30; Table 1) indicated potential opportunities to improve content validity and response scale of the ALS-CIQ.

### Conclusion

To our knowledge, this is the first study focused on understanding the lived experience of caring for pALS. Caregivers are profoundly impacted, affecting their independence and their ability to engage in activities, work, and other responsibilities. The ALS-CIQ was developed based on empirical evidence from caregivers of pALS. Further research is needed to refine and validate the ALS-CIQ for use in future studies.

**Fig. (abstract 104.1):**
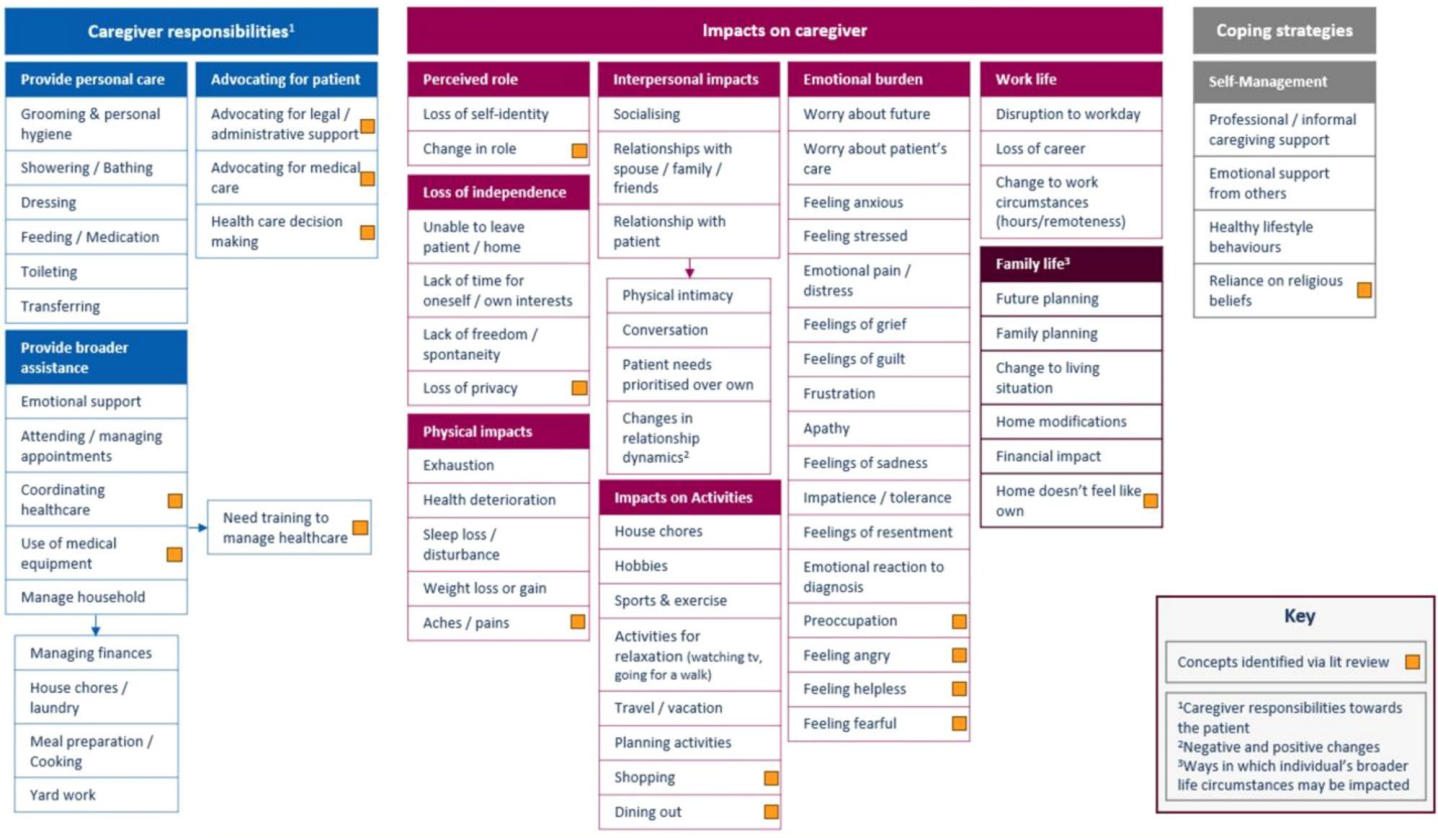
Consolidated conceptual model of the caregiver experience in ALS

**Table 1 (abstract 104.1):**
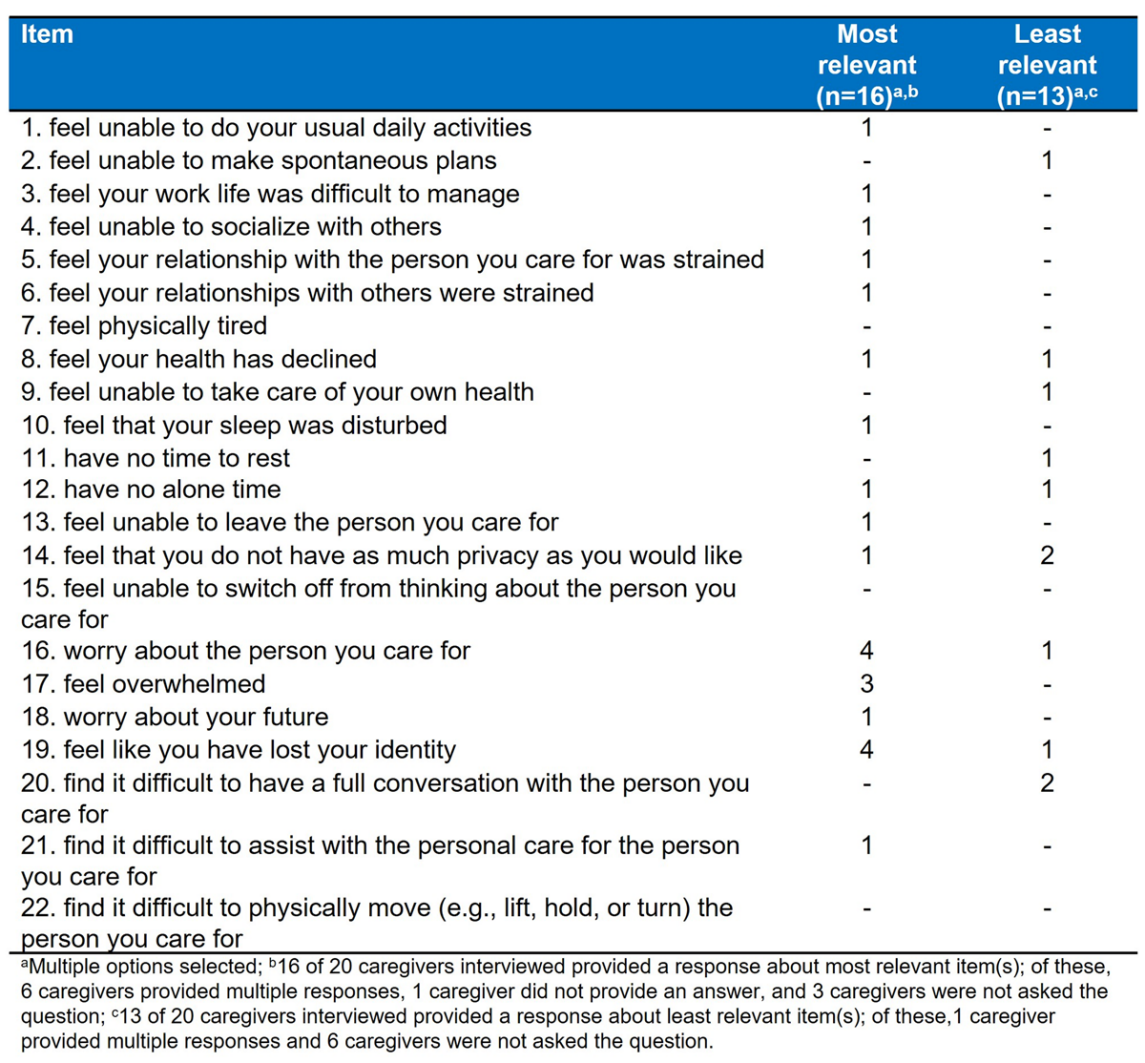
Characteristics of caregivers caring for pALS

**Table 2 (abstract 104.1):**
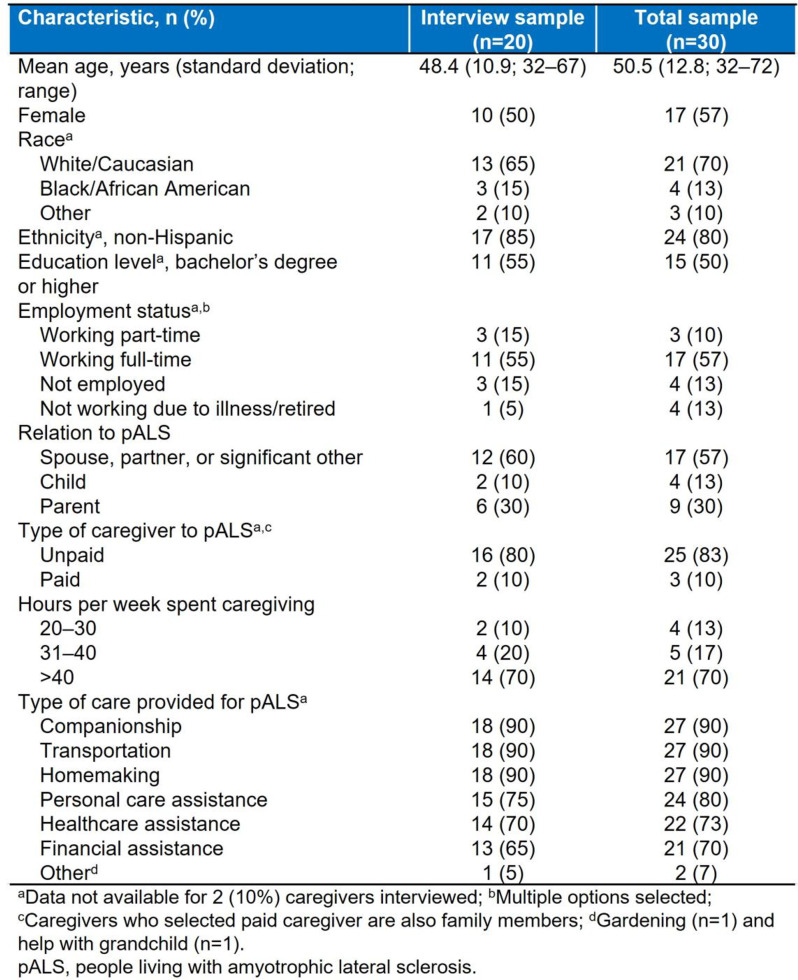
ALS caregiver impact questionnaire item relevance

## 106.4 Development and Validation of the Postoperative Recovery Assessment in Lung Cancer (PRA_Lung): A Modular Patient-Reported Outcome Measure

Cheng Lei^1^, Hongfan Yu^2^, Wen Zhou^2^, Qiuling Shi^2^

^1^Sichuan Cancer Hospital and Institute, Chengdu, China, ^2^State Key Laboratory of Ultrasound in Medicine and Engineering, Chongqing Medical University, Chongqing, China

*Journal of Patient-Reported Outcomes 2026*, **10(Suppl 1)**:106.4

### Aims

This study aimed to develop and validate a modular patient-reported outcome measure (PROM) for assessing postoperative recovery in lung cancer patients, integrating symptom burden and functional interference domains while prioritizing patient-centered perspectives.

### Methods

The three-phase development process included: 1) Qualitative interviews with 45 patients and content analysis of 428 WeChat patient-doctor communication records to establish an initial item pool; 2) Two-round Delphi expert consultation (n=29) for item refinement and draft scale; 3) Cross-sectional data from 451 patients informed the final scale. Psychometric evaluation combined classical test theory (CTT) and item response theory (IRT), with ROC analysis establishing clinical thresholds.

### Results

The final PRA_Lung comprises four modules: Physiological Symptoms (9 items), Psychological Symptoms (4 items), Daily Life Functions (5 items), and Social Functions (5 items). CTT demonstrated strong reliability (Cronbach’s α=0.745-0.823; ICC=0.703-0.814) and validity (item-module correlations r=0.572-0.847; criterion validity r=-0.579 to -0.555; Cohen’s d>0.5 for known-group validity). IRT confirmed unidimensional modules (RMSEA<0.08) with acceptable item parameters (discrimination a=0.16-1.56; difficulty b=-3.82-6.30) and peak precision at θ=-2-4. ROC analysis yielded discriminative thresholds: single-module AUC=0.712-0.796 (cutoffs 2.5-3.8), combined-module AUC=0.765-0.931 (cutoffs 2.7-3.3).

### Conclusion

The PRA_Lung represents a psychometrically robust, modular PROM that enables comprehensive assessment of postoperative recovery in lung cancer patients. Its flexible administration (modular or combined) and validated clinical thresholds enhance utility for personalized care planning and outcome monitoring in clinical practice.

**Fig. 1 (abstract 106.4):**
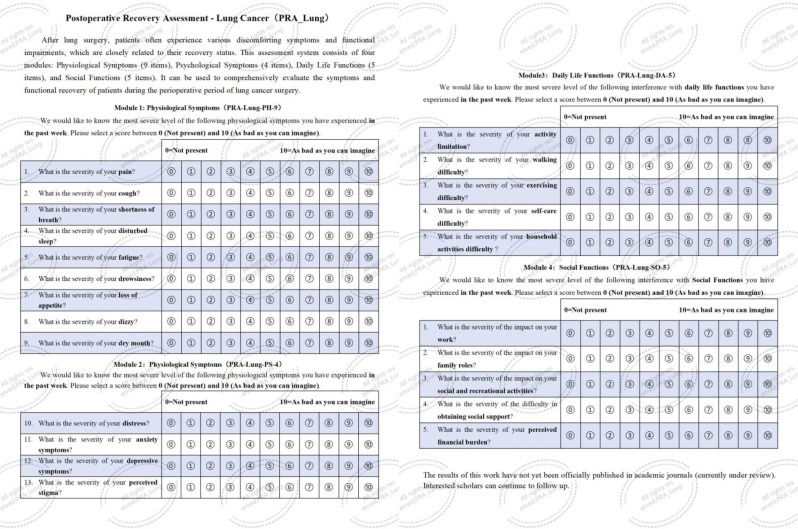
Development and Validation of the Postoperative Recovery Assessment in Lung Cancer (PRA_Lung): A Modular Patient-Reported Outcome Measure

## 106.1 Beyond Treatment Costs: Financial Hardship, Symptom Burden, and Long-term Complications in Head and Neck Cancer Survivors

Eden Brauer^1^, Ya-Chen Tina Shih^1^, Patricia A. Ganz^1^, Maie A. St. John^1^, Deborah J. Wong^1^

^1^UCLA, Los Angeles, California, USA

*Journal of Patient-Reported Outcomes 2026*, **10(Suppl 1)**:106.1

### Aims

Head and neck cancer (HNC) survivors face unique challenges that may contribute to long-term cancer-related financial hardship, including complex treatment regimens, specialized rehabilitation needs, and substantial dental expenses. This study aimed to characterize the prevalence of financial hardship among HNC survivors and examine its association with symptom burden, functional outcomes, and long-term complications.

### Methods

A cross-sectional survey was administered to adults diagnosed with HNC 2–6 years prior, identified through the institutional Tumor Registry. Financial hardship was assessed using the Comprehensive Score for Financial Toxicity (COST) instrument. Validated measures of symptom burden functional outcomes, and treatment-related complications were collected. Open-ended questions collected qualitative data to capture financial impacts on quality of life after treatment.

### Results

Among 347 respondents, 28.4% reported moderate to severe financial hardship. Financial hardship was significantly associated sociodemographic factors including younger age, living situation, and private insurance status, but not clinical factors such as disease stage (p=0.394) or time since diagnosis (p=0.891). Participants reporting higher financial hardship experienced significantly worse symptom burden, including pain, fatigue, depression, and anxiety, as well as poorer physical and cognitive function. Higher financial hardship was also significantly associated with increased prevalence of post-treatment swallowing problems, mouth pain, limited mouth opening, dental complications, and polypharmacy. Qualitative responses revealed challenges related to employment disruption, insurance limitations, and costly out-of-pocket expenses for post-treatment dental and rehabilitation services.

### Conclusion

HNC survivors face cancer-related financial challenges. Financial hardship is strongly associated with worse symptom burden and functional outcomes. Our findings suggest a potential cycle where treatment-related symptoms impact employment and finances, which in turn affect patients’ ability to access needed supportive care. Comprehensive approaches to address financial toxicity should be integrated into survivorship care for this vulnerable population.

**Fig. 1 (abstract 106.1):**
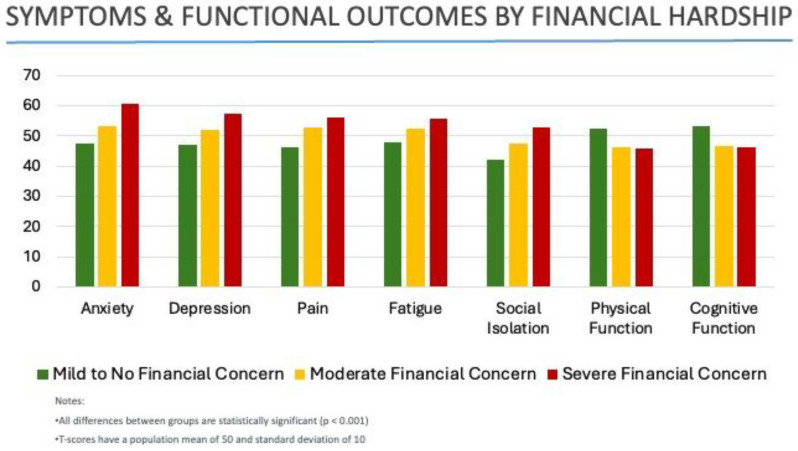
Beyond Treatment Costs: Financial Hardship, Symptom Burden, and Long-term Complications in Head and Neck Cancer Survivors

## 106.3 Experience of symptoms related to dysarthria in patients with nasopharyngeal carcinoma during radiotherapy: A descriptive qualitative study

Fan Li^1^, Lingling Xiong^1^, Cheng Lei^2^, Hongyao Leng^3^, Qiuling Shi^4^, Li Tang^1^

^1^Chongqing General Hospital, Chongqing, China, ^2^Sichuan Clinical Research Center for Cancer, Sichuan, China, ^3^Children’s Hospital of Chongqing Medical University, Chongqing, China, ^4^Chongqing Medical University, Chongqing, China

*Journal of Patient-Reported Outcomes 2026*, **10(Suppl 1)**:106.3

### Aims

Radiotherapy is the most effective treatment for patients with nasopharyngeal cancer. Radiation to the adjacent parts of the tumor and radiation to the larynx may lead to voice changes and the development of dysarthria, however, dysarthria is often overlooked compared to other complications. It is necessary to understand the experience of nasopharyngeal cancer patients with symptoms related to dysarthria after radiotherapy.

### Methods

This qualitative descriptive study enrolled 33 patients NPC radiotherapy patients who experienced dysarthria were recruited from May to August 2024. Data were collected using semi-structured interviews. The interviews were audio-recorded and converted verbatim into standard text, and the data were iteratively thematically analyzed.

### Results

Changes in speech and language quality after radiotherapy for nasopharyngeal carcinoma are common, but there are differences in the degree of symptoms perceived by patients. At the same time, dysarthria is often accompanied by other diverse oropharyngeal symptoms, and the trajectory-varying nature of these symptom experiences imposes a dual physical and psychological burden on patients. Lack of awareness of dysarthria and inadequate emotional support may lead to very different coping styles and a desire for professional ongoing voice management.

### Conclusion

This study helps to elucidate the current status of dysarthria faced by patients undergoing radiotherapy for nasopharyngeal cancer and provides multiple dimensions of dysarthria assessment and management goals for quantitative research. We call attention to the need for healthcare professionals to pay attention to patients’ perspectives and related needs and to develop targeted management strategies that match patients’ needs, and we emphasize the importance of continuity of care to effectively improve dysarthria-related symptoms.

**Fig. 1 (abstract 106.3):**
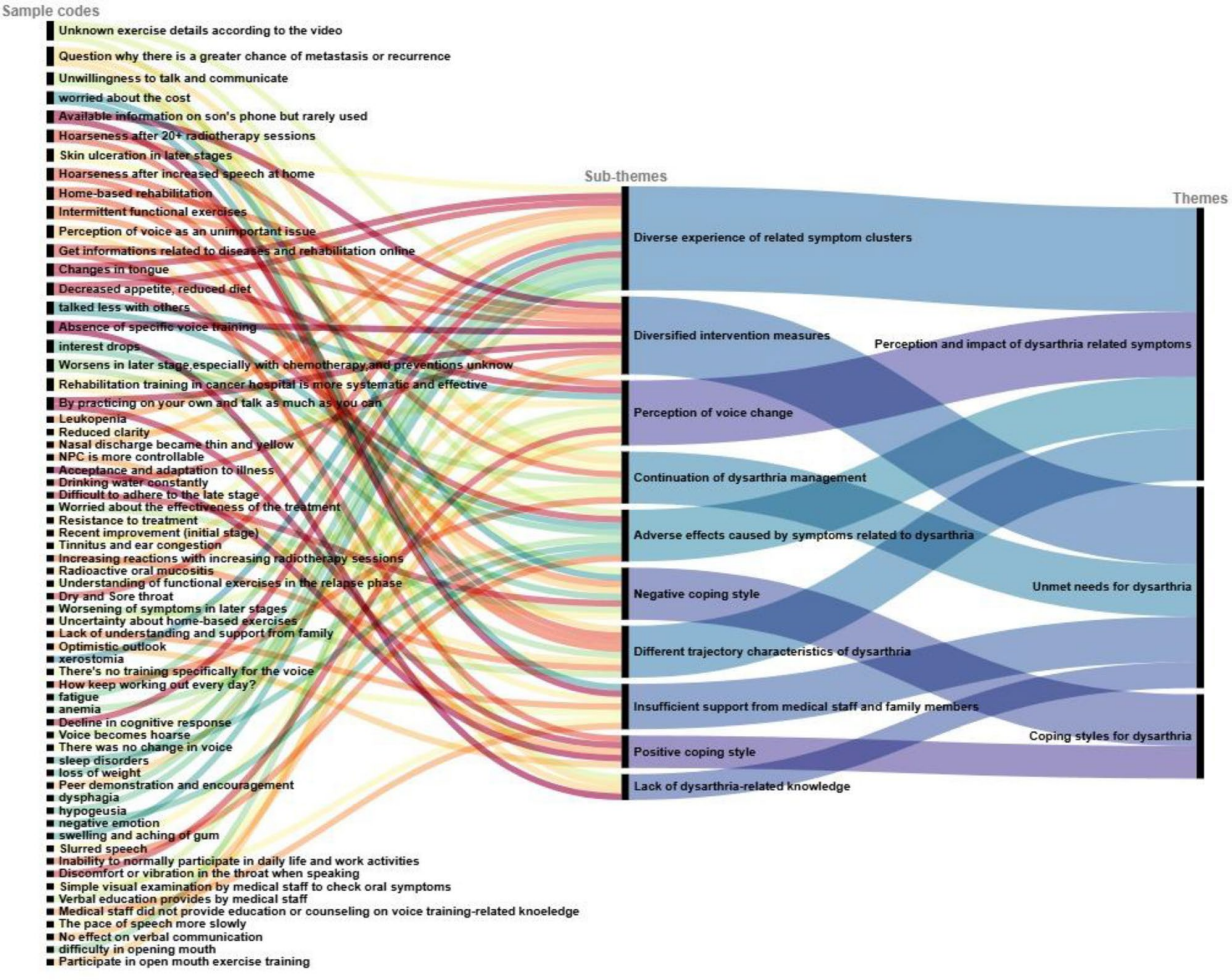
Experience of symptoms related to dysarthria in patients with nasopharyngeal carcinoma during radiotherapy: A descriptive qualitative study

## 106.5 Reliability and validity tests of the ISA Lung Symptom Scale reported by patients undergoing immunotherapy for lung cancer

Xiangyu Tan^1^, Qiuling Shi^1^, Jingyu Zhang^1^

^1^ChongQing Medical University, Chongqing, China

*Journal of Patient-Reported Outcomes 2026*, **10(Suppl 1)**:106.5

### Aims

This study aimed to develop and validate the ISA-Lung scale, designed to quantify symptom burden in lung cancer patients undergoing immunotherapy.

### Methods

The ISA-Lung symptom scale was developed through a combination of a preliminary qualitative study and a comprehensive literature review. The scale was subsequently used to assess patients meeting inclusion and exclusion criteria at multiple time points: day 1, day 3, day 5, week 1, week 2, and week 3 of each treatment cycle. The reliability and validity of the scale were also assessed. Inclusion criteria included: (1) aged 18–80 years, any gender; (2) confirmed diagnosis of lung cancer based on clear pathological or clinical criteria; (3) scheduled to receive immunotherapy; (4) voluntarily provided written informed consent. Internal consistency was evaluated using Cronbach’s alpha. Split-half reliability was assessed with Spearman’s correlation coefficient for odd-even groupings, while test-retest reliability was evaluated using the ICC coefficient. Criterion validity was assessed by comparing ISA-Lung scores with MDASI-inference and EQ-5D quality of life scores. Inter-item associations were analyzed through hierarchical cluster analysis based on Ward’s correlation distance.

### Results

A total of 203 patients (166 males [81.8%], median age 62) were included in the study. The majority of patients (93.1%) had an ECOG score of ≤1, likely reflecting the high proportion of newly diagnosed patients (60.6%). A total of 3,264 ISA-Lung assessments were completed by the patients. Cronbach’s alpha for individual items ranged from 0.749 to 0.772, with an overall Cronbach’s alpha of 0.772. Split-half reliability (Spearman’s correlation coefficient) was 0.7911. Test-retest reliability (ICC) ranged from 0.480 to 0.881, with an overall ICC of 0.833. The criterion validity of the ISA-Lung scale, compared to MDASI-inference and EQ-5D-qol scores at each time point, ranged from 0.528 to 0.765 and -0.421 to 0.534, respectively. Hierarchical cluster analysis revealed strong associations between the following pairs of symptoms: oral mucosal pain and edema, eye discomfort and dry mouth, poor appetite and fatigue, sleep disturbances and cough, chest tightness and shortness of breath, rash and itching.

### Conclusion

The ISA-Lung Scale has decent reliability and validity in assessing symptom burden in lung cancer patients receiving immunotherapy.

**Table 1 (abstract 106.5):**
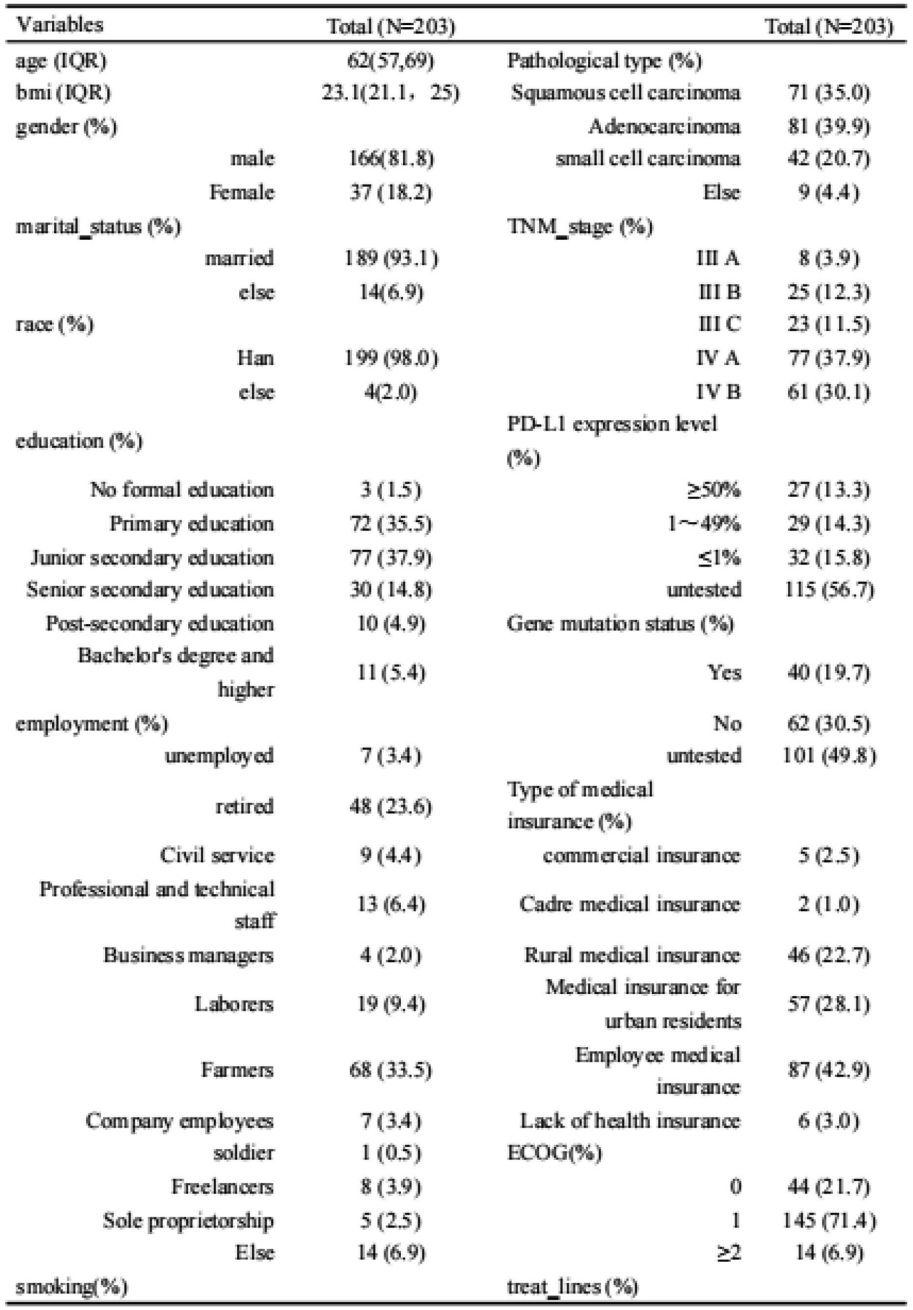

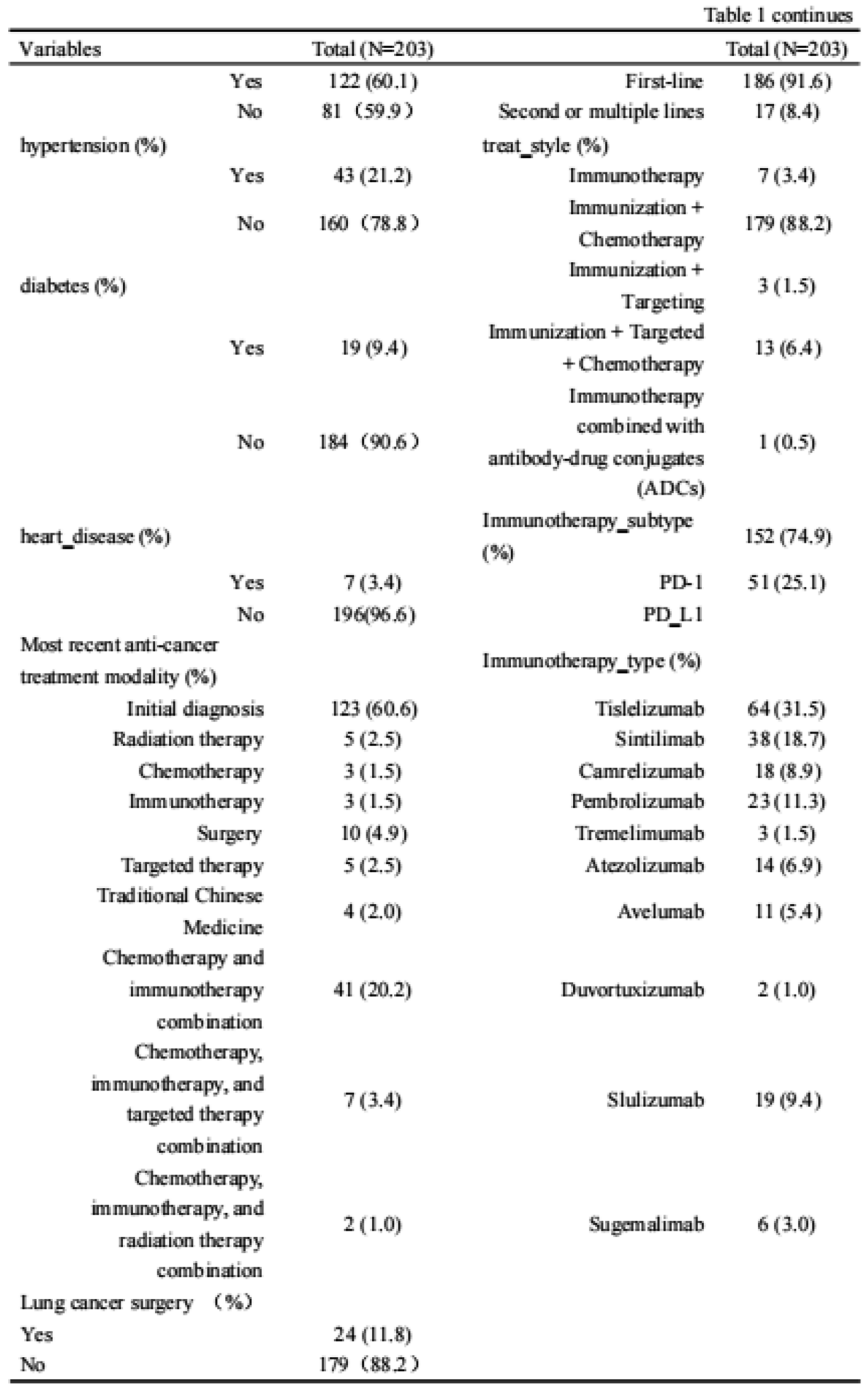
Demographic and clinical characteristics of participants

**Fig. 1 (abstract 106.5):**
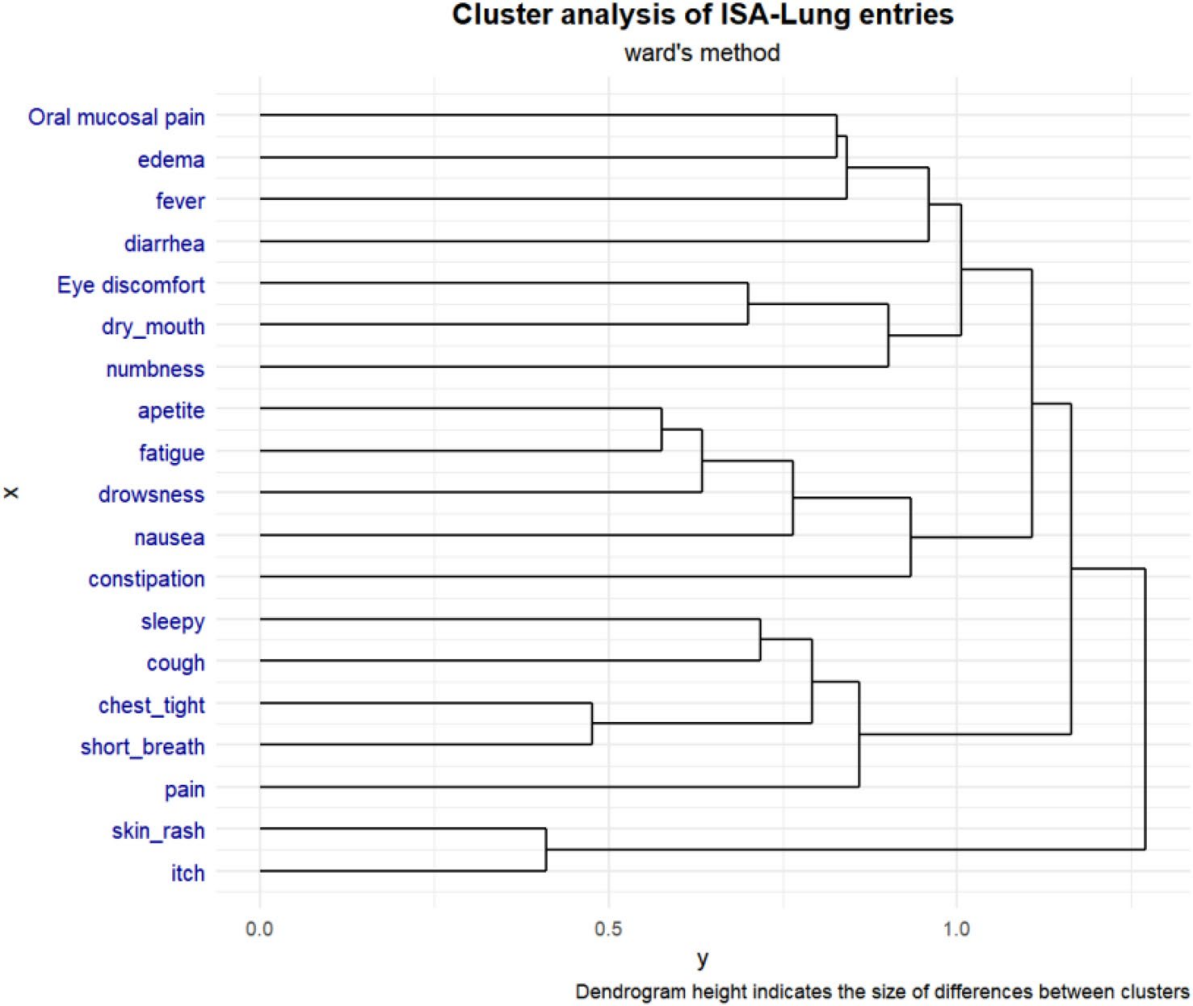
Reliability and validity tests of the ISA Lung Symptom Scale reported by patients undergoing immunotherapy for lung cancer

## 106.2

**Network Analysis of Symptom Clusters in Head and Neck Cancers**

Xiaodan Tang^1^, Jin-Shei Lai^1^, Krithika Suresh^2^, Nan Rothrock^1^, David Cella^1^, Donna Edwards^2^, Michelle Mierzwa^2^, Laila Gharzai^3^

^1^Northwestern University Feinberg School of Medicine, Chicago, Illinois, USA, ^2^University of Michigan, Ann Arbor, Michigan, USA, ^3^Northwestern Memorial Hospital, Northwestern University Feinberg School of Medicine, Chicago, USA

*Journal of Patient-Reported Outcomes 2026*, **10(Suppl 1)**:106.2

### Aims

Patients with cancer often experience co-occurring symptoms throughout the disease/treatment continuum, which is often referred as “symptom clusters”. In this study, we applied network analysis to study how co-occurring symptoms are interconnected. This approach can help researchers identify symptom clusters to understand why they co-occur, and facilitate target intervention that address core symptoms to influence related ones.

### Methods

This is a secondary analysis of a prospective surveillance study of patients with human papillomavirus-related oropharyngeal cancer that included the completion of monthly patient-reported outcomes (EORTC QLQ-C30 and HN35). A Pairwise Markov Random Field (PMRF) model, an undirected graphical network adhering to the Markov property, was applied to analyze symptom connections. The Walktrap algorithm was used to identify clusters within the network.

### Results

We collected 630 assessments from 63 patients over ten months (2021-2022), all of whom received radiotherapy or surgery between 2018 and 2021. Among the 24 symptom subscale scores, core symptoms included fatigue, sticky saliva, pain, and problems with speech, sexuality, social eating, and teeth. In the network graph (Figure 1), node size represents connection strength based on the number and intensity of connections with other symptoms, aiding in identifying core symptoms, while color indicates cluster membership. Red edges indicate negative relationships, while green indicates positive ones. The clusters included three major symptom clusters: a fatigue and speech cluster (orange), an oral and swallowing symptoms cluster (green), and a gastrointestinal and sexuality cluster (yellow). The “open mouth” symptom (grey) did not cluster with any of these groups. Network stability was supported by a reliable CS-coefficient of 0.59 for node strength.

### Conclusion

This exploratory study identified three major symptom clusters in head and neck cancer. Network analysis was used to uncover associations between symptoms, offering insights that could inform more effective symptom management. For example, interventions targeting the oral/swallowing cluster might integrate public or social eating strategies into standard speech-language pathology visits, which traditionally focus on dysphagia and xerostomia. Future research should assess the replicability of these findings and can use this study as a foundation to inform the development of AI-driven models for predicting symptoms, quality of life, survival, and treatment response.

**Fig. 1(abstract 106.2):**
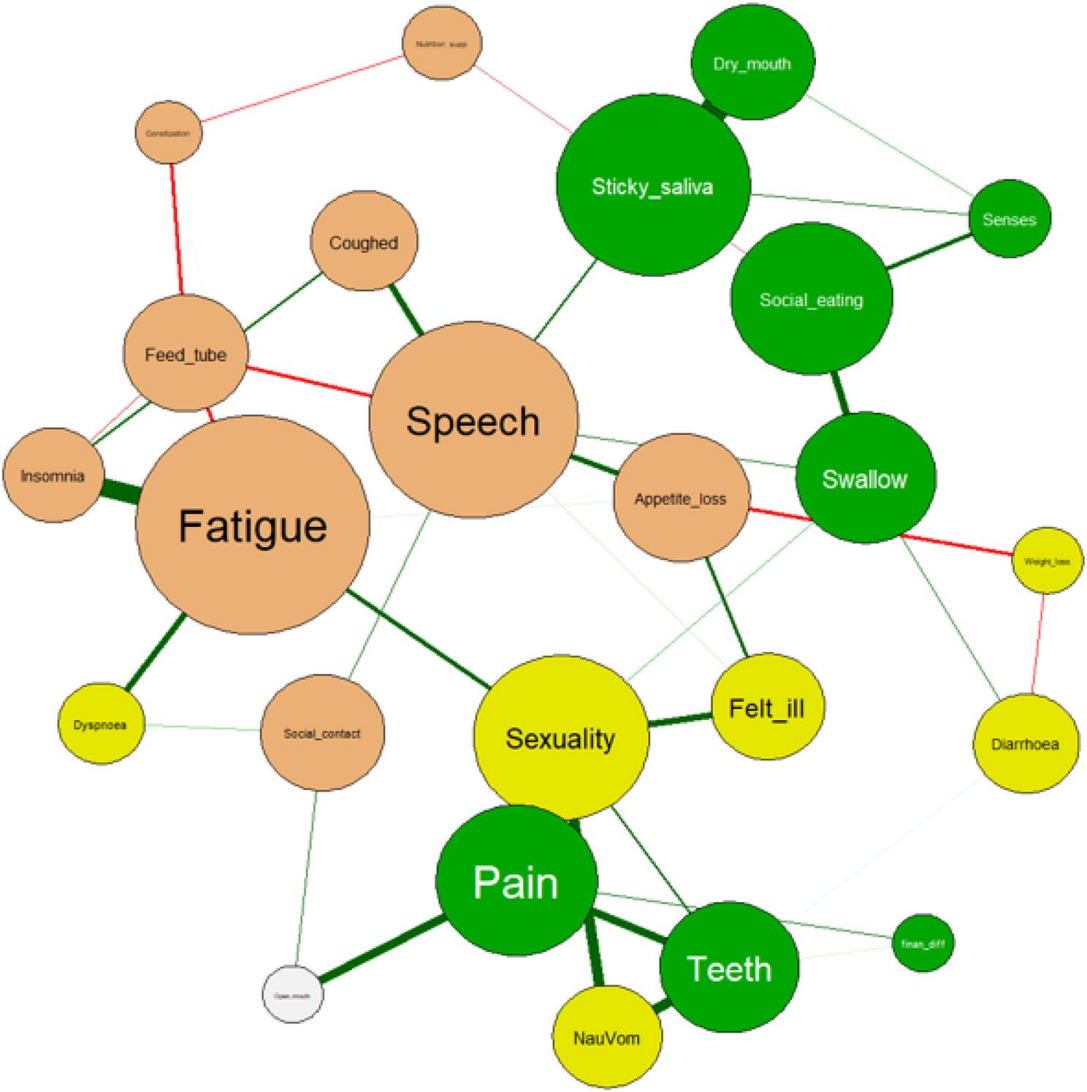
Network of symptom clusters in Head and Neck Cancers. Note. Node size represents connection strength based on the number and intensity of connections with other symptoms. Color indicates cluster membership. Red edges indicate negative relationships. Green indicates positive relationships

## 107.4 Evaluating patient reported outcome measures (PROM) implementation initiatives in cancer care organizations: A systematic review and recommendations for standardizing terminology

Charles Belden^1^, Nicole Henderson^2^, Angela Stover^1^

^1^University of North Carolina at Chapel Hill, Chapel Hill, North Carolina, USA, ^2^University of Alabama at Birmingham, Birmingham, Alabama, USA

*Journal of Patient-Reported Outcomes 2026*, **10(Suppl 1)**:107.4

### Aims

There is growing interest in implementing patient-reported outcome measures (PROMs) as a standard of care, but optimal evaluation methods are needed. We conducted a systematic review of how healthcare organizations have evaluated oncology PROM implementation initiatives.

### Methods

We systematically searched keywords and MeSH terms for articles (English or translated) in three databases (PubMed, Scopus, PsycINFO) through June 2024. Inclusion criteria were cancer organizations administering PROMs with clinician review as the standard of care. There were no restrictions by year, study design, nor cancer or treatment type. Exclusion criteria were inpatient care and effectiveness trials. Six coders independently reviewed entries. Methodological quality was assessed with SPIRIT-PRO and the Standards for Reporting Implementation Studies (StaRI).

### Results

Our search yielded 13,237 unique titles and 12,837 were excluded, leaving 400 for full-text review. Here, we report on 26 late implementation studies (Table 1), operationalized as occurring in at least two clinics from the same healthcare organization or scaled up to multiple organizations. PROM implementation studies were conducted in Australia, Belgium, Canada, Denmark, France, Germany, Netherlands, Spain, UK, and US. Patients completed the PROM for care delivery at each clinic visit (n=12 [46%]), remotely between visits daily, weekly, or monthly with alerts to clinicians (n=5 [19%]), both (n=3 [12%]), other (n=4 [15%]), or not reported [n=2 [8%]). PROM administration modes included electronic only (n=13 [50%]), paper only (n=2 [19%]), multiple modes (n=11 [38%]). Despite being a best practice, few studies reported that PROMs were integrated into the electronic medical record system (n=4 [15%]). Although terminology varied widely, the implementation constructs used by healthcare organizations mapped onto the implementation science frameworks of RE-AIM 3.0 and Proctor’s outcomes 2.0, including acceptability (patient and provider-level), adoption (provider-level), appropriateness (patient and provider-level), cost-effectiveness, effectiveness, reach, and maintenance/sustainment. Patient reach ranged from 37% to 95%, but equitable reach among underserved groups was rarely examined (n=19 [38%]).

### Conclusion

Methodological quality could be improved for evaluating PROM implementation initiatives in healthcare settings. Evaluation metrics and operational definitions varied greatly across healthcare organizations and implementation stages, making comparisons difficult. To guide future work, we present recommendations for standardized terminology by implementation stage (Figure 1).

**Fig. 1 (abstract 107.4a):**
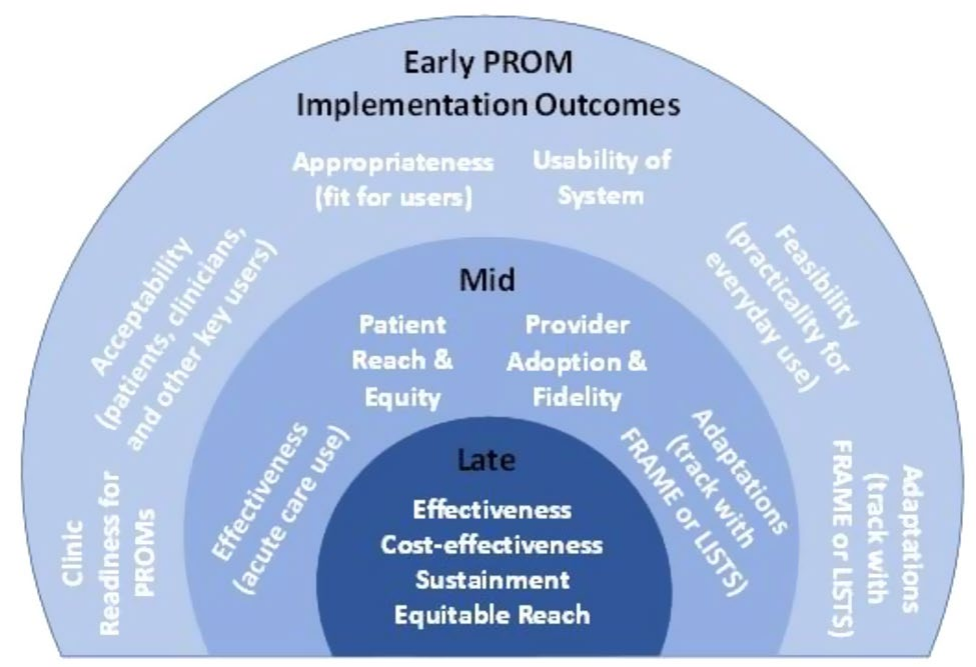
PROM Implementation Outcomes by Implementation Stage

**Fig. 1 (abstract 107.4b):**
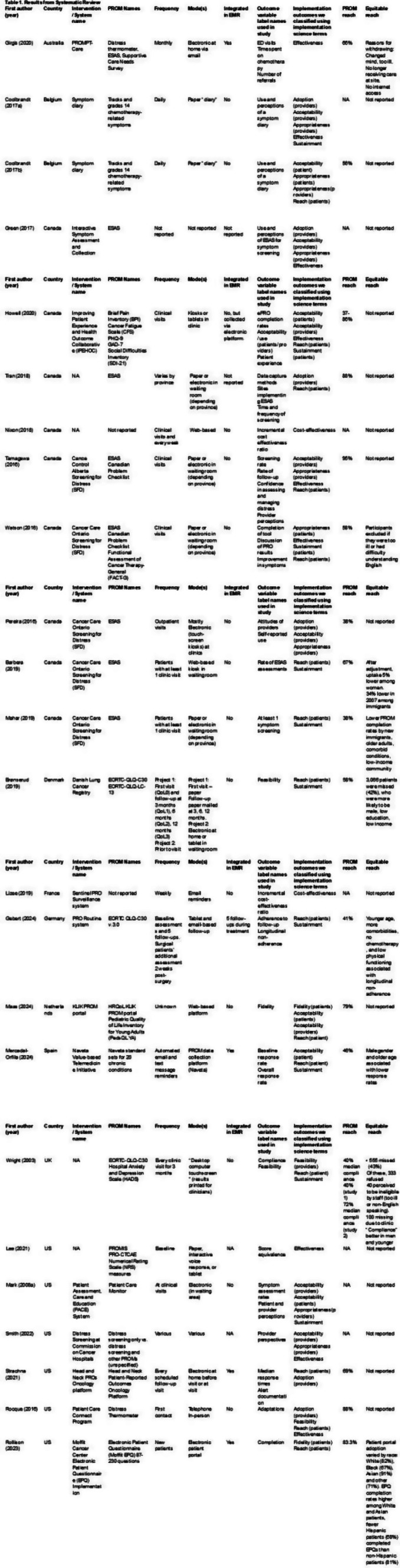
Evaluating patient reported outcome measures (PROM) implementation initiatives in cancer care organizations: A systematic review and recommendations for standardizing terminology

## 107.1 Bridging the gap: A conceptual framework for integrating Patient Reported Outcome Measures throughout the process of Shared Decision Making in clinical care

Anouk Groenewegen^1^, Maud van Muilekom^1^, Hedy van Oers^1^, Marij Hillen^2^, Ellen Smets^2^, Dirk Ubbink^3^, Glyn Elwyn^4^, Anne Stiggelbout^5^, Trudy van der Weijden^6^, Anna Beurskens^6^, Olga Damman^7^, Lotte Haverman^1^

^1^Department of Child and Adolescent Psychiatry and Psychosocial Care, Amsterdam UMC, University of Amsterdam, Emma Children’s Hospital, Amsterdam, The Netherlands, ^2^Department of Medical Psychology, Amsterdam UMC, University of Amsterdam, Amsterdam, The Netherlands, ^3^Department of Surgery, Amsterdam UMC, University of Amsterdam, Amsterdam, The Netherlands, ^4^Coproduction Laboratory, Dartmouth Institute of Health Policy and Clinical Practice, Hanover, USA, ^5^Department of Biomedical Data Sciences, Leiden University Medical Center, Leiden, The Netherlands, ^6^Department of Family Medicine, Maastricht University, Maastricht, The Netherlands, ^7^Department of Public and Occupational Health, Amsterdam UMC, Vrije Universiteit Amsterdam, Amsterdam, The Netherlands

*Journal of Patient-Reported Outcomes 2026*, **10(Suppl 1)**:107.1

### Aims

Patient Reported Outcomes Measures (PROMs) and Shared Decision Making (SDM) are both embraced in attempts to facilitate patient-centred care. Increasingly, calls are made to integrate patients (individual) PROM results within the process of SDM. However, SDM is known to be complex, and it remains largely unclear how this integration should occur exactly. Therefore, we developed a conceptual framework to illustrate how PROM results can be integrated throughout the SDM process in a clinical encounter between a clinician and a patient.

### Methods

In spring 2023, we established a collaboration between PROM- and SDM-experts, to initiate the development of the framework. We used a four-component SDM model (based on Stiggelbout et al. (2015) and Elwyn et al. (2017)) as a basis, to integrate PROM results within the SDM process. A mixed-method design was used, consisting of the following four phases:Identifying existing knowledge and views about the integration of PROM results throughout SDM, via a literature scan, two in-house expert meetings (N=13), and two interactive sessions held during international gatherings with researchers and healthcare workers (N±100) during a symposium and webinar on PROMs and SDM;Design of the conceptual framework by the core project group (N=4) in collaboration with a professional design company;Reviewing the framework by additional experts from the project group (N=5);Finalizing the framework.

### Results

We developed a six-component framework, including four core SDM components and one preceding (‘Signaling and Monitoring’) and one succeeding (‘Action plan and Evaluation’) component specific to preparing the clinical encounter and evaluating treatment decisions. PROM results were integrated in the components ‘Signaling and Monitoring’, ‘Options’, ‘Preferences’, and ‘Action plan and Evaluation’. The framework also specifies key conditions and caveats for integrating PROM results in SDM. We will present the framework at the conference.

### Conclusion

We used a multi-step approach to develop the first PROMxSDM conceptual framework, involving various stakeholders. The framework will be publicly available to support dissemination through training sessions, scientific and clinical events, and communication platforms. The next step is the dialogue between clinicians, patients, researchers, and policy makers, to identify what is needed for its uptake in practice.

## 107.3 Patient education on patient-reported outcome measure completion in clinical care settings: a scoping review

Anouk Groenewegen^1^, Olivia Biller^2^, Joanne Greenhalgh^3^, Andria Joseph^4^, Cecilie Lindström Egholm^5^, Joost Daams^6^, Maria Santana^7^, Lotte Haverman^1^, Maud van Muilekom^1^, Elizabeth Unni^8^

^1^Department of Child and Adolescent Psychiatry and Psychosocial Care, Amsterdam UMC, University of Amsterdam, Emma Children’s Hospital, Amsterdam, The Netherlands, ^2^Johnson & Johnson, Raritan, New Jersey, USA, ^3^University of Leeds, School of Sociology and Social Policy, Leeds, UK, ^4^York Health Economics Consortium (YHEC), University of York, York, UK, ^5^REHPA, The Danish Knowledge Centre for Rehabilitation and Palliative Care, Odense University Hospital, Nyborg, Denmark, ^6^Amsterdam UMC location University of Amsterdam, Medical Library, Research Support, Amsterdam, The Netherlands, ^7^University of Calgary, Department of Community Health Sciences, Calgary, Alberta, Canada, ^8^Touro College of Pharmacy, Department of Social, Behavioral and Administrative Sciences, New York, USA

*Journal of Patient-Reported Outcomes 2026*, **10(Suppl 1)**:107.3

### Aims

Patient-reported outcome measures (PROMs) are increasingly used in clinical care, and evidence demonstrates they enhance patient care processes and outcomes. However, low PROM completion rates can limit the use of PROMs for patient-level monitoring, clinical decision making, and can bias aggregated data. A potential method to overcome barriers in PROM completion is patient education, yet little is known about what instruction, if any, is offered. Therefore, the aim of this scoping review was to understand if and how education is provided to patients on PROMs in clinical care.

### Methods

This scoping review was conducted with an information specialist in MEDLINE, Embase, and APA PsychINFO, adhering to the PRISMA-ScR checklist. Included studies were performed in the clinical care context, used PROMs for individual (pediatric or adult) patient care where PROM results are discussed with the patient, and described patient education on PROMs. Excluded articles were protocols, reviews, conference proceedings, non-original data, or non-English studies. Articles were double-screened by four co-author pairs, and data extraction was done by one pair and validated by a third co-author.

### Results

Titles and abstracts of 4,392 articles were screened, and 292 full texts were reviewed with approximately 40% excluded for solely lacking reports on patient education. Finally, 76 articles were included, mostly on adult patients. Preliminary results show that education was provided before PROM completion and sometimes during when patients encountered difficulties. Education was often provided by clinicians or research staff, using verbal, written or multi-media methods. Five components of patient education on PROMs were identified in the literature (Figure 1). Most education focused on explaining the purpose of PROMs and how to complete them.

### Conclusion

While patients may be educated about PROMs in clinical care, it is premature to consider patient education as standard in PROM implementation, due to the lack of detailed reporting in the literature. A survey with healthcare stakeholders can be the logical next step to better understand current practices. The effect of patient education on PROM completion is still largely unknown, and it is recommended that future PROM implementation/feasibility studies measure the impact of education and report it in literature.

**Fig. 1 (abstract 107.3):**
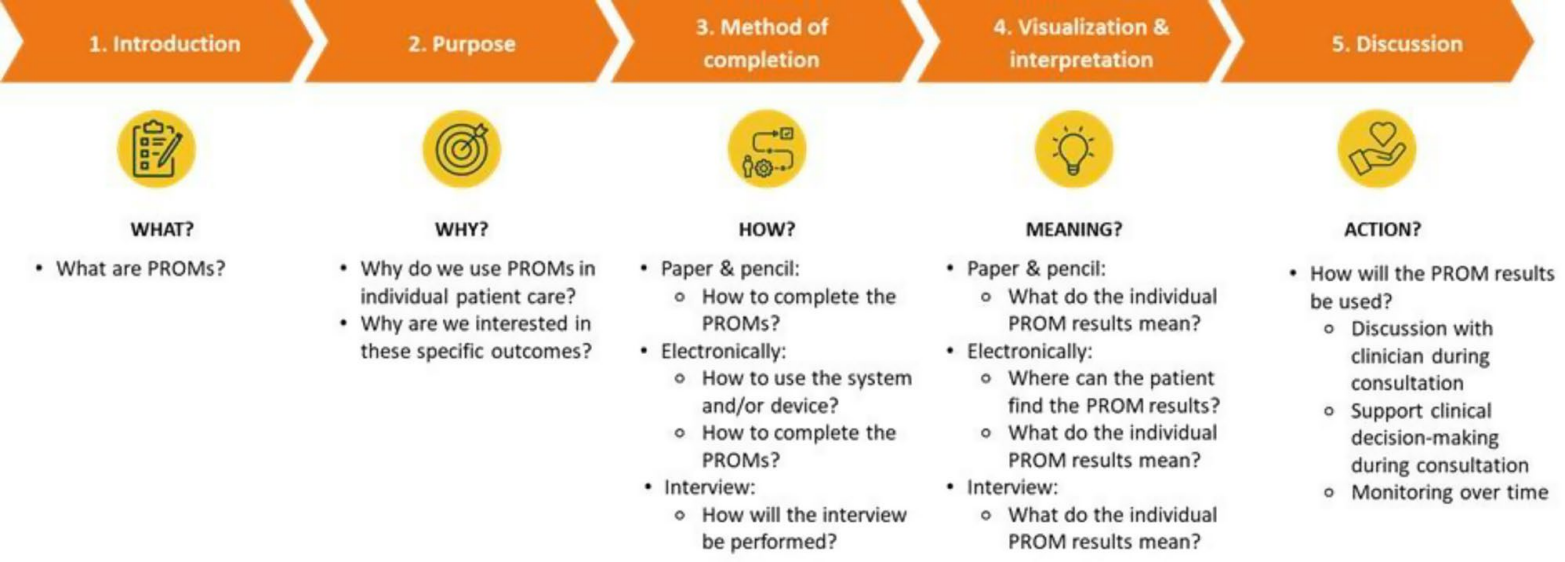
Patient education on patient-reported outcome measure completion in clinical care settings: a scoping review

## 107.2 Integrating PROMs into chronic disease management: A qualitative examination of patient and clinician views on PROM acceptability and timing

Jessica Nikolovski^1^, Marika Franklin^1^, Rachael L. Morton^1^, Brad Rossiter^2^, Margaret Fagan^2^, Matilda Armstrong^3^, Gill Hartas^3^, Claudia Rutherford^1^

^1^University of Sydney, Sydney, Australia, ^2^Consumer Representative, Sydney, Australia, ^3^Agency for Clinical Innovation (ACI), Sydney, Australia

*Journal of Patient-Reported Outcomes 2026*, **10(Suppl 1)**:107.2

### Aims

Patient-reported outcome measures (PROMs) are standardised tools that capture patients’ self-reported symptoms, functioning, and well-being. Little is known about the acceptability and timing of PROMs in the routine management of chronic conditions. This study aims to examine patient and clinician views on the acceptability and optimal timing of PROM completion in routine care.

### Methods

Semi-structured interviews were conducted with 33 patients and 25 clinicians across public healthcare settings in New South Wales, Australia. Six chronic conditions were selected based on variation in symptom trajectory and illness experience, and the type and timing of care clinicians provide. Interviews explored the acceptability and timing of PROMs in routine care for chronic condition management (osteoporosis, osteoarthritis, heart failure, chronic obstructive pulmonary disease, diabetes, kidney failure). The Theoretical Framework of Acceptability and social theory of time guided the deductive and inductive analyses.

### Results

Views on PROM acceptability and optimal timing were mixed within and across patient and clinician cohorts. Acceptability and timing emerged highly context-dependent and related to preferences for mode of PROM completion, content coverage, timing relative to disease trajectory and clinical encounters and intended use (e.g., goal setting in individual care, quality assurance activities for organisations and systems). Overall, clinicians’ views on acceptability and timing were influenced by workflow, available resources and perceived roles and responsibilities in what symptoms were actionable within their expertise. For patients, views on acceptability and timing were largely influenced by their daily experiences living with chronic condition(s) and how they anticipated their condition would progress over time.

### Conclusion

Standardised assessment time points are useful for health service use, but flexibility in the timing of PROM completion is needed to allow for personalised care that aligns with patient and clinician preferences, clinical contexts, disease trajectories and intended uses. This, in turn, may increase patient and clinician engagement in PROMs and increase the clinical utility of PROMs during clinical encounters. However, this may have implications for the ways in which PROMs are used more broadly.

## 107.5 Developing PROM-GRIP: a reporting guideline for the integration or use of PROMs in clinical practice

Ellen Elsman^1^, Maud Van Muilekom^1^, Anouk Groenewegen^2^, Harpreet Chhina^3^, Amy M. Cizik^4^, Elizabeth Gibbons^5^, Joanne Greenhalgh^6^, Asha Hareendran^7^, Sarah Hughes^8^, Melissa Rowthorn^9^, Rasa Ruseckaite^10^, Claudia Rutherford^11^, Sam Salek^12^, Maria J. Santana^13^, Angela Stover^14^, Galina Velikova^15, 16^, Philip van der Wees^17^, Meagan Whisenant^18^, Angela Wolff^19^, Claire Snyder^20^, Anna Beurskens^21^

^1^Department of Child and Adolescent Psychiatry and Psychosocial Care, Amsterdam UMC, Emma Children’s Hospital, Amsterdam, Netherlands, ^2^Department of Child and Adolescent Psychiatry and Psychosocial Care, Amsterdam UMC, University of Amsterdam, Emma Children’s Hospital, Amsterdam, The Netherlands, ^3^University of British Columbia, Vancouver, British Columbia, Canada, ^4^Department of Orthopaedics, Spencer Fox Eccles School of Medicine, University of Utah, Salt Lake City, Utah, USA, ^5^Evidera, Thermofisher, London, UK, ^6^University of Leeds, Leeds, UK, ^7^University of Bedfordshire, Sevenoaks, UK, ^8^Centre for Patient Reported Outcome Research, University of Birmingham, Birmingham, UK, ^9^Independent, Auckland, New Zealand, ^10^Monash University, Melbourne, Australia, ^11^University of Sydney, Faculty of Medicine and Health, Sydney Quality of Life Office, Susan Wakil School of Nursing and Midwifery, Sydney, Australia, ^12^School of Life and Medical Sciences, University of Hertfordshire, Hatfield, UK, ^13^Cumming School of Medicine, University of Calgary, Calgary, Alberta, Canada, ^14^University of North Carolina at Chapel Hill, Chapel Hill, USA, ^15^Leeds Institute of Medical Research, University of Leeds, Leeds, UK, ^16^Leeds Cancer Centre, St James’s University Hospital, Leeds, UK, ^17^Radboud University Medical Center, Science Department IQ Health & Department of Rehabilitation, Nijmegen, Netherlands, ^18^UTHealth Houston Cizik School of Nursing, Houston, USA, ^19^Trinity Western University, Langley, British Columbia, Canada, ^20^Johns Hopkins School of Medicine, Baltimore, USA, ^21^Maastricht University, Department of Family Medicine, Maastricht, Netherlands

*Journal of Patient-Reported Outcomes 2026*, **10(Suppl 1)**:107.5

### Aims

Patient-reported outcome measures (PROMs) are increasingly integrated or used in clinical practice for screening, monitoring, and discussing health outcomes, and their effectiveness has often been shown. However, it is unclear which underlying mechanisms play a role because of variability in reporting and lack of key information. To facilitate sufficient, transparent, and consistent reporting, we aimed to develop a new reporting guideline for studies on the integration or use of PROMs in clinical practice: PROM-GRIP (Patient-Reported Outcome Measures – Guideline for Reporting In clinical Practice).

### Methods

PROM-GRIP was developed following the EQUATOR (Enhancing the QUAlity and Transparency Of health Research) guidelines. Potential reporting items were identified by searching the literature and consulting PROM implementation experts. Then, a two-round international Delphi study and consensus meeting were held with panelists having experience with PROMs in clinical practice. Consensus meeting participants collaboratively drafted texts for the Explanation & Elaboration (E&E) document, consisting of a rationale and examples for each item. During the planned pilot testing, authors will test the guideline while writing manuscripts about the integration or use of PROMs in clinical practice. Results will be presented at ISOQOL 2025.

### Results

We identified 27 potentially relevant reporting items from the literature and expert consultations. In total, 161 panelists responded to Delphi round 1 and 119 panelists to round 2; 99 panelists completed both rounds. Panelists represented 21 countries and had an average of 12 years (range 0-25) work experience with PROMs in clinical practice. Twenty participants from 6 countries attended a full-day consensus meeting at the ISOQOL 2024 pre-conference day. This resulted in consensus on 22 reporting items addressing the abstract, introduction, methods, results, and discussion sections. We also reached consensus on the scope of PROM-GRIP, and created a conceptual framework covering the key aspects of integrating or using PROMs in clinical practice: purpose & planning, selection, administration, feedback, training, evaluation, and future perspectives.

### Conclusion

The newly developed PROM-GRIP reporting guideline provides structured guidance for transparent and comprehensive reporting, facilitating a better future understanding of how and when the integration or use of PROMs in clinical practice is effective.

## 108.4 Perceived Burden of Completing Patient-Reported Outcome Measures: Qualitative Interviews with Oncology Clinical Trial Participants

Ashley Geiger^1^, Kelly McCarrier^2^, Emily Evans^3^, Nancy Touba^4^, Josh Biber^1^, Dasha Cherepanov^1^

^1^Takeda Development Center Americas, Inc., Lexington, Massachusetts, USA, ^2^OPEN Health, Seattle, Washington, USA, ^3^OPEN Health, Durham, North Carolina, USA, ^4^OPEN Health, London, UK

*Journal of Patient-Reported Outcomes 2026*, **10(Suppl 1)**:108.4

### Aims

To enhance methods for collecting patient-reported outcome measure (PROM) data by exploring experiences of patients who had completed PROMs in oncology clinical trials. We sought to elicit (1) patients’ perspectives on the time and effort required to complete PROMs, (2) the perceived benefits and risks of completing PROMs, (3) burden of PROM completion in clinical trials, and (4) suggestions to reduce burden and increase perceived value of PROM completion.

### Methods

Semi-structured qualitative interviews were conducted with US-based adults who had completed PROMs in oncology clinical trials within the past 24 months. Participants were recruited through Rare Patient Voice using purposive sampling. Semi-structured interviews were conducted via web conference. Interview sessions were transcribed verbatim, deidentified, and managed within Atlas.ti for transcript coding and content analysis. Patients self-reported demographic and clinical information.

### Results

Participants (N=25) were 28–78 years old (median, 52 years); 76% were female; 76% were White; 8% reported Hispanic ethnicity. Most patients had breast cancer (40%), leukemia/lymphoma (20%), or lung cancer (16%). Most (56%) had completed their clinical trial participation prior to the interview, of which 71% had exited their trial within the previous 6 months. Evidence of concept saturation was observed in descriptions of burden and suggestions for improvement with PROM data collection. Participants reported completing an average of 28 PROM items with an average completion time of 19.7 minutes. Participants generally reported low levels of perceived burden, with one participant noting that completing the requested measures interfered with daily activities. Participants rated the importance of collecting PROM data highly (median=9, range=5-10 on scale of 0 [not at all important] to 10 [extremely important]). Participant suggestions to improve experiences with PROMs included: (1) reducing overall burden, (2) improving PROM content relevance, (3) optimizing format, setting, instructions, and reminders, and (4) addressing participant concern about potential risks of PRO measurement (Figure).

### Conclusion

Interviews provided valuable insights into patient experiences and suggestions to improve PROM data collection to reduce patient burden and potentially increase PROM completion rates. Future studies are warranted to understand how patient burden of PROM completion may be experienced differently depending on tumor type, disease severity, and treatment regimen.

**Fig. 1 (abstract 108.4):**
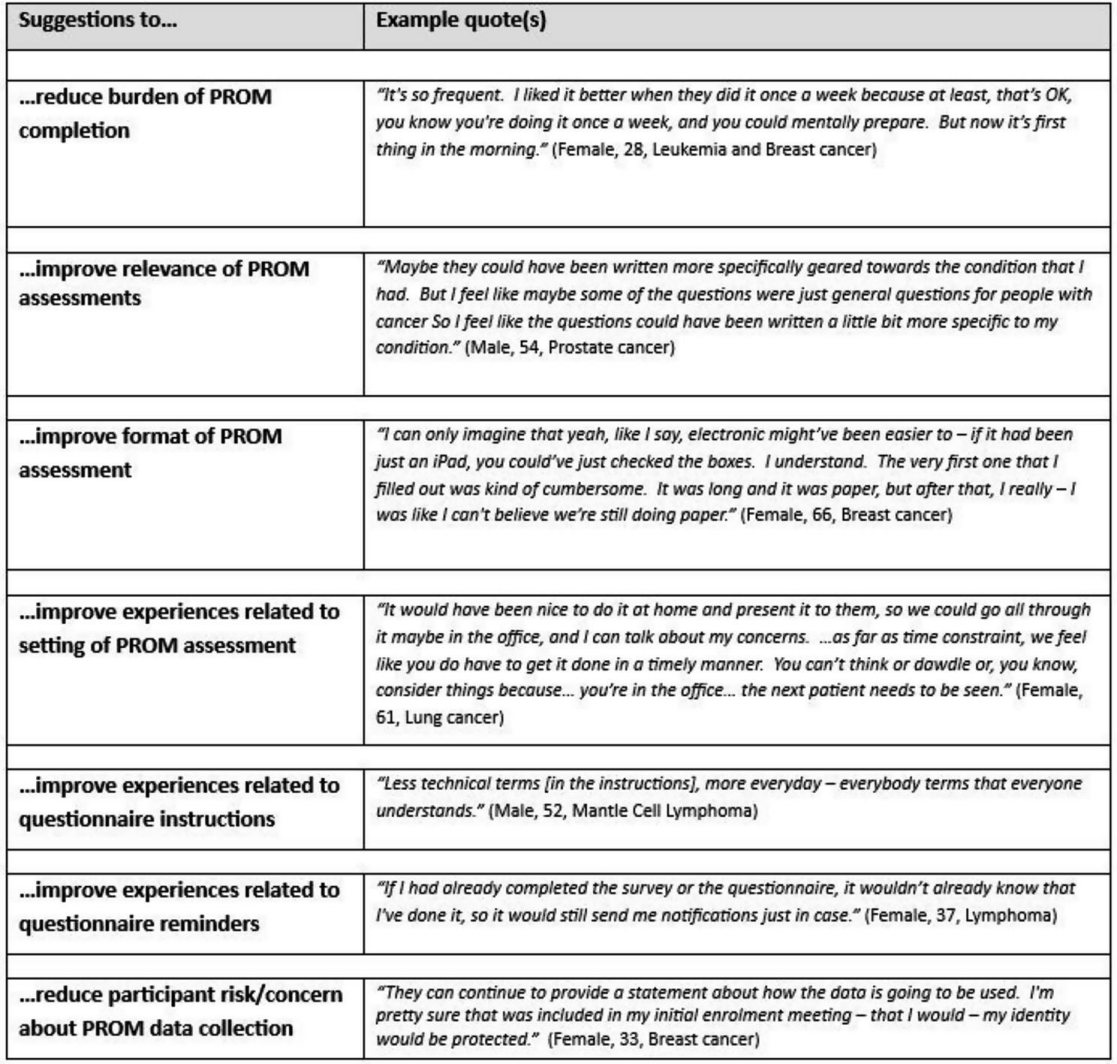
Example patient-expressed suggestions to improve experiences of PROM data collection

## 108.3 Stakeholder perspectives on possible open-label bias of patient-reported outcome results from cancer randomized controlled trials: the IMPROvE Project

Fabio Efficace^1^, Neil Aaronson^2^, David Cella^3^, Gary S. Collins^4^, Francesco Sparano^1^, Massimo Di Maio^5^, Johannes M. Giesinger^6^, Rajshekhar Chakraborty^7^, Amelie Anota^8^

^1^Italian Group for Adult Hematologic Diseases (GIMEMA), Data Center and Health Outcomes Research Unit, Rome, Italy, ^2^Division of Psychosocial Research and Epidemiology, Netherlands Cancer Institute, Amsterdam, The Netherlands, ^3^Department of Medical Social Sciences, Northwestern University Feinberg School of Medicine, Chicago, Illinois, USA, ^4^Center for Statistics in Medicine, NDORMS, University of Oxford, Oxford, UK, ^5^Department of Oncology, University of Torino, AOU Città della Salute e della Scienza, Torino, Italy, ^6^University Hospital of Psychiatry II, Medical University of Innsbruck, Innsbruck, Austria, ^7^Division of Hematology and Oncology, Department of Medicine, Columbia University Irving Medical Center, New York, USA, ^8^Biostatistics Unit, Direction of Clinical Research and Innovation, and French National Platform Quality of Life and Cancer, Centre Léon Bérard, Lyon, France

*Journal of Patient-Reported Outcomes 2026*, **10(Suppl 1)**:108.3

### Aims

Given that many randomized controlled trials (RCTs) now include patient-reported outcomes (PROs) and many of them are open-label studies, a better understanding of whether PRO results may be influenced by the lack of blinding becomes a critical question. We designed an international project, the IMportance of Patient-Reported Outcomes in Cancer Clinical Trials: Evaluating the Effects of Therapy Masking (IMPROvE) project, to investigate the relationship between study design (i.e., open-label vs. blinded trials) and PRO results, and determine the magnitude of open-label bias (if any) in RCTs investigating anti-cancer treatments of hematologic malignancies and solid tumors. We present the initial results of the first step of the project, reporting the stakeholders’ perspective on this matter.

### Methods

We conducted qualitative interviews with different stakeholders (academic and patient groups) to obtain insight into concerns related to the potential open-label bias of PRO endpoints and to identify factors possibly associated with such bias. The interview guideline was developed and discussed with the IMPROvE advisory board, and pilot-tested before starting the interviews. The first part of the interview consisted of open-ended questions, while in the second part interviewees rated the importance of predefined trial characteristics regarding their impact on the magnitude of open-label bias.

### Results

The nine stakeholders (i.e., academics with PRO expertise and oncologists) from Europe, USA and Australia we interviewed indicated that PRO responses in open-label trials could be influenced by patient-physician interaction, placebo as comparator, progression/recurrence status, interview as PRO mode of administration, type of PRO domain assessed (e.g. global QoL), and event-driven PRO assessment schedules. Eleven patients and patients’ representatives from Europe and USA were also interviewed and indicated advance disease and high expectations placed upon the experimental treatment as factors possibly influencing the open-label bias of PRO endpoints. Conversely, some suggested a good relationship/communication with the doctor could prevent such bias.

### Conclusion

These first results provide hypotheses regarding factors that might influence PRO assessments in open-label cancer RCTs. These factors are being considered in the second step of the project, which involves reviewing all cancer RCTs with a PRO endpoint to quantify the magnitude of this bias, if any.

## 108.1 Consensus-Based Recommendations for Designing, Analyzing, Interpreting, and Presenting Patient-Reported Outcomes in Cancer Clinical Trials

Kate Sandberg^1^, Ahu Alanya^2^, Cecilie Delphin Amdal^3^, Kristin Bjordal^3^, Elektra Papadopoulos^1^, Silene ten Seldam^4^, Anders Ingelgård^5^, Madeline Pe^2^

^1^AbbVie Inc., North Chicago, Illinois, USA, ^2^European Organisation for Research and Treatment of Cancer (EORTC), Brussels, Belgium, ^3^Research Support Services, Oslo, University Hospital and Medical faculty, University of Oslo, Oslo, Norway, ^4^Myeloma Patients Europe, Brussels, Belgium, ^5^Boehringer Ingelheim International GmbH, Ingelheim am Rhein, Germany

*Journal of Patient-Reported Outcomes 2026*, **10(Suppl 1)**:108.1

### Aims

Patient-reported outcomes (PROs) are important for evaluating the benefits and risks of cancer therapies. However, a lack of standards for PRO analysis and reporting has hindered optimal use of PRO data in decision making. Setting International Standards in Analysing Patient-Reported Outcomes and Quality of Life Endpoints (SISAQOL), a public-private collaborative research project under the Innovative Medicines Initiative (IMI), aims to address this gap. The consortium included multidisciplinary experts from a wide range of stakeholders and established consensus-based guidelines for designing, analyzing, interpreting and presenting PRO results in cancer clinical trials.

### Methods

The SISAQOL-IMI guidelines were developed over a 4-year period, with proposed recommendations being discussed and voted upon at yearly consensus meetings. Statements achieving a two-thirds majority were accepted. Preliminary recommendations were also independently tested with 12 external participants, who applied the recommendations to a study protocol, statistical analysis plan, and outline of PRO results presentation.

### Results

The final recommendations address four scientific areas: randomized controlled trials (RCTS), single-arm trials (SATs), visualization of PRO results, and PRO score interpretation thresholds. They will be presented in an interactive table organized by study objective (e.g., confirmatory, descriptive) and PRO variable of interest (e.g., change from baseline). Key topics addressed by the recommendations include the estimand framework, strategies for intercurrent events and missing data, and overall PRO analysis strategy. Guidelines for both scientific and plain language figure types are also provided. Finally, key criteria are provided for selecting an appropriate PRO score interpretation threshold. Other SISAQOL-IMI outputs include a guidebook outlining the SISAQOL-IMI consensus process and final recommendations, plain language checklists and tutorial videos, and an interactive glossary providing scientific and plain language definitions for over 200 terms.

### Conclusion

Establishing a unified set of standards for the analysis, interpretation and communication of PRO results is crucial to optimize the use of PRO data from cancer clinical trials in decision-making. Ultimately, by establishing consensus-based guidelines for PRO data, the SISAQOL-IMI recommendations can enhance the use and interpretation of PRO results to inform treatment decisions and improve individual patient well-being.

## 108.5 Meaningful tolerability endpoints to capture the dynamics of side-effect experience of cancer treatment

Konstantina Skaltsa^1^, Devin Peipert^2^, Antoine Regnault^3^, Jessica Roydhouse^4^

^1^IQVIA, Barcelona, Spain, ^2^University of Birmingham, Birmingham, UK, ^3^Modus Outcomes, Lyon, France, ^4^University of Tasmania, Hobart, Australia

*Journal of Patient-Reported Outcomes 2026*, **10(Suppl 1)**:108.5

### Aims

Tolerability has gained increasing attention in the past years for the evaluation of new treatments, especially in oncology. Regulatory agencies have recognized tolerability as a critical aspect in drug development for oncology compounds, as evidenced by the FDA’s guidance on Core Outcomes (2024). Two endpoint specifications, with corresponding estimands, have been proposed for the assessment of comparative tolerability: proportion of patients with high side-effect bother and time spent with high side-effect bother over a given exposure period. Supplementary insights on the timing of side-effects or side-effect bother may provide useful information when comparing different treatment options. In this talk, we introduce endpoints aiming to explore the timing of side effects to obtain a more comprehensive picture of tolerability.

### Methods

Meaningful questions to clinicians and patients were identified and endpoints operationalizing these questions based on the global items GP5 or 168 of the EORTC library, as well as PRO-CTCAE were defined. We systematically evaluated the analytical challenges associated with these endpoints, using the estimands framework. Fundamental statistical questions were examined, such as baseline adjustment, patient selection, missing data handling and multiplicity of tests.

### Results

Meaningful questions related to the timing and duration of side-effect occurrence include: when side effects or side-effect bother start, how long a bothersome side-effect episode lasts, and when side effects improve or resolve. Time-to-event endpoints will be presented: time to first worsening of a symptom, and time to recovery/symptom resolution. Definitions of worsening and recovery will be discussed, along with considerations around assessment schedules and missing data.

### Conclusion

Measuring and analyzing tolerability remains an active area of research in both early and late phase oncology trials. Extracting insights from tolerability measures can play a pivotal role in early phase trials and should be included with efficacy and safety evidence for an intervention in late phase trials. Critically, it can inform clinician-patient discussions and shared treatment decision-making. Additional questions aiming to reflect time dynamics that are relevant to patients initiating a treatment can be posed and answered via well-defined endpoints to ensure robust evidence is gathered.

## 108.2 How are digital COA endpoints defined? Some examples from current efforts in drug development

Gerasimos Dumi^1^, Aleksandra Sjöström-Bujacz^2^, Konstantina Skaltsa^3^

^1^IQVIA, Athens, Greece, ^2^IQVIA, Stockholm, Sweden, ^3^IQVIA, Barcelona, Spain

*Journal of Patient-Reported Outcomes 2026*, **10(Suppl 1)**:108.2

### Aims

Digital health technologies (DHTs) are increasingly used in clinical trials to measure either novel or existing physiological/behavioral outcomes, often remotely in patients’ daily lives and in a near-continuous manner. Transforming vast amount of such data to trial endpoints aimed at detecting treatment differences follows well-known principles, but also presents with novel challenges. In this talk we will discuss examples of digital endpoints currently used in clinical trials examining in detail how they were defined and planned to be analyzed. Building on the current practice, we aim to provide process guidance on the specific requirements of digital COA endpoints that would help sponsors and DHT developers avoid common problems associated with the use of DHT-based measures as endpoints in clinical trials.

### Methods

We reviewed selected clinical trial endpoints included in the Digital Medicine Society (DiMe) library and focused on the area of cough, sleep, and mobility in different indications. Trial identifier for the selected studies was used to search for further information on the endpoints and their planned analysis in PubMed, as well as grey literature in google scholar (including conference papers) and ClinicalTrials.gov (including statistical analysis plans and protocols).

### Results

In this review, we found different levels of aggregation being used for near-continuous data for each identified measure from the epoch level (e.g., minute) to the summary level (e.g., week or month), diverse summary statistics and measurement periods. The results also highlighted crucial considerations on required wear time and rules for handling the missing data. Finally, our review showed that digital COAs are typically aggregated and resemble traditional trial endpoints analyzed using standard statistical methods, such as Analysis of Covariance (ANCOVA) or Mixed Model for Repeated Measures (MMRM).

### Conclusion

In comparison to standard COA endpoints, building a digital COA endpoint requires several additional considerations and decisions to be made. While utilizing traditional methods for statistical analyses of DHT data may be convenient in terms of results interpretation and communication, near-continuous monitoring may be further exploited to provide more granular insights on patient experience and treatment impact.

## 109.4 A pilot study of emotion and non-cognitive measures of decision-making ability in older adults within ARMCADA

Elizabeth M. Dworak^1^, Sarah Pila^1^, Y. Catherine Han^1^, Miriam A. Novack^1^, Emily H. Ho^1^, Berivan Ece^1^, Patricia Bucko^1^, Tatiana Karpouzian-Rogers^2^, Molly A. Mather^2, 3^, Zahra Hosseinian^1^, Patricia Bucko^1^, David Cella^1^, Richard C. Gershon^1^, Sandra Weintraub^2, 3^

^1^Department of Medical Social Sciences, Northwestern University Feinberg School of Medicine, Chicago, Illinois, USA, ^2^Department of Psychiatry and Behavioral Sciences, Northwestern University Feinberg School of Medicine, Chicago, Illinois, USA, ^3^Mesulam Center for Cognitive Neurology and Alzheimer’s Disease, Northwestern University Feinberg School of Medicine, Chicago, USA

*Journal of Patient-Reported Outcomes 2026*, **10(Suppl 1)**:109.4

### Aims

The Advancing Reliable Measurement in Cognitive Aging and Decision-making Ability (ARMCADA) initiative aims to develop a multidomain decision-making (DM) battery for adults aged 45 and above that will be sensitive to changes in decision-making ability, potentially predicting cognitive decline. One of the domains our initiative assesses is emotion and non-cognitive decision-making. After conducting a scoping review of studies published between 2018 and 2023, we identified the most frequently used emotion/non-cognitive measures. The aim of the current pilot study is to assess the feasibility and reliability of these measures and to compare them with self-reported subjective cognitive decline.

### Methods

A pilot sample of 150 adults aged 45 and above will be recruited through an external online recruitment vendor. Participants will complete a demographic questionnaire, depression, anxiety, and social isolation screeners, the 12-item Everyday Cognition Scale (ECog-12), a Memory Complaint Scale, and the selected non-cognitive/emotion measures via REDCap online in a single session. Emotion/non-cognitive measures will include self-reported impulsivity, gullibility, and personality, along with the Mild Behavioral Impairment Checklist and the Reading the Mind in the Eyes Test. The measures are self-administered and take approximately 20 minutes. Feasibility will be evaluated by the percentage of participants completing all items, and reliability will be examined using Cronbach’s alpha coefficient.

### Results

We expect the emotion and non-cognitive measures to be feasible and reliable for middle-aged and older adults, with high completion rates (>95%) and strong internal consistency (Cronbach’s alpha > .80) for each measure. We also anticipate that better emotional and non-cognitive functioning will be associated with lower subjective cognitive decline. These findings will help identify the most relevant items, allowing for a more focused and efficient battery in future studies.

### Conclusion

The results of this study are anticipated to support the feasibility and internal consistency of the selected emotion/non-cognitive measures. The results will help guide which measures and individual items should be included in the final ARMCADA battery based on their practicality (e.g., readability) and psychometric soundness. Findings of the pilot study will also lay the foundation for future studies that will investigate possible domain interactions (e.g., financial and emotion/non-cognitive decision-making).

## 109.3 Comparative performance of the EQ-5D-5L and PROMIS-10 in the elderly: Data from the EQ-DAPHNIE project in five countries

Thao Nguyen^1^, Arto Ohinmaa^1^, Hilary Short^1^, Fatima Al Sayah^1^

^1^Alberta PROMs and EQ-5D Research and Support Unit (APERSU) School of Public Health, University of Alberta, Edmonton, Alberta, Canada

*Journal of Patient-Reported Outcomes 2026*, **10(Suppl 1)**:109.3

### Aims

This study compared the performance of the EQ-5D-5L and PROMIS-10 in measuring health-related quality of life (HRQL) among older adults in five English-speaking countries.

### Methods

Using data from the EQ-DAPHNIE project, 5403 respondents aged 65 years or older were included from Canada (n=1312), United States (US) (n=1069), United Kingdom (UK) (n=970), Australia (n=1224), and New Zealand (n=828), with analyses conducted overall and by age groups (65-69, 70-74, 75-79, ≥80) for each country. Spearman correlations assessed convergent and divergent validity, and known-groups analysis examined discriminative validity of EQ-5D-5L dimensions and total sum score (TSS), and PROMIS-10 Global Physical Health (GPH) and mental health (GMH) T-scores.

### Results

The average age of respondents was 72.6 years (72.1-73.6) and 48.2% were female (44.3%-53.3%). The lowest EQ-5D-5L TSS (mean 8.1; SD 2.9) and PROMIS-10 GPH (47.0; 4.5) and GMH (49.3; 4.9) T-scores were reported in Canada. Conversely, the highest TSS and PROMIS-10 GPH T-scores were in the UK (7.5; 3.0 and 48.5; 4.6, respectively), while New Zealand had the highest PROMIS-10 GMH T-score (51.5; 4.0).The EQ-5D-5L mobility and pain/discomfort dimensions had strong correlations (0.45-0.82) with PROMIS-10 physical health items and weak correlations (0.15-0.39) with PROMIS-10 mental health items. The EQ-5D-5L anxiety/depression dimension had strong correlations (0.55-0.70) with PROMIS-10 mental health items and weak correlations (0.23-0.45) with PROMIS-10 physical health items. These findings demonstrate convergent and divergent validity for physical and mental health dimensions across all five countries. Discriminative ability of the EQ-5D-5L TSS and PROMIS-10 GPH T-score was strongest amongst those with depression, kidney disease, obesity, and respiratory disease (effect sizes 0.49-1.32 and 0.45-1.06, respectively), and weaker in heart disease, diabetes, hypertension, and skin disease (0.23-0.75 and 0.09-0.90). The PROMIS-10 GMH T-score discriminated only between individuals with and without depression. The performance of both measures slightly varied across the five countries, with effect sizes systematically larger in the US in most diseases.

### Conclusion

Both EQ-5D-5L and PROMIS-10 effectively capture HRQL in older adults across five English-speaking countries. Despite their acceptable discriminative ability in some conditions, performance was relatively poor in common age-related diseases including diabetes, heart disease, hypertension, and skin disease.

## 109.2 Comparing age-specific and generic well-being scales: A systematic review and content analysis for older adults

Marie-Louise Möllerberg^1^, Marit Preuter^2^, Kristofer Årestedt^3^, Jeanette Melin^4^

^1^Malmö university, Malmö, Sweden, ^2^RISE, Research Institutes of Sweden, Division Built Environment, Department System Transition and Service Innovation, Local and Regional Transition, Gothenburg, Sweden, ^3^Linneaus University, Department of Health and Caring Sciences, Kalmar, Sweden, ^4^Swedish Defence University, Department of Leadership, Demand and Control, Karlstad, Sweden, Gothenburg, Sweden

*Journal of Patient-Reported Outcomes 2026*, **10(Suppl 1)**:109.2

### Aims

It is often assumed that scales designed to measure the same phenomenon can be used interchangeably and provide comparable information about respondents. However, a growing body of research on content overlap between scales reveals considerable heterogeneity among scales intended to capture the same phenomenon. Yet, little is known about the topic when measuring well-being in older adults. Therefore, the aim was to systematically review and analyze the content of existing scales used to measure older adults’ well-being.

### Methods

A comprehensive search across PubMed, PsycInfo, and CINAHL identified psychometric studies of scales assessing well-being or quality of life in older adults. Articles were screened, duplicates removed and selected for full-text review. To evaluate the content of the existing scales, categorization was made into 21 dimensions proposed by Iasiello et al. 2024. Content overlaps were analyzed using the Jaccard index, providing insights into the scales’ similarity and diversity.

### Results

The study categorized 830 items from 36 scales based on 21 dimensions (38 items categorized as not applicable). Scales developed explicitly for older adults prominently featured dimensions like Personal relationships, Personal circumstances, and Physical health, while generic scales highlighted Activities and functioning, Personal relationships, and Self-acceptance. The Jaccard index was used to analyze content overlaps, revealing varying degrees of similarity among the scales. Five scales for older adults contained only positively phrased items. While the overall distribution of dimensions was consistent between positively and negatively phrased items, notable exceptions included Self-Congruence, which was more common in positively phrased items, and Calmness, which was more common in negatively phrased items.

### Conclusion

The study confirms findings from other contexts; scales designed to measure the same phenomenon cannot provide comparable information about respondents. Through a systematic review and analysis of existing scales, key dimensions frequently represented have been identified. Yet there is a diversity in content, which threatens the validity of both the phenomenon being measured and the scales used. Differences in content between age-specific and generic scales support the validity of age-specific measures in validly capturing well-being in older adults.

## 109.1 A gender disaggregated growth curve analysis of mobility decline and its sociodemographic correlates among community-dwelling older Nigerians

Ogochukwu Kelechi Onyeso^1^, Chiedozie James Alumona^1^, Akin Ojagbemi^2^, Kelechi Mirabel Onyeso^3^, Adesola C. Odole^4^, Janice Victor^1^, Jon Doan^1^, Toyin Bello^2^, Oye Gureje^2^, Oluwagbohunmi A. Awosoga^1^

^1^Faculty of Health Sciences, University of Lethbridge, Lethbridge, Alberta, Canada, ^2^World Health Organization Collaborating Centre for Research and Training in Mental Health, Neuroscience, and Substance Abuse, Department of Psychiatry, College of Medicine, University of Ibadan, Ibadan, Nigeria, ^3^Department of Estate Management, Faculty of Environmental Sciences, University of Nigeria, Nsukka, Enugu, Nigeria, ^4^Department of Physiotherapy, Faculty of Clinical Sciences, College of Medicine, University of Ibadan, Ibadan, Oyo, Nigeria

*Journal of Patient-Reported Outcomes 2026*, **10(Suppl 1)**:109.1

### Aims

Gait speed is an important predictor of older adults’ physical and cognitive functioning, general health status, and quality of life. We estimated the influence of sociodemographic factors on the gait speed decline of community-dwelling older Nigerians.

### Methods

This study was a secondary analysis of the Ibadan Study of Ageing. A representative sample of Nigerian older adults aged 68 years and above were assessed for gait speed in 2007, 2008, and 2009. We completed a gender disaggregate analysis of participants’ sociodemographic and cycle-wise gait speed differences, and their effects on gait speed trajectory using Pearson’s chi-square, mixed-design ANOVA, and growth curve analysis (GCA).

### Results

At baseline, 53.2% of participants were female, 61.9% were married, with an average age of 75.5 ± 6.8 years and gait speed of 0.96 ± 0.32 m/s. The GCA revealed a significant decline in gait speed with age for all participants (β = -0.01, p < 0.001). Gender-specific models showed slower gait speed decline in men (β = -0.05, p < 0.001) compared to women (β = -0.09, p < 0.001). For women, widowhood (β = -0.07, p = 0.001) and chronic disease burden (β = -0.02, p = 0.010) were significant predictors of gait speed decline, while high socioeconomic status (β = -0.01, p = 0.009) and chronic disease burden (β = -0.03, p = 0.008) were significant for men. No significant difference was observed between linear and quadratic growth curve models (ꭓ2[1] = 2.409, p = 0.121).

### Conclusion

Addressing culture-related widowhood and women’s vulnerabilities, improving health coverage, and promoting lifestyle modifications may mitigate mobility decline and improve the quality of life among older Nigerians.

## 109.5 Fear of falling as a personality-mediated experience: older adults’ adaptation and identity in a shifting embodied world

Henrietha Adandom^1^, Michael Kalu^2^, Israel Adandom^3^, Adesola Odole^4^, Lisa Cook^1^, Gongbin Shan^1^, Oluwagbohunmi Awosoga^1^

^1^University of Lethbridge, Lethbridge, Alberta, Canada, ^2^York University, Toronto, Ontario, Canada, ^3^University of Alabama, Tuscaloosa, Alabama, USA, ^4^University of Ibadan, Oyo, Nigeria

*Journal of Patient-Reported Outcomes 2026*, **10(Suppl 1)**:109.5

### Aims

Fear of falling (FOF) significantly impacts older adults’ mobility, autonomy, and psychological well-being. While previous studies have explored its clinical and behavioral correlates, less attention has been paid to how older adults experience and make sense of FOF within sociocultural, environmental, and dispositional contexts. This study explored how personality traits shape older adults’ adaptation to FOF in Nigeria, offering foundational insights for future AI-informed quality of life interventions that honor the complexity of lived experience.

### Methods

This qualitative study used Interpretative Phenomenological Analysis (IPA) to explore how personality traits shape fear of falling (FOF) experiences among 17 older Nigerian adults. Participants (aged 60–75) completed a brief personality inventory and participated in semi-structured interviews. Data were analyzed idiographically to capture individual meaning-making processes. Analytic rigor was ensured through reflexive journaling, peer debriefing, and detailed audit trails.

### Results

Three superordinate themes emerged: (1) Living with Uncertainty – FOF was not constant but situational, shaped by changing bodily trust, spatial memory, and vulnerability in familiar and unfamiliar settings; (2) Coping as Personality-in-Action – participants high in conscientiousness structured routines and rituals to regain control; emotional stability supported reframing, and openness enabled adaptive engagement with aids and change, while neuroticism often intensified perceived vulnerability; (3) Relational Landscapes – coping was mediated by the availability of peer support, family presence, and institutional systems. When support was fragmented or absent, coping became emotionally taxing, contributing to withdrawal, increased caution, or dependence. Cultural scripts around aging, identity, and stigma also shaped help-seeking behaviors and adaptation, revealing how social expectations intersect with personal dispositions in shaping responses to FOF.

### Conclusion

Fear of falling is not solely a physical or psychological issue—it is a dynamic, meaning-laden experience filtered through personality traits and social environments. As AI tools increasingly inform health behavior interventions, fall prevention technologies, and predictive modeling, this study highlights the importance of grounding these tools in lived experience. Personality-informed, culturally grounded models can enhance personalization in AI-driven quality of life research and help avoid reductive risk profiling. Integrating qualitative insights into AI design will be key to ensuring older adults’ voices and identities are meaningfully represented in the future of health innovation.

**Fig. 1 (abstract 109.5):**
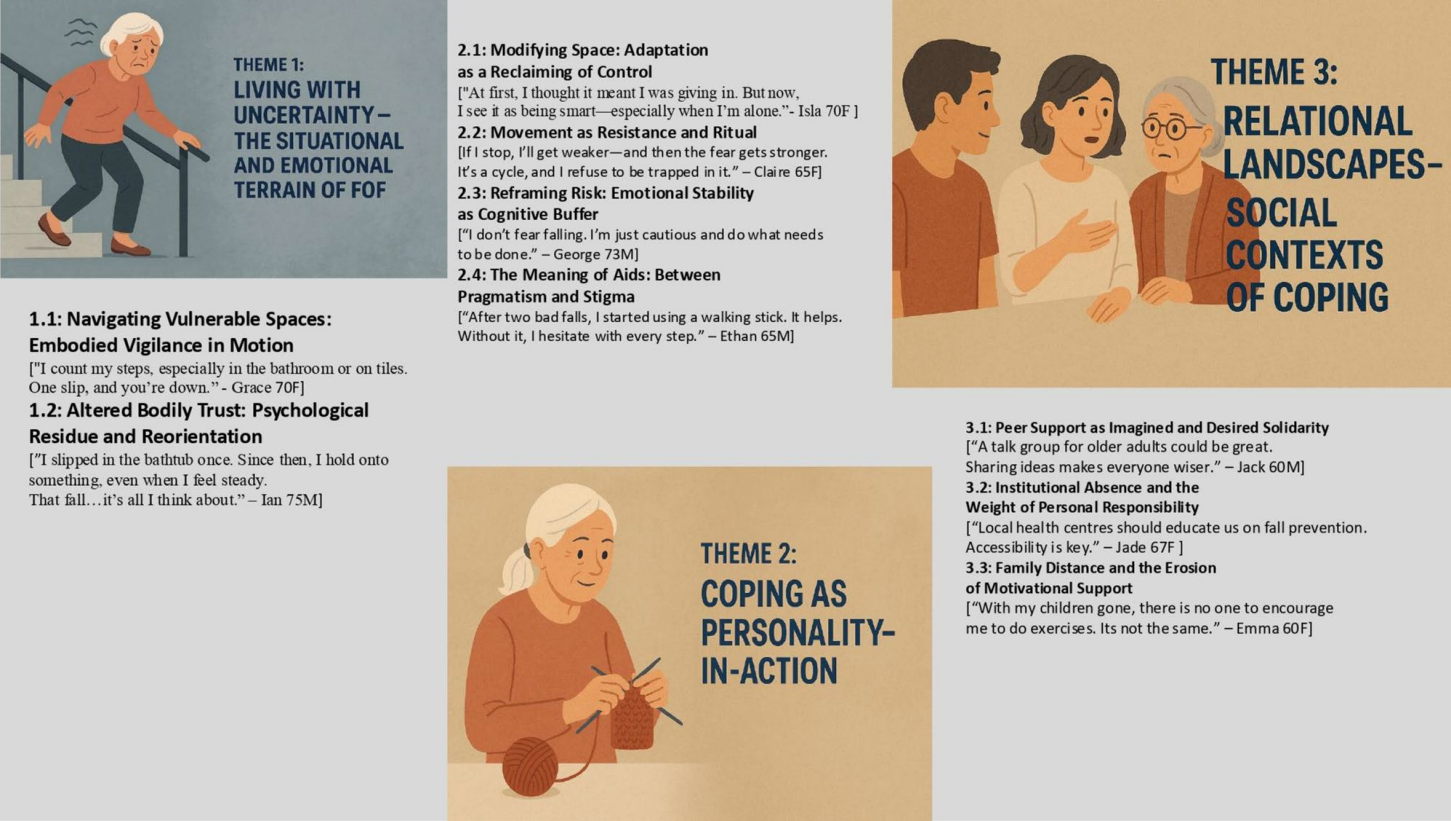
Fear of falling as a personality-mediated experience: older adults’ adaptation and identity in a shifting embodied world

## 110.2 Leveraging artificial intelligence (AI) for implementation of patient-reported outcome measures (PROMs) systems in mental health care

Amber Bailey^1^, Emily Berich-Anastasio^1^, Robert J. Schloesser^1,2^

^1^Sheppard Pratt, Baltimore, Maryland, USA, ^2^University of Maryland Department of Psychiatry, Baltimore, Maryland, USA

*Journal of Patient-Reported Outcomes 2026*, **10(Suppl 1)**:110.2

### Aims

This study explores the utilization of generative artificial intelligence (AI) tools to facilitate the implementation of patient-reported outcome measurement (PROM) systems in mental health care. Specifically, it aims to enhance the utility of diagnostic and symptom monitoring tools, optimize PROM data reporting, and assist with the collection and analysis of qualitative feedback about PROM systems. With its implementation, generative AI demonstrates the potential to reduce gaps in implementing evidence-based practices, including PROM, into real-world healthcare settings.

### Methods

For enhancing clinical utility of diagnostic and symptom monitoring tools, licensed clinicians reviewed available PROMs for ADHD to identify gaps in current measurement tools. Based on this review, they developed structured prompts about ADHD symptoms and current measurement tools, which were provided to a generative AI tool in order to optimize these tools for clinical utility. For data visualizations and reporting, the PROM implementation team developed simplistic descriptions of PROM data and provided these prompts to a generative AI tool to assist with identifying the best methods for reporting the data for providers and patients. To assist with reviewing qualitative data, the PROM implementation team utilized generative AI tools to transcribe and summarize meetings and support tickets.

### Results

Implementation of generative AI is ongoing. At this time, one PROM has been developed with generative AI assistance: ADHD screener (445 completions across 118 care episodes). PROM data presentation has expanded to determine patient-specific baselines based on time-weighted moving averages. Efficiency of qualitative data collection has increased with the ability to quickly summarize meeting notes and identify major tasks, which ultimately improves PROM system modification development.

### Conclusion

This study proposes the structured implementation of generative AI to improve measurement accuracy, data interpretation, and feedback analysis. By leveraging AI, this study aims to facilitate informed clinical decision-making and minimize the burden of implementing quality improvement practices.

## 110.3 Assessing ADHD in Psychiatric Care: Standardized Assessment and a Novel ADHD Assessment, Developed by Clinicians with Generative AI

Emily Berich-Anastasio^1^, Amber Bailey^1^, Robert Schloesser^1^

^1^Sheppard Pratt, Baltimore, Maryland, USA

*Journal of Patient-Reported Outcomes 2026*, **10(Suppl 1)**:110.3

### Aims

To describe development and early clinical use of the Point‑of‑Care Research & Innovation ADHD Assessment (POCRI‑ADHD) and to compare its performance with the Adult ADHD Self‑Report Scale (ASRS) within an electronic patient‑reported outcome (ePRO) platform. POCRI-ADHD is novel, briefer, addresses different core symptoms than the ASRS, and explores overmedication.

### Methods

Using generative AI tools, clinical experts created the POCRI-ADHD. This measure, and the ASRS, were incorporated into PoCDART (Point-of-Care Dashboards, Analysis, and Reporting Toolkit), an in-house ePRO system at a large psychiatric hospital in Maryland, USA. Administration frequency, score distributions, and diagnostic discrimination were examined; Welch two‑sample t‑tests compared scores between patients with and without ADHD diagnosis.

### Results

132 patients have completed 742 ADHD ePROs (331 ASRS and 411 POCRI-ADHD). 112 patients completed the POCRI-ADHD and the mean administration rate was 3.67 (range 1–31, SD 4.52); among 73 ASRS completers, the mean was 4.53 (range 1–31, SD 5.31). Mean initial POCRI-ADHD score was 8.9 (range 0–16, SD 0.41), while mean initial ASRS score was 3.9 (range 0–6, SD 0.50). Average per-patient means for multiple assessments were 8.86 for POCRI-ADHD (range 0.5–15.7, SD 3.36) and 0.75 for ASRS (range 1.5–6.0, SD 1.25). Notably, 63% (71/112) of POCRI-ADHD completers indicated potential overmedication.53 patients completed both measures, while 20 completed ASRS only and 59 completed POCRI-ADHD only. For patients who completed both assessments, those with ADHD had significantly higher ASRS scores (mean = 4.01) than those without (mean = 3.03), t(44.219) = −3.40, p < .01, 95% CI [−1.56, −0.40]. POCRI-ADHD scores also significantly differed between groups with (mean = 9.43) and without ADHD (mean = 6.97), t(50.74) = −4.26, p < 0.001, 95% CI [−3.62, −1.31].

### Conclusion

These findings examine two ADHD ePROs deployed in real-world clinical practice. Integrating ADHD-specific measures into routine care supports data-driven treatment. Promoting a measurement-based approach through development and application of disorder-specific ePROs can help advance data-driven approaches in psychiatry.

## 110.4 Valbenazine improves the impacts and symptoms of tardive dyskinesia: topline results from the phase 4 KINECT-PRO study

Eduardo Dunayevich^1^, M. Mercedes Perez-Rodriguez^2^, Joseph McEvoy^3^, Ashok Parameswaran^1^, Morgan Bron^1^, Ericha Franey^1^, Donna Sparta^1^, Cathy Zeng^1^, Susan D. Mathias^4^, Christoph U. Correll^5^

^1^Neurocrine Biosciences, Inc., San Diego, California, USA, ^2^Icahn School of Medicine at Mount Sinai, New York, New York, USA, ^3^Augusta University, Medical College of Georgia, Augusta, Georgia, USA, ^4^Health Outcomes Solutions, Palm Beach Gardens, USA, ^5^The Zucker Hillside Hospital, Glen Oaks, USA

*Journal of Patient-Reported Outcomes 2026*, **10(Suppl 1)**:110.4

### Aims

To evaluate the effects of once-daily valbenazine on the impacts of tardive dyskinesia (TD) using multiple reliable and valid patient-reported outcomes (PROs), including the Tardive Dyskinesia Impact Scale (TDIS), along with clinician-rated assessments for TD severity including the Abnormal Involuntary Movement Scale (AIMS).

### Methods

This open-label study included: screening (4 wks); treatment with valbenazine 40 mg (4 wks); continuation with 40 mg or increase to 60 or 80 mg (12 wks); stable dosing with 40, 60, or 80 mg (8 wks); and safety follow-up (2 wks). Participants had at least mild TD severity (per AIMS item 8) and were aware of their dyskinetic movements with at least mild associated distress (per AIMS item 10). PROs included the TDIS, Sheehan Disability Scale (SDS), and EuroQoL Group’s EQ Visual Analog Scale (EQ-VAS). Mean changes from baseline (CFB) for PROs and AIMS total score (sum of items 1-7) were analyzed by TD severity (mild or moderate/severe per AIMS item 8), psychiatric diagnosis (schizophrenia/schizoaffective disorder [SCHZ] or major depressive disorder/bipolar disorder [MOOD]), and overall.

### Results

Of 59 enrolled participants (24 mild, 35 moderate/severe; 27 SCHZ, 32 MOOD), 52 completed the Wk24 visit; 45 were included for efficacy analyses. In participants overall, TDIS, SDS, EQ-VAS, and AIMS improvements were observed by Wk4 (after initial treatment with valbenazine 40 mg) and sustained through Wk24. Mean CFB at Wk24 overall and by TD severity (mild, moderate/severe) were: TDIS (-8.0, -6.8, -8.9); SDS social life (-2.3, -1.8, -2.8) and family life (-1.6, -1.3, -1.8); EQ-VAS (+13.1, +12.8, +13.3). In the SCHZ and MOOD subgroups, mean CFB were: TDIS (-5.8, -9.7, respectively); SDS social life (-1.6, -2.9) and family life (-0.7, -2.3); EQ-VAS (+8.3, +17.0). CFB in AIMS total score were -6.8 (overall), -5.6 (mild), -7.8 (moderate/severe), -5.8 (SCHZ), and -7.6 (MOOD). Adverse events were consistent with the known safety and tolerability profile for valbenazine.

### Conclusion

Results of this study, the first to report the effects of a vesicular monoamine transporter 2 inhibitor on the impacts of TD using multiple reliable and valid PROs, demonstrate clinically meaningful and sustained improvements with valbenazine on TD severity and patient impact.

## 110.1 Prioritizing the Health and Well-Being of Frontline Healthcare Professionals in Canada

Oluwagbohunmi Awosoga^1^, Helen Kelley^2^, Claudia Steinke^3^, Suha Damag^4^

^1^216 Coalbanks Boulevard West, Lethbridge, Alberta, Canada, ^2^Dhillon School of Business, University of Lethbridge, Lethbridge, Alberta, Canada, ^3^Faculty of Health Sciences & Dhillon School of Business, University of Lethbridge, Lethbridge, Alberta, Canada, ^4^Faculty of Health Sciences, University of Lethbridge, Lethbridge, Alberta, Canada

*Journal of Patient-Reported Outcomes 2026*, **10(Suppl 1)**:110.1

### Aims

Frontline Healthcare Professionals (FHPs) are the backbone of healthcare systems, yet their health and well-being are often overlooked. Currently, half of the FHPs want to change jobs, with 94% showing signs of burnout and 83% concerned about being understaffed, impacting the quality of care/service. Nursing vacancies have surged by over 219% in the last five years, highlighting a significant crisis that leaders must address. These ongoing burdens are unsustainable and affect FHPs’ quality of life, work-life, and clinical care. There is an urgent need to prioritize the health and well-being of these professionals.

### Methods

Our main research questions were:i) What is the health status of FHPs in Alberta, Canada? ii) What strategies do FHPs use to maintain their health and well-being? iii) What supports do organizations provide for FHPs’ health and well-being? iv) How do these individual strategies and organizational supports affect FHPs’ quality of life, work life, and the care/services they provide? andv) What actions could enhance FHPs’ health and well-being?This mixed-methods study comprised two phases. Phase 1 involved semi-structured interviews with 20 FHPs from 11 hospitals in southern Alberta. Thematic analysis was conducted to identify key themes and actionable insights (see Table 1). Phase 2 consists of a quantitative online survey distributed to all FHPs working in acute care, emergency, and long-term care settings in hospitals in southern Alberta. Survey data collection is in progress.

### Results

Please see Table 1. Findings will inform FHPs about daily strategies for improving their health, well-being, and quality of life. Clinically, they will enhance understanding of how healthy strategies and organizational supports impact work life and clinical care. Organizationally, they may create an organizational culture and climate for the adoption of daily healthy strategies by FHPs, thus decreasing workloads and job demands, improving recruitment and retention of FHPs, and increasing the size of the FHP workforce.

### Conclusion

This study’s value is that it reinforces the immediate need to prioritize the health and well-being of FHPs. To ensure FHPs can provide high-quality care and service and maintain sustainability, we must improve how we support and care for them and serve them better.

**Table 1 (abstract 110.1):**
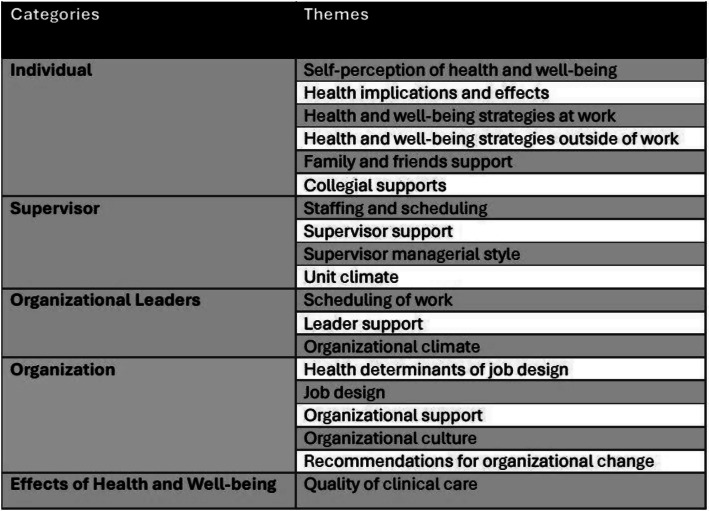
Prioritizing the Health and Well-Being of Frontline Healthcare Professionals in Canada

## 110.5 Association between concerns about AI in healthcare and self-reported anxiety among cancer survivors

Maria Rincon^1^, Roxanne Jensen^2^

^1^National Cancer Institute, Rockville, Maryland, USA, ^2^National Cancer Institute, Rockville, Virginia, USA

*Journal of Patient-Reported Outcomes 2026*, **10(Suppl 1)**:110.5

### Aims

Rapid expansion of Artificial Intelligence (AI) in healthcare offers exciting opportunities for care management, such as the employment of generative AI to enhance health information technology tools. Such is particularly relevant among patient populations, including cancer survivors, who have unique needs for managing their health post-treatment. The following analysis assesses the relationship between cancer survivors’ perceptions on the impact of AI in healthcare and self-reported anxiety.

### Methods

375 US-residing, English-speaking adults with a history of cancer completed a Qualtrics survey on eHealth literacy, e-health and health outcomes. Four items addressing concerns about AI in healthcare (impact on relationships with healthcare providers, improvement of health outcomes, concerns over medical errors and data security) were included as predictors in this analysis. The primary outcome for this study was PROMIS 29 anxiety t-scores, dichotomized at a score of 60. Binary logistic regression models were used to calculate odd ratios and p-values (p<0.05). Adjusted models included age, educational attainment and time since last cancer treatment as covariates.

### Results

The respondent sample for the survey was primarily White (80%), female (70%) and college-educated (63%). Unadjusted models determined that survivors reporting higher anxiety were more likely to report concern that AI will hurt their relationship with providers (OR=0.63, p=0.047) and that AI in medicine will increase medical errors (OR= 0.52, p=0.005). Statistical significance for the association between anxiety and concern about medical errors due to AI (OR=0.55, p=0.011) was retained in the adjusted model. Additional regression models assessing the association between the potential of AI to improve health outcomes, and concerns about data safety while using AI and anxiety were not significant.

### Conclusion

The increasing presence of AI in the healthcare setting poses interesting questions on what patients understand, and might react to, when facing emerging technologies. Individuals experiencing anxiety, who are often likely to exhibit avoidant behaviors when facing novel resources, are significantly more likely to be concerned with the impact of AI on undesirable outcomes to treatment and procedures. Future work should explore if these concerns impact care management behaviors among cancer patients and survivors.

## 201.1 Patient-reported outcomes (PROs) in the pragmatic comparative effectiveness trial of surgical versus nonsurgical management for patients with malignant bowel obstruction (S1316)

Amylou Dueck^1^, Kathryn Arnold^2^, Garnet Anderson^2^, Cynthia Thomson^3^, Virginia Sun^4^, Angeles Alvarez^5^, Gary Deutsch^6^, Jeremiah Deneve^7^, Robert Krouse^8^

^1^Mayo Clinic, Scottsdale, Arizona, USA, ^2^Fred Hutchinson Cancer Center, Seattle, Washington, USA, ^3^University of Arizona, Tucson, Arizona, USA, ^4^City of Hope National Medical Center, Duarte, California, USA, ^5^Secord Duke Cancer Institute, Durham, North Carolina, USA, ^6^Northwell Health Cancer Institute, Lake Success, USA, ^7^University of North Carolina, North Carolina, USA, ^8^University of Pennsylvania, Philadelphia, USA

*Journal of Patient-Reported Outcomes 2026*, **10(Suppl 1)**:201.1

### Aims

Malignant small bowel obstruction (MBO) is associated with gastrointestinal symptoms and reduced health-related quality of life (HRQOL) in patients. The S1316 (NCT02270450) pragmatic comparative effectiveness trial demonstrated that surgical and nonsurgical management resulted in similar number of good days and overall survival in the first 91 days after registration. Here we present comprehensive PRO data from the S1316 trial.

### Methods

Patients with MBO with surgical indication were randomized (1:1) to surgical or nonsurgical management. Patients who declined random assignment could participate in an observational cohort. HRQOL was a secondary endpoint measured using the MD Anderson Symptom Inventory for Gastrointestinal Cancer (MDASI-GI) and the EQ5D-5L. Patients completed surveys in English or Spanish via paper surveys at baseline and telephone interview in follow-up weekly for 13 weeks, and every 4 weeks until 1 year after registration. EQ5D was assessed at baseline and weeks 2, 4, 8, and 12 only. Mean changes from baseline were compared over time between surgical and nonsurgical management groups using general linear mixed models (GLMMs). Mean quality-adjusted life weeks were estimated and compared between groups using areas under GLMMs.

### Results

41 patients (18 surgical, 23 nonsurgical) in the randomized cohort and 133 patients (53 surgical, 80 nonsurgical) in the observational cohort completed a baseline survey and at least one survey during follow-up. Mixed models indicated a significant group effect for MDASI-GI GI Symptom Severity (p=0.02) and Total Symptom Severity (p=0.049) scores. Significant differences in changes from baseline were observed at multiple weeks (Figure 1-2) favoring surgical over nonsurgical management. At 12 weeks using the EQ5D index, the estimated mean quality-adjusted life weeks for surgical versus nonsurgical management were 6.6 and 5.9 weeks, respectively, with a nonsignificant difference of 0.7 (95% CI, -0.4-1.8) weeks. Based on GI Symptom Severity (transformed to a 0–1 scale, with 1 representing no symptoms to mimic utility weights) up to week 53, the estimated mean quality-adjusted life weeks were 44.2 and 41.6 weeks−a significant difference of 2.6 (95% CI, 0.4-4.8) weeks.

### Conclusion

Surviving patients who were randomized or selected to have initial surgery had improved patient-reported HRQOL, supporting surgical management for MBO. Funding: NIH/NCI/NCORP grant UG1CA189974

**Fig. 1 (abstract 201.1):**
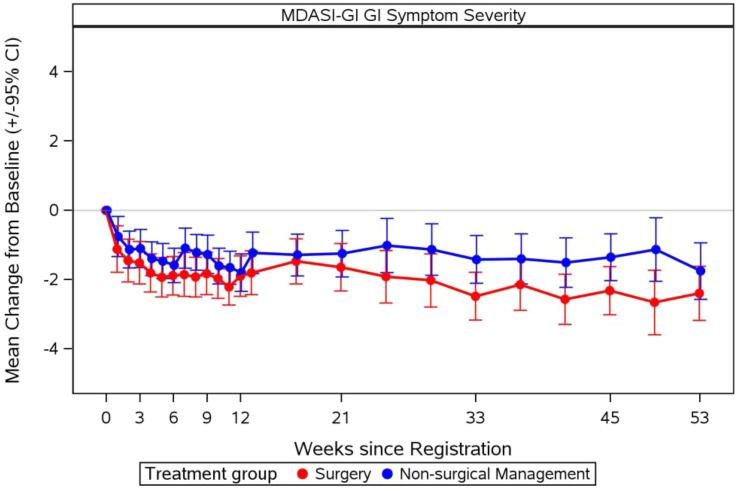
MDASI-GI GI Symptom Severity

**Fig. 2 (abstract 201.1):**
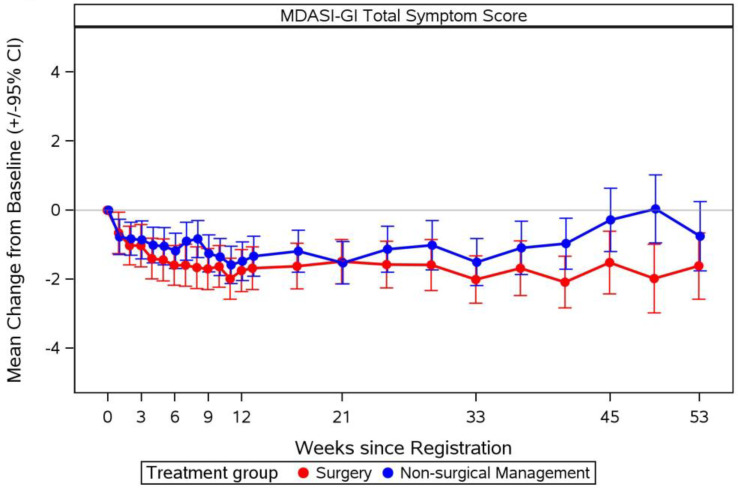
MDASI-GI Total Symptom Score

## 201.2 Patient-Reported Health Status Predicts Survival in Pancreatic Cancer: A Prospective Multicenter Cohort Study

Jingyu Zhang^1^, Kun Zhou^2^, Pin Chen^3^, Xiaobo Du^4^, Qiuling Shi^5^

^1^Chongqing Medical University, Chongqing, China, ^2^Second Affiliated Hospital of Chongqing Medical University, Chongqing, China, ^3^Chengdu Seventh People’s Hospital, Chengdu, China, ^4^Mianyang Central Hospital, Mianyang, China, ^5^State Key Laboratory of Ultrasound in Medicine and Engineering, Chongqing Medical University, Chongqing, China

*Journal of Patient-Reported Outcomes 2026*, **10(Suppl 1)**:201.2

### Aims

To investigate the prognostic value of patient-reported outcomes (PROs) for survival prediction in pancreatic cancer and evaluate their utility in risk stratification within real-world clinical settings.

### Methods

In this prospective multicenter cohort, 225 pancreatic cancer patients were consecutively enrolled from three tertiary hospitals (May 2022 onwards). Inclusion criteria required confirmed diagnosis, age ≥18 years, and baseline EQ-5D-5L completion. Trained staff collected ①Clinical characteristics via electronic health records; ②PROs using EQ-5D-5L with visual analog scale (EQ-VAS); ③Monthly survival status through structured telephone follow-up. Patients were stratified by baseline EQ-VAS scores: High health status (H-PRO) ≥80; Low health status (L-PRO) <80. Kaplan-Meier estimates and Log-rank tests compared survival between groups. Subgroup analyses focused on metastatic patients.

### Results

The analytic cohort comprised 225 patients stratified by baseline EQ-VAS: 117 (52.00%) in H-PRO (≥80) and 108 (48.00%) in L-PRO (<80) groups. Baseline characteristics were balanced between groups (Table 1), except for higher prevalence of distant metastasis in low health status group (67.29% vs 49.57%, P=0.01). With median follow-up of 5 months (IQR 2.50-8.00, Max 21.80 months), L-PRO group showed higher mortality risk than the H-PRO group (46/108, 42.59% vs 30/117, 25.64%), median survival difference: 7.07 vs 13.07 months (HR=0.42, 95%CI 0.27-0.68; log-rank P<0.001) (Figure A). In subgroup analysis of metastatic, 35 deaths (48.61%) occurred in the L-PRO groups and 19 deaths (32.76%) in the H-PRO, with a statistically significant difference in median survival time (5.77 vs 10.83 months, HR=0.41, 95%CI 0.23-0.73; log-rank P=0.002) (Figure B).

### Conclusion

This study demonstrates a close association between self-reported health status and survival rates in patients with pancreatic cancer, highlighting the critical role of PRO in survival prediction models for cancer patients. The≥80 EQ-VAS cutoff effectively stratifies mortality risk, particularly in advanced disease. These findings provide evidence for routinely integrating PRO assessments into clinical practice to identify high-risk individuals.

**Fig. (abstract 201.2):**
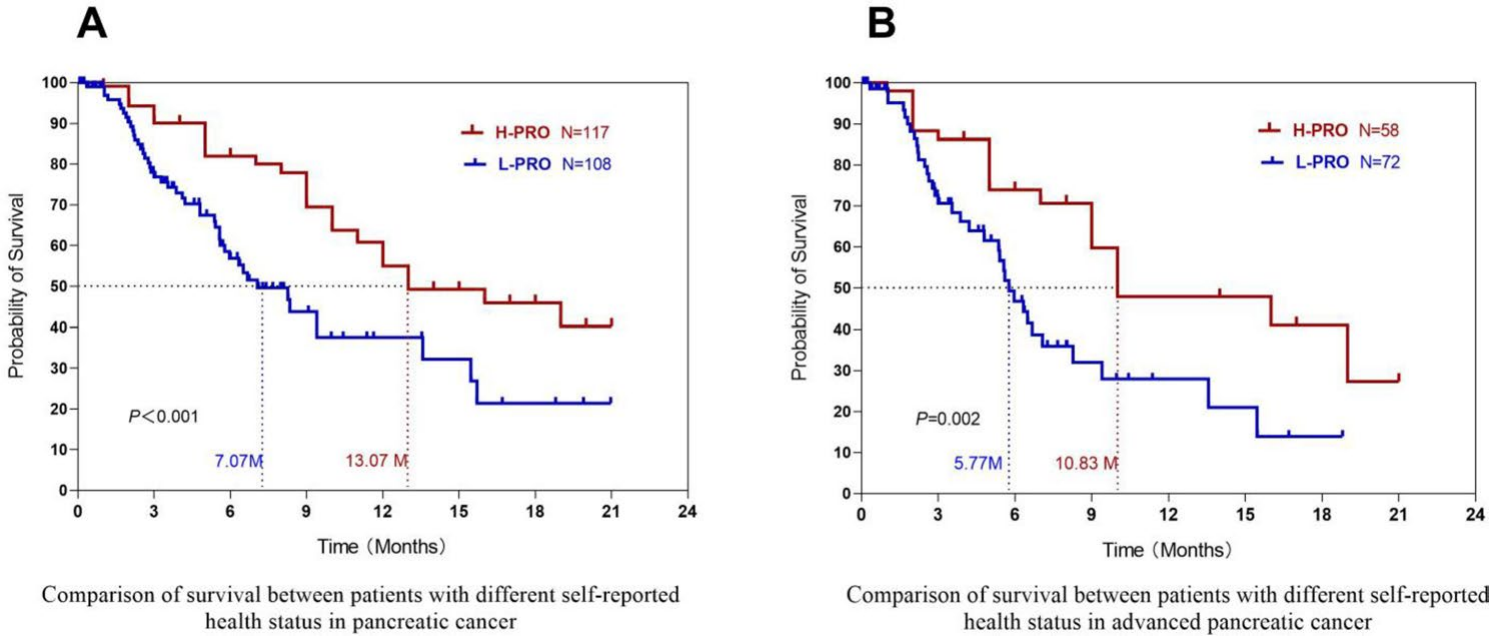
Comparison of survival between the H-PRO and L-PRO (**A**) in pancreatic cancer patients, (**B**) in advanced pancreatic cancer patients

**Table 1. (abstract 201.2):**
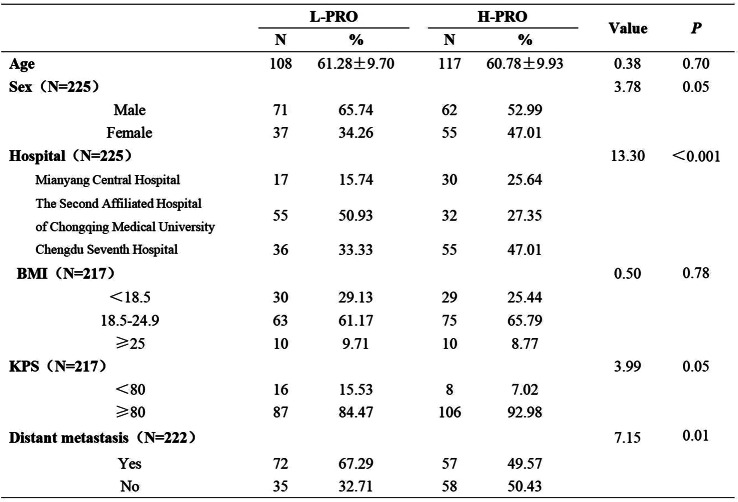
Demographic and Clinical Characteristics

## 201.5 Investigating the Mediating Effects within the Causal Chain of “FUAS Treatment—Pain Symptom Trajectory—Quality of Life” in Advanced Pancreatic Cancer Patients

Pan Ma^1^, Qiuling Shi^1^

^1^Chongqing Medical University, Chongqing, China

*Journal of Patient-Reported Outcomes 2026*, **10(Suppl 1)**:201.5

### Aims

Pain symptoms significantly impair the quality of life (QoL) of pancreatic cancer patients, making pain management and QoL improvement key therapeutic goals. Focused Ultrasound Ablation Surgery (FUAS) has shown potential in alleviating cancer-related pain. This study aims to quantify the mediating role of pain in the relationship between FUAS treatment and QoL using longitudinally collected, multi-time-point patient-reported outcomes (PROs).

### Methods

A prospective multicenter cohort of pancreatic cancer patients was analyzed. Structural Equation Modeling (SEM) was employed to construct a mediation model examining how pain transmits effects from FUAS treatment to QoL outcomes. The standardized effect sizes and significance levels of each pathway were calculated to elucidate the mechanisms underlying pain as a mediator.

### Results

Among 265 pancreatic cancer patients (121 in the FUAS treatment group and 144 in the non-FUAS group), the standardized effect size of path a (FUAS treatment → pain) was -0.234, indicating that the FUAS group exhibited a 0.234-unit reduction in pain compared to the non-FUAS group, demonstrating FUAS’s efficacy in pain alleviation. For path b (pain → QoL), the standardized effect size was -0.350, meaning each 1-unit increase in pain corresponded to a 0.350-unit decline in QoL. When mediation (pain) was incorporated into the model, the combined paths a×b explained 97.62% of the total treatment effect on QoL mediated through pain.

### Conclusion

This study elucidates the mediating mechanisms within the “FUAS treatment—pain symptom trajectory—quality of life” causal chain using time-dependent PROs. The findings confirm FUAS’s significant pain-relieving effects and provide a foundation for developing dynamic pain management strategies targeting pain pathways to optimize clinical outcomes. These results advance evidence-based approaches for integrating FUAS into multimodal cancer care.

**Fig. 1 (abstract 201.5a):**
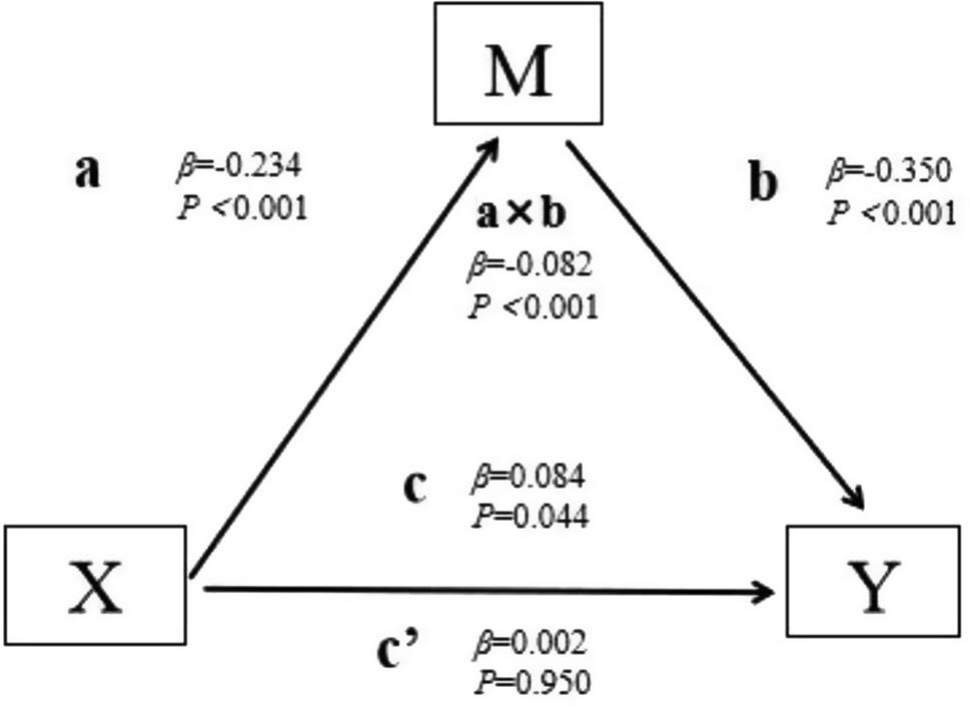
Investigating the Mediating Effects within the Causal Chain of “FUAS Treatment—Pain Symptom Trajectory—Quality of Life” in Advanced Pancreatic Cancer Patients

**Table 1(abstract 201.5b):**
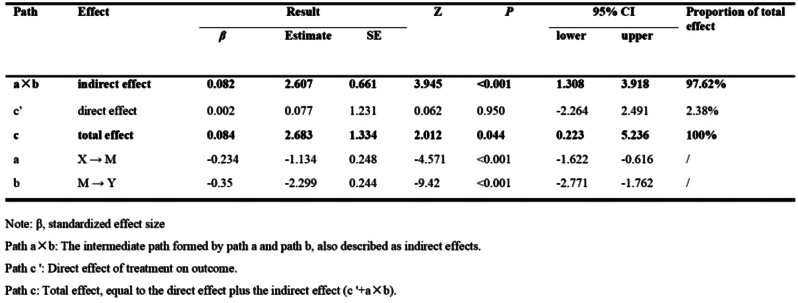
Path analysis of mediating effects of pain between FUAS and quality of life

## 201.3 Tai Chi Game Theory of Psychological Resilience in Pancreatic Cancer Patients: Based on Grounded Theory

Wen Zhou^1^, Cheng Lei^2^, Hongfan Yu^3^, Ping Chen^4^, Xiaojing Xue^5^, Gang Feng^5^, Qiuling Shi^1^

^1^State Key Laboratory of Ultrasound in Medicine and Engineering, Chongqing Medical University, Chongqing, China, ^2^Department of Thoracic Surgery, Sichuan Cancer Hospital, School of Medicine, University of Electronic Science and Technology of China, Chengdu, Sichuan China, ^3^State Key Laboratory of Ultrasound in Medicine and Engineering, College of Biomedical Engineering, Chongqing Medical University, Chongqing, China, ^4^Department of Oncology, Chengdu Seventh People’s Hospital, Chengdu, Sichuan, China, ^5^Department of Oncology, MianYang Central Hospital, Mianyang, Sichuan, China

*Journal of Patient-Reported Outcomes 2026*, **10(Suppl 1)**:201.3

### Aims

Pancreatic cancer, one of the most malignant tumors, imposes significant physical and psychological burdens on patients due to its severe symptoms and short survival period. Psychological resilience, a key construct in positive psychology, has been studied in lung and breast cancer patients，typically experience longer survival periods and lighter symptom burdens. However, this construct remains underexplored in patients with highly aggressive pancreatic cancer. This study employs grounded theory to investigate the psychological resilience of pancreatic cancer patients, aiming to construct a theoretical framework that elucidates the experience of living with high-burden malignancies.

### Methods

This study included Chinese patients with pancreatic cancer from July 2024 to February 2025. Semi-structured interviews were conducted. Analysis was completed in line with the process set out within constructivist grounded theory. Using constant comparison and memo writing, analysis moved from initial coding to focused coding, through to theoretical coding, resulting in the production of core concepts and categories, and theory development.

### Results

The study distilled two core concepts: (1) Burden Renders Patients Vulnerable and (2) Hope Empowers Patients. Despite awareness of the disease’s characteristics,patients maintained hope for survival, comfort from symptom alleviation, and care from healthcare providers. Some patients even engaged in self-hypnosis, fostering fantasy such as “this disease will disappear”, as a coping mechanism against their burdens. Severe symptom burdens (e.g., pain, fatigue, poor appetite) impaired functional abilities (e.g., walking, socializing), escalating family caregiving demands and financial strain, ultimately compelling patients to confront the reality of their illness,“It will not be worse any more”. Drawing on these findings, the study proposes a Tai Chi Game Theory Framework, formulating that the interplay between hope and burden exists in a dynamic yet fragile equilibrium.

### Conclusion

Pancreatic cancer patients navigate a complex interplay between hope for survival and the weight of their burdens characterized by a dynamic balance. Clinicians should leverage the Tai Chi Game Theory to mitigate patient burdens and amplify hope. We offers a novel paradigm for support systems in advanced cancer care, integrating cultural metaphor (Yin-Yang balance of Tai Chi philosophy) with clinical applicability. Future research could develop psychological resilience assessment tools and decision-support systems based on this model.

**Fig. 1 (abstract 201.3):**
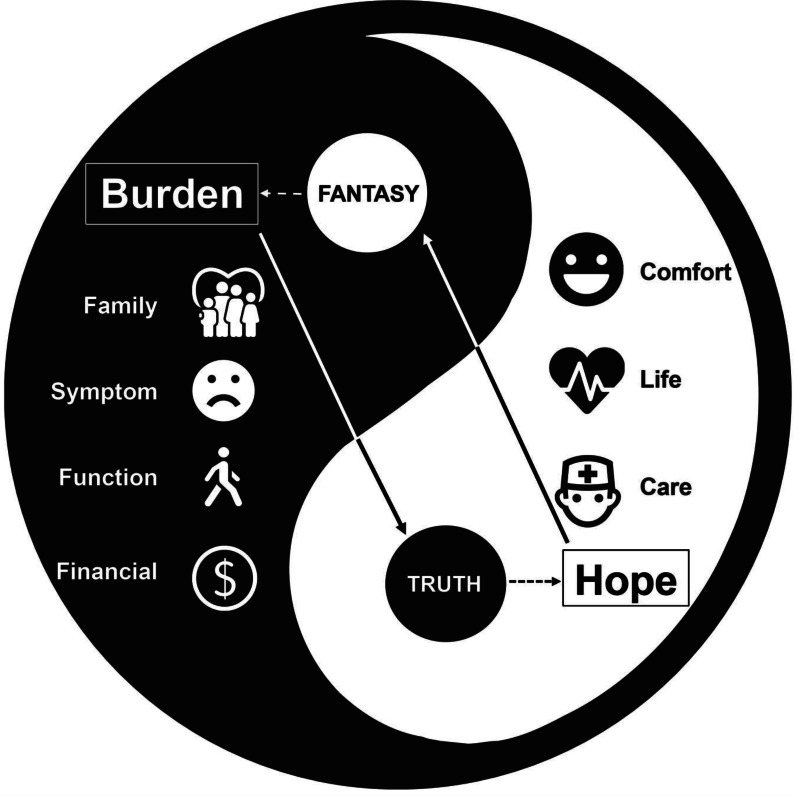
Tai Chi Game Theory of Psychological Resilience in Pancreatic Cancer Patients: Based on Grounded Theory

## 201.4 Pain Trajectory between Survival and Death Groups after Pancreatic Cancer Diagnosis: a Multicenter Longitudinal Cohort Study

Wen Zhou^1^, Cheng Lei^2^, Wenxi Li^3^, Hongfan Yu^4^, Wei Xu^5^, Xiaojing Xue^3^, Ping Chen^6^, Gang Feng^3^, Qiuling Shi^1^

^1^State Key Laboratory of Ultrasound in Medicine and Engineering, Chongqing Medical University, Chongqing, China, ^2^Department of Thoracic Surgery, Sichuan Cancer Hospital, School of Medicine, University of Electronic Science and Technology of China, Chengdu, Sichuan, China, ^3^Department of Oncology, MianYang Central Hospital, Mianyang, Sichuan, China, ^4^State Key Laboratory of Ultrasound in Medicine and Engineering, College of Biomedical Engineering, Chongqing Medical University, Chongqing, China, ^5^School of Public Health, Chongqing Medical University, Chongqing, China, ^6^Chengdu Seventh People’s Hospital, Chengdu, Sichuan, China

*Journal of Patient-Reported Outcomes 2026*, **10(Suppl 1)**:201.4

### Aims

Pancreatic cancer patients often experience significant pain-related symptom burden, which severely impacts quality of life and overall survival (OS) outcomes. However, the pain trajectory following pancreatic cancer diagnosis remains unclear. This study aims to analyze the symptom burden outcomes between survival group and death group from 1 to 9 months after diagnosis.

### Methods

Demographic and clinical characteristics were extracted from a prospective multicenter longitudinal cohort study of pancreatic cancer patients in China conducted between May 2022 and March 2024. Pain were assessed by Numerical Rating Scale (NRS), collected via face-to-face interviews or telephone follow-ups at monthly intervals after diagnosis. Univariate analysis was used for risk factors of death. Pain score trajectories were compared by linear mixed-effects model (Figure 1A), and the percentage of moderate-to-severe pain (score≥4) was calculated using generalized estimating equations (GEE) as shown in Figure 1B.

### Results

A total of 252 patients were included, of whom 160 (63.49%) were male. The mean age was 61.47 ± 10.14 years, and the mean BMI was 20.42±2.99. Approximately 70% of patients were in advanced stage, with a median survival time of 256 days. Univariate analysis identified pathology, transportation history, disease stage, surgical history, high-intensity focused ultrasound (HIFU) surgery history, systemic therapy during admission, and partial therapy during admission as risk factors for mortality. Significant differences in pain mean scores over nine months were observed between the survival group and death group (3.59 vs. 4.56, respectively). The percentage of moderate-to-severe level in the death group reached 100% from 6 to 9 months after diagnosis. Both the percentage of moderate-to-severe level and mean pain scores trajectories were significantly higher in the death group compared to the survival group (p < 0.001), primarily due to a higher proportion of severe level in the death group.

### Conclusion

The death group exhibited significantly higher pain scores and a greater proportion of moderate-to-severe pain because of higher incidence of severe level. Continuous monitoring of severe pain may have potential prognostic value for survival in pancreatic cancer patients. Medical staff in clinical settings should prioritize pain symptom monitoring, place emphasis on patient-reported pain outcomes, and pay special attention to severe pain.

**Fig. 1 (abstract 201.4a):**
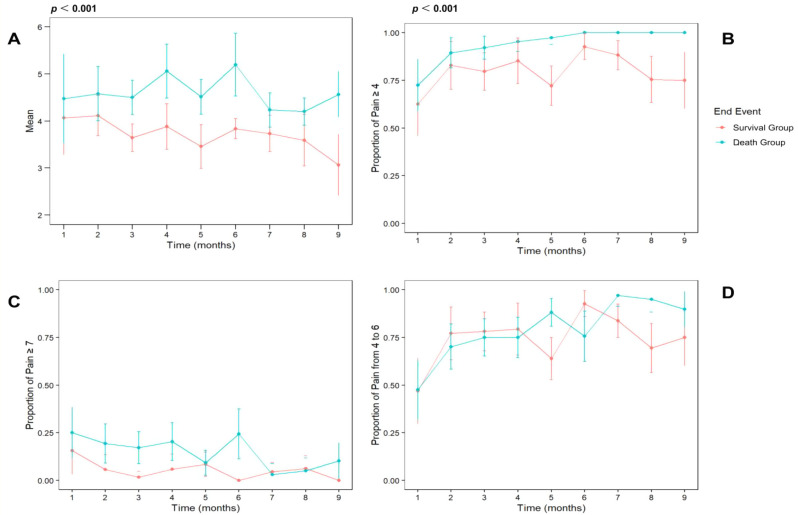
Pain Trajectory between Survival and Death Groups after Pancreatic Cancer Diagnosis: a Multicenter Longitudinal Cohort Study

**Table 1 (abstract 201.4b):**
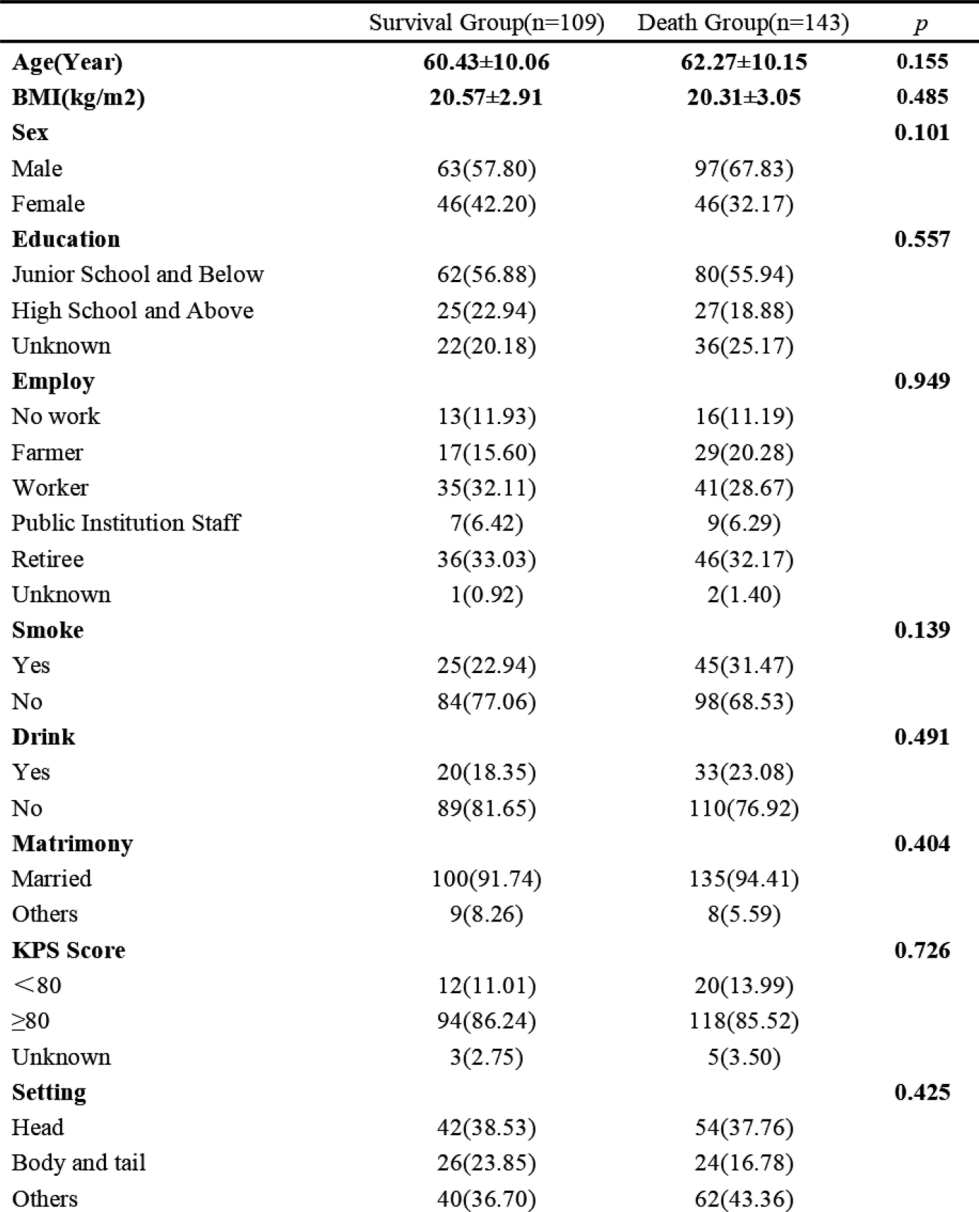

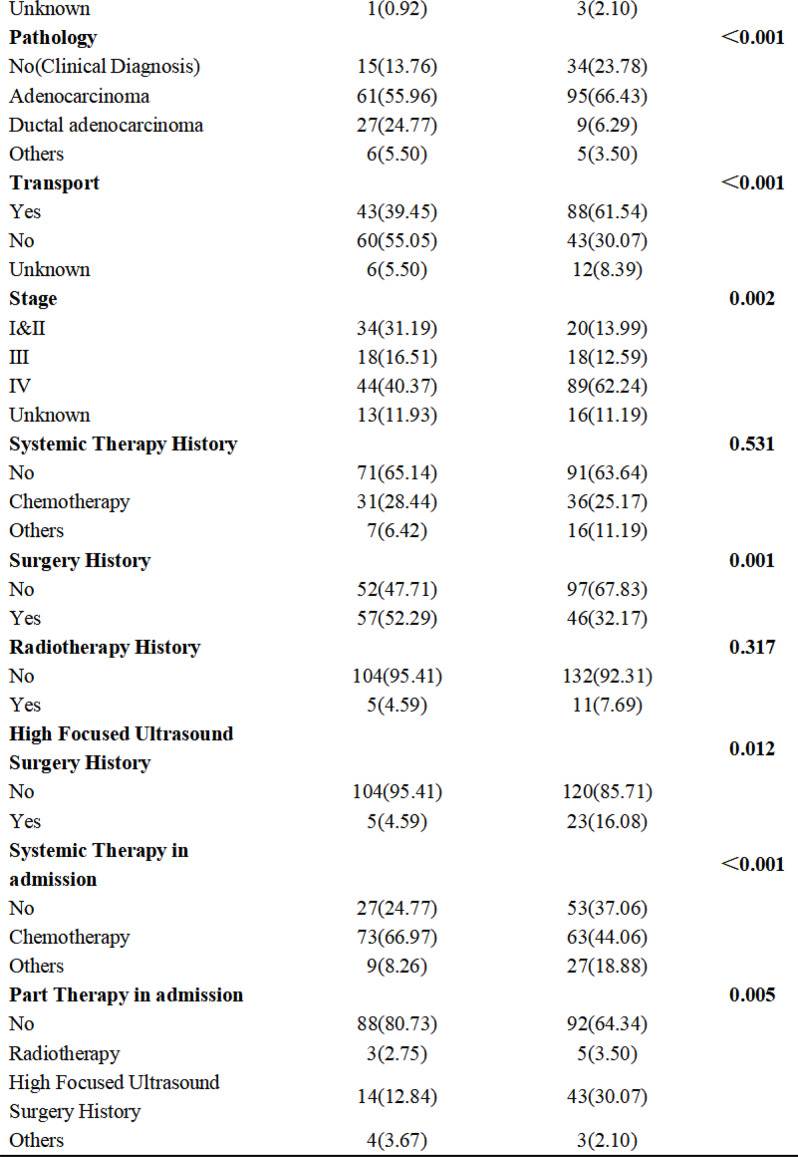
Demographic and clinical characteristics of participants

## 202.2 Response Shift in Patient-Reported Outcome Assessment among Older People Living with HIV/AIDS

Renjun Gu^1^, Huayu Li^2^, Yiqing Weng^1^, Jia Li^1^, Xinhui Xu^1^, Yuyuan Zhong^1^, Jing Hu^1^, Hongmei Wang^2^

^1^School of Public Health, Zhejiang University, Hangzhou, China, ^2^Department of Social Medicine of School of Public Health, Department of Pharmacy of the First Affiliated Hospital, Zhejiang University School of Medicine, 866 Yuhangtang Road, Hangzhou, 310058, Zhejiang, China

*Journal of Patient-Reported Outcomes 2026*, **10(Suppl 1)**:202.2

### Aims

Response Shift (RS) effect refers to a discrepancy between observed changes and target constructs’ changes. It may violate patient-reported outcome (PRO) measures’ application principle of Longitudinal Measurement Invariance and confound longitudinal PRO assessment. This study aimed to analyze the occurrence of RS and its effect on longitudinal PRO assessment among older people living with HIV/AIDS (PLWHA) from both domain- and item-level perspectives.

### Methods

Two time-point surveys at a 3-month interval were conducted. Totally 1245 cases of older PLWHA participated at baseline survey and 1057 cases at follow up. The PROHIV-OLD instrument and general information were collected. Oort Structural Equation Method was performed dimensionally to detect Reconceptualization, Recalibration and Reprioritization types of RS with Mplus 8.0. ROSALI-RMT method (using the five-item Mental Status dimension as an example) was performed to detect Recalibration at item level with Stata 15.0, integrating group effects of diagnostic durations, disease stages, and HIV-1 RNA levels. The effect of Differential Item Functioning (DIF) was also examined.

### Results

Dimensionally, Physical Symptom (Est.=+0.650, Δχ2(1)=86.217) and Mental Status (Est.=-0.276, Δχ2(1)=18.466) were detected with moderate and small uniform Recalibration, respectively. Family Relationship was detected with small Reconceptualization (Est.=0.120, Δχ2(1)=3.912) and moderate Reprioritization (Est.=-0.584, Δχ2(1)=23.248). Treatment Dimension was detected with non-uniform Recalibration (Δχ2(1)=21.728). All above were statistically significant (P<0.05). At item level, DIF was not found, while positive RS occurring in newly diagnosed patients (Est.=+0.34) led to underestimate Mental Status. Negative RS in patients diagnosed for more than one year (Est.=-0.09) led to overestimate Mental Status. The MS(RS-adjusted) of two subgroups were 1.32 and -0.51, respectively. Regarding different disease stages, negative RS made Mental Status overestimated among patients at stage I (Est.=-0.13) and stage II (Est.=-0.02). Positive RS (Est.=+0.09) made Mental Status underestimated among patients at AIDS-stage. The MS(RS-adjusted) were 0.51, -0.23, and 0.61, respectively. Positive RS was detected only in the HIV-1 RNA Undetected group (Est.=+0.26), making Mental Status underestimated. The MS(RS-adjusted) was 0.85.

### Conclusion

This study found that RS effect would influence PRO measures’ longitudinal scores in older PLWHA. It would provide new perspectives on accurate longitudinal PRO assessment, inform methodological integration of multilevel RS studies.

## 202.4 Evaluation of demographic DIF in the PROMIS and EORTC cognitive function measures in a nationally representative US sample

Miranda Kapfhammer^1^, Benjamin Schalet^2^, Kathryn Flynn^1^, Bronwen Shaw^1^, Rachel Cusatis^1^

^1^Medical College of Wisconsin, Milwaukee, Wisconsin, USA, ^2^Amsterdam UMC, Amsterdam, Netherlands

*Journal of Patient-Reported Outcomes 2026*, **10(Suppl 1)**:202.4

### Aims

PROMIS and EORTC cognitive function (CF) are widely used measures, yet neither has been evaluated for demographic differential item functioning (DIF). We evaluated DIF in the PROMIS CF item bank and the EORTC CF subscale across demographic groups and described the correlation between PROMIS and EORTC CF scores.

### Methods

Data was collected in July 2024 using the YouGov online panel. The initial sample of 3,304 respondents was matched down to 2,470 using a sampling frame on age, gender, race, and education based on the American Community Survey. A final weight was developed with propensity scores and used for linear regression analysis. DIF was assessed for sex (male, female), age (under 55, 55 and above), race (White, Black, Hispanic, other), and income (below 200% of the poverty line, above poverty line). DIF analyses were conducted on all 32 PROMIS CF items and the 2-item EORTC QLQC-30 CF subscale using the lordif package in R. McFadden’s pseudo R2 change for a total DIF effect > 0.02 indicated noteworthy DIF. Weighted bivariate linear regression analysis assessed score differences across demographics and health conditions separately for PROMIS-8a short form and EORTC QLQC-30 CF subscale. Pearson correlation assessed the correlation between PROMIS and EORTC scores.

### Results

Most respondents were over the age of 55 (64.5%), White (47.7%), and had income above the threshold (54.9%). All demographic DIF effects were below the R2 cutoff for both PROMIS and EORTC. Participants who were male, over the age of 55, Black, did not provide income data, and did not experience headaches, depression, anxiety, or have cancer, scored significantly higher on PROMIS. EORTC results aligned, except no significant differences were found by sex or cancer status. The Pearson correlation between EORTC and PROMIS scores was 0.70.

### Conclusion

The absence of noteworthy DIF provides confidence for the use of PROMIS and EORTC to measure CF across sociodemographic subgroups. PROMIS and EORTC measures showed similar CF score patterns, though EORTC scores did not differ significantly by sex and cancer status while PROMIS scores did. Future research is needed to investigate the relationship between the measures, including conducting a crosswalk directly comparing scores.

**Fig. 1 (abstract 202.4a):**
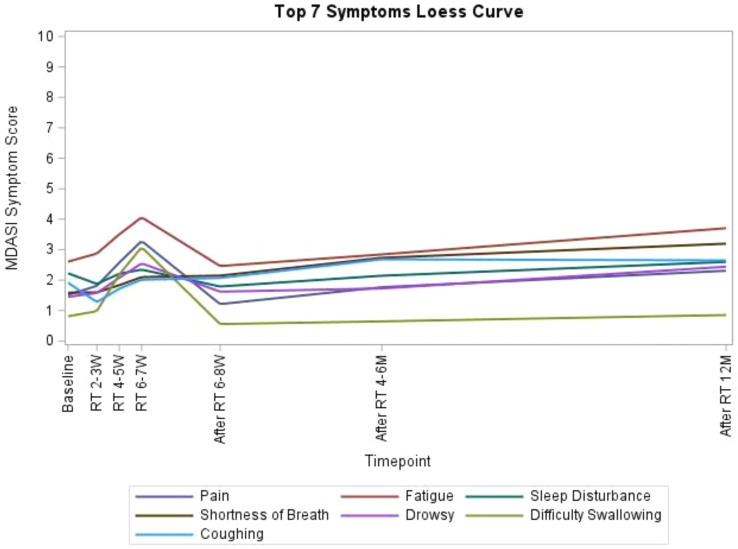
Evaluation of demographic DIF in the PROMIS and EORTC cognitive function measures in a nationally representative US sample

**Fig. 2 (abstract 202.4b):**
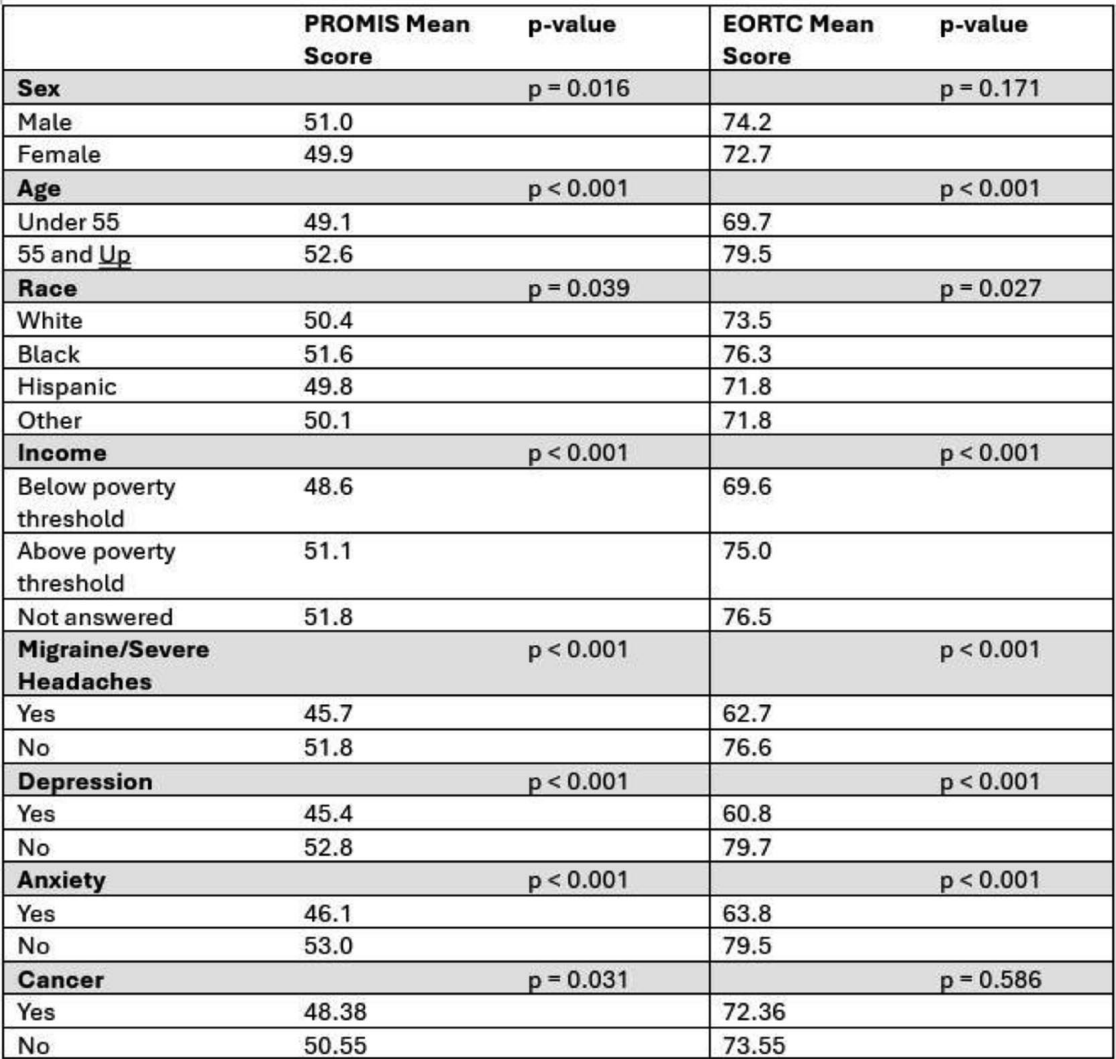
Results of weighted bivariate linear regression analysis and adjusted Wald tests

**Fig. 1 (abstract 202.4c):**
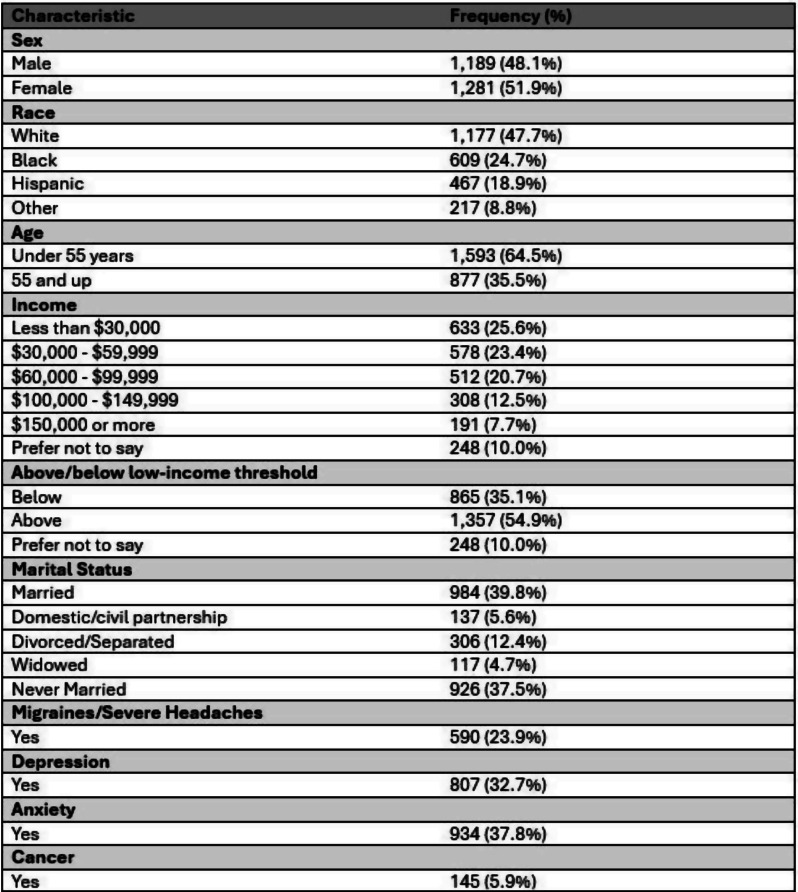
Sociodemographic characteristics of sample (n=2,470)

## 202.5 Predictors of missing instruments in a postoperative lung cancer cohort

Jian Li^1^, Shizhu Li^1^

^1^Chongqing Medical Univerisity, Chongqing, China

*Journal of Patient-Reported Outcomes 2026*, **10(Suppl 1)**:202.5

### Aims

Missing patient-reported outcome (PRO) data can significantly impact the validity of cohort studies. Identifying predictors of missing instruments is crucial for developing targeted strategies to mitigate this issue. This study aimed to investigate the factors associated with missing PRO instruments on the day of discharge in a postoperative lung cancer cohort.

### Methods

This is a prospective observational analysis of a PRO database collected as part of routine clinical care after surgery in a lung cancer cohort. The investigation examines PRO completion rates from preoperative assessment through the first postoperative week. Univariate and multivariate logistic regression analyses were performed to identify factors associated with missing PRO data on the day of hospital discharge.

### Results

This study analyzed perioperative PRO data for 2079 patients, each of whom should have completed 12 longitudinal assessments. Data were collected through a Wechat-based program and paper forms (Perioperative Symptom Assessment for Lung surgery,PSA_lung) as part of routine clinical care. The preoperative phase had the lowest rate of missing items (1.89%), while the day of discharge exhibited the highest rate of missing data (42.76%), including missing items (4.52%) and missing instruments (38.24%).(Figure 1) Multivariate analysis revealed that younger age, being married, and higher education levels were significantly associated with lower missing rates.(Table 2).

### Conclusion

The completion rates of PRO on the day of discharge were significantly low in this lung cancer cohort, which was associated with specific patient characteristics. Identifying the root predictors of missing data could improve the clinical utility of PRO and offer opportunities for personalized care.

**Fig. 1 (abstract 202.5a):**
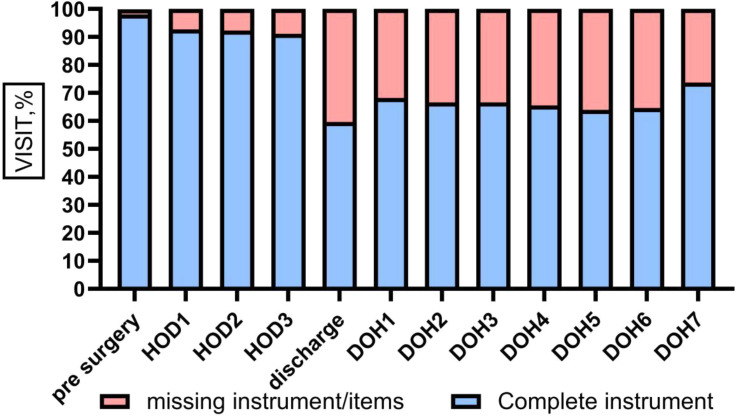
Predictors of missing instruments in a postoperative lung cancer cohort

**Table. 1 (abstract 202.5b):**
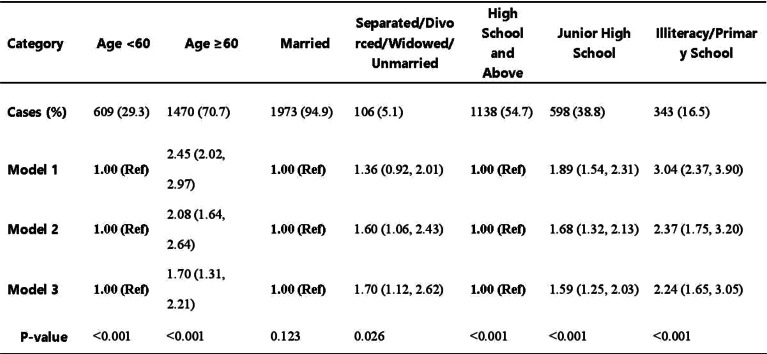
Predictors of missing instruments in a postoperative lung cancer cohort

**Table 1 (abstract 202.5c):**
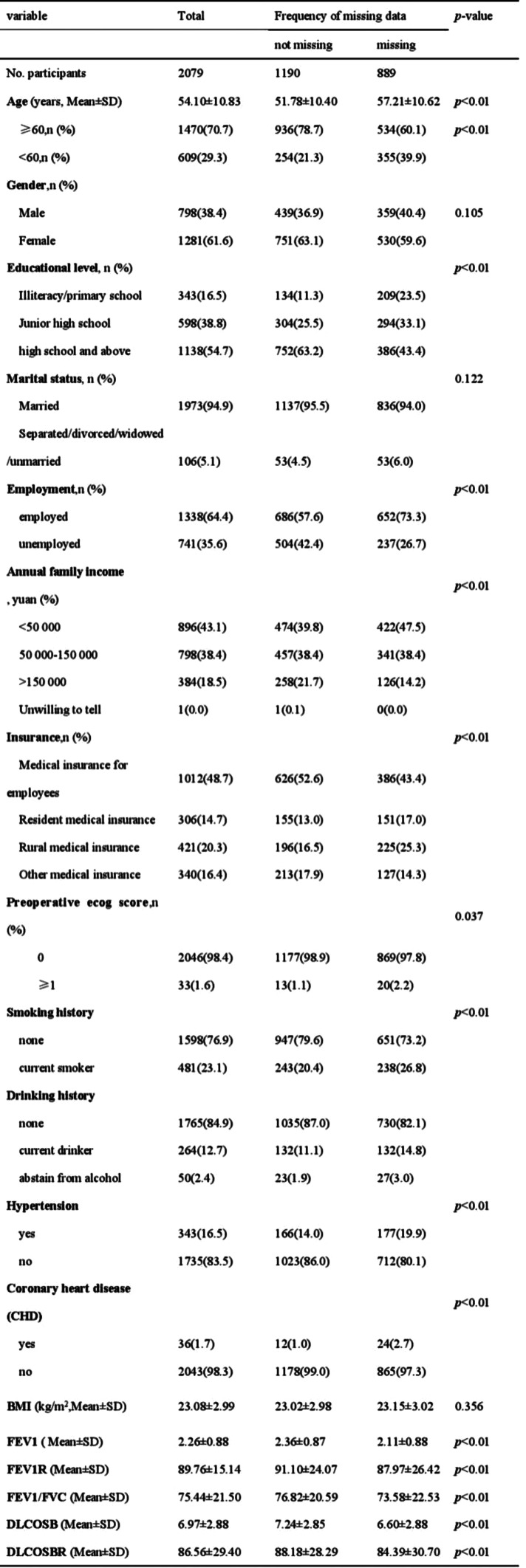
Predictors of missing instruments in a postoperative lung cancer cohort

## 202.3 A tree-based graded response theory model to test for differential item functioning in patient-reported outcome measures

Olayinka Arimoro^1^, Matthew James^1^, Maria Santana^1^, Lisa Lix^2^, Tolulope Sajobi^1^

^1^University of Calgary, Calgary, Alberta, Canada, ^2^University of Manitoba, Winnipeg, Manitoba, Canada

*Journal of Patient-Reported Outcomes 2026*, **10(Suppl 1)**:202.3

### Aims

Item-response theory models based on recursive partitioning have been developed for evaluating differential item functioning (DIF) in patient-reported outcome measures and for identifying population subgroups that exhibit DIF. However, existing methods have only been developed for Rasch models, which assume that all the items are equal at distinguishing between individuals with different levels of the latent construct. The study describes the implementation of the tree-based graded response model (GRMTree) to test for DIF on the Medical Outcomes Study Social Support Survey (MOS-SS).

### Methods

Data were from individuals with coronary artery disease who completed the eight items of the emotional domain of the MOS-SS two weeks after receiving cardiac catheterization in Alberta, Canada. GRMTree was used to identify subgroups of individuals for which the GRM parameters (discrimination and threshold parameters) were non-invariant across subgroups. Patients’ characteristics (age, sex, education, residency, body mass index, employment status, smoking status, depression, and multimorbidity) were included as covariates while Bonferroni correction and a minimum node sample size of 400 were imposed on the model to avoid model overfitting.

### Results

Of the 5,305 patients included, 1,037 (19.5%) were female, and the mean (standard deviation) age was 65.1 (10.6) years. GRMTree identified three distinct subgroups exhibiting DIF on the MOS-SS emotional domain, defined by age and sex, indicating that these covariates were associated with DIF in patients’ responses. Subgroup 1 was ≤62 years (n = 2,141), subgroup 2 was females >62 years (n = 666), and subgroup 3 was males >62 years (n = 2,498). Items “someone you can count on to listen to you,” “someone to give you information,” “someone to confide in,” “someone to share your most private worries or fears,” and “someone who understands your problem” showed a change in thresholds ranging from 0.60 to 1.16 between subgroups. Additionally, the item “someone to confide in or talk to about yourself or your problems” showed a change in discrimination = 0.81 between subgroups 2 and 3.

### Conclusion

Only demographic covariates were associated with DIF on the MOS-SS emotional domain. The GRMTree is a useful methodology to test for DIF in potentially heterogeneous populations.

**Fig 1(abstract 202.3):**
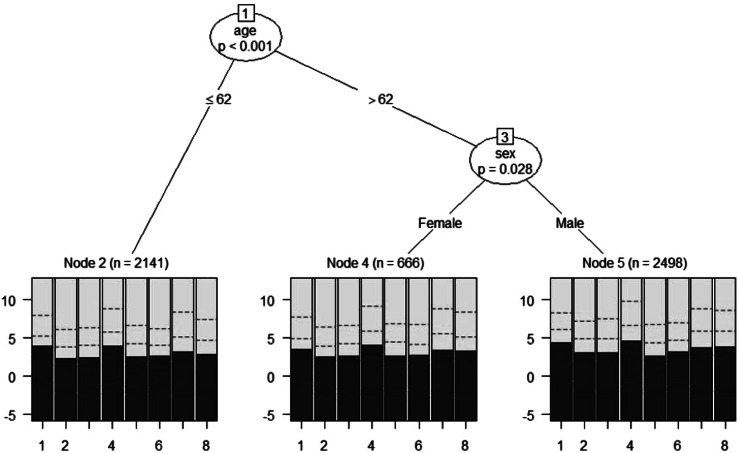
(Region) plot of GRMTree of MOS-SS emotional domain at baseline (2 weeks)

**Table 3 (abstract 202.3):**
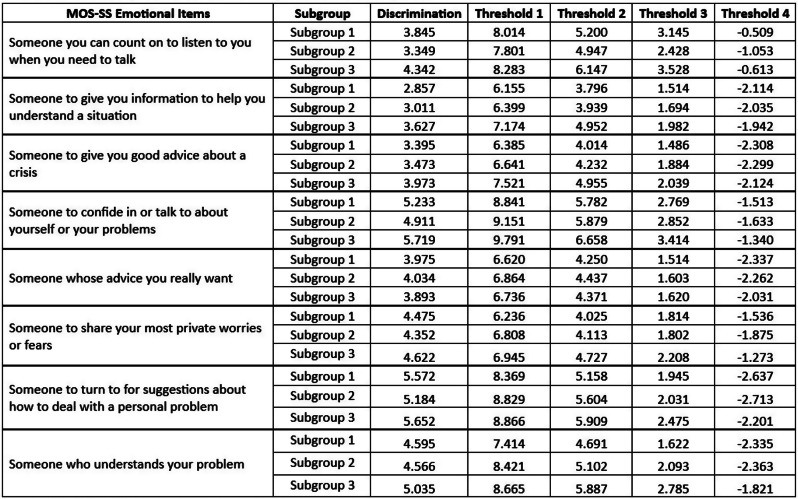
Item discrimination and threshold parameters across subgroups

## 202.1 Evaluating Responsive Shift in Total Knee Arthroplasty Patients

Ademola Itiola^1^, Tolulope Sajobi^2^, Deborah Marshall^2^, Jeffrey Johnson^1^

^1^School of Public Health, University of Alberta, Edmonton, Alberta, Canada, ^2^Department of Community Health Sciences, Cumming School of Medicine, University of Calgary, Calgary, Alberta, Canada

*Journal of Patient-Reported Outcomes 2026*, **10(Suppl 1)**:202.1

### Aims

After undergoing total knee arthroplasty (TKA), patients may adjust their internal standards (recalibration), reframe how they conceptualize certain questions (reconceptualization) and even shift their priorities (reprioritization) over time. These phenomena, collectively known as response shift (RS), can make patient-reported outcome measures (PROMs) scores taken at different time points—such as before and after surgery— sometimes not directly comparable. Ignoring this shift may lead to inaccurate estimation of intervention effect. As there is limited understanding of RS among total knee arthroplasty (TKA) patients, we explored RS in PROM of TKA patients using extant administrative health data.

### Methods

We identified a cohort of 3,389 individuals who had completed EQ-5D-5L pre-surgery and at 3- and 12-months post-surgery between 2013 and 2023 in Alberta, Canada. RespOnse Shift ALgorithm in Item response theory Rasch Measurement Theory (ROSALI-RMT) was used to test for recalibration RS on EQ-5D-5L item(s). We also examined the association between biological sex, age and presurgical WOMAC physical function (PF) score on response shift effect on EQ-5D-5L items while adjusting for baseline differential item functioning. We assessed partial credit model fit at baseline using infit and outfit indices and visualization of item and test characteristic curves.

### Results

The average age of the cohort was 66.99 years (SD=8.41), 61.61% were female and only 20.15% had no comorbidity. All EQ-5D-5L items, except for pain and discomfort at 3 months and mobility at 12 months, were susceptible to non-uniform recalibration RS. The RS-adjusted intervention effect was larger than the unadjusted intervention effect at 3 months (-1.97 [-1.84, -2.10] vs -1.58 [-1.48, -1.68]) and 12 months (-1.69 [-1.58, -1.81] Vs -1.41 [-1.32, -1.50]) with an attenuation of RS effect on the intervention effect over time. Response shift effect varied with age, sex, and presurgical WOMAC PF score. At 12 months, the unadjusted and RS-adjusted intervention effect were similar for males and females, higher for patients <65 years and those with WOMAC PF score of ≤ 31.

### Conclusion

TKA patients experienced recalibration RS, potentially overestimating presurgical health-related quality of life due to disease adaptation. The impact of total knee arthroplasty on patients’ HRQOL may be underestimated when response shift effect is ignored.

## 203.1 Implementation of PROMIS Global Health as a Screener to Trigger Construct-Specific PROs in Clinical Care: a mixed methods study of patients’ perspectives

Brittany Lapin^1^, Yadi Li^1^, Andrew Schuster^1^, Irene Katzan^1^

^1^Cleveland Clinic, Cleveland, Ohio, USA

*Journal of Patient-Reported Outcomes 2026*, **10(Suppl 1)**:203.1

### Aims

Patient-reported measures of health-related quality of life (HRQL), such as PROMIS Global Health (GH), are collected across healthcare systems to track patient health status and monitor changes over time. Many systems additionally collect condition-specific patient-reported outcome measures (PROMs). In the effort to reduce burden and tailor administration to individual patient needs, our study evaluated the patient perspective following implementation of a system to use PROMIS-GH as a screener to trigger additional construct-specific PROMs.

### Methods

A mixed-methods study evaluated patient perspective following implementation of a patient-entered data algorithm in 3 clinical areas (general neurology, functional medicine, and rehabilitation medicine) of a large health system. As standard care, patients completed PROMIS-GH and construct-specific PROMIS computer adaptive tests (CATs). Starting June 2023, only patients who at least moderately endorsed PROMIS-GH items received additional construct-specific CATs (ie PROMIS-GH fatigue item triggered PROMIS Fatigue v1.0). Patients also completed an 11-question survey on their perception of and satisfaction with the patient-entered data system pre-algorithm (9/19/22-10/18/22) and post-algorithm (12/5/23-2/1/24) implementation which were compared using t-test. Additionally, a focus group including 7 patients was conducted with themes analyzed using content analysis framework.

### Results

Surveys were completed by 323 patients (average age 56.0±14.9 years, 75.5% female) pre-implementation and 624 patients (56.8±15.4, 70.1% female) post-implementation. There were no significant differences in patient perception or satisfaction with the questionnaire sets from pre- to post-implementation, with patients agreeing that both questionnaire sets were appropriate lengths (89.6% vs 91.6%), had relevant content (81.0% vs 85.1%), improved communication with providers (37.4% vs 33.7%) and control over their own care (40.1% vs 35.6%). All participants in the focus group preferred the new algorithm for its brevity, relevance, and ability to have control over content.

### Conclusion

Our study provides support for the feasibility of implementing PROMIS-GH items as screening tools to identify patients who would most benefit from additional construct-specific PROMs. Satisfaction with the patient-entered data system remained high pre- and post-implementation. Patients preferred the new algorithm integrating PROMIS-GH items as a screening tool, valuing its potential to reduce questionnaire burden while tailoring the surveys to address their individual health.

## 203.3 Psychometric properties of CHOICEs-22, a patient reported experience measure of shared decision-making in perinatal care

Rachel Breman^1^, Ester Villalongo Olives^2^, Crystal Trent Paultre^1^, Alexandra Mora^1^

^1^University of Maryland, School of Nursing, Baltimore, Maryland, USA, ^2^University of Maryland, School of Pharmacy, Baltimore, Maryland, USA

*Journal of Patient-Reported Outcomes 2026*, **10(Suppl 1)**:203.3

### Aims

This study tested the psychometric properties of Childbirth Options, Information, and person-Centered Explanation (CHOICEs)-22, revised from CHOICEs-15. CHOICEs-22 addresses interactions during prenatal and intrapartum care using a patient centered approach.

### Methods

Based on qualitative interviews additional items were added to CHOICEs-15. The new CHOICEs-22 was translated into Spanish. To test the revision of the measure, postpartum people were recruited from the What to Expect and Baby Center pregnancy tracking and parenting apps, e-mails and advertisements posted on social media. Inclusion criteria were: age >18 and a recent birth in a U.S. hospital. Internal consistency of CHOICEs-22 was evaluated using a Rasch measurement model and item reliability. Differential Item Functioning (DIF) analysis was done to determine measurement invariance between Asian versus White, Black versus White, English versus Spanish speaking, low versus higher income, married versus unmarried, those with private or public insurance, young versus older individuals, and single versus multiple parity participants. The Mothers on Respect index (MOR) was used for hypothesis testing that the concepts are associated.

### Results

A total of 5820 surveys were completed. Twenty-seven percent were Hispanic/Latino/a, and 61% were White. The majority had an income of greater than or equal to 50,000 dollars per year (57%) and the majority had private insurance (58%). Reliability was supported with an alpha coefficient of .70. Validity was supported in that all the items fit with the concept of shared decision-making except for four OUTFIT statistics. We found evidence of DIF between Black and White participants regarding one item and between Hispanic/Latino/a and White participants on 3 items. The DIF was small overall. Validity based on hypothesis testing noted that, after controlling for age, primary English or Spanish speaking, race, or having one or more births, the MOR index score was associated with CHOICEs-22 (F=5.45, p =.02).

### Conclusion

There was evidence of support for the reliability and validity of CHOICEs-22. The measure is brief and can be used for research or clinical care to identify and improve shared decision-making during the prenatal and childbirth periods.

## 203.5 Impact of demographics, information access, and patient involvement in treatment decision-making on quality of life in leukaemia: insights from a global survey

Sam Salek^1^, Sarah Gunn^2^, A. J. Poots^2^, Esther Oliva^3^, Tatyana Ionova^4^, Samantha Nier^5^

^1^University of Hertfordshire, Hatfield, UK, ^2^Picker Institute Europe, Oxford, UK, ^3^London North West University Healthcare NHS Trust, London, UK, ^4^Saint Petersburg State University Hospital, St Petersburg, Russia, ^5^Acute Leukaemia Advocates Network (ALAN), Bone, Switzerland

*Journal of Patient-Reported Outcomes 2026*, **10(Suppl 1)**:203.5

### Aims

Leukaemia is a blood cancer that impacts survival and patients’ quality of life (QoL). The Haematological Malignancy Patient-Reported Outcome (HM-PRO) is a validated tool, assessing symptom burden and impact of disease on QoL, comprisimg Part-A (physical, social well-being, emotional behaviour, eating/drinking habits) and Part-B (symptoms). Higher scores indicate worse QoL. Research is limited using HM-specific tools in leukaemia subtypes on how demographics, information, access, and treatment-decision involvement influence QoL. We aimed to address that research gap.

### Methods

Methods An online global cross-sectional survey conducted in 2023 recruited patients with leukaemia. The survey included the HM-PRO, demographics, and personal experiences. We used Kruskal-Wallis (KW) tests, Bonferroni-corrected for multiple comparisons.

### Results

2260 participants (mean age=53.8; standard deviation=16.5; female=1251, 55.4%) from 64 countries completed the survey). 1993 (88.2%) completed HM-PRO Part-A and 1951 (86.3%) completed HM-PRO Part-B.We report diferences where KW tests returned a p-value less than the Bonferroni-adjusted 95% threshold (p<0.00125).Patients with acute lymphoblastic leukaemia (ALL, n=219) and acute myeloid leukaemia (AML, n=278) reported lower QoL than people with chronic lymphocytic leukaemia (CLL, n=771) or chronic myeloid leukaemia (CML, n=601); medians (Mdn) 20, 18, 11, and 14 respectively.For HM-PRO Part-B, ALL (n=211, Mdn=8) and CML (n=589, Mdn=7) had lower symptom burden compared to AML (n=271, Mdn=5) and CLL (n=759, Mdn=5).Patients aged 18–25 had lower QoL for HM-PRO Part-A (n=101, Mdn=22) and lower symptom burden for Part-B (n=97, Mdn=8).Generally, the more information recieved resulted in better QoL. However, for HM-PRO Part-A, respondents to having “been given clear diagnostic information” answering “No” or “Yes, to some extent” had lower QoL (Mdn=16), compared to “Yes, definitely” (Mdn=12). We found a similar pattern for HM-PRO Part-B and the sensitivity of information delivery (Mdns: 7 vs. 5).

### Conclusion

We highlight the need for tailored support, particularly for younger patients and people diagnosed with ALL or AML. Effective communication and patient engagement can help mitigate the impact of leukaemia on QoL. Clinicians should prioritise clear, compassionate information-sharing, and collaborative decision-making to enhance patients’ experiences. Ensuring patients are well-informed and involved in their care could be key for improving QoL.

## 203.2 Assessment of patient needs and treatment benefits - a scoping review on the Patient Benefit Index (PBI)

Stefanos Boudouroglou-Walter^1^, Chantal Wieting^1^, Patricia Vester^1^, Matthias Augustin^1^, Christine Blome^1^

^1^University Medical Center Hamburg-Eppendorf, Hamburg, Germany

*Journal of Patient-Reported Outcomes 2026*, **10(Suppl 1)**:203.2

### Aims

The Patient Benefit Index (PBI) assesses patient-relevant treatment benefits using a two-step approach: measuring patient needs pre-treatment and goal attainment post-treatment. The global PBI score reflects post-treatment benefits weighted by the importance of each pre-treatment need. Implementing the PBI in clinical practice may enhance patient-centeredness. This scoping review aims to collate the existing literature on the PBI, abridge the development and validation of instruments following its methodology, and explore applications in research and clinical care, with a focus on healthcare provision and quality implications.

### Methods

The review adhered to PRISMA-ScR guidelines and employed systematic literature searches across five electronic databases, supplemented with manual snowball searches. The JBI manual for evidence synthesis guided the study, and the protocol had been registered with the Open Science Framework (OSF).

### Results

We included 143 publications, with 85.3% from dermatology, 5.6% from angiology/vascular surgery, and 9.1% from other areas like oncology and neurology. We identified 26 development/validation studies encompassing 18 PBI versions for different conditions or target groups, 42 real-world applications, 18 clinical trial uses, and 57 other research uses, including treatment needs elicitations and cost-effectiveness analyses. Of the publications utilizing a PBI, 62 were psoriasis-related, with a rise in publications for non-dermatology indications in recent years. In 20 publications that compared findings by patient group, 13 revealed significant subgroup differences in needs or benefits. No use cases in clinical practice were identified, but 76 publications suggested potential implications for healthcare provision and quality. Seven studies specifically examined differences in treatment assessment between physicians and patients.

### Conclusion

The findings highlight the importance of disease-specific patient-reported outcome measures that capture patient preferences and treatment goals for optimizing treatment decisions. Patient needs often extend beyond specialty-specific ailments and may also diverge from physician assessments, indicating that patient-defined benefits surpass mere symptomatic relief. Traditional measures of disease severity may not encompass the overall disease burden, establishing the PBI as a valuable instrument for patient-reported treatment evaluation. Notable gaps remain regarding its practical implications in routine healthcare settings.

## 203.4 Framework for patient-reported measurement of diagnostic excellence

Vadim Dukhanin^1^, Lakshmi Krishnan^2^, Anushka Jajodia^3^, Kelly Gleason^3^, Kathryn McDonald^3^

^1^Johns Hopkins Bloomberg School of Public Health, Baltimore, Maryland, USA, ^2^Georgetown University School of Medicine, Washington, District of Columbia, USA, ^3^Johns Hopkins University School of Nursing, Baltimore, Maryland, USA

*Journal of Patient-Reported Outcomes 2026*, **10(Suppl 1)**:203.4

### Aims

Diagnostic excellence is an emerging construct that could be materialized by capturing relevant domains of experiences and outcomes reported by patients and care partners. Characterization of when, where, and how these domains can be assessed, while considering populations most vulnerable to diagnostic disparities, is critical. We aimed to describe a variety of patient diagnostic journeys for equitable capturing of patient-reported experiences and outcomes; map diagnostically relevant patient-reported domains onto these journeys; and organize findings into a framework that presents specific timing and setting opportunities of assessment.

### Methods

We adapted an existing set of patient diagnostic error journeys from the National Quality Forum report and used human-centered design to illustrate additional journeys and identify opportunities for patient reporting on their experiences and outcomes throughout. We mapped previously identified diagnostically relevant patient-reported domains into the journeys exploring specifications of these domains on a measurement continuum from diagnostic error to diagnostic excellence. We relied on internal expert consultations and a five-session international expert convening.

### Results

We grouped patient journeys into seven pairs of diagnostic error and counterfactual excellence scenarios including journeys of those who are “invisible” to the health system. We organized the journeys into a taxonomy based on their timing, setting, and diagnostic care utilization. We specified the diagnostically relevant domains and aggregated them into a 21-domain map of patient-reported experiences and outcomes of diagnostic excellence. We identified up to four measurement opportunities throughout diagnostic journeys: pre-encounter, within-encounter, immediate post-encounter, and subsequent cross-sectional, agnostic of setting. We synthesized elements necessary to reflect the complexity of the continuum from diagnostic error to diagnostic excellence - taxonomy, domain map, and measurement opportunities - into a novel eight-part framework for patient-reported measurement of diagnostic excellence. (See depictions of parts I [measurement continuum and journey types], II [a journey type with mapped domains], and VIII [measurement opportunities by journey type]).

### Conclusion

The presented measurement framework anticipates needs and supports the development of a suite of patient-reported measures to assess the full range of diagnostic experiences and outcomes of patients at various settings and timings. Patient-reported assessments along diagnostic journeys bring unique value enabling diagnostic co-production and advancing diagnostic equity.

**Fig. 1 (abstract 203.4a):**
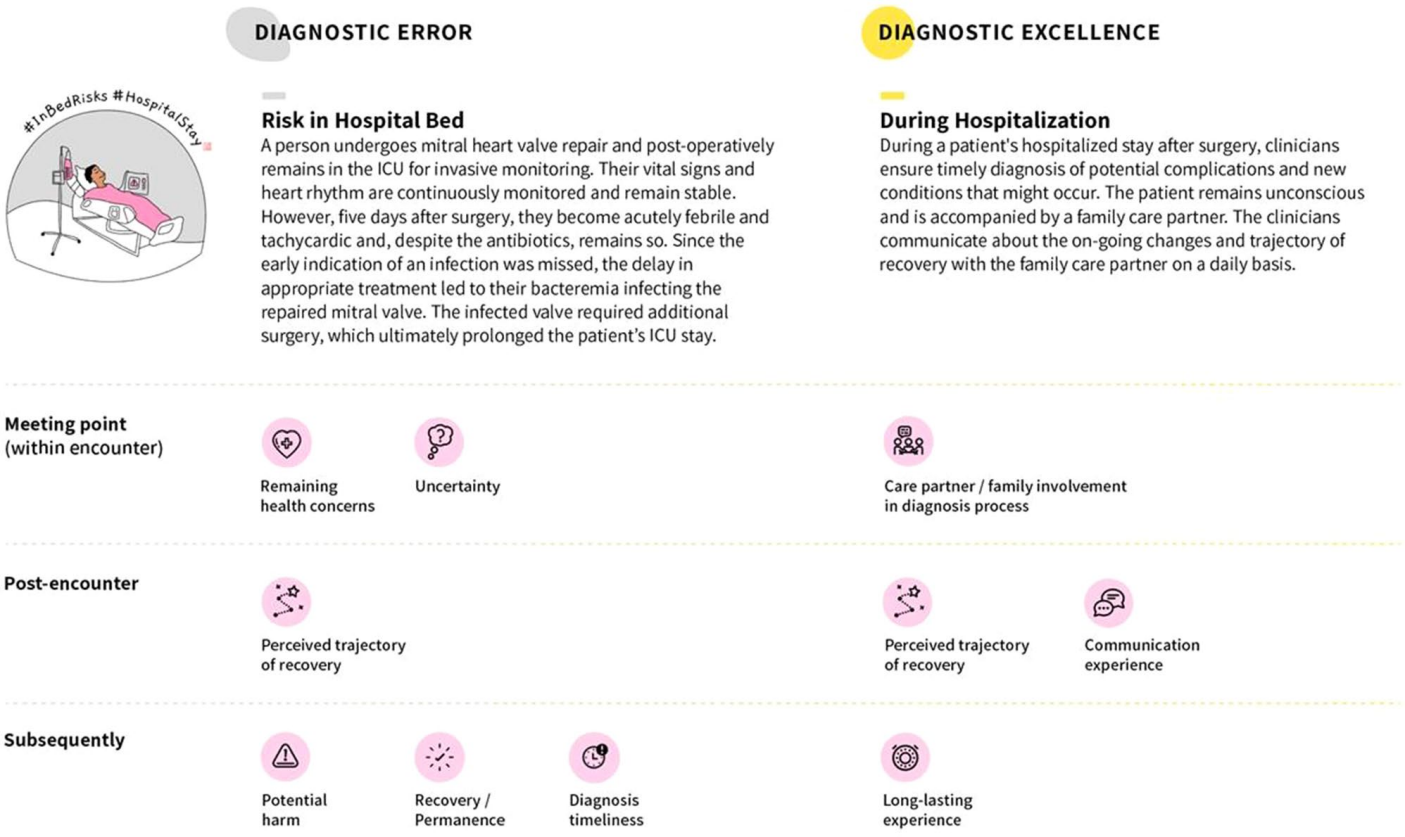
Framework for patient-reported measurement of diagnostic excellence

**Fig. 1 (abstract 203.4b):**
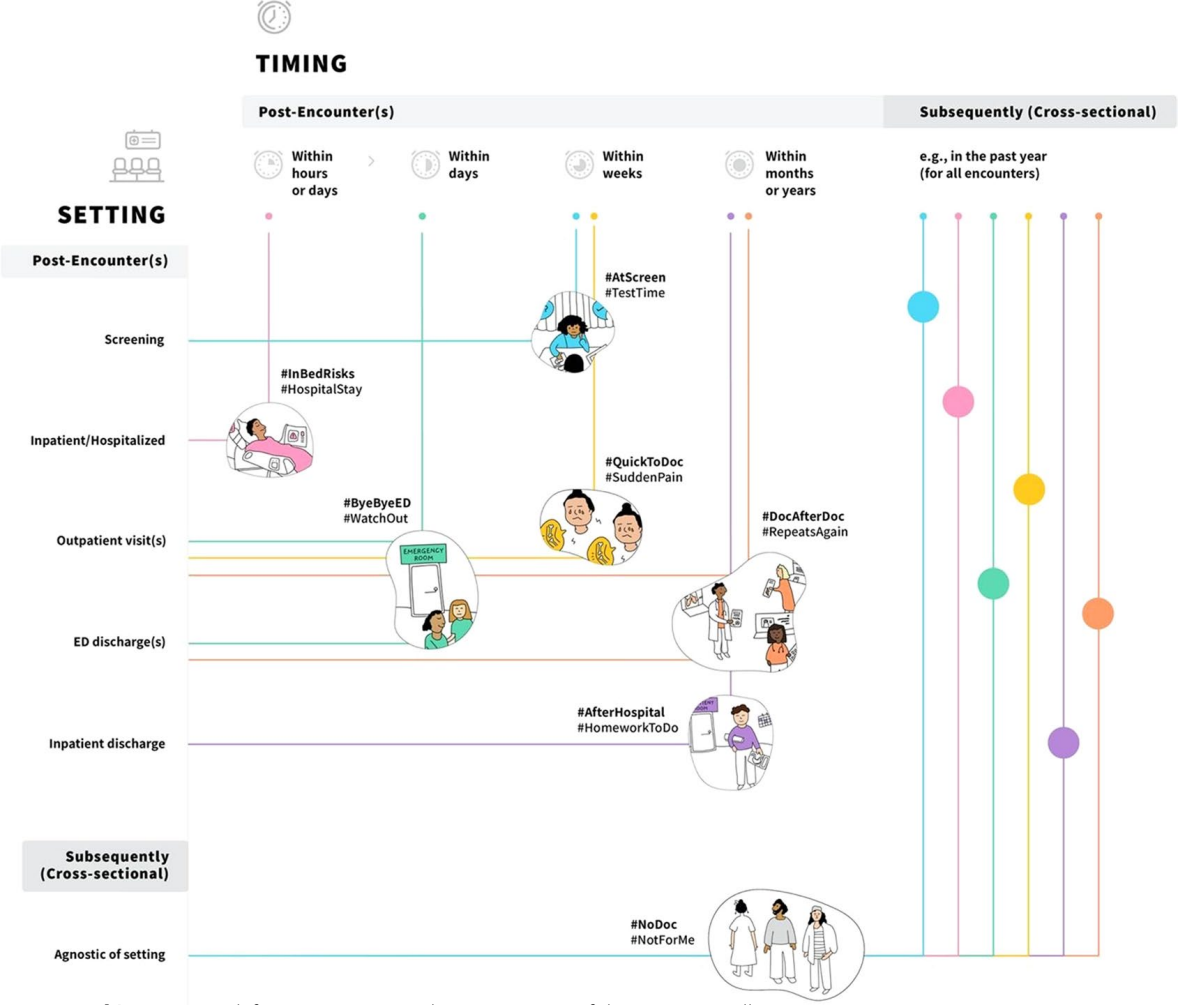
Framework for patient-reported measurement of diagnostic excellence

**Fig. 1 (abstract 203.4c):**
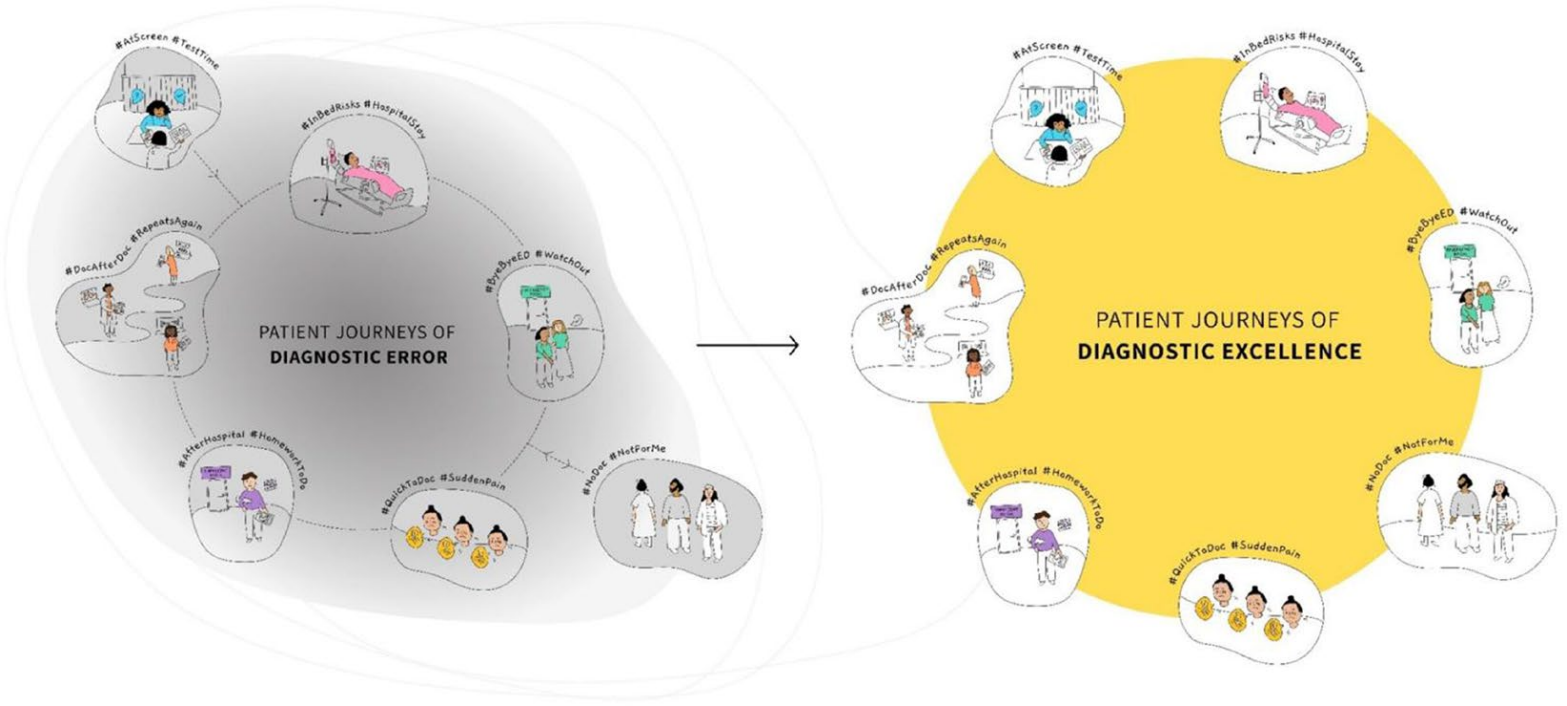
Framework for patient-reported measurement of diagnostic excellence

## 204.3 Development of a patient-reported outcome measure of postoperative recovery for lung cancer: a modular conceptual framework

Cheng Lei^1^, Hongfan Yu^2^, Qiuling Shi^2^

^1^Sichuan Cancer Hospital and Institute, Chengdu, China, ^2^State Key Laboratory of Ultrasound in Medicine and Engineering, Chongqing Medical University, Chongqing, China

*Journal of Patient-Reported Outcomes 2026*, **10(Suppl 1)**:204.3

### Aims

To address the unmet need for patient-centered outcome assessment in thoracic oncology, we launched a research program to develop a lung cancer surgery-specific patient-reported outcome measure (PROM). Guided by FDA PRO development guidelines, this first phase aimed to construct a modular conceptual framework delineating health domains critical to postoperative recovery through systematic evidence synthesis.

### Methods

This study was conducted in three phases: (1) Systematic review: To identify PROMs with measurement properties appraised in the context of recovery after lung cancer surgery, (2) ICF-based content mapping: Categorization of 378 identified items into International Classification of Functioning, Disability and Health (ICF) codes through independent dual coding; (3) Framework development: Iterative synthesis of ICF components into clinically meaningful modules using inductive method principles.

### Results

A total of 986 research articles were retrieved. 63 studies that met the criteria were included. The systematic review identified 18 PROMs covering 378 items. All items have been summarized, categorized, and associated with the content of different levels of the ICF. 3 Domains, 21 one-level classification items, and 60 two-level classification items of the ICF have been covered (Figure 1). Based on this content, we have developed a modular conceptual framework for postoperative recovery in lung cancer (Figure 2). This framework consists of four modules: Physiological Symptoms, Psychological Symptoms, Daily Life Functions, and Social Functions.

### Conclusion

This modular conceptual framework is an essential first step in our research program. The sound methodological approach used to derive this framework may be valuable for studies aimed to develop PROMs according to FDA standards.

**Fig. 1 (abstract 204.3):**
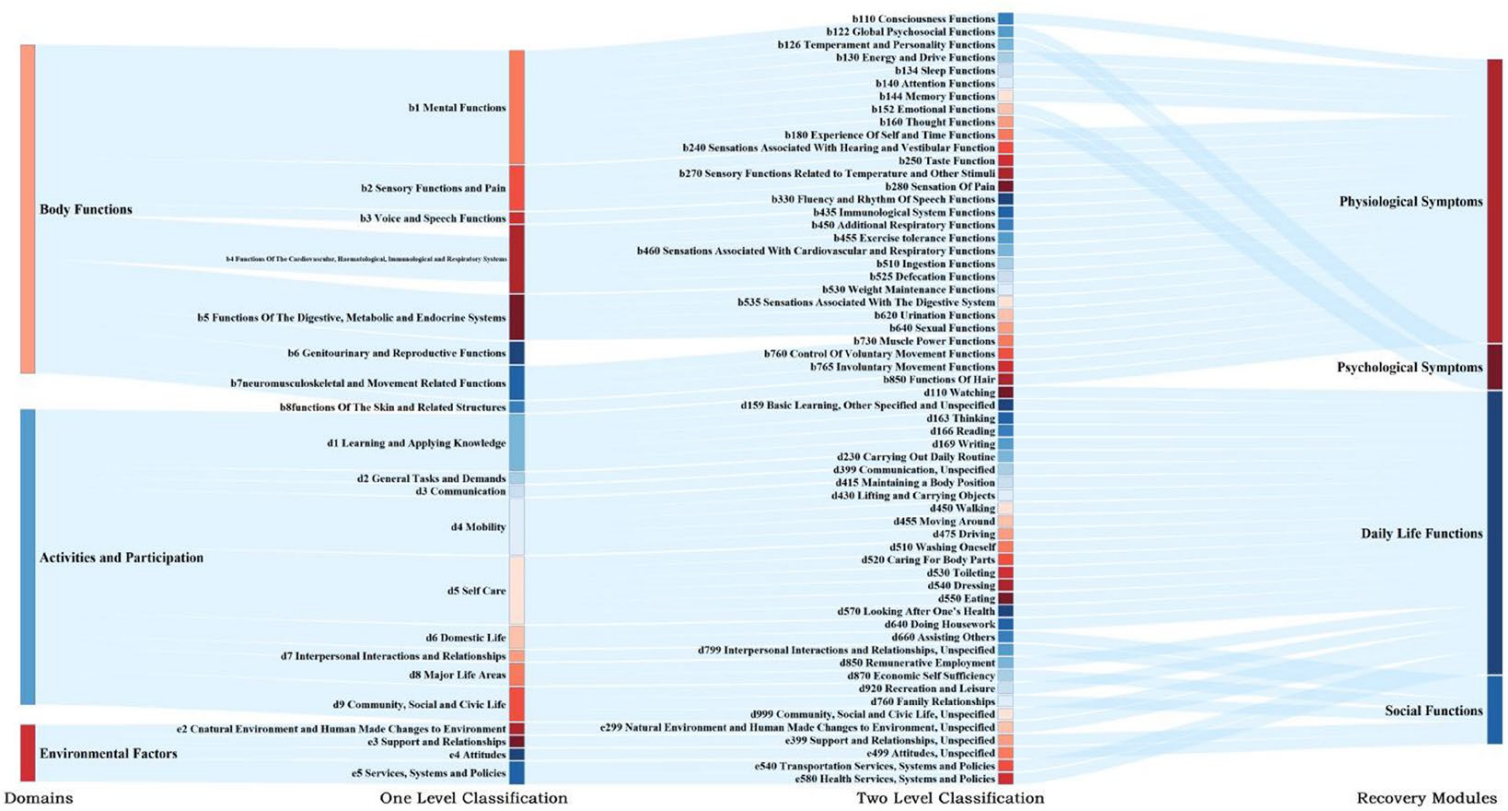
The sankey diagram of the conceptual framework formation process

**Fig. 2 (abstract 204.3):**
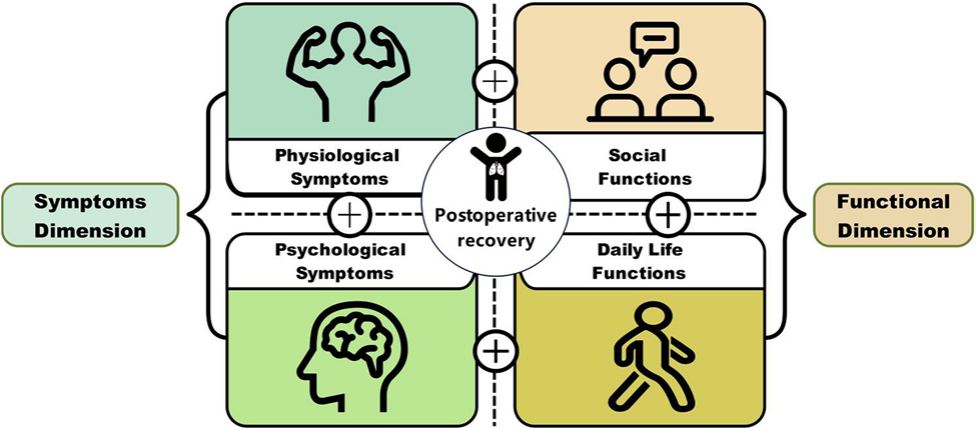
The conceptual framework of postoperative recovery for lung cancer

## 204.4 Time in Severe Symptom State With Eribulin Versus Paclitaxel in Patients With Locally Recurrent or Metastatic Breast Cancer (ACCRU RU011201I)

Gina Mazza^1^, Ethan Basch^2^, David W. Hillman^3^, Erik J. Asmus^3^, Minji K. Lee^3^, Lauren Rogak^1^, Minetta C. Liu^4^, Gita Thanarajasingam^3^, Amylou C. Dueck^1^

^1^Mayo Clinic, Scottsdale, Arizona, USA, ^2^Lineberger Comprehensive Cancer Center, Chapel Hill, North Carolina, USA, ^3^Mayo Clinic, Rochester, Minnesota, USA, ^4^ Natera, Inc., Austin, USA

*Journal of Patient-Reported Outcomes 2026*, **10(Suppl 1)**:204.4

### Aims

During treatment, patients may transition in and out of a state of severe symptom burden. We developed a Markov model to estimate patients’ time spent experiencing severe symptom burden, as well as the probability of transitioning between states. Unlike counting the amount of time patients report experiencing severe symptom burden, Markov modeling can account for non-informative missing data.

### Methods

We used data from a randomized phase III trial of eribulin compared to paclitaxel as first- or second-line therapy for patients with locally recurrent or metastatic breast cancer. We estimated a 3-state, continuous-time, first-order Markov model for patient-reported fatigue over the first 12 weeks, with treatment arm as a covariate. Transitions between the 3 states (i.e., not experiencing severe fatigue, experiencing severe fatigue, off study treatment) could occur at any time (Figure). Experiencing severe fatigue was defined by a PRO-CTCAE score ≥3. Discontinuing study treatment was an absorbing state, meaning patients remained in this state and could not transition to the other 2 states thereafter. The probability of worsening to a state of severe fatigue and the time spent experiencing severe fatigue over the 12-week period were estimated for each treatment arm.

### Results

The sample included 192 patients who completed at least one PRO-CTCAE questionnaire, with 98 and 94 patients in the eribulin and paclitaxel arms. 60% (59/98) and 55% (52/94) of patients in the eribulin and paclitaxel arms reported experiencing severe fatigue (Fisher’s exact test p=0.56). Based on estimates from the Markov model, the probability of transitioning from a state of non-severe fatigue to a state of severe fatigue was numerically higher in the paclitaxel arm (transition rate=0.18, 95% CI [0.14, 0.23]) than in the eribulin arm (transition rate=0.13, 95% CI [0.10, 0.17]). Patients in the paclitaxel arm spent more time in a state of severe fatigue than did patients in the eribulin arm over the 12-week period (2.12 versus 1.81 weeks).

### Conclusion

Markov modeling is an innovative approach for understanding patients’ probability of worsening or improving symptom burden, as well as their total time spent in a state of severe symptom burden in the presence of missing data.

**Fig. 1 (abstract 204.4):**
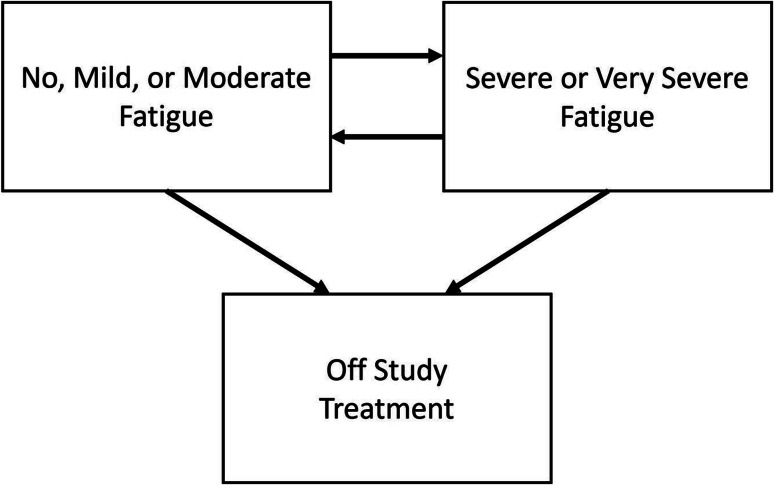
Time in Severe Symptom State With Eribulin Versus Paclitaxel in Patients With Locally Recurrent or Metastatic Breast Cancer (ACCRU RU011201I)

## 204.5 Identifying Longitudinal Trajectories of Quality of Life and Associated Risk and Protective Factors Among Cancer Patients

Jiwon Kim^1^, Maja Kuharic^1^, Nicola Lancki^1^, Kimberly Webster^1^, Karen Llave^1^, Shaili Ganatra^1^, David Cella^1^

^1^Northwestern University, Chicago, Illinois, USA

*Journal of Patient-Reported Outcomes 2026*, **10(Suppl 1)**:204.5

### Aims

The 7-item Functional Assessment of Cancer Therapy-General (FACT-G7) is a validated, brief measure of health-related quality of life (QoL) used in oncology settings. While many conceptualize QoL as a static trial endpoint, growing evidence underscores the value of understanding its dynamic nature over time to inform point-of-care interventions. Grounded in the Wilson and Cleary (1995) model, this study adopts a person-centered, longitudinal approach to (1) identify distinct trajectories of QoL over a 12-month period using FACT-G7, and (2) incorporate clinical, mental health, sociodemographic, and healthcare system related factors to uncover both risk and protective factors that shape patients’ QoL trajectories.

### Methods

FACT-G7 scores were measured at baseline, 3, 6, 9, and 12 months from a sample of 3,648 cancer patients (66% female, aged 19-92, M=60.91, SD=12.70) receiving care for a cancer diagnosis within a large academic medical center and enrolled in an ePRO implementation study. Growth mixture modeling was used to identify latent subgroups based on longitudinal QoL patterns. Model selection was guided by model fit indices of Akaike Information Criterion (AIC), Bayesian Information Criterion (BIC), and Lo-Mendell-Rubin likelihood ratio tests. To examine predictors of class membership, a multinomial logistic regression was conducted using baseline covariates.

### Results

A comparison of the model fit indices indicated that the three-class model provided optimal fit, revealing three distinct QoL trajectories: high QoL (40.8%, FACT-G7 scores 24-25), average QoL (39.5%, scores 18-19), and low QoL (19.7%, scores 12-13). Psychosocial factors - particularly loneliness (OR=1.62, 95% CI:1.30-2.01) and financial difficulties (OR=1.46, 95% CI:1.26-1.69) - strongly predicted membership in the low QoL trajectory compared to the average QoL trajectory. Clinical factors, including comorbidities (Charlson Comorbidity Index; CCI) and symptom burden severity (PRO-CTCAE), were associated with poorer QoL trajectories, with insomnia (OR=1.87, 95% CI:1.52-2.30) and nausea (OR=1.95, 95% CI:1.60-2.39) demonstrating the strongest negative effects. Conversely, higher satisfaction with cancer care (CAHPS) and cancer-related self-efficacy were protective factors associated with higher QoL trajectories.

### Conclusion

Nearly 20% of participants experienced persistently low QoL throughout the 12-month period. These findings identify modifiable risk and protective factors that can inform targeted early interventions to improve long-term QoL outcomes across the cancer care continuum.

## 204.1 Enhancing quality of life through policy-driven integration of clinical pharmacists in cancer pain management: Evidence from the PharmaCAP Trial in Nepal

Sunil Shrestha^1^, Simit Sapkota^1^, Siew Li Teoh^2^, Bhuvan Kc^2^, Vibhu Paudyal^3^, Shaun Wen Huey Lee^2^, Siew Hua Gan^2^

^1^Kathmandu Cancer Center, Bhaktapur, Nepal, ^2^Monash University Malaysia, Bandar Sunway, Malaysia, ^3^Kings College London, London, UK

*Journal of Patient-Reported Outcomes 2026*, **10(Suppl 1)**:204.1

### Aims

To assess the feasibility, acceptability, and preliminary impact of integrating clinical pharmacists into multidisciplinary teams (MDTs) for cancer pain management in low-resource settings. The study also aimed to explore the policy implications of such integration on improving health-related quality of life (HRQoL) among cancer patients.

### Methods

A multicenter, open-label, feasibility-pilot randomized controlled trial (PharmaCAP) was conducted in two oncology centers in Nepal. Adult cancer patients experiencing pain were randomized into either a control group receiving usual care or an intervention group receiving clinical pharmacist-led services. These services included comprehensive medication review, patient education, and adherence counseling over a 4-week period. Primary outcomes assessed feasibility indicators (recruitment, retention, satisfaction), while secondary outcomes included pain intensity, HRQoL (EORTC QLQ-C30), anxiety and depression (HADS), and medication adherence (MARS-5).

### Results

Out of 140 screened patients, 92 were enrolled and randomized, with 85 (93.4%) completing the study. The intervention was highly acceptable (100% approval of randomization), with high retention in both groups (PharmaCAP: 93.4%, control: 91.3%). The PharmaCAP group showed statistically significant improvements in global HRQoL (p<0.001), physical functioning (p=0.006), and reduced financial difficulties (p<0.001). Psychological outcomes also improved with lower anxiety and depression scores (p<0.001), alongside better medication adherence (p=0.004). Although both groups saw reductions in pain intensity, the between-group difference was not statistically significant. Patient satisfaction with the pharmacist-led intervention was high (93%).

### Conclusion

Integrating clinical pharmacists into MDTs is feasible and acceptable in low-resource oncology settings and leads to meaningful improvements in HRQoL, psychological well-being, and medication adherence among cancer patients. These findings highlight the need for supportive health policy to formally recognize clinical pharmacists as key contributors to cancer care, especially in resource-constrained systems.

## 204.2 ePRO Vision’s Latest PROM-AI apps to Accelerate PROM Clinical Implementations

Xu Frank Wang^1^, Xin Shelley Wang^2^, Qing Guo^1^

^1^ePRO Vision (Beijing) Health Technology Co., Ltd., Beijing, China, ^2^UT MD Anderson Cancer Center, Houston, Texas, USA

*Journal of Patient-Reported Outcomes 2026*, **10(Suppl 1)**:204.2

### Aims

In support of clinical research and clinical practice projects, ePRO Vision, a Chinese PRO-based health technology company, developed the Real-World Data Management Platform (RWDMP), and made an effort for further effectively using artificial intelligence (AI) on it. This pilot study tested efficiency of application services at different stages of screening, diagnosis, treatment and follow-up, which represents a progress of applying Large Language Model (LLM) technology represented by China’s Deepseek.

### Methods

For a localized deployment of Deepseek-based apps into ePRO clinical implementations with multi-source clinical data, we (1) used AI version of patient situation summary report on RWDMP. It calls the domestic clinical expert consensus on specific diseases or NCCN/CSCO treatment guidelines supported by Deepseek to review and analyze the uploaded patient’s date (such as PROs scores and CT results), and quickly provides a summary of recommended intervention and follow-up action point. (2) tested the AI app on evaluation, risk identification and early warning of adverse events (AE) during patient treatment. The grade of CTCAE (version 5.0) on hospital’s Electronic Medical Record (EMR) were gathered by RWDMP’s engine. The platform automatically storage, calculate and update the evaluation of AE data.

### Results

We have tested 78 patients so far. Based on the severity of PRO score, the time for preparing outpatient consultation and follow-up by defined pathway was reduced from more than 15 minutes to less than 1 minute. The time for preparing preoperative information was shortened to less than 1 minute. The results of the survey on the satisfaction of follow-up to the out-of-hospital management of patients are being counted for further report. The average time patient’s AE data collection on the platform was reduced to less than 5 seconds in average. There was 60% increase use of AI-based AE data collection by clinicians.

### Conclusion

LLM based AI accelerates scientific and technological making it easy and fast to view the content for individualized consultation and review AE assessment results on ePRO Vision’s platform. It’s better supports the application of symptom management based on ePRO clinical research- and practice, improves user experience and efficiency, and better promotes the clinical application of PROM implementation.

## 205.1 Evaluating the Association Between Insurance Type and Financial Toxicity in Patients with Cancer

Ada Lu^1^, Alireza Ebrahimi^1^, Jean Yi^1^, Nora B. Henrikson^2^, Laura Panattoni^1^, Diana Lowry^1^, Salene M. W. Jones^1^

^1^Fred Hutchinson Cancer Center, Seattle, Washington, USA, ^2^Kaiser Permanente Washington Health Research Institute, Seattle, Washington, USA

*Journal of Patient-Reported Outcomes 2026*, **10(Suppl 1)**:205.1

### Aims

Financial toxicity and insurance-related administrative burdens are among the consequences of cancer treatment, adversely impacting survivors’ psychological well-being and quality of life. Variations in insurance coverage, cost-sharing mechanisms, and administrative complexity may influence patients’ financial toxicity and access to care. This study aims to evaluate the association between insurance type at the time of diagnosis and the extent of financial toxicity and administrative burdens among cancer survivors.

### Methods

This cross-sectional survey was conducted on 459 cancer survivors between November 2022 to June 2023 through the Fred Hutchinson Cancer Center Survivorship program and online survey platform Prolific (Table 1). Financial toxicity was measured using previously validated questionnaires, assessing financial depression, anxiety, consequences, and coping. Participants were also asked about experiences with administrative burdens, including delays, denials, surprise bills, restricted medicine, and out-of-network care. Insurance coverage at the time of cancer diagnosis was categorized as employer- or school-issued (private, commercial), self-purchased, Medicare (public), Medicaid (public), other, or uninsured. Sequential linear regressions were performed on variables of financial difficulty, while logistic regressions were performed on variables of administrative burdens.

### Results

The most common insurance was employer- or school-issued insurance (50.8%), followed by Medicare (21.4%), Medicaid (8.5%), other insurance (7.7%), self-purchased insurance (7.0%), and no insurance (4.8%). Compared to employer/school insurance, self-purchased insurance was associated with heightened levels of financial depression (b=0.32), financial consequences (b=0.571), and financial coping (b=0.419; p’s < 0.05); Medicaid was associated with increased financial consequences (b= 0.404; p=0.047) (Table 2). Insurance type was not broadly a strong predictor of administrative burdens, except for increased out-of-network care in self-purchased plans (b=1.117, p < 0.05).

### Conclusion

In cancer survivors, self-purchased insurance was correlated with significantly higher financial toxicity, including financial depression, anxiety, and coping, while Medicaid was correlated greater financial consequences, all of which are known to negatively impact quality of life. Additionally, our study indicated that insurance type did not significantly affect patients’ experience of administrative burden. These findings underscore the need for policy interventions aimed at reducing financial toxicity to improve the overall quality of life for cancer survivors.

**Table 1 (abstract 205.1):**
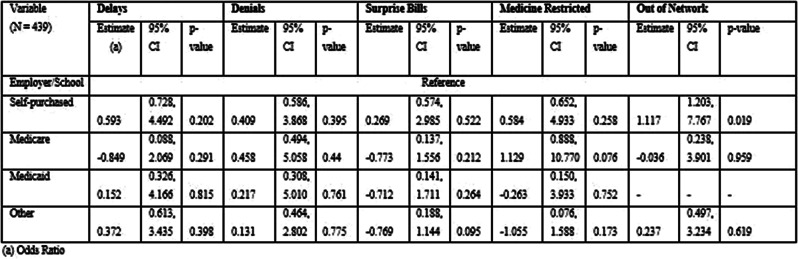
Patient demographics

**Table 2 (abstract 205.1):**
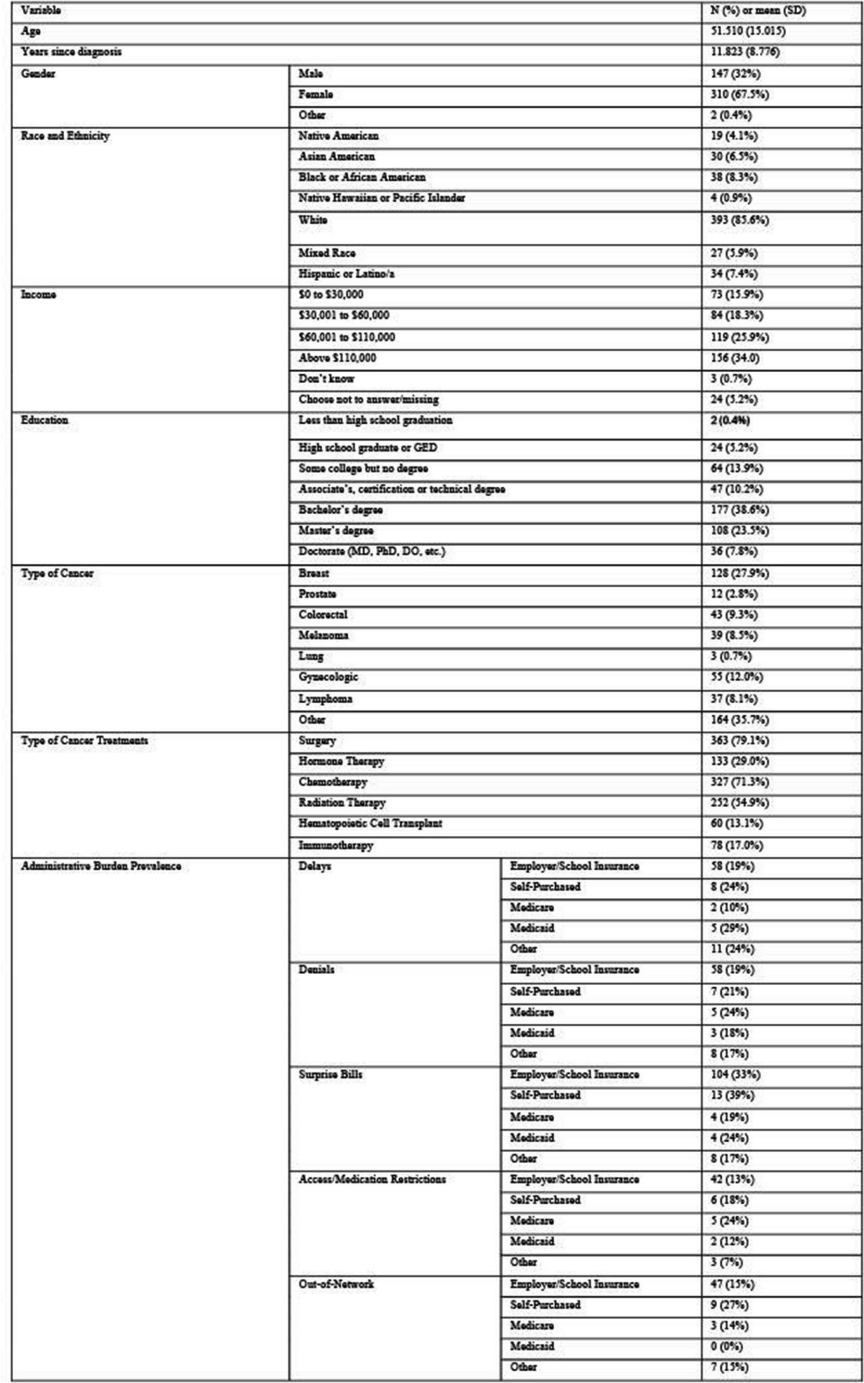
Adjusted Regression Analysis for the Association Between Insurance Type and Financial Hardship Dimensions

**Table 3 (abstract 205.1):**
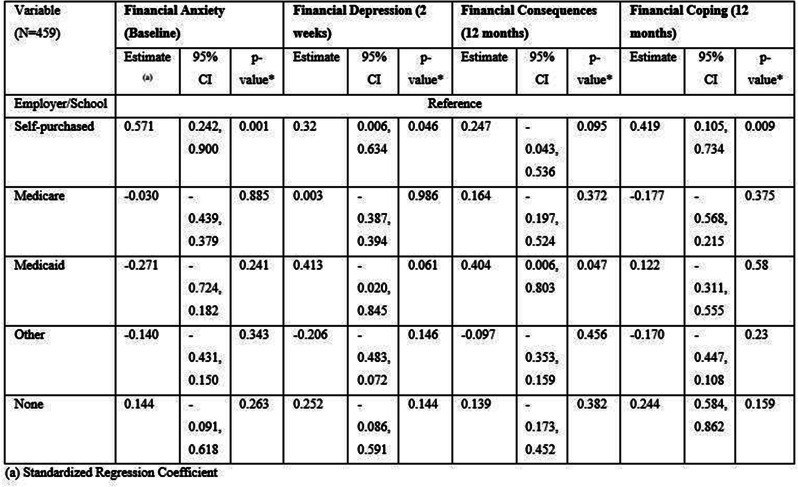
Adjusted Regression Analysis for the Association Between Insurance Type and Administrative Burdens

## 205.5 Evaluating missing data imputation strategies for PRO-CTCAE composite average

Allison Deal^1^, Brie Noble^2^, Gina Mazza^2^, Amylou Dueck^2^, Minji Lee^3^

^1^UNC Lineberger Comprehensive Cancer Center, Chapel Hill, North Carolina, USA, ^2^Department of Quantitative Health Sciences, Mayo Clinic, Scottsdale, Arizona, USA, ^3^Department of Quantitative Health Sciences, Mayo Clinic, Rochester, Minnesota, USA

*Journal of Patient-Reported Outcomes 2026*, **10(Suppl 1)**:205.5

### Aims

The PRO-CTCAE composite average, calculated as the mean of PRO-CTCAE composite scores, summarizes overall symptom and side effect burden. This study investigates the impact of imputation methods on score precision in the presence of missing composite scores.

### Methods

Simulations compared person mean imputation (PMI) and random forest imputation (RFI). Data were generated using the correlation matrix of composite scores from real-world data, then converted into ordinal scores (0–3) with zero inflation. The simulated sample was divided into complete and incomplete responders (50%:50%), with missing data introduced under three mechanisms: (1) missing completely at random (MCAR); (2) missing at random (MAR-Best), where patients answered items with the highest factor loadings for symptom burden from a prior CFA model; and (3) MAR-Worst, where patients answered items with the weakest loadings for symptom burden. 24 conditions, with 1,000 replicates, included 3 missing data mechanisms × 2 form lengths (8 or 16 composites) × 2 missing data rates (50%, 75%) × 2 imputation methods. Bias and root mean square error (RMSE) assessed accuracy and magnitude of imputation errors.

### Results

Mean bias (Figure 1) was zero using PMI and ranged from -0.01 to -0.22 for RFI. For RFI, higher rates of missingness (75%) doubled the mean bias. Bias was lowest with MCAR. MAR-Worst had higher bias than MAR-Best in shorter forms, but longer forms reduced this bias in RFI. For RMSE (Figure 2), mean estimates ranged from 0.23 to 0.55. RFI outperformed PMI, particularly at 75% missing, with longer forms yielding lower RMSE across methods. In shorter forms, MCAR excelled, followed by MAR-Best with RFI.

### Conclusion

PMI is recommended for the PRO-CTCAE composite average, with mean bias near zero across all scenarios, though RFI yields slightly lower RMSE than PMI. PMI or RFI works best when at least 50% of items are present, and longer forms are preferred to minimize both bias and RMSE.

**Fig. 1 (abstract 205.5):**
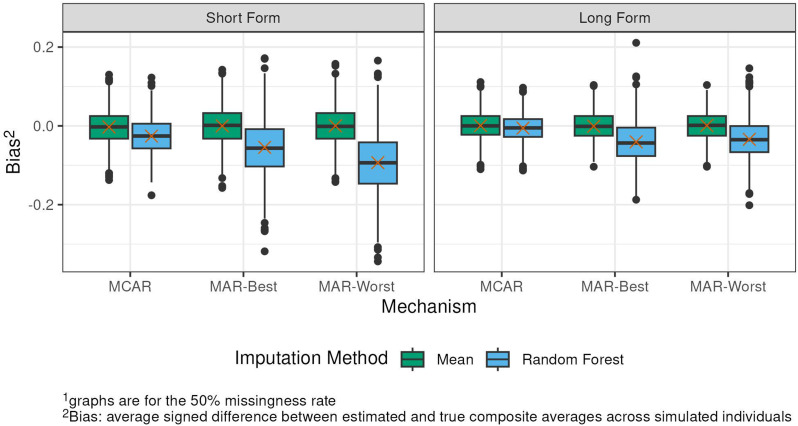
Distribution of Bias by Missing Data Mechanism^1^ and Imputation Method

**Fig. 2 (abstract 205.5):**
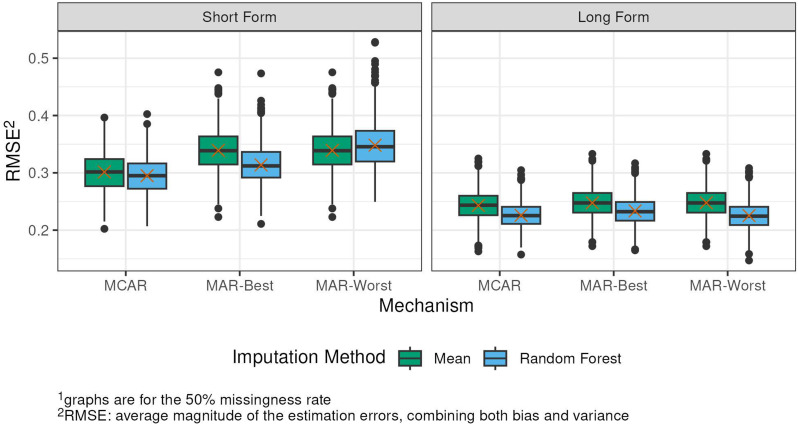
Distribution of RMSE by Missing Data Mechanism^1^ and Imputation Method

## 205.3 Needs and health-related quality of life domains relevant to people in Europe with advanced cancer in palliative care: A systematic review of qualitative research

Catalina Lizano-Barrantes^1^, Clara Amat‑fernandez^1^, Olatz Garin^1^, Ricardo Luer‑aguila^1^, Yolanda Pardo^1^, Leslye Rojas‑concha^2^, Melissa S.Y. Thong^3^, Giovanni Apolone^4^, Cinzia Brunelli^4^, Augusto Caraceni^5^, Norbert Couespel^6^, Nanne Bos^7^, Mogens Groenvold^8^, Stein Kaasa^9^, Gennaro Ciliberto^10^, Claudio Lombardo^11^, Ricardo Pietrobon^12^, Gabriella Pravettoni^13^, Aude Sirven^14^, Hugo Vachon^15^, Alexandra Gilbert^16^

^1^Hospital del Mar Research Institute, Barcelona, Spain, ^2^Copenhagen University Hospital – Bispebjerg, Copenhagen, Denmark, ^3^German Cancer Research Center (DKFZ), Heidelberg, Germany, ^4^Fondazione IRCCS Istituto Nazionale Dei Tumori-Milano, Milano, Italy, ^5^Dipartimento di Eccellenza 2023–2027 - Università Degli Studi Di Milano, Milano, Italy, ^6^European Cancer Organisation (ECO), Brussels, Belgium, ^7^Netherlands Institute for Health Services Research (Nivel), Utrecht, Netherlands, ^8^Department of Public Health and Bispebjerg/Frederiksberg Hospital, University of Copenhagen, Copenhagen, Denmark, ^9^Oslo Universitetssykehus HF, Oslo, Norway, ^10^IRCCS National Cancer Institute “Regina Elena” Rome, Rome, Italy, ^11^Organisation of European Cancer Institutes, Brussels, Belgium, ^12^SporeData OÜ, Tallinn, Estonia, ^13^Istituto Europeo Di Oncologia IRCCS, Milano, Italy, ^14^Unicancer, Paris, France, ^15^European Organisation for Research and Treatment of Cancer, Brussels, Belgium, ^16^Leeds Institute of Medical Research at St. James’s, University of Leeds, Leeds, UK

*Journal of Patient-Reported Outcomes 2026*, **10(Suppl 1)**:205.3

### Aims

The EU-funded project “Quality of Life in Oncology: measuring what matters to cancer patients and survivors in Europe” aims to co-design, validate and disseminate the EUonQoL-Kit, an electronic PROM with three versions (active treatment, survivors, and palliative care). We aimed to systematically review qualitative studies on outcomes, needs, experiences, preferences, concerns and quality of life of people in Europe with advanced cancer requiring palliative care over the last decade.

### Methods

Protocol registered (http://www.crd.york.ac.uk/PROSPERO, CRD42024575065). Search was performed in PubMed and Scopus, from 2013 onward. The inclusion criteria were studies employing qualitative methods, including mixed-method approaches, with samples from the 27 European Union (EU) countries, the United Kingdom (UK), and the 11 associated countries, that examined disease-related outcomes, needs, preferences, concerns, worries, or quality of life, in persons with advanced cancer in need of palliative care defined as: projected prognosis <12 months, or referred to a specialist palliative care team for receiving non-curative treatment for symptom control. Abstracts and full texts were reviewed, data extracted, and risk of bias assessed independently by two researchers. A thematic analysis stratified according to the study objective was performed to group into categories the emerging themes (primary outcome).

### Results

Of 18,256 articles identified, 20 fulfilled the inclusion criteria. Half of the studies had generic objectives on outcomes, concerns and quality of life in this population, and the other 10 focused specifically on: treatment, services and self-management; pain; and spiritual well-being Primary thematic analysis of over 35 themes and subthemes from the studies with generic objectives revealed that most themes aligned with Wilson and Cleary’s framework domains: ‘Psychological Function’ (n=15), ‘Symptoms and Physical Function’ (n=6), ‘Social Function’ (n=5), and ‘End-of-life’ (n=1). Notably, the ‘Clinical Management’ category, with 8 themes, emerged as a significant concern outside the framework domains.

### Conclusion

These findings emphasise the predominance of the psychological function domain in cancer patients requiring palliative care, including cancer-related anxiety and distress, coping mechanisms, control and decision-making, and fearing and expecting death. Additionally, clinical management unmet needs were identified in health care, information and communication, and end-of-life settings (home vs hospital).

## 205.2 Assessing the Understanding of ‘Quality of Life’ Amongst Global Oncology Patients

Hayley Simpson-Finch^1^, Shawn McKown^2^, Elan Josielewski^3^, Angie Lee^4^, Mark Kosinski^5^

^1^IQVIA, Reading UK, ^2^IQVIA, Hartford, Connecticut, USA, ^3^IQVIA, Roebling, New Jersey, USA, ^4^IQVIA, Seattle, Washington, USA, ^5^IQVIA, Providence, Rhode Island, USA

*Journal of Patient-Reported Outcomes 2026*, **10(Suppl 1)**:205.2

### Aims

‘Quality of Life’ is defined by the World Health Organisation (WHO) as ‘an individual’s perception of their position in life in the context of the culture and value systems in which they live and in relation to their goals, expectations, standard and concerns’[1]. Many clinical outcome assessments (COAs) ask patients to rate their ‘quality of life’, providing global data in a clinical study. However, the definition of ‘quality of life’ depends highly on the individual’s own culture and experiences. This study aims to better understand the global patient perception of the meaning of ‘quality of life’, taking into consideration socio-economic factors. [1] https://www.who.int/toolkits/whoqol

### Methods

IQVIA conducted linguistic validation of a new oncology questionnaire across diverse geographical and cultural locations, encompassing the main global language families. Data was gathered from in-person interviews with five oncology patients per country. Qualitative questioning was included to better understand patients’ interpretations of ‘quality of life’ in relation to their social reality. Additionally, cognitive debriefing was performed on overlapping physical functioning items from the SF-36 to determine if comprehension had changed since the original linguistic validation.

### Results

The perception and understanding of ‘quality of life’ varied based on factors such as cancer type, age, gender, country of residence, and economic and social status.

### Conclusion

This study highlights the diverse interpretations of ‘quality of life’ among oncology patients globally, influenced by a range of demographic and socio-economic factors. Understanding these variations is crucial for developing patient-centered care and effective communication strategies in oncology.

## 205.4 Quality of Life in Oncology: Psychometric properties of the EUonQoL-Kit static version

Olatz Garin^1^, Clara Amat^1^, Yolanda Pardo^1^, Cinzia Brunelli^2^, Morten Aagaard Petersen^3^, Àngels Pont^1^, Giovanni Apolone^2^, Augusto Caraceni^4^, Norbert Couespel^5^, Nanne Bos^6^, Mogens Groenvold^3^, Stein Kaasa^7^, Gennaro Ciliberto^8^, Claudio Lombardo^9^, Ricardo Pietrobon^10^, Gabriella Pravettoni^11^, Aude Sirven^12^, Hugo Vachon^13^, Galina Velikova^14^, Montse Ferrer^1^

^1^Hospital del Mar Research Institute, Barcelona, Spain, ^2^Fundazione IRCCS Istituto Nazionale Dei Tumori, Milan, Italy, ^3^University of Copenhagen, Copenhagen, Denmark, ^4^Università Degli Studi Di Milano, Milan, Italy, ^5^European Cancer Organisation (ECO), Brussels, Belgium, ^6^Netherlands Institute for Health Services Research (Nivel), Utrecht, The Netherlands, ^7^Oslo Universitetssykehus HF, Oslo, Norway, ^8^IRCCS National Cancer Institute “Regina Elena”, Rome, Italy, ^9^Oranisation of European Cancer Institutes, Brussels, Belgium, ^10^SporeData OÜ, Tallinn, Estonia, ^11^Instituto Europeo Di Oncologia IRCCS, Milan, Italy, ^12^Unicancer, Paris, France, ^13^European Organisation for Research and Treatment of Cancer, Brussels, Belgium, ^14^University of Leeds, Leeds, UK

*Journal of Patient-Reported Outcomes 2026*, **10(Suppl 1)**:205.4

### Aims

The EU-funded project “Quality of Life in Oncology: measuring what matters to cancer patients and survivors in Europe” aims to co-design, validate and disseminate the EUonQoL-Kit, an electronic PROM available in the languages of the EU27 member states and several associated countries. This communication describes the psychometric results of the 3 EUonQoL-Kit static versions respectively for patients in active treatment, survivors, and patients in need of palliative care.

### Methods

Observational study involving 44 clinical sites from 25 EU Member States and 7 associated countries (clinicalTrials.gov, NCT05947903, 2023-06-28). Participants filled out the EUonQoL-Kit as a static administration (n=4,139). In addition, two subsamples (each n=400) were randomly selected to complete the FACT-G and the EQ-5D-5L (validity subsample); or the EUonQoL-Kit at least one hour after the first completion (test-retest subsample). Construct validity was assessed by examining the patterns of EUonQoL-Kit scores across known groups. All analyses were stratified by the three target populations.

### Results

The EUonQoL-Kit versions are composed by 42–48 items; some are selected from IRT calibrated item banks, (Physical, Emotional, Cognitive, Role or Social Functioning, and symptoms), while others are CCT based items. For the latter, factor analyses have confirmed a 3 sub-domain structure: Disease Burden, Cancer-Related Worries, and Healthcare Experience, and an additional sub-domain only for survivors (Impact of Medical Appointments). All EUonQoL sub-domains showed Cronbach’s alpha ranging 0.7-0.9 in the 3 populations, with the exception of two sub-domains on survivors (0.63, 0.62). The majority of the previously hypothesized correlations between the EUonQoL-Kit and the FACT-G or EQ-5D-5L were confirmed. Regarding construct validity based on known groups, the higher Effect Sizes (ES) in the 3 populations were found between ECOG 0 and 4 (most ES>0.5). Differences by education level were confirmed in active treatment and survivors (ES >0.2 for 5 or more sub-domains). Differences according to the number of treatments were negligible.

### Conclusion

The static versions of the EUonQoL-Kit showed satisfactory psychometric properties (reliability and validity) in the three target groups. These results support the EUonQoL-Kit use as a standardised tool for the assessment of “quality of life in oncology” across Europe.

## 206.2

**Generative AI for qualitative analysis: speed at what cost?**

Anne Skalicky^1^, Carla Dias Barbosa^2^, Karen Bailey^2^, Sonya Stanczyk^3^, Meredith Smith^3^

^1^Evidera, Seattle, Washington, USA, ^2^Evidera, London, UK, ^3^Evidera, Wilmington, South Carolina, USA

*Journal of Patient-Reported Outcomes 2026*, **10(Suppl 1)**:206.2

### Aims

In the last decade, established Computer Assisted Qualitative Software (CAQDAS) programs have integrated machine learning and Artificial Intelligence (AI) into their tools, culminating in recent launches of generative AI capabilities, with the promise of speeding up qualitative coding analysis and reporting. Using these tools with qualitative data submitted to health regulatory bodies raises philosophical, ethical and operational questions, namely patient privacy and confidentiality and maintaining the standards of robust qualitative research.

### Methods

Acquiring informed consent prevents the use of patient generated data to test new AI tools; therefore, de novo qualitative data had to be generated for this pilot testing. One-on-one qualitative interviews were conducted with patient-centered research (PCR) scientists to explore their perspectives on the qualitative analysis and quality check (QC) processes that are the most time-consuming and prone to human errors. Interviews were conducted using semi-structured interview guide, recorded and transcribed using Microsoft Teams. The transcripts were analyzed following four approaches: 1) traditional analysis using human creation of codebook and conduct of coding and analysis; 2) ATLAS.ti automated coding; 3) ATLAS.ti automated coding with human checks; and 4) Atlas.ti intentional coding with the use of human input. The final outputs generated through these different approaches were compared.

### Results

Fifteen interviews were completed (n=5 with research associates, n=5 scientific project managers, n=5 principal investigators). Results highlight 1) the time and resources required for the completion of each approach 2) the balance of AI and human intelligence required to optimize outputs; and 3) patient data privacy concerns for implementation of AI in clinical outcome assessment (COA) research and potential solutions.

### Conclusion

ATLAS.ti AI use in qualitative data analysis, has the potential to speed up the process of conducting qualitative research, but requires the interaction of human input to ensure robust quality. Interview participants should be informed about the use of AI as part of informed consent, and analytic processes outlined in qualitative analysis plans. These approaches require further testing for more complex research questions such as assessment of meaningful treatment benefit or meaningful within-patient change for COAs. Future work will need to assess how regulatory and study sponsors view these approaches.

## 206.4

**Patient perspectives and experiences related to interviewing methodology for clinical outcomes assessment research**

Jake Macey^1^, Tamara Al-zubeidi^1^, Natalie Aldhouse^1^, Helen Kitchen^1^

^1^Clarivate, Clinical Outcome Assessment, London, UK

*Journal of Patient-Reported Outcomes 2026*, **10(Suppl 1)**:206.4

### Aims

Qualitative interviews are vital for obtaining in-depth understanding of the patient experience. Ideally, the design of qualitative studies would be patient-centric – involving active, meaningful patient and researcher collaboration – but access, timeline and funding restrictions all influence decision making. Thus, post-interview feedback was obtained to understand the qualitative study participation experience, including preferred interviewer profile and feelings about interview duration and administration method.

### Methods

From 2018–2024, participants were asked to optionally complete a brief feedback form after their remote (online/telephone) or in-person clinical outcome assessment concept elicitation and/or cognitive debriefing interview. Multiple-choice questions were analyzed descriptively and qualitative content analysis was applied to free text questions.

### Results

Feedback forms from 115 remote (n=68) and in-person (n=47) interviews lasting 90-minutes (n=77) or 30-75-minutes (n=38) with patients (n=98) and caregivers (n=17) from the US (n=80), Germany (n=13), Japan (n=11) and China (n=11) across various indications/diseases (e.g. infectious, digestive, congenital/hereditary) were analyzed. Both remote (90%) and in-person (87%) interview experiences were reported as appropriate. Interview duration was generally felt to be appropriate after interviews lasting ≤75-minutes (87%) and 90-minutes (78%). Participants would have been happy being interviewed by a researcher (42%), their doctor/nurse (22%), a patient/caregiver peer (18%), none of these (2%) and/or had no preference (16%). Participants would most like to be interviewed by a researcher (53%) over their doctor/nurse (8%), a patient/caregiver peer (8%) and/or had no preference (32%). Most (77%) participants reported being very likely to participate in other research interviews based on their experience. Participants who reported being somewhat likely (22%) or not likely (1%) to participate in other research interviews felt their interviews were too long/short (37%/4%) and/or would have preferred a different format (14% remote, 7% in-person and/or 4% written). Free text responses indicated participants experienced some trepidation before their interviews but had a positive experience overall and valued the professionalism/supportiveness of the study team and the opportunity to use their experience/insights to contribute to research.

### Conclusion

Although interviews conducted by a researcher and lasting ~60-minutes may be preferred, researchers can facilitate a positive interview experience for participants through approaching them professionally and sensitively, regardless of the interview administration method.

## 206.5 How low can you go? PROM developers’ perspectives on the involvement of young children in concept elicitation and cognitive interviews in quality of life research

Victoria Gale^1^, Jill Carlton^1^, Philip Powell^1^

^1^University of Sheffield, Sheffield, UK

*Journal of Patient-Reported Outcomes 2026*, **10(Suppl 1)**:206.5

### Aims

Entrenched recommendations suggest 8-years-old as a conventional cut-off for involving children in qualitative quality of life (QoL) research to inform patient reported outcome measure (PROM) development. This threshold is based on limited evidence, yet is rarely subject to scrutiny. The aim of this study was to audit the contemporary perspectives of PROM developers on the involvement of younger children in PROM development research.

### Methods

An online survey was developed to capture PROM developers’ perspectives. Participants were recruited from several sources (ISOQOL, the UK PROMs Network, and outcomes research groups in English-speaking countries) between August-November 2024. The survey captured developers’ background, experience with children, and perspectives on the minimum feasible age to involve children in concept elicitation and cognitive interviews for PROM development. Questions also asked about participants’ experience of involving children in PROM development. Data were analysed descriptively, with exploratory comparisons based on developers’ characteristics.

### Results

Fifty-eight responses were analysed. The majority were female (67%), had 5+ years’ experience developing PROMs (82.8%) and had experience working with children aged ≤ 11-years-old (77.6%). The mean minimum ages developers’ believed it feasible to involve children in concept elicitation and cognitive interviews were 6.66 years and 7.36 years, respectively. The mean minimum ages they reported including in practice were higher, at 7.67 years and 8.13 years, respectively. The most common reason for a lack of perceived feasibility at younger ages was limited ‘cognitive and/or linguistic skills’ and the least common was the research being ‘ethically inappropriate’. Participants with greater experience with younger children tended to give lower perceived feasibility ages. Those with experience of concept elicitation in children gave a higher perceived feasibility age for concept elicitation, while those with experience of cognitive interviews gave a lower age for cognitive interviews.

### Conclusion

PROM developers consider it feasible to involve children younger than 8-years-old in the qualitative development of PROMs in QoL research. Variation in views was linked to experience with children per se and prior qualitative research with children specifically. Future research should focus on establishing how children <8 years can best be included in PROM development.

## 206.3 Sentiment analysis of children’s open-text survey responses during the pandemic: Using PROMs to assess differences in affective states

Michiel Luijten^1^, Chris Gibbons^2^, Conrad Harrison^3^, Hedy van Oers^1^, Josjan Zijlmans^1^, Jacintha Tieskens^4^, Hekmat Alrouh^5^, Emma Broek^4^, Janna de Boer^6^, Lotte Haverman^1^, Tinca Polderman^1^

^1^Amsterdam UMC, Amsterdam, Netherlands, ^2^Oracle, Austin, Texas, USA, ^3^University of Oxford, Oxford, UK, ^4^LUMC Curium, Leiden, Netherlands, ^5^Vrije Universiteit, Amsterdam, Netherlands, ^6^University of Groningen, Groningen, Netherlands

*Journal of Patient-Reported Outcomes 2026*, **10(Suppl 1)**:206.3

### Aims

Sentiment analysis is a lexicon-based approach to assess the affective state of a sentence or piece of open text. It is applied to quickly evaluate the sentiment behind an open-text response by using a polarity score. This is a continuous score that ranges from -1 (negative) to +1 (positive)based on the relative amount of positive/negative words used in a sentence. In this study we apply sentiment analysis to an open-text question on the impact of the pandemic regulations on the lives of children.

### Methods

During the COVID-19 pandemic we asked children (age 8 to 18 years old) an open question regarding the impact of the pandemic regulations on their lives at seven different occasions. We calculated a polarity score for each response and provide mean polarity scores and percentages of positive (>0.03), negative (<-0.03) and neutral responses. Subsequently, we split the group into children with/without (sub)clinical anxiety scores (T-score > 50.6 on PROMIS Anxiety) and compared the mean polarity scores by independent T-test (total and per topic emerging from a structural topic model), where a Cohen’s D >0.2 was considered as at least a small, relevant effect.

### Results

In total there were 7604 responses from 3821 children. The sentiment analysis shows that overall children mainly had negative responses (polarity = -0.09, 49% of responses negative) to the pandemic regulations. Children with (sub)clinical anxiety scores were more negative on average (-0.12 vs -0.06, Cohen’s D = -0.14). From eight topics (adaptation/resilience, distress, social distancing measures, bonding, activities/boredom, future perspectives, homebound (school closures) and lack of celebratory events) differences between children with (sub)clinical anxiety and children without (sub)clinical anxiety were only found on the topics adaptation/resilience (-0.19 vs -0.10 polarity; Cohen’s D = -0.24, 59% vs. 49% negative responses) and distress due to lockdown (-0.06 vs 0.02 polarity; Cohen’s D = -0.20).

### Conclusion

Performing sentiment analysis allows us to quickly assess the affective states of respondents from open-text. In combination with PROMs it can be used to assess how outcomes are related to the affective states, which provides us with additional insights of possible relationships between children’s mental health and open-text responses.

## 206.1 Leveraging MAXQDA AI-Assist for Qualitative Workflows in Patient Experience Data

Trena Paulus^1^, Kristi Jackson^2^

^1^East Tennessee State University, Johnson City, Tennessee, USA, ^2^IQVIA, Golden, Colorado, USA

*Journal of Patient-Reported Outcomes 2026*, **10(Suppl 1)**:206.1

### Aims

Demonstrate the use of MAXQDA’s AI Assist features to enhance digital workflows for qualitative research in Patient Experience Data (PED), and address two questions: How can qualitative researchers confidently integrate AI-assisted tools without compromising analytic quality or transparency? How can we communicate this clearly to clients (and, subsequently, to regulatory bodies)? Rigor in the analysis of qualitative PED (such as Patient Reported Outcomes) must drive our understanding of concept elicitation, cognitive debriefing, and other forms of interview data. Patient-Focused Drug Development and other regulatory guidance do not provide this level of specificity for conducting our qualitative research, but many lessons can be learned from the use of AI in other fields that harness the power of qualitative data. By showcasing a concrete example from medical education in which our team developed an AI-Assisted workflow to analyze standardized patient interviews for how students conducted assessments for suicidal ideation, attendees will gain clear guidance and best practices for integrating MAXQDA AI-Assist effectively into qualitative analysis of PED.

### Methods

Using examples from our recent collaborative qualitative analysis of standardized patient interviews, we illustrate the practical integration of MAXQDA’s AI features within established digital qualitative workflows, guided by methodological principles previously published by the author. Methods include AI-assisted interview, document and coded data summarization tools; key topic identification; sub-code suggestions; and conversing with interview transcripts. We illustrate how an AI-Assist workflow can be used to establish coder consistency and guide iterative analytic discussions. Special emphasis is placed on how we used the workflow to include audit trail management, transparency, and replicability, in alignment with FDA regulatory guidelines.

### Results

Application of MAXQDA AI-Assist tools significantly streamlined the qualitative research workflow, while enhancing traceability and the strengths of the analysis. Team members reported increased confidence in analytic transparency and replicability.

### Conclusion

MAXQDA AI-Assist tools represent a robust innovation for qualitative research in the PRO and PFDD contexts. The integration of AI tools within structured digital qualitative workflows supports researchers in achieving higher efficiency, regulatory compliance, and methodological rigor, ultimately strengthening the role of qualitative data in PED.

## 207.1 Psychometric properties of Dutch-Flemish PROMIS Pediatric measures in the Dutch pediatric oncology population 8–17 years

Anne Westerweel^1^, Michiel A.J. Luijten^2^, Marte W. van der Wijk^1^, Martha A. Grootenhuis^1^, Caroline C. Terwee^3^, Lotte Haverman^2^, Kelly L.A. van Bindsbergen^1^

^1^Princess Máxima Center for Pediatric Oncology, Utrecht, The Netherlands, ^2^Amsterdam UMC Location University of Amsterdam, Emma Children’s Hospital, Child and Adolescent Psychiatry & Psychosocial Care, Amsterdam, The Netherlands, ^3^Amsterdam Public Health Research Institute, Methodology, Amsterdam, Netherlands

*Journal of Patient-Reported Outcomes 2026*, **10(Suppl 1)**:207.1

### Aims

Each year, approximately 600 children are diagnosed with cancer in the Netherlands, impacting their physical, mental, and social health. The Patient-Reported Outcomes Measurement Information System (PROMIS®) offers a promising approach to assessing these outcomes due to its strong psychometric properties and international applicability. This study evaluates the psychometric properties of PROMIS Pediatric measures in Dutch pediatric oncology patients aged 8–17 years to support their implementation.

### Methods

Pediatric oncology patients (n=100) completed PROMIS Pediatric item banks for Anxiety v3.0, Depressive Symptoms v3.0, Peer Relationships v3.0, Mobility v3.0, Fatigue v3.0, and Sleep Disturbances v1.0, as well as Global Health v1.0 scale, Anger 9a v3.0 short form, and Pain Intensity item v1.0. Additionally, they completed two PedsQL modules as legacy instrument. Measures were administered twice with a two-week interval. Construct validity was assessed by comparing PROMIS T-scores with PedsQL subscales. Reliability of item banks, extracted short forms, and simulated Computerized Adaptive Testing (CATs) was evaluated based on the number of participants with a standard error (SE) of measurement ≤0.32 (reliability ≥0.90). Efficiency ((1-SE(θ)2)/nitems) was calculated to compare measures’ performance relative to the number of administered items. Test-retest reliability was assessed using the Intraclass Correlation Coefficient (ICC>0.70 considered acceptable).

### Results

Preliminary analyses (first measurement n=50; second measurement n=28) confirmed construct validity, showing alignment between PROMIS measures and PedsQL subscales. Reliability was high around the sample mean and extended into clinically relevant ranges. PROMIS CATs were most efficient. Test-retest reliability was acceptable, with ICCs between 0.65 (Peer Relationships) and 0.90 (Fatigue). Mean T-scores ranged from 44.8 to 50.8 (SD=8.6–10.7).

### Conclusion

This study provides insights into psychometric properties of PROMIS Pediatric measures in the Dutch pediatric oncology population, supporting their implementation and improving patient-centered outcome assessments. At the time of the congress, data collection will be complete (n=100), allowing full evaluation of psychometric properties.

## 207.3 PROtocols matter: A systematised evaluation of adherence to Standard Protocol Items: Recommendations for Interventional Trials (SPIRIT) patient-reported outcome guidelines in interventional paediatric oncology trials

Denise Connolly^1^, Sarah Cohen-Gogo^1^, Sharon De Graves^2^

^1^The Hospital for Sick Children, Toronto, Ontario, Canada, ^2^University of Melbourne, Melbourne, Australia

*Journal of Patient-Reported Outcomes 2026*, **10(Suppl 1)**:207.3

### Aims

Despite increasing recognition of their importance, patient-reported outcomes (PROs) remain poorly implemented in interventional paediatric oncology trials. The Standard Protocol Items: Recommendations for Interventional Trials (SPIRIT) PRO guidelines outline the minimum protocol items required for trials that include PROs as primary or secondary outcomes. This work aimed to evaluate protocol adherence to the SPIRIT PRO guidelines and identify protocol-level gaps in PRO implementation in interventional paediatric oncology trials.

### Methods

A modified Preferred Reporting Items for Systematic Reviews and Meta-Analyses framework was employed, incorporating two adaptations: a novel search strategy on clinicaltrials.gov and use of the SPIRIT 2013 statement to appraise overall protocol completeness. Interventional paediatric oncology trials, registered on clinicaltrials.gov between January 2014 and May 2024, were identified. Trials were considered eligible if they included patients aged 0–18 years with solid or haematological malignancies, reported PROs as primary or secondary outcomes and had publicly available full-text protocols. Protocol adherence to the SPIRIT-PRO guidelines was assessed by a single reviewer using manifest content analysis. Each full-text protocol was assessed for the presence or absence of 16 SPIRIT PRO checklist items, comprising 42 sub-items. Items were only considered complete if all sub-items were present. The number of complete items per protocol was used to generate a ‘complete PRO score’. A ‘SPIRIT PRO percentage’ was calculated to reflect the proportion of SPIRIT sub-items present in the protocol. Descriptive statistics summarised these metrics.

### Results

Nine out of 245 identified trials were included. The mean ‘complete PRO score’ was 2 (range 0-3, Standard Deviation [SD] 1.1), out of a maximum score of 15-16, depending on the applicability of proxy-reporting items. The mean ‘SPIRIT PRO percentage’ was 25% (range 5-45%, SD 13.81). Figure 1 outlines the distribution of each SPIRIT PRO item and sub-item present, across all nine trials, highlighting missing protocol elements that are integral to effective PRO implementation.

### Conclusion

Low SPIRIT PRO adherence and widespread PRO-related protocol deficiencies were identified. Findings were limited by use of a single reviewer, restricted access to full-text protocols and variable protocol quality. This calls for more effective strategies to target protocol-based barriers to PRO implementation in interventional paediatric oncology trials.

**Fig. 1 (abstract 207.3):**
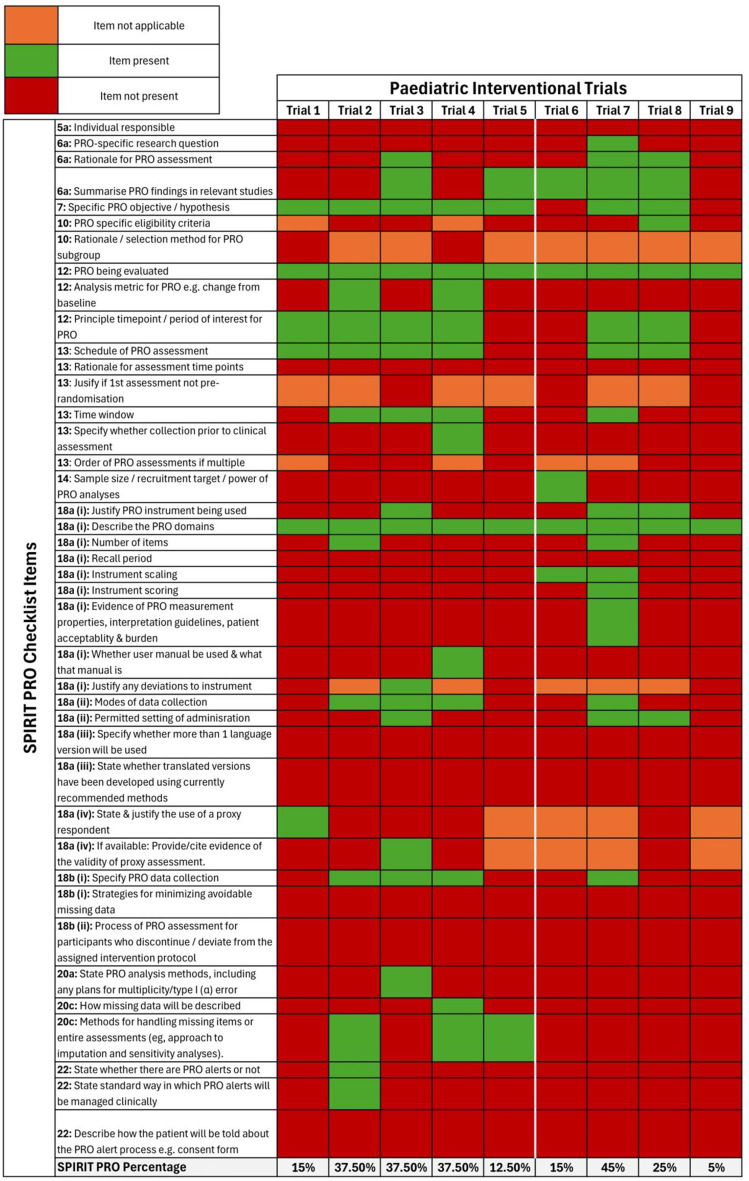
Distribution of SPIRIT PRO checklist items across nine paediatric trials (2014-2024). Green = present; orange = not applicable; red = absent. White line = time of SPIRIT PRO publication (2018)

## 207.2 Health-related quality-of-life among pediatric sibling hematopoietic stem cell donors; findings from RDSafe and DonorKids QL studies

Galen Switzer^1^, Jessica Bruce^1^, Bronwen Shaw^2^, Vidya Kuniyil^1^, James Varni^3^, Brandan Butler^4^, Connor Erickson^4^, Alisha Mussetter^4^, Allison Weiss^4^, Hisham Abdel-Azim^5^, Paibel Aguayo-Hiraldo^6^, Eric Anderson^7^, Victor Aquino^8^, Karlie Boone^9^, Joseph Chewning^10^, Ann Dahlberg^11^, Christopher Dvorak^12^, Jorge Galvez-Silva^13^, Ann Haight^14^, Jennifer Hoag^15^, Michelle Hudspeth^16^

^1^University of Pittsburgh, Pittsburgh, Pennsylvania, USA, ^2^Center for International Blood and Marrow Transplant Research (CIBMTR), Medical College of Wisconsin, Milwaukee, Wisconsin, USA, ^3^Colleges of Architecture and Medicine, Texas A&M University, College Station, Texas, USA, ^4^CIBMTR® (Center for International Blood and Marrow Transplant Research), NMDP, Minneapolis, Minnesota, USA, ^5^Division of Transplant and Cell Therapy/Hematological Malignancies, Departments of Pediatrics, Loma Linda University, Loma Linda, USA, ^6^Children’s Hospital Los Angeles, University of Southern California, Los Angeles, USA, ^7^University of California/Rady Children’s Hospital, San Diego, USA, ^8^UT Southwestern Medical Center Dallas, Dallas, USA, ^9^Pediatric Hematology/Oncology, University of Colorado, Aurora, USA, ^10^Pediatric Blood and Marrow Transplant Program, University of Alabama at Birmingham, Birmingham, USA, ^11^Clinical Research Division, Fred Hutchinson Cancer Research Center, Dept of Pediatrics, University of Washington, Seattle, USA, ^12^Division of Pediatric Allergy, Immunology & Bone Marrow Transplantation, University of California San Francisco, San Francisco, USA, ^13^Blood and Bone Marrow Transplant Program, Nicklaus Children’s Hospital, Miami, USA, ^14^Aflac Cancer and Blood Center, Emory University School of Medicine, Atlanta, USA, ^15^Department of Pediatrics, Medical College of Wisconsin, Milwaukee, USA, ^16^Pediatric Hematology/Oncology, Medical University of South Carolina, Charleston, USA

*Journal of Patient-Reported Outcomes 2026*, **10(Suppl 1)**:207.2

### Aims

Although the use of minors as HSC donors is medically and legally accepted there is a lack of understanding of HRQoL effects of pediatric HSC donation. Our goal is to describe HRQoL in two investigations and to identify factors associated with poor HRQoL in this group of otherwise healthy children.

### Methods

In RDSafe (2009-2014), researchers at the University of Pittsburgh interviewed 105 donors and parents at pre-donation, and 4-weeks and 1-year post-donation. A comparison group of matched healthy children was generated from existing data. HRQoL was assessed with the Pediatric Quality of Life Inventory (PedsQL). DonorKids QL (201802024) is the largest study of pediatric donors and their families to date and collected telephone interview data, including the PedsQL, from 673 total participants from 29 transplant centers pre-donation, and 4-weeks, 6-months, and 1-year post-donation.

### Results

RDSafe found that (1) across the three assessment time points, 21%, 19%, and 17% of donors had very poor HRQOL, (2) younger children were at greater risk of poor HRQoL, and (3) parents overestimated donor HRQoL by up to 10%. Pre-donation DonorKids QL found similar percentages of poor donor HRQoL – analysis of additional timepoints is pending. Additionally, DonorKids QL found donor characteristics associated with poorer pre-donation HRQoL that included depression (r = -.45 to -.78; p<.001) and anxiety (r = -.58 to -.69; p<.001), poorer donor-reported family cohesion (r = .54; p<.001), ambivalence about donation (r = -.37; p=.003), perceived pressure to donate (r = -.24; p=.006), and poorer understanding of donation (r = .30; p<.001). Other family characteristics associated with poorer donor HRQoL included poorer recipient HRQoL, lower parent education, parent depression and stress, and lower family cohesion. Key demographics including gender, race, income, employment status and partnership status were not associated with donor HRQoL.

### Conclusion

Combined, these investigations represent the most comprehensive investigation of HRQoL among pediatric HSC donors. They demonstrate a pattern of poor HRQoL among a subset of donors. In addition, they provide evidence that donor and family HRQoL and other characteristics are closely interrelated and suggest that interventions to mitigate HRQoL risks during donation/transplantation should involve the full family unit.

## 207.4 Development and Validation of the Observer-Reported Outcome for Children with Cancer (ObsRO-CC)

Rui Zhou^1^, Bei-Jia Wang^1^, Jing Hu^1^, Feng-Yang Jiang^1^, Wen Wang^1^, Yu-Yuan Zhong^1^, Hua-Yu Li^1^, Xin-Hui Xu^1^, Ren-Jun Gu^1^, Yi-Qing Weng^1^, Hong-Mei Wang^1^

^1^Department of Social Medicine, School of Public Health, Zhejiang University School of Medicine, Hangzhou, China

*Journal of Patient-Reported Outcomes 2026*, **10(Suppl 1)**:207.4

### Aims

As the survival rate of childhood cancer increases, the need to monitor quality of life becomes increasingly important. Given the severity of illness and immature cognitive function, observer-reported outcome (ObsRO) plays an important role in reporting health information of childhood cancer patients. This research aimed to develop a disease-specific observer-reported outcome instrument for Chinese children with cancer (ObsRO-CC) and to preliminarily test its psychometric properties.

### Methods

Items were elicited from qualitative interviews with parents of children with cancer. The content of items and the format of the initial instrument were refined through two rounds of cognitive interviews. Item reduction was conducted using classical test theory (CTT) and item response theory (IRT). Psychometric properties of the resulted final instrument were assessed in terms of internal consistency, test-retest reliability, structure validity, known-groups validity, and criterion validity.

### Results

A pool of 52 items were generated based on qualitative interviews. Item reduction using CTT and IRT in sequence resulted in a 25-item ObsRO-CC instrument containing five dimensions: somatic symptoms, psychological distress, disease perception, social adaptation, and treatment compliance. The Cronbach’s α coefficient and composite reliability (CR) of each dimension all exceeded 0.70. Most dimensions exhibited acceptable test-retest reliability except for the somatic symptoms dimension (ICC=0.68). The final ObsRO-CC instrument was confirmed to be of good structure validity by confirmatory factor analysis (χ2/df=2.71, CFI=0.94, TLI=0.93, RMSEA=0.06, SRMR=0.05). Known-groups validity was demonstrated with significant differences on scores of the ObsRO-CC being identified between demographic and clinical subgroups. Criterion validity was good with higher correlations between more comparable dimensions of ObsRO-CC and PedsQLTM 4.0 generic core module, PedsQLTM 3.0 cancer module.

### Conclusion

ObsRO-CC demonstrated robust reliability and validity, suggesting that it is a proper tool to assess the health outcomes of Chinese children with cancer. Additional research should be conducted to examine other properties including responsiveness and interpretability.

## 207.5 Family members/partners of people with autism experience a major impact on quality of life as measured by the Family-Reported Outcome Measure (FROM-16)

Rubina Shah^1^, Andrew Y. Finlay^1^, Faraz M. Ali^1^, Kennedy Otwombe^2^, Stuart J. Nixon^3^, Marie-Elaine Nixon^3^, John R. Ingram^1^, Sam Salek^4^

^1^Division of Infection and Immunity, School of Medicine, Cardiff University, Cardiff, UK, ^2^Statistics and Data Management Centre, Perinatal HIV Research Unit, Chris Hani Baragwanath Academic Hospital, University of the Witwatersrand, South Africa, Johannesburg, South Africa, ^3^Family Members Research Partner, Cardiff, UK, ^4^School of Life & Medical Sciences, University of Hertfordshire, Hatfield, UK

*Journal of Patient-Reported Outcomes 2026*, **10(Suppl 1)**:207.5

### Aims

The quality of life (QoL) of family members/partners living with and caring for people with autism is greatly affected, yet this impact is often ignored or neglected. Measuring this impact is critical to planning and providing appropriate support services to impacted families. This study aims to measure the impact of a person’s autism on the QoL of their family members using the generic and extensively validated Family Reported Outcome Measure (FROM-16).

### Methods

A UK cross-sectional online study recruited family members/partners of people with autism through the Autism Research Centre Cambridge database and Healthwise Wales. The family members/partners of people with autism completed FROM-16, and basic demographic details about themselves and their relative with autism. The data analysis included descriptive and other statistics, including non-parametric Mann-Whitney U-test and Kruskal-Wallis tests for group comparisons. Multiple linear regression was used to investigate relationships between dependent and independent variables. FROM-16 descriptive score banding was used to describe the severity of the impact of a person’s autism on family members/partners.

### Results

A total of 129 family members/partners (mean age=51.2 years, SD=12.8; females:n=100, 77.5%) of people with autism (mean age=28.1, SD=17.3; females: n=48, 37.2%) completed the FROM-16. The FROM-16 mean total score was 18.5 (SD=8.6), meaning “a very large effect” on the QoL of family members, with being a female and being a parent both significant predictors of impact. Feeling worried had the highest mean score of 1.55 (max=2), followed by ‘feeling frustrated’ (mean=1.45, max=2), ‘family activities’ (mean=1.40, SD=0.7) and ‘difficulty caring’ (mean=1.31, SD=0.7). The majority (60.5%) had a mean FROM-16 score ≥17, indicating “a very large effect” to “extremely large effect” on the QoL of these family members.

### Conclusion

The QoL of family members/partners of people with autism is impacted profoundly, underscoring the need for comprehensive family-centred care to provide needed support to families and empower them with skills to help their autistic family members live fuller lives. The FROM-16 could be used routinely to inform the assessment of the needs of family members and partners.

## 208.2 Refining the Spanish version of the awareness and beliefs about cancer (ABC) instrument: Supporting valid assessment for cancer knowledge and screening interventions

Christopher Amissah^1^, Jennifer Contreras^1^, Ester Villalonga Olives^1^

^1^University of Maryland School of Pharmacy, Baltimore, Maryland, USA

*Journal of Patient-Reported Outcomes 2026*, **10(Suppl 1)**:208.2

### Aims

Limited cancer knowledge hinders informed decision-making about prevention, screening, and treatment—especially among Hispanics with limited English proficiency. A previous study culturally adapted the Awareness and Beliefs about Cancer (ABC) instrument into Spanish for Spanish-speaking populations in the U.S., but several subscales underperformed. This study aims to improve the Spanish-language ABC by refining three underperforming subscales: Anticipated Delay in Seeking Care, Cancer Beliefs, and Cancer Screening Beliefs.

### Methods

We used item response theory (IRT) methods to analyze data on the ABC instrument from 726 Hispanic adults in the U.S., including 516 females and 210 males, aged 18 to 93 years. We excluded five items from the Anticipated Delay in Seeking Care subscale due to violations of IRT assumption of local item independence. We also reorganized the response options to reflect a more logical progression along the underlying latent construct. For the Cancer Beliefs and Cancer Screening Beliefs subscales, we reverse-scored negatively worded items to ensure consistency in item direction and improve score interpretation.

### Results

Our analysis showed high reliability for the Anticipated Delay in Seeking Care subscale (α = 0.83), but low reliability for the Cancer Beliefs subscale (α = 0.45) and the Cancer Screening Beliefs subscale (α = 0.60), indicating that the items may not cohesively represent their intended constructs. There was a narrow range of item difficulties for the Anticipated Delay in Seeking Care subscale, and negative item discrimination indices for some items in the Cancer Beliefs and Cancer Screening Beliefs subscales (Figure 1). Some response options of the Anticipated Delay in Seeking Care subscale did not function as expected (Figure 2).

### Conclusion

The low reliability and negative item discrimination indices for the Cancer Beliefs and Cancer Screening Beliefs subscales indicate suboptimal psychometric performance. To improve the validity of the instrument for use in cancer screening and prevention research, we are revising these subscales. Revisions include rewording items for clarity, developing new items to strengthen content validity, assessing the impact of negatively worded items, and re-evaluating the subscales’ dimensional structure. Our goal is to produce a psychometrically sound, culturally appropriate instrument that reliably supports efforts to advance cancer knowledge, screening, and prevention.

**Fig 1 (abstract 208.2):**
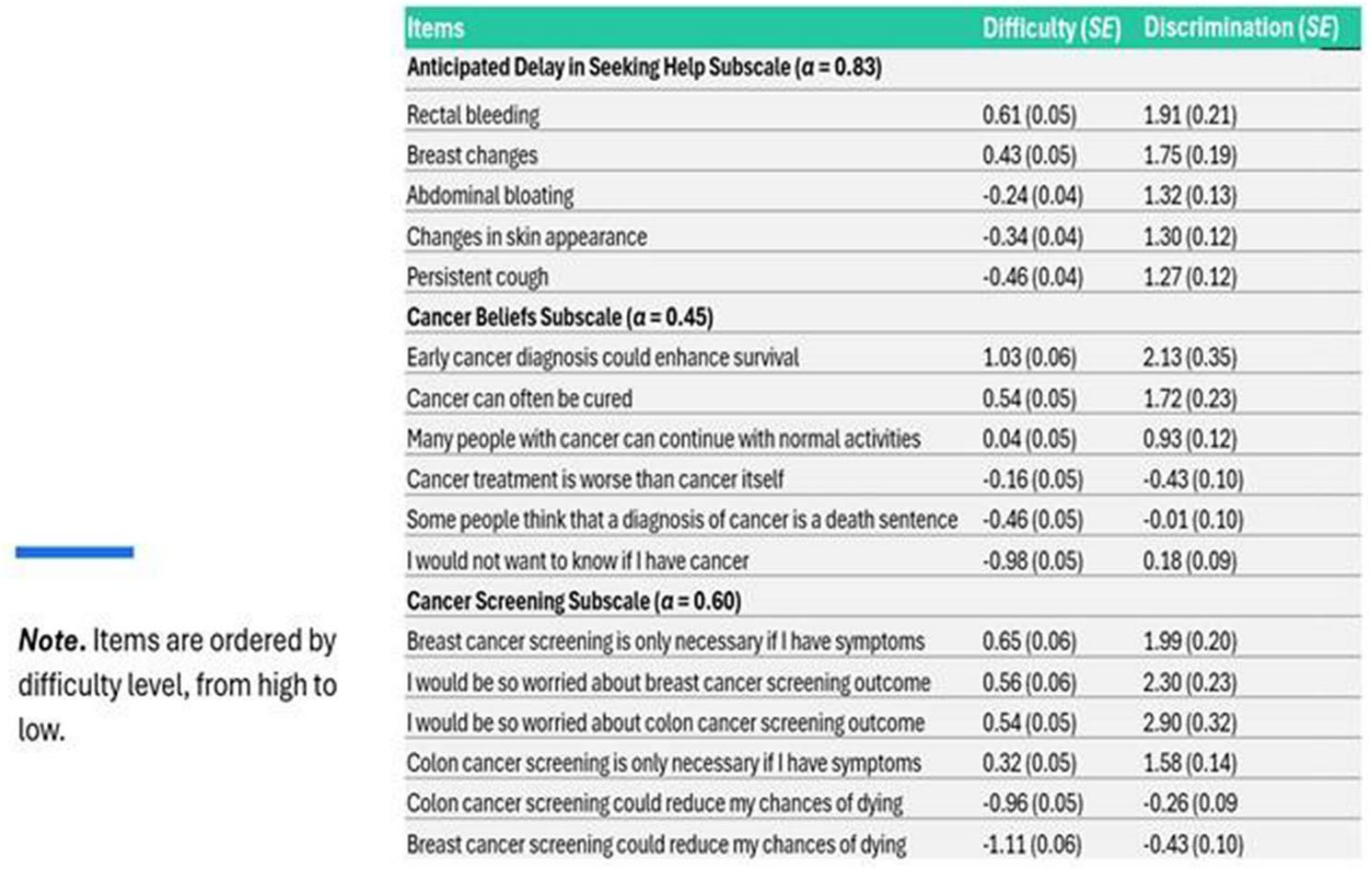
Item difficulty and discrimination of ABC Instrument-Hispanic version

**Fig. 2 (abstract 208.2):**
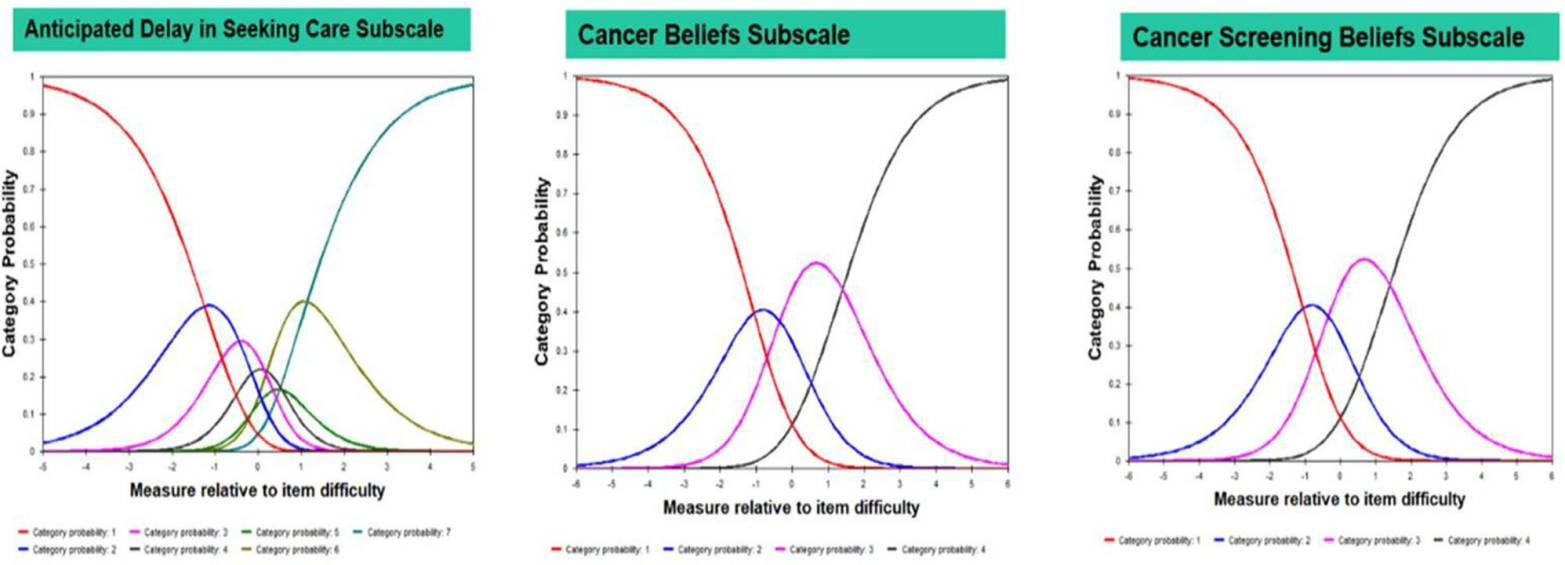
Rating scale functioning of ABC Instrument - Hispanic version

## 208.5 Assessing the Quality of AI-Generated Swahili Translations of the BREAST-Q: Enhancing PROM Accessibility in Low-Resource Settings

Sarah Nyakiongora^1^, Oluchukwu Dele-Oni^2^, Matteo Laspro^3^, Jinjie Liu^4^, Manraj Kaur^5^, Andrea Pusic^6^

^1^Division of Plastic and Reconstructive Surgery, Kenyatta National Hospital, University of Nairobi, Nairobi, New Hampshire, Kenya, ^2^Geisel School of Medicine at Dartmouth, Hanover, New Hampshire, USA, ^3^Division of Plastic Surgery, Department of Surgery, USC Keck School of Medicine, Los Angeles, California, USA, ^4^Harvard University, Boston, Massachusetts, USA, ^5^Brigham and Women’s Hospital, Department of Surgery, Boston, Massachusetts, USA, ^6^Brigham and Women’s Hospital, Division of Plastic and Reconstructive Surgery; Harvard School of Medicine, Boston, Massachusetts, USA

*Journal of Patient-Reported Outcomes 2026*, **10(Suppl 1)**:208.5

### Aims

Artificial intelligence (AI) shows promise in facilitating the translation of patient-reported outcome measures (PROMs) for underserved populations, though challenges in capturing linguistic and cultural nuances persist. The BREAST-Q, a PROM widely used to assess patient satisfaction post-breast surgery, has been translated into over 45 languages but lacks a Swahili version. With Swahili spoken by 16 million as a first language and up to 100 million as a lingua franca across East Africa, there is a need for this translation. Given the time and cost constraints of human translation, AI-driven solutions may offer a viable alternative. This study compares the quality of Swahili translations of the BREAST-Q Mastectomy module produced by human translators and four AI models: ChatGPT-4o, Google Gemini, Claude, and Google Translate. The primary objective is to assess translation quality using the METEOR metric, a widely used machine translation evaluation metric that assesses precision, recall, and synonym matching, with a secondary aim of determining which AI model performs most accurately.

### Methods

Forward and back translations of eight BREAST-Q Mastectomy scales were obtained using each AI model. Human translations followed a standardized process aligned with the International Society for Pharmacoeconomics and Outcomes Research (ISPOR) guidelines for translation and cultural adaptation. Back translation quality was evaluated using METEOR scores, with the original BREAST-Q Mastectomy scales serving as reference text. We applied the errors-in-variables regression (EIV) with bootstrapping procedure to assess the quality of each AI-generated translation by comparing it against human translation. This statistical method corrects estimation bias in regression models with error-prone METEOR scores and produces cluster-robust and heteroskedasticity-robust standard errors.

### Results

Preliminary findings indicate significant variability in AI-generated translations, with encoder-decoder models (Google Translate, Google Gemini) demonstrating higher METEOR scores compared to decoder-only models (ChatGPT-4o, Claude). As anticipated, human translation remains the gold standard. Final analysis will assess whether AI-generated translations meet clinical standards for PROM implementation.

### Conclusion

AI translation tools present a cost-effective, scalable alternative to traditional methods, particularly in low-resource settings. However, current limitations highlight the need for continued evaluation and refinement to improve AI translation reliability, advancing equitable access to quality healthcare across diverse linguistic populations.

## 208.1 Cross-cultural validation of the Dermatology Life Quality Index (DLQI) across 13 European languages

Sam Salek^1^, Jeffrey Johns^2^, Faraz M. Ali^3^, Florence Dalgard^4^, Jörg Kupfer^5^, Andrew Y. Finlay^3^

^1^University of Hertfordshire, Hatfield, UK, ^2^Division of Infection and Immunity, School of Medicine, Cardiff University, Cardiff, UK, ^3^Cardiff University, Cardiff, UK, ^4^Skåne University Hospital, Giessen, Norway, ^5^Dipl-Psych, Justus Liebig University, Giessen, Germany

*Journal of Patient-Reported Outcomes 2026*, **10(Suppl 1)**:208.1

### Aims

The Dermatology Life Quality Index (DLQI) is the most widely used tool to measure burden of skin diseases and assess intervention effectiveness. It is embedded in national guidelines and disease registries in >45 countries and available in >138 translations. The study aim was to examine the psychometric properties of the DLQI across 13 European languages.

### Methods

Data were analysed from a cross-sectional study conducted in 13 European languages where consecutive adult out-patients were recruited from 250 dermatology clinics. Parallel analysis (scree plots) and confirmatory factor analysis (CFA) were performed across datasets of each country. Known group analysis was performed of the DLQI sum score against physician assessed severity, and EQ-5D VAS score quartiles by country using the Jonckheere-Terpstra test.

### Results

From 3,635 patients, 3,408 patients completed the DLQI questionnaire with no missing data. 55.8% of patients were female and mean age was 46.6 years (SD=17.8). In every country, slightly more females were recruited than males. The commonest conditions reported were psoriasis (17.4%), non-melanoma skin cancer (10.8%), infection of the skin (6.7%), hand eczema (6.2%), acne (6.2%), nevi (5.0%), atopic dermatitis (4.5%), benign skin tumors (4.2%), and eczema (contact dermatitis) (4.1%). Spearman’s correlations between DLQI and other measures were EQ-5D VAS -0.409, disease severity 0.407, EQ-5D mobility 0.211, selfcare 0.257, activity 0.370, pain 0.409, and anxiety and depression 0.365 (all p<0.001), indicating that all these variables were suitable as anchors. All countries’ DLQI data showed unidimensionality from parallel analysis and very good fits to the 1-factor CFA model with CFI, TLI and NNFI values all >0.97 (COSMIN criteria). Cronbach alpha reliability was excellent (>0.9) in 6 countries and good (>0.8) in 7 countries. Known-group analysis of DLQI versus disease severity and versus EQ-5D VAS quartiles were significant for every country (all p<0.025).

### Conclusion

Excellent psychometric properties were present across all 13 languages examined. Unidimensionality of the DLQI construct has been confirmed and the DLQI is able to differentiate between levels of disease severity and EQ-5D levels across all these languages. This adds further confidence in the appropriateness of using the DLQI across this range of languages.

## 208.3 Data and Methods to Support Translation of Critical COA Terminology in a Shifting Linguistic Validation Landscape: Improving Quality of Life in the AI-mediated Age

Tim Poepsel^1^, Rebecca Israel^2^, Allyson Nolde^3^, Chryso Hadjidemetriou^4^, Rachael Browning^4^

^1^RWS, St. Louis, Missouri, USA, ^2^RWS, Philadelphia, Pennsylvania, USA, ^3^RWS, Chicago, Illinois, USA, ^4^RWS, London, UK

*Journal of Patient-Reported Outcomes 2026*, **10(Suppl 1)**:208.3

### Aims

Linguistic validation (LV) aims to ensure well-comprehended, culturally relevant COA translations for use in global clinical trials. An important role of LV is identifying solutions for difficult-to-understand or translate COA terminology. An emerging role given increased attention on AI-mediated translation is building translation databases for common and critical COA concepts, to enable accurate, consistent, and fit-for-purpose translation across many applications and levels of human engagement in translation. Such databases can also minimize the impact of applying AI translation techniques to languages infrequently used in clinical trials, where insufficient training data may exist for critical COA concepts. Aiming to develop these databases, we surveyed translators from many languages and countries on translation of the frequent and increasingly emphasized term “quality of life” (QoL), a multidimensional assessment of physical and psychological wellbeing related to disease and treatment.

### Methods

Translators (n=79) representing 43 countries and 41 languages were surveyed on the term “QoL”. Questions focused on the term’s language-specific meaning, cultural relevance, translations, elaborations, and difficulty of interpretation. Translators averaged 24 years of experience and 97.5% had college or graduate degrees.

### Results

Translators provided the most common “QoL” translations for each language, totalling 37 unique translations. A secondary translation was provided in 17 languages. 35% of languages additionally recommended and provided descriptive phrases to improve patient comprehension. 79% of linguists reported QoL is commonly discussed in their culture, and 86% considered its translation linguistically straightforward, even if further conceptual elaboration was recommended. Thematic analysis of feedback on language-specific meanings of QoL identified social health (n=85), physical health/functionality (n=68), psychological health (n=61), and financial health (n=54) as highly frequent interpretations. Variation in interpretation across languages was low.

### Conclusion

We present data to support accurate, consistent translation and elaboration of “Quality of Life” in 40+ languages within an HRQol COA context. Compiling robust human-generated databases for key COA concepts across diverse linguistic and cultural contexts is increasingly needed as LV and AI-mediated translation techniques begin to intersect and guidelines for the relationship develop. This approach also strengthens global COA data collection against conceptual drift across language and culture, or changing word usage over time.

## 209.3 Recovery from Breast Cancer Surgery: Implementation of the ICHOM Standard Order to Understand the Patient Perspective

Eva Roy^1^, Amanda Higgins^1^, Mariem Ahmed^1^, Maria Edelen^1^, Chengbo Zeng^1^, Manraj Kaur^1^, Andrea Pusic^1^

^1^Brigham and Women’s Hospital, Boston, Massachusetts, USA

*Journal of Patient-Reported Outcomes 2026*, **10(Suppl 1)**:209.3

### Aims

This project aims to evaluate the implementation of the International Consortium for Health Outcomes Measurement (ICHOM) Standard Set for breast cancer to better understand the patient perspective during recovery from breast cancer surgery. Specifically, we sought to (1) integrate patient-reported outcomes into the pre- and postoperative care process, (2) assessed how the ICHOM Standard Set captures meaningful aspects of recovery, and (3) identified opportunities to enhance patient-centered care by aligning clinical practices with the values and experiences reported by patients.

### Methods

Eligible patients were identified through schedules at designated clinics at Dana Farber Cancer Institute and Brigham and Women’s hospital from June 2021 to September 2024. Patients downloaded imPROVE, our breast care companion app, and were asked to complete assessments pre-operatively and then post-operatively at 2 and 6 weeks and 3, 6, and 12 months. These patient-reported outcome (PRO) assessments included the BREAST-Q and the EORTC QLQ C-30.

### Results

A total of 2,562 EORTC QLQ-C30 were completed by 2,428 unique patients, of whom 121 (5%) completed two or more times. Of the 2,562 completions, there were 1,618 (63.2%) in phase 1 (pre-operatively), 256 (10%) in phase 2 (1 day to 6 weeks post-operatively), 244 (9.5%) in phase 3 (> 6 weeks to 6 months post-operatively), and 444 (17.3%) in phase 4 (> 6 months post-operatively). Surgeries ranged from lumpectomy, mastectomy with flat closure, implant reconstruction, and autologous reconstruction including DIEPs. Looking at the BREAST-Q, the average psychosocial well being scores in phase 1 were higher than those in phases 2 and 3, but lower than those in phase 4. The average physical wellbeing, satisfaction with breast, and sexual wellbeing scores in phase 1 were higher than those at later phases. Regarding the EORTC QLQ-C30, cognitive, social and emotional function were higher in phase 4, as compared with phase 1. Financial difficulties were greater in phases 2 and 3, compared with phases 1 and 4.

### Conclusion

Here we report the successful implementation of the ICHOM breast cancer PROMs order set into clinical care. Our results show that breast cancer surgery has significant impact on physical function and other aspects of quality of life. The findings from this study can help guide clinicians with preoperative counseling and provide patients with tailored resources for support in the recovery process.

## 209.5 How does Surgical Complexity impact Health-Related Quality of Life in Patients with Ovarian Cancer?

Tanja He Heidelberg^1^, Chengbo Zeng^2^, Manraj Kaur^2^, Maria O. Edelen^2^, Jason B. Liu^3^, Andrea L. Pusic^2^, Rachel Sisodia^4^

^1^National Center for Tumor Diseases (NCT)/Heidelberg University Hospital (UFK)/Patient Reported Outcomes, Value and Experience (PROVE) Center, Department of Surgery, Brigham and Women’s Hospital, Heidelberg Germany, ^2^Patient Reported Outcomes, Value and Experience (PROVE) Center, Department of Surgery, Brigham and Women’s Hospital, Boston, MA, USA, ^3^MD Anderson Cancer Center, Houston, Texas, USA, ^4^Gynecologic Oncology, Mass General Cancer Center, Boston, USA

*Journal of Patient-Reported Outcomes 2026*, **10(Suppl 1)**:209.5

### Aims

When planning treatment for advanced ovarian cancer, health-related quality of life (HRQL) is a key consideration alongside oncologic outcome. Successful cytoreductive surgery with minimal residual disease is associated with improved overall survival. While prior studies have identified surgical complexity and extensiveness as significant short-term determinant of HRQL, its long-term implications remain uncertain.

### Methods

This analysis comprised data from 350 patients with ovarian cancer treated within an integrated health system between January 2016 and December 2023. All patients completed the EORTC QLQ-C30 Questionnaire pre-operatively and at least once post-operatively at 30, 90, 180, or 360 days to assess symptoms and functional status. Surgical complexity was evaluated using the validated Aletti score, with a threshold of ≥4 indicating high-complexity procedures. To examine the impact of surgical complexity on PROM scores over time, we employed a linear mixed-effects model, analyzing both functional and symptom domains while adjusting for demographic and clinical characteristics.

### Results

54.9% of patients were older than 65 years, 30.0% underwent high-complexity surgery, and 64.0% received chemotherapy. Over time, functioning scores showed a notable improvement at 180 days postoperative (β = 3.13 [0.77; 5.48]) and a further significant increase at 360 days postoperative (β = 6.57 [3.95; 9.18]). Symptom burden decreased at 180 days postoperative (β = -2.15 [-4.20; -0.09]) and at 360 days (β = -4.81 [-7.10; -2.52]).At 30 days postoperatively, patients after high-complexity surgery experienced a significant decline in functioning (β = –6.74 [-10.97; -2.51]). This effect diminished at 90 days postoperatively (β = –0.07 [-4.12; 3.99]).

### Conclusion

When counseling patients in clinical decision-making, managing expectations is essential. The impact of complex surgery on health-related quality of life (HRQL) appears to be short-term, while its long-term effects are comparable to those of moderate surgery. Our data, derived from routine PROM collection, is highly likely to reflect real-world application and usage. Our findings can help inform patient discussions and guide treatment decisions based on real world clinical data.

**Fig. 1(abstract 209.5):**
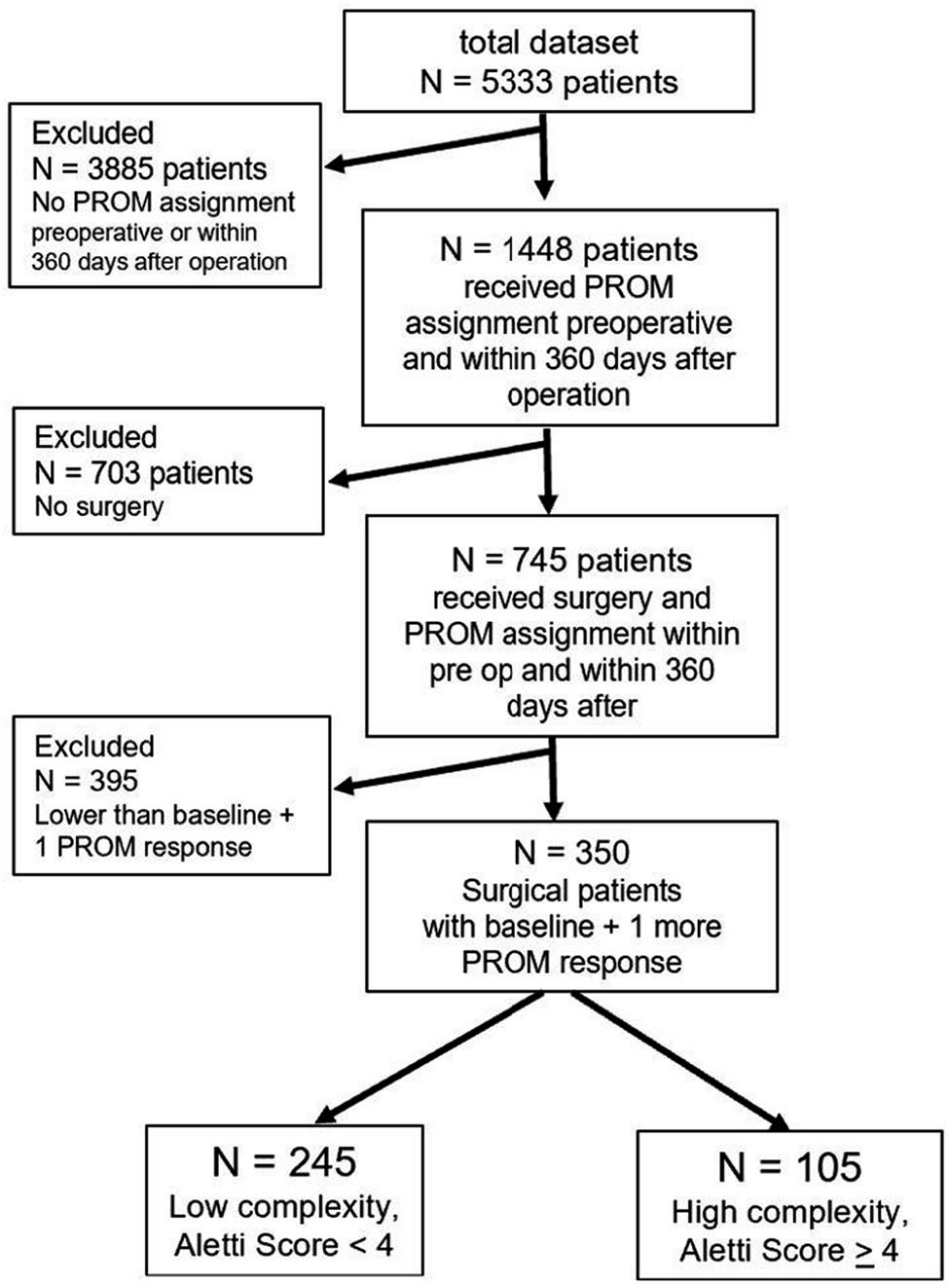
Patient flow chart: Patients with ovarian cancers treated and undergoing surgery between January 2016 – December 2023, with at least two PROM submissions

**Table 1 (abstract 209.5):**
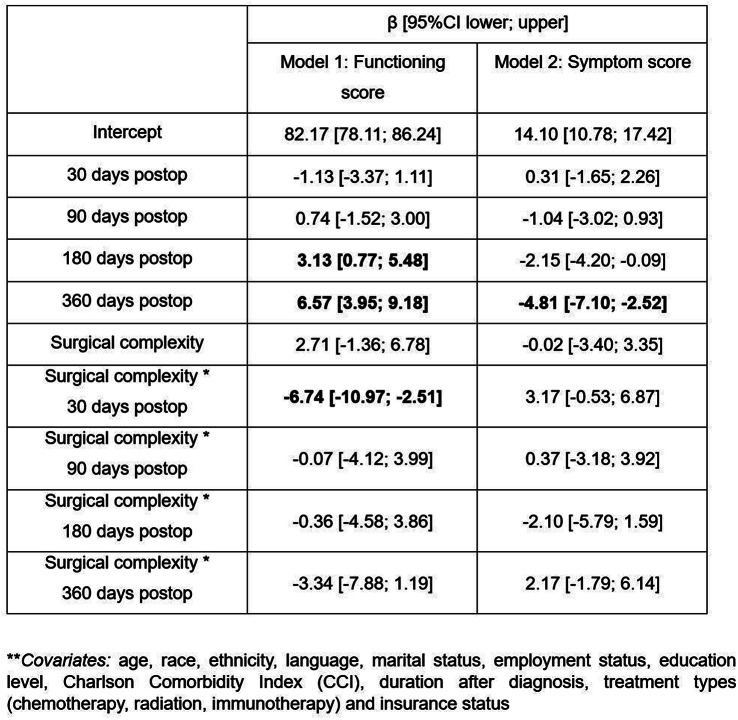
Results of linear mixed effect model**

**Fig. 1 (abstract 209.5):**
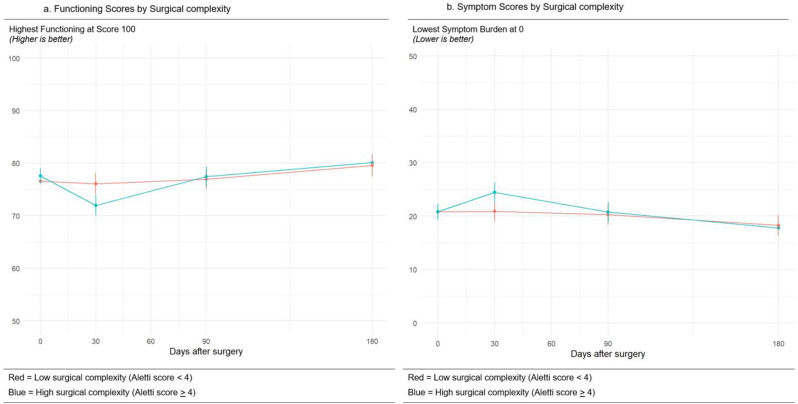
Plots of Symptom and Functioning by Subgroups

## 209.4 Assessing Socioeconomic Disparities in Patient-Reported Outcome Measure Response Rates Among Ovarian Cancer Surgery Patients

Tanja He^1^, Chengbo Zeng^2^, Manraj Kaur^2^, Maria O. Edelen^2^, Jason B. Liu^3^, Andrea L. Pusic^2^, Rachel Sisodia^4^

^1^Heidelberg National Center for Tumor Diseases (NCT)/Heidelberg University Hospital (UFK)/Patient Reported Outcomes, Value and Experience (PROVE) Center, Department of Surgery, Brigham and Women’s Hospital, Heidelberg, Germany, ^2^Patient Reported Outcomes, Value and Experience (PROVE) Center, Department of Surgery, Brigham and Women’s Hospital, Boston, MA, USA, ^3^MD Anderson Cancer Center, Houston, Texas, USA, ^4^Gynecologic Oncology, Mass General Cancer Center, Boston, USA

*Journal of Patient-Reported Outcomes 2026*, **10(Suppl 1)**:209.4

### Aims

Nonresponse to PROMs can impose bias in HRQL research and, clinically, higher response rates have been associated with better survival outcomes. Although ovarian cancer patients generally show high PROM completion, response rates vary across subgroups—limiting clinical utility and potentially widening disparities. Our study explores factors contributing to nonresponse in ovarian cancer patients, aiming to improve response rates and reduce biases in clinical practice and research.

### Methods

This analysis included 745 patients with ovarian cancer within an integrated health system undergoing surgical treatment from January 2016 to December 2023. All patients were assigned with the general QLQ-C30 and the specific OV-28 preoperatively and postoperatively at 30, 90, 180, or 360 days. Non-Response was defined as no record of any PROM assignment. We compared the differences in demographic and clinical characteristics between responders and non-responders using Chi-square tests and identified the significant predictors of nonresponse using logistic regression with stepwise selection.

### Results

In total, 178 patients (23.9%) did not respond to any assigned PROMs. Age was evenly distributed, with a total of 382 (52.3%) older than 65 years. Lower response rates were found in participants with high school education or less (69.3%), unknown insurance status (66.7%) and being non-English-speaking (44.7%). In the logistic regression analysis, disabled or retired individuals were significantly less likely to respond compared to employed or students (0.395 [0.254–0.614]). Participants who were separated, divorced, or widowed were less likely to respond than those who were married or living in a civil union (0.496 [0.320–0.771]). Participants with unknown or declined racial status were less likely to respond than their white counterparts (0.253 [0.144–0.445]).

### Conclusion

Overall, we found higher rate of nonresponse in historically underserved populations, including non-White, unemployed, separated, divorced, or widowed individuals. Our data, derived from clinical routine, reflects real-world issues in PROM collection. Higher PROM response rates can enhance clinical outcomes and support more inclusive future research. Ensuring better data collection from these underserved populations is essential to reducing healthcare disparities and should be targeted in future studies.

**Fig. 1 (abstract 209.4):**
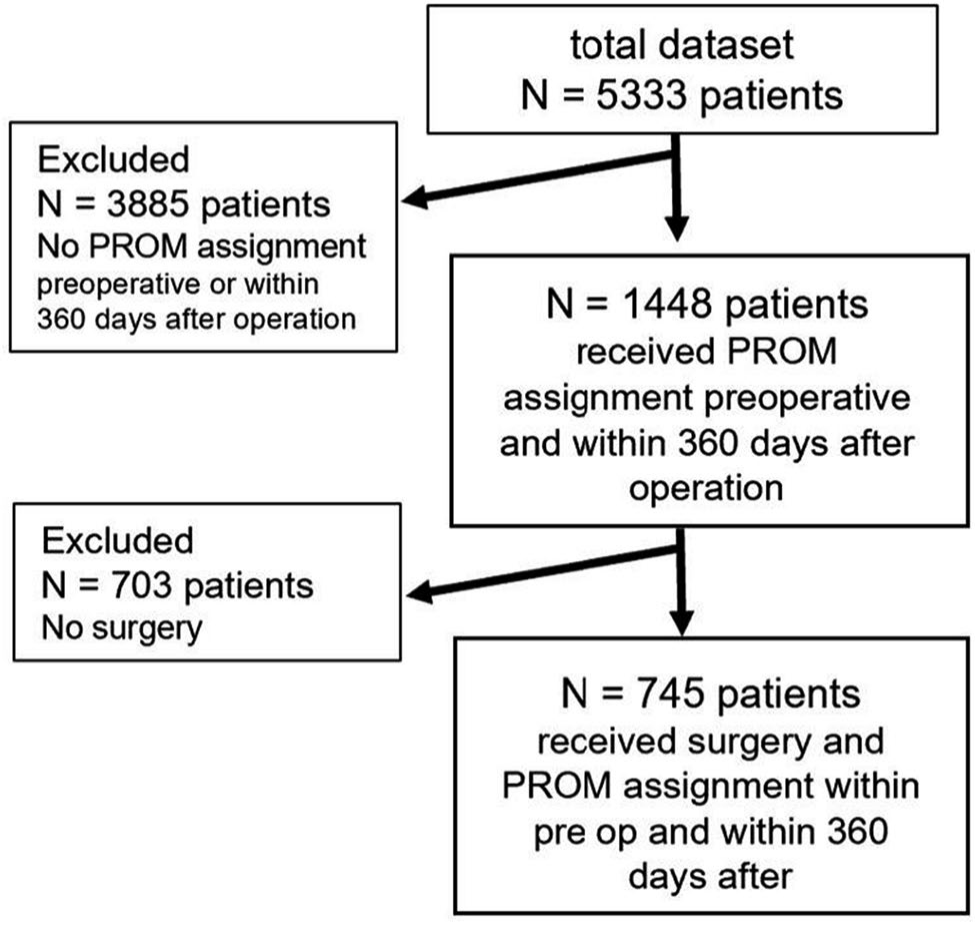
Patient flow chart: Patients with ovarian cancers undergoing surgery between January 2016 – December 2023

**Table 1 (abstract 209.2):**
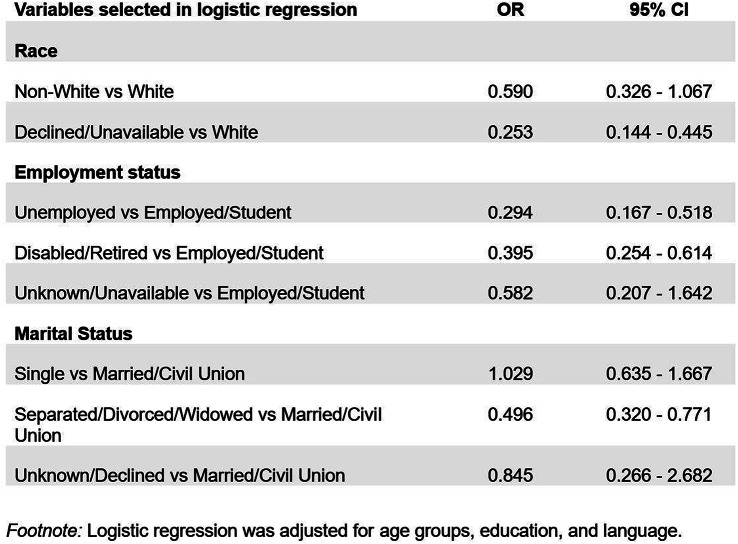
Logistic regression of nonresponse predicted by demographic and clinical characteristics among the 745 patients with ovarian cancers treated between January 2016–December 2023

## 209.2 Health-Related Quality of Life (HRQoL) Assessment using the Validated Mongolian FACT-ES among Mongolian Women with Breast Cancer (BC) on Adjuvant Endocrine Therapy (AET)

Rila Su^1^, Claire Snyder^2^, Albert W. Wu^3^, Alden L. Gross^4^, Jiafu Ji^5^, Laura Morlock^3^

^1^Inner Mongolia People’s Hosptial, Hohhot, China, ^2^Johns Hopkins Department of Medicine, Johns Hopkins University School of Medicine, Balitimore, Maryland, USA, ^3^Department of Health Policy and Management, Johns Hopkins Bloomberg School of Public Health, Baltimore, Maryland, USA, ^4^Department of Epidemiology, Johns Hopkins Bloomberg School of Public Health, Baltimore, Maryland, USA, ^5^State Key Laboratory of Holistic Integrative Management of Gastrointestinal Cancers, GI Cancer Center of Peking University Cancer Hospital & Institute, Beijing, China

*Journal of Patient-Reported Outcomes 2026*, **10(Suppl 1)**:209.2

### Aims

Breast cancer (BC) is the most common malignancy for women worldwide. Mongolian women face socioeconomic barriers that may affect their health-related quality of life (HRQoL), particularly during adjuvant endocrine therapy (AET). This study assessed the HRQoL of Mongolian BC patients undergoing AET.

### Methods

This was a descriptive, cross-sectional study of Mongolian BC patients on AET conducted. between September and October 2024. Participants recruited from two hospitals in Inner Mongolia, China completed the Functional Assessment of Cancer Therapy-Endocrine Symptoms (FACT-ES) and Eastern Cooperative Oncology Group (ECOG) performance status instruments.

### Results

Of 215 patients approached, 200 participated (response rate 93%) and completed the survey. The mean age = 55.45 ± 9.63 years; 72.5% were Stage I-II, 76% had mastectomy, and 12% had breast-conserving surgery. Over 20% of participants reported 14 symptoms. Symptoms reported “very much” or “quite a bit” included joint pain (14.5%), loss of interest in sex (13%), and hot flashes (10%). Compared to healthy females and female patients with other cancers in the U.S. and China, study participants reported the lowest scores for emotional well-being (EWB) (16.6 vs 19.4, 18.7, 17.5, 17.7, respectively). However, they reported the highest functional well-being (FWB) (20.5 vs 18.3, 19.5, 19.5, 17.0, 16.9, respectively). Multivariate regression analysis revealed that ECOG performance status was negatively associated with all subscales and overall FACT-ES, with physical well-being (PWB) (R^2^ =0.274, P<0.001), social well-being (SWB) (R^2^ =0.289, P<0.001), EWB (R^2^ =0.115, P<0.001), FWB (R^2^ =0.294, P<0.001), ES (R^2^ =0.072, P=0.001), and overall FACT-ES (R^2^ =0.282, P<0.001). Annual income was associated with EWB (R^2^ =0.115, P=0.009), and those with >200,000 RMB/year (27,357 USD) had better HRQoL versus those with < 20,000 RMB/year (P<0.008). Occupational status was associated with PWB (R^2^ =0.274, P=0.033), SWB (R^2^ =0.288, P=0.019), and EWB (R^2^ =0.115, P=0.001), and employed participants reported higher HRQoL vs unemployed participants (P < 0.004). Cancer stage did not show significant associations, but stage I patients reported significantly better HRQoL vs Stage II and III (P=0.024, P<0.001).

### Conclusion

These findings underscore the complex relationships between AET and HRQoL and specific challenges faced by Mongolian women with BC receiving AET, reinforcing the need for multidimensional approaches to improving HRQoL.

## 209.1 A systematic review of Patient-Reported Outcome Measures (PROMs) to assess Health-Related Quality of Life (HRQoL) for breast cancer patients on adjuvant endocrine therapy: Application of the COSMIN guidelines

Rila Su^1^, Claire Snyder^2^, Albert W. Wu^3^, Alden L. Gross^4^, Jiafu Ji^5^, Laura Morlock^3^

^1^Inner Mongolia People’s Hosptial, Hohhot, China, ^2^Johns Hopkins Department of Medicine, Johns Hopkins University School of Medicine, Balitimore, Maryland, USA, ^3^Department of Health Policy and Management, Johns Hopkins Bloomberg School of Public Health, Baltimore, Maryland, USA, ^4^Department of Epidemiology, Johns Hopkins Bloomberg School of Public Health, Baltimore, Maryland, USA, ^5^State Key Laboratory of Holistic Integrative Management of Gastrointestinal Cancers, GI Cancer Center of Peking University Cancer Hospital & Institute, Beijing, China

*Journal of Patient-Reported Outcomes 2026*, **10(Suppl 1)**:209.1

### Aims

Adjuvant Endocrine Therapy (AET) reduces the recurrence of hormone receptor-positive breast cancer, but its side effects negatively impact patients’ health-related quality of life (HRQoL). We conducted a systematic review to identify and evaluate patient-reported outcome measures (PROMs) to assess HRQoL in breast cancer patients undergoing AET using the COnsensus-based Standards for the selection of health Measurement INstruments (COSMIN) guidelines.

### Methods

A systematic search was conducted across MEDLINE, EMBASE, Cochrane Library, and Google Scholar. The measurement properties of each PROM were appraised following COSMIN guidelines, and their relevance, comprehensiveness, and comprehensibility were assessed. The quality of the evidence was rated using Grading of Recommendations Assessment, Development, and Evaluation (GRADE), and an overall recommendation was made for each PROM.

### Results

The review included 24 studies assessing six PROMs (EORTC-BR23, EORTC-BR45/42, FACT-B, FACT-Endocrine Symptoms (ES), FACT Breast Symptom Index, Menopause-Specific Quality of life). PROMs were assessed for comprehensiveness regarding the 16 most common symptoms with high patient-reported intensity and distress. EORTC-BR45/42 covered 14/16 and FACT-ES covered 15/16 symptoms and were selected for further evaluation of psychometric performance. Studies assessed the structural validity of EORTC-BR45/42 using confirmatory factor analysis with RMSEA ≤0.05, SRMR ≤0.08 for most, while no study assessed these for FACT-ES. Both PROMs demonstrated strong internal consistency, with Cronbach’s α > 0.70 in most subscales across studies. EORTC-BR45/42 showed acceptable test-retest reliability and intraclass correlation (r=0.768). Test-retest reliability of FACT-ES was 0.86. Study results aligned with hypotheses for construct validity. FACT-ES was tested for responsiveness, with SRM>0.5. Both PROMs were rated adequate or very good in the methodological rating.

### Conclusion

Based on this systematic review using a modified COSMIN approach, EORTC-BR45/42 and FACT-ES are recommended for assessing HRQoL in breast cancer patients undergoing AET. This study underscores the importance of capturing the full spectrum of patient symptoms of a treatment in PROM selection. Identifying relevant PROMs can inform clinical trial design for targeted and personalized interventions. The results can ultimately be used by clinicians to improve patients’ HRQoL.

## 210.2 Individual and Community-Level Social Determinants Associated with Representation in a Cellular Therapy Registry

Carlos Litovich^1^, Miranda Kapfhammer^1^, Sarah Thryselius^1^, Deborah Mattila^2^, Nick Feng^1^, Mehdi Hamadani^1^, Bronwen Shaw^1^, Jessica Olson^1^, David Nelson^1^, Rachel Cusatis^1^

^1^Medical College of Wisconsin, Milwaukee, Wisconsin, USA, ^2^NMDP, Minneapolis, Minnesota, USA

*Journal of Patient-Reported Outcomes 2026*, **10(Suppl 1)**:210.2

### Aims

Representation in national clinical and patient-reported outcome (PRO) registries is driven by both clinical and sociocultural factors. While disparities in clinical outcomes data collection have been studied, less is known about the impact of community-level characteristics on PRO participation. This study uses data from a national registry to examine how social determinants, such as social vulnerability and rurality, are associated with representation in a subset of patients eligible for PRO data collection after hematopoietic cell transplantation (HCT) or cellular therapy (CT).

### Methods

Data were analyzed from the Center for International Blood and Marrow Transplant Research (CIBMTR), which collects clinical and PRO data on HCT/CT recipients in the US. PRO data are collected only from a subset of participating centers. We assessed two outcomes: agreement to be contacted for future research, a prerequisite for PRO participation, using multivariable logistic regression; and submission of at least one PRO survey using a Fine-Gray subdistribution hazard model to account for the competing risk of death without survey submission. Key covariates included race, ethnicity, age, sex, treatment type, functional status, rurality, and the Social Vulnerability Index (SVI).

### Results

Among 97,255 patients from 55 centers, 67.4% agreed to be contacted for future research. Race, ethnicity, age, sex, treatment type, functional status, rurality, and SVI were significantly associated with agreement. Patients from less vulnerable and rural areas had 38% and 8% higher odds of agreement, respectively. In contrast, Black, Hispanic, female, and lower functional status patients had 74%, 67%, 87%, and 95% lower odds of agreement, respectively. Among 3,083 patients from centers participating in PRO collection, 27.6% submitted at least one survey. Race, ethnicity, age, treatment type, and functional status were associated with submission in the multivariable analysis; however, sex, rurality, and SVI were not. Hispanic, Black, and Native Hawaiian or Pacific Islander participants were about half as likely to submit at least one PRO.

### Conclusion

Community-level characteristics influence who is represented in a national clinical registry. While disparities exist in who agrees to be contacted for research, these differences diminish once patients are engaged. Tailored engagement strategies may help improve equitable representation in PRO data collection.

## 210.3 Barriers and facilitators to Patient Reported Outcome (PRO) collection. Interim findings from a mixed-methods, multi-site study

Joseph Lanario^1^, Nicola Anderson^1^, Olalekan Lee Aiyegbusi^1^, Ameeta Retzer^1^, Foram Khatsuria^1^, Nancy Bhardwaj^1^, Sarah Hughes^1^, Christel McMullan^1^, Flic Gaete Carvajal^1^, Philip Collis^1^, Roger Wilson^1^, Kamil Sterniczuk^1^, Yvonne Yalder^1^, Sarah Markham^1^, Emily Lam^1^, Clarice McKay^1^, Ian Clarke^1^, Robert Michell^1^, John Rose^1^, Samantha Drewett^1^, Melanie Calvert^1^

^1^University of Birmingham, Birmingham, UK

*Journal of Patient-Reported Outcomes 2026*, **10(Suppl 1)**:210.3

### Aims

Patient-Reported Outcomes (PROs) can provide valuable insights into the impact of disease and treatment from the patient perspective. We aimed to explore barriers of collecting PRO data in research and routine care settings, identify potential solutions with particular focus on the inclusivity, accessibility and acceptability of PRO collection.

### Methods

A mixed methods study using a cross-sectional survey and semi-structured interviews, recruiting people attending outpatient clinic appointments from five hospitals in Birmingham, UK. Participants completed the 18-question survey which was co-designed with patient-partners to improve the relevancy and usability. This interim analysis used data from completed surveys only.

### Results

To date, a total of 613 participants have enrolled in the study, of which 503 provided completed surveys. Preliminary findings indicate most participants (58%) had not taken part in research before and, did not know someone who had taken part in research (77%). The majority (76%) were happy to complete PROs for health research. Most preferred electronic PROs (60%) compared to paper completion (20%). Most participants (67%) indicated they would be encouraged to complete a PRO if they perceived the questions as relevant. Barriers to providing PRO data were irrelevant questions (54%); being unsure how to answer the questions (23%); having to complete it in a phone call (20%); worries about how the data would be used (22%); and a health condition making some tasks difficult (18%). Enablers of PRO completion included, having a friend/relative there to complete it with you (29%); having a professional there to complete it with you (14%); having a phone number to call for help (13%) and having access to help resources online (12%).

### Conclusion

Findings highlight the importance of giving participants questionnaires that contain content relevant to them, and ensuring they know these PROs can influence their treatment/management. While there is a preference for electronic PROs, some preferred paper completion. This, in combination with the identified barriers can be addressed at the study design stage to ease PRO collection from people that may be otherwise excluded.

## 210.4 How do we ensure ePRO solutions meet accessibility standards?

Florence Mowlem^1^

^1^uMotif, London, UK

*Journal of Patient-Reported Outcomes 2026*, **10(Suppl 1)**:210.4

### Aims

Digital data capture has become the mainstream method of capturing patient-reported outcome measures (PROMs). Historically, concerns have existed as to whether the measurement properties of the measure will be impacted during the migration from paper to electronic. Largely, migration best practices have focused predominately on this issue. In contrast, app and web-design best practices outside of clinical research have focused on the accessibility and usability of these electronic systems for the widest range of users. Key issues with accessibility of patient-reported outcome measures themselves will be discussed, along with identifying where ePRO systems are not meeting web accessibility criteria, and determining opportunities to optimize PROM data capture by ensuring accessibility for all.

### Methods

Current best practices for the implementation of PROMs electronically were compared to Web Content Accessibility Guidelines (WCAG 2.2) to identify areas of (mis)alignment, where further evidence would be required to ensure the maintenance of the questionnaire measurement properties when applying a given accessibility success criteria, and what accessibility practices can be incorporated into ePROM best practices now.

### Results

The majority of the accessibility criteria focus on the software being programmed to support the implementation of content in a way that enhances overall accessibility and its usability with assistive technologies, will not impact the measurement properties of the measure, and so can be applied today. Some of the tensions identified between accessibility success criteria and ePROM best practices included content size and device orientation, as well as inaccessible response scale types.

### Conclusion

Robust data is critical to achieving trial endpoints, but suboptimal usability of ePRO systems can risk study success. As an industry we must ensure the accessibility of data capture systems to ensure we do not impact inclusion in clinical research, and question if we have been overly conservative when migrating measures from paper to electronic.

## 210.5 Evaluation of enrollment rates to PRO data collection in a large clinical registry in the US – the first 5 years

Idayat Akinola^1^, Deborah Mattila^2^, Miranda Kapfhammer^1^, Corrigan Luetke^2^, Bronwen E. Shaw^1^, Kathryn E. Flynn^1^, Rachel Cusatis^1^

^1^Medical College of Wisconsin, Milwaukee, Wisconsin, USA, ^2^NMDP, Minneapolis, Minnesota, USA

*Journal of Patient-Reported Outcomes 2026*, **10(Suppl 1)**:210.5

### Aims

The CIBMTR® (Center for International Blood and Marrow Transplant Research®) collects clinical data from >300 medical centers worldwide on hematopoietic cell transplantation (HCT), chimeric antigen receptor therapy (CAR-T), and other cellular therapies (CT). In 2020, the CIBMTR launched collection of patient-reported outcomes (PRO) at 3 sites and has expanded to 43 sites. We evaluated the enrollment rates to PRO data collection based on mode of initial outreach.

### Methods

Adult HCT/CT patients at U.S. centers who agree to be contacted by the CIBMTR and understand English or Spanish are invited to participate. Initial invitation to participate is conducted via email and phone. CIBMTR makes up to 6 calls and sends 2–3 emails before closing outreach to unresponsive patients. NIH PROMIS measures assess mental, physical, and social health. The COST-FACIT measure assesses financial toxicity, and individual items on occupational function, social determinants of health, and caregiver availability are captured. Surveys are collected electronically, on paper, or via telephone at multiple timepoints. In 2024, a 3-month pilot program evaluated whether initial outreach by postal mail affected enrollment among groups with low enrollment rates: those of Hispanic/Latino ethnicity and those without email addresses. Enrollment rates by initial contact are described first, followed by pilot program results.

### Results

From August 2020 to August 2024, 4,463 patients were approached, with 1,454 (32.6%) enrolled, a rate that stayed consistent across these years. Among patients who enrolled, the average number of contacts was 2.3 emails and 3.3 calls if initially contacted by email compared to 1.7 emails and 3.5 calls if initially contacted by phone. During the 3-month letter pilot, of the 44 Hispanic/Latino patients approached, 21 (46.7%) enrolled, compared to 176 (25.3%) of the 985 approached pre-pilot. For patients with no email address numbers were small; 2 (15%) of the 13 approached during the pilot enrolled compared to 49 (18%) of the 275 approached pre-pilot.

### Conclusion

Overall enrollment rates for CIBMTR PRO data collection have remained consistent with small differences between initial contact by email or phone. Tailored outreach methods may improve enrollment rates. Future research will evaluate postal (mail) outreach in other historically underserved groups.

**Fig. 1 (abstract 210.5a):**
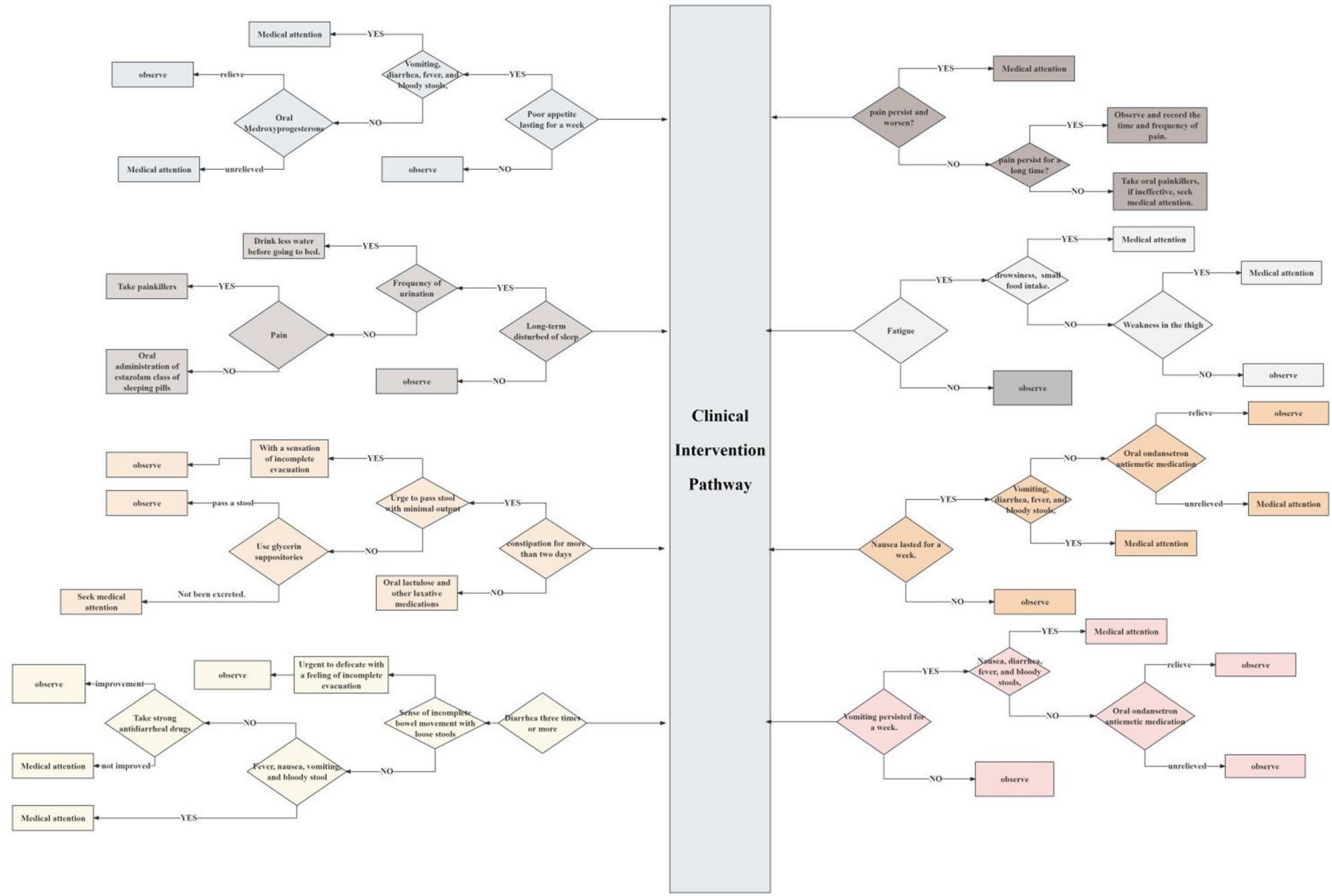
Evaluation of enrollment rates to PRO data collection in a large clinical registry in the US – the first 5 years

**Fig. 1 (abstract 210.5b):**
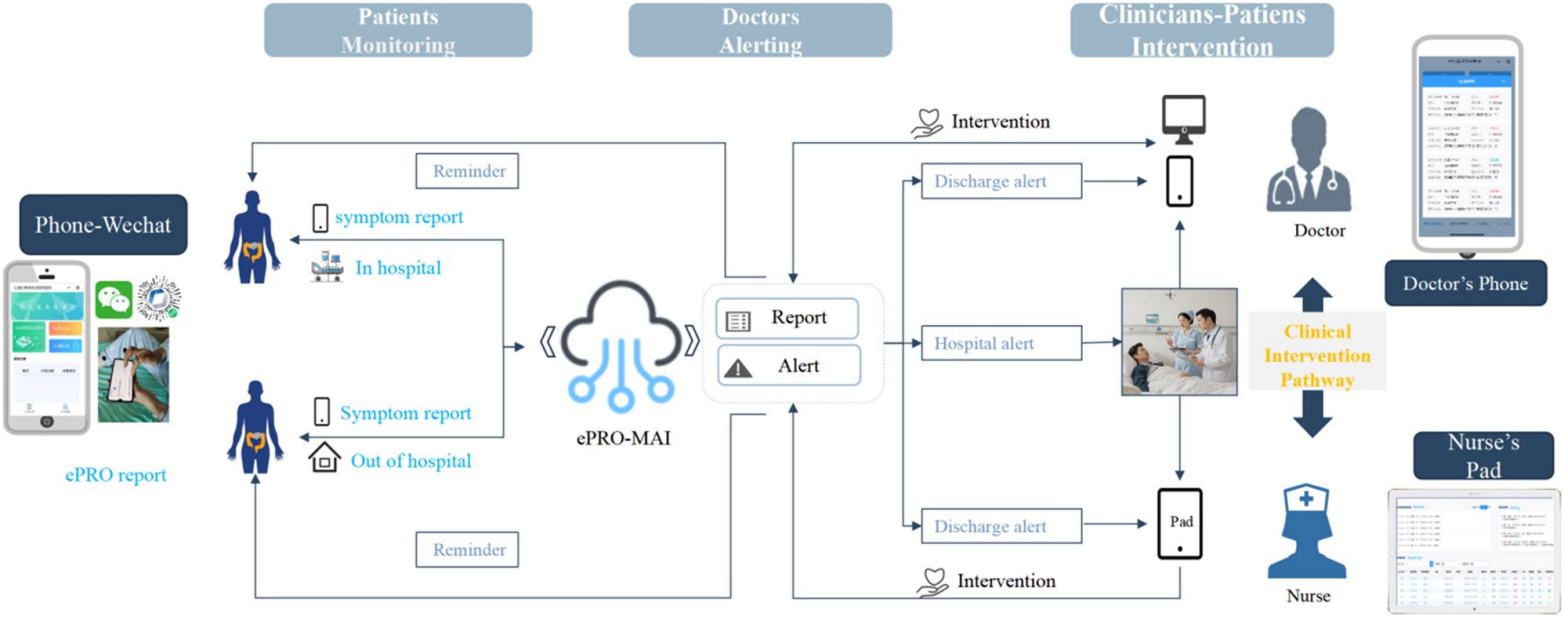
Evaluation of enrollment rates to PRO data collection in a large clinical registry in the US – the first 5 years

**Fig. 1 (abstract 210.5c):**
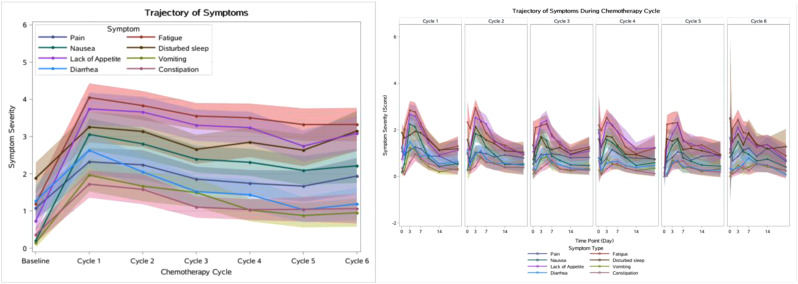
Evaluation of enrollment rates to PRO data collection in a large clinical registry in the US – the first 5 years

## 210.1 Implementation of patient-reported outcome measures in clinical registries worldwide

Rasa Ruseckaite^1^, Randi Jayasinghe^2^, Susannah Ahern^2^, Ashika Maharaj^2^

^1^Monash University, Melbourne Australia, ^2^School of Public Health and Preventive Medicine, Monash University, Melbourne, Victoria, Australia

*Journal of Patient-Reported Outcomes 2026*, **10(Suppl 1)**:210.1

### Aims

Collection of patient-reported outcomes measures (PROMs) is becoming common in clinical registries worldwide. However, no comprehensive, evidence-based guideline currently exists to effectively guide the implementation, data analysis and reporting of PROMs in clinical registries globally. This study seeks to understand the applicability of recently-developed Australian recommendations for PROMs inclusion, while also gaining insights into current practices, barriers, and enablers to PROMs implementation in the international clinical registries. The overarching goal of the project is to adapt the existing Australian recommendations for PROMs inclusion in clinical registries globally.

### Methods

This mixed methods study consisted of 1) a survey of international clinical registries to understand current practices for PROMs data collection and reporting, and 2) semi-structured interviews with international registry representatives to assess the relevance of Australian recommendations and to explore barriers and enablers for implementing PROMs abroad. The survey data were analysed using descriptive statistics, while qualitative interview data were analysed thematically.

### Results

A total of 43/67 survey responses were received, and 15 registries from seven countries were interviewed. Survey results revealed that 19 registries were aware of the existing Australian recommendations, and 12 had either developed new guidelines specific to their setting or adapted already existing ones. Twenty-nine registries used PROMs data for quality improvement purposes, and to assist clinicians with shared decision-making. Overall, the Australian recommendations for PROMs implementation were accepted positively. Thirteen of the 15 interviewees indicated that the Australian recommendations were applicable to their registries; however, they also agreed, that a comprehensive guideline tailored to the international clinical registries would be highly beneficial.

### Conclusion

Findings of this study indicated a large variation in approaches to PROMs program implementation across different clinical registries round the world. While registries utilised guidelines tailored to their specific needs, no generic guidelines have been developed for PROMs implementation. Therefore, a standard set of guidelines will ensure consistent implementation of PROMs, effective PROMs data capture, meaningful analysis and reporting of results in clinical registries globally.

## 301.5 The impact of severity prevalence on cut points estimated from receiver operating characteristic (ROC) curve analysis; implications for threshold and meaningful change estimation

Helen Doll^1^, Michael DeRosa^2^, Shruti Dave^2^, Alyssa Bamer^3^

^1^Clinical Outcomes Solutions, Folkestone, UK, ^2^Clinical Outcomes Solutions, Chicago, Illinois, USA, ^3^Clinical Outcomes Solutions, Tucson, Arizona, USA

*Journal of Patient-Reported Outcomes 2026*, **10(Suppl 1)**:301.5

### Aims

Receiver operating characteristic (ROC) curve analysis is often used to estimate thresholds related to meaningful change or condition severity. It is not widely recognized, however, that disease prevalence (or severity) impacts the thresholds estimated. This is called ‘spectrum bias’ in which the sensitivity and specificity of a particular cut point is dependent on prevalence. In this study we examined how ROC analysis cut points estimating severity thresholds differed when using different sample groupings from the same population, and how severity prevalence impacts the thresholds obtained.

### Methods

Using an existing longitudinal clinical trial dataset, we simulated data varying on disease prevalence. The dataset included Patient and Clinician Global Impression of Severity (PGIS/CGIS) anchor items with 5-point Likert scales (No, Almost No, Mild, Moderate, Severe symptoms) used to define mild, moderate, and severe scores on a multi-item Clinician Reported Outcome (ClinRO) scored 0-100. ROC analyses were completed to identify cut-points for severity thresholds between anchor categories, including a) the whole population (e.g., No to Moderate versus Severe) or b) only individuals in adjacent categories (e.g., Moderate versus Severe) to estimate the threshold, first at all timepoints in a stacked dataset and then split by timepoint (Baseline/Post-Baseline).

### Results

Initial findings suggest that the thresholds estimated differ based on both the strategy used for grouping patients (all categories or adjacent categories) and the level of patient severity (e.g., Baseline or Post-Baseline), both of which influence prevalence. For example, in the stacked analysis the Moderate/Severe threshold estimated was lower when patients in all anchor categories were included (lower severity prevalence) than when the analysis included only Moderate and Severe patients (higher prevalence). Because there were fewer Severe patients Post-Baseline, this effect was more marked Post-Baseline. Likewise, at Baseline, when there were more No/Almost No patients, the Mild vs No/Almost No threshold was lower when only adjacent categories were used (lower prevalence).

### Conclusion

This study shows how the prevalence of the condition and choice of inclusion of the full sample versus adjacent categories only directly affects ROC analysis cut points. The implications of this when using ROC analysis to estimate thresholds including those for meaningful change will be considered.

## 301.2 Establishing Meaningful Change Thresholds Through Personalized Narratives and Tailored Definitions: A Feasibility Study in Patients with Cancer

Kathleen Yost^1^, Rachel L. Benson^1^, Selena Daniels^2^, Amylou C. Dueck^3^, Elizabeth Duke^2^, Erica Horodniceanu^2^, Julie Schneider^2^, Vishal Bhatnagar^2^, Paul Kluetz^2^, Gita Thanarajasingam^1^, Minji Lee^1^

^1^Mayo Clinic, Rochester, Minnesota, USA, ^2^U.S. Food and Drug Administration, Silver Spring, Maryland, USA, ^3^Mayo Clinic, Scottsdale, Arizona, USA

*Journal of Patient-Reported Outcomes 2026*, **10(Suppl 1)**:301.2

### Aims

Establishing score thresholds for meaningful change typically relies on anchor-based methods applied to large sample data. Complementary methods to directly capture the patient’s perspective are needed. In our prior qualitative research, patients were able to articulate whether varying degrees of symptom changes were personally meaningful and to tailor individualized definitions. However, the extensive moderator guidance required limits scalability for routine applications. Our objective was to assess the feasibility of using a web-based survey to guide patients to personalize their definitions of meaningful change.

### Methods

We designed a baseline web-based survey to introduce adult patients with cancer to the concept of meaningful change by prompting them to provide a brief written description of their current fatigue and descriptions of recent days when their fatigue was noticeably worse or better. Starting with a basic definition of meaningful change, participants were instructed to tailor the definition to better reflect their experiences. Standardized patient-reported outcome measures (PROMs) of fatigue were also completed. After 6 weeks, participants completed another web-based survey that repeated the PROMs and included a fatigue global rating of change (GRC) item, with responses ranging from “Much Worse” to “Much better”. The 6-week survey then displayed the participants’ original descriptions of their fatigue at baseline, allowing them to reconsider and change their GRC response. Finally, the participants were shown their personalized definition of meaningful change from baseline, and they rated whether their change in fatigue in the last 6 weeks was meaningful according to that definition.

### Results

Administration of baseline surveys began March 24, 2025. As of abstract submission, 14 of the 80 patients contacted (17.5%) had completed the baseline survey. Preliminary data show 50% found providing descriptions of fatigue was “Easy” or “Very easy”, and 69% reported tailoring definitions of meaningful change was “Easy” or “Very easy”.

### Conclusion

The feasibility and patient acceptability of this personalized, web-based approach to defining meaningful change will be evaluated comprehensively upon completion of both baseline and 6-week assessments. Complete findings will be presented at the annual conference. If feasible, this approach may be useful for establishing meaningful change thresholds for clinical care and research, including clinical trials.

## 301.1

**What anchor correlation value should I use when deriving Meaningful Within Patient Change (MWPC) values**

Alexander Hind^1^, Mike Greenwood^1^, Kim Cocks^1^

^1^Adelphi Values, Manchester, UK

*Journal of Patient-Reported Outcomes 2026*, **10(Suppl 1)**:301.1

### Aims

Anchor-based analyses are routinely used to derive MWPC thresholds for Clinical Outcome Assessments (COAs) for use in clinical trials. The literature suggests that anchors should correlate with target COA by at least 0.3. It is also important to state, a priori, those anchors that are expected to correlate with the target COA. Researchers should always be wary of any post-hoc theories to justify an observed correlation as practical experience has shown that researchers can easily suggest a plausible rationale.

### Methods

Firstly, we used standard statistical formulae, and simulated data, to demonstrate the relationship between sample size and width of confidence intervals for different correlation values. Secondly, using random samples of observed COA data from clinical trials, we looked at the size of correlation that could be observed for different samples using clearly non-sensical anchors (anchors that can’t correlate with the target COA). The non-sensical anchors were derived from baseline height and weight.

### Results

Both the simulated results and the theoretical considerations show that a correlation of 0.3 is not reliable with low sample sizes (e.g. N=50) [Figure1] but is with larger sample sizes (e.g. N=500) [Figure2]. In the second part, results were demonstrated using random samples from study data with anonymised PRO instrument data and fake anchors. Correlations of 0.3 were observed with samples sizes as high as 150.

### Conclusion

There is no ‘one size fits all’ threshold for anchor relevancy. Both the simulation results and the results from real world data justify the need for thresholds to be varied given the sample size. Smaller sample sizes (e.g. <100) require a higher pre-specified threshold, as a threshold of 0.3 can pick up anchors that are non-sensical and unrelated to the target COA under consideration.

**Fig. 1 (abstract 301.1a, b):**
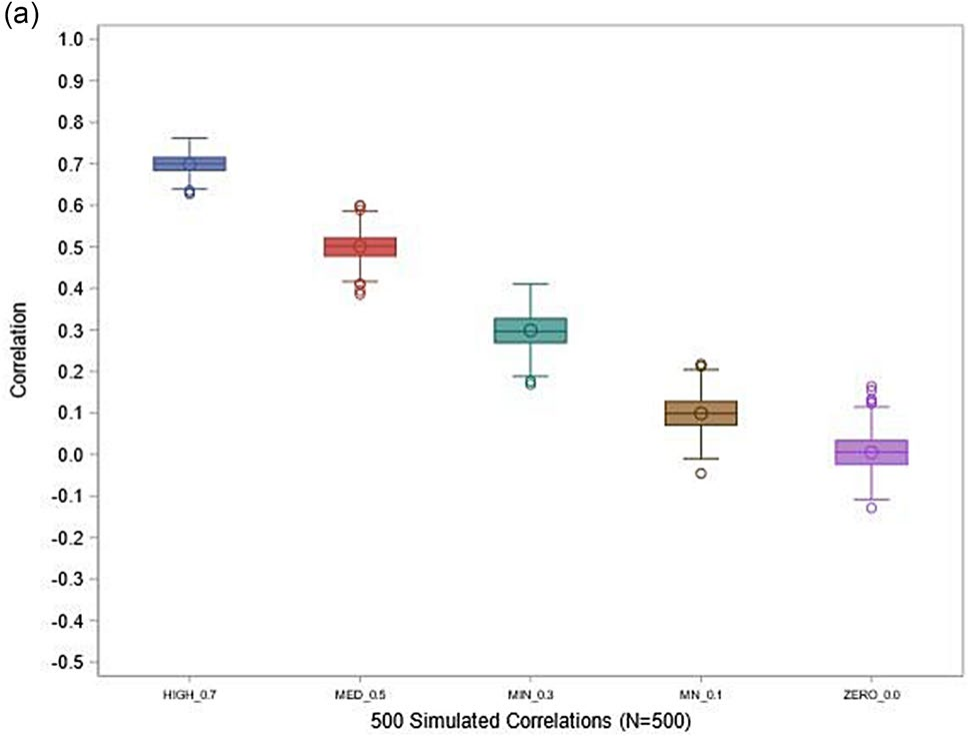

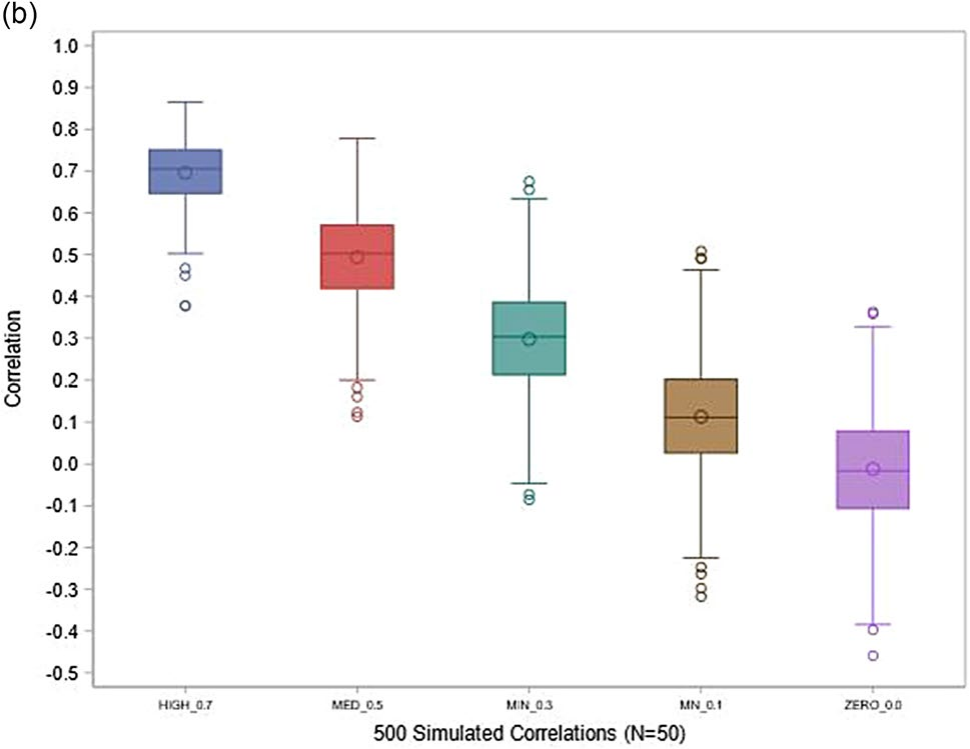
What anchor correlation value should I use when deriving Meaningful Within Patient Change (MWPC) values

## 301.3 A Comparison of Minimally Important Difference Definitions for Quality of Life in Four Clinical Trials

Salene Jones^1^, Evan Hall^1^, Cathee Till^1^, N. Lynn Henry^2^, Donna Barry^3^, Dawn Hershman^4^, Sapna Patel^5^, Joseph Unger^1^

^1^Fred Hutchinson Cancer Center, Seattle, Washington, USA, ^2^University of Michigan, Ann Arbor, Michigan, USA, ^3^University of Washington, Seattle, Washington, USA, ^4^Columbia, New York City, New York, USA, ^5^University of Colorado, Aurora, USA

*Journal of Patient-Reported Outcomes 2026*, **10(Suppl 1)**:301.3

### Aims

The minimally important difference (MID) represents how much change on a patient-reported outcome (PROs) is considered meaningful either for defining a patient as responding or worsening or for defining differences between groups. One challenge is the use of multiple MIDs with no gold standard. This study used four SWOG clinical trials with treatments of known effectiveness and toxicities and positive quality of life (QOL) results as the gold standard to compare different definitions of the MID.

### Methods

Two breast cancer trials (S1200 and S1202) compared true acupuncture (TA; S1200) to sham acupuncture (SA) and waitlist control (WC) or duloxetine (S1202) to placebo to improve PROs. The third trial (S9346) compared continuous versus intermittent androgen deprivation therapy in metastatic prostate cancer. The fourth trial (S1404) compared pembrolizumab with physician’s choice of interferon or ipilimumab in melanoma. Two MID distribution definitions (30% change, 50% change) and one MID anchor-based definition were compared. Logistic regression was used to compare the number of participants defined as improving or worsening between the two study groups using each MID definition, and the chi-square statistics for intervention effect were used to compare different definitions of MID.

### Results

In S1200, the anchor method performed well in distinguishing differences between intervention groups in four of eight comparisons (Figure 1), 50% change performed well in three of six comparisons and 30% change performed well in two of eight comparisons. In S1202, both the anchor method and 50% change performed well in two of three comparisons and the 30% change did not perform best for any comparisons. For S9346, the anchor method performed well in four of six comparisons, and 50% change and 30% change performed well in one of six comparisons each. For S1404, only 30% change performed well in the one comparison. Across all comparisons, the anchor method generated the largest mean chi-square values for both improvement (4.4) and worsening (2.5), followed closely by the 30% change (4.3 and 2.1).

### Conclusion

Results from these 4 trials suggest that anchor-based MID may be most sensitive to change with some cases where distribution methods are acceptable, particularly the 30% change.

**Fig. 1 (abstract 301.3):**
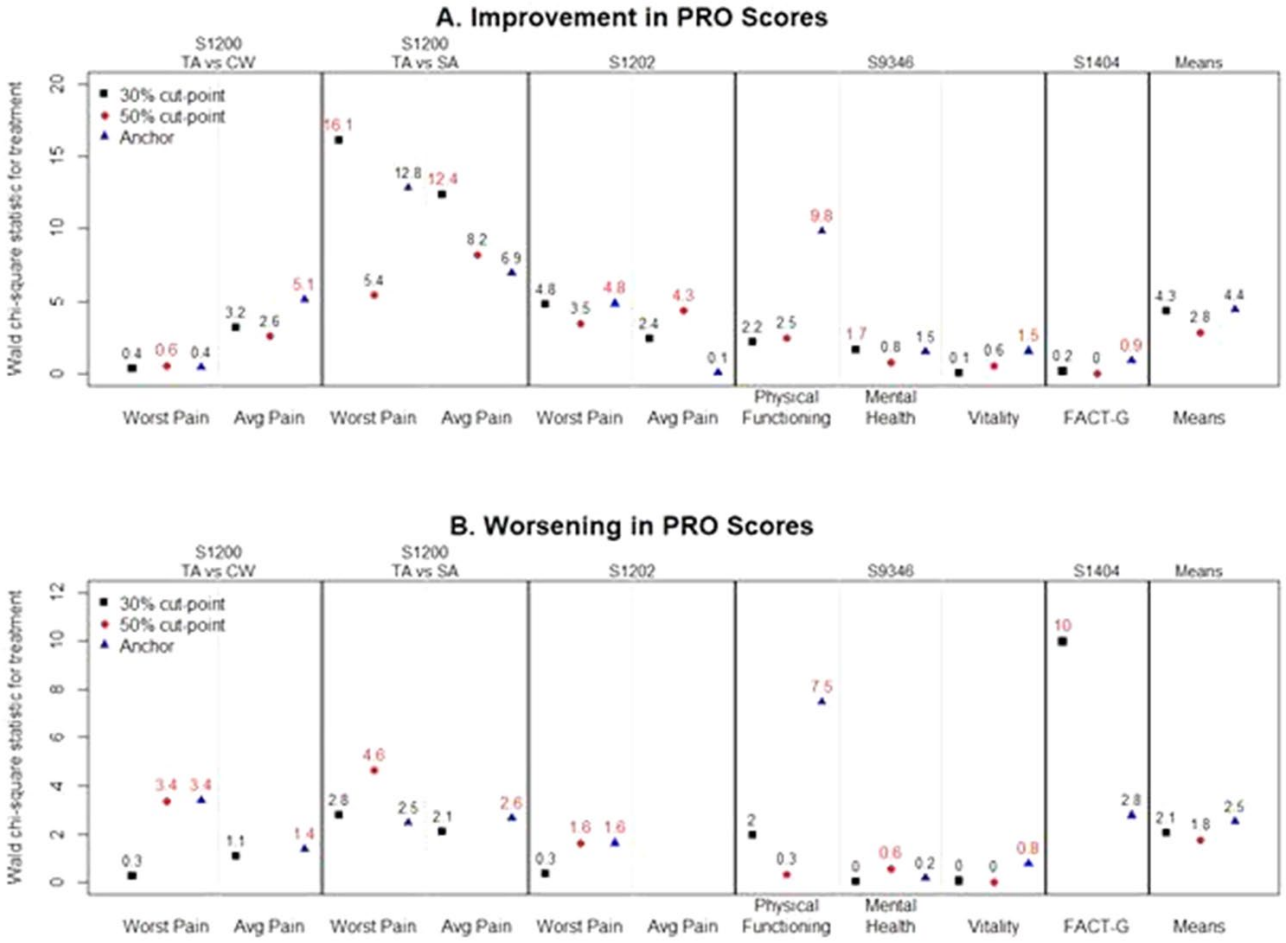
Chi-square statistics from logistic regression comparing number of participants who improved (**A**) or worsened (**B**) by study arms for different outcome and MID definitions

## 301.4 Which of these response choices would reflect a meaningful change? Methods of asking about meaningful change in qualitative interviews outside of a clinical trial

Chelsea Perschon^1^, Emilie Jaeger^1^, Sonya Eremenco^2^, Cheryl Coon^2^, Maria Mattera^2^, Sara Shaunfield^1^, Xiaodan Tang^1^, Jin-Shei Lai^1^, Karen Kaiser^1^

^1^Feinberg School of Medicine, Department of Medical Social Sciences, Chicago, Illinois, USA, ^2^Critical Path Institute, Patient-Reported Outcome Consortium, Tucson, Arizona, USA

*Journal of Patient-Reported Outcomes 2026*, **10(Suppl 1)**:301.4

### Aims

Meaningful change estimates for clinical outcome assessments (COAs) guide interpretation of COA data in clinical care, research, and drug development. Triangulating quantitative and qualitative data can clarify what amount of change will have a meaningful impact on patients’ lives. However, interviewing patients about meaningful change is challenging, given the theoretical and cognitively complex nature of questions about health status change, particularly without an intervention. We aim to advance this field by providing recommendations on how to address these challenges.

### Methods

A cognitive interview study of multiple sclerosis (MS) participants’ views of meaningful change on 10 single-item anchors of fatigue or physical function was conducted per the recommendation of FDA’s Center for Drug Evaluation and Research to support the quantitative anchor-based meaningful change analysis proposed under Drug Development Tool COA #000069. The interview guide was drafted via an iterative process with input from academic researchers specializing in qualitative methods and COA development, members of the PRO Consortium’s MS Working Group, and FDA.

### Results

During guide development, 3 issues emerged. First, should participants be asked to think back to a previous timepoint and define what would be a meaningful change today? Or should they look forward and consider what would be a meaningful change in the future? Second, what is the best way to ask participants about “no change”? Third, should participants be prompted to define their “minimum” level of meaningful change? After discussion and revisions, 1) participants were instructed to look forward when assessing meaningful change; 2) views of no change were elicited with the question, “How would you feel if your [concept] were to stay the same over time?” and 3) participants were asked about overall change not “minimum” change. Subsequent interviews with 26 people with MS demonstrated that these approaches worked well.

### Conclusion

Incorporating the patient perspective into considerations of meaningful change via qualitative methods aligns with patient-focused drug development. However, asking patients to evaluate hypothetical disease states presents challenges. Our study of MS participants’ views of meaningful change demonstrated that they can imagine future health, articulate the importance of change, and consider the significance of no change.

## 302.1 Predicting oncologic adverse events from patient-reported symptoms: AI models developed using data from a large national multicenter trial

Bahareh Modanloo^1^, Chris Gibbons^2^, Brenda Ginos^3^, Andre Pfob^4^, Amylou Dueck^3^, Ethan Basch^5^, Claire Snyder^1^

^1^Johns Hopkins University, Baltimore, Maryland, USA, ^2^Oracle Health, Austin, Texas, USA, ^3^Mayo Clinic, Scottsdale, Arizona, USA, ^4^Heidelberg University, Heidelberg, Germany, ^5^University of North Carolina at Chapel Hill, Chapel Hill, North Carolina, USA

*Journal of Patient-Reported Outcomes 2026*, **10(Suppl 1)**:302.1

### Aims

Nearly all patients receiving cancer therapies experience side-effects from their treatment. About 40% of patients will be admitted to the emergency department due to uncontrolled symptomatic side-effects. Evidence shows that routine electronic patient-reported outcome (ePRO) collection and feedback to clinical teams can facilitate proactive care which can improve key patient outcomes including health-related quality of life and survival. However, practical issues with ePROs have been identified which include missing data, late reports, and false positive reports.

### Methods

We use a retrospective design with data from the PRO-TECT (AFT-39, NCT03249090) cluster randomized trial, which includes 1,191 adults with advanced or metastatic cancer across 52 U.S. sites. After conducting feature engineering on ePRO responses, clinical, and demographic data, we develop machine learning models to predict symptom exacerbations and adverse events. Models include logistic regression with elastic net penalty, gradient boosted forests (XGBoost), and a neural network. Each model follows three steps: data preprocessing, training, and evaluation, with ten-fold cross-validation. We assess performance using Area under the Receiver Operating Characteristic Curve (AUROC), sensitivity, positive predictive value, and negative predictive value to evaluate accuracy and clinical applicability.

### Results

We will present the following results which will quantify the extent to which missing ePRO scores can be inferred as well as both how accurately and how far in advance symptomatic adverse events can be predicted using predictive AI models trained on ePRO and clinical data. These results will be shared for the first time at the 2025 ISOQOL conference.

### Conclusion

Our findings will improve the feasibility of ePRO monitoring during cancer care by increasing the clinical relevance of collected information by filtering out false positive alerts and adjusting for missing PRO timepoints.

## 302.2 Assessment of Treatment Response Through the Use of Personalized Endpoints: Using Artificial Intelligence to Assist Goal Attainment Scaling

Gunes Sevinc^1^, Katie Crespo^1^, Andrea Escoto^1^, Kari Pope^1^, Chere Chapman^1^, Susan E. Howlett^1^, Kenneth Rockwood^1^

^1^Ardea Outcomes, Halifax, Nova Scotia, Canada

*Journal of Patient-Reported Outcomes 2026*, **10(Suppl 1)**:302.2

### Aims

Goal attainment scaling (GAS) is a personalized outcome assessment that quantifies the impact of interventions on individualized goals. With the increasing focus on patient-centered drug development, regulators have recommended personalized clinical outcome assessments like GAS to evaluate treatment effects, though concerns about potential inconsistencies in scale development remain. Standardizing the GAS process through clinician training and utilizing artificial intelligence (AI) to complement formal training may help minimize inconsistencies and improve the robustness of goal scales. However, AI-generated outputs highly depend on the guiding questions, known as prompts, that are used. Here, we examined the role of AI in improving the psychometric properties of goal scales and the influence of prompt structure.

### Methods

We developed prompts based on OpenAI’s guidelines to guide ChatGPT (OpenAI, 2025) in evaluating the psychometric properties of goal scales using predefined quality criteria (n=20). To test these prompts, we created four goal scales with common errors, including overlapping attainment levels, vagueness, multidimensionality, and incorrect ordering of levels. We then evaluated ChatGPT’s responses using four prompting strategies: (i) simple batch prompting; (ii) batch prompting with context and rationale for each goal-scale criteria; (iii) incorporating chain-of-thought prompting into the previous prompt; and (iv) multi-turn prompting with context and rationale. We applied each prompting strategy to all four goal scales and replicated the evaluation process three times for each scale.

### Results

ChatGPT consistently detected multidimensionality with simple batch prompting but failed to identify the other common errors. Batch prompting with context and rationale consistently identified multidimensionality and vagueness in scales but did not reliably detect incorrect level ordering or overlap between attainment levels. On the other hand, with chain-of-thought and multi-turn prompting, ChatGPT consistently identified multidimensionality, overlap, and vagueness, but both failed to detect incorrectly ordered levels of attainment.

### Conclusion

ChatGPT’s ability to identify common errors improved when prompts included context and relevant examples. While some inconsistencies in error detection persisted, chain-of-thought and multi-turn prompting enabled AI to provide feedback that could enhance goal scales. These findings suggest that AI-assisted feedback may support GAS training and monitoring and, ultimately, may be utilized to strengthen the psychometric properties of individual goal scales.

**Table 1 (abstract 302.2):**
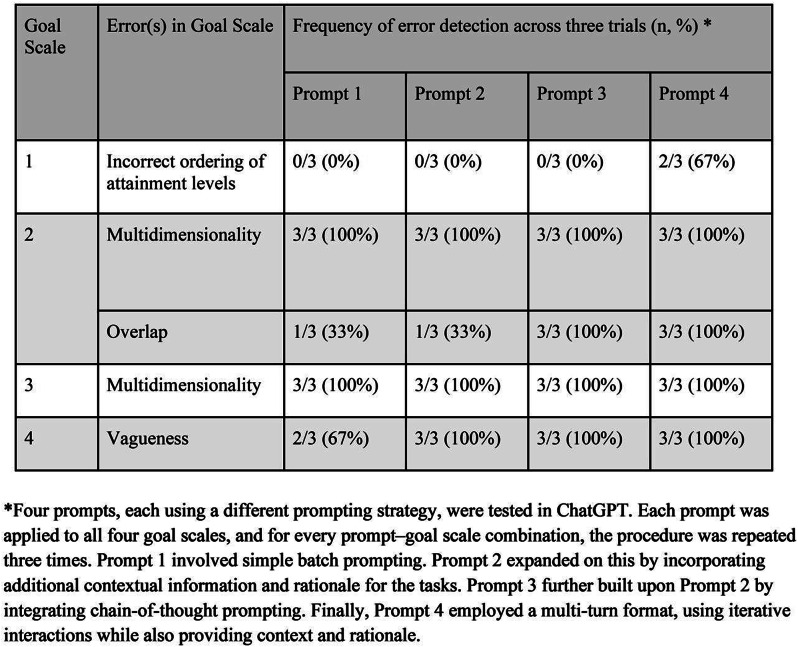
Overview of prompting strategies applied in ChatGPT for evaluating the psychometric properties of goal scales and error detection across three trials. *Four prompts, each using a different prompting strategy, were tested in ChatGPT. Each prompt was applied to all four goal scales, and for every prompt- goal scale combination, the procedure was repeated three times. Prompt 1 involved simple batch prompting. Prompt 2 expanded on this by incorporating additional contextual information and rationale for the tasks. Prompt 3 further built upon Prompt 2 by integrating chain-of-thought prompting. Finally, Prompt 4 employed a multi-turn format, using iterative interactions while also providing context and rationale

## 302.5 Patient perceptions toward the use of AI in clinical and research settings

Richard Skolasky^1^, Janiece Taylor^2^

^1^Johns Hopkins University, Baltimore, Maryland, USA, ^2^Johns Hopkins University School of Nursing, Baltimore, Maryland, USA

*Journal of Patient-Reported Outcomes 2026*, **10(Suppl 1)**:302.5

### Aims

This study aimed to explore the concerns of patients with chronic low back pain (cLBP) regarding the use of AI to administer patient-reported outcome measures (PROMs) in clinical and research settings.

### Methods

A mixed-methods design was employed (interviews and survey) for 45 cLBP patients (mean age 52.3 ± 11.7 years, 58% female). The Oswestry Disability Index assessed current back pain severity. The Back Pain Attitude Questionnaire and custom AI acceptability scales assessed beliefs about back pain and technology adoption. Thematic analysis identified themes from interviews, while regression models evaluated associations between psychosocial factors and AI acceptance.

### Results

Patients expressed optimism about using AI to administer PROMs, recognizing potential benefits but voicing reservations. Thirty-one noted that AI could streamline the process and reduce clinician workload (Participant: “If it saves time for both me and my doctor, I think it could be helpful, as long as it doesn’t miss anything important.”)Concerns about trust and accuracy were prominent. Twenty-eight doubted AI’s ability to fully capture the complexity of their pain (Participant: “A computer can’t understand what my bad days are like, or how it affects my mood and sleep. I worry it would just see numbers, not the whole story.”) The importance of human interaction was a recurring theme, with thirty-three emphasizing the value of empathy (Participant: “Sometimes just talking to my doctor about my pain makes me feel better. I’d miss that if it was just a computer asking questions.”)Data privacy and security also emerged as significant barriers, cited by twenty-four participants (Participant: “I’m not sure where my answers would go or who might see them. I’d need to know my information is safe before I’d feel comfortable.”)Notably, patients with higher health literacy and more positive attitudes toward PROMs were more open to AI.

### Conclusion

While AI-administered PROMs offer efficiency gains, cLBP patients prioritize relational aspects of care and data security. Successful implementation requires addressing misconceptions through education, transparent AI decision-making processes, and hybrid models blending AI automation with clinician oversight. Future research should validate AI systems against gold-standard clinician assessments to build trust and refine patient-centered design.

## 303.4 Developing a conceptual framework of health-related quality of life of informal carers in Amyotrophic Lateral Sclerosis (ALS)

Rosie Bamber^1^, Jill Carlton^1^, Christopher McDermott^1^, Theocharis Stavroulakis^1^

^1^University of Sheffield, Sheffield, UK

*Journal of Patient-Reported Outcomes 2026*, **10(Suppl 1)**:303.4

### Aims

ALS is progressive neurodegenerative life-limiting condition with frequently swift progression. Informal carers of people with ALS provide extensive care and support which impacts their own health-related quality of life (HRQoL). Existing studies examining the impact vary in their approach. Different person-reported outcome measures (PROMs) have been used to assess their HRQoL (or an aspect thereof). Such inconsistency means existing literature may under-estimate the impact of caring in HRQoL of informal carers. The aim of this study was to develop a comprehensive conceptual framework for HRQoL for ALS carers.

### Methods

This study comprised two components. Firstly, a scoping review was undertaken in March 2024 where key databases (Medline, Embase and Cinahl) were searched to identify primary studies investigating HRQoL in ALS carers. This included studies (qualitative, quantitative or mixed-methods) that had used multi-item PROMs or HRQoL assessment. The identified PROMs were examined to extract themes and sub-themes of HRQoL, which were incorporated into an existing framework of HRQoL (QuALS). QuALs conceptualises HRQoL for people living with ALS and consists of three domains (physical, psychological and social). The modified framework (Carer-QuALS) was then reviewed by ALS carers through online collaborative engagement workshops to ratify the framework.

### Results

715 articles were identified, with 82 articles examined. 44 PROMs were identified that met the inclusion criteria. Data extraction identified a new subtheme (‘physical caring activities’), with 7 subthemes not supported by the literature. The draft Carer-QuALS framework (consisting of seven themes and 43 subthemes) was discussed with carers. Based on feedback, one new subtheme (‘privacy’) was added, six subthemes were removed, and one existing subtheme—absent from the review literature—was retained. The final framework includes 38 subthemes: 9 physical, 6 social, and 23 psychological.

### Conclusion

This study has produced a comprehensive conceptual framework of HRQoL of informal carers of people living with ALS. It can be used to demonstrate the multidimensional impact that ALS caregiving can have on HRQoL. The framework can be used by researchers, clinicians, and patient advocacy groups for multiple purposes (e.g., support PROM selection to measure HRQoL, guide future PROM development, and facilitate discussions between informal carers and clinicians).

## 303.5 Minimally important differences (MIDs) and minimally important changes (MICs) were determined for the Vestibular Schwannoma Quality of Life (VSQOL) Index

Kathleen Yost^1^, Christine M. Lohse^1^, Michael J. Link^1^, Matthew L. Carlson^1^

^1^Mayo Clinic, Rochester, Minnesota, USA

*Journal of Patient-Reported Outcomes 2026*, **10(Suppl 1)**:303.5

### Aims

Vestibular schwannoma (VS) is a noncancerous tumor that develops from the vestibulocochlear nerve. We previously developed and validated the 40-item VS Quality of Life (VSQOL) Index to evaluate six domains of QOL impacted by VS: Hearing, Dizziness, Pain, Problems with Face/Eyes, General Well-being, and Cognition. Scores range from 0–100 with higher scores indicating better QOL. Our objective was to derive minimally important differences (MIDs) and minimally important changes (MICs) for the six QOL domain and Global QOL scores.

### Methods

The VSQOL and several anchors were administered at initial (T1) and 12-month follow-up (T2) timepoints to a registry of patients diagnosed with VS. Several 5- and 10-point single-item anchors were used to form clinically relevant groups (none, mild, moderate, severe, extreme) using cross-sectional data for the MID analyses, and to create somewhat worse/somewhat better groups using longitudinal data for the MIC analyses. Anchor-based estimates were not reported (NR) if the correlation between the anchor and VSQOL score was <0.3.

### Results

Questionnaires were completed by N=1,050 at T1 and N=644 at T2. Most participants were women, mean age at diagnosis was 53 years, and microsurgery was the predominant treatment (Table 1). Median (interquartile range (IQR)) of the anchor-based MID estimates were as follows: Hearing, 17 (12.5-21); Dizziness, 21 (18-23); Pain, 19.5 (15-23.5); Face/Eyes, 18 (17-28); General Well-being, 19.5 (14.5-23.5); Cognition, 23.5 (18.5-28.5); and Global QOL, 12 (8-16). Effect sizes for 39% of MID estimates were >0.8. There were too few estimates of the MIC to report median and IQR for individual domains. MIC estimates were: Hearing, (NR because of a lack of anchors with sufficient correlation); Dizziness, 7.2 and 8.3; Pain, 8.3 and 8.5; Face/Eyes, 7.0 and 7.4; General Well-being, 2.1 and 8.0; Cognition, 12.9 and 15.0; and Global QOL, median 5.2 (IQR 3.7-5.6). Effect sizes for only 5% of the MIC estimates were >0.8.

### Conclusion

MID estimates were often associated with large effect sizes; thus, they may be more accurately interpreted as “clinically” rather than “minimally” important differences. MIDs and MICs can facilitate interpretation of group mean differences and within-patient change scores, respectively, for the VSQOL Index.

**Table 1 (abstract 303.5):**
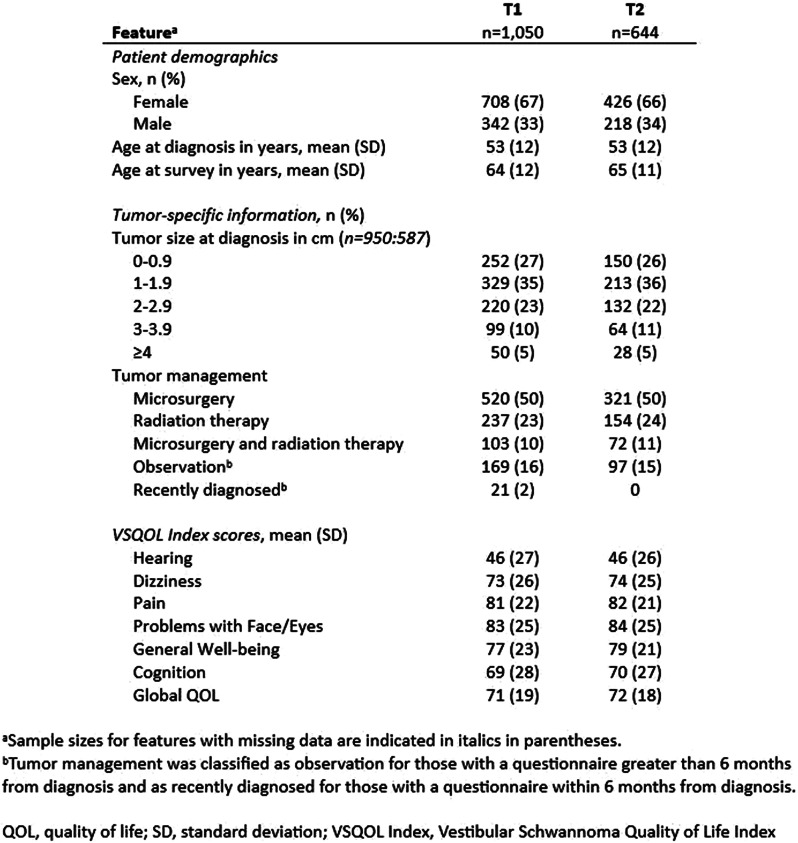
Summary of patient demographics, tumor-specific features, and VSQOL Index scores for the initial (T1) and 12-month (T2) assessments. ^a^ Sample sizes for features with missing data are indicated in italics in parentheses. ^b^ Tumor management was classified as observation for those with a questionnaire greater than 6 months from diagnosis and as recently diagnosed for those with a questionnaire within 6 months from diagnosis. QOL, quality of life; SD, standard deviation; VSQOL Index, Vestibular Schwannoma Quality of Life Index

## 303.2 The trajectory of depression-related symptom clusters following stroke

Qi Zhang^1^

^1^Sun Yat-Sen University, Guangzhou, China

*Journal of Patient-Reported Outcomes 2026*, **10(Suppl 1)**:303.2

### Aims

Post-stroke depression (PSD) frequently co-occurs with other symptoms, forming complex symptom clusters. While research has identified associations between PSD and specific symptoms, the intricate interrelationships within these clusters remain largely unexplored. This study aimed to examine the longitudinal trajectory of these symptoms, classify distinct symptom cluster profiles, uncover symptom interconnections, identify core symptoms, and explore associated factors.

### Methods

A longitudinal study with a 6-month follow-up was conducted in two large urban hospitals in Guangzhou, China. A total of 195 stroke survivors meeting the inclusion criteria (e.g., age ≥ 18 years, stable vital signs, no psychotropic medications or severe cognitive impairment) were enrolled. Demographic and clinical data were collected, and symptoms were assessed using the Zung Self-Rating Depression Scale (SDS), Zung Self-Rating Anxiety Scale (SAS), 9-item Fatigue Severity Scale (FSS), Pittsburgh Sleep Quality Index (PSQI), and Stroke Stigma Scale (SSS) at baseline (T1), one month (T2), three months (T3), and six months (T4) post-stroke. Network analysis was used to examine symptom relationships and identify core symptoms, while latent profile analysis classified distinct symptom clusters.

### Results

Network analysis revealed positive correlations among the five symptoms across four time points. “Anxiety” was the most central symptom within the symptom network at T1 (Strength = 2.134, Closeness = 0.127, EI = 2.134), while “depression” held the central position at T2 (Strength = 2.595, Closeness = 0.157, EI = 2.595), T3 (Strength = 2.689, Closeness = 0.161, EI = 2.689), and T4 (Strength = 2.789, Closeness = 0.172, EI = 2.789). At T1, four clusters emerged: moderately affected, anxiety-insomnia, resilient, and severely affected. At T2, T3, and T4, a two-cluster solution was identified: resilient and symptomatic. Gender, average monthly income, primary caregiver type, and stroke severity associated with symptom cluster membership.

### Conclusion

This study revealed a dynamic interplay of psychological symptoms following stroke. Depression, anxiety, fatigue, sleep disturbance, and stigma exhibited a U-shaped trajectory, initially improving but subsequently worsening. Network analysis demonstrated a stable symptom cluster structure, with depression and anxiety as core components. Symptom cluster profiles varied across time points, influenced by factors including gender, income, caregiving status, and stroke severity.

## 303.3 Major impact of Multiple Sclerosis on family members and partners: evidence using the Family Reported Outcome Measure (FROM-16)

Rubina Shah^1^, Sam Salek^2^, Faraz M. Ali^1^, Kennedy Otwombe^3^, Stuart J. Nixon^4^, Marie-Elaine Nixon^4^, Gillian Ingram^5^, John R. Ingram^1^, Andrew Y. Finlay^1^

^1^Division of Infection and Immunity, School of Medicine, Cardiff University, Cardiff, UK, ^2^School of Life & Medical Sciences, University of Hertfordshire, Hatfield, UK, ^3^Statistics and Data Management Centre, Perinatal HIV Research Unit, Chris Hani Baragwanath Academic Hospital, University of the Witwatersrand, Johannesburg, South Africa, ^4^MS Society, Cardiff, UK, ^5^Department of Neurology, Swansea Bay University Health Board, Swansea, UK

*Journal of Patient-Reported Outcomes 2026*, **10(Suppl 1)**:303.3

### Aims

The physical, social, and psychological well-being of family members and partners of people with multiple sclerosis (MS) is poorly understood. This study aims to measure the impact of a person’s MS on the QoL of their family members/partners using the extensively validated generic family-specific QoL instrument, the Family Reported Outcome Measure (FROM-16).

### Methods

This online cross-sectional study recruited family members/partners of people with MS (pwMS) through the MS Trust, MS Society and Healthwise Wales and Social Services Departments in Wales. The pwMS completed basic information about themselves and gave permission for their family member/partner to contribute to the study. The family member/partner of pwMS completed basic demographic questions and FROM-16. Data analysis included descriptive and other statistics, including non-parametric Mann-Whitney U-test and Kruskal-Wallis tests for group comparisons. Multiple linear regression was used to investigate relationships between dependent and independent variables. FROM-16 descriptive score banding was used to describe the severity of the impact of a person’s MS on family members/partners.

### Results

219 family members/partners (mean age=49.3 years, SD=13.7; females=55.3%) of pwMS (mean age=50.1, SD=12.5; females=56.6%) completed the FROM-16. The FROM-16 mean total score was 16.9 (SD=7.8), indicating “a very large effect” on family members’ QoL. Of the individual FROM-16 items, “being worried” had the highest mean score of 1.49 (max=2), followed by “feeling frustrated” (mean=1.37, max=2), “effect on family activities” (mean=1.37, max=2) and “feeling sad” (mean=1.35, max=2). The increasing age of pwMS, being a male pwMS and being a female carer were significant predictors of family impact. 50.7% of family members had FROM-16 scores ≥17, indicating “a very large effect” to “extremely large effect” on the QoL of these family members. Spouses/partners (170/219) of pwMS reported a greater impact on their sex life compared to other relationships (p=0.001).

### Conclusion

MS substantially impacts the QoL of family members/partners of pwMS. This indicates a need to assess this impact routinely to support family members appropriately. FROM-16 could be used to measure MS family impact in routine practice and to include this impact in health economic appraisal and therapeutic clinical trials.

## 303.1 Understanding the experience of people living with amyotrophic lateral sclerosis: a qualitative study and conceptual model informed by patients, caregivers, and clinicians

William Nowell^1^, Nadine McGale^2^, Oren Levy^1^, Sarah Wilding^2^, Phoebe Heinrich^2^, Nick Patel^1^, Jinsy Andrews^3^, Diana Rofail^1^

^1^Regeneron Pharmaceuticals, Inc., Tarrytown, New York, USA, ^2^Modus Outcomes, Cambridge, Massachusetts, USA, ^3^Department of Neurology, Colombia University, New York, USA

*Journal of Patient-Reported Outcomes 2026*, **10(Suppl 1)**:303.1

### Aims

This qualitative study aimed to (1) explore the experience of living with amyotrophic lateral sclerosis (ALS) through interviews with patients, caregivers, and clinicians, and (2) develop a conceptual model for this rare, progressive disease.

### Methods

Sixty-minute, semi-structured, concept elicitation interviews were conducted (Jan–Sep 2024) with people living with ALS (pALS), caregivers, and clinicians. Qualitative data from each participant type were analyzed separately to expand and refine a preliminary conceptualization of the experience of living with ALS. Concept saturation was assessed every 5–6 interviews, and the preliminary disease conceptual model was updated.

### Results

Thirty-one interviews were conducted with pALS: seven (23%) had SOD1-ALS, the mean age was 42.4 years (SD=11.5), 81% were female, 84% were White, and 61% were living with their spouse/significant other (Table). Mean time since ALS diagnosis was 4.6 years (SD=4.2); mean normed score on the patient-reported Rasch Overall ALS Disability Scale (ROADS) was 76 (SD=17.16; range=36–105), higher scores indicate better functioning. A comprehensive conceptual model of the patient experience of living with ALS was developed. Signs, symptoms, and functions reported by pALS included neuromuscular, bulbar, speech, neurocognitive, fatigue, respiration, autonomic, pain, and a range of physical functioning issues. Impacts on a wide range of activities and psychosocial interactions, as well as management strategies employed by pALS were also reported. Twenty interviews with caregivers and 10 with clinicians were also conducted. Caregivers identified additional signs such as drooling/excess salivation, and impacts related to ALS management (i.e., need for writing aids, vehicle modifications). Clinicians also considered loss of speech and neurocognitive signs (executive functioning, behavior change, personality change) as clinical manifestations of ALS. Concept saturation was reached, and a consolidated, comprehensive conceptual model of the experience of living with ALS was developed (Figure).

### Conclusion

This research provides a holistic understanding of the experience of living with ALS as reported by pALS, caregivers, and clinicians. The conceptual model is the first based on in-depth concept elicitation interviews across these perspectives and highlights the range of signs, symptoms, and impacts that pALS experience in their lives, emphasizing the serious humanistic impact and high unmet needs of ALS.

**Table (abstract 303.1):**
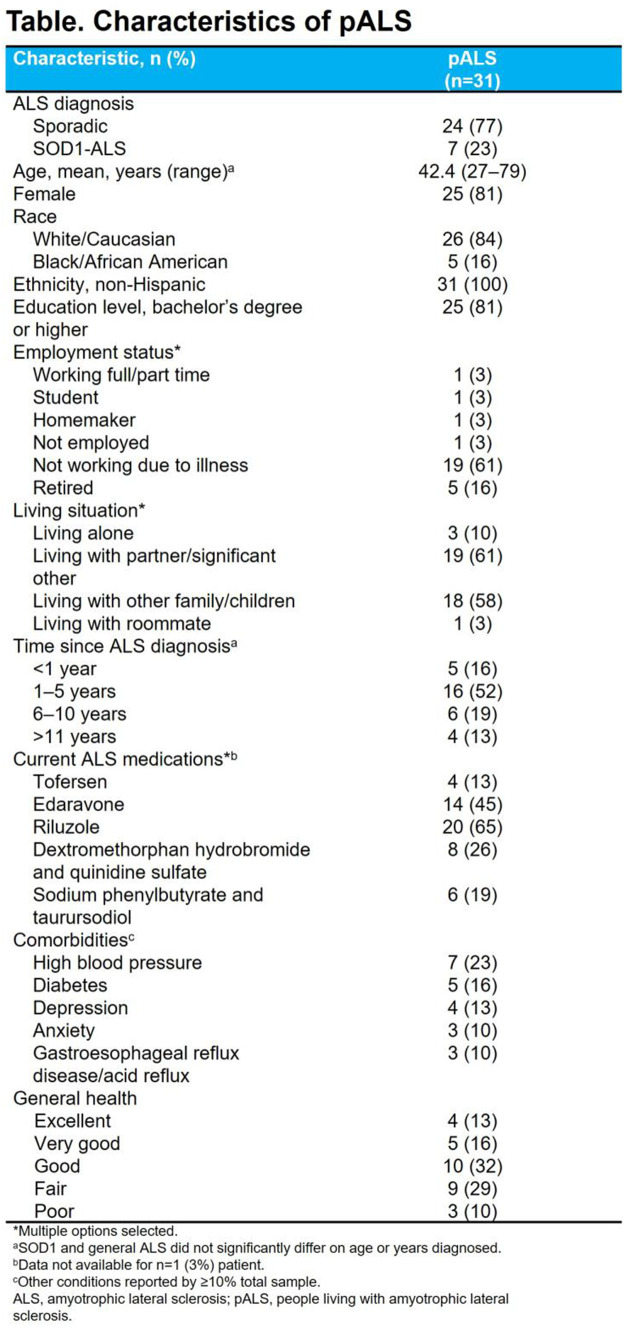
Characteristics of pALS

**Fig. 1 (abstract 303.3):**
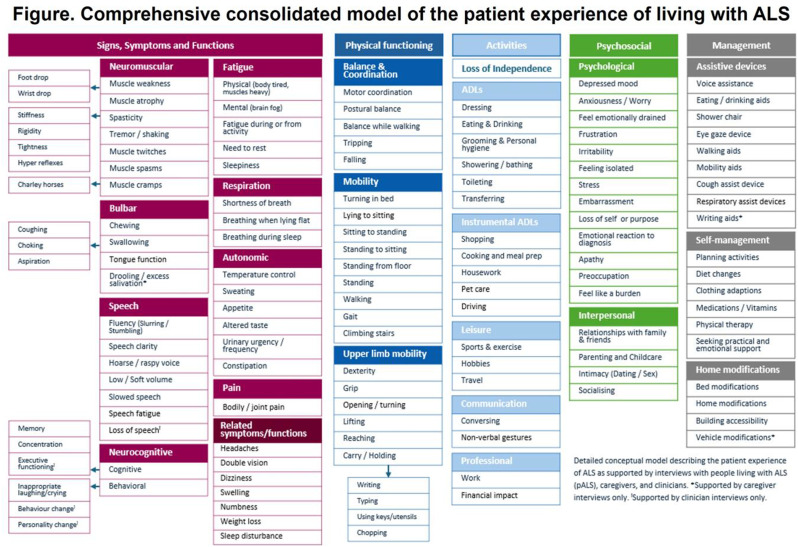
Comprehensive consolidated model of the patient experience of living with ALS

## 304.5 Understanding response rates of electronic PROMs in clinical care: trends and characteristics

Margot de Jong^1^, Sabine Super^1^, Anouk Groenewegen^2^, Harm Gijsbers^3^, Maud van Muilekom^2^, Hedy van Oers^2^, Lotte Haverman^2^, Nynke Venema-Taat^1^

^1^Amsterdam Public Health, Digital Health, Amsterdam, Netherlands, ^2^Department of Child and Adolescent Psychiatry and Psychosocial Care, Amsterdam UMC, University of Amsterdam, Emma Children’s Hospital, Amsterdam, The Netherlands, ^3^Amsterdam UMC location University of Amsterdam, Department of Medical Informatics, eHealth Living & Learning Lab Amsterdam, Amsterdam, The Netherlands

*Journal of Patient-Reported Outcomes 2026*, **10(Suppl 1)**:304.5

### Aims

Using Patient Reported Outcome Measures (PROMs) on an individual level in clinical care have been shown to enhance patient-clinician communication, improve quality of life and even increase survival. In the Amsterdam UMC, PROMs are implemented in clinical care via Epic (Electronic Health Record) since 2019, guided by the PROM Expertise Center (PEC). An essential element of the PROM implementation process guided by PEC, is an annual evaluation with healthcare teams. A common concern in these evaluations is the low response rate of completed PROMs by patients. Low response rates are worrisome, as these limit the ability to monitor patient-reported outcomes and to incorporate patient perspectives into clinical decision making, and can bias aggregated data. The primary aim of this study is to gain insight into the response rates of over 80 healthcare teams currently using PROMs in clinical care. Second, we aim to identify characteristics that are associated with response rates, on a patient, system, healthcare team and PROM level.

### Methods

PROM response rates are calculated as the percentage of completed PROMs relative to those sent. The response rates will be derived per healthcare team and presented over time, using Epic query/reporting tools. A logistic regression analysis will be performed to study associations between response rates and characteristics including age, gender and condition of the patients; patients access to complete PROMs via Epic; specialisms within and the size of healthcare teams; the number, type (e.g. generic or specific), and frequency of PROMs sent.

### Results

Response rates from over 80 healthcare teams using PROMs between 2019 to March 2025 will be determined. Most healthcare teams using PROMs in the Amsterdam UMC fall within the departments of Internal Medicine (including HIV and pulmonology), Woman/Child, and Neurology/Head-Neck (including multiple sclerosis and head-neck oncology). Results of the analyses will be presented at the conference.

### Conclusion

With the outcomes of this study, we will have more insight into ‘real-world’ response rates of electronic PROMs in an academic hospital setting. Together with the identified characteristics related to the response rates, this will enable the development of implementation strategies to enhance the completion of PROMs in clinical care.

## 304.1 Establishing optimal Cut-off Points for Remote Monitoring of Cancer Symptoms via ePROs in China’s Primary Health Care Settings

Min Li^1^, Qiuling Shi^1^, Xiaojun Dai^2^, Dai Wei^3^

^1^Chongqing Medical University, Chongqing, China, ^2^Yangzhou Hospital of Traditional Chinese Medicine, Jiangsu, China, ^3^Sichuan Clinical Research Center for Cancer, Chengdu, China

*Journal of Patient-Reported Outcomes 2026*, **10(Suppl 1)**:304.1

### Aims

Electronic patient-reported outcomes (ePROs) have been widely adopted to improve symptom management in oncology. The community-dwelling patients with cancer are typically in the symptom maintenance phase after completing active hospital-based treatment. During this phase, symptom scores tend to be lower, and cut-off points (CPs) for triggering alerts may differ from those used in active treatment (CPs, 4,6). This study aimed to establish optimal cut-off points for mild, moderate, and severe symptoms to support remote symptom monitoring via ePROs in the China’s Primary Health Care Settings.

### Methods

Data were extracted from a prospective cohort study of community-dwelling patients during the cancer symptom maintenance phase. Symptoms were assessed using the MD Anderson Symptom Inventory–Traditional Chinese Medicine module (MDASI-TCM). The quality of life was assessed using the EuroQol 5-Dimension 5-Level Questionnaire (EQ-5D-5L). For the cutoff point analyses, the severity was categorized as “none/mild” (0 ≤ none/mild < lower cutoff point), “moderate” (lower cutoff point ≤ moderate<upper cutoff point), and “severe” (upper cut off point≤severe≤10 points). we used the EQ-5D-VAS score at baseline as the anchor. One-way ANOVA was used to identify the optimal cutoff point of the symptom. Bootstrap resampling with 2000 samples was used to verify whether the chosen cutoff points were robust.

### Results

In total, 351 community-dwelling patients with cancer during the symptom maintenance phase were recruited. The scores of alerting symptoms were: fatigue (1.9±2.23), sleep disturbance (1.87±2.36), lack of appetite (1.75±2.26), dry mouth (1.62±2.26), and pain (1.25±2.16).We examined 36 different sets of cutoff points, and the optimal cutoff point was selected based on the largest F-value. The optimal CPs (lower, upper) for alerting symptoms were: pain (3, 5; F = 38.94), fatigue (2, 5; F = 29.85), sleep disturbance (3, 5; F = 36.08), lack of appetite (3, 6; F = 38.56), and dry mouth (2, 4; F = 38.56).

### Conclusion

Community-dwelling patients with cancer reported relatively low symptom scores, requiring distinct cut-off points from those used during active treatment. Adaptive adjustment of ePRO alert CPs is recommended to align with the needs of the target population and care setting.

**Table 1 (abstract 304.1):**
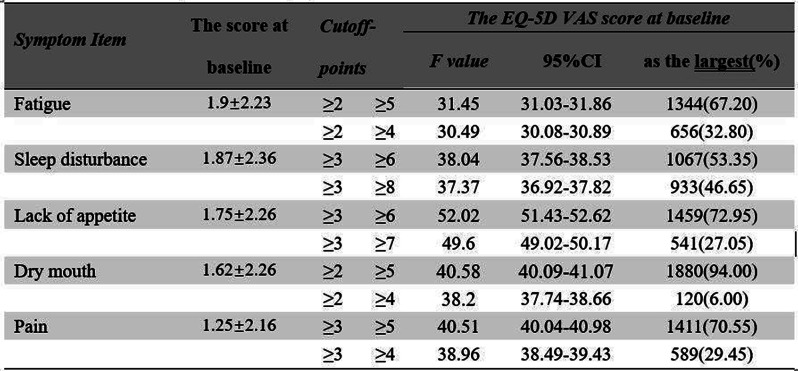
The cutoff-points of five symptoms

## 304.2 Defining Critical Symptom Profiles to Build ePRO-based Pathway for Patient Care During First Year After Chemoradiation and Immunotherapy for NSCLC

Xin Shelley Wang^1^, Shu-en Shen^1^, Ting Xu^1^, Rachel Maguire^1^, Diana Amaya^1^, Aileen Chen^1^, David Qian^1^, Steven Lin^1^, Anita Deswal^1^, Efstratios Koutroumpakis^1^, Anne Tsao^1^, Mehmet Altan^1^, Mei Chen^1^, Xiaodong Zhang^1^, Ruitao Lin^1^, Zhongxing Liao^1^

^1^MD Anderson Cancer Center, Houston, Texas, USA

*Journal of Patient-Reported Outcomes 2026*, **10(Suppl 1)**:304.2

### Aims

Patients with non-small cell lung cancer (NSCLC) may experience significant symptom burden relevant to toxicities during the first year caused by concurrent chemoradiation therapy (CRT) and immunotherapy. The goal of this study is to define clinically meaningful symptoms to trigger symptom intervention on a pathway with electronic patient-reported outcomes (ePROs) based patient care.

### Methods

Patients were consented to enroll on a prospective trial (NCT05010109) to perform longitudinal assessment of cardiac injury, cardiac fitness trajectory, and model-based treatment selection. We collected NCI-CTCAE rated cardiac and lung toxicities and examined mixed modeling of longitudinal profile of PROs on the lung module of the MD Anderson Symptom Inventory (MDASI-L) up to 12 months. Logistic regression models examined the association between the end of RT PRO severity levels and grade 2+ toxicities after therapy.

### Results

The peak symptom burden occurred at the end of CRT, followed by significant recovery within 6–8 weeks post-CRT, and a slow increase in severity up to 12 months post-RT (Fig 1). The seven symptoms rated as most severe persistently were pain, fatigue, sleep disturbance, shortness of breath, drowsy, difficulty swallowing, and coughing. During 12 months study, at least 25% of patients rated each symptom moderate to severe (5+ on 0–10 scale). Shortness of breath was greater for patients had grade 2+ cardiac AEs (P<.01). Coughing worsened overtime (P<.001) and was more severe for grade 2+ cardiac or pneumonitis AEs (P<.001) (Fig 2). By end of CRT, more severe shortness of breath OR=1.430 (1.020-2.005), interference to general activity OR=1.325 (1.001-1.754), interference to walking OR=1.401 (1.040-1.887) were predictive of any after-RT grade 2+ cardiac AEs (all P < 0.05).

### Conclusion

This is the first study evidenced a significant proportion of patients suffering from prolonged moderate to severe symptom burden during and post the CRT and immunotherapy for NSCLC. This is also the first study to identify a relationship between symptoms burden and grade 2+ AE development during the first year. These 7 identified most severe symptoms appear as clinically-meaningful PROs to be considered for inclusion in the ePRO patient care pathway in routine clinical care settings.

**Fig. 1 (abstract 304.2a):**
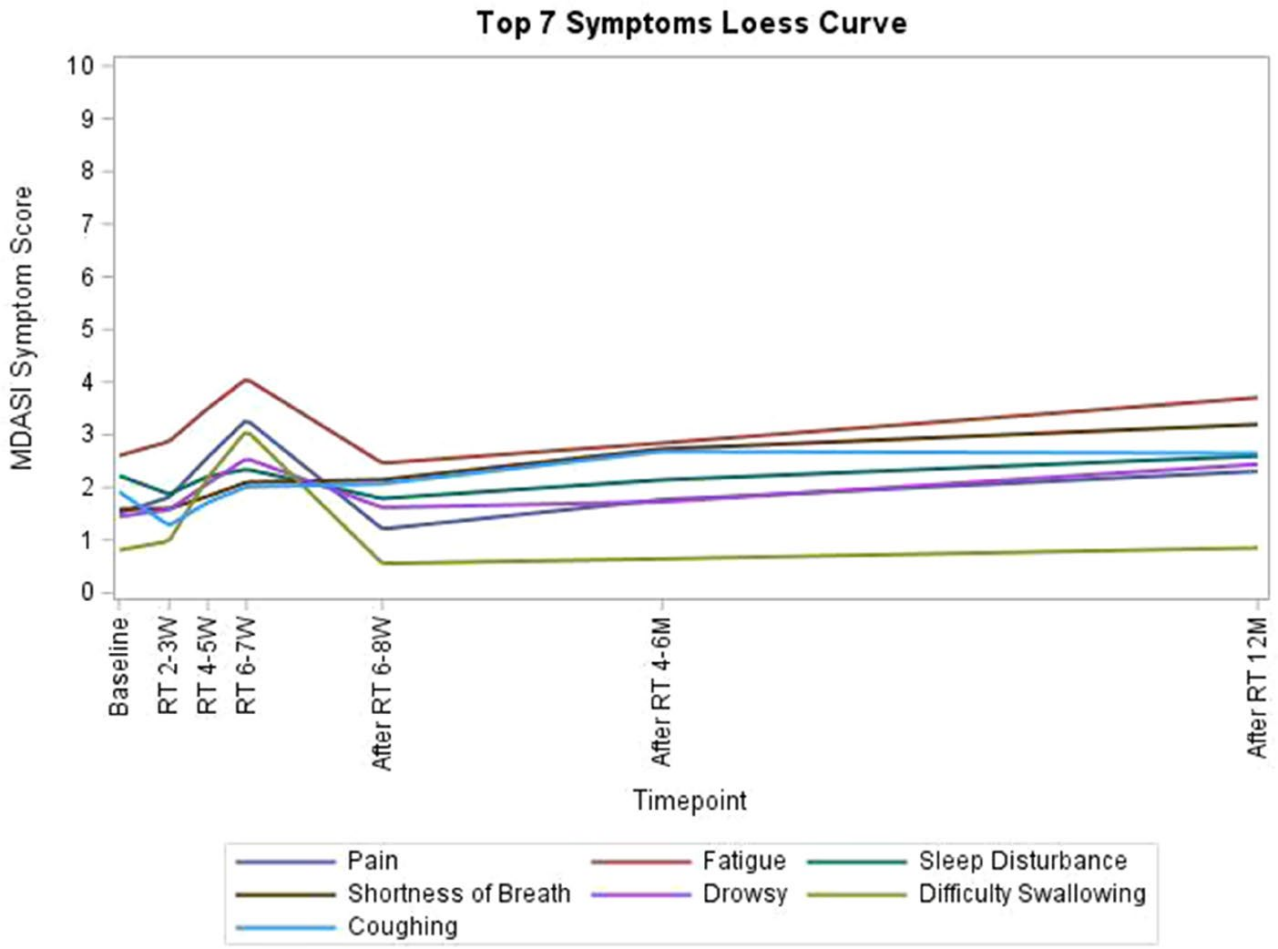
Defining Critical Symptom Profiles to Build ePRO-based Pathway for Patient Care During First Year After Chemoradiation and Immunotherapy for NSCLC

**Fig. 1 (abstract 304.2b):**
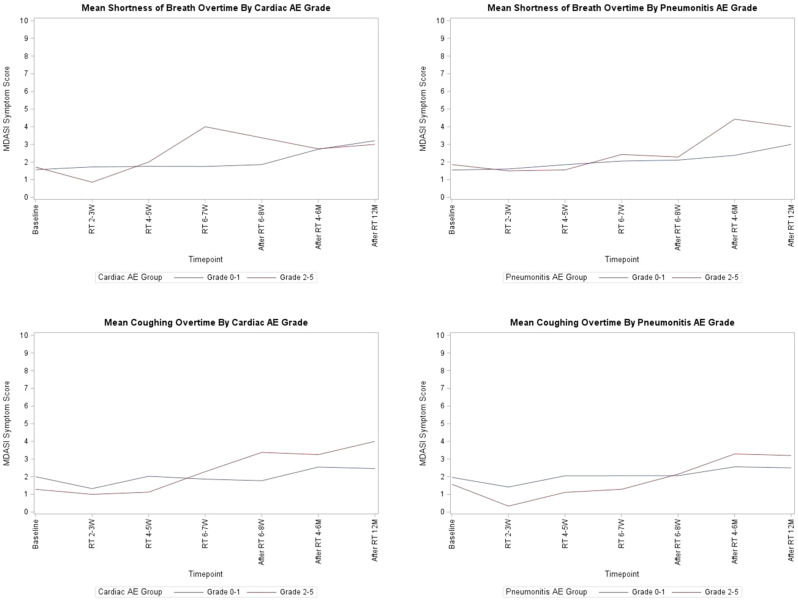
Defining Critical Symptom Profiles to Build ePRO-based Pathway for Patient Care During First Year After Chemoradiation and Immunotherapy for NSCLC

## 304.3 ePRO-MAI-CRC: AI-Powered Real-Time Symptom Monitoring and Proactive Triage for Home-Based Chemotherapy in Colorectal Cancer

Xu Wei^1^, Chenxi He^2^, Feng Gao^3^, Qiuling Shi^2^

^1^College of Public Health, Chongqing Medical University, Chongqing, China, ^2^Chongqing Medical University, Chongqing, China, ^3^Mianyang Central Hospital, Sichuan, China

*Journal of Patient-Reported Outcomes 2026*, **10(Suppl 1)**:304.3

### Aims

Colorectal cancer (CRC) patients undergoing chemotherapy face severe symptom burden, with 85% of exacerbations occurring at home. Current systems lack real-time monitoring between clinic visits, increasing risks of treatment delays. We developed ePRO-MAI-CRC, an AI-powered electronic Patient-Reported Outcome (ePRO) platform, to enable continuous remote symptom monitoring, automated risk alerts, and proactive clinical triage during home-based chemotherapy.

### Methods

A prospective cohort of 218 CRC patients (mean age 61.08±10.3; 79.4% stage III/IV) receiving chemotherapy was enrolled. Symptoms were assessed via a WeChat mini-program using the MDASI-GI scale at baseline, Days 1/3/5/7/10/14/21 per cycle. Eight symptoms (Pain, Fatigue, Nausea, etc.) triggered alerts at scores ≥7. Patients received automated reminders for incomplete surveys, while clinicians received real-time mobile alerts for actionable symptoms, prompting phone consultations and evidence-based interventions via predefined pathways.

### Results

The ePRO-MAI-CRC platform demonstrated high adherence (77.59%-93.43%) across six chemotherapy cycles. A total of 1,247 alerts were generated, peaking on Day 3 (281 alerts, 22.09%), reflecting a “single-peak” symptom trajectory, fatigue was the most frequent (22.69%) symptom. Across all six cycles, Cycle 1 exhibited the highest burden, with progressive reduction in subsequent cycles. In each cycle, symptom severity escalated post-chemotherapy initiation, peaked at Days 3–5, then subsided gradually. Fatigue consistently emerges as the most severe and persistent symptom, maintaining high severity scores (4.05±3.67) with minimal improvement, followed similar trajectories by Lack of Appetite (3.74±3.31) and Disturbed Sleep (3.26±2.85), highlighting unmet supportive care needs.

### Conclusion

ePRO-MAI-CRC bridges home-to-clinic monitoring gaps in CRC chemotherapy by identifying Days 3–5 as critical intervention windows. The AI-driven system’s real-time tracking and triage capabilities demonstrate potential to reduce treatment disruptions through early symptom control. Future integration of machine learning models will predict individual symptom escalation patterns, enabling personalized risk stratification and automated prioritization of high-need patients, ultimately advancing precision supportive care.

## 304.4 Dynamic Electronic Monitoring of Chemotherapy-Induced Neurotoxicity: Patterns and Clinical Impact in colorectal and breast Tumor Cohorts

Xu Wei^1^, Qiuling Shi^2^

^1^College of Public Health, Chongqing Medical University, Chongqing, China, ^2^Chongqing Medical University, Chongqing, China

*Journal of Patient-Reported Outcomes 2026*, **10(Suppl 1)**:304.4

### Aims

Chemotherapy-induced peripheral neuropathy (CIPN) affects 30-68% of oncology patients, causing dose-limiting sensory/motor deficits that severely compromise quality of life. This study deployed an electronic real-time monitoring platform to characterize dynamic CIPN trajectories across chemotherapy regimens.

### Methods

This prospective dual-center cohort study across two tertiary hospitals assessed chemotherapy-induced peripheral neuropathy (CIPN) using the Chinese version of the Total Neuropathy Assessment Scale (TNAS) via a WeChat mini-program at baseline and Days 1/3/7/10/14 per chemotherapy cycle. TNAS applied from MD Anderson Cancer Center. Longitudinal numbness trajectories were modeled through linear mixed-effects analyses with restricted maximum likelihood (REML) estimation, incorporating fixed effects for chemotherapy regimen cycle number (1-6), and regimen×time interactions, alongside random intercepts for subjects and random slopes for cycles. Means and Marginal means with 95% confidence intervals (CIs) were estimated at each time-regimen combination to quantify dynamic progression patterns.

### Results

The prospective cohort comprised 328 patients, including 218 colorectal cancer cases (oxaliplatin-based: 67.9%, n=148; non-platinum: 32.1%, n=70) and 108 breast cancer cases (taxane-based: 61.1%, n=66; anthracycline-based: 38.9%, n=42), with a mean age of 58.0 ± 10.8 years. The longitudinal trajectories of nine TNAS-assessed symptoms across six chemotherapy cycles. Among all symptoms, numbness exhibited the most severe and progressive worsening, escalating from baseline (1.2) to peak severity at Cycle 6 (1.5±1.2, 95%CI:1.1-1.7). We compares cancer-specific trajectories: breast cancer patients experienced cumulative symptom escalation, numbness increased to cycle 6, 1.8±1.2, 95%CI:1.1-2.1), whereas colorectal cancer patients on platinum-based regimens displayed a Stepwise increase change pattern, with maximum symptom burden at Cycle 2/3 (numbness means score 2.4±0.4, 95%CI:2.0-2.8) followed by gradual decline. We showed contrasts platinum vs. non-platinum regimens in colorectal cancer, revealing higher numbness scores in non-platinum groups with statistical significance (means change=0.8, p<0.001). Notably, in breast cancer cohorts, taxane-treated patients exhibited lower numbness severity versus non-taxane groups (means change=0.9, p<0.001), aligning with regimen-specific neurotoxicity profiles.

### Conclusion

This study delineates regimen-specific neurotoxicity trajectories that mandate tailored monitoring and intervention strategies. The heightened numbness burden in colorectal cancer patients receiving non-platinum regimens, particularly during cycles 2–3 when platinum-induced symptoms peak. For taxane-treated breast cancer patients, cumulative symptom escalation to cycle 6 demands sustained monitoring beyond treatment completion.

**Fig. 1 (abstract 304.4a):**
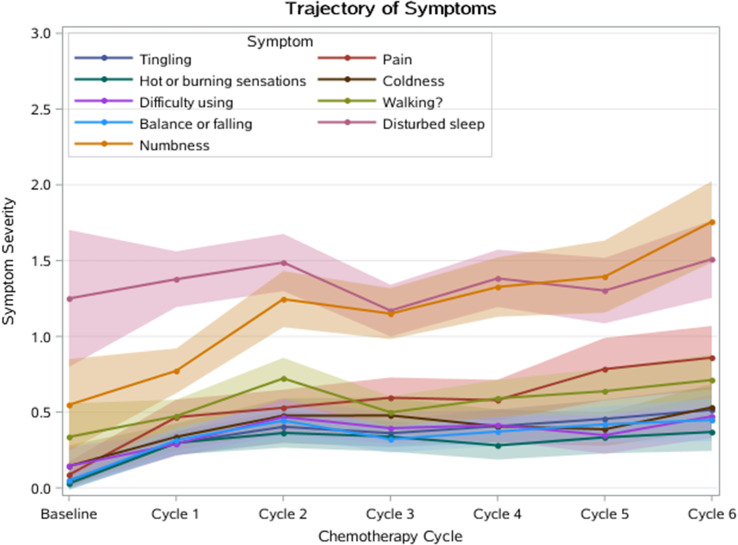
Dynamic Electronic Monitoring of Chemotherapy-Induced Neurotoxicity: Patterns and Clinical Impact in colorectal and breast Tumor Cohorts

**Fig. 1 (abstract 304.4b):**
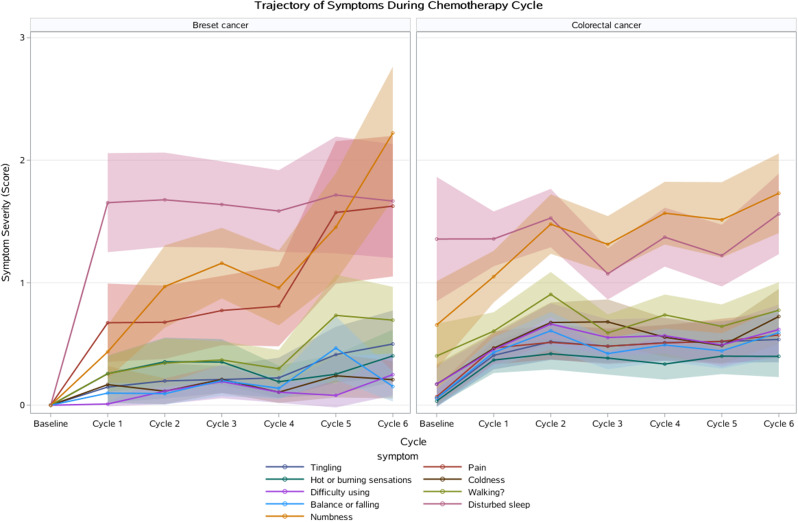
Dynamic Electronic Monitoring of Chemotherapy-Induced Neurotoxicity: Patterns and Clinical Impact in colorectal and breast Tumor Cohorts

**Fig. 1 (abstract 304.4c):**
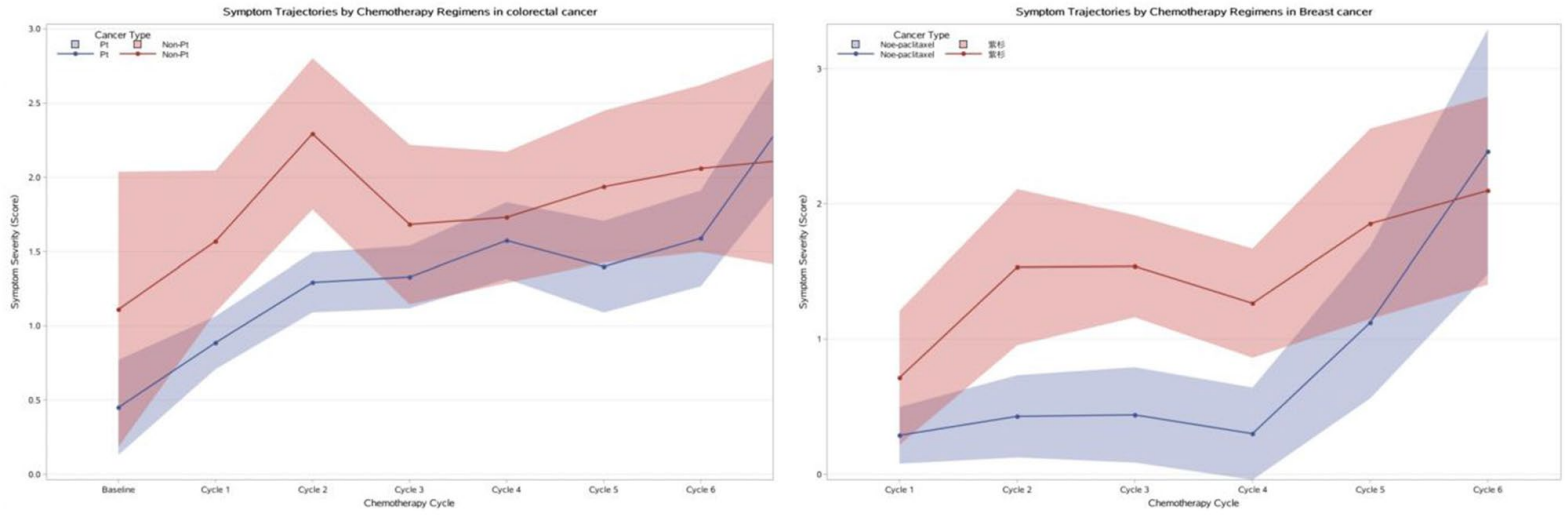
Dynamic Electronic Monitoring of Chemotherapy-Induced Neurotoxicity: Patterns and Clinical Impact in colorectal and breast Tumor Cohorts

## 305.5 Impact of Severe Neonatal Complications on Long-Term Health-Related Quality of Life in Very Preterm and/or Very Low Birth Weight Survivors: Evidence from Meta-Analysis

Corneliu Bolbocean^1^, Paula Dommelen^2^, Sylvia van der Pal^2^

^1^University of Oxford, Oxford, Arkansas, UK, ^2^TNO, Hague, Netherlands

*Journal of Patient-Reported Outcomes 2026*, **10(Suppl 1)**:305.5

### Aims

Understanding the impact of Bronchopulmonary Dysplasia (BPD), Intraventricular Hemorrhage (IVH), or Necrotizing Enterocolitis (NEC) on adult health-related quality of life (HRQoL) beyond the effect of prematurity itself is crucial for health economic evaluation and policy. This study evaluated the impact of BPD, IVH, NEC, and multiple birth status on preference-based HRQoL utility scores in adulthood among preterm survivors.

### Methods

Individual participant data were obtained from six prospective cohorts of individuals born VP/VLBW. The combined dataset included over 2300 adult VP/VLBW survivors with an age range of 18–29 years. The main exposures: BPD, IVH, NEC, and multiple birth status. Outcome measures included multi-attribute utility scores generated by the Health Utilities Index Mark 3, the Short Form 6D and optimal functioning within specific HRQoL domains. Data were analysed using generalised linear mixed models in a one-step approach using fixed-effects and random-effects models.

### Results

Preliminary results show that BPD, IVH, NEC, or multiple birth status were not associated with statistically significant differences in overall HUI3 or SF-6D utility scores compared to peers without these specific conditions. Specific domain effects were observed: BPD was associated with poorer physical functioning (SF-6D, 35y, p=0.05), NEC was linked to poorer speech (HUI3, 28y, p <0.001) and dexterity (HUI3, 28y, p=0.03). Multiple birth was associated with poorer cognitive functioning (HUI3, 19y, p=0.05). Analyses focusing on IVH (grade 3/4) revealed that severe IVH was associated with statistically significant decrements in HUI3 at 19 (-0.08, p=0.04) and 28 years (-0.15, p=0.03) and SF-6D at 35 years (-0.07, p=0.03).

### Conclusion

Severe IVH (grade 3/4) was associated with clinically significant decrements in HUI3 at 19 and 28 and in SF-6D at 35 years. Among very preterm or very low birth weight survivors followed into adulthood, BPD, NEC or multiple birth status did not consistently result in significantly lower overall preference-based HRQoL.

## 305.4 Are child patient reported-outcome measures used to inform assessments of health technologies and highly specialised technologies? A review of recent evidence in England

Donna Rowen^1^, Tessa Peasgood^1^, Anju Keetharuth^1^

^1^Sheffield Centre for Health and Related Research, University of Sheffield, Sheffield, UK

*Journal of Patient-Reported Outcomes 2026*, **10(Suppl 1)**:305.4

### Aims

Economic evaluation of health technologies and interventions require the generation of health benefits across the ages of patients affected. Where the health technology indication includes children, the economic evaluation should consider the health benefits of the technology for children. In recent years there has been increased interest in generic preference-weighted measures for children based on patient reported outcome measures for capturing health benefits for children. This systematic review aims to determine whether there has been recent use of child patient reported outcome measures to capture health benefits to children in health technology and highly specialised technology assessments.

### Methods

A systematic search of health technology and highly specialised technology assessments submitted to the National Institute of Health and Care Excellence (NICE), England, in the last 2 years where the indication included children was conducted in November 2024. Information was extracted on how health benefits were assessed, including details on the measure used, whether this was child- or proxy-reported, and any comments on the appropriateness of this from the evidence review group or from the committee making recommendations on the technology based on the clinical and cost-effectiveness evidence. Information was double extracted for 30% of assessments.

### Results

The search identified 30 assessments; 19 health technology and 11 highly specialised technology assessments. The results indicated that 6 assessments used a child measure to inform the calculation of health benefits to the child from treatment, other approaches included vignettes and adult measures. Criticisms of the approach used to capture health benefits of children were made in 6 assessments from the evidence review group reviewing the evidence and one assessment from the committee making recommendations on the technology.

### Conclusion

Child-appropriate measures are not (yet) commonly used to capture health benefits for children in health technology assessments. In general, this means that either: 1) health benefits to children are not included at all in the assessments; or 2) that vignettes are used (bespoke descriptions of health not based on a patient reported outcome measure); or 3) adult responses to adult patient reported outcome measures are assumed to be appropriate to indicate health benefits for children.

## 305.2 Have relative preferences for different dimensions of health changed over time? Evidence from the UK using EQ-5D

Donna Rowen^1^, Jill Carlton^1^, Nathan Bray^2^, Louise Longworth^3^, David Meads^4^, Clara Mukuria^1^, Ciaran O’Neill^5^, Yemi Oluboyede^6^, Yaling Yang^7^

^1^Sheffield Centre for Health and Related Research, University of Sheffield, Sheffield, UK, ^2^School of Health Sciences, Bangor University, Bangor, UK, ^3^Arrow Health Economics, London, UK, ^4^Academic Unit of Health Economics, University of Leeds, Leeds, UK, ^5^Centre for Public Health, Queen’s University; National University of Ireland, Belfast, UK, ^6^Putnam Associates, Newcastle, UK, ^7^University of Oxford, Oxford, UK

*Journal of Patient-Reported Outcomes 2026*, **10(Suppl 1)**:305.2

### Aims

The economic evaluation of treatments involves assessments of both costs and benefits of different treatments. These health benefits are typically measured using patient reported outcome measures (PROs) combined with utility values to generate quality adjusted life years (QALYs). Utility values are generated using preference elicitation methods with the public and reflect preferences for different health states described by PROs. EQ-5D is commonly used in economic evaluation. EQ-5D has five dimensions: mobility, self-care, usual activities, pain/discomfort and anxiety/depression. EQ-5D-3L has three severity levels in each dimension while EQ-5D-5L has five. In the UK, EQ-5D-3L preference weights were derived in 1993 and in 2023 EQ-5D-5L was valued but results are not yet available. This study explores how preferences have changed over time for EQ-5D dimensions using UK valuation study evidence.

### Methods

Both sets of preference weights were elicited from a representative sample of the UK public using the time trade-off technique (TTO). The two sets of preference weights were compared by assessing methodological and sample differences, utility value range, and ordering of impact of dimensions based on which dimensions were most impactful/valued worst.

### Results

Methodological differences exist across the two studies, including different TTO protocols particularly for how states worse than dead were valued, different modes of administration of interviews, and different sample sizes and composition. Recent preferences indicate that pain/discomfort has the largest impact and is valued as the worst dimension. Anxiety/depression has the second largest impact, followed by mobility and usual activities, and self-care has the smallest impact. In contrast, the 1993 preferences indicated the ordering of impact as pain/discomfort, mobility, anxiety/depression, self-care, followed by usual activities. Recent preferences indicate that a lower proportion of health states are considered as being worse than being dead.

### Conclusion

Relative preferences of the UK public for different dimensions of health have changed over time, though methodological differences including advances in preference elicitation methods might have impacted results. Greater relative importance is now given to mental health, and lower relative importance to self-care. The dimension with most impact remains pain/discomfort. Use of recent preference information is recommended since this most accurately captures current population preferences.

## 305.3 Assessing Health State Utilities Across Different Severity Levels of Friedreich Ataxia by the General Population

Kristen Deger^1^, Sheng-Han Kuo^2^, Walter Morris^3^, Louis S. Matza^1^, Richard Lawson^4^, Peter Pemberton-Ross^5^, Michael Urbich^5^, Cuixia Tian^6^

^1^Evidera (Part of Thermo Fisher Scientific), Bethesda, Maryland, USA, ^2^Columbia University Medical Center, New York, New York, USA, ^3^Evidera (Part of Thermo Fisher Scientific), London, UK, ^4^Biogen Inc., Cambridge, Massachusetts, USA, ^5^Biogen International GmbH, Baar, Zug, Switzerland, ^6^Cincinnati Children’s Hospital Medical Center & University of Cincinnati, College of Medicine, Cincinnati, USA

*Journal of Patient-Reported Outcomes 2026*, **10(Suppl 1)**:305.3

### Aims

Friedreich ataxia (FA) is a rare, progressive, inherited, neurodegenerative movement disorder. Common symptoms include gait ataxia, clumsiness, loss of fine dexterity, cardiomyopathy, fatigue, and slurred speech. Health-related quality of life data are limited in FA, and it remains unclear whether such data correlate with clinical measures of disease severity. This study aimed to estimate health state utilities associated with severity levels of FA.

### Methods

Five health state vignettes were developed to represent ranges on the modified Friedreich’s Ataxia Rating Scale (mFARS). The mFARS measures neurological severity of FA, with scores ranging from 0-93; higher scores indicate more severe FA. Data from 810 patients in the Friedreich’s Ataxia Clinical Outcome Measures Study (FACOMS) registry were analyzed to link patients’ mFARS scores with their scores on the Friedreich’s Ataxia Activities of Daily Living (FA-ADL) measure. The vignette content was developed based on literature review, input from clinicians, and the most frequently observed FA-ADL item scores corresponding to each mFARS range. The vignettes described the disease’s impact on mobility (e.g., walking and falling), daily living (e.g., dressing, speaking and bladder control), symptoms, quality of life, and management of condition. The health states were valued in time trade-off interviews to generate utilities that are anchored to 1 (full health) and 0 (equivalent to dead). None of the participants were diagnosed with FA.

### Results

A total of 223 participants completed interviews (42.6% male, mean age = 47.1; 114 from London, 109 from Edinburgh). In a ranking task, all participants ranked the health states in order of increasing severity. Mean (SD) utilities were 0.85 (0.17) for mFARS 0-19; 0.76 (0.27) for mFARS 20-39; 0.60 (0.41) for mFARS 40-59; 0.25 (0.57) for mFARS 60-79; and -0.18 (0.63) for mFARS 80-93.

### Conclusion

The health state utilities followed expected patterns with lower scores corresponding to worse clinical measures. A decline in utility was observed when the health states described increased mobility restrictions, increased discomfort, and increased dependence on others. The most severe health state, which depicted full-time wheelchair use and no independence had a negative utility value which highlighted the significant impact of FA on quality of life.

## 305.1 Collaborative involvement in valuing health-related quality of life is underdeveloped: Novel insights and emerging recommendations

Philip Powell^1^, Victoria Gale^1^, Gurdas Singh^2^, Anthea Sutton^1^, Janine Verstraete^3^, Nancy Devlin^4^, Michael Herdman^5^, Simone Schieskow^6^, Jill Carlton^1^

^1^University of Sheffield, Sheffield, UK, ^2^Gurdas Singh Consulting, London, UK, ^3^University of Cape Town, Cape Town, South Africa, ^4^University of Melbourne, Melbourne, Australia, ^5^National University of Singapore, Queenstown, Singapore, ^6^Bielefeld University, Bielefeld, Germany

*Journal of Patient-Reported Outcomes 2026*, **10(Suppl 1)**:305.1

### Aims

In most areas of health-related quality of life (HRQoL) research, collaborating with a range of invested parties – beyond the research team – is an established practice. However, the extent to which such collaboration is present when valuing HRQoL for use in cost-effectiveness analyses is less clear. The aim of this project was to establish the degree of collaborative involvement with different partners in HRQoL valuation and map it against emerging good practice recommendations from the health economics and patient-reported outcomes (PRO) literature.

### Methods

A scoping review: (i) identified good practice recommendations for collaborative involvement; (ii) identified instances of collaborative involvement in HRQoL valuation studies; and (iii) mapped (ii) onto (i) to identify gaps. Eight scholarly databases (last searched May 2024) and complementary grey literature sources (last searched September 2024) were searched. Records were screened by two independent reviewers, with discrepancies ratified by a third party. Articles in the health economics and/or PRO literature providing at least one recommendation for best practice in collaborative involvement were included, as were HRQoL valuation studies published since 2019 including example(s) of collaborative involvement activities. Data were extracted by two reviewers independently and ratified. Instances of collaborative involvement in HRQoL were mapped against a thematically synthesised framework of best practice recommendations.

### Results

Twenty-one articles that featured best practice recommendation(s) and 15 HRQoL valuation studies were included. A novel 15-item framework was developed detailing potential best practice recommendations for collaborative involvement. Most valuation studies (n=13) exclusively featured patients and/or experts contributing to the design of the HRQoL states to be valued. There was no evidence in the valuation studies to suggest that 11 out of 15 synthesised recommendations had been applied. There was some evidence for the application of the remaining four, but it was minimal, with the level of evidence uncertain or limited to one or two studies.

### Conclusion

Collaborative involvement in the valuation of HRQoL is underdeveloped and appears unaligned with recommendations provided in the health economics and PRO literature. A novel 15-point best practice framework has been developed, acting as a springboard for refining collaborative working with partners in the valuation of HRQoL.

**Symposium sessions**

## Symposium 1 Key Findings from the EuroQol DAPHNIE Project: A Comprehensive International Study on Population Health Assessment and Comparative Analysis of Health Measurement Instruments Across 15 Countries

Moderator: Fatima Al Sayah^1^

^1^Alberta PROMs & EQ-5D Research & Support Unit (APERSU), School of Public Health, University of Alberta, Edmonton, Alberta, Canada

**Overview**

The symposium will provide an overview of the EQ-DAPHNIE project, an international initiative commissioned by the EuroQol Group to gather comparative data on the performance of EuroQol instruments (EQ-5D, EQ-HWB) and other widely used health-related quality of life and well-being measures, including PROMIS-10, WHO-5, ASCOT, OPQOL, PHQ-2, and GAD-2. The project also examines measures of health behaviors and socio-economic determinants of health. Utilizing online survey panels, the project recruited a representative sample of 4,500 adults from each participating country, drawn based on census data. Countries involved include the UK, Canada, the US, Australia, New Zealand, Germany, France, the Netherlands, Spain, Japan, China, Mexico, Chile, Argentina, and Brazil. In presentation 1, the authors will present an overview of population norms and health inequalities for 15 countries (n=68,375) based on EQ-5D dimension scores, EQ VAS ratings and utility values. Health inequalities will be presented using odd ratios, the Cowell and Flachaire index and the concentration index. In addition, a cross-country analysis will highlight similarities and differences between the 15 countries.In presentation 2, the authors will present on the psychometric validity of the EQ-5D-5L, EQ-HWB-S, and PROMIS-10 across five countries (Australia, Canada, New Zealand, United Kingdom, and United States) in individuals with and without health conditions. In presentation 3, the authors will present on the comparative performance of the EQ-5D-5L, EQ-HWB, and PROMIS-10 in screening for anxiety and depression in the general adult population in 15 countries from around the world. In presentation 4, the authors will examine the psychometric performance of seven EQ-5D-5L bolt-ons (breathing problems, cognition, hearing, tiredness, social relationships, sleep, and vision) in the general populations of the Netherlands and China to determine the value of these bolt-ons in providing a more comprehensive evaluation of health-related quality of life across diverse cultural contexts. In presentation 5, the authors will explore the association between body mass index (BMI) and health-related quality of life (HRQoL) in a diverse global sample from 14 countries participating in the EQ-DAPHNIE project.

**Individual Presenters**

### EQ-5D-5L Population Norms and Health Inequalities across 15 Countries (the EQ-DAPHNIE Project)

Mathieu Janssen^1^, Henry Bailey^2^, Dominik Golicki^3^, Erica Lubetkin^4^, Fredrick Purba^5^, Des Scott^6^, Trudy Sullivan^7^, Jeff Johnson^8^

^1^EuroQol Research Foundation, Rotterdam, Netherlands, ^2^The University of the West Indies, Trinidad, Trinidad and Tobago, ^3^Medical University of Warsaw, Warsaw, Poland, ^4^CUNY School of Medicine, New York, USA, ^5^Padjadjaran University, Bandung, Indonesia, ^6^University of Cape Town, Cape Town, South Africa, ^7^University of Otago, Otago, New Zealand, ^8^University of Alberta, Alberta, Alberta, Canada

#### Aims

The purpose of the current study is to present EQ-5D-5L population norms for 15 countries (N=68,375), resulting from the EQ-DAPHNIE project. Population norms can be used to benchmark the outcomes of patients against the health of the general population or may facilitate the calculation of quality-adjusted life years.

#### Methods

Descriptive EQ-5D-5L data were calculated for the total population by country, gender and seven age groups. Population norms are presented in terms of the five EQ-5D-5L dimension scores, self-reported EQ VAS ratings and EQ-5D-5L utility values. Health inequalities were assessed using odd ratios, the Cowell and Flachaire index and the concentration index. In addition, a cross-country analysis was conducted.

#### Results

Mean self-rated EQ VAS scores ranged from 69.9 in the United Kingdom to 79.8 in China in the total population by country, while mean utility values ranged from 0.797 in the United States to 0.912 in China. Inequality as measured by the concentration index ranged from 0.108 in China to a maximum of 0.172 in the United Kingdom for the EQ VAS. Among the dimensions, the Cowell and Flachaire index ranged from 0.337 for pain/discomfort in Canada to 2.964 for self-care in Japan. Odds ratios generally showed age, sex, education and income to be associated with reporting higher levels of problems on mobility and pain/discomfort in all countries with subsets of these variables having associations with reporting problems on the other dimensions.

#### Conclusion

EQ-5D-5L population norm values may be used in cost-utility analysis, but can also serve as reference data to assess health outcomes or the burden of disease of patients groups. Such information can inform policy-making and assist in setting priorities in health care. Similarities and differences between the 15 countries demonstrate the relevance of assessing and interpreting population norms and health inequalities across countries.

### Comparative performance of EQ-5D-5L, EQ-HWB-S and PROMIS-10 in the EQ-DAPHNIE study

Brendan Mulhern^1^, Fatima Al Sayah^2^, Zhihao Yang^3^, Mihir Gandhi^4^, Erica Lubetkin^5^, Dominik Golicki^6^, MF Bas Janssen^7^, Jeffrey Johnson^2^

^1^University of Technology Sydney, Sydney, Australia, ^2^University of Alberta, Edmonton, Alberta, Canada, ^3^Guizhou Medical University, Guizhou, China, ^4^Duke-NUS, Singapore, Singapore, ^5^City University of New York, New York, USA, ^6^Medical University of Warsaw, Warsaw, Poland, ^7^Erasmus Medical Centre, Amsterdam, Netherlands

#### Aims

The number of generic measures available to assess health-related quality of life (HRQoL) and wellbeing is growing, and it is important to evaluate and compare their psychometric properties across diverse populations. The EQ-DAPHNIE dataset offers a unique opportunity to do this. The aim of this study was to explore the psychometric performance of the EQ-5D-5L, EQ-HWB-S, and PROMIS-10 in the general adult population of Australia (AU), Canada (CA), New Zealand (NZ), the United Kingdom (UK), and the United States (US).

#### Methods

Online surveys were conducted within the five countries in 2024. The EQ-5D-5L and PROMIS-10 were completed by all participants. The EQ-HWB-S was collected in AU, CA and US, and the EQ-HWB-long in NZ and UK. Classical psychometric approaches were used to assess the relationship between measures at the item, domain and value set levels (using country-specific EQ-5D-5L values, and the UK EQ-HWB value set). Analyses were conducted at the overall and country-specific levels, and assessed floor/ceiling effects, convergent validity, and known group validity across demographic and health condition prevalence and severity indicators.

#### Results

A total of 23,610 respondents (AU: 5,036; CA: 4,703; NZ: 4,846; UK: 4,505; US: 4,520) completed the survey and met the quality control criteria. The mean (SD) age was 49 (17) years, and 55% were female. The EQ-5D-5L showed a ceiling effect (25% in the best health state, ranging from 19% (CA) to 30% (NZ)). Strong correlations between the EQ-5D-5L and EQ-HWB-S index values (>0.7) were observed across all countries. The EQ-HWB-S had stronger correlations with the PROMIS global physical and mental health domains (0.67 – 0.73) compared to the EQ-5D-5L (0.49 – 0.64). There was clear divergence between items measuring HRQoL and wellbeing (correlations < 0.4). All measures detected group differences based on health condition and severity indicators, with moderate to large effect sizes.

#### Conclusion

This study uses a unique multi-country dataset to explore the relative psychometric performance of the EQ-5D-5L, EQ-HWB-S and PROMIS-10. The results suggest that all of the measures have a good level of psychometric validity, but the specific HRQoL and wellbeing constructs measured should guide their use in different populations and settings.

### The performance of the EQ-5D-5L, EQ-HWB, and PROMIS-10 in screening for anxiety and depression in the general adult population in 15 countries

Hilary Short^1^, Fatima Al Sayah^1^, Mathieu F. Janssen^2^, Erica Lubetkin^3^, Rosalie Viney^4^, Jeffrey A. Johnson^1^

^1^University of Alberta, Edmonton, Alberta, Canada, ^2^EuroQol, Rotterdamthe, Netherlands, ^3^CUNY School of Medicine, New York City, USA, ^4^University of Technology Sydney, Sydney, Australia

#### Aims

To examine if the EQ-5D-5L, EQ-HWB, and PROMIS-10 are useful measures in screening for anxiety and depression in the general adult population in Argentina, Australia, Brazil, Canada, Chile, China, France, Germany, Japan, Mexico, Netherlands, New Zealand, Spain, United Kingdom (UK), United States (US).

#### Methods

Cross-sectional data from “EuroQol Data for Assessment of Population Health Needs and Instrument Evaluation” project was used. The performance of the EQ-5D-5L, EQ-HWB, and PROMIS-10 were compared to the Generalized Anxiety Disorder 2-item questionnaire (GAD-2) and the Patient Health Questionnaire 2-item version (PHQ-2). GAD-2 and PHQ-2 were used to categorize anxiety and depression, respectively, into absent (<3) and present (≥3). Descriptive statistics were computed for demographic variables. Performance of EQ-5D-5L, EQ-HWB, and PROMIS-10 in screening for anxiety and depression was evaluated using receiver operating curve (ROC) analysis.

#### Results

The mean (SD) age of participants varied from 36.3 (12.6) in Chile to 51.7 (17.0) in Canada. New Zealand had the most females (62.9%) and Germany had the fewest (45.9%). Brazil had the most positive screens for depression (30.1%), anxiety (30.6%), and the combination (20.4%), while China had the least (8.8%, 9.9%, and 4.7%, respectively). Across all countries, EQ-HWB anxiety item performed the best in screening for anxiety, with the highest performances in Australia, UK, and US (area under ROC: 0.90) and the lowest in Chile (0.78). EQ-HWB depression item performed the best in screening for depression with the highest performances in New Zealand, US, and Japan (0.88) and the lowest in China, Mexico, and Argentina (0.81). EQ-HWB anxiety and depression items both performed the highest depending on the country in screening for the combination of anxiety and depression (range: 0.79-0.90). The EQ-5D-5L anxiety/depression dimension (range: 0.71-0.87) and total sum score quintiles (range: 0.71-0.83) also performed well for all screenings across all countries. EQ-VAS and the 4 mental health items of PROMIS-10, PROMIS-10 global mental health T-score and physical health T-score did not perform well for all screenings (<0.37).

#### Conclusion

Generic measures, like EQ-5D-5L and EQ-HWB, are commonly used in population health assessment surveys that could be useful tools in screening for anxiety and depression in the general adult population.

### Comparative Assessment of EQ-5D-5L Bolt-ons in the Netherlands and China: Results from the EQ-DAPHNIE project

Fanni Rencz^1^, Fatima Al Sayah^2^, Zhuxin Mao^3^, Seo-Ho Cho^4^, Lajos V. Kemény^5^, Mathieu F. Janssen^6^

^1^Corvinus University of Budapest, Department of Health Policy & EuroQol Research Foundation, Budapest, Hungary, ^2^Alberta PROMs and EQ-5D Research and Support Unit (APERSU), School of Public Health, University of Alberta, Edmonton, Alberta, Canada, ^3^Centre for Health Economics Research and Modelling Infectious Diseases (CHERMID), University of Antwerp, Antwerp, Belgium, ^4^Faculty of Medicine & Department of Dermatology, Venereology and Dermatooncology, Semmelweis University, Budapest, Hungary, ^5^Department of Dermatology, Venereology and Dermatooncology & HCEMM-SU Translational Dermatology Research Group & Department of Physiology, Faculty of Medicine & MTA-SE Lendület “Momentum” Dermatology Research Group, Hungarian Academy of Sciences and Semmelweis University, Budpapest, Hungary, ^6^Section Medical Psychology and Psychotherapy, Department of Psychiatry, Erasmus MC, Rotterdam, Netherlands

#### Aims

EQ-5D-5L bolt-ons are additional dimensions designed to provide a more comprehensive assessment of health-related quality of life. This study aimed to compare the psychometric performance of a set of EQ-5D-5L bolt-ons in the Netherlands (NL) and China (CN).

#### Methods

As part of the EuroQol Data for Assessment of Population Health Needs and Instrument Evaluation (EQ-DAPHNIE) project, online cross-sectional surveys were conducted in 2024 with representative samples from the adult general population in the NL (n=4506, mean age 49 years, 26% aged 65+, women 55%, (sub)urban 88%) and CN (n=4519, mean age 47 years, 20% aged 65+, women 47%, (sub)urban 66%). Data were collected on the EQ-5D-5L, seven bolt-ons (breathing problems, cognition, hearing, tiredness, social relationships, sleep and vision) along with sociodemographic and health-related characteristics. Psychometric assessments included distributional characteristics, convergent and divergent validity (Spearman’s correlations), explanatory power on EQ VAS (multivariable linear regressions) and known-groups validity (eta-squared effect size) for general health (excellent-to-poor scale).

#### Results

The EQ-5D-5L ceiling effect was lower in the NL (32%) compared to CN (45%). The vision (NL: 22%, CN: 30%), tiredness (NL: 23%, CN: 35%) and sleep (NL: 25%, CN: 35%) bolt-ons reduced the ceiling most, while breathing problems had the smallest effect (NL: 30%, CN: 43%). Most bolt-ons were only weakly correlated with EQ-5D-5L dimensions in both countries, with only tiredness and social relationships correlating strongly with any of the EQ-5D-5L dimensions. The core dimensions explained more variance in EQ VAS in NL (37%) than in CN (24%), with tiredness, sleep and cognition bolt-ons contributing the most to increasing the variance explained. The EQ-5D-5L level sum scores discriminated between general health groups in both countries, with a larger effect size in NL (0.337) compared to CN (0.205). In NL, only the tiredness bolt-on increased the effect size notably (0.367), while in CN, tiredness, sleep and cognition also performed well (0.237-0.247).

#### Conclusion

The results highlight the value of the tiredness, sleep and vision EQ-5D-5L bolt-ons for use in general population samples. Cross-country differences in the psychometric gain compared to the EQ-5D-5L can, in part, be explained by the different demographic structures of the samples.

### The Association Between Body Mass Index and Health-Related Quality of Life in the General Population: Data from 14 Countries in the EQ-DAPHNIE Project

Erica Lubetkin^1^, Fatima Al Sayah^2^, Dominik Golicki^3^

^1^CUNY School of Medicine, New York, USA, ^2^Alberta PROMs & EQ-5D Research & Support Unit (APERSU), School of Public Health, University of Alberta, Edmonton, Alberta, Canada, ^3^Department of Experimental and Clinical Pharmacology, Medical University of Warsaw, Warsaw, Poland.

#### Aims

The association between overweight or obesity and reduced health-related quality of life (HRQoL) has been described in populations from highly developed countries (e.g., the USA, United Kingdom), specific provinces, or particular subgroups (e.g., the elderly). The aim of this study is to investigate this relationship in a diverse group of countries from five continents participating in the EQ-DAPHNIE project.

#### Methods

Data from 14 countries collected between 2023 and 2024 as part of the EQ-DAPHNIE project were used. Body mass index (BMI) was calculated based on self-reported weight and height. Respondents were classified into four categories according to BMI: underweight, normal weight, overweight, and obesity. HRQoL outcomes, based on the EQ-5D-5L questionnaire, including EQ-Index, EQ VAS, and limitations across five dimensions (mobility, self-care, usual activities, pain/discomfort, anxiety/depression), were assessed. The EQ-5D-5L index was calculated using both country-specific value sets and a common value set for all countries. The prevalence of overweight and obesity was compared across countries. The impact of overweight and obesity on HRQoL was examined using multivariate regression analysis, adjusting for sociodemographic factors and the prevalence of chronic diseases.

#### Results

Among 51,529 respondents, the prevalence of underweight, normal weight, overweight, and obesity was 8.2%, 45.4%, 26.6% and 19.8%, respectively. The prevalence of obesity varied between countries, ranging from 4.3% in Japan to 29.7% in Argentina. Unadjusted HRQoL, as measured by EQ VAS, was highest in normal weight respondents (76.9) and lowest in obese (69.3). Multivariate regression analysis is currently underway.

#### Conclusion

Overweight and obesity are increasingly significant public health issues, negatively affecting HRQoL as measured by the EQ-5D-5L. Ongoing analyses will further elucidate the detailed nature of these relationships, adjusting for sociodemographic factors and the prevalence of chronic diseases.

## Symposium 2 Sharing knowledge and seeking solutions: Lessons from research networks to advance the use of PROs in clinical practice

Moderator: Norah Crossnohere^1^

^1^The Ohio State University, Columbus, USA

**Overview**

Institutions are increasingly working to use patient-reported outcomes (PROs) in routine care because of growing evidence supporting their benefits, but PRO use can be challenging. The PROTEUS Consortium, the OncoPRO Initiative, and the UK PROMs Network have been formed to share knowledge and seek solutions to these challenges. In this symposium, these networks will summarize the real-world barriers their participants have faced and the solutions they have identified to advance the use of PROs in clinical practice.The PROTEUS Consortium Learning Health Network, comprised of institutions conducting PRO implementation and quality improvement projects, convened monthly to identify challenges and share solutions related to the 16 topics from the PROTEUS-Practice Framework on designing, implementing, and managing PRO systems.OncoPRO is a national initiative in the US that supports oncology practices and health systems in the implementation and sustainability of remote symptom monitoring programs using electronic PROs. The central activities of OncoPRO encompass co-learning collaborative monthly meetings where leaders in clinical care, information systems, and value-based care convene and discuss barriers and strategies for implementation.The UK PROMs Network brings together academics, clinicians, patient representatives, and policy makers who are committed to working collaboratively to share experiences and support on processes for PRO collection, analysis, and benchmarking. Their aim is to ensure transparency and effective utilization of PRO measures. The network supports newly appointed PROMs coordinators in NHS Trusts so that they can develop their own PROMs programs and learn from the experiences of others, through working groups and educational webinars.The presentations from the three networks will be followed by a patient advocate discussant who will (1) reflect on the cross-cutting themes regarding challenges and solutions and (2) describe the role of patient engagement in identifying challenges and finding solutions. The symposium will conclude with an interactive audience discussion.

### Navigating challenges to PRO use in oncology care: Insights from the PROTEUS Learning Health Network

Anne Schuster^1^, Aileen Chen^2^, Angela Stover^3^, Anobel Odisho^4^, Debbie Liang^3^, Jennifer Cracchiolo^5^, Jordan Marchak^6^, Kenneth Meehan^7^, Kuang-Yi Wen^8^, Laila A. Gharzai^9^

^1^The Ohio State University, Columbus, USA, ^2^University of Texas MD Anderson, Houston, USA, ^3^University of North Carolina Chapel Hill, Chapel Hill, USA, ^4^University of California San Francisco, San Francisco, USA, ^5^Memorial Sloan Kettering Cancer Center, New York, USA, ^6^Emory University, Atlanta, USA, ^7^Dartmouth, Hanover, USA, ^8^Thomas Jefferson University, Philadelphia, USA, ^9^Northwestern University, Evanston, USA

#### Aims

Growing evidence indicates that using patient-reported outcomes (PROs) in cancer care can improve patient outcomes, but multi-level barriers hinder their use. The PROTEUS Learning Health Network (LHN) helped groups navigate the real-world challenges of routine PRO use.

#### Methods

The PROTEUS LHN included 10 projects focused on implementing or improving PRO use in oncology care, along with other PROTEUS participants. The LHN met monthly to address key considerations for designing, implementing, and managing PRO systems from the “PROTEUS-Practice Framework”. Meetings included structured presentations, resource sharing, open discussions, and developing strategies to address project-specific challenges.

#### Results

The LHN projects reflect diverse applications of PROs in oncology care led by multidisciplinary experts across pediatric and adult populations. Project goals include screening, monitoring, and outcomes assessment, spanning in-patient, out-patient, and pharmacy-based settings. During LHN sessions, groups developed strategies to address five real-world challenges. (1) Clinical workflow challenges were addressed by providing tailored provider training, scripts, and standard operating procedures, as well as engaging key stakeholders to refine PRO collection into existing workflows. (2) PRO administration issues were addressed by offering multiple methods for PRO collection, along with providing clear instructions and explanations on the purpose and value of PROs for staff and patients. (3) Electronic health record (EHR) integration issues were tackled by creating or establishing close relationships with technical implementation teams (including EHR engineers and analysts), creating tailored EHR templates, and building in systems for feedback and support. (4) Approaches to improve reporting of and responding to PRO results include being purposeful about PRO result visualization, having internal champions regularly share impact, and reminding providers to explicitly mention to patients they had seen their PRO results. (5) Efforts to achieve more equitable and inclusive PRO collection included translating PRO measures into different languages, providing PRO collection options that did not require internet access or digital literacy, and making PRO screening/monitoring universally available (with an opt-out option).

#### Conclusion

The PROTEUS LHN facilitated continuous learning and capacity building for PRO use across diverse oncology clinical practice projects. The identified challenges and solutions offer valuable insights for others interested in implementing PROs in clinical practice.

### Learning from the OncoPRO Initiative

Gabriele Rocque^1^, Jennifer Jansen^2^, Patty Spears^2^, Ethan Basch^2^

^1^UAB, Birmingham, USA, ^2^UNC, Chapel Hill, USA

#### Aims

Remote symptom monitoring using electronic patient-reported outcomes (ePROs) results in improved symptoms management, communication, quality of life, and in some cases survival. Furthermore, value-based healthcare is increasingly requiring RSM. However, implementation at scale in real-world settings remains challenging for practices.

#### Methods

OncoPRO is a national initiative in the United States that supports oncology practices and health systems in the implementation and sustainability of remote symptom monitoring programs using electronic patent-reported outcomes, integrated with electronic health record systems. OncoPRO is funded by the Patent-Centered Outcomes Research Institute (PCORI), and is led by operational groups at the University of North Carolina and the University of Alabama (Co-Leads Ethan Basch and Gabrielle Rocque), in partnership with the American Society of Clinical Oncology (ASCO), the American Cancer Society (ACS) and PROTEUS.

#### Results

The central activities of OncoPRO encompass co-learning collaborative monthly meetings where leaders from each practice in clinical care, information systems, and value-based care convene to review progress, barriers and strategies for implementation, billing tactics, and other guidance. Practices share data on implementation, discuss challenges they face, and exchange success stories under the facilitation of ASCO coaches. Support materials and standard operating processes are shared by the operational team, ACS, and software vendors. Currently, there are 15 large practices/health systems participating, as well as two national practice networks, four EHR software companies, four ePRO software companies, and observers from the U.S. Centers for Medicare and Medicaid Services and the Food and Drug Administration. Additional practices are joining the initiative both within the US and internationally. A goal is to demonstrate that implementation of remote symptom monitoring with ePROs is feasible on a wide basis and leads to improved operational and clinical outcomes.

#### Conclusion

The OncoPRO initiative is supporting practices, including both community and academic, in implementing remote symptom monitoring using ePROs through a learning collaborative that is anticipated to be of interest to others considering ePRO implementation.

### Sharing knowledge and seeking solutions: Lessons from research networks to advance the use of PROs in clinical practice

Antoinette Davey^1^, Jonathan Evans^2^, Anji Kingman^3^, Kanthan Theivendran^4^, Joanne Greenhalgh^5^, Claire Snyder^6^

^1^University of Exeter and Devon Partnership NHS Trust, Exeter, UK, ^2^University of Exeter, Exeter, UK, ^3^Northumbria Healthcare NHS Trust, Hexham, UK, ^4^Sandwell and West Birmingham Hospitals NHS Trust, Birmingham, UK, ^5^University of Leeds, Leeds, UK, ^6^John Hopkins University, Baltimore, Maryland, USA

#### Aims

The UK provides a universal healthcare system with access based on clinical need rather than the ability to pay. However, despite efforts to ensure equitable access to healthcare, there has been limited investment in the routine collection of Patient-Reported Outcome Measures (PROMs). This seminar aims to explore the challenges associated with implementing PROMs in the UK healthcare system and identify successful examples of overcoming these barriers.

#### Methods

An analysis of the current state of PROMs collection in the UK was conducted, focusing on national strategies such as the English National PROMs Programme. Insights were drawn from the Cumberlege independent review (2018–2020), which recommended the wider and more routine collection of PROMs and Patient-Reported Experience Measures (PREMs). Additionally, case studies of existing PROMs implementation within NHS Trusts, such as Northumbria Healthcare NHS Foundation Trust, were examined.

#### Results

Findings reveal that PROMs data collection in the UK has been fragmented due to factors such as a lack of coordination within clinical teams, the absence of standardized assessment approaches, poor or non-existent reporting, and limitations within the IT infrastructure. Despite these challenges, pockets of good practice exist, with successful implementation observed in some healthcare settings through the use of electronic health systems, such as the openOutcomes platform. Furthermore, NHS England is actively transitioning several registries, including the Breast and Cosmetic Implant Registry and the Hip and Knee PROMs, to a new platform to improve data integration.

#### Conclusion

While the UK faces significant challenges in embedding PROMs into clinical practice, examples of successful initiatives demonstrate that these barriers can be overcome with strategic coordination and improved technological infrastructure. The seminar will discuss these challenges in detail and highlight best practices that can inform future efforts to enhance the routine collection and utilization of PROMs within the UK healthcare system.

## Symposium 3 Treatment burden in cancer: Exploring the unsung work of patients and survivors

Moderator: David T. Eton^1^

^1^Outcomes Research Branch, Healthcare Delivery Research Program, DCCPS, National Cancer Institute, Rockville, USA.

**Overview**

Treatment burden is the personal work associated with healthcare for chronic medical conditions and its impact on functioning and well-being. This work can consist of a range of self-care activities like managing medications, organizing and attending medical appointments, monitoring health conditions, knowledge acquisition, and managing medical expenses and reimbursement. The volume, complexity, and time it takes to complete this work can impact a person’s ability to participate in valued life roles and activities and lead to a sense of exhaustion. Yet this work and its effects are less visible to healthcare providers. Treatment burden research over the past 15 years has largely centered on people with multiple chronic conditions and has been primarily focused on conceptualizing and measuring the construct as well as characterizing its effects. Less attention has been paid to understanding how treatment burden is experienced by people living with certain index conditions. The goal of this symposium is to explore how treatment burden is experienced by people living with cancer, including how the concept extends beyond notions of “financial toxicity.” We bring together nursing and health services researchers from the United States and Norway to help set an agenda for “cancer-related” treatment burden research. This will include consideration of the following questions: (1) what are the most salient elements of treatment burden to cancer patients, survivors, and caregivers, and how might they interact? (2) how does treatment burden differ across phases of the cancer continuum? (3) do symptoms and treatment side effects exacerbate treatment burden? (4) how do personal and social factors modify the experience of treatment burden? (5) how can healthcare providers (both those in oncology and primary care) help minimize treatment burden and improve care delivery? The three individual presentations will be followed by a moderated panel discussion integrating audience Q&A. Learning objectives include acquiring a basic understanding of treatment burden and how it manifests in cancer, recognizing the costs of treatment burden to patients, survivors, caregivers, and the healthcare system, understanding measures and interventions available to monitor and ameliorate treatment burden, and identifying future directions for research on cancer-related treatment burden.

**Individual Presenters**

### The intersecting time, administrative, and financial burdens of a cancer diagnosis

Helen Parsons^1^

^1^University of Minnesota, Minneapolis, USA

#### Aims

Cancer and its care create substantial financial, time, and administrative burdens both for patients and their loved ones. Althoughcancer-related financial burdens have been well documented in the past decade, time and administrative burdens of cancercare have received substantially less attention. The aim of this abstract is to map, measure, and describe example interventions to address the time, administrative, and financial burdens of cancer and its care.

#### Methods

We created a conceptual framework based on the literature of the intersecting time, administrative and financial burdens that may exist after a cancer diagnosis. Time burdens are defined as the burdens patients and caregivers experience because of the time needed to complete cancer-relatedtreatment and tasks that take away from other life responsibilities. Relatedly, administrative burdens are burdens patients and caregivers experience because of cancer-related, resource-consuming bureaucratic and logistical tasks. Finally, financial hardship can be conceptualized as problems patients experience related to the cost of medical care. Using this conceptual framework, we then examples of our work that demonstrate these burdens and present key opportunities for intervention that may address underlying burdens.

#### Results

Our conceptual framework highlights that time, administrative and financial burdens are not distributed equally across the population, with those who have lower incomes, females, younger individuals, persons of color, non-partnered and those providing care to dependents likely experiencing higher burden. These burdens have important immediate outcomes such as potentially lower adherence to recommended care, higher cancer-related costs, less income and higher mental load. Over time, these burdens can lead to poorer long-term outcomes such as lower quality of life, increased anxiety and debt. Based on this conceptual framework, we highlight key examples of work from our team within each area, including estimates of financial hardship after a cancer diagnosis, projections of contact days with the healthcare system across key demographic and treatment characteristics and examples of administrative burdens from qualitative interviews. We then highlight key areas of intervention and ongoing work to address burdens after a cancer diagnosis.

#### Conclusion

A cancer diagnosis can result in substantial time, administrative, and financial demands resulting in poor outcomes and creating important opportunities to intervene.

### Communication of expected treatment burden, quality of life, and survival to patients with brain metastases for shared decision-making

Roger Anderson^1^, Kathleen Porter^1^, Gloribel Bonilla^1^, Camilo Fadul^2^

^1^University of Virginia, Charlottesville, USA, ^2^University of Virginia UVA Health, Charlottesville, USA

#### Aims

The impact of treatment burden on people living with incurable, metastatic cancer is poorly understood. Patients with brain metastases (BMETS) face considerable treatment burden and QoL impact, including long-term neurotoxicity from brain-directed therapies, such as whole brain radiation therapy (WBRT). The tradeoffs of treatment burden for possible gains in survival time are personal decisions which require informed shared decision-making to align goals of care with treatment. We reviewed patients’ experience with the BMETS treatment burden valued aspects of quality-of-life (QoL) to guide the development of a decision-aid tool to support optimal care.

#### Methods

BMETS patient QoL goals and treatment experience were summarized from the literature and interviews conducted with patients (n=18) and caregivers (n=13) recruited from three academic cancer centers. We analyzed interview transcripts using a hybrid of directed and conventional content coding. To quantify time toxicity with BMETS, we examined number of days with an inpatient stay or outpatient visit, and number of treatment providers using pooled EHR data from the Cosmos Data Science Virtual Machine using Microsoft SQL and R. Log-rank and Wilcoxon rank-sum tests were used to analyze median and overall survival (OS).

#### Results

QoL goals reported by patients and caregivers included engaging with family and friends, limiting decreases in or regaining lost physical function, maintaining or regaining lost role function, and traveling or engaging in desired social activities. For time toxicity and visit burden, among the 4,023 patients with BMETS retrieved from Cosmos, median survival was 286 days of which approximately 55 days (19% of OS) involved inpatient stays and outpatient visits from an average of 10 different providers. Time toxicity and complexity of treatment were significantly higher for patients who WBRT versus stereotactic radiation(SRS), but resulted in statistically similar OS (p=0.298). In regression analysis, an increase in time toxicity was associated with shorter survival (-0.61, p < .001).

#### Conclusion

Treatment burden and QoL impact from BMETS is profound. For some patients more intense treatment confers little benefit to OS. Study findings revealed the need for patient decision-aid tools to support informed shared decision-making and clarify preferences and values regarding potential time toxicity, treatment burden, QoL and survival.

### Effects of remote patient monitoring on treatment burden in colorectal cancer patients – a randomized, controlled trial

Anne Marie Lunde Husebø^1^, Ingvild Morken^1^, Marianne Storm^1^, Hege Wathne^1^, Rosalynn Austin^1^, Bjørg Karlsen^1^, Kristin Hjorthaug Urstad^1^

^1^Faculty of Health Sciences, University of Stavanger, Stavanger, Norway

#### Aims

We report results of a study to test the effects of a 6-week RPM intervention on self-management patient workload and QOL in patients surgically treated for early-stage CRC.

#### Methods

In this RCT, 75 CRC patients surgically treated for cure at a university hospital in Norway were randomly assigned to a 6-weeks nurse-assisted posthospitalization RPM intervention (n=36), or to standard care (n=38) between May 2023 and May 2024. The RPM intervention included daily and weekly monitoring of vital signs, symptom checklists, and real-time communication with hospital-based nurse specialists via chat and video conferences facilitated by a patient application system (MyDignio). Data collection included The Patient Experience with Treatment and Self-management (PETS) measure of treatment burden and the European Quality of Life-5 dimensions questionnaire at baseline (hospital discharge), 6 weeks (post-intervention) and 6 months post-baseline (follow-up). Data are analyzed according to intention-to-treat principles in SPSS and R-STATA.

#### Results

Data analyses are currently ongoing. We will present results on analyses of the effects of RPM on treatment burden and quality of life. We hypothesize that compared to standard care, RPM will meet post-discharge needs for support and diminish the treatment burden of patients with CRC, leading to higher levels of QOL.

#### Conclusion

Our results will determine whether RPM when transitioning from an inpatient hospital setting to home after CRC surgery can positively affect a patient’s experience of treatment burden, ultimately leading to improvements in the patient’s well-being. Challenges with implementation and opportunities for integration into clinical practice will be discussed.

## Symposium 4 PRO be nimble, PRO be quick: Challenges, opportunities, and the unique experiences of standing up a centralized PRO data collection system alongside a clinical registry for hematopoietic stem cell transplantation and cellular therapy patients

Rachel Cusatis^1^

^1^Medical College of Wisconsin, Milwaukee, USA

**Overview**

Hematopoietic cell transplantation (HCT) is used as an established therapy to control and/or cure many hematologic diseases, including malignant and non-malignant, congenital and acquired diseases of the hematopoietic system, and some solid tumors. Further, more recent advances have led to the development of other cellular therapies (such as gene and CAR-T therapies) to treat diseases, some of which have been approved in childhood and adult oncologic diseases. As cellular therapy recipients are living longer, late effects and survivorship research is a crucial initiative of the transplant community. PROs have been shown to have great importance in the evaluation of patients, studying late effects and survivorship, and even serving as predictive indicators of important transplant outcomes, including survival.Center for International Blood and Marrow Transplant Research (CIBMTR) is clinical outcomes registry for recipients of cellular therapies with longitudinal data collected on >700,000 patients since the 1970s. Recognizing the importance of quality of life (QOL) and the value of PROs, the registry more recently developed an infrastructure to collect PROs on registry patients. The overarching goal is to incorporate patient-centered measures with clinical data to understand the impact of outcomes and events on patients, too assess associations of QOL and other factors to inform interventions, and overall management of late effects and survivorship of recipients of cellular therapies. The process began with a convening stakeholders in 2017, enrolled our first patient August of 2020, to now having enrolled over 1500 patients. Standing up an infrastructure to centrally collect PROs among hundreds of centers inevitably comes with unexpected obstacles, surprises, and learning lessons along the way. But, already the data are used to understand patients’ symptoms and functioning over time, assess patients’ well-being prior to receiving treatment, and leveraged as real-world data to compare to clinical trial patient populations. This symposium expands upon the successes and challenges of the CIBMTR PRO program and we present two illustrative examples of the value of this registry-based real world PRO data collection.

**Fig. 3:**
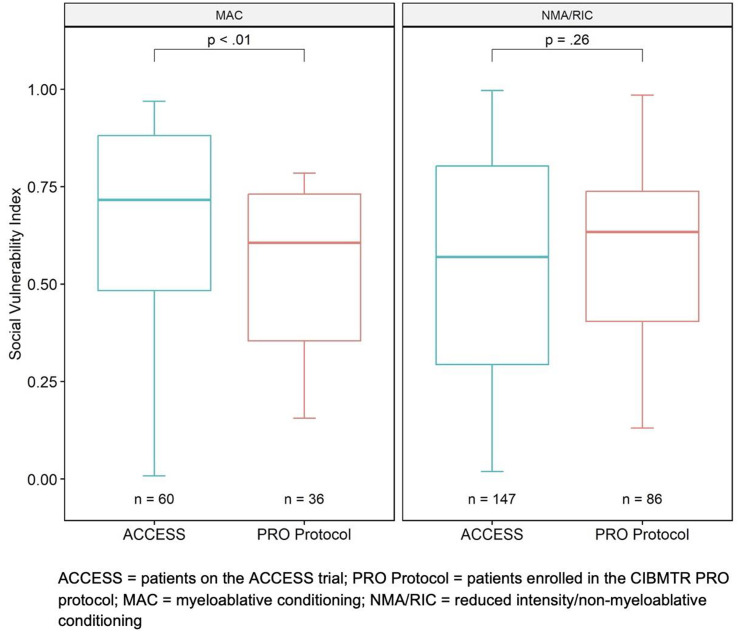
Box Plots of Average Social Vulnerability Index Scores for ACCESS trial and CIBMTR PRO Protocol patients, separately by conditioning regimen. ACCESS = patients on the ACCESS trial; PRO Protocol = patients enrolled in the CIBMTR PRO protocol; MAC = myeloablative conditioning; NMA/RIC = reduced intensity/non-myeloablative conditioning

**Fig. 1:**
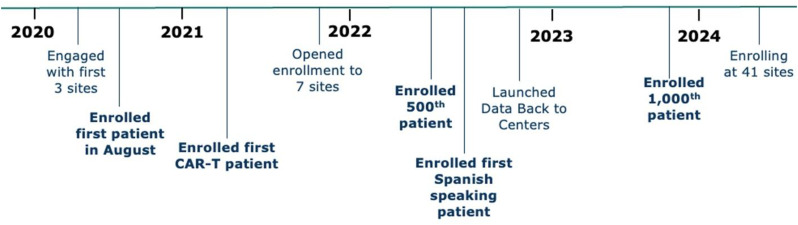
Timeline of CIBMTR PRO Protocol Development

**Fig. 2:**
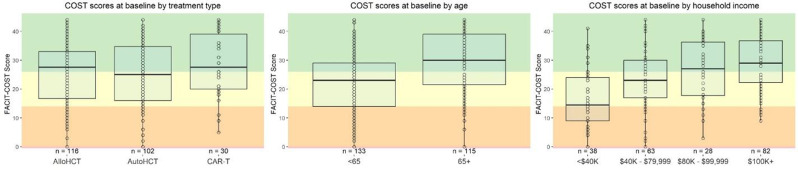
Box Plots of COST-FACIT Scores by treatment type, age (<65 v. 65+), and household income

**Fig. 1:**
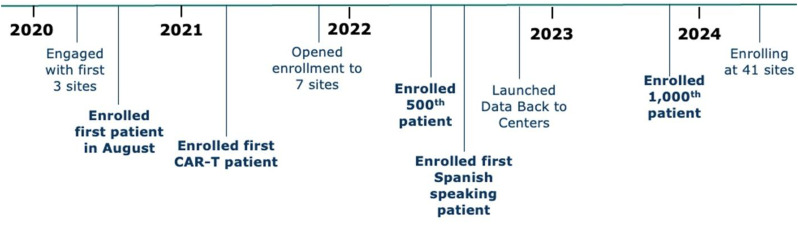
Timeline of CIBMTR PRO Protocol Development

**Fig. 2:**
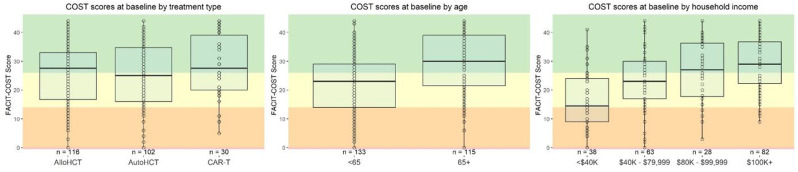
Box Plots of COST-FACIT Scores by treatment type, age (<65 v. 65+), and household income

**Individuals Presentations**

### The Who, What, Where of CIBMTR PRO registry and an example of troubleshooting low response rates in the CIBMTR PRO registry data collection

Deborah Mattila^1^

^1^CIBMTR/NMDP, Minneapolis, USA

#### Aims

Building an infrastructure to centrally collect PROs from transplant centers inevitably comes with challenges and opportunities to learn and grow. Here we describe the build out of the CIBMTR PRO data collection and how obstacles were addressed.

#### Methods

First, CIBMTR convened a panel of experts to recommend the domains and measurement tools most relevant to the HCT population. PROMIS measures were selected with preferred delivery through an electronic system to administer CATs, though paper and telephone versions are available to meet modalities preferences of all patients. Timepoints to collect PROs included pre-treatment (baseline), day 100 post-treatment, day 180, 1 year, and yearly thereafter. CIBMTR developed an electronic PRO (ePRO) system for routine PRO data collection, under a centralized IRB-approved protocol with all enrollment, consent, and data collection activities performed by CIBMTR. The ePRO incorporates a patient-friendly interface in Qualtrics, an API link to the PROMIS measures, links to a client management system to track and trigger PROs and links to the CIBMTR clinical database to store PROs. Individual patient PROs are linked to their clinical data within the CIBMTR data warehouse. The ePRO system also facilitates electronic consent and most outreach is by email or telephone.

#### Results

PRO Protocol enrollment began in August 2020 with 3 champion centers. Expansion to other centers was intentionally gradual. As of August 2024, we have enrolled 1299 patients from 45 centers who completed 3584 surveys. The enrollment was consistently at 34%, with a marked difference by race/ethnicity. Multiple solutions were tested to improve this. For example targeting outreach to racial/ethnic minorities, expanding language offerings to Spanish speaking patients, and changing the modality of initial contact to Hispanic patients. The most successful of these was a letter outreach pilot where Hispanic patient enrollment rates improved from 25% to 48%.

#### Conclusion

Overall, enrollment rates for CIBMTR PRO Protocol are comparable to other cancer registries, with promising improvements when tailoring outreach to meet patients needs. Collecting real world PRO data in a registry has multiple benefits for research, and ultimately patient care and experience.

**Fig. 1 (Symposium 4.1):**
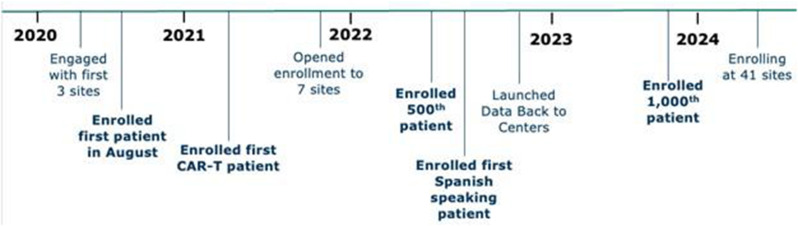
The Who, What, Where of CIBMTR PRO registry and an example of troubleshooting low response rates in the CIBMTR PRO registry data collection

### Understanding the whole patient prior to receiving transplant and cellular therapy: Real-world data on patients financial toxicity prior to receiving treatment

Miranda Kapfhammer^1^, Rachel Cusatis^1^, Deborah Mattila^2^, Sarah Smith^2^

^1^Medical College of Wisconsin, Milwaukee, USA, ^2^National Marrow Donor Program (NMDP), Minneapolis, USA

#### Aims

Cellular therapy (CT) recipients are at high risk for financial toxicity (FT) given the frequent need for temporary re-location, loss of income for patients and caregivers in addition to the expected medical expenses of cancer care. This study leverages the patient reported outcome (PRO) data collected by the CIBMTR to describe pre-CT patient-reported FT.

#### Methods

Patients were included in this analysis if they completed a pre-HCT/CT baseline survey through the CIBMTR PRO protocol. FT was measured using the COmprehensive Score for FT (COST), a validated 12-item measure on financial stressors of cancer care. COST includes a summary score (0 to 44), with higher scores indicating better financial well-being. Validation studies established clinically meaningful grades of FT: scores ≥26 = grade 0 (no FT); 14–25 = grade 1 (mild FT); < 14 = grade 2 (moderate FT); 0 = grade 3 (severe FT). Medians and standard deviations were used to describe COST by patient characteristics. Kruskal Wallis statistics were used to test categorical variables for differences in scores by patient characteristics. Associations between FT and anxiety and depression, measured with Patient Reported Outcomes Measurement System (PROMIS), were examined using Spearman correlation.

#### Results

Of the 255 patients from 17 US centers, 46% (n=118) received allogeneic HCT, 42% (n=106) autologous, and 12% (n=31) CAR-T therapy. Median age at infusion was 63.5 years; 50% were female; 80% were white Non-Hispanic. Pre-HCT/CT median COST scores was 26 (range 0-44), indicating low FT; with no significant differences by CT type. Younger patients (23 for <65 versus 30 for 65+, p<.01)], racial and ethnic minorities (16 vs 28 in non Hispanic white, p<0.1) and patients with lower household income (15 <$40K, 29 >$100K, p<.01) had significantly worse FT. Pre-HCT/CT anxiety and depression were moderately correlated with COST scores at baseline (spearman -.37 for both).

#### Conclusion

These findings confirm a varied experience of FT among a convenience sample of HCT/CT patients pre-infusion. Our data confirms potential disparities in FT by age, race/ethnicity, and household income. Our findings provide evidence to identify at risk patient populations about to receive HCT/CT, which can recognize important opportunities to intervene.

**Fig. 1 (Symposium 4.2):**
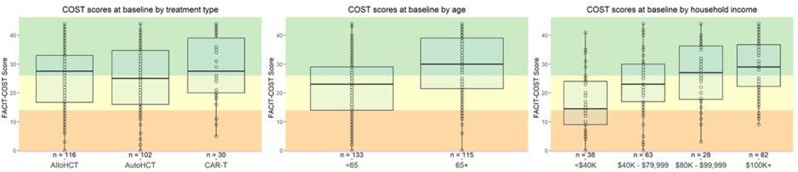
Understanding the whole patient prior to receiving transplant and cellular therapy: Real-world data on patients financial toxicity prior to receiving treatment

### PRO be nimble, PRO be quick: Challenges, opportunities, and the unique experiences of standing up a centralized PRO data collection system alongside a clinical registry for hematopoietic stem cell transplantation and cellular therapy patients

Rachel Cusatis^1^, Deborah Mattila^2^, Miranda Kapfhammer^3^

^1^Medical College of Wisconsin, Milwaukee, USA, ^2^National Marrow Donor Program, Minneapolis, USA, ^3^Center for International Blood and Marrow Transplant Research, Austin, USA.

#### Aims

Disparities in access to HCT and in clinical trial participation exist among lower socioeconomic and racial/ethnic diverse patients. To address such disparities, the ACCESS trial (NCT04904588) studied the safety and efficacy of using peripheral blood stem cells from mismatched unrelated donors (MMUD) using post-transplantation cyclophosphamide as GVHD prophylaxis in patients with high-risk hematologic malignancies. Here we compare social vulnerabilities of ACCESS trial patients with a real-world sample of HCT patients reported to the CIBMTR registry.

#### Methods

This study included patients enrolled in the ACCESS trial and patients enrolled in the CIBMTR PRO Protocol (PRO Protocol) who received unrelated donor HCT. Baseline socioeconomic, social determinants of health (SDoH), and patient characteristics, were analyzed by conditioning regimen. SDoH was operationalized using the CDC developed social vulnerability index (SVI), a place-based index designed to quantify social vulnerability. SVI creates a score from 0–1 where higher values indicate greater social vulnerability. Financial toxicity was assessed using the 12-item COmprehensive Score for financial Toxicity (COST). PROMIS cognitive function, anxiety, and depression were used to identify mental well-being. Wilcoxon rank-sum test and Fisher exact test were used to determine statistically significant differences between groups (p<0.05).

#### Results

208 patients (n=60 MAC, 80% response rate and n=148 RIC/NMA, 77% response rate) enrolled in the ACCESS study and 122 PRO protocol patients (n=36 MAC; n=86 RIC/NMA) completed baseline surveys. ACCESS included 53% Non-Hispanic White (NHW) patients versus 85% NHW patients on the PRO protocol. no significant differences were noted between ACCESS and PRO protocol patients for baseline financial toxicity cognitive function, anxiety, or depression. However, differences included: (1) in the MAC stratum, ACCESS patients had higher socially vulnerability versus patients on the PRO protocol (.72 and .61, respectively; p<0.01) (Figure 3). (2) in the RIC stratum, a significantly larger portion of trial patients had government-sponsored insurance (44%) compared to PRO Protocol patients (29%; p<0.05).

#### Conclusion

ACCESS trial patients were significantly more socially vulnerable, which was not simply attributable to increased racial/ethnic diversity, compared to real-world registry patients receiving comparable treatment. Understanding these differences can help in targeting future interventions, as well as providing guidance for appropriate patient-assistance resources.

**Fig. 1:**
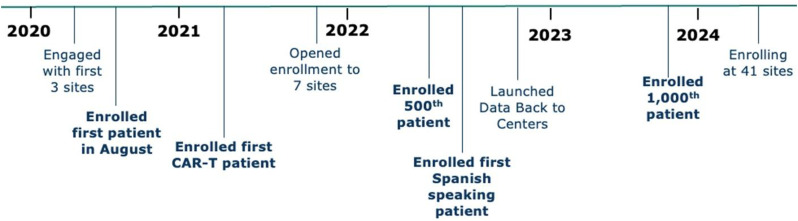
Timeline of CIBMTR PRO Protocol Development

**Fig. 2:**
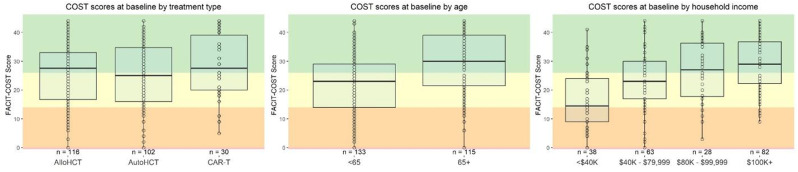
Box Plots of COST-FACIT Scores by treatment type, age (<65 v. 65+), and household income

**Fig. 3:**
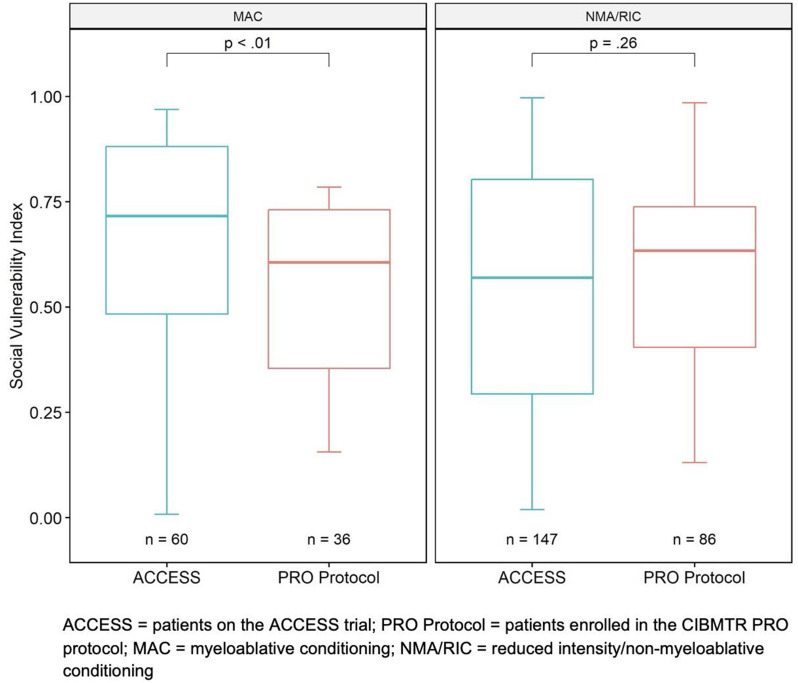
Box Plots of Average Social Vulnerability Index Scores for ACCESS trial and CIBMTR PRO Protocol patients, separately by conditioning regimen

## Symposium 5 Possibilities for measuring and scoring whole-person health

Moderator: Ron Hays^1^

^1^UCLA Department of Medicine, Los Angeles, USA

**Overview**

Whole-person health (WPH) is the end goal or outcome of whole-person care. While there is increasing interest in measuring WPH, there is noteworthy variation in measuring it and how it is scored. Four United States (U.S.) Behavioral Risk Factor Surveillance System items were combined to create a summary score to monitor population trends. A subsequent study supported aggregating Patient-Reported Outcomes Measurement and Information System (PROMIS®) measures, the personal well-being index, and the EQ-5D-5L into a WPH score study. Authors of a scoping review recommended that WPH measures represent physical, mental, social, spiritual, and personal development domains. The Whole Person Health Score goes beyond health and includes questions about resource utilization, socioeconomics, ownership, nutrition, and lifestyle. A committee report of the U. S. National Academies of Sciences, Engineering, and Medicine suggested incorporating existing measurement efforts, having stakeholder involvement, and distinguishing between WPH and its social and cultural determinants. Reflective (“effect”) indicators are observed variables assumed to be affected by an underlying latent variable. Formative (“causal”) indicators affect the underlying latent variable. Covariance among causal indicators can exist regardless of associations with an underlying latent variable. Understanding how interventions work requires the correct specification of indicators. In this symposium, a panel of speakers will present different perspectives on measuring and scoring WPH. Speakers include the developer of the PROMIS portfolio of measures, a health preference expert, and two psychometricians with extensive experience evaluating self-report measures. Topics to be discussed include how PROMIS measures represent whole-person health, a framework for assessing preferences for health states, a scoping review of whole-person health measures, use of the bifactor model to evaluate whole-person health, and distinguishing between causal and reflective health indicators. The panel will also provide recommendations for future research.Panelists will touch upon:1)Use of the Patient-Reported Outcomes Measurement and Information System to Estimate Whole-Person Health2)A Framework for Assessing Preferences for Whole-Person Health States3)Scoping Review of the Measurement of Whole-Person Health4)Using the Bifactor Model to Estimate Whole-Person Health.

**Individual Presenters**

### Measuring Whole Person Health: A Scoping Review

Maria Edelen^1^, Graham DiGuiseppi^2^, Anthony Rodriguez^3^, Nabeel Qureshi^4^, Chengbo Zeng^5^, Ian Coulter^4^, Ron Hays^6^, Patricia Herman^4^

^1^RAND Corporation, Boston, USA, ^2^RAND, Pittsburgh, USA, ^3^RAND, Boston, USA, ^4^RAND, Santa Monica, USA, ^5^Brigham and Women’s Hospital, Boston, USA, ^6^UCLA, Los Angeles, USA

#### Aims

Whole-person health (WPH) involves multiple domains (e.g., physical, mental, and spiritual). To date, however, there is little consensus on which domains should be included in WPH. To inform the structure and measurement of WPH, we conducted a scoping review of conceptual domains and existing WPH measurement instruments.

#### Methods

Peer-reviewed articles and grey literature published from January 2014 to December 2023 that included a theoretical model or empirical measure of self-reported “whole person health” were reviewed. Theoretical/conceptual sources and empirical studies with observational or intervention study designs, including adults 18 or older, were eligible for inclusion. Studies focusing on pediatric populations, educational and personality constructs, and whole health systems of care without mentioning WPH were excluded.We searched five databases (Pubmed, CINAHL, PsycINFO, ERIC, Web of Science) and Google Scholar for peer-reviewed articles and grey literature published in English. Two research team members screened articles and extracted study characteristics. Results describe WPH conceptual domains, published self-report measures, and their psychometric properties.

#### Results

Our search identified 1,143 unique sources, with 29 deemed eligible for review. Eleven conceptual articles mentioned four to six of seven total WPH domains each: biological/physical, behavioral/mental, social, environmental, spiritual, socioeconomic, and individual/other. Our search identified six WPH measures. All six WPH measures included assessments of the biological/physical, behavioral/mental, social, and spiritual domains, and all the conceptual WPH domains were assessed by at least one self-report measure. The self-report measures strongly emphasized the assessment of spirituality and individual domains relative to the conceptual models. They were less likely to include assessments of environmental and socioeconomic domains.

#### Conclusion

This scoping review provides a greater understanding of the domains involved in WPH as a multidimensional construct. Although no existing WPH measures are suitable for broad use, their structural commonalities imply that WPH measures development efforts should consider assessing physical, mental, social, spiritual, and individual domains. The Patient Reported Outcomes Measurement Information System (PROMIS) collection of measures may represent an ideal starting point for developing a comprehensive WPH instrument.

**Table 1:**
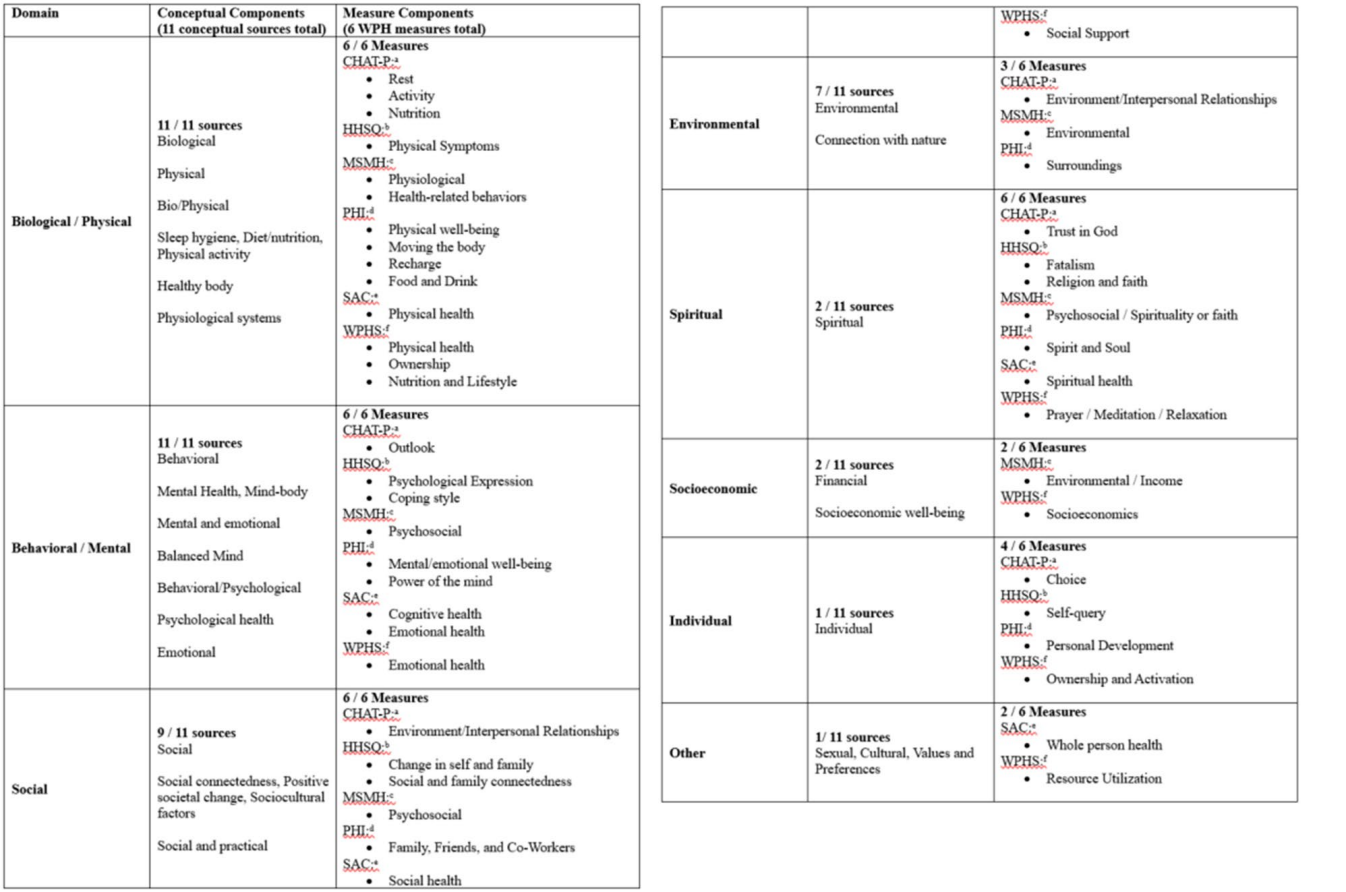
Concordance Between Conceptual and Empirical Components of WPH. Note: ^a^ CREATION Health Assessment Tool for Patients, ^b^ Holistic Health Status Questionnaire, ^c^ MyStrengths+MyHealthapp. ^d^ Personal Health Inventory, ^e^ Self-Assessment of Change, ^f^ Whole Person Health Score

### Use of the Patient-Reported Outcomes Measurement and Information System (PROMIS) to estimate whole-person health

David Cella^1^

^1^Northwestern University, Chicago, USA

#### Aims

Whole-Person Health (WPH) is a complex, multidimensional concept that encompasses a wide range of self-reported health domains and social, environmental, and behavioral factors that influence health. The self-reported health domains can be considered effect indicators of a “health” latent variable. In contrast, the social, environmental, and behavioral factors (such as nutrition, wealth, neighborhood safety, resource utilization, etc.) can be regarded as determinants of health or causal indicators. This presentation aims to evaluate the extent to which PROMIS can contribute to measuring WPH.

#### Methods

The PROMIS domain framework of physical, mental, and social self-reported health was examined to assess its coverage of the domains typically considered in the “effect” components of the WPH concept. The PROMIS domain framework was based on the World Health Organization’s expanded definition of health as “a state of complete physical, mental, and social well-being and not merely the absence of disease and infirmity.

#### Results

All relevant self-reported WPH domains that reflect effect indicators of health are included in the PROMIS domain framework. Physical, mental, social, and spiritual dimensions of self-reported health are all reflected in depth across the approximately 100 PROMIS item banks. In addition, summary health measures already developed combine these dimensions into summary effect indicators. Based on its grounding in mixed method development and item response theory modeling, PROMIS contains rigorous measurement of the physical and mental health-related quality of life domains necessary to evaluate the health of an individual receiving medical care. In addition, existing summary measures reflect a single score to locate people on a WPH continuum.

#### Conclusion

PROMIS provides a good starting point for developing a measure of WPH. PROMIS provides a sustainable ontological structure to enable coherent standard measurement of WPH. The availability of several summary measures across PROMIS domains has been developed and can provide a summary assessment of the effect indicators of WPH.

### Empirical support for an underlying whole-person factor

Ron Hays^1^

^1^UCLA Department of Medicine, Los Angeles, USA

#### Aims

There is increasing interest in measuring “whole person” health (WPH) and deriving an overall summary score. Underlying physical and mental health dimensions have been found consistently in prior studies of self-reported health, but it is unclear whether a single underlying health factor is supported across health domains. The objective of this study is to assess whether there is empirical support for a single summary WPH score from multiple health-related quality of life scales assessing physical, mental and social functioning and well-being.

#### Methods

We examine the dimensionality of nine domains from the Patient-Reported Outcomes Measurement Information System (PROMIS®)-29 + 2 profile measure, the PROMIS social isolation scale, the Personal Wellbeing Index, and the EQ-5D-5L preference score in a sample of 1256 adults with back pain in the United States. A bifactor model was evaluated that included all measures loading on the general factor, 3 loadings on a physical health group factor (physical function, pain interference, pain intensity), and 4 loadings on a mental health group factor (depression, personal well-being,anxiety, social isolation). We assess model fit using the comparative fit index and the root mean square error of approximation (RMSEA).

#### Results

The mean age was 55 (range 18–94), 52% female, 74% non-Hispanic White, 61% were married or living with a spouse, and the highest level of education completed for 35% of the sample was a high school degree or general education diploma. The sample reported substantially more pain intensity, pain interference, and worse physical function than the U.S. general population. Product-moment correlations among the measures ranged from 0.25 to 0.83 (median correlation = 0.52). A bifactor model showed that a general health factor accounted for most of the covariation among measures, but physical function, pain interference, and pain intensity loaded slightly more on the physical health group factor than on the general health factor.

#### Conclusion

The analyses support combining multiple aspects of self-reported health into an overall indicator of whole-person health. Future research is needed to put this work in the context of emerging interest in WPH.

### Health Utility Measures to Estimate Whole-Person Health

David Feeny^1^

^1^McMaster University, Hamilton, Ontario, Canada

#### Aims

Examining the implications of whole-person health for developing preference-based measures of whole-person health.

#### Methods

The breadth of the concept of whole-person health has implications for the development of descriptions of health states (for direct assessment of preferences for health states) and for the creation of new multi-attribute utility functions (MAUFs) for the description and valuation of whole-person health states.

#### Results

There will likely be preference interactions among the attributes (domains) included in a MAUF for whole-person health. In general, preference complementarity has often been observed among legacy preference-based MAUFs. For example, the decrement in the utility for impairments in two attributes is less than the sum of the decrements associated with each impairment independently. The breadth of the concept of whole-person health may require the development of more complicated MAUFs.

#### Conclusion

Developing reliable and valid preference-based measures for whole-person health states will require substantial conceptual effort and careful empirical work. Examples of approaches used for similar previous challenges will be discussed.

## Symposium 6 From outset to outcome - The pertinence of baseline severity and possibility of time dependence in defining meaningfulness of change

Moderator: Jessica Roydhouse^1^

^1^Menzies Institute for Medical Research, University of Tasmania, Hobart, Australia

**Overview**

Estimating the meaningfulness of change on a clinical outcome assessment (COA) requires careful consideration of the context of use. Principally this is understood with reference to the disease or condition that characterizes the patient population of interest. However, other contextual factors may need consideration in ensuring meaningful change evidence generated in evaluation of a treatment is valid and credible.For example, the patient population of interest may enter the study with varied/heterogeneous severity on the domain being assessed (symptoms, impact, etc.). This variability may influence how patients experience and interpret the meaningfulness of their subsequent treatment response. Recognition of this possible source of variability and its potential impact on meaningful change thresholds is reflected in the FDA’s 2023 draft guidance on evaluating the meaningfulness of treatment benefit. However, the form, extent and consequence of any impact of baseline severity on perceptions of meaningful change remain unclear. A related consideration is the length of the assessment period following baseline, particularly when disease severity or treatment tolerability may fluctuate over time. Published research on the impact of assessment interval on clinically meaningful thresholds is sparse. However, what evidence has been generated suggests that duration of follow-up is a factor and that it may differentially impact estimates of meaningful improvement compared to meaningful worsening. Notably, this research has to date focused on limited assessment cycles. As we move towards increased adoption of daily diaries and digital measurements, new evidence is needed to understand how longitudinal variability influences patients’ perceptions of meaningful change, i.e., whether meaningful change thresholds are time-dependent. This symposium will offer an in-depth and evidence-based exploration of these contextual factors and their impact on the assessment (and by extension, interpretation) of meaningful change in evaluations of clinical interventions. Bringing together speakers from academia, industry and regulatory agencies, this session will provide new insights and seek to foster on-going discussion on how we can ensure the evidence we generate on the assessment of treatment impact is accurate, relevant, and reflective of demonstrable benefit to the intended patient population. The session will conclude with an audience Q&A.

**Individual Presenters**

### Estimating Meaningful Score Differences to Support the Interpretability of Clinical Outcome Assessment Scores in Clinical Trials

Monica Morell^1^

^1^US Food and Drug Administration, Silver Spring, USA

#### Aims

Interpreting clinical outcome assessment (COA) scores can be an integral but challenging aspect of medical product development. When COA scores are used to construct endpoints in clinical trials, it is necessary to understand how well the results of the COA-based endpoint correspond to a treatment benefit that is meaningful to patients. The US Food and Drug Administration’s Patient-Focused Drug Development (PFDD) draft Guidance 4 describes two families of methods: meaningful score differences (MSDs) and meaningful score regions (MSRs). The MSD approaches are a collection of methods which aim to translate differences on COA scores into differences in patients’ experiences. The current work aims to introduce MSDs and provide an illustrative example to aid in the application of these approaches.

#### Methods

Data were simulated for a change-from-baseline COA-based endpoint using a fictional, multi-item COA, based on a parallel groups study design. Additionally, data simulated for a patient global impression of severity (PGIS) anchor measure was used to demonstrate how a range of MSDs may be estimated using anchor-based methods. The effect of baseline dependency on the range of MSD estimates is explored.

#### Results

A range of MSD values were estimated to aid in the interpretation of the COA scores. Descriptive statistics and empirical cumulative distribution function (eCDF) curves are presented for the COA scores.

#### Conclusion

MSDs and MSRs are two families of approaches which may be used to relate the less familiar metric of COA scores to patients’ experiences that are better understood. The current work uses simulated data to demonstrate how MSDs may be estimated using anchor-based methods. The resulting MSDs can create a richer context for understanding the meaningfulness of endpoint scores.

### Challenges and Opportunities in Estimating Meaningful Change Thresholds: An Industry Perspective

Mary Kay Margolis^1^

^1^AstraZeneca, Gaithersburg, USA

#### Aims

Capturing and amplifying the patient voice in the development of new clinical therapies is vitally important. Interpretation of These efforts have accelerated over the past 15 years, as regulatory agencies underscore the importance of patient-reported outcomes (PROs) in clinical trials. Within-patient meaningful change thresholds (MCTs) aid in the interpretation of PRO results and understanding of the treatment response in the trial. This talk will offer an industry-based perspective on the importance of estimating MCTs in consideration of context of use and will explore the opportunities and challenges in doing so.

#### Methods

This presentation will leverage real-world case studies from clinical research programs to illustrate how industry practice approaches defining and refining the context of use, with focus on patients’ disease stage, baseline severity, and expected progression over time. The presentation will discuss the challenges of leveraging precedence while balancing risk aversion and risk tolerance. Learnings from this applied experience will be shared, including the pragmatic considerations involved in estimating and applying MCTs within specific contexts of use.

#### Results

This talk will present applied experience and learnings in a narrative framing. It will identify key questions for symposium participants and the wider community to collectively explore on the sufficiency of our current approaches to estimating MCTs with clinical trial data in.

#### Conclusion

Our research is more patient-centered than ever but challenges remain, especially in estimating and interpreting MCTs. These challenges can only be effectively overcome through collaboration and shared understanding among all stakeholders.

### Shifting goalposts? Exploring the invariance of within-patient meaningful change thresholds across differing assessment intervals and its interplay with baseline severity

Dara O’Neill^1^

^1^IQVIA, Barcelona, Spain

#### Aims

The interpretability of clinical outcome assessment (COA) scores can depend on the context of their application. Different population characteristics may influence how patients perceive and regard the change they experience. As we work to formalize and align on quantitative methods for estimating meaningful change thresholds (MCTs) on COAs, a key issue is determining what level of contextual specificity is required. Even within populations, baseline severity may vary and influence patients’ perspectives on their own treatment response. Moreover, in examining the onset rate of that response, it remains unclear if MCTs are also contingent on the assessment interval across which change is assessed or how they may vary during the course of treatment. Such variability would have pertinent implications for responder definitions and time-to-event analyses. The present study will leverage real-world clinical trial data to provide new insight on these contextual considerations (baseline severity and assessment interval) and illustrate their potential influence on estimates of within-patient meaningful change.

#### Methods

Data from two Phase III clinical trials in distinct disease areas will be utilized. In each trial, a patient-reported outcome (PRO) measure was collected from baseline through repeated follow-up assessment (up to 28 timepoints). Within-patient MCTs (for change from baseline) will be estimated for both PRO measures using anchor-based methods, supplemented with distribution-based strategies, for iteratively longer assessment intervals. MCTs estimates across these intervals will be examined for the overall trial populations and for patient subsets stratified by their severity status at baseline.

#### Results

For the PRO measures in each trial, variability in MCT estimates across different assessment intervals and baseline severity strata will be presented, alongside evidence on the interaction between these contextual factors. Longitudinal trajectories in MCT estimates will be described to investigate the evidence of longitudinal trends, and whether these can be differentiated from measurement error.

#### Conclusion

Understanding the potential impact of contextual factors on estimates of meaningful change is essential to ensuring our analytic strategies for patient-centered research are truly fit-for-purpose. The illustrative evidence presented in this study will inform on-going efforts to refine these strategies.

### Meaningful Score Differences, Meaningful Change: What Does it All Mean?

Jessica Roydhouse^1^

^1^Menzies Institute for Medical Research, University of Tasmania, Hobart, Australia

#### Aims

By amplifying the patient voice, clinical outcome assessments (COAs), and more specifically patient-reported outcome (PRO) measures, play a pivotal role in clinical research. As the basis for determining the meaningfulness of change to patients, PRO measures offer unique insight into the direct impact and benefit of treatments amongst those they are intended to help. However, there remains marked heterogeneity in how we define and therefore measure the meaningfulness of treatment response as perceived by patients. Patient engagement in this process has also been variable. This talk will seek to bring clarity to these sources of variability and divergence, by clarifying the aims and assumptions that underpin our efforts to establish such interpretability guidance for PRO measures and other COAs.

#### Methods

This presentation will comprise a narrative overview of the aims, terminology, and analytic approaches utilized in meaningful change research, including the role of anchor-based and distribution-based methods, and the overarching assumptions that inform such research efforts. The presentation will also cover important conceptual and measurement issues including patient engagement, direction of change, and the assessment interval.

#### Results

Points of convergence and agreement in terminology will be highlighted, with the aim of establishing a common language for the discussion of meaningful change. Key considerations in defining the context of use for COAs will be identified, and the specificity required for establishing valid and defensible thresholds for interpreting clinically meaningful response on these measures will be explored. Factors that may inform this specificity will be discussed, including the degree of heterogeneity in the target patient population(s), and the pattern/directionality of treatment response expected.

#### Conclusion

Frameworks for describing and assessing the meaningfulness of treatment impact remain diverse and the target of debate. By elucidating the assumptions that underpin these frameworks and defining key terms and assumptions, we can more effectively seek to foster greater precision and utility in this important area of clinical research.

## Symposium 7 Advancing PRO integration in Oncology Drug Development: Challenges and Complexities

Moderator: Bill Byrom^1^

^1^Signant Health, Nottingham, UK

**Overview**

Patient-reported outcomes (PRO) data enrich oncology research by providing insights into the patient experience, which are crucial for understanding cancer’s full impact. They also support patient-centered care, regulatory submissions, and health policy decisions. However, in cancer clinical trials, there is a notable disparity in the implementation of PROs, and they are underused in early-stage drug development. This symposium will explore the challenges of implementing PROs in oncology research and propose solutions based on recent regulatory guidelines.

Although there is a growing recognition of the importance of PROs in oncology drug development, product labeling based on PROs remains low. This session reviews recent oncology product labels, focusing on inconsistencies in PRO-related value statements and their impact on decision-makers, especially payers. It explores the role of PROs in labels and alternative promotion strategies aligned with FDA guidelines, including peer-reviewed publications and product websites.

We will explore evidence showing that clinicians tend to underestimate both the frequency and severity of adverse events compared to patient reports, highlighting the necessity of capturing the patient voice directly. With reference to the FDA guidance on core PROs in cancer trials, we will examine challenges of its implementation, with particular focus on the measurement properties of stand-alone subscales of validated measures, and the impact of more frequent measurement on patient burden.

While PROs offer insights into the impact of disease and treatments, their integration into oncology trials presents challenges. We will share sponsor perspectives on organizational and regulatory hurdles and propose solutions. Additionally, we’ll focus on integrating PROs in early phase trials and measuring tolerability across development programs. Lastly, the importance of partnering with patients to inform PRO strategies and generate value beyond regulatory decisions and product labeling will be emphasized.

This symposium provides pragmatic guidance on implementing PROs in oncology trials, while acknowledging areas that require considerations and further methodological development. Ultimately it advocates for continued integration of patient-reported measures in clinical research to enhance our understanding of treatment effects and patient experiences.

**Individual Presentations**

### Exploring the role of PRO-related value messages in oncology product labeling and promotion

Ari Gnanasakthy^1^

^1^RTI Health Solution, Research Triangle Park, USA

#### Aims

This session aims to review recent examples of oncology product labels, explore the value messages within these labels and their impact on key decision-makers, and propose strategies for maximizing value messages beyond labeling.

#### Methods

The session will review the current state of product labeling in oncology, focusing on recent examples from the FDA and EMA to highlight inconsistencies and gaps. These inconsistencies demonstrate that the value of PRO-related labeling is not uniform, as stakeholders—particularly payers—may perceive some labels as more valuable than others. For value messages not included in labels, we will explore avenues for product promotion that align with FDA labeling guidelines, using oncology-specific examples.

#### Results

Recent reviews of drugs approved between 2010 and 2020 found that only 46 included PRO-related value statements (FDA = 9, EMA = 42), highlighting a lack of consistency in PRO inclusion across labels. While evidence shows that US payers place importance on PRO data, they may consider PRO-related labels of limited use without key information, such as numerical data, clinical significance, and thresholds for meaningful changes. However, surveys of US payers also indicate that they rely on PRO-related messages disseminated through peer-reviewed publications for decision-making. Additionally, examples of promotions containing PRO-related messages on oncology product websites exist, which may be consistent with FDA-required labeling, even though these messages are not included in labels.

#### Conclusion

PRO-related labels in oncology treatments are rare and often lack consistency, which may hinder key stakeholders, especially payers, from making informed decisions. Even in the absence of PRO-related statements in product labels, study sponsors can promote PRO-related value messages that may be consistent with FDA-required labeling for the product, through peer-reviewed publications and product websites.

**Individual Presenters**

### The importance of the patient voice in cancer treatment tolerability assessment, and unanswered methodology questions

Bill Byrom^1^

^1^Signant Health, Nottingham, UK

#### Aims

This session will review published evidence supporting the importance of patient-reported tolerability data in addition to that reported by the clinician, and will explore knowledge gaps associated with the application of the FDA guidance on core patient-reported outcomes in cancer trials.

#### Methods

Differences in reporting frequency and severity of tolerability data between patients and clinicians will be examined using case studies from the literature. The key elements of the FDA guidance on PROs in cancer trials will be overviewed, and methodology questions explored with specific reference to potential patient burden associated with frequent assessment and the use of sub-scales as stand-alone measures. These will be explored with wider reference to the literature, and through invited discussion with the symposium panel members.

#### Results

Several published studies provide evidence that clinicians tend to underestimate both the frequency and severity of adverse events compared to patient reports, highlighting the necessity of also capturing the patient voice directly. The implication of applying the FDA guidance on core PROs in cancer trials changes the measurement strategy for PROs to require more frequent (at home) completion, and to leverage sub-scales of existing instruments as stand-alone measures. This raises questions regarding the impact of more frequent assessment on patient burden, and the impact of context to determine whether sub-scales used as part of a full instrument have the same measurement properties as those used as stand-alone item lists. Little definitive evidence is published, but data on daily completion in cancer patients, and the timings of measure completion, provides a positive signal. Published research on the comparability of sub-scales with stand-alone item lists is less definitive, but on balance provides a broadly supportive signal.

#### Conclusion

Collecting patient-reported tolerability data is vitally important in oncology clinical trials, including early phase development. The FDA guidance provides a framework to enhance the value of PRO data in oncology trials, but more research is welcomed on providing definitive guidelines on understanding the burden associated with frequent measurement, and the comparability of measures delivered as sub-scales or stand-alone item lists.

### Optimizing Impact: Pharma’s Insights on the Benefits and Challenges with Assessment of PROs in Oncology Clinical Trials

Rohini Sen^1^, Bill Byrom^2^, Ari Gnanasakthy^3^

^1^AbbVie, Arlington, USA, ^2^Signant Health, Nottingham, UK, ^3^RTI Health Solutions, New Jersey, USA

#### Aims

With evolving regulatory and reimbursement frameworks, leveraging patient experience data (PED) in oncology drug development is increasingly important. Understanding PEDPED’s opportunities and challenges is crucial for effective patient-reported outcome (PRO) strategies amid rapid treatment advancements and compressed timelines. This session will highlight sponsor perspectives on integrating PED, focusing on PROs in early phase oncology trials, specifically examining how PROs guide dosing decisions and generate value beyond traditional medical product labeling. Lastly, this session will underscore the importance of involving patients as partners throughout the drug development process to execute PED strategies successfully.

#### Methods

Developing robust PRO strategies for early- and late-phase oncology trials requires generating evidence that meets the needs of multiple stakeholders, including regulators and HTA/payers, while addressing the challenges of aligning diverse functional groups and navigating the complexities of trial designs that integrate and elevate PRO strategies. Methodologically, sponsors should focus on (1) selecting measures for symptomatic AEs and overall bother in early phase trials, (2) engaging patients throughout drug development, (3) educating cross-functional teams on value of PROs, particularly with respect to applications beyond regulatory labeling, and (4) empowering teams such as clinical operations to advocate for PED. These steps are central to navigating innovative and seamless implementation of PROs in oncology trials.

#### Results

Strategies for implementing PROs will focus on leveraging the FDA’s Core PRO Guidance, specifically integrating PRO-CTCAE to measure symptomatic AEs and FACT-G GP5 to assess overall impact of treatment side effects, beginning in early-phase trials. Patient involvement in informing trial design, modular and staggered PRO assessment schedule strategies to meet regulatory demands for more frequent evaluations will also be discussed. Lastly, the session will present approaches to effectively communicate PRO value and recommendations to overcome barriers in operational implementation and data monitoring.

#### Conclusion

The industry plays a critical role in making oncology drug development more patient-centered by integrating PED in clinical trials. By integrating PROs early in drug development and involving patients as partners, there is an opportunity to generate high quality patient-reported data to inform dosing selection and demonstrate treatment benefits/risks on the outcomes that matter most to patients.

**Fig. 1:**
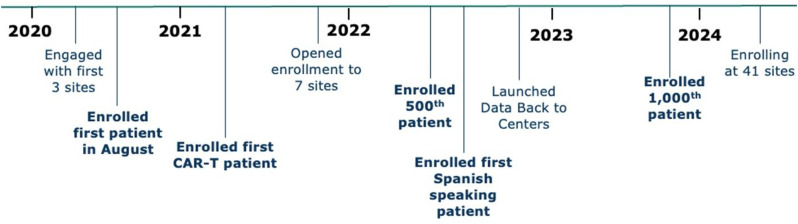
Timeline of CIBMTR PRO Protocol Development

## Symposium 8 Identifying meaningful digital measures and turning them to meaningful endpoints: the challenges and barriers to regulatory approval and industry uptake

Moderator: Bill Simpson^1^

^1^IQVIA, Toronto, Ontario, Canada

**Overview**

Over the last decade, there has been a concerted effort to explore how digital measures (DMs), i.e. endpoints derived from Digital Health Technologies (DHTs), could transform drug development. The potential for DMs remains high, with advantages in sampling frequency, objectivity, scalability and capture of novel health-related behaviours/concepts. There are however numerous challenges, including 1) ensuring DMs measures meaningful aspects of health (MAH); 2) identifying the most appropriate parameters to evaluate the MAH; 3) ensuring that the DMs and selected parameters are fit-for-purpose, per patient-focused drug development (PFDD) guidelines and analytical validation requirements; 4) defining endpoints and analytical approaches that maximize insights from available data.The goal of this symposium is to discuss the role of DMs in clinical trials, outline the scientific considerations for their use, and encourage their strategic integration as trial endpoints. The symposia will examine current practices and existing guidelines for the selection of DMs, formation and validation of endpoints, and analysis of DHT data.The moderator will start the session presenting a general framework for digital endpoint development.The first speaker (DHT scientist) will introduce approaches to identifying candidate MAHs to be measured by DHTs and methods for selecting DHT parameters to quantify those MAHs.The second speaker (statistician/psychometrician) will discuss how intensive longitudinal data obtained by DHTs are typically aggregated to produce trial endpoints that resemble traditional endpoints (e.g., from clinical outcome assessments (COA)). They will also discuss how the richness of DHT data could be exploited to better model clinical change, thereby creating novel endpoints which have the potential be more meaningful to patients.The third speaker (DHT developer) will provide an example of developing a sensor-derived endpoint, showing a practical example of the process introduced by the 3 prior speakers.Finally, the last speaker from the Patient-Focused Statistical Science (PFSS) group (Food and Drug Administration (FDA)) will discuss the development of fit-for-purpose digitally derived endpoints using DHT and COA guidance with examples on common pitfalls.The moderator will then lead a Q&A with the presenters, encouraging audience participation in both offering thoughts and experiences.

**Individual Presentations**

### Maximizing relevance and validity of DHTs for use in clinical trials: conceptual development, validation and fit-for-purpose assessment of endpoints to support regulatory decision-making

Bill Simpson^1^, Belen Rubio^2^, Salma Ajraoui^3^

^1^IQVIA, Toronto, Ontario, Canada, ^2^IQVIA, Barcelona, Spain, ^3^IQVIA, London, UK

#### Aims

Patient-focused drug development (PFDD) encourages sponsors to collect data on meaningful aspects of health (MAH) in clinical trials, using fit-for-purpose (FFP) methods. Digital Health Technologies (DHTs) hold immense potential for transforming the collection of data during clinical trials as they have advantages in objectivity, sampling frequency, and dimensionality (the ability to measure multiple MAH via multiple measures). For example, an accelerometer can measure both activity level (total step count and walking bouts) and sleep behaviour (sleep time, number of nighttime awakenings, etc.) in a single device. Successfully leveraging these digital measures (DMs), as endpoints requires strong alignment between the MAH, the derived disease Concepts of Interest (COIs) and the DMs themselves. The strength of alignment between these constructs dictates the degree to which a proposed endpoint is FFP.

#### Methods

There is no single recommended approach from regulators, payers or the scientific community to guide sponsors in how construct these FFP endpoints. We aim to review and discuss pathways to (a) selecting MAHs and parameters, (b) defining endpoints using DMs, and (c) assessing overall FFP of these novel endpoints. Published DHT guidance from both the FDA and EMA was reviewed and the literature was searched for consortium recommendations.

#### Results

Two pathways to selecting MAHs and DMs were identified: a patient-centric approach (which allows patients to specify the most relevant MAH and most interpretable DMs from their perspective), and a data-centric approach (which identifies the MAHs and DMs which statistically offer the most sensitivity and specificity in the population). Both have benefits and drawbacks from a scientific and regulatory perspective. A hybrid pathway, containing both patient and data centric elements in some combination, may be most appropriate. All 3 approaches will be discussed.

#### Conclusion

A hybrid approach may present distinct advantages when developing novel, FFP, digital endpoints. The patient centric elements ensure strong alignment with patient MAH while the data centric components maximize available signal and focus on strong psychometrics. As sponsors continue to explore the utility of these DHT based endpoints, further scientific advances and regulatory interactions will continue to help refine these approaches and elucidate best practices.

### Defining and analyzing digital endpoints: a review of current practices with examples from cough, sleep, and physical activity

Aleksandra Sjöström-Bujacz^1^, Gerasimos Dumi^2^, Konstantina Skaltsa^3^

^1^IQVIA, Stockholm, Sweden, ^2^IQVIA, Athens, Greece, ^3^IQVIA, Barcelona, Spain

#### Aims

Digital health technologies (DHTs) frequently generate numerous parameters measured continuously over a given period. Accordingly, DHTs generate large amounts of data which could be used in multiple ways to inform study endpoints. For example, counting steps over 7 days could be synthesized for an endpoint as average number of steps per day or total number of steps over 7 days etc. Each endpoint definition will require different statistical considerations, including the allowability of missing data, the level of aggregation, and the statistical analysis. We aim to present and discuss common ways of combining DHT data and the impact of each on analytical considerations.

#### Methods

Using the Library of Digital Endpoints, hosted by the Digital Health Measurement Collaborative Community (DATAcc) by the Digital Medicine Society (DiMe), we reviewed currently used endpoints for cough, sleep, and physical activity measured using DHTs. We examined which parameters informed the endpoint, if and how parameter data were aggregated, how missing data were handled, and how these were statistically analyzed.

#### Results

The review indicates that DHTs informing cough, sleep, and physical activity endpoints were commonly based on aggregation of parameters over certain periods of time. The rationale for the selection of aggregation periods and rules for acceptable missing data were frequently omitted from endpoint definitions. As a result of the aggregation, statistical analysis of the endpoints employed standard methods. We did not identify clinical trials where data were analyzed without aggregation, e.g., by employing statistical methods that allow for analysis of continuous trends in data, although these may have been performed for exploratory purposes and not made available.

#### Conclusion

Due to the relative novelty of digital endpoints, sponsors need to carefully consider the rationale for selecting parameters, the potential aggregation periods, the missing data rules, the endpoint definition and the statistical analysis that will best answer their research question. While aggregation brings measures to familiar formats and may enhance interpretability and communication of findings to all audiences, the richness of the collected data (and therefore opportunities to better understand aspects of the disease and define potentially more meaningful endpoints in future trials) is lost through aggregation.

### A Data-Guided Framework for Developing a Meaningful Digital Endpoint based on Wearable DHTs

Christine Guo^1^

^1^ActiGraph, Pensacola, USA

#### Aims

Wearable sensors offer potential advantages over traditional COA instruments in their temporal resolution, robustness to subjective and recall bias, and minimal burden to the users. The use of sensor-derived measures as clinical endpoints, however, presents methodological challenges as the source data, continuous timeseries data from the sensor, require data processing and biostatistical approaches distinct from the processing of discrete questionnaire data. Our aim is to develop a tailored framework for sensor-derived endpoints to address these methodological gaps while adhering to the established guidance for developing and validating meaningful clinical endpoints.

#### Methods

We identify the key differences between the processing steps to derive sensor-based endpoints versus traditional questionnaire-based endpoints. We then adapted the FDA’s Patient-Focused Drug Development Guidance 3 framework to address these key difference and challenges presented by sensor data. We then use a large-scale publicly available wearable dataset as part of the National Health and Nutrition Examination Survey (NHANES) to demonstrate key methodological steps needed to optimize the measurement properties of sensor-derived digital outcome scores.

#### Results

We recommend technical feasibility to be considered as part of the initial stage and identify candidate digital measures that can capture the relevant concept of interests. We recommend a systematic evaluation of the measurement properties of candidate digital measures to inform measure selection and statistical approach to derive digital endpoints, including construct validity, reliability, and bias to data missingness. We demonstrate the impact of key parameter selections on the measurement properties of sensor-derived endpoints using wearable data collected from NHANES study and the value of this data-informed process to minimize the risk of suboptimal endpoint construction from continuous wearable data.

#### Conclusion

This work highlights key technical and statistical consideration in developing novel sensor-derived endpoints that are not specifically addressed in current regulatory guidance. The uniqueness of the sensor-derived measure requires data-informed steps to ensure successful uses of the big but complex timeseries data collected from wearable DHTs.

### Meaningful and Interpretable Endpoints

Seung Won Chung^1^

^1^FDA, Silver Spring, USA

#### Aims

The aim of this talk is to discuss the development of fit-for-purpose endpoints using data from digital health technologies (DHTs). It will highlight key considerations when using DHTs in clinical investigations, such as identifying a meaningful aspect of health and concept of interest, defining the concept of use, selecting or developing clinical outcome assessments (COAs), evaluating fitness-for-purpose, and finally developing an endpoint.

#### Methods

Three key FDA guidances form the basis of this discussion: 1) Digital Health Technologies for Remote Data Acquisition in Clinical Investigations; 2) Patient-Focused Drug Development [PFDD] draft Guidance 3: Selecting, Developing or Modifying Fit-for-Purpose Clinical Outcomes Assessments; and 3) PFDD draft Guidance 4: Incorporating Clinical Outcome Assessments into Endpoints for Regulatory Decision-Making. Examples specific to DHTs are included to guide the development of fit-for-purpose endpoints.

#### Results

PFDD draft Guidance 3 outlines an evidence-based rationale describing eight components to consider when supporting or justifying the appropriateness of a COA for a specific context of use. These principles are largely applicable to DHTs. Furthermore, PFDD Draft Guidance 4 provides detailed discussions of the requirements for endpoints derived from data collected using DHTs. These align with the standards for any clinical trial endpoint intended to support labeling claims.

#### Conclusion

Although such endpoints present unique challenges and considerations, the existing PFDD guidance series remains broadly applicable. Establishing connections between these guidance and the relevant FDA documents on DHTs is essential.

## Symposium 9 Cooperative Agreement-based Development & Validation of the Symptom Assessment for Bronchiectasis (SABRE) for FDA Qualification: Insights Gained and Lessons Learned from FDA Research Grants to Support Drug Development Tools

Moderator: Daniel Serrano^1^

^1^The Psychometrics Team, Ltd., London, UK

**Overview**

This symposium will consist of three presentations detailing the clinical development need for NCFBE-specific endpoints useful in efficacy determination, the qualitative development of the SABRE, and the psychometric validation of the SABRE. Each presentation will describe research considerations, methods, and results. In addition, each presentation will discuss key insights gained from conducting research under this funding mechanism. For example, the modern psychometric methods approaches for determining domain specification and scoring preferred under this mechanism will be discussed and contrasted with alternative approaches. This aspect of the symposium will help disseminate to the field strategies useful in collaborating with FDA to successfully execute qualification program grants. Because the funded research was conducted during the COVID-19 pandemic; challenges posed and novel solutions employed will also be discussed; this includes the real limitations of the yet-to-be fulfilled promise of remote digital recruitment often preoccupying the field. The symposium will be structured with the following three presentations and time allotments Presentation 1, Kevin Mange, MD, MSCE, Insmed The Clinical and Drug-Development Program Needs Motivating The Development of Novel PRO-Based Endpoints in Bronchiectasis Presentation 2, Kelly McCarrier, PhD, OPEN Health GroupQualitative Instrument Development and Content Validation of The Symptom Assessment for Bronchiectasis (SABRE)Presentation 3, Daniel Serrano, PhD, The Psychometrics TeamPsychometric Validation of The Symptom Assessment for Bronchiectasis (SABRE): Design, Sampling, Modern Methods, & Classical methods evidenceSymposium order and time allocations:1.Moderator will frame the symposium; 10 minutes 2.Presentation 1; 15 minutes 3.Presentation 2; 15 minutes 4.Presentation 3; 15 minutes 5.Discussion/Q&A; 20 minutes Total: 75 minutes Methods: Results: Conclusion:

**Individual Presentations**

### The Clinical and Drug-Development Program Needs Motivating The Development of Novel PRO-Based Endpoints in Bronchiectasis

Kevin Mange^1^, Mariam Hassan^1^, Kelly McCarrier^2^, Daniel Serrano^3^

^1^Insmed, Bridgewater, USA, ^2^OPEN Health, Bethesda, USA, ^3^The Psychometrics Team, London, UK

#### Aims

To characterize the clinical and drug-development program needs motivating the development of the Symptom Assessment for Bronchiectasis (SABRE), assessing key patient-relevant symptoms of non-cystic fibrosis bronchiectasis (NCFBE) with and without nontuberculous mycobacteria (NTM) infection.

#### Methods

Disease Background and Challenges: Bronchiectasis is a suppurative lung disease with heterogeneous phenotypic features. Patients typically have a chronic cough with daily sputum production, but may also have complaints of rhinosinusitis, fatigue, and/or hemoptysis. Bronchiectasis is diagnosed also based on axial images of high-resolution chest computed tomography (HRCT) scans. Studies in bronchiectasis reveal colonization with familiar pathogens such as Haemophilus sp., Pseudomonas aeruginosa and Moraxella sp. In general, the presence of Pseudomonas aeruginosa has been associated with worse lung function, and more exacerbations. Beyond these bacterial organisms, infection by non-tuberculous mycobacteria (NTM) organisms, such as Mycobacterium avium complex (MAC), is of particular clinical significance for patients with bronchiectasis. There are no approved therapies in the US for bronchiectasis. This is despite the efforts to develop investigational agents using exacerbation-related events as primary endpoints. Although there has been an attempt to use PROs to evaluate the efficacy of inhaled antibiotics, these trials also have either failed to detect statistically significant effects or have not shown consistent results.

#### Results

These and related challenges motivating the development of new endpoints designed to measure bronchiectasis symptoms with precision will be reviewed and discussed.

#### Conclusion

The development of a novel PRO instrument that is advanced to the stage of readiness to be included in an interventional trial to allow qualification for drug development and regulatory decision making would be a significant advancement and innovation for patients. It would enable the development of new therapies for patients with these diseases using an instrument that reliably assesses the disease-related symptoms that dominate patients’ quality of life (QoL).

### Qualitative Instrument Development and Content Validation of The Symptom Assessment for Bronchiectasis (SABRE)

Kelly McCarrier^1^, Jui-Hua Tsai^2^, Nancy Touba^3^, Daniel Serrano^4^, Mariam Hassan^5^, Kevin C. Mange^5^

^1^OPEN Health, Seattle, USA, ^2^OPEN Health, Bethesda, USA, ^3^OPEN Health, London, UK, ^4^The Psychometrics Team, London, UK, ^5^Insmed Incorporated, Bridgewater, USA

#### Aims

To complete qualitative research activities to develop the Symptom Assessment for Bronchiectasis (SABRE), a new patient-reported outcome (PRO) instrument. Developed to assess key patient-relevant symptoms of non-cystic fibrosis bronchiectasis (NCFBE), the SABRE is intended to support measurement of clinical trial efficacy endpoints among patients with and without co-occurring nontuberculous mycobacteria (NTM) infection. The study was conducted during the COVID-19 pandemic; challenges posed and solutions employed will be discussed.

#### Methods

Qualitative concept elicitation (CE) and cognitive interviews (CI) were conducted with US-based adult participants diagnosed with NCFBE, audio-recorded, and transcribed. The semi-structured CE interviews elicited spontaneous reports of NCFBE symptom experiences and used probing and rating exercises to further explore and confirm concepts. Interview transcripts were coded for qualitative content analysis using Atlas.ti. CE interview data were considered alongside review of existing scales, published literature, and input from clinical experts and patient advisors during an item-generation meeting (IGM) to develop draft SABRE item content. The draft content was refined through four iterative waves of CIs to evaluate the comprehension and relevance of SABRE’s item wording, recall period, and response options among patients with NCFBE.

#### Results

Participants in CE interviews (N=40; 22 with NTM co-infection) expressed 69 different symptoms associated with their NCFBE. Evidence of concept saturation was observed in both NTM cohorts. Cough (85%) and mucus/sputum production (78%) were the most frequently-reported symptoms (Figure 1). CE data considered during the IGM supported drafting of SABRE items to assess frequency of 5 key NCFBE symptoms: cough, sputum/mucus production, chest congestion, shortness of breath, and fatigue. CIs (N=20) supported refinement to the draft SABRE content and confirmed the comprehension of item wording, relevance of included symptoms, and interpretability of a 5-point verbal response scale (“never” to “almost always”).

#### Conclusion

The SABRE is a newly developed and refined 5-item daily diary PRO measure that effectively captures the key symptoms of NCFBE from the patient’s perspective. This qualitative study informed key symptoms, supported appropriate item wording, and established the content validity of the SABRE ahead of further evaluation of the instrument’s scoring and measurement properties in a non-interventional validation study.

**Fig. 1 (Symposium 9.3):**
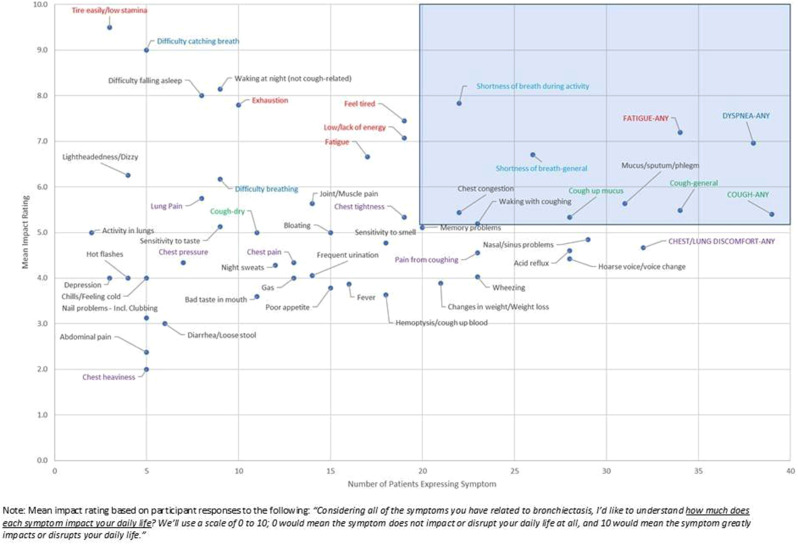
Prevalence of Symptom Concept Expression by Mean Impact Rating. Note: Mean impact rating based on participant responses to the following: “Considering all of the symptoms you have related to bronchiectasis, I’d like to understand how much does each symptom impact your daily life? We’ll use a scale of 0 to 10; 0 would mean the symptom does not impact or disrupt your daily life at all, and 10 would mean the symptom greatly impacts or disrupts your daily life”

### Psychometric Validation of The Symptom Assessment for Bronchiectasis (SABRE): NIVS Design & Sampling; Modern & Classical Psychometric Evidence

Daniel Serrano^1^, Medha Satyal^2^, Lauren Podger^1^, Sam Parsons^3^, Jack Wakefield^4^, Tim-Hong Hoe^3^, Kelly McCarrier^5^, Mariam Hassan^6^, Kevin Mange^6^

^1^The Psychometrics Team, Ltd., London, UK, ^2^Open Health Group, Bethesda, USA, ^3^Open Health Group, London, UK, ^4^Formerly of Open Health Group, London, UK, ^5^Open Health Group, Seattle, USA, ^6^Insmed, Inc., Bridgewater, USA

#### Aims

To complete the Psychometric Validation of the Symptom Assessment for Bronchiectasis (SABRE), assessing key patient-relevant symptoms of non-cystic fibrosis bronchiectasis (NCFBE) with and without nontuberculous mycobacteria (NTM) infection. The study was conducted during the COVID-19 pandemic; challenges posed and solutions employed will be discussed.

#### Methods

The draft SABRE instrument emerging from Phase 1 of the grant (qualitative phase) consisted of 5 daily diary items assessing past 24-hour frequency of cough, sputum production, congestion, dyspnea, and fatigue with response options of “never” (1), “rarely” (2), “sometimes” (3), “most of the time” (4), and “almost always” (5). Phase 2 of the grant consisted of a non-interventional validation study (NIVS) for SABRE. Sampling was conducted from four clinical sites. NIVS design consisted of 7 days of SABRE daily diaries in both baseline and follow-up weeks, separated by a 14-day retest interval.Modern psychometric methods (MPMs) were employed to test structural validity, including domain specification (C2 RMSEA-based model fit), local dependence (LD, Chen’s G2), and dimensionality and scoring statistics (e.g., McDonald’s). Uniformity of structural validity between the NTM+ and non-NTM population was evaluated via differential item functioning (DIF; Wald DIF Sweep) and differential test functioning (DTF; e.g., expected test score standardized difference [ETSSD]). Classical methods evaluated the reliability (e.g., Cronbach’s a) and validity (convergent and known-groups) of SABRE scores at baseline. Test-retest reliability (TRTR) was estimated via intraclass correlation coefficient (ICC[2,1]) among stable participants (no change in PGIF in the 14-day retest interval).

#### Results

Data collection was completed in 7 months; Sampling flow is presented in Figure 1. MPM evidence for SABRE demonstrated no LD or DIF/DTF for the final bifactor structure and scoring/dimensionality statistics supported a weekly average total sum score for the SABRE. The SABRE demonstrated strong internal consistency (a: 0.78), TRTR (ICC[2,1]: 0.87), and convergent validity (range: 0.68 - 0.79). Known-groups validity was demonstrated across PGIF groups.

#### Conclusion

The SABRE is psychometrically robust, and with 5 items was found to explain 81% of the variance in the 14-item EXACT PRO scores at cross-section. Per funding requirements, future work will estimate responsiveness in an interventional study.

**Fig. 1 (Symposium 9.4):**
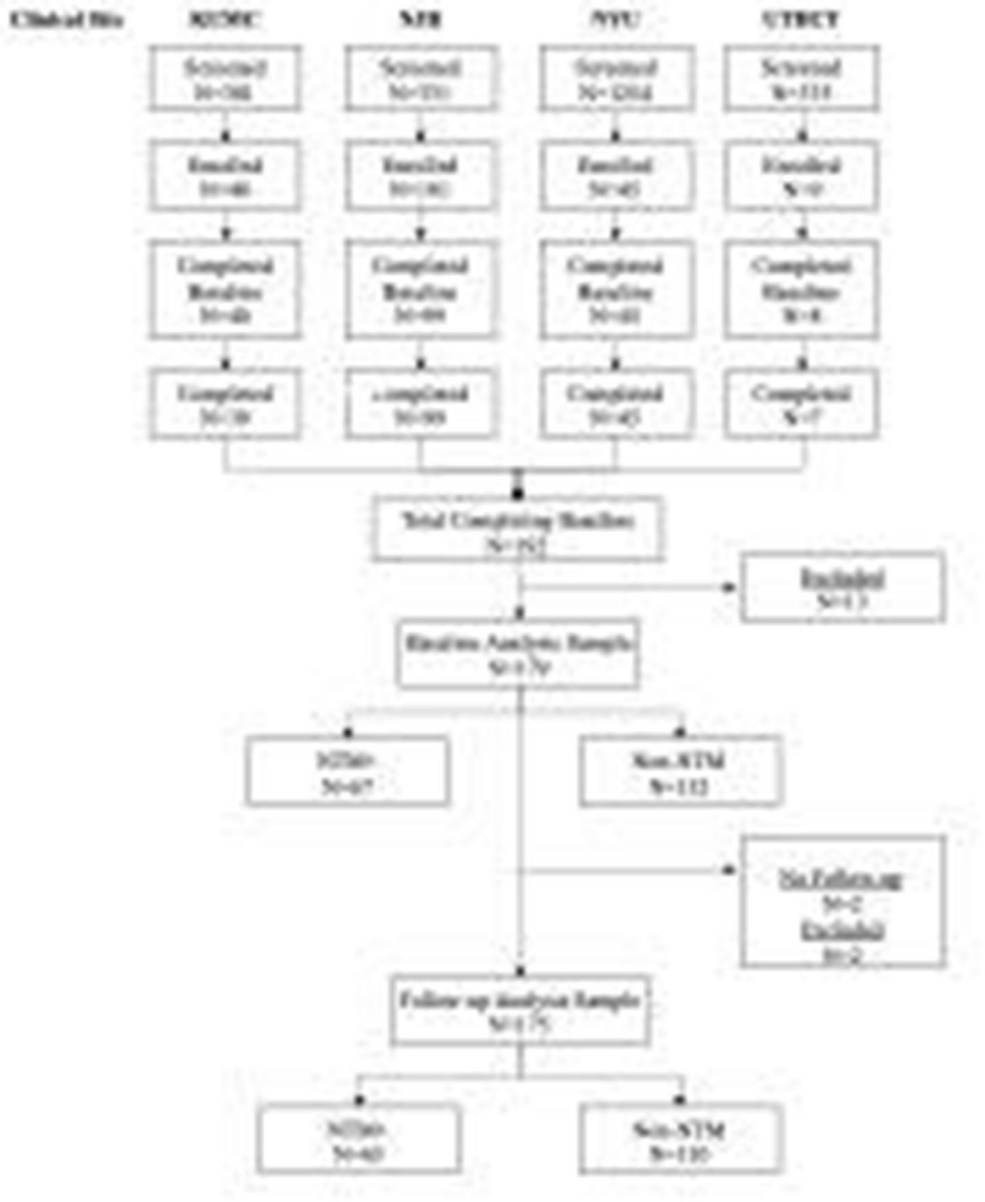
Psychometric Validation of The Symptom Assessment for Bronchiectasis (SABRE): NIVS Design & Sampling; Modern & Classical Psychometric Evidence

## Symposium 10 The Value of Patient-Reported Outcomes Research in the Modern Treatment Landscape in Hematology

Moderator: Fabio Efficace^1^

^1^Italian Group for Adult Hematologic Diseases (GIMEMA), Data Center and Health Outcomes Research Unit, Rome, Italy

*Journal of Patient-Reported Outcomes 2026*, **10(Suppl 1)**:Symposium 10

**Overview**

Notable advances have been made in the treatment of patients with hematologic malignancies over the last two decades such that the life expectancy of patients with some diseases (i.e., chronic myeloid leukemia) now approach that of their peers in the general population. As patients are living longer, hematologists and patients face challenging choices regarding the best treatment approaches, given most published endpoints demonstrate similarities in treatment options with respect to safety or clinical effectiveness. Several novel drugs have been developed as oral agents designed for patients to take for several years, or even lifelong in some diseases, which introduces an additional challenge of ensuring an optimal adherence to therapy in order to maximize drug effectiveness. Therefore, within this rapidly evolving treatment landscape, patient-reported outcomes (PROs) data have become critical to better inform benefit/risk assessment beyond traditional clinical endpoints for safety and effectiveness studies. In parallel, the use of PROs has become highly relevant not only in clinical trial settings, but also in routine care settings. The proposed Symposium include presenters from across Europe and the US and will provide an overview of how PROs have contributed to treatment advances in hematology, for example, by describing real-world data on the use of PROs across different areas of hematology. It will also describe studies which have shown the added value of PROs in enhancing accuracy of survival prediction, and evidence-based data indicating how digital patient-reported symptom monitoring may facilitate adherence and clinical response to therapy. The successful integration of PROs into large hematopoietic stem cell transplant and cellular therapy patient registries will also be presented.

**Individual Presenters**

### The use of digital patient-reported symptom monitoring to facilitate adherence to therapy and improve clinical response in patients with hematologic malignancies receiving oral therapies

Fabio Efficace^1^

^1^Italian Group for Adult Hematologic Diseases (GIMEMA), Data Center and Health Outcomes Research Unit, Rome, Italy

*Journal of Patient-Reported Outcomes 2026*, **10(Suppl 1)**:Symposium 10

#### Aims

Therapy with tyrosine kinase inhibitors (TKIs) in chronic myeloid leukemia (CML) is generally lifelong for most patients with adherence being a key challenge due to chronic (low-grade) adverse events (AEs). Previous evidence, mainly in patients with solid tumors, showed the beneficial effects of routinely integrating patient-reported symptomatic AEs in routine practice. This presentation will describe the clinical utility of an online monitoring system for patient-reported symptoms in the setting of newly diagnosed CML.

#### Methods

An international pilot trial enrolling patients with newly diagnosed CML planned to receive TKIs was conducted. Patients used a tablet PC to self-rate symptoms before clinical consultations, with graphical results available in real-time to physicians. Adherence was assessed in two ways: via pill count and with the ARMS-7 self-reported questionnaire. Secondary outcome measures assessed at 3 and 6 months included: health-related quality of life (assessed with the FACT-G), fatigue (assessed with the FACT-F), as well as patients’ and physicians’ acceptability. Molecular clinical response was also evaluated at 3 and at 6 months to determine the prevalence of “optimal responders” according to international standard criteria.

#### Results

Between July 2020 and August 2021, 94 newly diagnosed patients with CML were enrolled across the United States and Italy. Pill count adherence analysis showed that >90% of evaluable patients took at least 90% of prescribed TKI therapy during the 6-month observation period. Mean scores on the ARMS-7 self-reported questionnaire indicated high levels of adherence at both 3 and 6 months, irrespective of TKIs. No differences were observed in mean scores of the FACT-G scales and of the FACIT-F questionnaires over time. The online platform was very well accepted both by patients and physicians and the large majority stated this approach improved the quality of communication between them. Findings about drug effectiveness were also considered good, indicating that optimal molecular response was achieved by 87% and 75% of patients at 3 and at 6 months, respectively.

#### Conclusion

Patient-reported symptom monitoring from the beginning of TKI therapy in patients with newly diagnosed CML may be instrumental to facilitate adherence to therapy and improve clinical response.

### Enhancing Survival Prediction Using Patient-Reported Outcomes in Hematologic Malignancies: A Real-World Example from an International Study

Francesco Sparano^1^

^1^Italian Group for Adult Hematologic Diseases (GIMEMA), Data Center and Health Outcomes Research Unit, Rome, Italy

*Journal of Patient-Reported Outcomes 2026*, **10(Suppl 1)**:Symposium 10

#### Aims

There is substantial evidence indicating that patient-reported outcomes (PROs) provide prognostic information for survival across several cancer populations. However, this data has been mainly generated from studies including patients with solid tumors. This presentation will show the independent prognostic value for survival of PROs, beyond well-established laboratory-based risk classifications, in patients with hematologic malignancies (i.e., myelodysplastic syndromes-MDS). We will then also outline how PROs could enhance prognostic accuracy by developing a patient-centric prognostic index.

#### Methods

The current gold-standard risk classification for MDS is the Revised International Prognostic Scoring System (IPSS-R), which is purely based on clinical and laboratory indicators. We will present the prognostic value for survival of baseline (i.e., diagnostic workup) patient’s self-reported fatigue (by the EORTC QLQ-C30) in a sample of 902 patients with MDS enrolled in an international study. We will then describe how a patient-centric prognostic index (FA-IPSS-R) can be developed by incorporating fatigue into the IPSS-R. This newly developed index was externally validated in an independent cohort.

#### Results

In multivariate analysis including the IPSS-R and other key potential prognostic factors, a higher score in the fatigue scale of the EORTC QLQ-C30 was independently associated with overall survival (OS). Based on these findings, a new prognostic index was developed, namely, the FA-IPSS-R. Self-reported fatigue severity was integrated into the two broad IPSS-R risk categories (i.e. lower- and higher-risk), enabling to identify four risk group categories, with statistically significant different OS (p<0.001). The predictive accuracy of this new index was higher than the IPSS-R alone. Substantial OS survival differences were observed amongst the four risk groups derived from this new patient-centric index. For example, the three-year OS for the lowest and the highest FA-IPSS-R risk-group was 76.2% and 17.8%, respectively.

#### Conclusion

Assessment of self-reported fatigue should be included in the diagnostic workup of patients with MDS and considered as a standard baseline stratification factor in future randomized controlled trials. Inclusion of a PRO into a well-established laboratory-based risk stratification is feasible and its use may enhance physicians’ ability to more accurately predict survival.

### The Value of Patient-Reported Outcomes Research in the Modern Treatment Landscape in Hematology

Rachel Cusatis^1^

^1^Medical College of Wisconsin, Milwaukee, USA

*Journal of Patient-Reported Outcomes 2026*, **10(Suppl 1)**:Symposium 10

#### Aims

The Center for International Blood and Marrow Transplant Research (CIBMTR) is a clinical outcomes registry in the United States for recipients of hematopoietic cell transplantation (HCT) and cellular therapies (CT) with longitudinal data collected since the 1970s on >600,000 patients. Recognizing the importance of quality of life (QOL) and the value of PROs, the registry developed an infrastructure to collect PROs on adult registry patients. In late 2021, CIBMTR began providing that data back to clinics in dashboards with the ability to download their center data. This symposium presentation demonstrates current enrollment in the CIBMTR PRO protocol and illustrates how the registry returns data back to centers.

#### Methods

A panel of experts to recommend nine PROMIS domains with preferred delivery through an electronic system to administer CATs, though paper and telephone versions are available to meet modality preferences of all patients. CIBMTR developed an electronic PRO (ePRO) system for routine PRO data collection, under a centralized protocol with all enrollment, consent, and data collection activities performed by CIBMTR. To share PRO data with centers, CIBMTR leveraged an existing portal, Data Back to Centers (DBtC), where clinicians can log in to see aggregate and individual patient-level data for their center.

#### Results

Enrollment into the PRO Protocol began in August 2020 with 3 champion centers. Expansion to other centers was intentionally gradual. As of August 2024, CIBMTR PRO Protocol has enrolled 1299 patients from 45 centers who completed 3584 surveys. Thirty-eight percent of patients enrolled received allogeneic HCT (n=488), 48% of patients received autologous HCT (n=623), and 14% received chimeric antigen receptor T-cell therapy (n=188). The enrollment has remained consistent at 34%. After steady expansion of the adult HCT and CT PRO data collection, CIBMTR leveraged the existing infrastructure to share clinical data back and provide modifiable dashboards of PRO data, displaying average scores at each timepoint for the patients at their center (see Figure 1).

#### Conclusion

Overall, enrollment rates for CIBMTR PRO Protocol are comparable to other cancer registries. Collecting PRO data in a registry can have direct clinical implications by sharing PRO data back to clinicians.

**Fig. 1:**
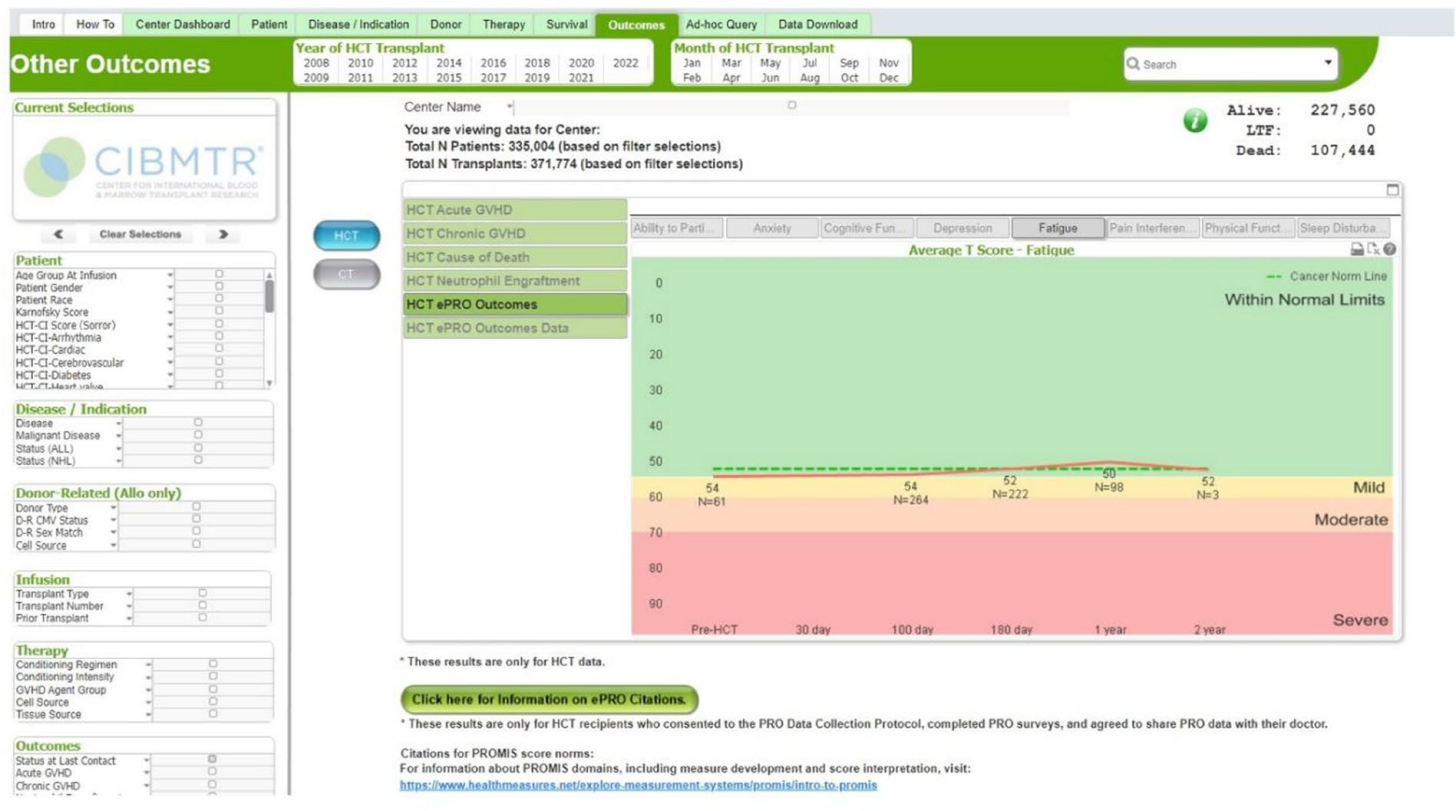
Example of Data Back to Centers Dashboard Displaying Average Fatigue Scores

### Scaling the collection of socioeconomic and quality of life data among haematopoietic cell transplant patients in the United Kingdom

Gemma Pugh^1^, Dawn Hart^1^, Christina Yiallouridou^1^, Karen Dean^1^, Lauren Young^1^, Tomos Llyod-Roberts^1^, Rachel Miller^1^, Robert Danby^1^

^1^Anthony Nolan, London, UK

*Journal of Patient-Reported Outcomes 2026*, **10(Suppl 1)**:Symposium 10

#### Aims

Social and demographic factors can influence patient outcomes following haematopoietic cell transplant (HCT). However, within the United Kingdom (UK) there is no systematic collection of socioeconomic or patient reported outcome data among HCT recipients. The SEQoL study has been designed to scale the collection of socioeconomic and quality of life data among HCT recipients to measure and understand inequalities.

#### Methods

The SEQoL study was developed with input from patients and health professionals and was preceded by a successful feasibility pilot study. Within SEQoL, adult (>18 years) allogeneic HCT patients are invited to complete a brief questionnaire before conditioning therapy on: the day of transplant (Day 0); day 28; day 100; day 180; day 270; and day 365. Data on individual socioeconomic position including housing tenure, income, education and occupational status is collected using items adapted from the UK Office of National Statistics census. Subjective socioeconomic status data is collected using the MacArthur Ladder, a two-item questionnaire which asks participants to rank their standing within a) UK society and b) their own community. Patient-reported outcome measures include the Functional Assessment of Cancer Therapy – Bone Marrow Transplantation, and the Lee Symptom Scale. Transplant centres (TCs) are responsible for the recruitment and consent of participants, but outcome data collection and follow-up are managed by the research team at Anthony Nolan. Each TC has received a tablet computer to facilitate e-consent and electronic data collection at baseline. Patient consent to link patient-reported outcome data from the SEQoL study with clinical outcome data held by national registries is obtained at enrolment.

#### Results

The SEQoL study has received positive expressions of interest from 23 adult TCs in the UK. The study is currently open in 11 of these TCs, the remaining are planned to open in 2025. The primary outcomes of SEQoL are recruitment rate, participant retention and data completion at each data collection interval.

#### Conclusion

Once linked to clinical outcomes data, the SEQoL study will enable investigation into the temporal change in quality-of-life outcomes post-transplant and the effect of subjective and objective socioeconomic status on patient outcomes.

## 1001 Refining well-being measures for Sweden´s ageing population: Through a cognitive testing approach

Marie-Louise Möllerberg^1^, Marit Preuter^2^, Kristofer Årestedt^3^, Jeanette Melin^4^

^1^Malmö University, Malmö, Sweden, ^2^RISE, Research Institutes of Sweden, Division Built Environment, Department System Transition and Service Innovation, Local and Regional Transition, Gothenburg, Sweden, ^3^Linnaeus University, Department of Health and Caring Sciences, Kalmar, Sweden, ^4^Swedish Defence University, Department of Leadership, Demand and Control, Karlstad, Gothenburg, Sweden

*Journal of Patient-Reported Outcomes 2026*, **10(Suppl 1)**:1001

### Aims

As populations in Sweden and globally continue to age, the demand for efficient healthcare and social services is rising. Yet high-quality measures of well-being are lacking in ensuring tailored and effective interventions. This study focuses on cognitive testing of 21 well-being items to ensure their clarity, relevance, and feasibility for use in Sweden’s ageing population.

### Methods

From a comprehensive literature review of existing well-being scales, item contents were identified, and 21 questions were distilled and standardized in language and tone. Cognitive interviews were conducted with 26 older people living in Sweden, using the think-aloud method complemented by probes to get a deeper insight into how participants responded to the well-being items. The interviews were analyzed according to a theoretical framework to understand respondents’ cognitive processes: (1) comprehending the question, (2) retrieving relevant information, (3) judging the information, and (4) providing an answer.

### Results

Overall, participants had no problem comprehending the questions (1). Most participants retrieved information well (2) and easily made judgements for answers (3). Some participants found it challenging to decide between “often” and “always”, but found the response options appropriate (4). Almost all participants perceived none of the questions as sensitive or difficult to answer honestly. The instructions to answer based on the past two weeks were often either forgotten or not noticed by the participants. Furthermore, the order of the questions matters; “I’m satisfied with my life” required more cognitive effort when placed at the beginning of the questionnaire than at the end. Therefore, some items were reordered. One item was added to the initial 21, “I feel that others perceive me as significant,” as participants brought up this aspect when answering “I feel significant to others”.

### Conclusion

Most participants found the well-being questions easy to comprehend, retrieve relevant information, and judge when choosing between the response options, supporting clarity, relevance, and feasibility for use in Sweden’s ageing population. The next step will include psychometric testing to ensure high-quality measures of well-being in older people. However, through this study, the first crucial steps have been taken to enable better assessment and understanding of well-being in older adults.

## 1002 Patient-Reported Outcome (PRO) Consortium’s Chronic Heart Failure (CHF) Working Group: Collaboration on qualitative research to support qualification of a step count-based measure to evaluate physical activity in persons with CHF

Maria Mattera^1^, Milena Anatchkova^2^, Ana C. S. Liberato^3^, Sonya Eremenco^1^

^1^Critical Path Institute, Tucson, Arizona, USA, ^2^Evidera, Wilmington, North Carolina, USA, ^3^Evidera, London, UK

*Journal of Patient-Reported Outcomes 2026*, **10(Suppl 1)**:1002

### Aims

Based on emerging technologies enabling data collection via mobile sensor devices (e.g., activity monitors), there is increasing interest in leveraging them to collect clinical trial endpoint data in persons with chronic heart failure (CHF). The PRO Consortium’s CHF Working Group developed a measurement strategy to assess physical activity for adults with CHF through an activity monitor-based endpoint measure. A main challenge was determining what variable(s) from the activity monitor would be used to derive a meaningful endpoint, therefore, qualitative investigation was warranted.

### Methods

The digital health technology (DHT)-passive monitoring clinical outcome assessment (COA) intends to assess aspects of objectively measured physical activity that reflect a person with CHF’s ability to perform meaningful daily activities; it was accepted into FDA’s Clinical Outcome Assessment Program (DDT COA #000114). In response to FDA feedback, a concept elicitation study, supported by an FDA grant, was conducted to generate qualitative evidence regarding the day-to-day activities that are meaningful to persons with CHF and the dimensions for carrying out these activities.

### Results

Thirty-one interviews were conducted with CHF participants (mean age=65.7; 51.6% female) in 2020 in the United States. Activities frequently reported involved light to moderate physical activity (e.g., cleaning, cooking, doing laundry) and walking (e.g., shopping, going to appointments). Duration of continuous activity, including taking steps, was highlighted as an important dimension to assess. An advisory panel was convened and, subsequently, an informal FDA meeting was held, to discuss the proposed metrics and obtain feedback. The CHF Working Group decided to focus on step count metrics related to duration of continuous steps as the best reflection of the concept of interest based on the qualitative research and multi-stakeholder feedback.

### Conclusion

Step count was identified through qualitative research and confirmed by subject matter experts and regulators as a promising DHT-based feature for use in the CHF population. The CHF Working Group’s next step toward qualification is to conduct analytic and clinical validation of metrics related to duration of continuous steps, using an approach that combines COA-based and DHT-related methods to evaluate the step count algorithm, derive step count metrics, and evaluate psychometric properties of the metrics.

## 1003 Symptom Severity and Quality of Life Among Long Term Metastatic Breast Cancer Survivors

Chloe Hery^1^, Kayla Williams^1^, Juan Peng^2^, Ashley Davenport^1^, Michelle Naughton^1^

^1^The Ohio State University College of Medicine, Columbus, Ohio, USA, ^2^The Ohio State University, Columbus, Ohio, USA

*Journal of Patient-Reported Outcomes 2026*, **10(Suppl 1)**:1003

### Aims

Metastatic breast cancer (MBC) survivors have unique psychosocial and medical needs that may go unrecognized. We examined the demographic and clinical characteristics of a cohort of MBC survivors, and the occurrence and severity of their physical and psychological symptoms.

### Methods

239 patients from the Ohio State University Stefanie Spielman Comprehensive Breast Center, who had been diagnosed with MBC for ≥ 1 year, completed a REDCap survey about their quality of life, self-rated health, symptoms, and supportive care needs. Wilcoxon rank and chi-square tests examined the severity of symptoms reported on the Patient-Reported Outcomes version of the Common Terminology Criteria for Adverse Events (PRO-CTCAE).

### Results

Patients were 59.4 years old (±12.9 years) and 5.7 years (±4.6 years) past their metastatic diagnosis of either HER2+ (n=60), HR+/HER2- (n=155) or triple negative (n=24) breast cancer. The majority were female (n=236), white (92%), and 51% had a bachelor’s degree or higher. Overall, the most bothersome or severe symptoms reported by the patients (Figure 1) were decreased sexual interest (24%), fatigue (14%), insomnia (11%), pain during vaginal sex (10%), and general pain (10%). Symptoms least commonly reported as severe or very severe were breast tenderness, problems with memory, and vomiting (all <1%). Quality of life was rated as excellent or very good by 53% of the patients, and fair or poor by 6.7%, with no significant differences by hormone receptor type (p=0.29). However, symptoms varied by receptor type. Abdominal pain (p=0.04), constipation severity (p=0.028), and hot flash frequency (p=0.031) were more common among HR+/HER2- metastatic patients. Muscle ache severity (p=0.02), and numbness severity (p=0.0001) were most common among HER2+ patients.

### Conclusion

MBC survivors in this study reported bothersome symptoms related to their treatment regimens, including sexual function, pain, fatigue, sleep, constipation and hot flashes. Reports of gastrointestinal discomfort and mental health concerns were less common. Detailed assessments of ongoing physical and psychological symptoms will provide information about the care needs of this understudied, but growing patient population. These assessments have the potential to improve supportive care treatments and offerings for these patients in the future.

**Fig. 1 (abstract 1003):**
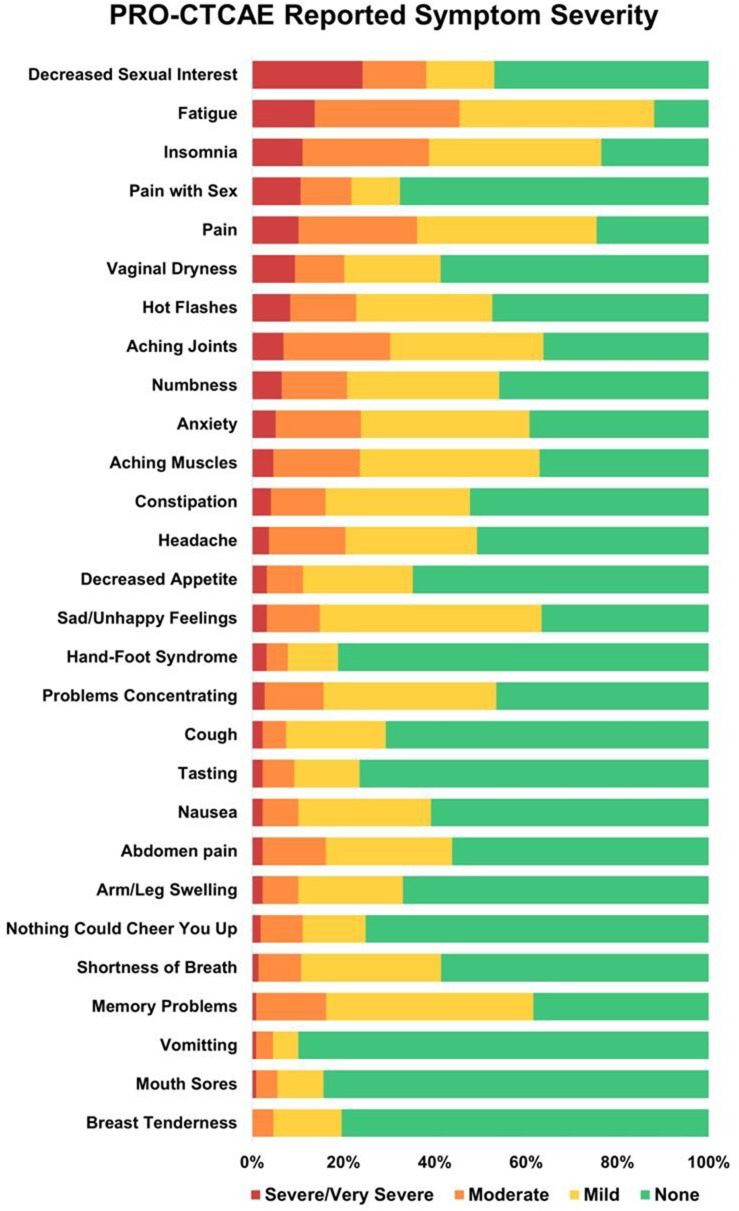
Symptom Severity and Quality of Life Among Long Term Metastatic Breast Cancer Survivors

## 1004 Using self-reported measures to assess financial toxicity in cellular therapy patients with hematologic diseases in a US setting: A scoping review

Sarah Reed-Thryselius^1^, Miranda Kapfhammer^1^, Lexi Finck^1^, Rachel Cusatis^1^

^1^Medical College of Wisconsin, Milwaukee, Wisconsin, USA

*Journal of Patient-Reported Outcomes 2026*, **10(Suppl 1)**:1004

### Aims

Hematopoietic cell transplantation (HCT) and chimeric antigen receptor t-cell therapy (CAR-T) are commonly employed treatments for patients with hematologic diseases. However, these treatments are associated with significant costs or other financial burdens, coined as financial toxicity (FT). Currently, there is no standard recommendation on how to assess FT experiences among these patient populations. Given the paucity of these findings, this study aimed to describe the self-reported FT measures used in youth and adult HCT/CAR-T populations in observational and trial research in the US by conducting a scoping review.

### Methods

Our search terms related to FT, HCT, and CAR-T were employed in Medline, PsycINFO, Scopus, and Web of Science databases. Articles were screened for eligibility by two independent researchers who conducted three rounds of reviews: article/title, methods, full-text review. Article conflicts were resolved through discussion and reaching consensus. Articles that were not original, peer-reviewed research, conducted in an international setting/foreign language, contained an inappropriate population or outcome, or did not use a patient-reported outcome that could produce a score were excluded.

### Results

Of the 2,949 articles eligible for title/abstract review, 56 were eligible for methods review, and 37 were eligible for full-text review. Of these, 19 were included in the final review. Among the included studies, 18 focused on HCT populations (three pediatric, 15 adult) and 1 focused on CAR-T populations (adult). A total of 11 different patient-reported outcome measures were utilized in these studies, with the COST questionnaire used the most frequently (n= 7, 37%) followed by the CTXD (n= 3, 18%). Other FT measures included the American Changing Lives Survey, Cancer Problem In-Living Scale, CPASS-7, IFDFW, SEWBS, and SUNS. Self-designed measures or unspecified questions were found in three studies (16%). About half of the studies assessed larger experiences like cancer distress, stress(ors), or burden but included specific FT subscales/domains (n= 8, 42%).

### Conclusion

A variety of self-reported measures were used to assess FT experiences in cellular therapy patients in a US setting. While some FT literature exists for HCT patients, less research has focused on CAR-T patients and future work should explore these experiences for this patient population.

**Fig. 1 (abstract 1004):**
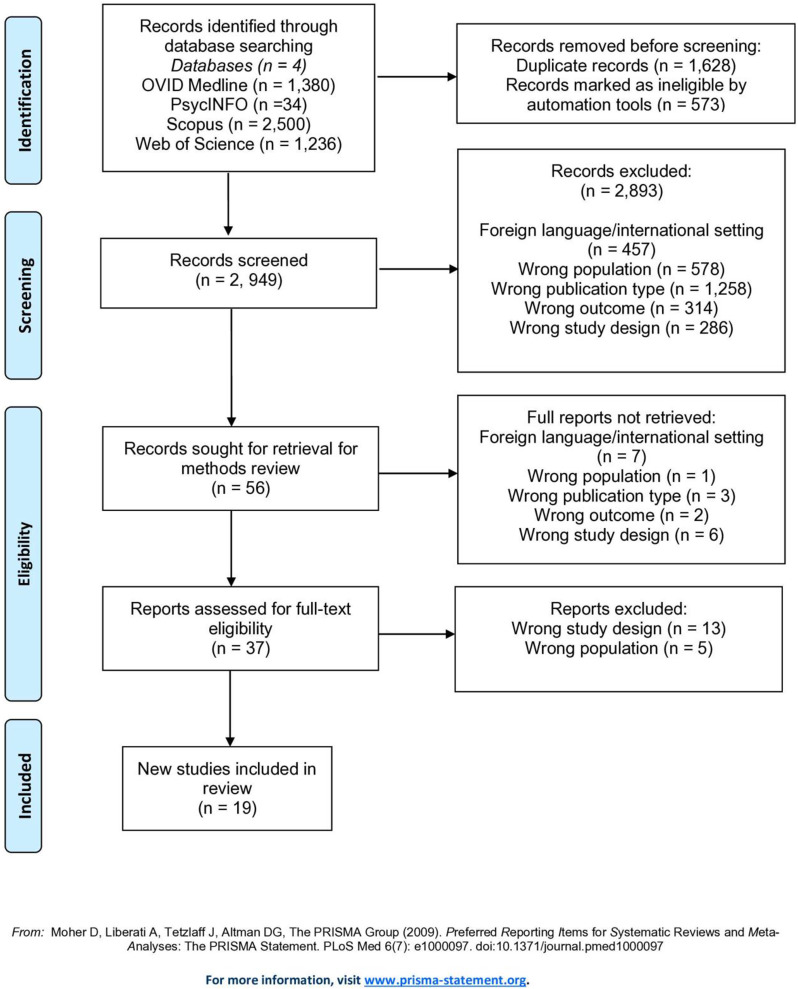
PRISMA Flow Diagram

**Table 1 (abstract 1004):**
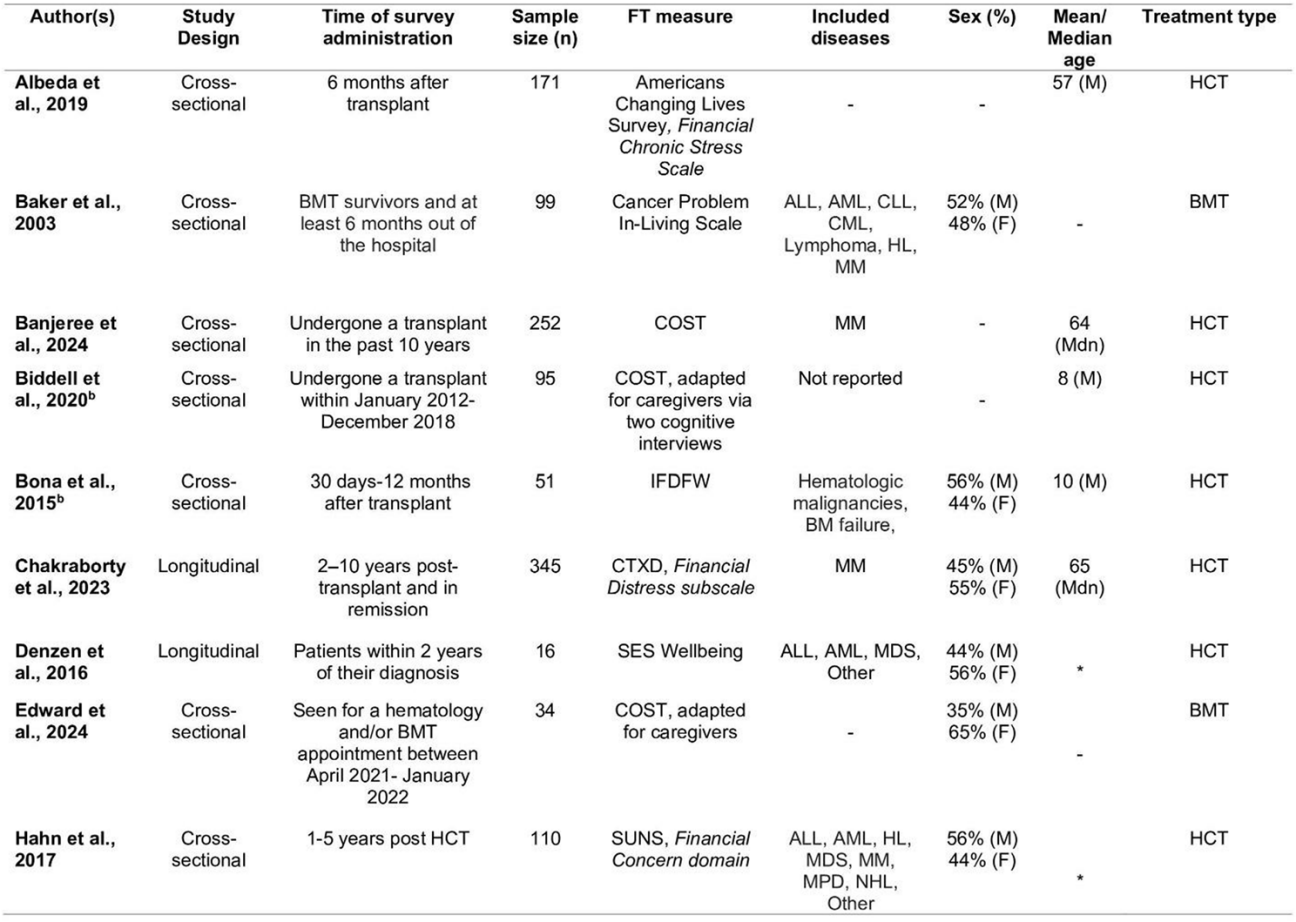
Study characteristics of included articles (n = 19)

**Table 2 (abstract 1004):**
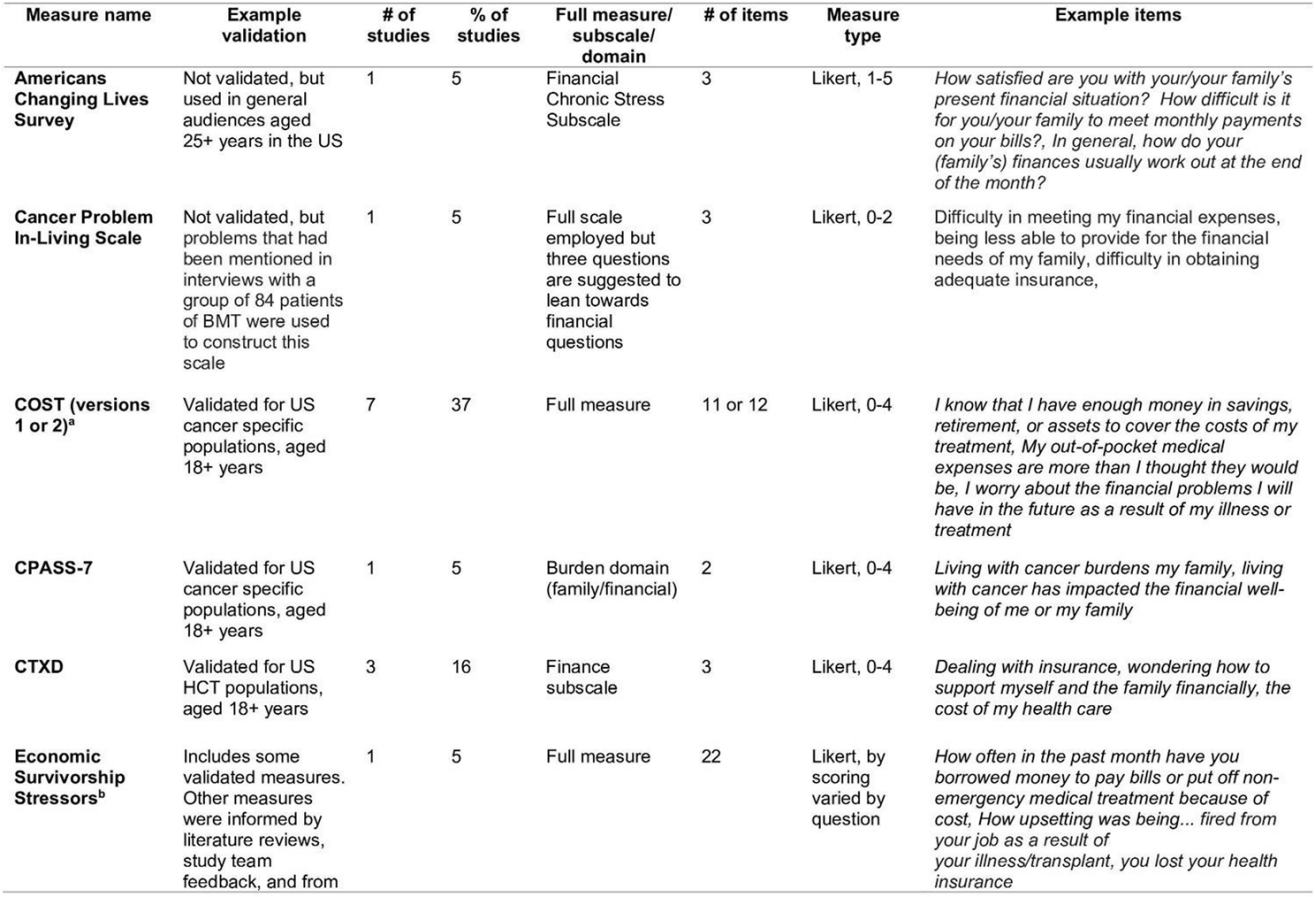
Self-reported FT measures used in included articles (n = 11)

## 1005 Expansion of the Rare Disease Clinical Outcome Assessment (COA) Resource, a Tool to Aid in COA-derived Endpoint Selection

Lindsey Murray^1^, Naomi Knoble^2^, Michelle Campbell^2^, Natalie Engmann^3^

^1^Critical Path Institute, Tucson, Arizona, USA, ^2^US FDA, Silver Spring, Maryland, USA, ^3^Denali Therapeutics, San Francisco, California, USA

*Journal of Patient-Reported Outcomes 2026*, **10(Suppl 1)**:1005

### Aims

In 2023, Critical Path Institute’s Rare Disease Clinical Outcome Assessment Consortium (RD-COAC) launched the Rare Disease COA Resource (Resource) for pediatric, non-oncologic rare disease populations. The 1st iteration of the Resource included COAs to measure domains of gross motor function, fine motor function, self-care, and communication/language. The Resource has recently been expanded.

### Methods

Representatives from biopharmaceutical companies, regulators, clinical researchers, COA experts, and patient advocates from the RD-COAC prioritized pain behavior, pain impact, sleep disturbance, and sleep impact for the 2nd iteration of the Resource. Aligned with the development process for the 1st iteration of the Resource, landscape analyses were conducted to identify COAs for selected domains. A subcommittee of experts from within the RD-COAC reviewed the COAs identified and moved a subset of these COAs forward for a detailed gap analysis that evaluated each COA against evidentiary expectations published by FDA, EMA, and PMDA. External advisory panels were convened and a consensus process between external advisory panels and subcommittee members determined final COAs for inclusion in the Resource.

### Results

Activities expanding the Resource were conducted throughout 2024, with an anticipated launch by Q3 2025. COAs included in the update represent tools most commonly used in rare disease research, published in the literature, available to examine against evidentiary criteria, and deemed most likely to support efficacy endpoints in rare disease clinical trials. Information from the gap analysis is presented on a publicly available website and the populated fields can be downloaded to support COA dossiers for specific medical product applications. The expanded Resource can be viewed at https://rdcoas.c-path.org.

### Conclusion

COA-based endpoints are critical to support efficacy analyses in rare disease clinical trials. The Resource accelerates treatments for rare disease patients by significantly reducing the time and cost of identifying COAs for a specific concept of interest by pre-specifying COAs with the highest potential to support efficacy analyses in rare disease clinical trial programs. The Resource is unique by making this information freely available and downloadable. COAs identified in the Resource may be considered (modified or as-is) for use as COA-based endpoints for rare disease treatment trials.

## 1006 Building better patient recruitment strategies: What the FDA recommends and how researchers are listening

Amanda Roussel^1^, Madison C. Bernstein^2^, Monica Brova^2^, Leighann Litcher-Kelly^2^, Tina Nguyen^2^, Daniella Olonilua^2^, Samantha L. Power^2^, Liam Quidore^2^, Kaelyn Rupinski^2^

^1^Adelphi Values Patient-Centered Outcomes, Boston, Massachusetts, USA, ^2^Adelphi Values, Boston, Massachusetts, USA

*Journal of Patient-Reported Outcomes 2026*, **10(Suppl 1)**:1006

### Aims

Recruiting clinical trial patients representing the wider patient population is vital to the integrity of results. Patient-centered clinical research can ensure generalizability of results by enrolling diverse samples reflecting the patient population, as advised by the United States Food and Drug Administration (FDA) Diversity Action Plan (DAP) guidance. Although DAPs have been voluntarily implemented, recruitment methods vary. This abstract summarizes the barriers and mitigators for achieving diversity in clinical research, as discussed in published literature, and demonstrates how patient-centered qualitative research can translate FDA guidance into action to support clinical outcome assessment (COA) strategy development.

### Methods

A PubMed search identified publications from the past 10 years on recruiting diverse patient populations in clinical research. Review of relevant articles identified barriers and mitigators, which were categorized into thematic domains. FDA guidance documents were reviewed to consolidate recommendations on enhancing diversity in clinical trials. The authors aggregated practices and procedures that support recruitment of diverse patient populations from their work in patient-centered qualitative COA research.

### Results

Across 21 articles, six distinct barriers to recruiting a diverse and representative patient sample were identified: fear and mistrust, lack of education or clear/accessible information, personal beliefs, study design, logistical barriers, and implicit bias of study staff. The FDA guidance documents articulate five relevant recommendations for mitigation strategies, which align closely with the reviewed literature. The authors’ experience in patient-centered qualitative COA research further mirrors these approaches. Together, these insights illustrate how FDA-aligned strategies can be operationalized to support diverse recruitment and inclusive COA strategy development (Figure 1).

### Conclusion

Understanding barriers and mitigators to diverse recruitment is essential to implementing FDA DAP recommendations. Effective recruitment strategies commonly identified in the literature align with FDA DAP recommendations and include trust-building, communication, and accessibility, but implementing these recommendations in qualitative research studies remains challenging. The authors’ recommendations for patient-centered qualitative COA research include setting diverse recruitment targets and proactively leveraging mitigators, such as inclusive study document design and community partnership, to support equitable participant representation. Continued application and documentation of these approaches in qualitative research will be key in strengthening development of COA strategies that reflect the experiences of all patients.

**Fig. 1 (abstract 1006):**
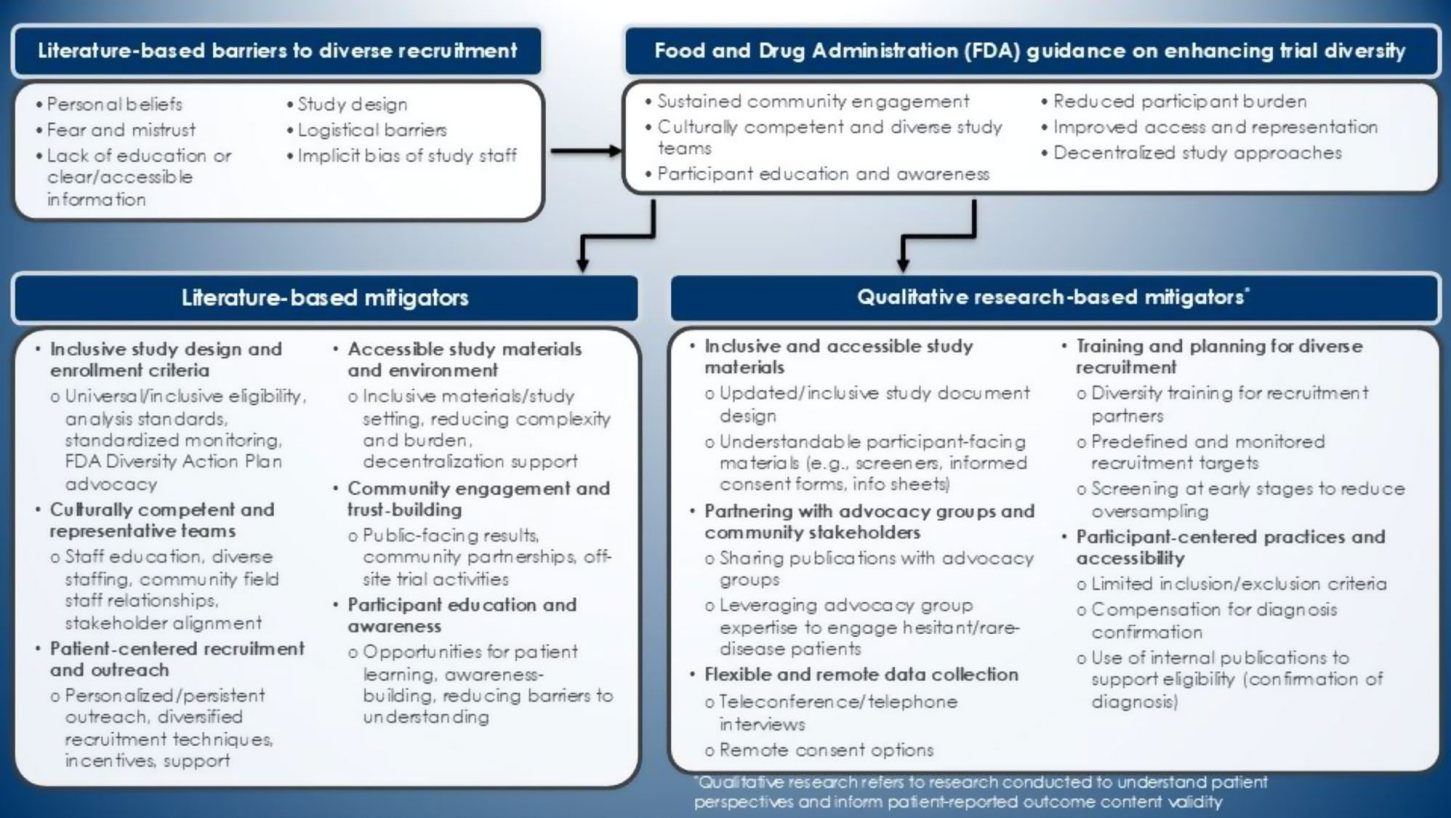
Building better patient recruitment strategies: What the FDA recommends and how researchers are listening

## 1007 Comparing Linking Approaches for Patient Reported Outcomes under Non-Normal Distribution: A Monte Carlo Simulation Study

Jiwon Kim^1^, Benjamin Schalet^2^

^1^Northwestern University, Chicago, Illinois, USA, ^2^Amsterdam University Medical Center, Amsterdam, Netherlands

*Journal of Patient-Reported Outcomes 2026*, **10(Suppl 1)**:1007

### Aims

In the field of patient reported outcomes, linking refers to the psychometric process used to establish a relationship between scores from different instruments. Empirical data in this field often exhibit zero-inflated distributions, with a large proportion of individuals clustered at the lower end of the distribution, reporting little to no symptoms. Despite this common pattern, there is limited research on how different linking methods perform under such data. This study aims to compare the performance of three linking approaches (unidimensional IRT, equipercentile, calibrated projection linking) when the underlying theta distribution deviates from normality, using item parameters from Patient Reported Outcomes Measurement Information System (PROMIS) Anxiety bank and Generalized Anxiety Disorder 7-item scale.

### Methods

Monte Carlo simulation study was conducted to evaluate the performance of the three linking methods under varying degrees of non-normality and latent correlations. Theta distributions for both instruments were generated using a mixture of two normal distributions: one centered at 0, and another centered at -1.5, to simulate the zero-inflated peak at the lower end. Mixing proportions ranged from 0% (normal) to 40% in 10% increments, and latent correlations from 0.6 to 1 in 0.05 increments. For each of the resulting 45 conditions (5 mixing proportions x 9 latent correlations), 20 datasets (N = 1,000) were simulated and linked using the three methods. Performance was evaluated using the Root Mean Square Error (RMSE) values of the difference between the linked and the true PROMIS theta.

### Results

Across all mixing proportions, RMSE increased as latent correlation decreased, reflecting greater linking error. The calibrated projection method consistently demonstrated the lowest RMSE, indicating strong robustness under non-normal conditions. The equipercentile and unidimensional IRT methods performed similarly across most conditions, with equipercentile method slightly outperforming the unidimensional IRT method at correlations above 0.9 with higher zero inflation (30-40%).

### Conclusion

Calibrated projection emerged as the most reliable linking method for patient reported outcomes with non-normal, zero-inflated distribution. While equipercentile method provides a viable alternative for high correlation scenarios, researchers should exercise caution under severely non-normal conditions. These findings provide practical guidelines for selecting linking approaches for patient-reported outcome measures under a more realistic distributional assumption.

## 1008 Characterizing disease-related stigma and treatment-related stigma among people with HIV: insights from BEYOND

Irina Kolobova^1^, Tamra Keeney^2^, Christina Donatti^3^, Amara Orji^2^, Lauren Henning^2^, Cindy Garris^1^, Dominy Browning^3^, Mark Kosinski^2^

^1^ViiV Healthcare, Durham, North Carolina, USA, ^2^IQVIA Quality Metric, Inc., Providence, Rhode Island, USA, ^3^ViiV Healthcare, London, UK

*Journal of Patient-Reported Outcomes 2026*, **10(Suppl 1)**:1008

### Aims

Disease-related stigma is commonly experienced by people with HIV (PWH) and can be measured using the 6-item Internalized AIDS-Related Stigma Scale (IA-RSS). While disease-related stigma represents an important measurement construct for this population, IA-RSS items reflect societal influences that are difficult to ameliorate with treatment interventions alone. Qualitative research indicates that PWH may experience multiple treatment related challenges, including fear of disclosure, anxiety around treatment adherence, daily reminders of HIV, and feeling stigmatized by HIV medication. To explore potential differences between disease- and treatment-related stigma, this study used data from BEYOND, a prospective observational study of PWH who switched from daily oral to long-acting injectable medication, to characterize and compare the IA-RSS and Treatment Related Worries & Stigma Scale (TRWS).

### Methods

Secondary data analyses of BEYOND survey data, collected at baseline, month 6, and month 12. Standardized z-scores were calculated at the participant level for the IA-RSS and TRWS to enable comparison. Distribution-based score tertiles were used to categorize participants’ baseline TRWS levels into low (n=86), moderate (n=100), and high (n=122). Generalized mixed-effects linear regression models with a random subject-level intercept were used to estimate mean population-level scores at each study time point, stratified by TRWS tertiles. Mean scores for each measure were then plotted to construct unadjusted score trajectories.

### Results

Among participants with low TRWS at baseline, scores on both measures started low and remained so over the study period (Figure 1). For participants with moderate TRWS at baseline, the IA-RSS score remained stable, while the TRWS score declined by approximately 1 SD from baseline to month 6 and then remained stable through month 12. Participants with high TRWS at baseline also had relatively stable IA-RSS scores, but a larger decline in psychological challenges from baseline to month 6 (~1.5 SD), with further decline noted through month 12.

### Conclusion

These descriptive trends highlight potential differences between disease- and treatment-related stigma measured by each instrument. Differences in the construct of stigma, as experienced by PWH, have important implications for instrument selection and assessment of treatment interventions on stigma in HIV moving forward.

**Fig. 1 (abstract 1008):**
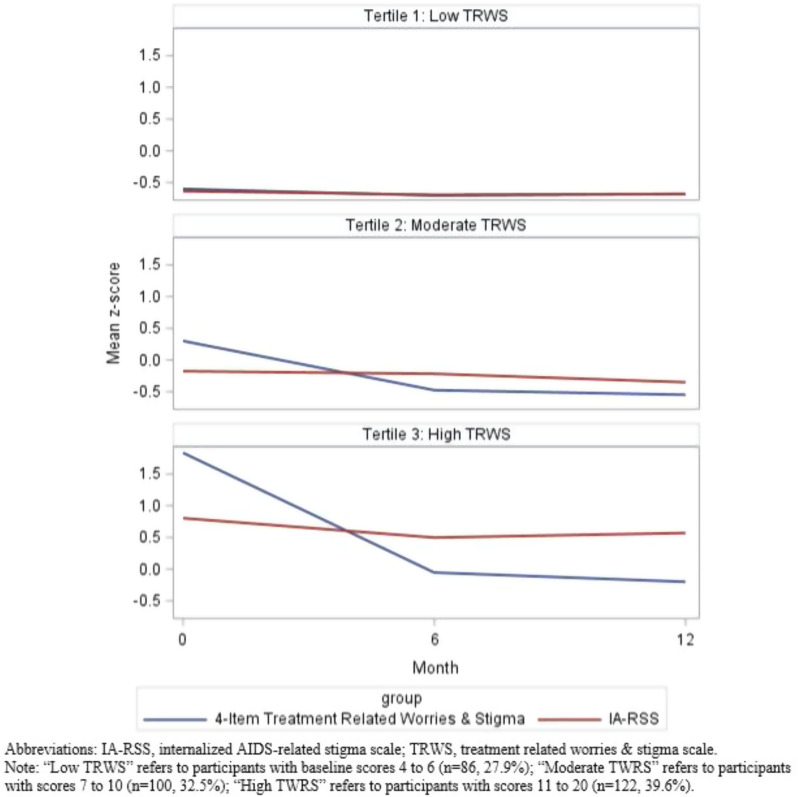
Mean Standardized Scores on the Treatment Related Worries & Stigma Scale and IA-RSS by Baseline Treatment Related Worries & Stigma Tertiles in BEYOND: Baseline to Month 12

## 1009 Can AI contribute to the development of a conceptual model of the patient experience? A feasibility assessment

Danielle Burns^1^, Elizabeth Collins^1^, Payton Ramsey^1^, Jake Macey^1^

^1^Clarivate, London, UK

*Journal of Patient-Reported Outcomes 2026*, **10(Suppl 1)**:1009

### Aims

The Food and Drug Administration (FDA) advises identifying and incorporating what is meaningful and important to patients during medical product development, including when selecting, modifying or developing clinical outcome assessments (COAs). Conceptual models, which visually depict the patient experience of any given health condition, are frequently used to guide COA selection. Conceptual models represent different aspects of the patients’ health experiences resulting from a disease/condition, through organized lists of concepts connected with lines to illustrate relationships between them. Primary and secondary research is typically synthesized, organized, and presented as a conceptual model, often produced manually using software such as PowerPoint. However, this consumes valuable human hours. Given that artificial intelligence (AI) is increasingly competent at generating and editing images, AI may be efficient for producing conceptual models. This study examined the feasibility of AI in the development and design of conceptual models, to understand whether AI would be a time-saving tool for model development.

### Methods

An online search was conducted to identify symptoms and impacts of depression, to produce a novel data source for this study. A list of unorganized concepts spanning multiple domains (the ‘data source’) was compiled by the study team. Microsoft CoPilot was given the data source and simple prompts (instructions) to first organize concepts into appropriate domains, and to then produce a conceptual model aligned with FDA guidance. Prompts were iteratively amended based on outputs, and Step 2 was repeated.

### Results

CoPilot could not initially produce a well-organized conceptual model of depression. Models contained spelling errors, fabricated words, and unrelated novel/nonsensical concepts not included in the data source. More detailed prompts did not eliminate these issues. An alternative request for CoPilot to produce code that could design a conceptual model in free third-party flowchart/diagram software (Mermaid Live Editor) was more successful and produced an organized conceptual model that was accurate to the data source.

### Conclusion

Whilst AI can generate a conceptual model when given a data source, extensive human prompting is still required to ensure its accuracy. However, AI may save time when generating third-party code to design a conceptual model.

## 1010 Meaningful change in adult attention-deficit/hyperactivity disorder (ADHD): Using vignettes to estimate thresholds for meaningful improvement and deterioration in scores on the Adult ADHD Investigator Symptom Rating Scale (AISRS)

Miranda Lauher-Charest^1^, Dorothee Oberdhan^2^, Rakhee Ghelani^1^, Clarissa Simas^1^, Kristi Jackson^1^, Caroline Ward^2^, Jakob Bjorner^1^

^1^IQVIA Quality Metric, INC., Providence, Rhode Island, USA, ^2^Otsuka Pharmaceutical Development & Commercialization, Inc., Rockville, Maryland, USA

*Journal of Patient-Reported Outcomes 2026*, **10(Suppl 1)**:1010

### Aims

Attention-deficit/hyperactivity disorder (ADHD) is a neurodevelopmental disorder characterized by difficulty focusing, inattentiveness, and impulsive behavior. The Adult ADHD Investigator Symptom Rating Scale (AISRS) is an investigator-administered measure with 18 items across 2 domains: inattentive (IA) and hyperactive-impulsive (HI). This research aimed to estimate thresholds for severity and meaningful change on the AISRS.

### Methods

This mixed-methods study consisted of one-on-one, 60-minute bookmarking interviews in which participants were prompted to assess vignettes (descriptions of hypothetical persons representing certain characteristics). Vignette development used non-parametric Item Response Theory models and data from Otsuka’s clinical trials in adults with ADHD. Three sets of 10 vignettes were developed: each reflecting different AISRS scores (total, IA, and HI). During interviews, participants read 1 set of vignettes and assigned each a severity rating. Participants then completed meaningful improvement and deterioration bookmarking activities. Interviews were audio-recorded, and transcripts were coded and analyzed using inductive and deductive approaches. Thresholds for meaningful severity scores, improvement, and deterioration were derived using ordinal and binary logistic regression mixed models (LRMMs) and simple linear interpolation (SLI); 95% confidence intervals were estimated by person-level empirical bootstrap.

### Results

Seventy-two adults with ADHD participated (72% female). Severity threshold estimates were similar (total and HI scores) or identical (IA scores) between LRMM and SLI (Table 1). For meaningful improvement, total score changes of -7 and IA/HI score changes of -3 or better had a >50% chance of being rated meaningful. For meaningful deterioration, total score changes of +6, HI score changes of +2, and IA score changes of +1 or worse had a >50% chance of being rated meaningful (Table 2). Anxiety, inattentiveness, fidgeting, task avoidance, impacts on work/finances, and quality of life were noted as concepts most important to decision-making.

### Conclusion

Severity ratings consistently increased/decreased with vignette scores. Thresholds for meaningful deterioration were smaller than those for improvement, suggesting less change is needed to identify meaningful deterioration than improvement in ADHD. Total score threshold values provide the most confidence in meaningful change. These AISRS thresholds offer an alternative to the clinical global impression (CGI)–based threshold of –18, which is not aligned with ADHD clinical practice.

**Table 1 (abstract 1010):**
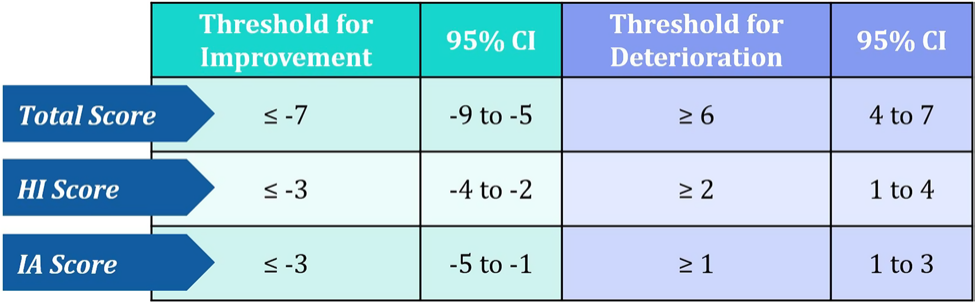
Thresholds for Meaningful Score Regions

**Table 2 (abstract 1010):**
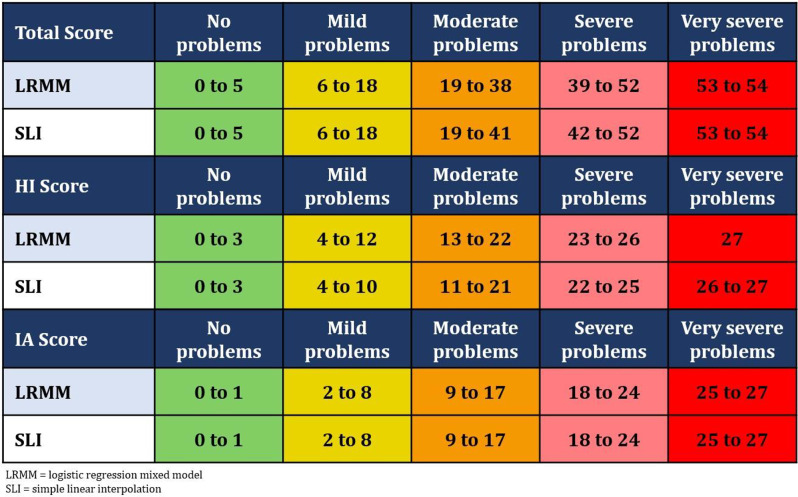
Meaningful Change Thresholds

## 1011 Differential item functioning of the PROMIS sleep disturbance – short form 8a across levels of health literacy in a community sample of adults

Rowida Mohamed^1^, Michael K. Paasche-Orlow^2^, Eloisa Serrano^3^, Melissa Marquez^1^, Lori Henault^2^, Claire Weaver^3^, Noël C. Slesinger^4^, Francesca R. Farina^1^, Emily Hurstak^5^, Michelle Taddeo^3^, Katherina Hauner^3^, James W. Griffith^1^

^1^Pritzker School of Medicine, The University of Chicago, Chicago, Illinois, USA, ^2^Tufts University School of Medicine, Boston, Massachusetts, USA, ^3^Feinberg School of Medicine, Northwestern University, Chicago, Illinois, USA, ^4^Department of Pediatrics, University of Minnesota, Chicago, USA, ^5^Department of Adult Primary Care, Boston University Medical Center, Boston, USA

*Journal of Patient-Reported Outcomes 2026*, **10(Suppl 1)**:1011

### Aims

Health literacy may introduce bias in self-reported health assessments. This cross-sectional study examined the psychometric properties of the PROMIS Sleep Disturbance—Short Form 8a (SF 8a) across varying health literacy levels in a diverse community sample.

### Methods

Participants aged ≥ 18 years old were recruited from Boston and Chicago through community outreach and ResearchMatch. Health literacy was measured using the Health Literacy Assessment Using Talking Touchscreen Technology (Health LiTT), with a cutoff of 55 to categorize participants into low versus adequate health literacy groups. Confirmatory factor analysis and parallel analysis evaluated the scale dimensionality of the PROMIS Sleep Disturbance–SF 8a. Convergent and divergent validity were examined via Pearson correlations with other PROMIS-57 domains. To assess differential item functioning (DIF), we used the ‘lordif’ package in the R platform, which uses an ordinal logistic regression framework and graded response model for IRT trait estimation. Cutoff thresholds for identifying DIF were established through Monte Carlo simulations with 5,000 iterations and an alpha level of 0.01.

### Results

The sample (n=551) was predominantly female (59.7%) and Black (53.0%), with 53.7% demonstrating low health literacy. Participants with low health literacy reported higher scores on sleep disturbance (48.9 ± 15.9) than those with adequate health literacy (48.9 ± 15.9). The scale demonstrated adequate unidimensionality and expected validity patterns, including positive correlations with depression, anxiety, fatigue, and pain interference, and negative correlations with physical functioning and social participation. Three of eight items showed significant DIF by health literacy: Item 1 (“My sleep quality was …”), Item 3 (“I had a problem with my sleep …”), and Item 5 (“My sleep was restless …”). Although these items showed both uniform and non-uniform DIF, the overall impact on total scale scores was negligible (Figure 1).

### Conclusion

Despite minor DIF at the item level, the psychometric properties of the PROMIS Sleep Disturbance- Short Form 8a support its use in populations with varying levels of health literacy. These findings emphasize the importance of evaluating health literacy as a potential source of bias in patient-reported outcomes.

**Fig. 1 (abstract 1011):**
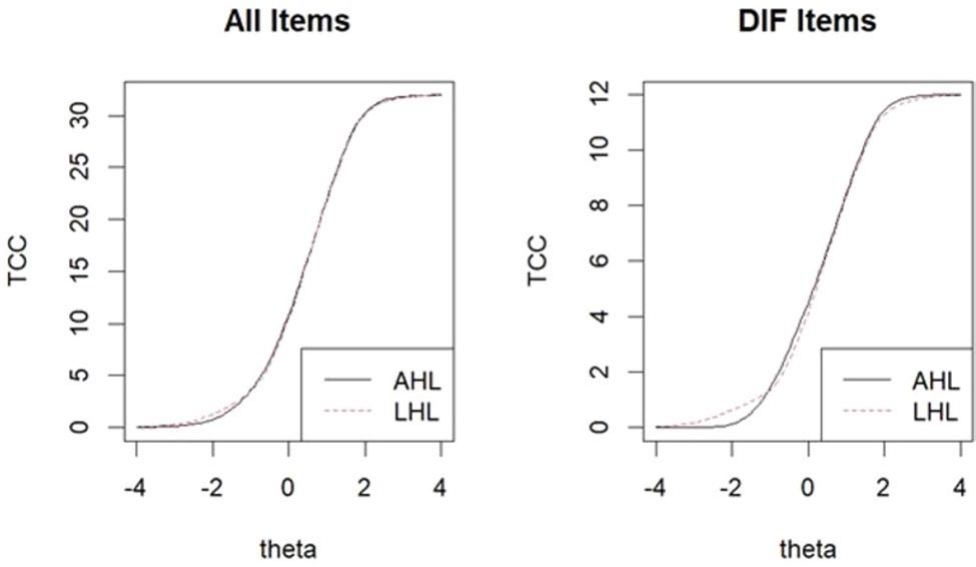
Total impact of differential item functioning (DIF) on test characteristics curves (TCCs) of the PROMIS Sleep Disturbance - Short Form 8 for all items (with or without DIF) (Left Panel) and only items with DIF (Right Panel). DIF: differential item functioning; TCCs: test characteristics curves; AHL: adequate health literacy group; LHL: low health literacy group

## 1012 Artificial intelligence meets clinical trial design: A test case in predicting patient achievement of minimal significant difference using conditional generative adversarial networks

Siddharth Kakked^1^

^1^OPEN Health, Falmouth, Maine, USA

*Journal of Patient-Reported Outcomes 2026*, **10(Suppl 1)**:1012

### Aims

This study investigates whether a conditional Generative Adversarial Network (cGAN) can reliably predict the probability of successfully achieving a clinical trial endpoint. The primary objective was to evaluate the model’s ability to produce accurate probability estimates using Minimal Significant Difference (MSD) as a test case by analyzing patient-specific variables—including demographics, baseline patient-reported outcome (PRO) scores, Patient Global Impression of Change (PGIC) as the anchor, clinical measurements, and treatment history. A secondary objective was to determine the extent to which these predictions could support the optimization of trial design parameters.

### Methods

Secondary patient records from previous clinical trials were preprocessed as conditioning inputs for the cGAN. The generator produced synthetic probability distributions of MSD achievement while the discriminator compared these against actual outcomes to enhance accuracy. A specialized loss function incorporating clinical benchmarks was implemented, with training conducted over 200 epochs. The MSD as a test case was computed in R using anchor-based methods, with PGIC responses utilized to define MSD achievement levels, as recommended by the FDA’s Patient-Focused Drug Development (PFDD) guidance and then compared with the cGAN’s outputs. Performance was assessed through discrimination metrics (AUC, precision, recall, F1 score, Matthews Correlation Coefficient) and calibration accuracy (Brier Score), with comparative analyses against logistic regression and random forest models.

### Results

The cGAN discriminator achieved a higher discriminatory performance (AUC = 0.87, 95% CI: 0.83–0.91; Figure 1) than logistic regression (AUC = 0.79, 95% CI: 0.74–0.84) and random forest (AUC = 0.81, 95% CI: 0.76–0.86). Additional metrics confirmed this advantage, showing higher precision (0.84), recall (0.82), F1 score (0.83), and an MCC of 0.72. The model demonstrated well-calibrated probability estimates (Brier Score = 0.12). Moreover, subgroup analyses yielded AUC values ranging from 0.82 to 0.89.

### Conclusion

This proof-of-concept study establishes that cGANs can effectively synthesize realistic patient outcome distributions and accurately predict MSD achievement probabilities for clinical trial endpoints. This approach potentially enables more efficient trial design through improved patient selection and outcome prediction, potentially reducing sample size requirements and trial duration. Future work will validate these findings in larger prospective datasets and integrate this methodology into clinical trial optimization systems.

**Fig. 1 (abstract 1012):**
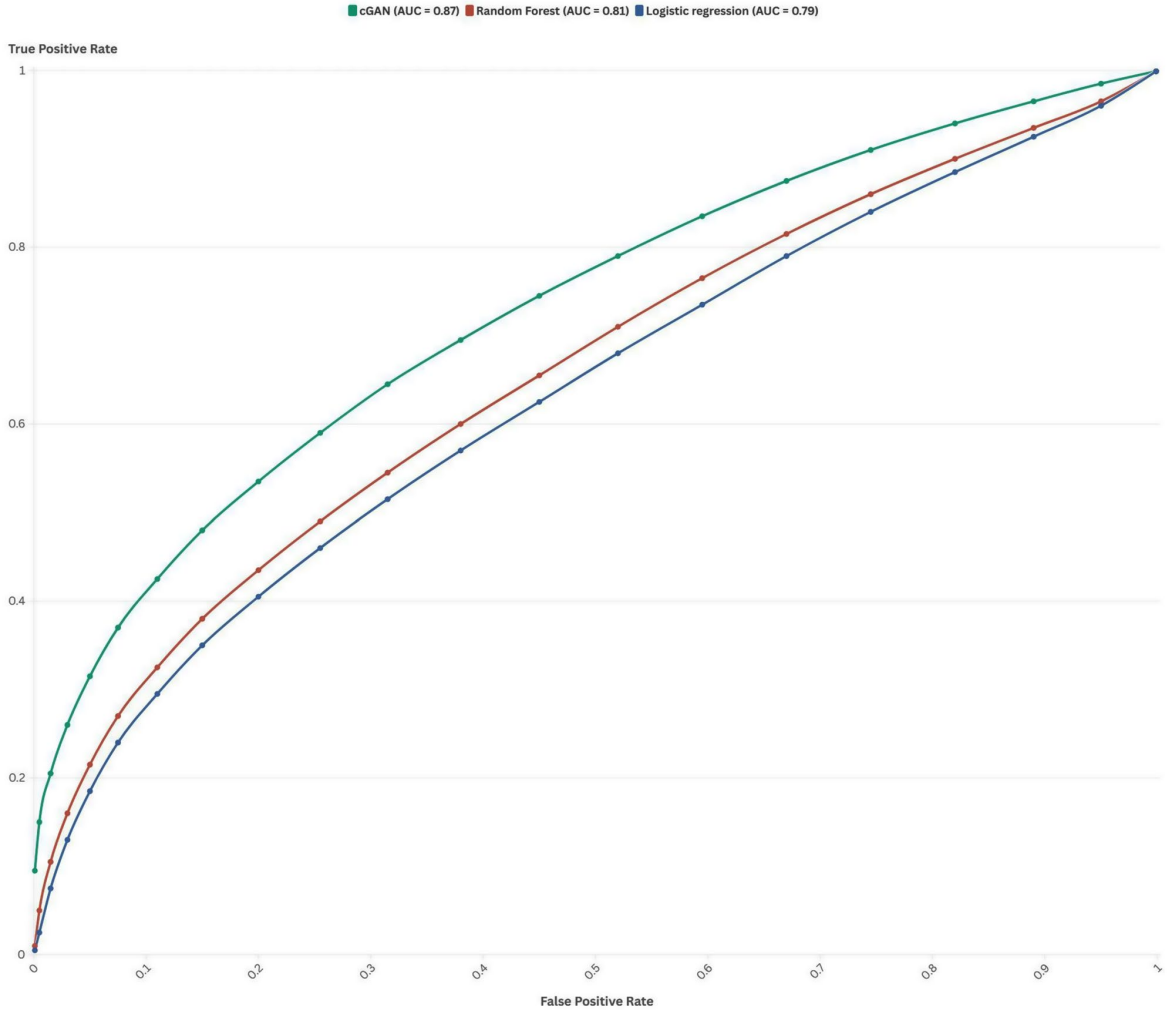
Comparison of ROC Curves: cGAN vs. Logistic Regression vs. Random Forest

## 1013 Assessing HIV treatment related worries and stigma: Psychometric properties of a novel, brief patient-reported outcome measure

Irina Kolobova^1^, Tamra Keeney^2^, Christina Donatti^3^, Amara Orji^2^, Lauren Henning^2^, Dominy Browning^3^, Mark Kosinski^2^

^1^ViiV Healthcare, Durham, North Carolina, USA, ^2^IQVIA Quality Metric, Inc., Providence, Rhode Island, USA, ^3^ViiV Healthcare, London, UK

*Journal of Patient-Reported Outcomes 2026*, **10(Suppl 1)**:1013

### Aims

Many people with HIV (PWH) experience heightened emotional burden and challenges related to daily oral medication regimens, which impact well-being and treatment adherence. A four-item patient-reported questionnaire, the Treatment Related Worries & Stigma (TRWS) Questionnaire, was developed to assess fear of disclosure, anxiety around adherence, unwelcome reminder of HIV, and how often taking HIV medication makes an individual feel stigmatized. Prior qualitative research confirmed relevance and interpretability of 3 of the 4 items. This study leveraged data from a prospective, observational study of PWH (BEYOND) to confirm the unidimensional scaling of the four items and evaluate internal consistency reliability and construct validity (convergent and known groups) of the scale score.

### Methods

Secondary data analysis of BEYOND survey data collected at baseline and month 12. Confirmatory factor analysis was conducted to confirm unidimensional scaling. Cronbach’s alpha was calculated to evaluate internal consistency. A priori hypotheses were constructed for testing construct validity. Correlation analyses were used to evaluate the convergent validity of the scale with 2 criterion measures: the HIV Treatment Satisfaction Questionnaire 12-item status version (HIVTSQ12s) and the Internalized AIDS-Related Stigma Scale (IA-RSS). Known-groups validity was evaluated by comparing mean scale scores across subgroups defined by distribution-based tertiles of the HIVTSQ12s and IA-RSS.

### Results

Confirmatory factor analysis confirmed the unidimensional scaling of the four-item scale. The four-item scale demonstrated adequate internal consistency reliability at baseline (α=0.79). As hypothesized, strong, positive correlations (r≥ 0.40) were noted between the four-item scale and the IA-RSS; small, negative correlations were noted with the HIVTSQs. Mean scores on the TRWS were higher (worse) among those with lower satisfaction on the HIVTSQs (low 11.2 ± 4.4; moderate 9.7 ± 4.2; high 8.4 ± 3.9) and higher stigma on the IA-RSS (high 13.3 ± 4.1; moderate 8.7 ± 3.1; low 6.4 ± 2.5).

### Conclusion

Findings support that the TRWS is strongly related to the concept of stigma but may represent a concept that uniquely captures worries and stigma associated with oral HIV treatment. Future studies are needed to understand how different treatment modes and regimens can improve the treatment-related worries and stigma that PWH experience.

## 1014 Ascertainment of Minimal Clinically Important Differences in the Chronic Prostatitis Symptom Index

Zong-Shi Qin^1^, Yongpei Yu^1^, Jiani Wu^2^, Yangfeng Wu^1^

^1^Peking University Clinical Research Institute, Beijing, China, ^2^Guang’anmen Hospital, China Academy of Chinese Medical Sciences, Beijing, China

*Journal of Patient-Reported Outcomes 2026*, **10(Suppl 1)**:1014

### Aims

To establish an anchor-based metric of minimal clinically important difference (MCID) regarding the National Institutes of Health Chronic Prostatitis Symptom Index (NIH-CPSI) and its 3 subscale scores (pain, urinary, and quality of life).

### Methods

This secondary analysis of a randomized clinical trial with 440 participants used baseline and postintervention data, which evaluated acupuncture versus sham acupuncture. Participants included adults with CP/CPPS who received acupuncture or sham acupuncture in 10 hospitals in China. Data collection was completed in November 2020, and data analysis was completed in June 2021. The main outcome was anchor-based MCID values for the NIH-CPSI total score and three subscales (pain, urinary, and quality of life), calculated using the standard error of measurement. Baseline to postintervention changes in NIH-CPSI and its 3 subscale sores were grouped into 3 categories: improved, no change and worsened. The receiver operating characteristic (ROC) curve and estimated the Area Under the Curve (AUC) was used for find the best compromise between sensitivity and specificity in two-measure dataset (baseline and end of treatment). For longitudinal measures approach (baseline, middle of treatment, end of treatment, and follow-up period), a mixed linear regression with a random effect on the individual to estimate the mean change score in each category of patients were used.

### Results

This secondary analysis using data from 440 participants in a randomized clinical trial comparing the chronic prostatitis/chronic pelvic pain syndrome (CP/CPPS) patients in acupuncture intervention (220 participants) with sham acupuncture (220 participants) found that the within-group MCID value for CPSI was 6-point, and MCIDs were 1.8-point for pain subscales, 1.6-point for urinary subscales, and 2.6 for QoL subscales, respectively. In addition, the between-group MCID value for CPSI was 3-point.

### Conclusion

Based on the data from multi-center, large sample size randomized clinical trial, MCID improvement of more than 6-point on the CPSI total score was quantitatively significant for within-group change. Regarding between-group differences, a 3-point difference between groups is deemed as clinically significant.

## 1015 Patient-reported and transfusion-related outcomes in alpha- and beta-transfusion-dependent thalassemia: A multi-region real-world survey

Janet Kwiatkowski^1^, Khaled Musallam^2^, Maria Domenica Cappellini^3^, Christina Chamberlain^4^, Amey Rane^4^, Keely Gilroy^4^, Susan Morris^4^, Emma Chatterton^5^, Katie Lewis^5^, Brianne Kerr^5^, Ali Taher^6^

^1^Children’s Hospital of Philadelphia, Philadelphia, Pennsylvania, USA, ^2^Burjeel Medical City, Abu Dhabi, United Arab Emirates, ^3^Fondazione IRCCS Ca’ Granda Ospedale Maggiore Policlinico, Milan, Italy, ^4^Agios Pharmaceuticals, Cambridge, Massachusetts, USA, ^5^Adelphi Real World, Bollington, UK, ^6^American University of Beirut Medical Centre, Beirut, Lebanon

*Journal of Patient-Reported Outcomes 2026*, **10(Suppl 1)**:1015

### Aims

The aim of this analysis was to characterize humanistic disease burden and transfusion-related outcomes among adults with transfusion-dependent thalassemia (TDT) globally.

### Methods

Data were collected from the Adelphi Real World Thalassemia Disease Specific Programme™, a cross-sectional survey of physicians and their TDT patients (≥18 years; α and β) in Asia, Middle East and North Africa (MENA), Europe (EU)/North America (NA), and South America from February-November 2024. Physicians reported patients’ minimum pre-transfusion hemoglobin (Hb) level, maximum pre-transfusion Hb level, and transfusion-related data. Patients completed a corresponding survey, which included validated patient-reported outcomes measures (PROMs): Functional Assessment of Chronic Illness Therapy (FACIT)-Fatigue, Patient-Reported Outcomes Measurement Information System (PROMIS) Physical Function (PF), and Work Productivity and Activity Impairment (WPAI)-Thalassemia. Descriptive analyses were conducted by region and min and max pre-transfusion Hb level thresholds (<9.5g/dL vs ≥9.5g/dL). Patients with gene therapy or hematopoietic stem cell transplantation were excluded.

### Results

Overall, 51 physicians reported data for 223 patients with TDT. Min and max pre-transfusion Hb levels over the past 12 months varied by region (Table 1). Issues with blood supply in the past 12 months varied across regions with 52.5% (=21/40) of patients affected in Asia, 17.0% (n=17/100) in EU/NA, 63.2% (n=43/68) in MENA and 100.0% (n=1/1) in South America. Over 90% of patients experienced acute or long-term complications, and over 75% experienced iron overload, due to their transfusions across regions (Table 1). US norm PROM scores were referenced to evaluate the humanistic burden of patients with TDT (Table 2). Although PROM scores varied across regions and by min and max pre-transfusion Hb levels, fatigue, physical function impairment, and overall work impairment were observed in all subgroups. Fatigue and impairments tended to be less pronounced in the EU/NA region and for patients with min pre-transfusion Hb ≥9.5g/dL.

### Conclusion

Across geographical regions and pre-transfusion Hb levels, adults with TDT generally experience worse fatigue and greater impairment in physical function and work productivity than the general population. Blood supply issues and transfusion-related complications highlight an unmet need for novel therapies to reduce disease burden in TDT.

**Table 1 (abstract 1015):**
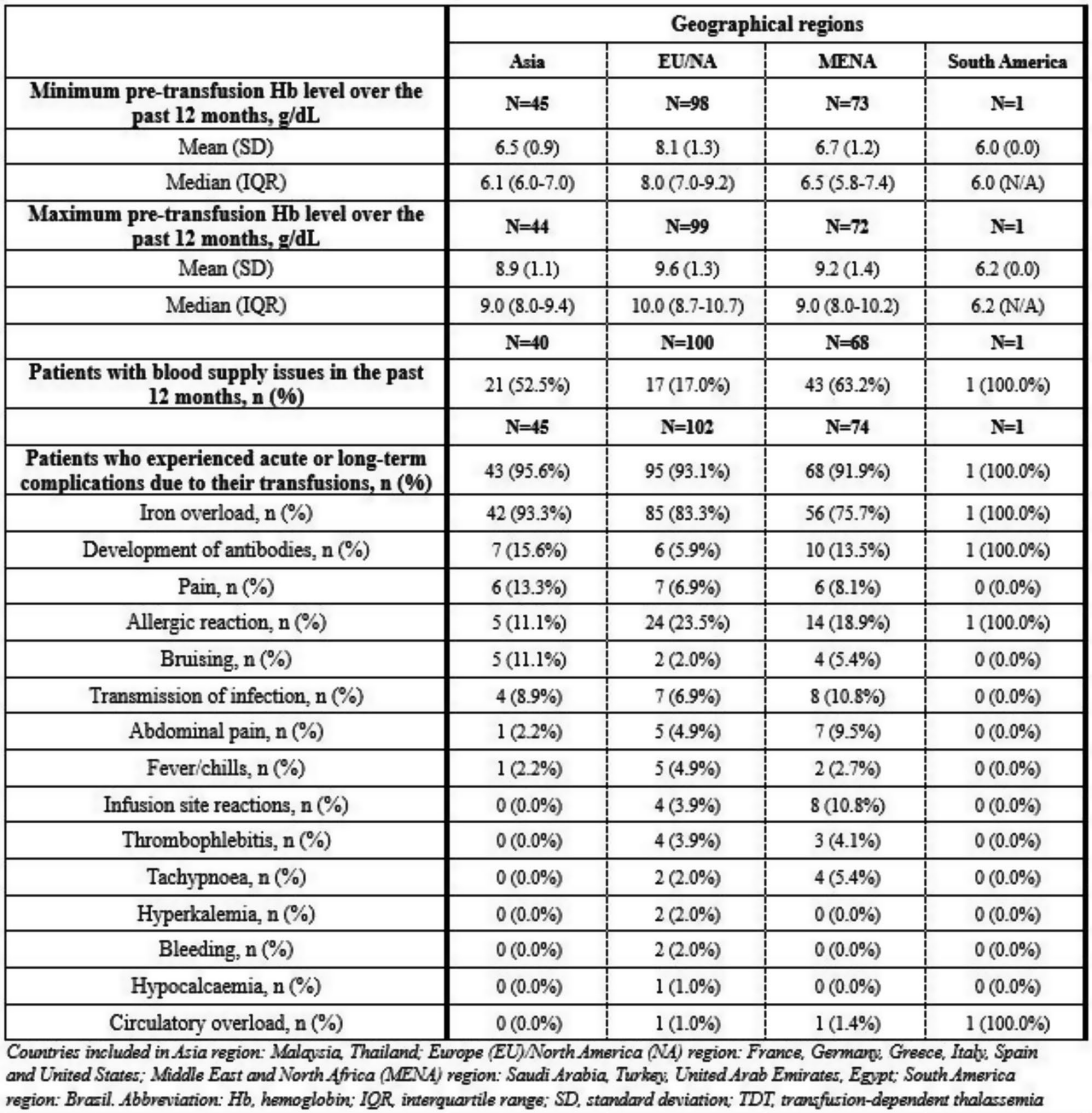
Physician-reported pre-transfusion Hb levels and transfusion-related outcomes in patients with TDT

**Table 2 (abstract 1015):**
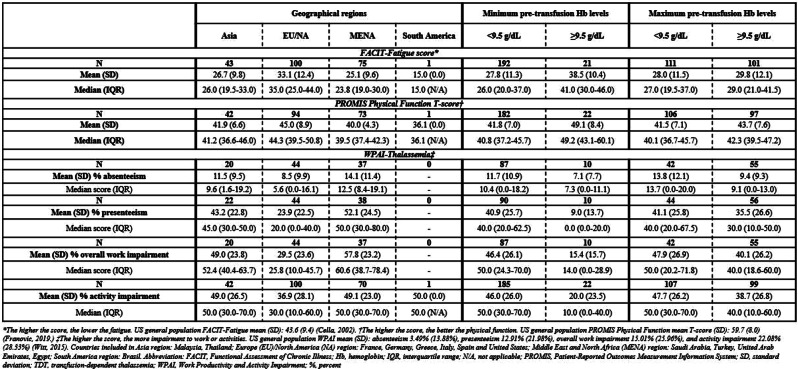
Patient-reported health-related quality of life and work productivity data for patients with TDT

## 1016 Enhancing health-related quality of life for care partners of individuals with traumatic brain injury: A randomized controlled trial of the CareQOL mobile health intervention

Noelle Carlozzi^1^, Madison Fansher^1^, Rongqi Bei^1^, Jennifer Miner^1^, Christopher Graves^1^, Sung Won Choi^1^, Zhenke Wu^1^, Srijan Sen^1^, Angelle Sander^2^

^1^University of Michigan, Ann Arbor, Michigan, USA, ^2^TIRR Memorial Hermann, Houston, USA

*Journal of Patient-Reported Outcomes 2026*, **10(Suppl 1)**:1016

### Aims

The study aimed to assess the impact of a personalized mobile health intervention, the CareQOL app, on the health-related quality of life (HRQOL) of care partners for individuals with traumatic brain injury (TBI). The app focused on promoting positive health behaviors through self-monitoring and personalized self-care push notifications.

### Methods

A randomized controlled trial (RCT) was conducted with 257 care partners of individuals with TBI. Participants were divided into two groups: 128 in a self-monitoring only group and 129 in a self-monitoring plus personalized self-care push notifications group. The trial included a baseline assessment and a 6-month home monitoring period featuring daily HRQOL patient-reported outcome measures (PROs; caregiver strain, depression, anxiety), monthly HRQOL domain assessments, and continuous monitoring of physical activity and sleep using a Fitbit®. An acceptability survey was also included at the end of month 6, and HRQOL surveys were also conducted at 3- and 6-months post-home monitoring. A subset of 36 participants participated in semi-structured interviews to explore their experiences with the app.

### Results

The addition of personalized self-care push notifications did not significantly enhance HRQOL, physical activity, or sleep duration compared to self-monitoring alone. However, approximately one-third of participants experienced clinically meaningful HRQOL improvements, one-third remained stable, and one-third experienced decreased HRQOL; engagement with the study was a predictor of HRQOL improvements. High compliance rates were noted across all study components, with an 84% completion rate for daily PROs, 90% for Fitbit®-based step counts, and 75% for Fitbit®-based sleep duration. Interviews revealed that the app fostered self-awareness and reflection but inaccuracies in the data and misaligned push notification content sometimes undermined users’ trust in the app and reduced its perceived helpfulness.

### Conclusion

This study concludes that participant engagement, rather than mere compliance, is crucial for improving HRQOL through mHealth interventions. Optimizing engagement can enhance the effectiveness of such interventions. Future designs should focus on aligning and synergizing app components to better serve the target population’s needs. This study provides valuable insights and lessons for the development of effective mHealth interventions for care partners of individuals with TBI.

## 1017 Using a Multiple-Case Study Design to Explore the Experiences of Caregivers of Children with Rare Disease: Lessons Learned from a Study in Leukocyte Adhesion Deficiency, Type 1 (LAD-I)

Meaghan O’Connor^1^, Clarissa Simas^2^, Miranda Bailey^3^, Michael Keith^3^, Maria Chitty Lopez^3^, Leslie Blake^3^, Kristi Jackson^2^

^1^IQVIA, Providence, Rhode Island, USA, ^2^IQVIA Quality Metric, Providence, Rhode Island, USA, ^3^Rocket Pharmaceuticals, Cranbury, New Jersey, USA

*Journal of Patient-Reported Outcomes 2026*, **10(Suppl 1)**:1017

### Aims

Although rare diseases impact approximately 400 million people globally, the small populations within each disease require researchers to use innovative methods, such as case studies, to generate evidence. This study highlights the use of a multiple-case study design to capture the experiences of caregivers of children with severe Leukocyte Adhesion Deficiency, type 1 (LAD-I), an ultra-rare disease marked by recurrent, often life-threatening infections and hyper-inflammation.

### Methods

Using purposive sampling, we leveraged the study sponsor’s existing network of LAD-I families and clinicians. Parents from 6 families with 1 or more child with severe LAD-I were identified and invited to participate in one-on-one, semi-structured interviews. Interview transcripts were analyzed using memoing (reflective writing following each interview) and thematic coding to identify themes across and singularities within participant experiences.

### Results

Nine parents representing 5 families (cases) participated in this study. Four of the families included both parents, each of whom participated in one interview. One family included one parent, who participated in 2 interviews. Families were based in North America and the United Kingdom. Within the families were 7 children with severe LAD-I who ranged in age from 3 to 18 years. The parents reported on their experiences with at least 1 of 3 treatment approaches. Findings (themes) were organized into 3 categories: the burdens of illness, caregiving, and treatment. Although each child’s disease pathway was unique, there were many shared experiences among the families (particularly pre-treatment), including recurrent health issues, complicated journeys to diagnosis, reorganization of life around LAD-I, and the need for physical and social restrictions to reduce infection risk.

### Conclusion

To our knowledge, this is the first time a multiple-case study has been used to study the experience of caregivers of children with severe LAD-I. Key to the success of this approach included the use of: purposive sampling to recruit, 2 interviews per family to provide additional perspectives, and memoing and inductive/deductive coding as analysis methods. The multiple-case methodology allowed the study team to delve deeply into the experiences of families living with severe LAD-I, shedding light on this rare condition and demonstrating the utility of this method in rare disease.

## 1018 Consumer perspectives towards patient reported measures in clinical quality registries

Rasa Ruseckaite^1^, Chethana Mudunna^2^, Belinda Gabbe^2^, Ilana Ackerman^2^, Susannah Ahern^2^

^1^Monash University, Melbourne, Australia, ^2^School of Public Health and Preventive Medicine, Monash University, Melbourne, Victoria, Australia

*Journal of Patient-Reported Outcomes 2026*, **10(Suppl 1)**:1018

### Aims

Clinical quality registries (CQRs) systematically monitor quality of health care within specific clinical domains by routinely collecting, analysing and reporting health-related information from patients who undergo certain medical procedures. Collecting patient reported measures (PRMs) in CQRs provides a personal perspective on the expectations and impacts of treatment; however, reporting CQR-collected PRMs data to patients and consumers is highly variable. This project aimed to elucidate facilitators, barriers, and key lessons learned regarding PRMs implementation and reporting in Australian CQRs.

### Methods

We conducted semi-structured qualitative interviews with 15 consumer representatives in Australia from January to March 2025. Interviews were transcribed and inductive-deductively coded. Coded excerpts were aggregated to (1) identify major themes and (2) analyse how themes interacted.

### Results

All study participants were familiar with PRMs data collection in CQRs and agreed that PRMs were a valuable tool because it enables patients to engage in a process of self-reflection. The interviewees believed that regular updates and feedback on their data would encourage future participation in CQR-PRMs programs and improve response rates. The study participants preferred to receive annual reports of their own PRMs data either online, or via real-life dashboards. These reports should include a lay-language summary accompanied by infographics and pictograms to represent data visually, making complex results easily understandable at a glance. The interviewees thought that CQRs should collaborate with patient organisations, and co-design PRM education and training programs for clinicians and patients. Everyone agreed that representation from culturally diverse groups and Indigenous Peoples was crucial.

### Conclusion

Our findings indicate the need for regular CQR-PRMs reporting and clear presentation of the data to consumers and patients. Next steps of our study will involve creating a user manual for clinicians and consumers on how to visualise and interpret CQR-PRMs. Partnerships with consumer organisations in CQRs will continue to be established to assist with improving patient outcomes, experiences and delivery of care by drawing on the knowledge, skills and experiences.

## 1019 Clinical Symptom Profiles for People with Multiple Sclerosis

Deborah Miller^1^, Yadi Li^2^, Scott Husak^2^, Dan Ontaneda^3^, Robert Bermel^3^, Brittany Lapin^2^

^1^Mellen Center, Cleveland Clinic, Cleveland, Ohio, USA, ^2^Cleveland Clinic, Quantitative Health Sciences, Cleveland, Ohio, USA, ^3^Cleveland Clinic, Mellen Center, Cleveland, Ohio, USA

*Journal of Patient-Reported Outcomes 2026*, **10(Suppl 1)**:1019

### Aims

Our study aimed to identify clinical profiles of patients with multiple sclerosis (PwMS) based on performance measures and self-reported health-related quality of life. MS affects multiple areas of a person’s life. Understanding how functioning and symptoms are experienced will help drive more targeted and effective interventions.

### Methods

A retrospective cohort study was conducted including adult PwMS treated at an academic MS-specialty center between 9/2015-2/2024. As standard care, PwMS completed performance measures (processing speed test [PST], manual dexterity test [MDT], and walking speed test [WST]) as well as 10 Neuro-QoL domains. Latent profile analysis identified clinical profiles based on these 13 outcomes. A multivariable multinomial logistic regression model was constructed to evaluate differences in the profiles based on demographics and clinical characteristics.

### Results

There were 8,952 adult PwMS included in the study (average age 48.9±13.1, 72.7% female, average disease duration of 14.6±12.2 years). Latent profile analysis identified 4 unique profiles: Profile 1 (“No Symptoms”, n=1765 (19.7%)), Profile 2 (“Moderate Symptoms”, n=4,543 (50.7%)), Profile 3 (“Mixed Symptoms”, n =381 (4.3%)), and Profile 4 (“Significant Symptoms”, n=2,263 (25.3%)). PwMS in Profile 3 had the worst scores on MDT, WST, Neuro-QoL Upper and Lower Extremity, but better scores than Profiles 2 and 4 on Neuro-QoL Cognitive Function, Depression, Anxiety, Fatigue, and Sleep Disturbance. Compared to Profile 1, PwMS in Profiles 3 and 4 were more likely to have Primary Progression (PP) MS, be of black race, unemployed, on Medicare/Medicaid, and living at home with assistance. Profile 4 was also more likely younger, female, had more comorbidities and higher area deprivation.

### Conclusion

PwMS have distinct clinical profiles which could be utilized for targeted symptom-based interventions. While Profile 4 had significant impairment on all domains, Profile 3 had poor mobility and function but average cognition, fatigue, and emotional symptoms. Further investigation of factors that contribute to Profile 3’s affective well-being may provide useful guidance in the management or prevention in those labelled as Profile 4.

**Fig. 1 (abstract 1019):**
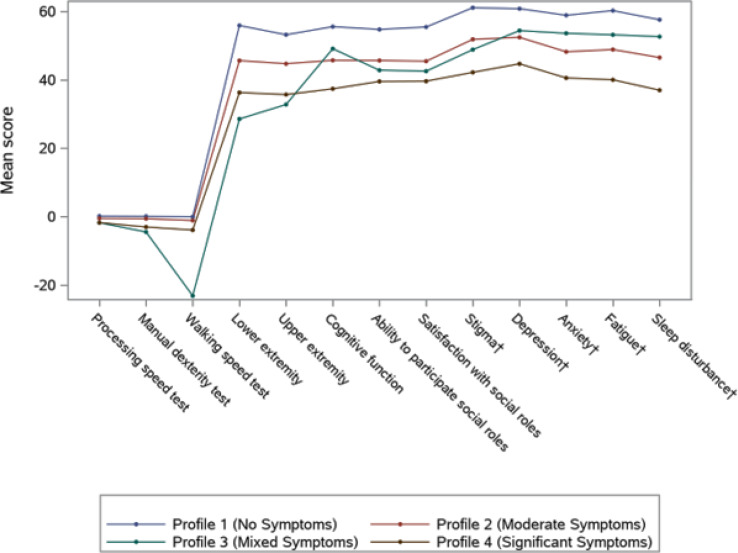
Clinical Symptom Profiles for People with Multiple Sclerosis

## 1020 Meaningful within-patient change of PROMIS-Fatigue in patients with cancer cachexia in the phase 2 ponsegromab study

Jarjieh Fang^1^, Joshua Roth^1^, Andrew Bushmakin^2^, Magdalena Harrington^1^, John Groarke^3^, Susie Collins^4^, Jeffrey Crawford^5^, Eric Roeland^6^, Joseph Cappelleri^2^

^1^Pfizer, New York, New York, USA, ^2^Pfizer, Groton, Connecticut, USA, ^3^Pfizer, Cambridge, Massachusetts, USA, ^4^Pfizer, Kent, North Carolina, UK, ^5^Duke Cancer Institute, Durham, USA, ^6^Knight Cancer Institute, Portland, USA

*Journal of Patient-Reported Outcomes 2026*, **10(Suppl 1)**:1020

### Aims

A meaningful within-patient change (MWPC) threshold is the difference between clinical outcomes assessment scores, such as patient-reported outcomes, that is considered meaningful to patients. This study estimated MWPC for the PROMIS-Fatigue 7a instrument in patients with cancer cachexia based on data from a recent randomized trial (NCT05546476; Groarke et al, NEJM, 2024).

### Methods

This prespecified analysis used blinded patient data from the randomized, double-blind, phase 2 trial in adults diagnosed with solid tumors and cachexia (by Fearon criteria). For PROMIS-Fatigue 7a, raw scores range from 7 to 35, with T-scores based on a mean of 50 and a standard deviation of 10 (for the general US population). MWPC thresholds were estimated using an anchor-based analysis. Patient Global Impression of Severity (PGI-S) of physical fatigue and Patient Global Impression of Change (PGI-C) in physical fatigue were used as anchors. Effect sizes were calculated by dividing MWPC values by the corresponding baseline standard deviation scores of the instrument.

### Results

Among the overall trial population (n=187), the median age was 67 (IQR 60-74) years, 63% of patients were male, and 40%, 32%, and 29% had non-small-cell lung, pancreatic, and colorectal cancer, respectively. In total, 95% of patients had received systemic anticancer therapy, ranging from 1 to ≥4 lines of treatment. Analyses based on PGI-S and PGI-C included all available longitudinal data from 148 patients, respectively. Values for MWPC were 4.63 (raw scoring; PGI-S based) and 2.50 (raw scoring; PGI-C based) when using a 2-category change in the anchor; MWPC estimations in terms of T-scores were 7.15 and 3.83. Regarding the effect sizes, these changes can be interpreted as “large” (PGI-S-based) and “medium” (PGI-C-based). Data are summarized in Table 1. The relationship between change from baseline in PROMIS-Fatigue and change in PGI-S or PGI-C was approximately linear (Figure 1).

### Conclusion

When a 2-point change in the anchor is used to define MWPC in cancer cachexia, values of 4.63 and 2.50, scored in the original units, can be considered MWPCs for PROMIS-Fatigue. Values of 7.15 and 3.83 can be considered MWPCs for PROMIS-Fatigue when represented as T-scores.

**Table 1 (abstract 1020):**
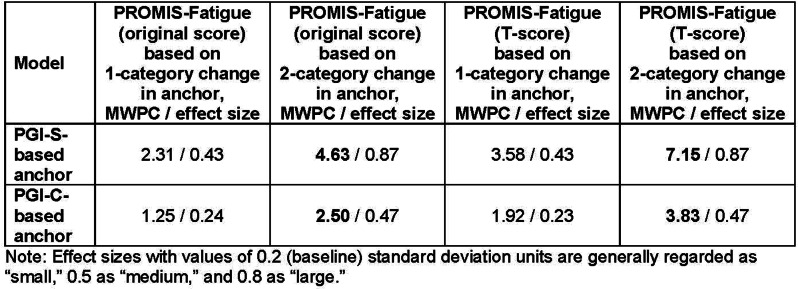
MWPC estimates

**Fig. 1 (abstract 1020):**
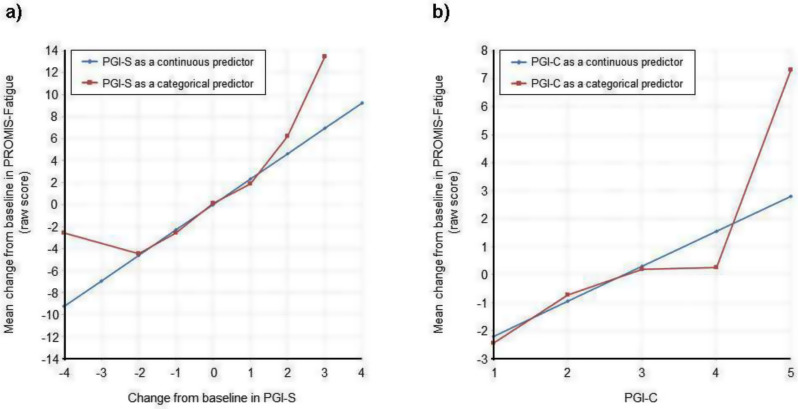
Relationship between changes in PROMIS-Fatigue and changes in PGI-S (**a**) or PGI-C (**b**)

## 1021 Idiopathic multicentric Castleman disease symptom burden study (ISBUS): Emerging findings and insights

Philip Powell^1^, Anju Keetharuth^1^, Sé Maria Francis^1^, Jill Carlton^1^, Antonio Adolfo Guerra Soares Brandão^2^, Karthik Ramasamy^3^, Francis Shupo^4^, Kelley Dacus^5^, Yildiz Kelahmetoglu^6^, Kelly Makarounas-Kirchmann^7^, Annmarie Bosco^8^, Satyen Gohil^9^, H. Miles Prince^10^, Sudipto Mukherjee^11^

^1^University of Sheffield, Sheffield, UK, ^2^Hospital BP - A Beneficência Portuguesa de São Paulo, São Paulo, Brazil, ^3^Oxford University Hospitals NHS Foundation Trust, Oxford, UK, ^4^Recordati, Hemel Hempstead, UK, ^5^Recordati, Bridgewater, USA, ^6^Recordati, Hemel Hempstead, UK, ^7^KMC Healthcare, Mt Eliza, Australia, ^8^Prince of Wales Hospital, Sydney, Australia, ^9^University College London Hospitals NHS Foundation Trust, London, UK, ^10^University of Melbourne, Melbourne, Australia, ^11^Cleveland Clinic Taussig Cancer Institute, Cleveland, USA

*Journal of Patient-Reported Outcomes 2026*, **10(Suppl 1)**:1021

### Aims

Idiopathic multicentric Castleman Disease (iMCD) is a rare, life-threatening non-neoplastic lymphoproliferative disorder characterized by generalized lymphadenopathy and a chronic hyper inflammatory state. Patients with iMCD have a high symptom burden, which adversely impacts their quality of life (QoL). The iMCD symptom burden study (ISBUS) is a multi-institution international study that aims to develop a novel patient-reported outcome measure (PROM) to quantify this symptom burden. This will help in real-time dynamic monitoring of symptoms and assessing response to treatment interventions.

### Methods

A four-stage development process is being followed, including: (i) drafting items; (ii) cognitive interviews; (iii) psychometric survey; and (iv) preliminary measures of change. Stage (i) and (ii) are complete and (iii) is ongoing. In stage (ii), the content validity of draft content of the PROM including 42 items was assessed through cognitive interviews with 10 people living with iMCD. In stage (iii), a revised version of the PROM is being administered online, alongside other QoL measures, to a sample of 50+ people living with iMCD. Due to the rarity of the condition and to enhance reach, a multi-country approach is being taken, involving patients from the UK, US, Canada, Australia, New Zealand, and Brazil. Advisory groups include people with lived experience of iMCD, clinicians, researchers, and industry representatives.

### Results

From 42 potentially relevant symptom items tested in cognitive interviews, 30 were retained. Participants found the draft PROM comprehensive. Most of the PROM was well-understood, with minor revisions made to the instructions and certain items (e.g., “shortness of breath” rather than “difficulty breathing”). Most retained symptoms were considered relevant, with those less relevant (e.g., “vomiting”) omitted from the scale. To date, seventeen participants have been recruited in the psychometric survey (US=8, UK=6, Australia=2, Canada=1) with recruitment ongoing.

### Conclusion

The ISBUS study will produce a novel PROM for symptom burden in iMCD, representing an international collaboration of patients, clinicians, and academic and industry experts. Initial testing has helped inform the PROM and establish preliminary evidence for comprehensibility, relevance, and comprehensiveness. Results of the stage (iii) psychometric survey will be used to further refine the instrument.

## 1022 Lessons learned from the development and implementation of an electronic patient-reported outcomes platform (MyPRO) in a Toronto community health clinic serving racialized immigrant and refugee women

Kirsten Wade^1^, Rob Fredericksen^2^, Edmund Scacchitti^3^, Mary Ndung’u^4^, Majorie Kabahenda^4^, Wangari Tharao^4^, Notisha Massaquoi^5^, Gauri Inamdar^6^, Roger Prasad^6^, Sarah Mixson^2^, Nayan Kalnad^7^, Sangeeta Patil^8^, Aaron Pond^8^, Heidi Crane^2^, Duncan Short^9^

^1^Syneos Health, Morrisville, North Carolina, USA, ^2^University of Washington, Seattle, Washington, USA, ^3^ViiV Healthcare, Durham, North Carolina, USA, ^4^Women’s Health in Women’s Hands, Toronto, Ontario, Canada, ^5^University of Toronto, Toronto, Ontario, Canada, ^6^The Ontario HIV Treatment Network, Toronto, Ontario, Canada, ^7^Avegen Health, London, UK, ^8^TCC Health, London, UK, ^9^ViiV Healthcare, London, UK

*Journal of Patient-Reported Outcomes 2026*, **10(Suppl 1)**:1022

### Aims

We developed and piloted a web-based patient-reported outcomes platform (MyPRO) in a Toronto clinic primarily serving racialized women with immigrant and refugee status, both living with and without HIV. We report lessons learned along the implementation process.

### Methods

MyPRO is a clinic-facing platform that administers and summarizes responses from an electronic patient-reported outcomes (ePRO) assessment. Patients complete ePROs remotely, using links sent via email or SMS, or in-person on clinic tablets. ePRO responses are automatically summarized for providers before the appointment. We monitored implementation progress via weekly study meetings and solicited stakeholder feedback (e.g. value and acceptability of MyPRO; integration into workflow) through in-person semi-structured interviews with staff (n=6) and healthcare providers (HCPs; n=4) near the end of the study.

### Results

The MyPRO implementation process described by clinic staff is presented in Figure 1. Pre-implementation training was highly valued among staff; HCPs felt a separate session tailored to provider roles would be beneficial. Implementation was adapted throughout to meet the specific needs of the clinic population (e.g. varying English fluency; high digital and research hesitancy). Staff emphasized the importance of trust and face-to-face support to address these needs, including the value of a peer navigator to facilitate recruitment and ePRO completion. Some clinic staff felt MyPRO integration added time to patient visits due to unexpected high reliance on in-person ePRO completion or due to time spent discussing patient results. One observation included some patients completing the ePRO after their appointment rather than before. These patients were offered follow-up telehealth visits to discuss results if deemed necessary by their HCPs. Patients were subsequently requested to arrive at the clinic earlier to complete the ePRO beforehand, helping to streamline the visit. The MyPRO summary sheet was clear and easy to use, and MyPRO was especially useful for identifying sensitive situations (e.g. intimate partner violence, housing insecurity). Clinic staff, leadership, and HCPs valued MyPRO’s impact on care, including encouraging patient reflection on health experiences and facilitating HCP awareness of unanticipated patient priorities.

### Conclusion

MyPRO implementation was facilitated by clinic staff flexibility and added value to patient care in a population demonstrating significant digital and research hesitancy.

**Fig. 1 (abstract 1022):**
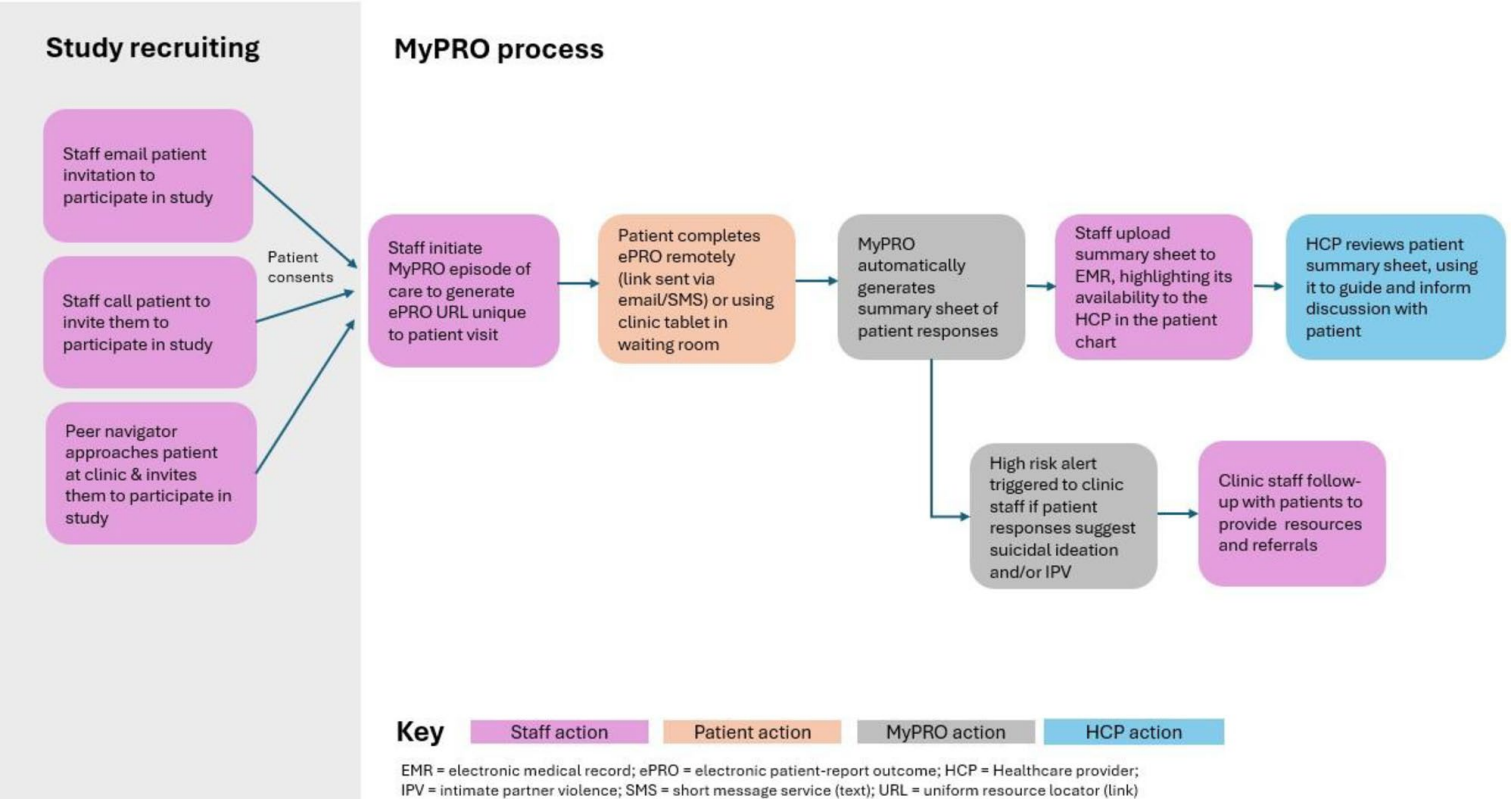
MyPRO implementation process

## 1023 Promoting professional well-being through resilience and self-care: A qualitative study

Sabrina Figueiredo^1^, Eliezer Oliveira^2^, Karen Schlump^3^, Kathleen Ennis-Durstine^4^

^1^The George Washington University, Washington, District Of Columbia, USA, ^2^Children’s National Hospital, Washington, District Of Columbia, USA, ^3^George Washington University, Washington, Maryland, USA, ^4^Children’s National Hospital, Washington, District Of Columbia, USA

*Journal of Patient-Reported Outcomes 2026*, **10(Suppl 1)**:1023

### Aims

Chaplains at Children’s National Hospital (CNH) developed the Comfort Corner (CC) program to promote the well-being of healthcare professionals through resilience and self-care. This initiative supports the mind, body, and spirit by offering peer support, relaxing videos, snacks, aromatherapy, and soothing music. Comfort Corner operates in quiet rooms across inpatient units for 90 minutes once a week, with a chaplain available for emotional and spiritual support. To ensure the program’s effectiveness, it is essential to tailor it based on insights from stakeholders regarding their needs and challenges, as well as to identify barriers and facilitators to participation. This study explores the needs of healthcare professionals and the factors that influence attendance at Comfort Corner.

### Methods

Employees from one Pediatric Teaching Hospital participated in this observational, cross-sectional study, which was reviewed and deemed exempt by the Institutional Review Board (IRB). 480 respondents participated in a voluntary electronic survey administered via REDCap between February and May 2023. Open-ended questions were used to assess needs, barriers, and facilitators. Content analysis was conducted to identify common themes in the responses.

### Results

Among the respondents who visited CC in the past year, 267 completed the survey on needs, barriers, and facilitators. 92% indicated that CC met their needs, frequently citing reasons such as the importance of self-care, a relaxing environment, and feeling appreciated. For those who felt that CC didn’t meet their needs, the main reasons included busy workloads and its unavailability during weekend and night shifts. A few respondents expressed a desire for a more comprehensive program that could offer support beyond what CC provides. Reported barriers to program’s attendance included a heavy patient load. Conversely, having backup nurses facilitates their attendance at CC.

### Conclusion

Comfort Corner (CC) addresses the needs of healthcare professionals working in a pediatric hospital. Although heavy patient loads make it difficult to attend CC, colleagues who support one another help facilitate attendance. While attending CC is beneficial, offered alone it is not sufficient to promote professional well-being. Therefore, these efforts should be complemented by organizational changes.

## 1024 Mediating effects of diabetes distress on glycemic level and health-related quality of life among patients with type 2 diabetes mellitus: a cross-sectional study

Yiqing Weng^1^, Xinhui Xu^1^, Huayu Li^1^, Xiaoyuan Jin^1^, Fengyang Jiang^1^, Hongmei Wang^1^

^1^Department of Social Medicine of School of Public Health, and Department of Pharmacy of the First Affiliated Hospital, Zhejiang University School of Medicine, Hangzhou, China

*Journal of Patient-Reported Outcomes 2026*, **10(Suppl 1)**:1024

### Aims

Type 2 Diabetes Mellitus (T2DM) requires long-term medication and lifestyle management and may have serious complications, is prone to causing mental burden and psychological stress, which negatively impact patients’ Health-Related Quality of Life (HRQOL). This study aims to examine the mediating role of diabetes distress in the relationship between glycemic control and HRQOL, based on Wilson and Cleary’s health-related quality of life model.

### Methods

A cross-sectional questionnaire survey was conducted among patients with T2DM attending diabetes or endocrinology outpatient clinics in three general hospitals in Zhejiang Province. A total of 450 valid questionnaires were collected. The questionnaires included basic patient information (demographic characteristics, health/disease information, and most recent blood glucose results), Diabetes Specific Quality of Life Scale (DSQL), and Diabetes Distress Scale (DDS). The correlation analysis used the Spearman rank correlation test. Structural Equation Modeling (SEM) was employed to test the proposed hypotheses, particularly focusing on the mediating effect of diabetes distress.

### Results

Among the surveyed patients, 10.89% experienced moderate or severe diabetes distress. Correlation analysis revealed a positive association between diabetes distress and glycemic levels (r1 = 0.240, P < 0.001). Additionally, diabetes distress was positively correlated with DSQL scores (r2 = 0.315, P < 0.001), indicating that higher distress levels were linked to lower HRQOL. Glycemic control had a direct positive predictive effect on DSQL scores (β = 0.159, P < 0.001); diabetes distress partially mediated between patients’ glycemic level and DSQL score (β = 0.071, P < 0.001), accounting for 30.87% of the total effect.

### Conclusion

Although the proportion of T2DM patients experiencing moderate or severe diabetes distress was relatively low in this study, diabetes distress significantly affected all dimensions of HRQOL. Regular screening for diabetes distress and timely intervention might reduce the negative impact of patients’ glycemic levels on HRQOL, ultimately improving patient well-being.

## 1025 Intraclass Correlation Coefficients in Multilevel Structural Validation of Intensively Collected Questionnaires: A Targeted Literature Review

Christina Daskalopoulou^1^, Aleksandra Sjöström-Bujacz^2^, Jakob Bjørner^3^, Dara O’Neill^4^

^1^IQVIA, Athens, Greece, ^2^IQVIA, Solna, Sweden, ^3^IQVIA, Copenhagen, Denmark, ^4^IQVIA, Barcelona, Spain

*Journal of Patient-Reported Outcomes 2026*, **10(Suppl 1)**:1025

### Aims

The last decade has seen increases in intensive longitudinal data (ILD) collection via clinical outcome assessments (COAs), including daily diaries and digital measures. These ILD COAs can capture within- and between-person variability. Intraclass correlation coefficients (ICCs) are important metrics for assessing variability at both levels and indicating the need for multilevel structural analytical strategies. This targeted literature review (TLR) aimed to collate insight on within vs between-person variability via ICC values within ILD studies that adopted a multilevel framework.

### Methods

Search terms included ‘intraclass correlation coefficient’ together with ‘ecological momentary assessment (EMA)’, ‘experience sampling’, ‘digital’, ‘diary’, ‘ambulatory assessments’ and ‘multilevel’, ‘hierarchical’ structural validity. Searches were conducted in PsycNet, PsycInfo, PubMed, and Web of Science as of November 11, 2024. Additional papers were identified through snowballing.

### Results

Initial searching identified 55 unique papers. Abstract screening provided a shortlist of 6 papers, and 14 papers were identified through snowballing. For these 20 papers, sample sizes ranged from 49 to 2,104 participants; the total number of observations ranged from 377 to 29,950, but reporting was inconsistent. Most studies centered on daily diaries, followed by EMA, and diaries with less frequent assessment. Studies covered a wide array of topics, most commonly mental health. ICCs were highly variable with most studies reporting the range or average of these values. Based on the reported ICC range, 15% were below 0.20, 53% were between 0.20 and 0.50, and 30% were between 0.50 and below 0.85, with the median value being 0.40. Most models were multi-factorial at both within- and between-person levels, with over 50% observing the same number of factors at both levels.

### Conclusion

Many ILD studies found that a significant portion of the COA variance is attributable to within-person variation. These findings highlight the need for multilevel modeling strategies that investigate both between- and within-person variation, providing insights into whether the latent structure of the COA may vary between levels. Additionally, reporting practices in papers, abstracts, and keywords need standardization, to enhance the comparability of future research by improving the identification of applied modelling techniques.

## 1026 Regional influences on quality of life in leukaemia patients: uncovering key predictors for personalised care

Sam Salek^1^, Sarah Gunn^2^, Aj Poots^2^, Esther Oliva^3^, Tatyana Ionova^4^, Samantha Nier^5^

^1^University of Hertfordshire, Hatfield, UK, ^2^Picker Institute Europe, Oxford, UK, ^3^London North West University Healthcare NHS Trust, London, UK, ^4^Saint Petersburg State University Hospital, Saint Petersburg, Russia, ^5^Acute Leukaemia Advocates Network (ALAN), Bern, Switzerland

*Journal of Patient-Reported Outcomes 2026*, **10(Suppl 1)**:1026

### Aims

Quality of life (QoL) is a key element when caring for patients with leukaemia, influencing treatment choices and well-being. The Haematological Malignancy Patient-Reported Outcome (HM-PRO) is a validated tool for measuring QoL of patients with hematologic cancers, including leukaemia. It comprises HM-PRO Part-A (physical & psychosocial well-being), and HM-PRO Part-B (symptoms). The research question was: can identifying predictive factors for QoL and symptom burden inform practice and policy for patient-centred care? This study therefore aimed to examine whether region-based analyses identify these predictions.

### Methods

Using a global online cross-sectional survey to explore patient experiences and perceptions, we recruited patients with different types of leukaemia. Statistical analysis was carried out using Gradient Boosting Decision Trees (GBDT) to identify predictive factors for QoL dimensions and symptom burden, using subsets for European and North American respondents.

### Results

All 1993 patients completed the HM-PRO Part-A (mean age=54.5, median (Mdn)=58, range=17-92; female=1102, 55.3%) and 1951 patients (mean age=54.6, Mdn=58, range=17-92; female=1075, 55.1%) completed Part-B. The HM-PRO classifications for the entire data set were made with moderate accuracy (HM-PRO Part-A: 54.0%, AUC 0.81; HM-PRO Part-B: 47.5%, AUC 0.75). Isolation and depression were strong predictors. Regional analyses revealed variability in model performance, and found different key factors: HM-PRO Part-A: Europe (n=851): Accuracy 58.5%, AUC 0.82; key factors: isolation, year diagnosed, general anxiety, depression, disease-related anxiety. North America (n=289): Accuracy 49.5%, AUC 0.67; key factors: general anxiety, isolation, symptoms, year of diagnosis. HM-PRO Part-B: Europe (n=836): Accuracy 52.3%, AUC 0.78; key factors: symptoms, isolation, treatment length, general anxiety. North America (n=286): Accuracy 50.0%, AUC 0.76; key factors: symptoms, side effect frequency, depression, isolation, having to ask for results.

### Conclusion

Although the models showed low accuracy, the high AUC suggests potential in identifying key QoL predictive factors for leukaemia patients. In Europe, isolation and disease-related anxiety were prominent predictors, suggesting psychosocial aspects strongly influence QoL. In North America, symptoms, depression, and side-effect frequency were key factors. These variations emphasise a need for personalised care strategies and can inform clinical practice and policy to improve QoL across diverse populations. However, the low sample sizes limit generalisability.

## 1027 Mind mapping: an accessible solution for qualitative data collection and visual data summarization and dissemination

Kirsten Glynn^1^, Upal Basu Roy^2^, Aurora Lucas^3^, Rasika Bombatkar^4^, Gloria Arroya^5^, Tendai Chihuri^6^, Bellinda King-Kallimanis^7^

^1^LUNGevity, Boston, Massachusetts, USA, ^2^LUNGevity Foundation, Bethesda, Maryland, USA, ^3^Patient Advocate, Romeoville, Illinois, USA, ^4^Patient Advocate, Oxford, UK, ^5^Patient Advocate, San Juan, Puerto Rico, USA, ^6^LUNGevity, Bethesda, Maryland, USA, ^7^LUNGevity, Bethesda, USA

*Journal of Patient-Reported Outcomes 2026*, **10(Suppl 1)**:1027

### Aims

To present mind-mapping as a method for data collection and data summarization/dissemination in qualitative research.

### Methods

We conducted three Focus Groups (FGs). Two FGs were conducted with survivors of non-small cell lung cancer (NSCLC) and one with care partners to survivors of NSCLC. The FG discussions centered on five areas of unmet needs. During each FG, mind maps were created in situ displaying categories and sub-categories as they emerged. Participants were asked to review, clarify, or correct the mind maps in real time to ensure that they reflected the speakers’ intentions. Researchers iteratively distilled the mind map concepts across FGs to create meta mind maps for each area of need. The mind mapping methodology occurred alongside traditional qualitative coding and analysis.

### Results

Important considerations for using mind mapping throughout the study included:1) Data collection: stimulation and engagement of discussion with participants while maintaining focus on the central research question(s).2) Data analysis/summarization: the process of creating meta mind maps added a complementary, iterative layer to analysis that aided in conceptualizing themes and relationships between concepts, solidifying researcher consensus.3) Data dissemination: mind maps offered a snapshot of the FGs that was accessible and intuitive, allowing immediate collaboration with team members as well as efficient and accessible dissemination of research findings.

### Conclusion

Patient-focused research aims to engage patients and care partners throughout research, but there are limited methods for visualizing qualitative data. Mind mapping, a proven tool in other disciplines, has high applicability in health care. It provides an engaging way to interact with dense data across different phases of research from collection to dissemination. The mind map framework allowed patients, team members, and our partners to quickly and intuitively understand the unmet needs of participants, bolstering it as a candidate for further consideration in patient-focused research endeavors including PROM development and clinical trial exit interviews in addition to broad patient-focused health research. Mind maps also have high utility as dissemination aids because visually appealing formats lead to deeper engagement on the content, from patients considering unmet needs to clinic staff considering how to develop survivorship programs.

**Fig. 1 (abstract 1027):**
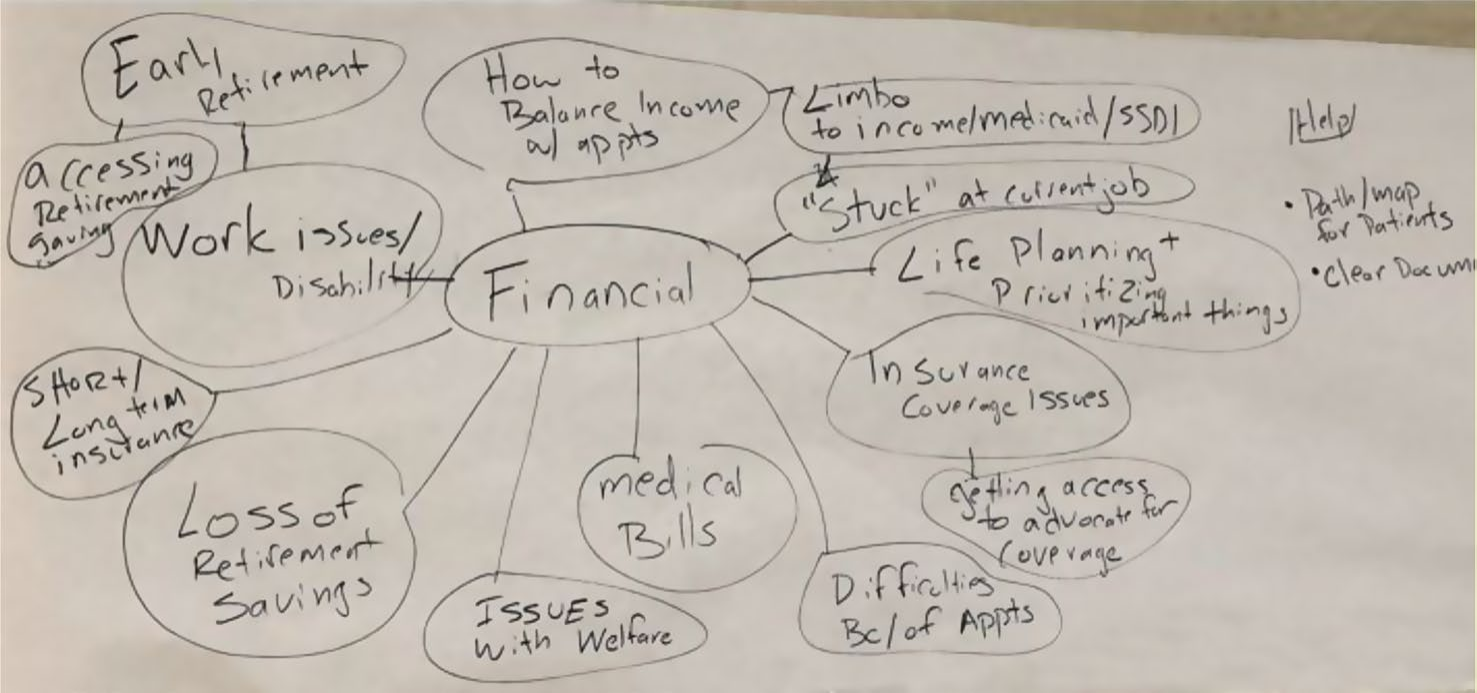
Mind map for financial needs made during focus group

**Fig. 2 (abstract 1027):**
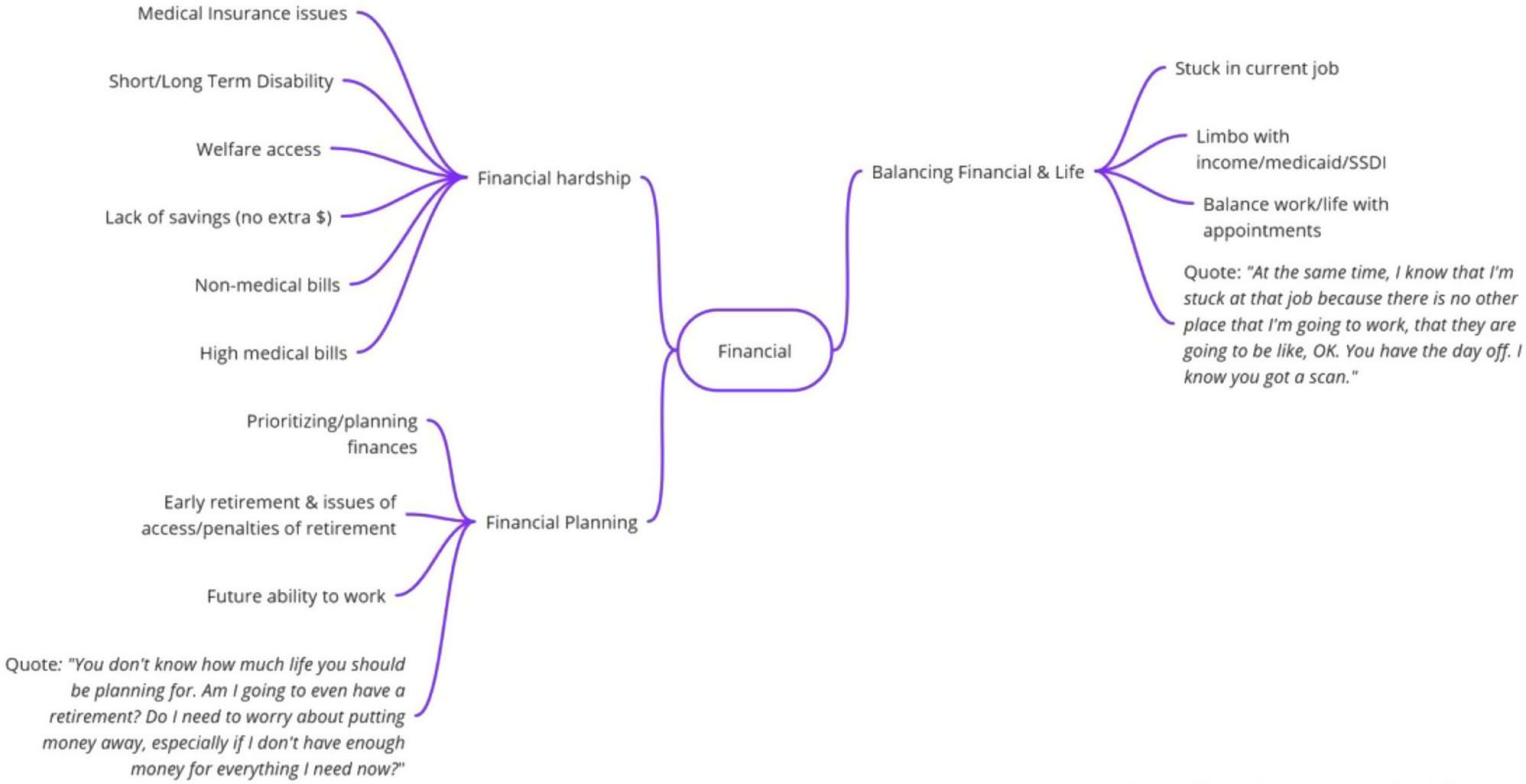
Meta Financial Needs Mind Map

## 1028 Assessing CFA model fit criteria in data conditions resembling clinical trials: A simulation study

Brandon Foster^1^

^1^Lumanity, Boston, Massachusetts, USA

*Journal of Patient-Reported Outcomes 2026*, **10(Suppl 1)**:1028

### Aims

Psychometric evaluation of multi-item clinical outcome assessments often uses confirmatory factor analysis (CFA) to validate proposed scoring algorithms based on clinical trial data. However, widely cited fit criteria, such as those by Hu and Bentler (1999), assume normally distributed continuous data and moderate-to-large samples, conditions rarely met in clinical trial psychometric analyses. This study investigates how item skewness, sample size, and estimation method influence the performance of commonly used CFA fit indices (CFI, TLI, RMSEA, SRMR).

### Methods

A simulation study employed a two-factor CFA model (12 items, 6 per factor, r = 0.50) with three skewness levels (none, moderate, high), six sample sizes (50–175), and two estimation methods (ML, WLSMV). Each scenario tested the correct two-factor model plus two misspecified models—a unidimensional model (all items on one factor) and a two-factor model with cross-loadings (λ = 0.40)—across 500 replications. The distributions of fit indices were analyzed against conventional performance criteria.

### Results

Fit indices performed unevenly across conditions. Increased skewness notably reduced false acceptance rates of misspecified cross-loading models using ML estimation. For non-skewed data, false acceptance rates for CFI and RMSEA were high (~25–40% and ~22–36%, respectively), but these rates decreased substantially under high skewness (~13–19% and ~15–19%). SRMR showed consistently low false acceptance (0–3%) regardless of skewness. WLSMV estimation was less affected by skewness and maintained reliable performance with correctly specified models down to N = 100, but consistently demonstrated liberal acceptance compared to ML when using conventional performance criteria.

### Conclusion

Conventional approaches to evaluating CFA model fit are unsuitable, particularly when data are skewed or WLSMV estimation is employed. Traditional fit criteria may be too liberal under these conditions. Researchers must carefully select and justify fit criteria based explicitly on the characteristics of their data, such as skewness and sample size.

**Fig. 1 (abstract 1028a):**
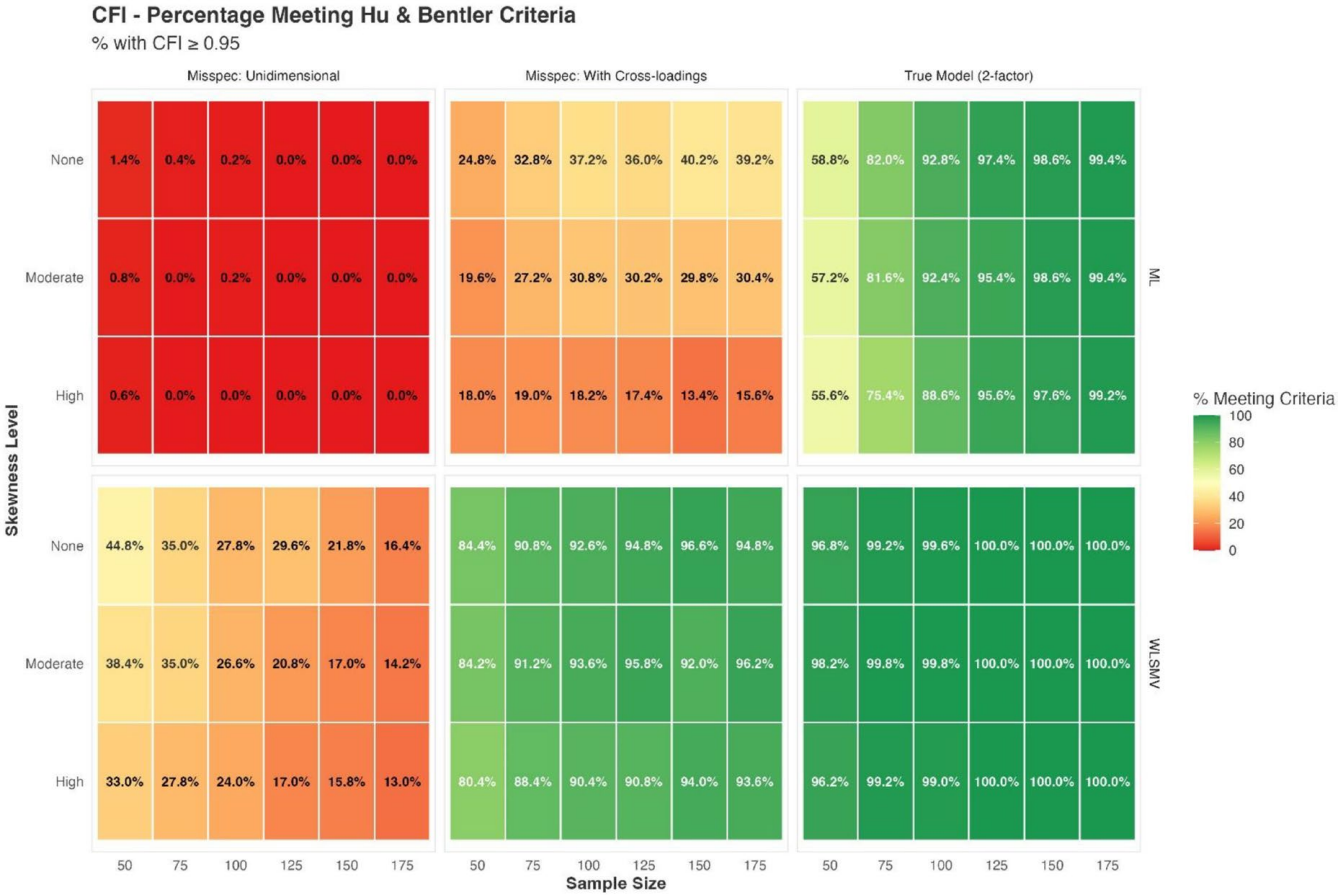
Assessing CFA model fit criteria in data conditions resembling clinical trials: A simulation study

**Fig. 1 (abstract 1028b):**
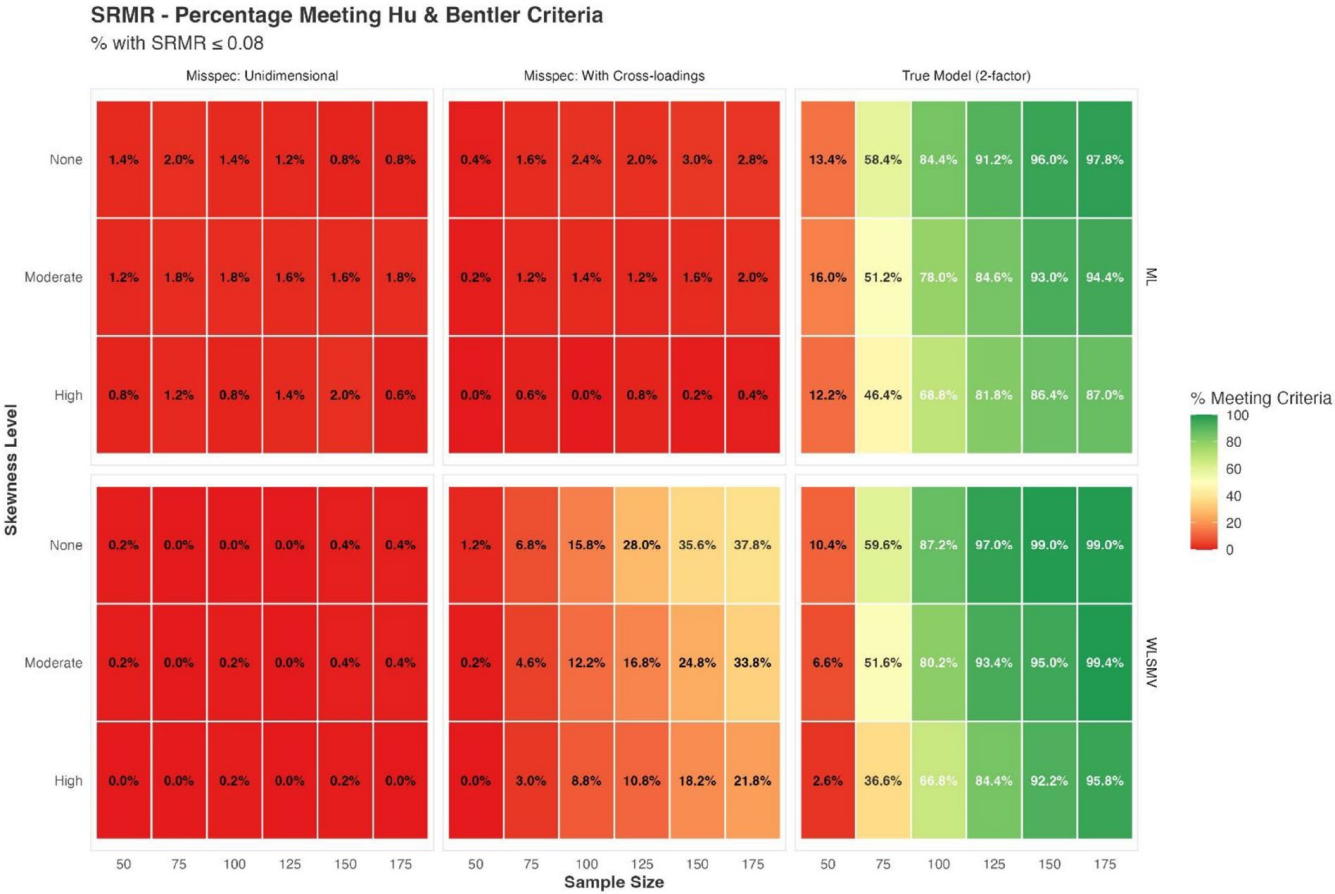
Assessing CFA model fit criteria in data conditions resembling clinical trials: A simulation study

**Fig. 1 (abstract 1028c):**
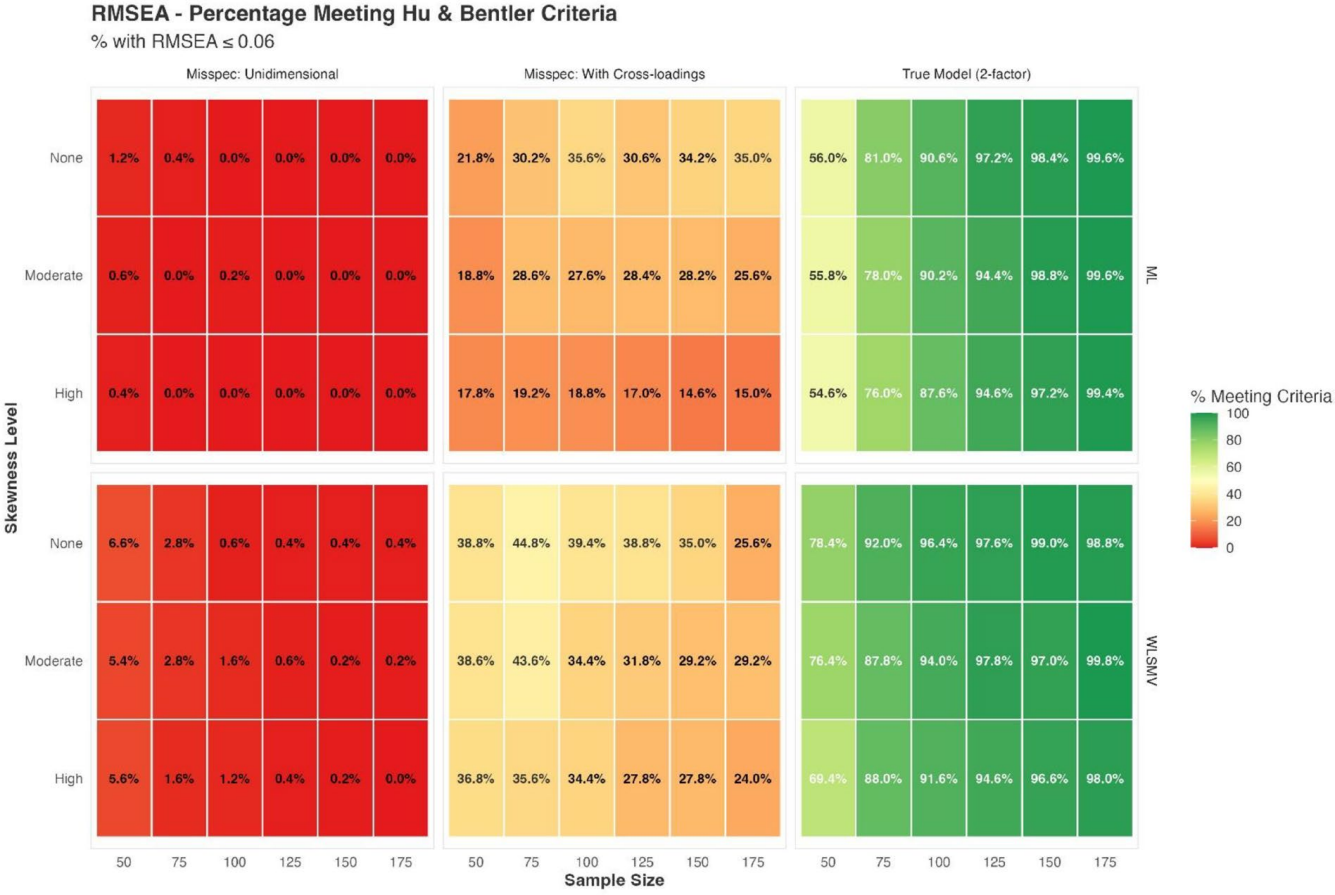
Assessing CFA model fit criteria in data conditions resembling clinical trials: A simulation study

## 1029 Incorporating Qualitative Data as Evidence into Rare Disease Clinical Development Programs: Leveraging the Rare Disease Clinical Outcome Consortium

Nicola Williamson^1^, Cara O’Neill^2^, Hafiz Oko-osi^3^, Lindsey Murray^4^, Asha Hareendran^5^

^1^UCB, London, Arizona, UK, ^2^Cure Sanfilippo Foundation, Columbia, South Carolina, USA, ^3^BioMarin, Los Angeles, California, USA, ^4^Critical Path Institute, Tucson, Arizona, USA, ^5^University of Bedfordshire, London, UK

*Journal of Patient-Reported Outcomes 2026*, **10(Suppl 1)**:1029

### Aims

To document how qualitative scientific methods have been used as evidence to support regulatory (FDA and EMA) and health technology assessment (HTA) bodies decisions.

### Methods

The Rare Disease Clinical Outcome Consortium (RD-COAC) is a pre-competitive, multi-stakeholder collaboration which aims to drive consensus on best practices for methodological challenges associated with rare disease research. Experts in medical product development, clinical outcome assessment (COA) measurement, regulatory science, and patient advocates worked together to examine drug approvals, paying particular attention to how qualitative research can be used to support COA-based endpoints by healthcare decision makers to determine the value of new treatments for rare disease. This example can be used as a resource to inform stakeholders and decision makers for future trials using qualitative research.

### Results

Leveraging member experiences in a neutral, program-agnostic setting, the RD-COAC identified case studies to illustrate how qualitative research within and alongside clinical trials has been used as evidence to identify concepts of interest, support the selection of patient relevant COA-based endpoints, and inform the interpretation of benefit–risk assessment from the patient and caregiver perspective. One study was explored to assess how qualitative data was used in their trial program. Evidence from qualitative research in Phase 2 and 3 studies (n=11 and n=35) illustrated the relevance of bowel movement frequency as a key concept of interest, justifying its use as the primary endpoint to inform regulatory approval (FDA and EMA) and by HTA bodies (UK NICE, France HAS). Additionally, evidence from qualitative research informed the definition of within-patient meaningful change thresholds for the primary endpoint (FDA).

### Conclusion

Scientific evidence based on qualitative research has been used in rare disease clinical development programs by both regulatory and HTA bodies to inform decision-making for medical product approvals. Pre-competitive, multistakeholder collaborations help advance rare disease medical product development. Sharing expertise across multiple disciplines enhances methodological problem-solving and reduces risk to individual sponsors.

## 1030 A Mixed Methods Analysis Using Trial Data to Describe Patients with Glycogen Storage Disease Type Ia Who Met Baseline Expectations for Meaningful Change in Daily Cornstarch Intake

Diane Turner-Bowker^1^, Shayna Egan^1^, Deepali Mitragotri^1^, Blaise Cureg^2^, Martha Gauthier^2^

^1^Ultragenyx Pharmaceutical, Novato, California, USA, ^2^Lumanity, Boston, Massachusetts, USA

*Journal of Patient-Reported Outcomes 2026*, **10(Suppl 1)**:1030

### Aims

A mixed methods analysis was conducted using qualitative and quantitative data from a phase 3 clinical trial evaluating an investigational treatment for glycogen storage disease type Ia (GSDIa) to determine whether individual patient-level baseline expectations for clinically meaningful change on the primary endpoint were met at the efficacy timepoint.

### Methods

Data were from a phase 3, randomized, double-blind, placebo-controlled trial (NCT05139316) in pediatrics (8 to <18 years) and adults (≥18 years) with GSDIa assessing efficacy/safety of DTX401, an investigational adeno-associated virus serotype 8 vector designed to express the human G6PC1 gene. Patient-level expectation for meaningful percent reduction in daily cornstarch intake collected during phase 3 baseline interviews were compared with patient-level actual percent reduction in daily cornstarch intake at Week 48 and at Week 96. Each patient was then categorized as having not met, met, or exceeded baseline expectation. Results were summarized by treatment group. The average change in daily cornstarch intake was described for the group that met or exceeded baseline expectations. Sample quotes from baseline, Week 48, and Week 96 interviews describe the patient experience.

### Results

A higher proportion of DTX401-treated patients met or exceeded baseline expectations for meaningful reduction in daily cornstarch intake at Week 48 versus placebo patients. Similarly, a high proportion of DTX401-treated patients met or exceeded baseline expectations for meaningful reduction in daily cornstarch intake in the crossover period at Week 96. Adult and pediatric patients treated with DTX401 commonly described positive quality of life benefits associated with reduced daily cornstarch intake.

### Conclusion

Findings from this mixed methods research offer a novel approach for investigating clinically meaningful change in key trial endpoints at the patient level. Results from this research align with patient feedback on what would constitute a meaningful reduction in daily cornstarch intake gathered during a phase 3 protocol planning advisory board and yield impactful patient experience results demonstrating the potential treatment benefit of DTX401.

## 1031 Migration inconsistencies in electronic Quality of Life (QOL) instruments in clinical trials

Lindsay Hughes^1^, Jowita Marszewska^2^

^1^IQVIA, New York, New York, USA, ^2^IQVIA, Cleveland, Ohio, USA.

*Journal of Patient-Reported Outcomes 2026*, **10(Suppl 1)**:1031

### Aims

Quality of life (QOL) questionnaires can be collected electronically (eCOA) instead of on paper. The well-defined migration process ensures the original COA’s measurement properties are retained. However, inconsistencies like minor mistranslations, formatting changes, or screen navigation issues are common across a range of countries and languages. These inconsistencies can affect data quality, reduce credibility, and discourage patients from completing questionnaires. This research focuses on migration-related challenges in less common languages compared to those frequently used in QOL instruments in clinical trials.

### Methods

Trials were chosen based on the availability of COAs measuring QOL and presence of common and less common languages. Language pairs understood as common include Spanish (US), German (Germany), Japanese (Japan), and French (Canada and France). Less common languages are understood as languages used in countries where eCOA is not popular or unofficial or immigrant language pairs for example Haitian Creole (US), Vietnamese (US), Cebuano (Philippines), Armenian (Armenia), Farsi (Iran).

### Results

Spanish, German, Japanese, and French languages were more common in the eCOA trials under consideration and, as a result, were overrepresented. 35 inconsistencies were found in Spanish, 102 in German, 9 in Japanese, 76 in French (mean of 8 inconsistencies per trial for common languages), 62 in Haitian Creole, 43 in Vietnamese, 1 in Cebuano, 22 in Armenian, and 83 in Farsi (mean of 42 inconsistencies per trial for less common languages). Inconsistencies were present in items, response scales, and the navigation buttons. There were more QOL instrument versions in the common languages so more opportunity to identify inconsistencies, but that there were any is worthy of note. On average, there were more inconsistencies per trial in less common languages. These languages often face significant challenges. For example, while common languages have many well-trained linguists familiar with clinical trials, less common languages often rely on fewer trained linguists or non-specialized ones. This can lead to delays and the exclusion of these languages from trials.

### Conclusion

It’s essential to ensure that patients feel that eCOA is tailored specifically for them, including the use of appropriate language, to avoid introducing cultural bias and jeopardizing the integrity of the study and its endpoints.

## 1032 Psychometric Properties of the Subcutaneous Administration Assessment Questionnaire (SQAAQ)

Chisom Kanu^1^, Fangyu Wang^1^, Anthony Zagar^1^, Mingyang Shan^1^

^1^Eli Lilly and Company, Indianapolis, Indiana, USA

*Journal of Patient-Reported Outcomes 2026*, **10(Suppl 1)**:1032

### Aims

Several currently available obesity management medications are injectables. However, there is currently no patient-reported outcome measure for assessing injection device usability that has been validated in individuals living with obesity. The Subcutaneous Administration Assessment Questionnaire (SQAAQ) is a 12-item, self-administered questionnaire used to assess the ease of use of a medical device and the participant’s confidence while using the device. This study aimed to assess the psychometric properties of SQAAQ in adults living with obesity.

### Methods

The SQAAQ was administered at Week 4 to participants in a phase 3 obesity trial for retatrutide (NCT05882045). Responses to each item were on a 7-point Likert scale ranging from “strongly disagree” (scored as 1) to “strongly agree” (scored as 7). Higher scores indicate greater ease of use and confidence with using the subcutaneous injection device. Internal consistency of the items was assessed using Cronbach’s alpha and an Exploratory Factor Analysis (EFA) was conducted to evaluate the number of concepts the questionnaire captured and the structure of the questionnaire.

### Results

Cronbach’s alpha for the 12 questions was 0.98. An EFA showed a strong underlying single factor (eigenvalue 1 =54.6 with 98.2% of the variance represented, eigenvalue 2 = 1.50). A single factor model showed factor loadings ranging from 0.76 to 0.95.

### Conclusion

The items in the SQAAQ showed high internal consistency in individuals participating in an obesity trial. The EFA suggests that the items evaluate several aspects of one primary concept, ease of device use. The SQAAQ is a reliable PRO measure to assess ease of device use for medications administered subcutaneously in obesity clinical trials.

## 1033 Patient-reported walking difficulty predicting the post-discharge overall function in patients with lung cancer undergoing minimally invasive surgery

Xin Tian^1^, Cheng Lei^2^, Hongfan Yu^3^, Wei Dai^4^, Xing Wei^4^, Qiuling Shi^5^

^1^ChongQing Medical University, Mianyang, China, ^2^Sichuan Cancer Hospital & Institute, Sichuan Cancer Center, Affiliated Cancer Hospital of University of Electronic Science and Technology of China, Chongqing, China, ^3^State Key Laboratory of Ultrasound in Medicine and Engineering, College of Biomedical Engineering, Chongqing Medical University, No. 1, Medical College Road, Yuzhong District, Chongqing 400016, China, ^4^Department of Thoracic Surgery, Sichuan Clinical Research Center for Cancer, Sichuan Cancer Hospital & Institute, Sichuan Cancer Center, Affiliated Cancer Hospital of University of Electronic Science and Technology of China, Chengdu, Sichuan, China, ^5^School of Public Health, Chongqing Medical University, Chongqing, China

*Journal of Patient-Reported Outcomes 2026*, **10(Suppl 1)**:1033

### Aims

Postoperative mobility of patients with lung cancer is crucial for their physical rehabilitation. This study aimed to identify severe walking difficulty by a threshold and predicting the functional recovery of patients with lung cancer undergoing minimally invasive surgery (MIS).

### Methods

This prospective study enrolled patients with lung cancer who underwent MIS, divided into two cohorts. The 0–10-scale walking difficulty score was assessed daily during hospitalization following surgery and weekly for 4 weeks after discharge. Chi-square and receiver operating characteristic curve analyses guided to define the threshold, with the Timed Up and Go Test on postoperative day 2 as an anchor in cohort 1. The European Organization for Research and Treatment of Cancer Quality of Life Questionnaire Core 30 was assessed biweekly for 1 month post-discharge. Post-discharge functional status trajectories were compared based on the threshold.

### Results

Based on cohort 1, the threshold for walking difficulty was set to 4. Cohort 2 patients were categorized using the threshold: 71.26% exhibited no or mild, while 28.74% experienced severe walking difficulty upon discharge. Compliance rates for reporting walking difficulty post-discharge consistently exceeded 60%. Significant differences in post-discharge physical function (P<0.001), emotional function (P=0.008), role function (P<0.001), and quality of life (P=0.033) were observed among patients with differing walking difficulty severities.

### Conclusion

A patient-reported walking difficulty score of ≥4 indicates severe walking difficulty. Significant differences in post-discharge functional status were observed among patients with different walking difficulty degrees. Intensive care for severe walking difficulty is instructive for post-discharge functional recovery.

**Table 1 (abstract 1033):**
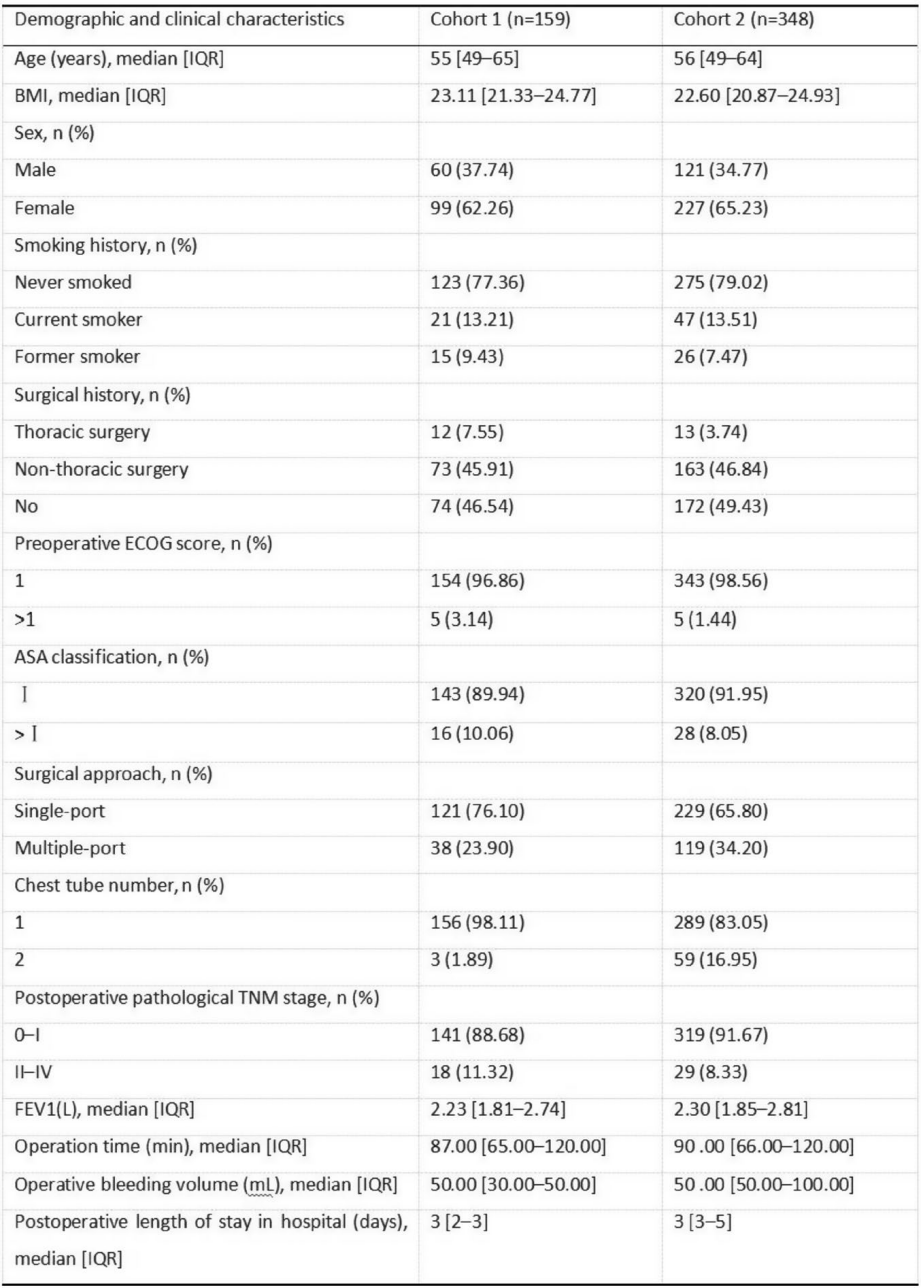
Patient demographics and clinical characteristics in cohorts 1 and 2

**Table 2 (abstract 1033):**
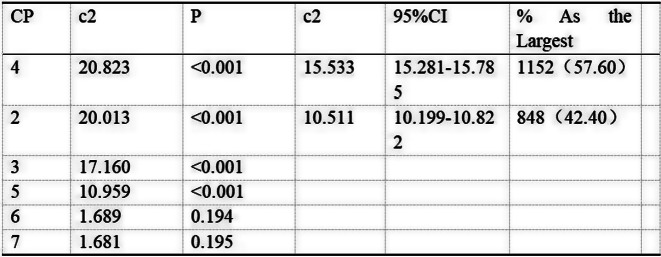
Optimal CP Analysis Based on the TUGT and Bootstrap with 2000 Resamplings for CPs of Walking Difficulty

**Fig. 1 (abstract 1033):**
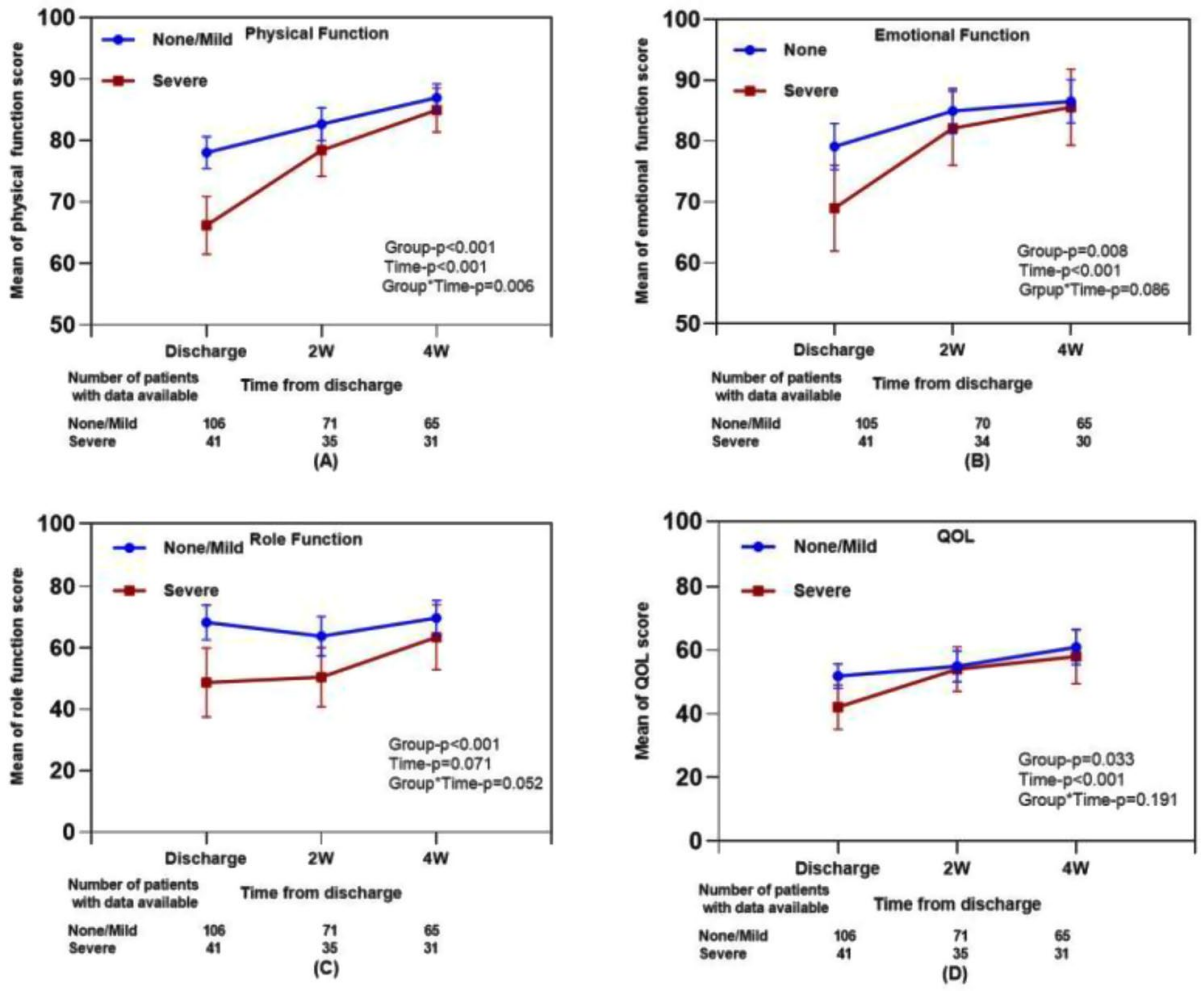
Mean score of Physical, Emotional, and Role Function score, QOL from discharge, defined by the optimal CP. Adjusted variables: age, postoperative length of stay in hospital, FEV1

## 1034 Association between socio-economic status and health-related quality of life in patients with relapsed/refractory multiple myeloma treated in countries with universal healthcare system and the mediating role of financial difficulties

Francesco Sparano^1^, Gianluca Gaidano^2^, Pasquale Niscola^3^, Serena Puglia^1^, Katia Codeluppi^4^, Elisabetta Antonioli^5^, Catello Califano^6^, Lajos Floro^7^, Ombretta Annibali^8^, Claudio Fozza^9^, Agostino Tafuri^10^, Patrizio Mazza^11^, Leonardo Potenza^12^, Marco Vignetti^1^, Michele Cavoi^13^, Maria Teresa Petrucci^14^, Fabio Efficace^1^

^1^Italian Group for Adult Hematologic Diseases (GIMEMA), Data Center and Health Outcomes Research Unit, Rome, Italy, ^2^Division of Hematology, Department of Translational Medicine, University of Eastern Piedmont, Novara, Italy, ^3^Hematology Unit, Sant’Eugenio Hospital, Rome, Italy, ^4^Hematology Unit, Azienda Unità Sanitaria Locale-IRCCS di Reggio Emilia, Reggio Emilia, Italy, ^5^Hematology Unit, Careggi University Hospital, Florence, Italy, ^6^Onco-Hematology Unit, “A. Tortora” Hospital, Pagani, Italy, ^7^Haematology Department, University College London Hospital, London, Italy, ^8^Hematology, Stem Cell Transplantation, Fondazione Policlinico Universitario Campus Bio Medico, Rome, Italy, ^9^Department of Medicine, Surgery and Pharmacy, University of Sassari, Sassari, Italy, ^10^Azienda Ospedaliera Sant’Andrea, Rome, Italy, ^11^Haematology, Ospedale G. Moscati, Taranto, Italy, ^12^Hematology Unit, Azienda Ospedaliera Universitaria di Modena, Department of Medical and Surgical Sciences, University of Modena and Reggio Emilia, Modena, Italy, ^13^RCCS Azienda Ospedaliero-Universitaria di Bologna, Istituto di Ematologia “Seràgnoli”, Dipartimento di Medicina Specialistica, Diagnostica e Sperimentale, Università di Bologna, Bologna, Italy, ^14^Hematology, Department of Translational and Precision Medicine, Azienda Ospedaliera Policlinico Umberto I, Sapienza University of Rome, Rome, Italy

*Journal of Patient-Reported Outcomes 2026*, **10(Suppl 1)**:1034

### Aims

We assessed the association between socio-economic status (SES) and the health-related quality of life (HRQoL) profile of patients with relapsed/refractory multiple myeloma (RRMM). A secondary objective was to explore if financial difficulties mediated the relationship between SES and HRQoL.

### Methods

We conducted a cross-sectional analysis of a prospective observational study involving RRMM patients in Italy and the UK. SES was determined by scoring education level (0 = low, 1 = intermediate/high), living arrangements (0 = living alone, 1 = living with others), and employment status (0 = no income from a salary/pension, 1 = receiving a salary/pension). Patients were classified into low (score 0-1), middle (score 2), and high SES (score 3). Mean differences in the EORTC QLQ-C30 scores between patients with low SES vs middle/high SES were assessed, while adjusting for key potential confounding factors. The clinical relevance of between-groups differences was evaluated according to the criteria identified by Cocks et al (J Clin Oncol 29:89-96, 2011). Mediation analysis was conducted to investigate the role of financial difficulties in mediating the relationship between SES and overall HRQoL (i.e., QLQ-C30 summary score).

### Results

Overall, 505 RRMM patients with a median age of 70 (IQR 62-75) were considered for this analysis. More than one-third of patients (35.1%) had a high SES, 49.3% a middle SES, and 15.6% a low SES. Patients with low SES had clinically relevant worse scores in most QLQ-C30 scales (see Table). The largest clinically relevant mean differences between patients with low vs middle/high SES were observed in insomnia (∆ = 14.9 [95% CI, 7.0 to 22.8]; medium clinically relevant difference), financial difficulties (∆ = 12.3 [95% CI, 6.2 to 18.5]; medium clinically relevant difference), physical functioning (Δ = –12.0 [95% CI, -17.8 to -6.3]; small clinically relevant difference), and fatigue (∆ = 10.8 [95% CI, 4.6 to 16.9]; small clinically relevant difference). Overall, financial difficulties mediated 46% of the total effect of SES on HRQoL.

### Conclusion

Low SES is associated with a worse HRQoL profile among RRMM patients even in universal healthcare settings. Financial difficulties partially mediate this relationship, suggesting that lower SES amplifies financial strain, thereby further impairing HRQoL.

**Table (abstract 1034):**
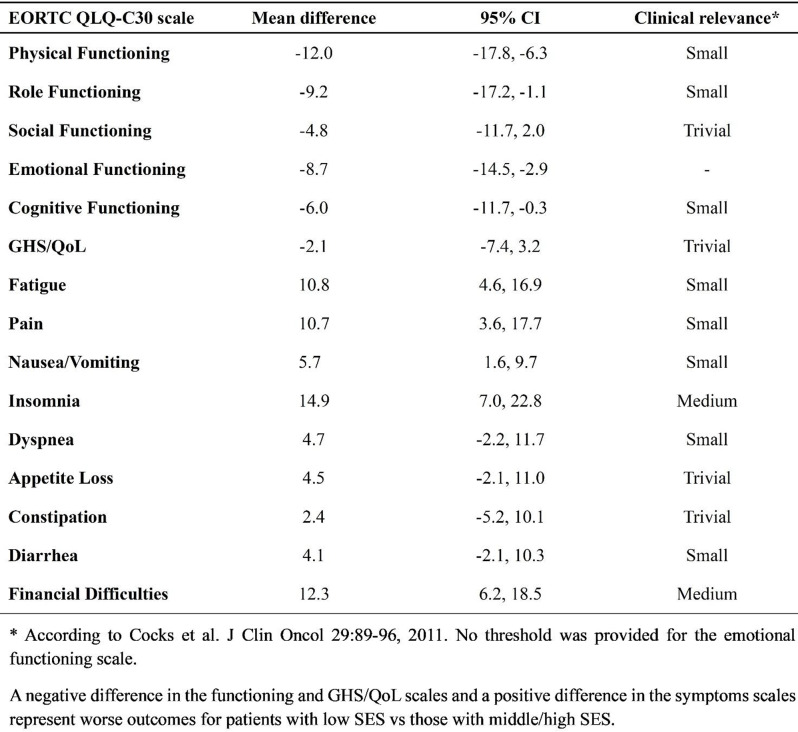
Adjusted Mean Differences in EORTC QLQ-C30 Scales of Patients with Low versus Middle/High Socio-economic status

## 1035 Imputing item-level missingness in PRO data using continuous-time Markov models: a simulation study

Shizhu Li^1^, Qiuling Shi^1^

^1^Chongqing Medical University, Chongqing, China

*Journal of Patient-Reported Outcomes 2026*, **10(Suppl 1)**:1035

### Aims

Item-level missingness frequently occurs in longitudinal patient-reported outcome (PRO) data, potentially biasing analysis results and weakening statistical power. Conventional imputation methods, such as mean imputation or multiple imputation, may not fully capture the ordinal structure or temporal transitions of symptom trajectories. This study evaluates the performance of continuous-time Markov models to impute missing symptom data under both missing at random (MAR) and not missing at random (NMAR) mechanisms.

### Methods

We used a real-world clinical dataset comprising 193 postoperative patients who reported symptoms daily over four consecutive days using 11 core items from the MD Anderson Symptom Inventory (MDASI). Missing data were simulated at a 20% rate for each item under MAR and NMAR assumptions. Two modeling strategies were compared: (1) full-scale modeling using the original 11-point ordinal ratings and (2) a simplified 3-category severity scale (mild, moderate, severe). Imputation was conducted via the R package msm, with Viterbi decoding to reconstruct the most likely state sequences. Covariates including age, gender, BMI, and cancer stage were incorporated into the model.

### Results

Results showed that collapsing ordinal scales consistently improved imputation accuracy and model stability across all items and mechanisms. As illustrated in our MAE, RMSE, and accuracy (within ±1) figures, collapsed models substantially outperformed full-scale models. For instance, in the “fatigue” item under NMAR, the mean absolute error (MAE) dropped from 2.12 to 0.56, and accuracy within ±1 increased from 53% to 96%. Similar improvements were observed for items such as pain, nausea, sleep, and shortness of breath.Under NMAR conditions, full-scale models often yielded inflated RMSEs and poor convergence. In contrast, collapsed models maintained high accuracy and computational stability. Additionally, metrics allowing a tolerance margin (±1 or ±2) provided a more clinically relevant evaluation of imputation performance, especially for ordinal symptom scales.

### Conclusion

In conclusion, Markov model-based imputation is a viable and effective strategy for handling item-level missingness in longitudinal PRO data. Collapsing ordinal states not only enhances numerical stability but also improves imputation accuracy, particularly under NMAR scenarios. This approach enables better reconstruction of symptom trajectories and supports more reliable downstream analyses in clinical research.

**Fig. 1 (abstract 1035a, b, c):**
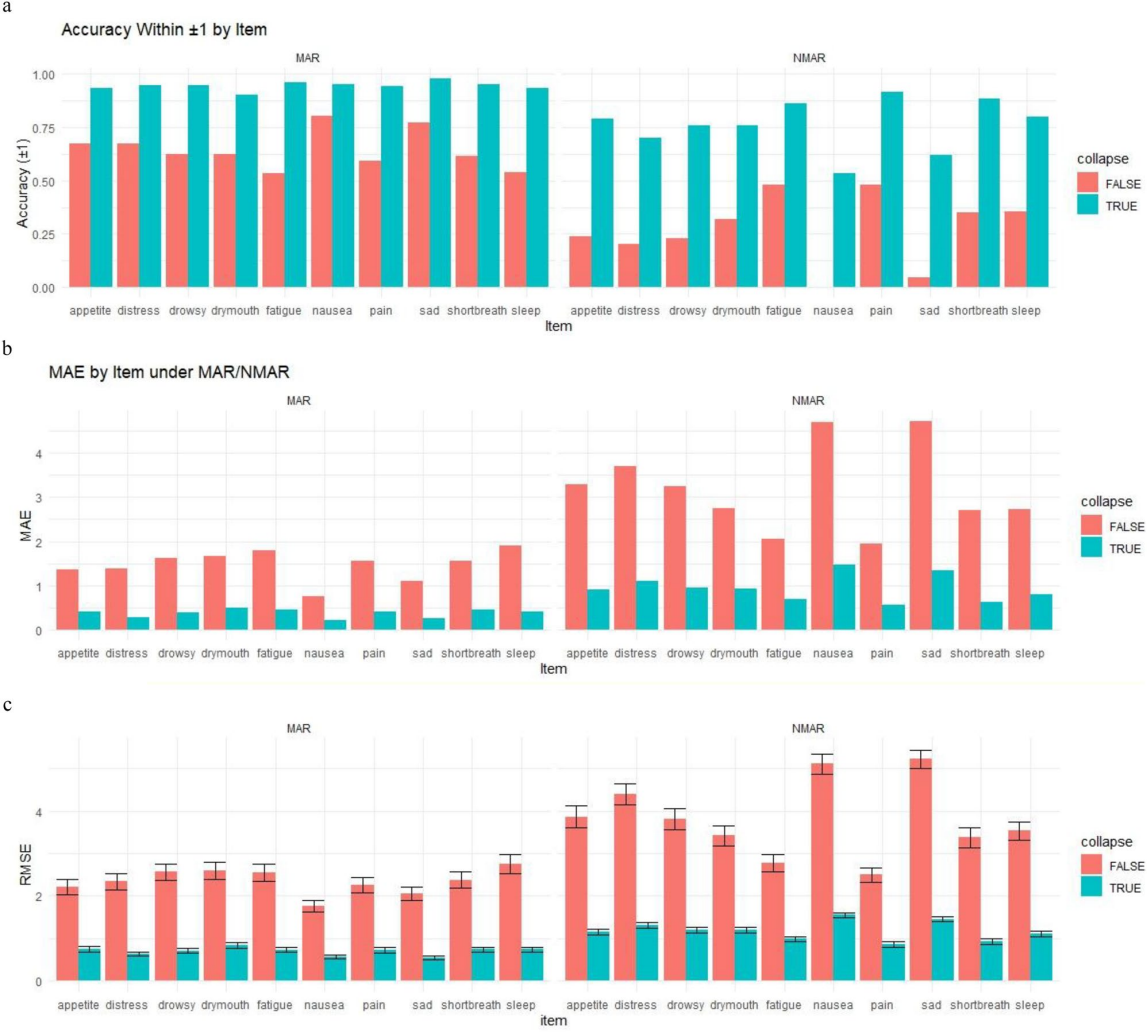
Imputing item-level missingness in PRO data using continuous-time Markov models: a simulation study

## 1036 From Data Overload to Insight: Interactive visualisation of PRO-CTCAE Data with an R Shiny Tool

Alexander Hind^1^, Joel Sims^1^, Rachael Lawrance^1^, Kim Cocks^1^

^1^Adelphi Values, Manchester, UK

*Journal of Patient-Reported Outcomes 2026*, **10(Suppl 1)**:1036

### Aims

Patient-Reported Outcomes version of the Common Terminology Criteria for Adverse Events (PRO-CTCAE) data is now widely collected in oncology trials to evaluate patients’ perspective of symptomatic toxicity over time. Multiple PRO-CTCAE symptoms (items) are collected in trials and interpretation is often made challenging due to the volume of adverse event (AE) items, attributes, timepoints and diverse means to examine PRO-CTCAE data by treatment arm. Our aim was to develop an interactive visualisation tool for multi-stakeholder collaboration to support efficient and accurate interpretation of PRO-CTCAE data.

### Methods

R Shiny was utilised to develop the tool using recommended methods to operationalise and visualise PRO-CTCAE data. It is adaptable to include any of the 124 items available in the PRO-CTCAE library. Descriptive visualisations include stacked bar charts of item responses over time and derived summaries (worst on-treatment score and baseline-adjusted worst score) by treatment arm. The dashboard was designed flexibly with the ability to toggle on/off missing data categories and collapse severity categories, and the use of hover functionality to view category details. The tool is published on a priority and confidential publishing platform. Data presented is from a simulated dataset reflecting typical patterns seen in oncology clinical trials for two treatment arms.

### Results

The tool enables effective review of comparative data for patterns of PRO-CTCAE symptoms over short- and long-term periods. It enables review of the relationship between the symptom attributes (e.g. frequency/severity/interference), exploration of missing data, and presents summary measures such as baseline-adjusted worst scores. The hover functionality can be used on each data point to provide the exact proportion of patients in each response category and the ability to collapse response categories aids interpretation by focusing on the most severe response categories. The dashboard is equipped with different display types (tabs) according to the research question to be addressed as well as interactive functionality options (Figure 1) and response levels are displayed using stacked bar charts (Figure 2).

### Conclusion

Compared to producing a vast number of static bar charts, this R Shiny interactive tool allows more efficient review and interpretation of the totality of the PRO-CTCAE data included in a clinical trial.

**Fig. 1 (abstract 1036a):**
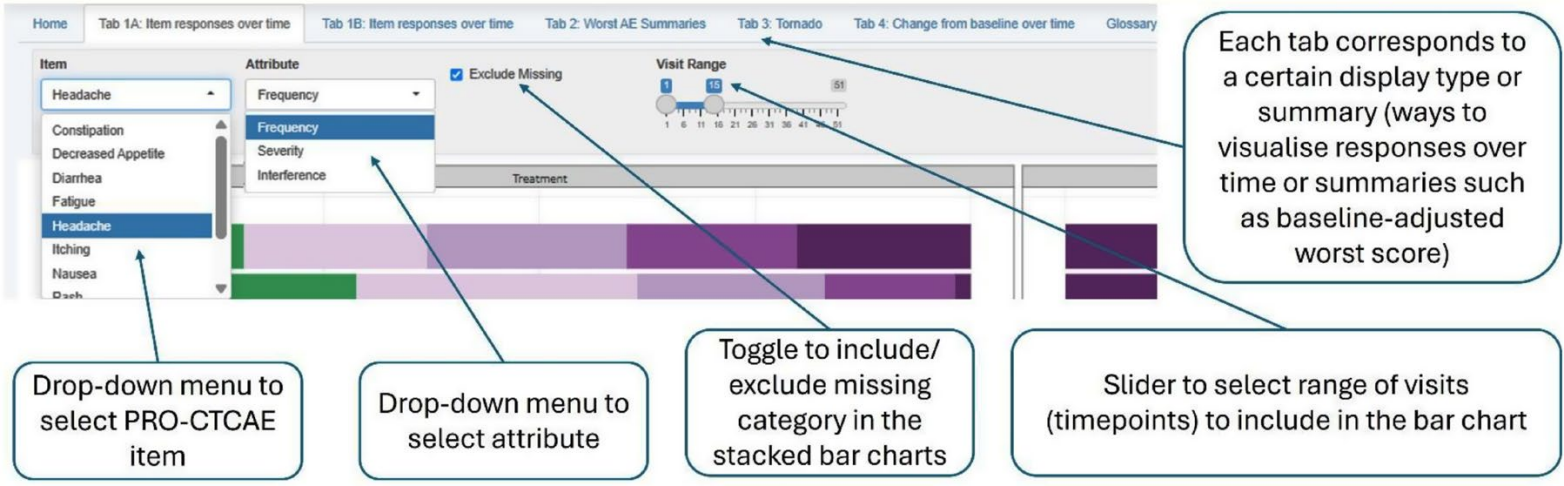
From Data Overload to Insight: Interactive visualisation of PRO-CTCAE Data with an R Shiny Tool

**Fig. 1 (abstract 1036b):**
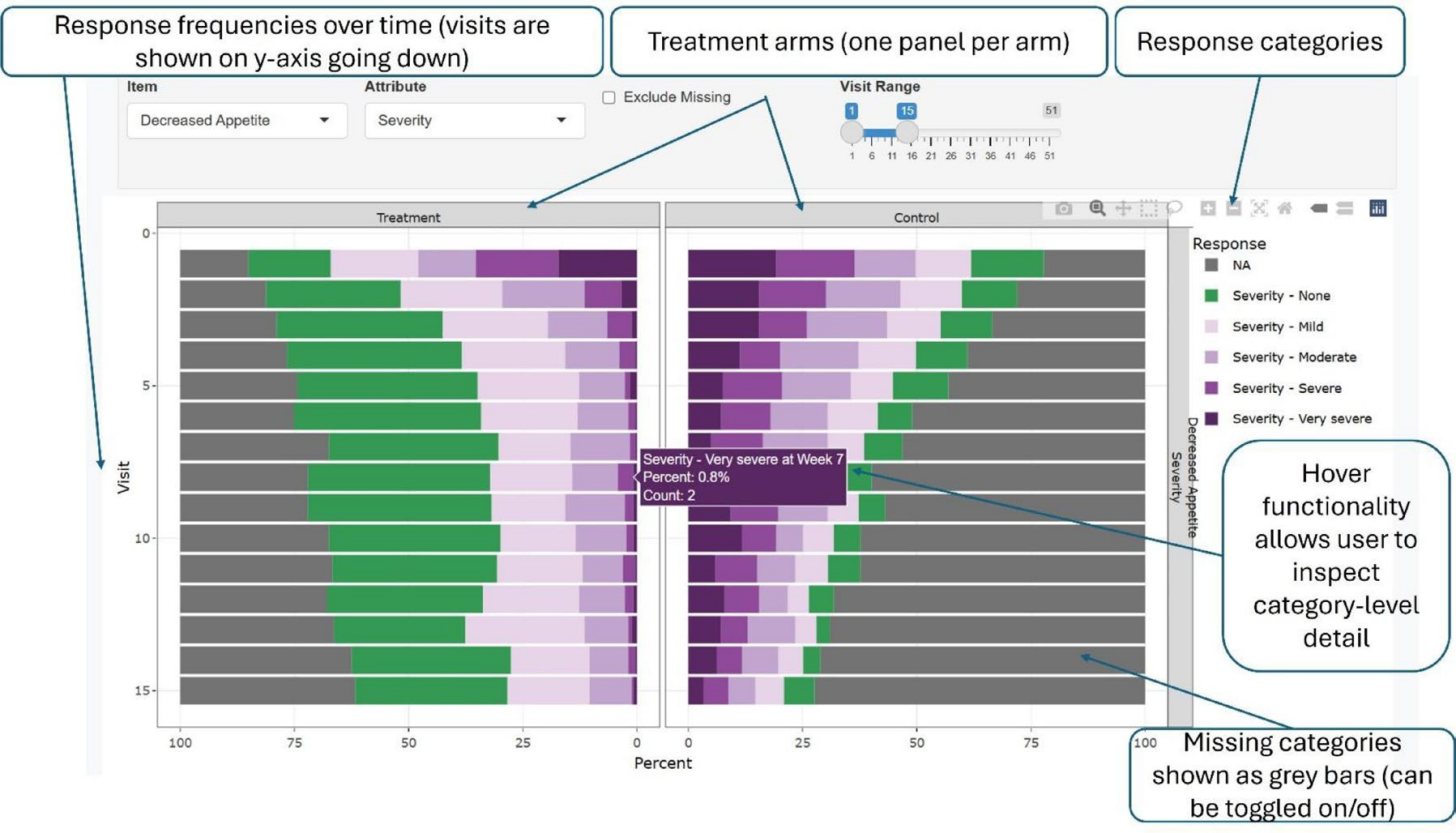
From Data Overload to Insight: Interactive visualisation of PRO-CTCAE Data with an R Shiny Tool

## 1037 Semantic analysis of the multidimensional health locus of control

Joel Alcantara^1^, Ryan Whetten^2^

^1^The International Chiropractic Pediatric Association, San Jose, California, USA, ^2^University of Avignon, Avignon, France

*Journal of Patient-Reported Outcomes 2026*, **10(Suppl 1)**:1037

### Aims

While many health-related quality-of-life (HRQoL) instruments have been validated with a variety of methods (i.e., reliability, factor analysis, responsiveness), little work has been done to analyze the semantic meaning or wording of many of these instruments. Semantic analysis plays a crucial role in contributing to the validity of a HRQoL instrument. The multidimensional health locus of control (MHLOC) is a psychological construct that assesses an individual’s beliefs about control over their health outcomes. The aim of this study was to perform a semantic analysis of the MHLOC questionnaire using recent methods developed in the field of Natural Language Processing.

### Methods

To analyze the items of the MHLOC questionnaire from a semantic perspective, we used a sentence transformer, a neural network language model trained to produce high quality vector representations (embeddings) of each item, to capture their semantic meanings. Once in vector form, the questionnaire items were analyzed using K-means clustering analysis to compare their semantic clustering to the 4-factor model of internal, chance, doctor and other people locus of control domains.

### Results

The semantic analysis and K means cluster analysis revealed four distinct clusters. The first cluster was composed of items belonging to chance (n=2) and internal (n=4) locus of control (LOC) domains. The second cluster was composed of items from the chance (n=2) LOC domain. The third cluster was composed of items from the chance (n=2) and doctor (n=1) LOC domains, and the fourth cluster was composed of internal (n=1), doctor (n=2) and other people (n=3) LOC domains (see Table 1 and Figure 1). Only one cluster was comprised of items belonging in the same LOC domain. Overall, the semantic clusters were not comprised of items as expected for the MHLOC instrument.

### Conclusion

The semantic meaning of the MHLOC items do not fully align with the underlying constructs/domains (i.e., internal, chance, doctor and other people) being measured. Our findings are insightful and potentially provide another method of analyzing the validity of HRQOL survey instruments.

**Table. 1 (abstract 1037):**
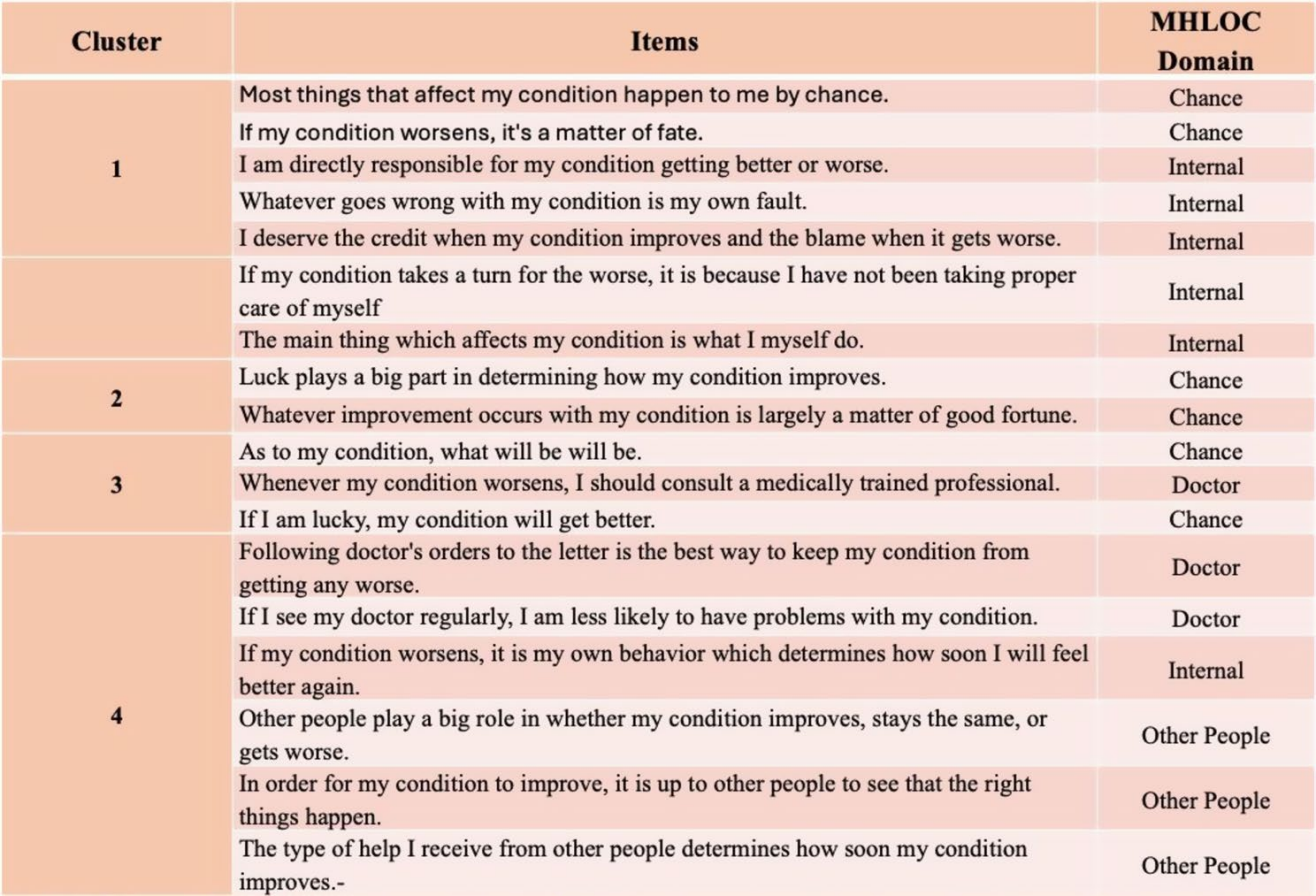
Semantic clusters of items of the MHLOC instrument and their associated locus of control domain

**Fig. 1 (abstract 1037):**
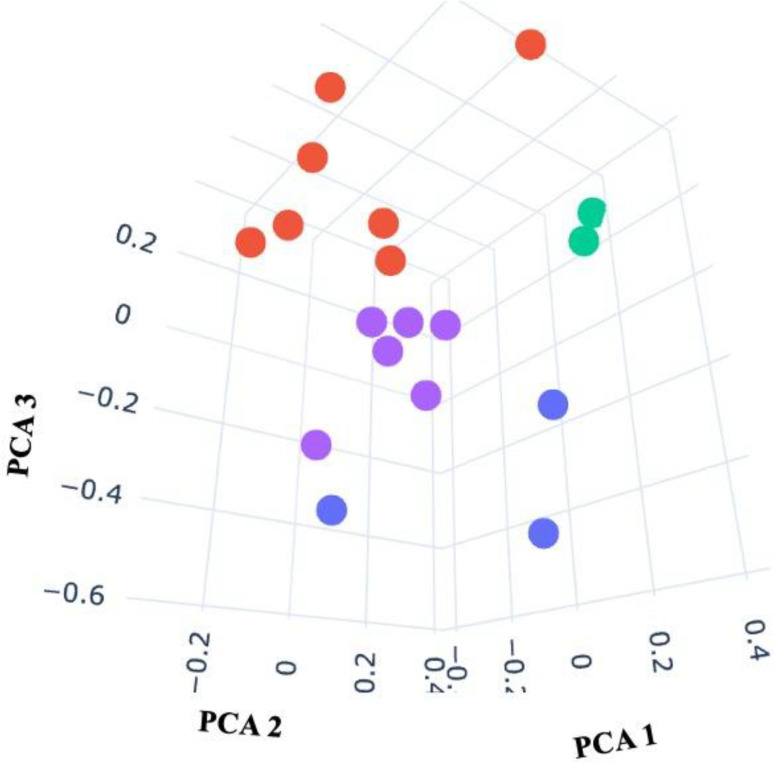
Results of the K means cluster analysis

## 1038 Implementation Strategies for Electronic Patient-Reported Outcomes (ePRO)-Based Cancer Symptom Management in Chinese Primary Care Settings

Changjin Wu^1^, Min Li^2^, Jingyu Zhang^2^, Jundi Zheng^3^, Qiuling Shi^2^, Xiaojun Dai^4^

^1^School of Public Health, Chongqing Medical University, Chongqing, China, ^2^State Key Laboratory of Ultrasound in Medicine and Engineering, College of Biomedical Engineering, Chongqing Medical University, Chongqing, China, ^3^School of Clinical Traditional Chinese Medicine, Yangzhou University, Yangzhou, China, ^4^Department of Oncology, Yangzhou hospital of Traditional Chinese Medicine, Yangzhou, China

*Journal of Patient-Reported Outcomes 2026*, **10(Suppl 1)**:1038

### Aims

Evidence indicates that contextually adapted implementation strategies produce better outcomes than non-tailored approaches. Previous research by our team identified key barriers to the adoption of electronic patient-reported outcomes (ePRO) in Chinese primary oncology care. To address these challenges, this study aims to develop and refine targeted strategies for integrating ePRO systems into routine cancer symptom management.

### Methods

Implementation strategies were selected based on identified barriers using the Consolidated Framework for Implementation Research-Expert Recommendations for Implementing Change (CFIR-ERIC) matching tool. The Level 1 strategies (those recommended by >50% of experts) and the top five cumulative percent ERIC strategies were chosen as the theory-informed implementation strategies in this study. To ensure contextual relevance, focus groups or individual interviews with stakeholders were conducted to adapt these strategies to the practical realities of ePRO adoption in Chinese primary care.

### Results

A total of 13 theory-informed implementation strategies were selected, including eight Level 1 strategies and the top five cumulative percent strategies. Fifteen participants were interviewed, comprising medical oncologists (n = 4), nurses (n = 2), hospital leaders (n = 2), ePRO specialists (n = 3), and research follow-up staff (n = 4). The adapted implementation strategies to ePRO symptom monitoring included engage evidence-based practices (EBP) stakeholders, peer-led EBP sessions, EBP practice remuneration, field-informed expert solutions for ePRO adoption, EBP training for ePRO adoption, develop tiered cancer symptom management, access new funding, expert-led EBP consultation, establish tiered clinical partnerships, develop EBP toolkits, audits with performance feedback, EBP-based continuing medical education, paper/web-based EBP dissemination.

### Conclusion

This study developed 13 tailored implementation strategies to facilitate the integration of ePRO-based cancer symptom management in Chinese primary care settings. Our future work will focus on expanding these strategies to a broader range of stakeholders to elicit their preferences. Strategies that are strongly endorsed will be prioritized during the co-design and implementation phases to enhance stakeholder engagement.

## 1039 Development of a tree-based graded response model R package to test for differential item functioning in patient-reported outcome measures

Olayinka Arimoro^1^, Matthew James^1^, Maria Santana^1^, Lisa Lix^2^, Tolulope Sajobi^1^

^1^University of Calgary, Calgary, Alberta, Canada, ^2^University of Manitoba, Winnipeg, Manitoba, Canada

*Journal of Patient-Reported Outcomes 2026*, **10(Suppl 1)**:1039

### Aims

Tree-based item response theory (IRT) models are a class of novel methods for testing differential item functioning (DIF) in patient-reported outcome measures (PROMs). Existing tree-based IRT models have been developed for binary and polytomous items within the Rasch models framework, which assume equal discrimination (i.e., the degree to which an item distinguishes between individuals with different levels of the latent construct) across items. This assumption may not be tenable in many PROMs data as items may not discriminate equally across populations. To address this gap, we developed an open-source R package for implementing a tree-based (i.e., recursive partitioning) graded response model (GRMTree) to identify a parsimonious set of variables associated with DIF in potentially heterogeneous populations.

### Methods

Major functions of the R package include (1) grmtree, which fits a single GRMTree for DIF detection, (2) grmforest, which implements a forest of GRMTrees on bootstrap samples or subsamples of the data to improve model robustness, (3) varimp, which computes permutation-based variable importance measures for bootstrap samples or subsamples and ranks the covariates, (4) plot, which visualizes the sample partitions across items and subgroups. Other functions extract item parameters and estimate factor scores.

### Results

The package successfully implements core DIF functions using tree-based models, including three specialized plotting systems: histograms of factor scores with normal curves, region plots displaying threshold parameters, and profile plots comparing item parameters across subgroups. The package is currently undergoing validation before submission to the Comprehensive R Archive Network.

### Conclusion

The GRMTree package fills a critical gap in DIF detection by extending the recursive partitioning model to the more flexible GRM framework. The package is developed as an open-source tool for analysts and applied researchers to test for DIF in PROMs data.

**Fig. 1 (abstract 1039):**
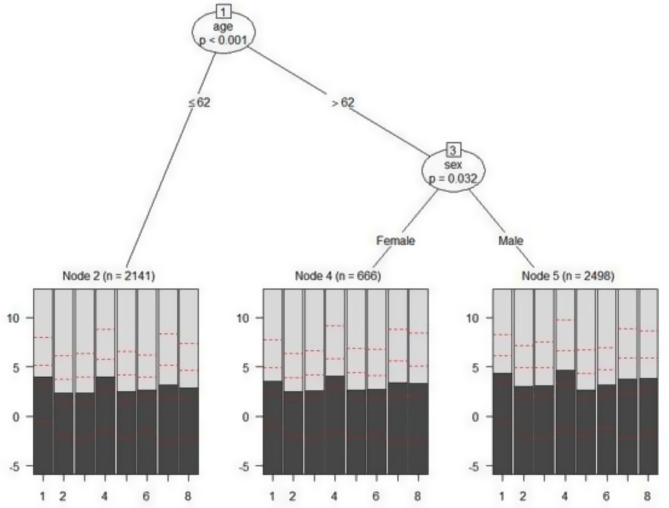
(Region) plot of GRMTree of MOS-SS emotional domain at baseline (2 weeks)

**Fig. 2 (abstract 1039):**
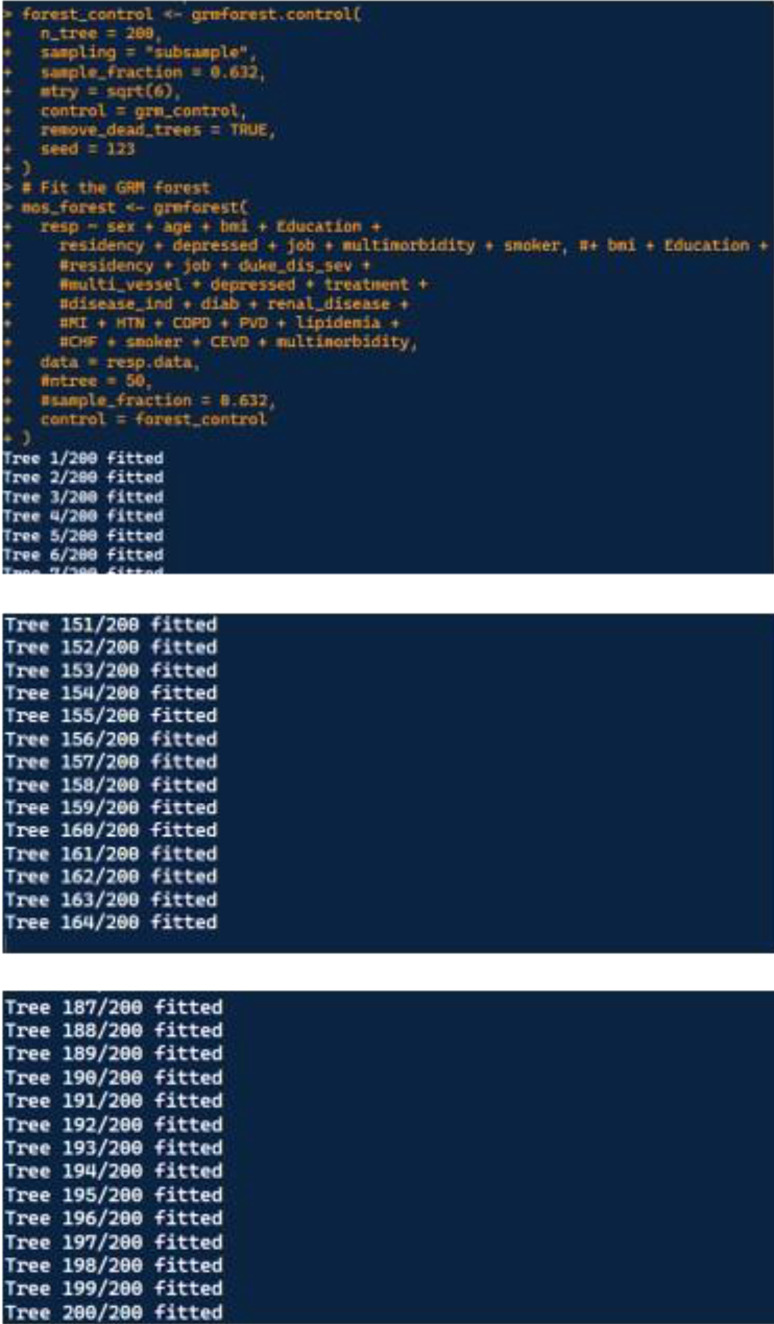
A Sample code output of the GRMForest with 200 GRMTrees

**Fig. 3 (abstract 1039):**
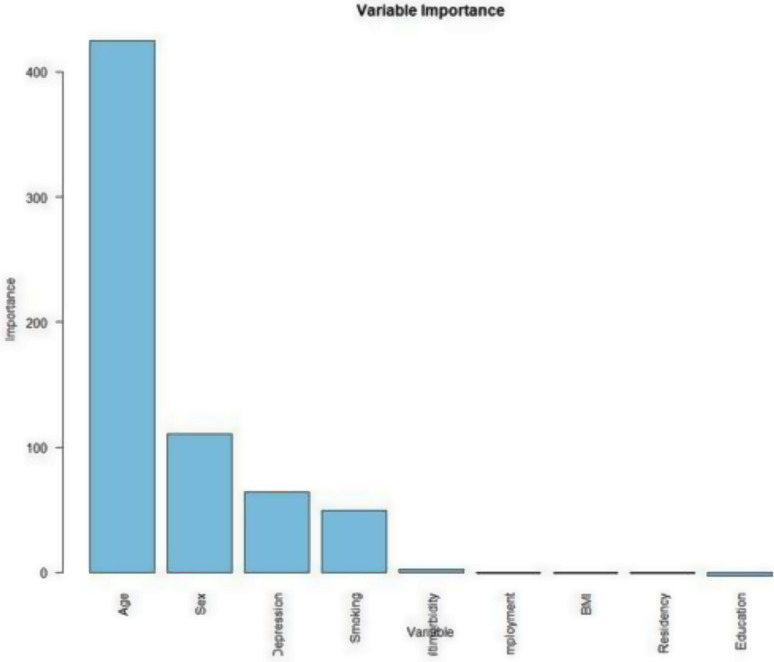
Variable importance of the GRMForest with 200 GRMTrees

## 1040 Contribution of patient-reported versus clinician-reported outcomes to core domain sets and differences in expert and patient voting patterns across these domains

Zachary Hopkins^1^, Dallin Ringwood^2^, Meg Takara^2^, Zachary Frost^3^, Mark Conley^4^, Aaron Secrest^5^, Jacob Kean^5^

^1^University of Utah Health, Salt Lake City, Utah, USA, ^2^University of Utah School of Medicine, Salt Lake City, Utah, USA, ^3^Noordt College of Osteopathic Medicine, Provo, Utah, USA, ^4^University of North Texas Health Sciences Center, Fort Worth, Texas, USA, ^5^University of Utah, Salt Lake City, Utah, USA

*Journal of Patient-Reported Outcomes 2026*, **10(Suppl 1)**:1040

### Aims

Core outcome domain sets define and help to establish critical concepts for clinical research and/or practice and include patient-reported outcome (PRO)- and clinician-reported outcome (CRO)-based concepts. Stakeholder voting patterns for inclusion in core domain sets inform perceptions of domain value and how value varies between stakeholder types.

### Methods

We reviewed dermatology-related core domain development manuscripts from the Core Outcome Measures in Effectiveness Trials-initiative database. Extracted data included stakeholder information, domain theme (i.e. quality-of-life [QoL], clinical signs, etc), and voting results. Domain types and categories were summarized descriptively and differences in voting between patient and expert shareholders for each domain theme were assessed using independent t-tests.

### Results

We identified 20 qualifying studies from 2011-2024. Most core domain sets were for clinical trials (17/20) multiple inflammatory and oncologic diseases. The median number of proposed PROs meeting patient voting approval threshold was 4 (2-7) and 4 (2-6) for CROs. The median number of CRO domains meeting patient criteria was 8 (3-12) for patients and 5 (3-8.5) for experts. Median voting for approved PROs and CROs (percent voting for inclusion) was similar between patients and experts (PROs: 83.0 [78.5-88.0] versus 85.0 [76.0-88.4] and CROs: 81.3 [78.2-86.0] versus 86.3 [81.0-90.7] respectively). Commonly endorsed domains were clinical signs (100% of studies), QoL (94.7%), symptoms (73.7%), and adverse events (68.4%).). Differences between patient and expert voting within each domain theme were not significant (p>0.05). Across individual domains within each study, large voting discrepancies (≥15%) did occur in 98/238 votes, with patients more often voting higher than experts (67/98).

### Conclusion

PROs make up approximately half of final core assessment domains and frequently included QoL, symptoms, and adverse events. High-quality instruments that can practically assesses these domains across the varied skin disease seen in clinic are needed. –Generally, voting for PRO- and CRO-based domains were similar across patients and experts, highlighting a largely unified valuation of domain importance. However, larger voting discrepancies occurred between patients and experts at the individual study level which may inform disease-specific differences in assessment value.

## 1041 Derivation of a preference-based value set for the Short Warwick-Edinburgh Mental Well-being Scale (SWEMWBS) to allow calculation of Mental Well-being Adjusted Life Years (MWALYs)

Hei Hang Edmund Yiu^1^, Stavros Petrou^2^, Sarah Stewart-Brown^3^, David Churchman^4^, Jason Madan^3^

^1^Department of Pharmacology and Pharmacy LKS Faculty of Medicine, The University of Hong Kong, Hong Kong, Hong Kong, ^2^Nuffield Department of Primary Care Health Sciences, University of Oxford, Oxford, UK, ^3^Warwick Medical School, University of Warwick, Warwick, UK, ^4^InSpired Health Outcomes, Witne, UK

*Journal of Patient-Reported Outcomes 2026*, **10(Suppl 1)**:1041

### Aims

Concerns have been raised about the sensitivity of widely used preference-based instruments, such as the EQ-5D, in valuing mental health benefits. There is a growing need for alternative outcome measures beyond QALYs due to increased interest in promoting mental well-being. This project aims to develop U.K. preference-based value sets for the Short Warwick-Edinburgh Mental Wellbeing Scale (SWEMWBS) to enable the estimation of Mental Well-being Adjusted Life Years (MWALYs).

### Methods

As this is the first attempt at valuing SWEMWBS states, a series of stages were implemented to ensure the robustness of the derived valuation sets. Initially, alternative valuation techniques were analysed to justify the appropriate strategy for mental well-being states. A sample of manageable mental well-being states for valuation was identified using various experimental designs. A qualitative piloting study employing a think-aloud interview technique was conducted to explore the cognitive processes involved in completing the valuation tasks. The modified valuation protocol, informed by the qualitative study, was subsequently validated in a larger quantitative study. The responses from this quantitative study were modelled to produce utility values for all 78,125 mental well-being states.

### Results

Both the qualitative and quantitative studies indicated the feasibility, practicality, and face validity of the SWEMWBS valuation. A total of 225 participants in the quantitative study each provided valuation responses to 10 composite time trade-off (C-TTO) and 10 discrete choice experiment (DCE) tasks completed in the EQ-PVT platform, allowing for the estimation of value sets based on heteroskedastic Tobit model, conditional logit model, and inverse variance weighting (IVW) hybrid model. The first two models revealed significant differences, with the hybrid approach offering a desirable blend of insights.

### Conclusion

This project has produced the first preference-based value set for a generic measure of mental well-being. The value set, derived from our hybrid approach, offers a valuable resource for cost-utility analyses of mental well-being interventions. It enables a more comprehensive evaluation of the benefits associated with these interventions, facilitating better-informed decisions in healthcare policy and practice.

## 1042 Cross-Cultural Adaptation and Psychometric Validation of the Danish and Norwegian Versions of the Steroid Symptom Questionnaire for Multiple Myeloma (SSQ-MM)

Tine Rosenberg^1^, Claudia Rutherford^2^, Tracy King^3^, Margaret-Ann Tait^2^, Frida Bugge Askeland^4^, Lene Kongsgaard Nielsen^1^

^1^Quality of Life Research Center, Department of Hematology, Odense University Hospital, Odense, Denmark, ^2^Cancer Care Research Unit, Faculty of Medicine and Health, The University of Sydney, Sydney, NSW, Australia, ^3^Clinical Research Fellow, Cancer Care Research Unit, Faculty of Medicine and Health, The University of Sydney, Sydney, NSW, Australia, ^4^Institute of Clinical Medicine, University of Oslo, Oslo, Norway

*Journal of Patient-Reported Outcomes 2026*, **10(Suppl 1)**:1042

### Aims

To translate and culturally adapt the Australian developed Steroid Symptom Questionnaire for Multiple Myeloma (SSQ-MM) into Danish and Norwegian and to evaluate the psychometric properties of the Norwegian version in newly diagnosed patients with MM.

### Methods

Following international guidelines for translation and cross-cultural adaptation of Patient Reported Outcome (PRO) measures, the SSQ-MM was independently forward- and back-translated by bilingual experts. Cognitive debriefing interviews with patients (n=5 per country) and clinicians (n=2 per country) were used to assess clarity, relevance, and cultural appropriateness of the items. The original developers were involved throughout the process and reviewed feedback from patients and clinicians, supporting content validity and agreement on item inclusion. Psychometric testing was performed using data collected as part of an investigator-initiated study of patients with multiple myeloma receiving steroid treatment (n=43). For the analyses, a confirmatory analytical approach was applied aiming to demonstrate comparable psychometric properties of the Norwegian and Australian measures. Internal consistency (Cronbach’s α), construct validity (correlations with EORTC QLQ-C30), and item-total correlations were evaluated.

### Results

The Danish and Norwegian versions of the SSQ-MM were well accepted and required only minor linguistic adaptations. Internal consistency reliability was acceptable for the SSQ-MM total score for use at a group level (Cronbach’s alpha >0.70). Construct validity was supported by strong correlations between sleep disturbance on the SSQ-MM and insomnia on the EORTC QLQ-C30 (r=0.852) and between the SSQ-MM psychological score and the EORTC QLQ-C30 emotional function (r=0.761). Item-total correlations were acceptable for key sub-scale items; however, results were inconsistent due to small participant numbers (r > 0.5).

### Conclusion

The Norwegian and Danish versions of the SSQ-MM were successfully adapted with evidence of acceptable reliability and strong construct validity. The findings support the preliminary use of the Norwegian SSQ-MM for assessing steroid-related symptom burden in patients with multiple myeloma. However, further validation in larger and more diverse patient populations is recommended to strengthen generalizability.

## 1043 Patient-Reported Outcome Measures for Addiction Treatment: Development of a Digital Health Platform using a Patient-Centered Approach

Juan-Ignacio Mestre-Pintó^1^, Maria Pellicer^1^, Esperanza Vergara^2^, Daniel Folch^1^, Laia Miquel^3^, Carlos Spuch^4^, Adolfo Piñón^5^, Julián Mateus^6^, Jesús Ruiz^6^, Gustavo Gil-Berrozpe^7^, Francina Fonseca^1^, Elena García de Jalón^7^, Fernando Rodríguez de Fonseca^8^, Claudio Tamarit^7^, Francisco Otero^5^, Marta Torrens^1^, Yolanda Pardo^9^

^1^Hospital del Mar Research Institute, Barcelona, Spain, ^2^Universidad de Cádiz, Cádiz, Spain, ^3^Hospital Clínic, Barcelona, Spain, ^4^Fundación Pública Galega de Investigación Biomédica Galicia Sur, Vigo, Spain, ^5^Unidad Asistencial de Drogodependencias CEDRO, Vigo, Spain, ^6^Fundación Hospitalarias, Barcelona, Spain, ^7^Instituto de Investigación Sanitaria de Navarra, Pamplona, Spain, ^8^IBIMA Investigación Biomédica de Málaga, Málaga, Spain, ^9^Universitat Autònoma de Barcelona, Barcelona, Spain

*Journal of Patient-Reported Outcomes 2026*, **10(Suppl 1)**:1043

### Aims

Substance Use Disorders (SUDs) are highly prevalent and impact individuals’ health, well-being, and social functioning. The management of SUD patient can be improved through the integration of Patient-Reported Outcome Measures (PROMs) in clinical practice. This study aimed to co-create with patients and professionals a Digital Health Platform (ADDPROMs) for the integration of PROMs in outpatient addiction treatment services.

### Methods

The ICHOM Addiction standard set was implemented in REDCap. Patient-Reported data is gathered through a link sent to patients by email. Two differents versions were designed in Tableau for giving feedback of the results to patients and health professionals. The first version of the ADDPROMs has been reviewed and updated according to the results of the System Usability Scale administered in a pilot study; and its final version agreed upon consensus reached after focus groups with patients and healthcare professionals.

### Results

Preliminary results support the design and content of the ADDPROMs, while suggesting several changes from healthcare professionals’ perspective: simplify PROMs prescription, add training/informative section in the platform, and facilitate interpretation strategies with anchors or reference values. SUD patients reported the ADDPROMs to be easy to use, even had some concerns about the usefulness of their answers. Suggested to remark the fact that clinicians will have patients answers available before their visit. SUD patients, have also ask for the integration of PREM items in the survey and more specific questions regarding their health status.

### Conclusion

The final version of the ADDPROMs has been co-created thanks to the feedback of both, SUD patients and health professionals, to facilitate the prescription, administration, visualization, and interpretation of PROMs data into routine clinical management. Implementing the ADDPROMs in addiction care could facilitate outcome comparisons, guide service improvement, and ultimately increase the effectiveness and quality of interventions, contributing to a stronger and more sustained recovery for patients undergoing treatment.

## 1044 Validation of the Treatment-Induced Neuropathy Assessment Scale (TNAS) in Chinese Cancer Patients: a prospective cohort study

Chenxi He^1^, Wei Xu^1^, Feng Gao^2^, Wenlin Wu^1^, Shi Wang^1^, Qiuling Shi^1^, Fan Li^1^

^1^Chongqing Medical University, Chongqing, China, ^2^Mianyang Central Hospital, Mianyang, China

*Journal of Patient-Reported Outcomes 2026*, **10(Suppl 1)**:1044

### Aims

Treatment-induced peripheral neuropathy(TIPN) is a critical adverse effect of Anti-tumor treatment. Chemotherapy-induced peripheral neuropathy (CIPN) is the most widely recognized form of treatment-induced peripheral neuropathy (TIPN). In China, the prevalence of CIPN was 50%~90%, with 42–84% of survivors still experiencing CIPN symptoms two years post-treatment. The Treatment-Induced Neuropathy Assessment Scale (TNAS) is a valid instrument for assessing the severity and course of neuropathy across various cancer treatments. However, the psychometric properties of the Chinese version of TNAS have not been established yet.

### Methods

A prospective cohort study was conducted in two hospitals, primarily enrolling patients who were treated with cisplatin, oxaliplatin, or taxane-platinum. The research obtained the Chinese version of TNAS from MD Anderson Cancer Center. The scales were administered to patients at baseline, on the day of chemotherapy, and at 3, 5, 7, 10 and 14 days after each treatment, across up to 12 follow-up cycles. The content, construct, criterion and discriminant validity of the TNAS were examined. The reliability of the instrument was tested by examining the internal consistency and test–retest reliability.

### Results

214 cancer patients with breast, colorectal, or biliary tract cancers participated in this cohort study. The top three most severe symptoms were numbness、disturbed sleep and coldness(1.27±1.81, 1.22 ± 1.89, 0.49 ± 1.23, respectively). Factor analysis identified 2 underlying constructs, sensory and motor subscale, which had Cronbach alpha coefficients of 0.84 and 0.88, respectively. The 7-day test–retest reliability(n=107) ranged from 0.51 to 0.75. Cognitive debriefing showed that 93.2% of participants found the scale easy to understand. Known-group validity (sensitivity) was supported by the ability of the TNAS to detect significant differences in sensory and motor subscales according to oxaliplatin and non-oxaliplatin (1.6±5.61, effect size=0.29, 1.18±2.66, effect size=0.44, respectively; P <0.05). The correlation coefficients for TNAS indicating good validity.The sensory and motor subscales were more severe at follow-up, demonstrating the instrument’s sensitivity to accumulating dose(8.86±7.5, effect size=1.36,3.78±4.66, effect size=0.94, respectively; P <0.001).

### Conclusion

The Chinese version of TNAS is a brief, valid, and feasible measure of TIPN for use with Chinese cancer patient with clinical utility in personalized monitoring.

**Table 3 (abstract 1044):**
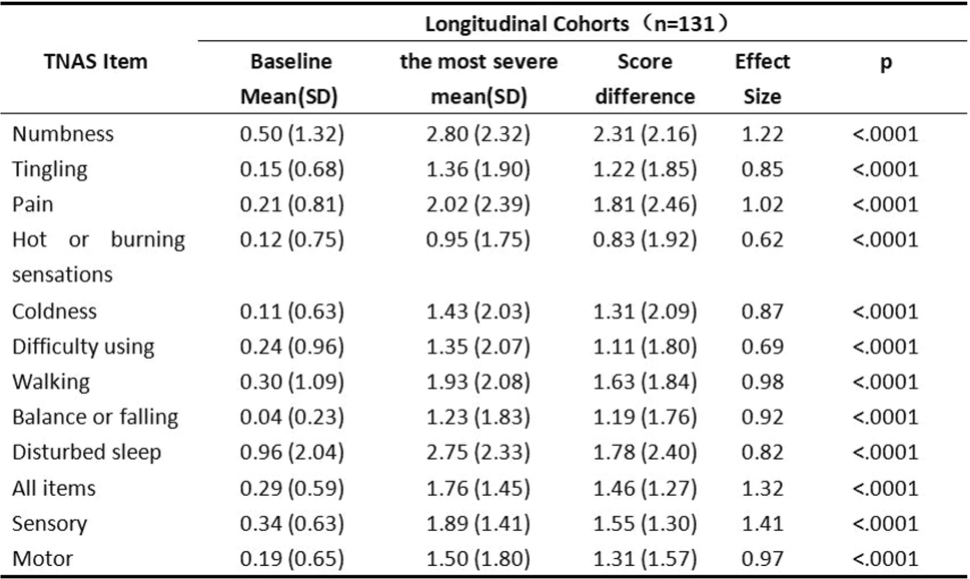
Sensitivity of the TNAS: Change in TNAS Items from Start of Treatment to the most severe symptoms (Longitudinal Cohorts)

**Table 2 (abstract 1044):**
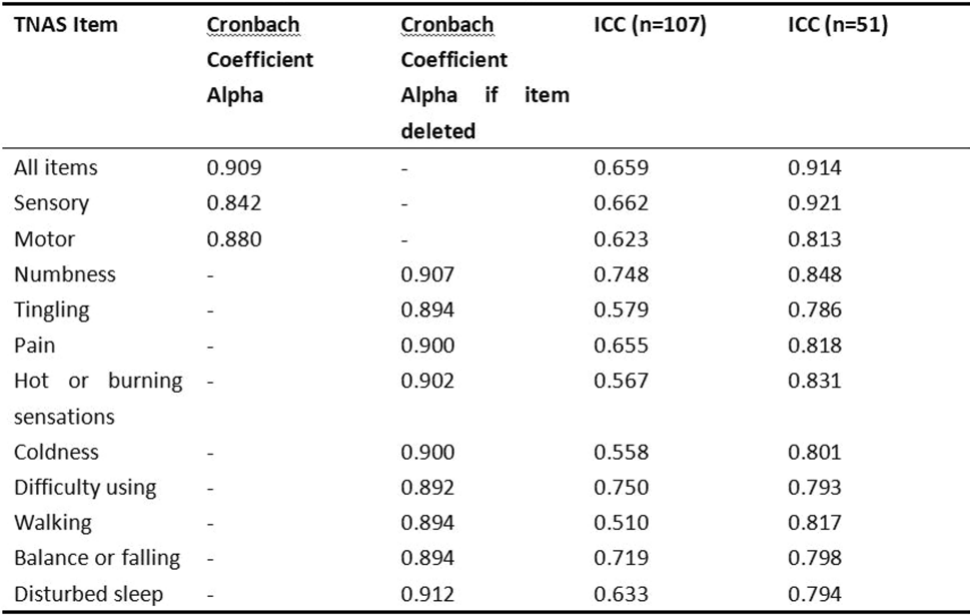
Internal consistency and test-retest reliability of the TNAS

**Table 1 (abstract 1044):**
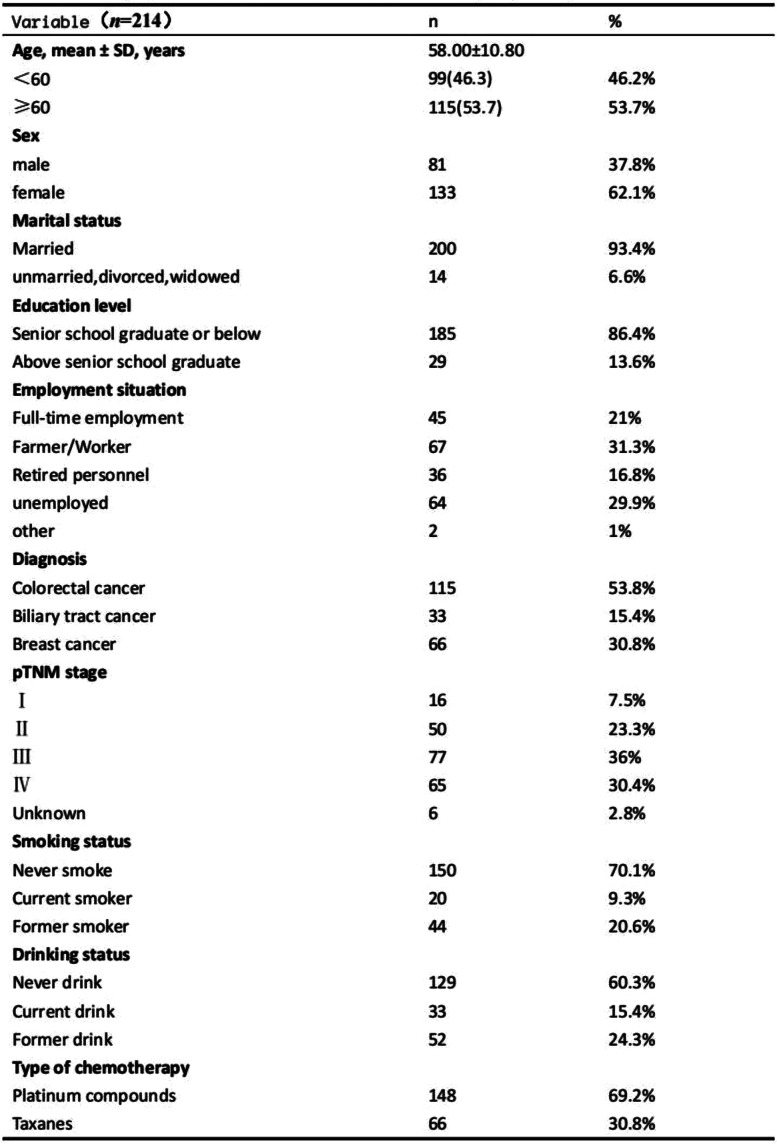
Demographic and Clinical Characteristics of the Samples (N = 214)

## 1046 Needs and health-related quality of life domains relevant to people in Europe undergoing cancer treatment: A systematic review of qualitative research

Clara Amat-Fernandez^1^, Yolanda Pardo^2^, Óscar Périz-Colón^1^, Pablo Notti Saint-Jean^1^, Leslye Rojas-Concha^3^, Melissa Thong^4^, Giovanni Apolone^5^, Cinzia Brunelli^5^, Augusto Caraceni^6^, Norbert Couespel^7^, Nanne Bos^8^, Mogens Groenvold^3^, Stein Kaasa^9^, Gennaro Ciliberto^10^, Claudio Lombardo^11^, Ricardo Pietrobon^12^, Gabriella Pravettoni^13^, Aude Sirven^14^, Hugo Vachon^15^, Catalina Lizano-Barrantes^16^, Olatz Garin^1^

^1^Hospital del Mar Research Institute, Barcelona, Spain, ^2^Universitat Autònoma de Barcelona, Barcelona, Spain, ^3^University of Copenhagen, Copenhagen, Denmark, ^4^German Cancer Research Center, Heidelberg, Germany, ^5^Fondazione IRCCS Istituto Nazionale Dei Tumori, Milan, Italy, ^6^Università Degli Studi Di Milano, Milan, Italy, ^7^European Cancer Organisation, Brussels, Belgium, ^8^Netherlands Institute for Health Services Research, Utrecht, Netherlands, ^9^Oslo Universitetssykehus HF, Oslo, Norway, ^10^Digital Institute for Cancer Outcomes Research, Brussels, Belgium, ^11^Organisation of European Cancer Institutes, Brussels, Belgium, ^12^SporeData OÜ, Tallinn, Estonia, ^13^Istituto Europeo Di Oncologia IRCCS, Milan, Italy, ^14^Unicancer, Paris, France, ^15^European Organisation for Research and Treatment of Cancer, Brussels, Belgium, ^16^Universidad de Costa Rica, San Jose, Costa Rica

*Journal of Patient-Reported Outcomes 2026*, **10(Suppl 1)**:1046

### Aims

The EU-funded project “Quality of Life in Oncology: measuring what matters to cancer patients and survivors in Europe” aims to co-design, validate and disseminate the EUonQoL-Kit, an electronic patient-reported outcome measure (PROM) with versions for individuals in active cancer treatment, survivors, and patients in need of palliative care. The aim of this study is to systematically review qualitative studies on outcomes, needs, experiences, preferences, concerns and health-related quality of life (HRQoL) of people in Europe undergoing active cancer treatment published over the last decade to contribute to the content of the EUonQoL-Kit.

### Methods

Protocol CRD42024575065 in http://www.crd.york.ac.uk/PROSPERO. The search was conducted in PubMed and Scopus since 2013. Inclusion criteria: studies with qualitative methods, constructs related to HRQoL, and adults in active cancer treatment in Europe. Abstracts and full text were revised, data extracted and study risk of bias assessed independently by two researchers. The primary outcomes were the themes arising from each study. A thematic analysis stratified according by study objective was undertaken by grouping themes (primary outcome) into categories.

### Results

Of 18,256 articles identified, 36 fulfilled the inclusion criteria: 21 studies with a generic objective on outcomes, concerns and HRQoL of individuals in active cancer treatment, and 15 focused specifically on: symptoms, health-care management, psychological factors, pain and work. Five categories (84 themes) emerged from the studies with generic objectives: ‘Psychological Function’ (n=36), ‘Clinical Management’ (n=24), ‘Symptoms and Physical Function’ (n=15), ‘Social Function’ (n=13), and ‘Life Disruption’ (n=10). Among studies with specific objectives, it stands out that 11 of them focused on health-care management, and most of its themes fitted into the same 5 categories.

### Conclusion

Results clearly showed the predominance of the social and psychological function domains over physical domains among people in active cancer treatment. Clinical management, generally understood as part of the patient’s experience instead of the content of a PROM, emerged as a prominent need with identified categories including: patient involvement in care, interaction with healthcare professionals, and unmet needs of patients. Therefore, these aspects should be incorporated into the evaluation of patient-centered initiatives for adults in active cancer treatment in Europe.

## 1047 Mapping the Edmonton Symptom Assessment System-revised (ESAS-r) to the EQ-5D-5L in Patients with Cancer

Hilary Short^1^, Jiabi Wen^1^, Fatima Al Sayah^1^, Siwei Qi^2^, Claire Link^2^, Linda Watson^2^, Andrea Deiure^2^, Jeff Johnson^1^, Lisa Barbera^3^

^1^University of Alberta, Edmonton, Alberta, Canada, ^2^Cancer Care Alberta - Alberta Health Services, Calgary, Alberta, Canada, ^3^University of Calgary, Calgary, Alberta, Canada

*Journal of Patient-Reported Outcomes 2026*, **10(Suppl 1)**:1047

### Aims

The Edmonton Symptom Assessment System-Revised (ESAS-r) is a widely used patient-reported outcome measure that captures symptoms commonly experienced by patients with cancer. However, it does not generate preference-based health utility scores. This study aimed to develop a mapping algorithm to predict the Canadian preference-based EQ-5D-5L index scores from the ESAS-r in cancer populations.

### Methods

Cross-sectional data collected between January 2018 and December 2021 from cancer clinics across Alberta, Canada, were utilized. Spearman correlation coefficients were computed between instruments. Response mapping was conducted using ordered logistic regression to predict response levels for each of the EQ-5D-5L dimensions. Various direct mapping model types were fitted. A 5-fold cross-validation method was used. Four model specifications were explored for each mapping type: 1) all ESAS-r items; 2) all ESAS-r items + age + sex + cancer stage; 3) select ESAS-r items; 4) select ESAS-r items + age + sex + cancer stage. Purposeful selection procedures were performed in models 3 and 4 to select significant ESAS-r variables. Model performance was assessed using mean square error (MSE) and mean absolute error (MAE).

### Results

Preliminary results focus on the breast cancer group (n=318). Mean age was 60.1 years (SD 14.3), and 33.7% had stage IV disease. The mean EQ-5D-5L index score was 0.660 (0.250 SD), with no significant variation by stage. The most strongly correlated ESAS-r items with the EQ-5D-5L index were pain (r: -0.57), wellbeing (r: -0.54), and tiredness (r: -0.52). Across all model specifications (MAE: 0.136 - 0.157, MSE: 0.0330 - 0.0444), the model including all ESAS-r items tended to perform best. Among mapping models, Tobit models (MAE: 0.136 - 0.138, MSE: 0.035 - 0.036) and ordered logistic regression models (MAE: 0.137-0.139 MAE, MSE: 0.0330 – 0.334) provided the highest predictive accuracy.

### Conclusion

We developed a robust mapping algorithm to estimate EQ-5D-5L index scores from ESAS-r data. This will support health economic evaluations in oncology settings where EQ-5D-5L data are unavailable.

## 1048 Impact of fruits and vegetable intake on HRQoL: Results from a randomized trial comparing produce prescription delivery modes

Corneliu Bolbocean^1^, Lacey McCormack^2^, Christine Hockett^3^

^1^University of Oxford, Oxford, Arkansas, UK, ^2^Avera Research Institute, Sioux Falls, South Dakota, USA, ^3^University of South Dakota, Sioux Falls, South Dakota, USA

*Journal of Patient-Reported Outcomes 2026*, **10(Suppl 1)**:1048

### Aims

Suboptimal fruit and vegetable consumption is strongly associated with chronic disease risk. This study evaluated the impact of a produce prescription program—comparing traditional in-store vouchers with home-delivered produce boxes—on health-related quality of life (HRQoL) among low-income adults attending a clinic-based setting in an urban area of South Dakota, USA over a 6-month period.

### Methods

Adults from participating clinics eligible for SNAP, WIC, or Medicaid/CHIP were randomized (1:1) to receive either $25/week in vouchers for fresh fruit or vegetable usable at partner grocery stores/farmers markets (in-store group) or a curated produce box of equivalent value delivered weekly (produce box group) for up to 24 weeks. Data on socio-economic variables, self-rated general health, and EQ-5D-5L were collected at baseline, and six months. Parametric and non-parametric tests along with generalized linear regression models were used to evaluate differences between groups and over time.

### Results

A total of 224 participants enrolled at baseline, of whom 171 completed the 6-month follow-up visit. There were no significant differences in the EQ-5D-5L scores between delivery modes (in-store vs. produce box), however, overall significant improvements in self-rated general health were observed: the proportion reporting “good or better” health rose from 38.3% at baseline to 59.8% at six months (p<0.001). Mean EQ-5D-5L visual analogue scale scores trended upward: 66.8 at 6 months vs. 60.8 at baseline, p=0.12; similar trend observed for median EQ-5D-5L visual analogue scale scores trended upward: 70.0 vs 63.5, p=0.25.

### Conclusion

Preliminary results indicate that the mode of delivery does not differently impact health status, but overall fresh produce prescriptions may improve self-reported health status within six months among individuals facing food insecurity and chronic disease risk.

## 1049 Barriers and Facilitators to Implementation of Breast Cancer Digital Care Companion App in Community Oncology Settings

Amanda Higgins^1^, Manraj Kaur^1^, Maria Edelen^1^, Patricia Dykes^1^, Kelly Aschbrenner^2^, Meghan Garstka^3^, Andrea Pusic^1^

^1^Brigham and Woman’s Hospital, Boston, Massachusetts, USA, ^2^Dartmouth College, Hanover, New Hampshire, USA, ^3^University of Maryland Medical Center, Baltimore, Maryland, USA

*Journal of Patient-Reported Outcomes 2026*, **10(Suppl 1)**:1049

### Aims

Digital health interventions, such as patient-facing care companion apps, can enhance patient engagement and improve quality of life (QoL) in oncology care. However, integrating such technology into community cancer centers presents unique challenges and opportunities. This study explores potential barriers and facilitators to implementing a care companion app within clinical workflows in lower-resourced settings to support cancer patients.

### Methods

A qualitative, semi-structured interview study was conducted with 16 patients undergoing breast cancer-related surgery and 33 healthcare providers at five community cancer centers. Patients were recruited through clinician referrals and clinic posters based on inclusion criteria of a breast cancer diagnosis and seeking care in lower resourced settings. Virtual interviews were conducted using open-ended questions after a demo or real-time exposure to imPROVE, a breast cancer care companion web-based app, and focused on usability, perceived benefits, and implementation barriers and facilitators. Interviews were audio-recorded, transcribed verbatim, and analyzed thematically.

### Results

Patient interviews and clinical staff focus groups revealed key facilitators and barriers to implementing imPROVE. Patients emphasized that provider engagement and a focus on resources encouraged participation. They found the app user-friendly and easy to understand. Barriers included the burden of using an external app during a new diagnosis, e-literacy challenges, and privacy concerns—preferring to discuss sensitive topics with providers. Clinical staff noted that enthusiasm from clinicians and site champions, along with a dedicated staff member, would support implementation. App resources and EHR integration were also seen as facilitators. Barriers included limited IT capacity, patient e-literacy, time constraints in clinic, and staff turnover.

### Conclusion

This study highlights both the promise and complexity of integrating a digital care companion app into community oncology settings. While patients and providers identified clear benefits—such as improved engagement, user-friendly design, and resource support—successful implementation depends on strong provider involvement, dedicated staff, and seamless workflow integration. Addressing barriers like IT limitations, patient e-literacy, and privacy concerns will be critical for equitable, sustainable adoption. These findings offer insights for embedding digital health tools into routine cancer care, particularly in underserved populations.

## 1050 Optimizing Color Schemes for Graphs: Enhancing Accessibility and Inclusivity

Matthew O’Brien^1^, Katie Frampton^2^, Kim Cocks^2^

^1^Adelphi Values, Boston, Massachusetts, USA, ^2^Adelphi Values, Bollington, UK

*Journal of Patient-Reported Outcomes 2026*, **10(Suppl 1)**:1050

### Aims

Color in figures of patient-reported outcomes can be a useful tool in improving the ease of interpretation of results by highlighting trends between responder groups, clearly differentiating between severity of outcomes, and conveying intercurrent events (e.g. missingness, death) with minimal use of text. Commonly used color schemes, such as red for poor outcomes and green for good, may not be widely accessible for people with color vision deficiency (CVD). The aim is to develop color schemes and guidelines that are accessible while maintaining aesthetic appeal across a variety of figures and study designs.

### Methods

The three different types of CVD in humans are Protanopia, Deuteranopia, and Tritanopia. An online tool that simulates CVD that uses mathematics and confusion line theory was used to select visually distinct colors across each type. Color theory concepts of divergent, triadic, and analogous schemes were applied to create a variety of different plots and recommended colors for specific graphs were guided using AI.

### Results

Divergent schemes use two colors that diverge from a central point and can be used to display two groups for direct comparison or to show distribution of category responses. Similarly, the triadic scheme offers comparison between three groups, which can be used for displaying responder groups. Analogous colors are those that share a common hue and convey groupings that are similar. These can be used for one group or for multiple groups. A multicolor scheme shows maximum contrast between 7 different groups. Missing is shown as grey in all non-greyscale schemes. Figure 1 illustrates suggested color schemes that are accessible to individuals with CVD.

### Conclusion

AI advises the use of blue and orange, yellow and blue, and purple and green as the most accessible color schemes. Grey is also an accessible color that can be used in PRO figures and can be implemented into any scheme to visualize specific outcomes such as missing data. Typical visualizations of PRO data will demonstrate these color schemes and an online poll will be used to collect preferences from key stakeholders at the ISOQOL conference, e.g. patients, clinicians.

**Fig. 1 (abstract 1050):**
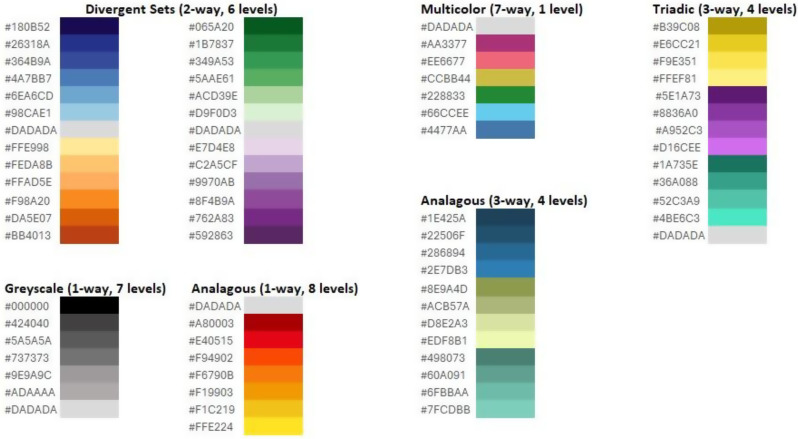
Optimizing Color Schemes for Graphs: Enhancing Accessibility and Inclusivity

## 1052 Adaptation of Goal Attainment Scaling for Prader Willi Syndrome (PWS): Development of a Goal Inventory for Standardized Implementation

Gunes Sevinc^1^, Chere Chapman^2^, Maria Picone^3^, Angeni Cordova^3^, Amee Revana^4^, Ann Sheimann^5^, Deepan Singh^6^, Harold Van Bosse^7^, Terry Jo Bichell^8^, Jessica Duis^9^, Jennifer Miller^10^

^1^Ardea Outcomes, Vancouver, British Columbia, Canada, ^2^Ardea Outcomes, Halifax, Nova Scotia, Canada, ^3^TREND Community, Philadelphia, Pennsylvania, USA, ^4^Texas Children’s Hospital, Baylor College of Medicine, Houston, Texas, USA, ^5^Johns Hopkins Hospital, Baltimore, Maryland, USA, ^6^Maimonides Medical Center, Brooklyn, USA, ^7^SSMHealth, Cardinal Glennon Children’s Hospital, St. Louis University, St.louis, USA, ^8^Combined Brain, Brentwood, USA, ^9^BioMarin Pharmaceutical Inc., Colorado, USA, ^10^University of Florida, Gainesville, USA

*Journal of Patient-Reported Outcomes 2026*, **10(Suppl 1)**:1052

### Aims

Prader-Willi Syndrome (PWS) is a complex neuroendocrine disorder with notable heterogeneity in disease manifestation and symptom progression, resulting in challenges with the assessment of treatment outcomes. Personalized outcome assessment through Goal Attainment Scaling (GAS) can address this challenge, as it quantifies change based on individualized goals. Semi-standardized goal inventories support goal setting by minimizing inconsistencies across GAS interviewers/patients and ensuring the goals are relevant to the treatment. Here, we aimed to develop a goal inventory with input from the literature, clinicians, patients, and/or their caregivers and adapted GAS for PWS.

### Methods

A targeted literature review (n=220) informed semi-structured interview guide. Three focus groups were conducted with clinicians (n=6). Individuals with PWS (n=25) were stratified by age category, sex at birth, PWS subtype, and use of growth hormones. One-on-one interviews were conducted with individuals with PWS and/or their caregivers. All interviews were recorded and transcribed, and the data was thematically analyzed by NVivo 12 to identify the symptoms and challenges of individuals with PWS and their families. The goal inventory was revised and refined after cognitive debriefing interviews (n=5) with individuals with PWS and/or their caregivers and additional expert input (n=2).

### Results

The symptoms and challenges reported by individuals with PWS, their caregivers, and clinicians encompassed various symptoms, challenges, and impacts. These included neuropsychological and behavioral challenges (n=11), emotional regulation difficulties (n=9), food-focused behaviors (n=7), sleep disturbances (n=4), metabolic issues (n=7), endocrine problems (n=9), delayed milestones and development (n=8), musculoskeletal symptoms and challenges (n=9), heart and respiratory issues (n=4), gastrointestinal problems (n=5), and other neurological symptoms such as pain and vision (n=6).

### Conclusion

Clinicians, patients, and families emphasized the diversity of PWS symptoms and manifestations, which highlighted the importance of patient input for developing goal inventories and the variability in symptom manifestations and impacts. Future work is recommended to tailor the goal inventory for any investigational drug and test it for feasibility. The heterogeneity in symptom manifestations corroborates the value of GAS in heterogeneous conditions such as PWS to enhance drug development efforts.

## 1053 Prognostic value of health-related quality of life for major adverse cardiovascular events in myocardial infarction patients: A systematic review of prognostic models

Raghad AL-Kafawin^1^, Tatendashe Dondo^2^, Ben Hurdus^3^, Rawan Abuzinadah^1^, Cris Gale^4^, Theresa Munyombwe^5^

^1^University of leeds, Leeds, UK, ^2^Cardiovascular Epidemiology, University of Leeds, Leeds, UK, ^3^Department of Cardiology, Leeds Teaching Hospitals (NHSTrust), Leeds, UK, ^4^Cardiovascular Medicine, University of Leeds, Leeds, UK, ^5^Biostatistics, University of Leeds, Leeds, UK

*Journal of Patient-Reported Outcomes 2026*, **10(Suppl 1)**:1053

### Aims

To describe the current state of the utility of health-related quality of life (HRQoL) in prediction models for major adverse cardiovascular events (MACE) in myocardial infarction (MI) patients.

### Methods

Five electronic databases (Ovid Medline, Embase, Web of Science, PsycINFO, and PubMed) were systematically searched from inception to December 2024. Eligible studies included those that developed or validated prediction models incorporating HRQoL as a predictor for MACE or its components, such as recurrent MI, stroke, all-cause mortality, heart failure, revascularization, unstable angina, or hospital readmission. Data was extracted using the CHARMS checklist, and only studies reporting at least one model performance metric were included. The risk of bias was evaluated using the prediction model risk of bias assessment tool (PROBAST).

### Results

Ten studies met the inclusion criteria. Most (70%) were model development-only studies, while 30% included both development and external validation; no validation-only studies were identified. Half of the studies focused exclusively on MI populations, while the rest included MI as a subgroup. Most were conducted at a country level, with the USA being the most common setting. Predicted outcomes included various components of MACE, such as all-cause or cardiac mortality, hospital readmission, and recurrent MI, with prediction horizons ranging from 30 days to five years. HRQoL was assessed using both generic and disease-specific patient-reported measures (e.g., EQ-5D, SF-12/36, KCCQ, SAQ, and MacNew), with varying methods of incorporation into models. Discriminative performance was reported in all studies (C-statistics 0.628–0.81), but confidence intervals were often missing. Calibration was reported in half of the studies. Only one study provided full model specifications, limiting reproducibility. Overall, studies had a high risk of bias, primarily due to limitations in the analysis domain.

### Conclusion

This review highlights that the utility of HRQoL as a predictor for MACE remains underdeveloped. The few existing models remain in their early stages, having limited validation and gaps in methodological transparency. Moving forward, there is significant potential for research to advance this field by creating more robust, well-validated models and leveraging standardized HRQoL measures to inform clinical care better.

## 1054 Understanding health-related quality of life and experience of care in individuals with face transplantation

Calvin R. Schuster^1^, Elena Tsangaris^1^, Anne F. Klassen^2^, Bohdan Pomahac^3^, Andrea L. Pusic^1^, Manraj N. Kaur^1^

^1^Brigham and Women’s Hospital, Harvard Medical School, Boston, Massachusetts, USA, ^2^McMaster University, Hamilton, Ontario, Canada, ^3^Yale New Haven Hospital, Yale School of Medicine, New Haven, Connecticut, USA

*Journal of Patient-Reported Outcomes 2026*, **10(Suppl 1)**:1054

### Aims

Since 2005, 50 face transplants have been performed globally. Facial vascularized composite allotransplants (fVCA) are a reconstructive option for patients with severe facial disfigurements to restore facial function and appearance. While data on graft survival has been reported, there is limited data on patient-reported outcome measures (PROMs) following fVCA.

### Methods

Semi-structured interviews were conducted with patients recruited from a single plastic surgery transplantation program. FACE-Q concepts informed the interview guide. Transcripts were coded using line-by-line coding and content analysis. Constant comparison was used to create themes and subthemes.

### Results

Interviews were conducted with the recipients of seven fVCAs (mean age 54.6, range 31-68). A majority were white (85.7%, n=6) and 57.1% were men (n=4). Time between injury and fVCA varied from 2–10 years and follow-up time varied from 3–10 years. After fVCA, 85.7% (n=6) expressed overall satisfaction with their facial appearance. 71.4% (n=5) felt their postoperative face was proportionate, one felt the donor’s face was too large, and one felt the teeth and palate were too large. A majority had an improved ability to eat postoperatively (85.7%, n=6); however, despite notable improvement, 57.1% (n=4) still experienced functional challenges with eating, such as food falling from the mouth. For drinking, 71.4% (n=5) experienced an improvement, but 42.9% (n=3) still experienced functional challenges. Of those who had difficulty breathing preoperatively, 60.0% (n=3) experienced improved breathing and 40.0% (n=2) experienced continued difficulty breathing postoperatively. Of those who discussed financial impact, 33.3% (n=2) experienced financial strain related to the fVCA. Overall, 57.1% (n=4) described decreased distress in social settings, for example, feeling like they were stared at less and were more comfortable meeting new people after fVCA. 85.7% (n=6) described a desire to help other fVCA recipients by sharing their experiences, or a benefit to hearing the experiences of other fVCA recipients. All patients expressed strong overall satisfaction with fVCA. As one recipient noted, “I’m so happy I got a second chance at life.”

### Conclusion

fVCA can impact quality of life for patients with severe facial disfigurements by improving functional and psychosocial outcomes such as the ability to eat, drink, breathe, and exist comfortably in public.

## 1055 Advancing Reliable Measurement in Cognitive Aging and Decision-making Ability (ARMCADA): A pilot study on financial decision-making ability measures

Emily H. Ho^1^, Berivan Ece^1^, Patricia Bucko^1^, Tatiana Karpouzian-Rogers^1^, Elizabeth Dworak^1^, Molly Mather^1^, Miriam Novack^1^, Zahra Hosseinian^1^, Sarah Plia^1^, S. Duke Han^2^, Peter Lichtenberg^3^, Sandra Weintraub^1^, Richard Gershon^1^

^1^Northwestern University, Feinberg School of Medicine, Chicago, Illinois, USA, ^2^University of Southern California, Department of Psychology, Los Angeles, USA, ^3^Wayne State University, Institute of Gerontology, Detroit, USA

*Journal of Patient-Reported Outcomes 2026*, **10(Suppl 1)**:1055

### Aims

The Advancing Reliable Measurement in Cognitive Aging and Decision-making Ability (ARMCADA) initiative aims to develop a multidomain decision-making (DM) battery for adults aged 45 and above. Financial decision-making (FDM) is one of the domains included in the ARMCADA project. Following a scoping review, a set of FDM measures was identified for potential inclusion in the final battery. The goal of the current pilot study is to evaluate the feasibility and reliability of selected FDM measures and compare performance with that of self-report subjective cognitive decline.

### Methods

A pilot sample of 150 adults (minimum 30% male) aged 45 and above will be recruited through an external online recruitment vendor. Participants will complete a demographic questionnaire, depression, anxiety, and social isolation screeners, and the selected FDM measures via REDCap online in a single session. FDM measures will include tasks assessing financial knowledge, financial exploitation vulnerability, numeracy, risk tolerance, and decision-making scenarios. The measures are self-administered and take approximately 25 minutes. Feasibility will be evaluated by the percentage of participants completing all items, and reliability will be examined using Cronbach’s alpha coefficient to assess the internal consistency of each measure.

### Results

We hypothesize that the selected FDM measures will be feasible and reliable for middle-aged and older adults. Specifically, we expect high completion rates (>95%), indicating that the measures are manageable for participants in this age group. Additionally, we anticipate high internal consistency (Cronbach’s alpha coefficients >.80) for all measures, supporting their reliability. We also expect that FDM ability will be inversely related to subjective cognitive decline, though the magnitude of the relationship may vary across the FDM measures.

### Conclusion

Results from this pilot study are expected to support the feasibility and internal consistency of the selected FDM measures, aligning with ARMCADA’s goal of developing a practical and psychometrically sound DM battery for middle-aged and older adults. Findings will inform the refinement and inclusion of FDM measures in the final multidomain ARMCADA battery.

## 1056 Scientific best practices in electronic clinical outcome assessments: a literature review from past to present

Lindsay Hughes^1^, Jowita Marszewska^2^

^1^IQVIA, New York, New York, USA, ^2^IQVIA, Cleveland, Ohio, USA

*Journal of Patient-Reported Outcomes 2026*, **10(Suppl 1)**:1056

### Aims

Clinical Outcome Assessments (COAs) measure how patients feel, function, and survive, and can support label claims by demonstrating impact of treatment on patients’ Quality of Life (QOL). Collecting COAs using technology-enabled solutions (eCOA) improves patient compliance and data quality, which is crucial for reliable research outcomes. The early development of technology in the COA space was grounded in strong scientific principles, including a focus on behavioral science and how people interact with technology. As technology use has increased, the industry focus shifted away from science prioritizing speed and cost and the intention of eCOA has diminished. Nowadays many groups claim to use scientific methods, but their practices often don’t match scientific evidence. This research aimed to conduct a literature review to explore presence of new empirical evidence supporting eCOA scientific best practices applied in the field.

### Methods

Searches were conducted across PubMed, Google Scholar, and the eCOA Consortium publications library using terms related to eCOA, ePRO, electronic diaries, design, digital data quality, data completeness, continuous monitoring, and patient engagement. Relevant publications from 2001 to 2024 were reviewed.

### Results

52 relevant abstracts were identified and reviewed. 26 focused on patient engagement, 7 on continuous remote monitoring, and 19 on best practices. Limited empirical evidence was found to supplement eCOA scientific best practices research since early 2000s. Significant research progress has been driven by wider availability of electronic devices on the topic of measurement comparability across various data capture modes. Other publications discussed: user-friendly intuitive eCOA design, flexible data collection and monitoring methods, patient-centered protocol design, and comprehensive participant and site training. These elements were highlighted as aims to improve data quality and completeness, but limited research data supported these recommendations, which primarily came from interest groups of field experts. Design-related topics that were discussed in the literature in early 2000s such as frequency of alarms or reminders, window of availability stopped being explored and consequently, new empirical evidence is missing.

### Conclusion

There is a need for new, robust guidelines that merge efficient and scientifically robust eCOA design in the era of routine digital data capture and present best practices for the field to implement.

## 1057 Patient-reported outcome measures used for differences of sex development: readability analysis and content mapping

Calvin R. Schuster^1^, Mélise Keays^2^, Reade Otto-Moudry^3^, Merel H. J. Hazewinkel^4^, Kevin Zhangxu^1^, Katherine Regis^2^, Jiali Cai^2^, Anh-Dao Cheng^2^, Carrie Wade^5^, Yee-Ming Chan^2^, Manraj N. Kaur^1^

^1^Brigham and Women’s Hospital, Harvard Medical School, Boston, Massachusetts, USA, ^2^Boston Children’s Hospital, Harvard Medical School, Boston, Massachusetts, USA, ^3^Geisel School of Medicine at Dartmouth, Hanover, New Hampshire, USA, ^4^Weill Cornell Medicine, New York, New York, USA, ^5^Harvard Medical School, Boston, USA

*Journal of Patient-Reported Outcomes 2026*, **10(Suppl 1)**:1057

### Aims

Congenital differences of sex development (DSD) include a range of conditions characterized by atypical chromosomal, gonadal, or anatomic sex. For patient-reported outcome measures (PROMs) to representatively capture outcomes, they must be easy to understand and comprehensive. The range, quality, and accessibility of PROMs used for DSD populations remain poorly characterized. The aim of this study was to assess the readability and evaluate the comprehensiveness of PROMs for individuals with DSD.

### Methods

A comprehensive search was conducted across electronic databases (Ovid MEDLINE, Embase, PsycINFO, CINAHL, Cochrane) from inception to September 15, 2023. Original studies published in English and reporting on PROMs for individuals with DSD were included. Studies reporting exclusively on caregiver-reported outcomes, reviews, conference abstracts, and letters to editors were excluded. For PROMs used by three or more studies, readability was assessed using established readability metrics, including the Coleman Liau Index, Flesch Kincaid Grade Level, Gunning Fog Index, and Simple Measure of Gobbledygook Index. To evaluate for comprehensiveness, the content of PROMs was mapped against established health-related quality-of-life frameworks. Descriptive statistics were calculated.

### Results

From 163 studies that met inclusion criteria, 23 PROMs were included in the readability analysis. Most frequently studies were conducted in Germany (n=27), the United States (n=25), or the Netherlands (n=18). Average participant ages ranged from 7 to 50 years. The most frequently used PROMs across all groups included the Female Sexual Function Index (n=19), Pediatric Quality of Life Inventory (n=15), and WHOQOL-BREF (n=14). Readability ranged from a reading grade level of 1.9-6.3 across PROMs designed for pediatric populations, 2.2-6.0 for adolescent/adults, and 3.5-9.5 for adults (Figure 1). A majority (n=12, 52.2%) failed to meet grade level readability recommendations. Individual PROM items (n=580) were mapped to seven domains. Most mapped to Psychological Well-being (n=257, 44.3%), Sexual Well-being (n=100, 17.2%), and Physical Function (n=88, 15.2%). Multiple PROMs contained outdated terminology and double-barreled questions.

### Conclusion

Most of the PROMs commonly used in the DSD literature failed to meet readability recommendations for patient-facing materials. Substantial heterogeneity was noted in PROM content and no single PROM was found to be comprehensive. A validated DSD-specific PROM is urgently needed.

**Fig. 1 (abstract 1057):**
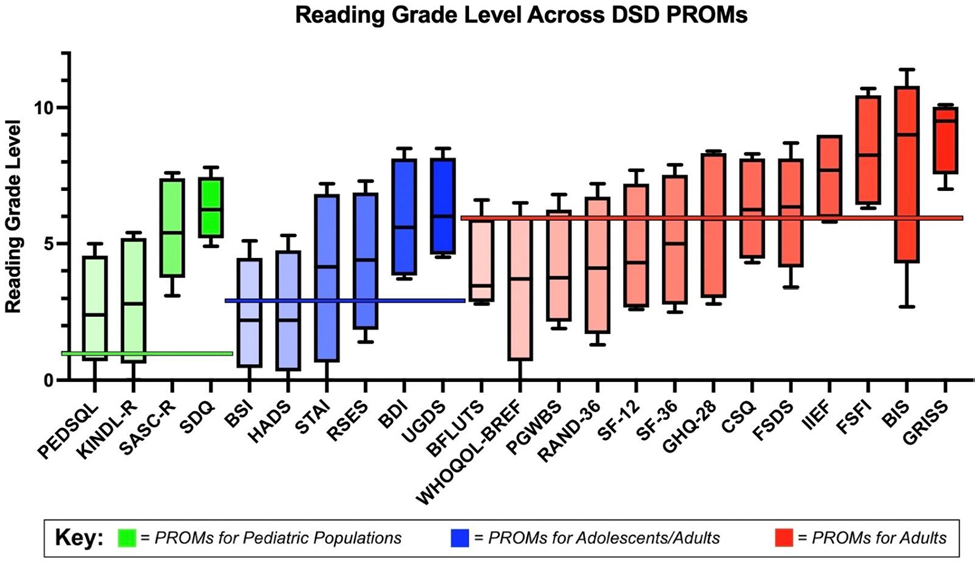
Reading Grade Level Across DSD PROMS The green boxes (left) represent PROMs designed for use exclusively in pediatric patient populations (under 18 years old). The blue boxes (middle) represent PROMs designed or validated for use in adolescent and adult populations (13 years old and older). The red boxes (right) represent PROMs designed for adult populations (18 years old and older). The red line marks the recommended reading grade level for patientfacing material for adults, grade 6. The green line marks reading grade level 1 and the blue line marks reading grade level 3. Boxes represent the interquartile range, whiskers represent the minimum to maximum, and the line represents the median reading grade level for each PROM based on the Coleman Liau Index, Flesch Kincaid Grade Level, Gunning Fog Index, and Simple Measure of Gobbledygook Index

## 1058 Design and Implementation of an Optimized ePRO System for Thoracic Surgery Patients (Recovery Monitor): a study protocol

Amanda Gentry^1^, Meghan O’Leary^2^, Allison Deal^3^, Jennifer Leeman^4^, Ethan Basch^3^, Chase Cox^5^, Kacee Little^3^, Jason Long^1^, Ben Haithcock^1^, Lauren Hill^6^, Gita Mody^1^

^1^University of North Carolina, Chapel Hill, North Carolina, USA, ^2^UNC Gillings School of Public Health, Chapel Hill, North Carolina, USA, ^3^UNC Lineberger Comprehensive Cancer Center, Chapel Hill, North Carolina, USA, ^4^UNC School of Nursing, Chapel Hill, North Carolina, USA, ^5^UNC School of Medicine, Chapel Hill, North Carolina, USA, ^6^UNC Health, Chapel Hill, USA

*Journal of Patient-Reported Outcomes 2026*, **10(Suppl 1)**:1058

### Aims

Thoracic surgery often leads to significant postoperative complications and symptom burden due to the complexity of the procedures and patients’ comorbidities. Traditional post-discharge care methods may fail to capture the full scope of patient recovery, highlighting the need for improved monitoring strategies. Routine collection and monitoring of electronic patient-reported outcomes (ePROs) provides real-time, actionable patient data, but guidance on their design and implementation in surgical settings for remote symptom monitoring is limited.

### Methods

The Recovery Monitor Study (NCT06075316) utilizes a sequential, explanatory design to evaluate an optimized ePRO system for thoracic surgery. Implementation is guided by an integrated, multi-level conceptual framework (RE-AIM and CFIR) and evidence-based practices. Phase 1 involves enrolling 100 patients to a refined ePRO intervention, assessing initial outcomes, and gathering qualitative feedback from patients and providers through semi-structured interviews. Data integration and mixed methods analysis will inform the development of an optimized intervention and implementation strategies. Phase 2 will enroll an additional 100 patients to the optimized ePRO intervention, with re-measurement of outcomes.

### Results

101 patients were enrolled and participated in post-discharge ePROs during Phase 1. 18 patients and 10 providers have undergoing semi-structured interviews to understand gaps and barriers to implementation. Findings will be integrated and used to inform the development of an optimized intervention (Table 1).

### Conclusion

This study aims to develop an optimized ePRO system specially for remote symptom monitoring of patients following discharge from thoracic surgery. Its strengths lie in its iterative design, theoretical framework, and application of implementation science. Our findings will address a critical gap in the literature regarding the implementation of ePRO systems in surgical practice, offering the potential to significantly enhance patient care and postoperative outcomes.

**Fig. 1 (abstract 1058):**
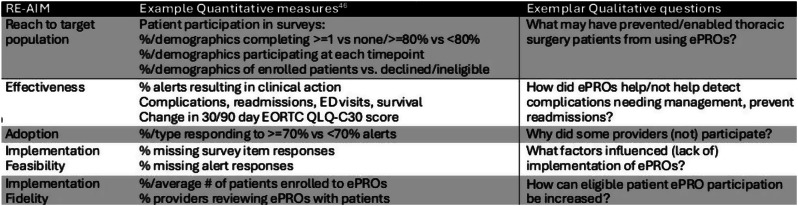
Design and Implementation of an Optimized ePRO System for Thoracic Surgery Patients (Recovery Monitor): a study protocol

## 1059 Assessing linguistic and cultural appropriateness of patient-reported outcome measures (PROMs) for people with a migration background

Lisa Stikvoort^1^, Ellen Elsman^2^, Olga Damman^3^, Caroline Terwee^2^, Lotte Haverman^4^

^1^Amsterdam UMC, Amsterdam, Netherlands, ^2^Amsterdam UMC, location University of Amsterdam, Epidemiology and Data Science, Amsterdam, The Netherlands, ^3^Amsterdam Public Health Research Institute, Amsterdam, Netherlands, ^4^Amsterdam UMC, location University of Amsterdam, Emma Children’s Hospital, Child and Adolescent Psychiatry & Psychosocial Care, Amsterdam, The Netherlands

*Journal of Patient-Reported Outcomes 2026*, **10(Suppl 1)**:1059

### Aims

An estimated 27.8% of the Dutch population has a migration background, with many being non-native Dutch speakers. This may hinder their completion of patient-reported outcome measures (PROMs). As a result, they do not benefit from the clinical use of PROMs and are underrepresented in clinical trials and PROM research, further exacerbating health inequalities. PROMIS measures are available in many different languages, but these translations were developed and tested in the country where the language is natively spoken. It thus remains unclear whether they are appropriate for migrants living in the Netherlands, who are not proficient in Dutch. Therefore, this study aimed to assess whether available English, German, French, Spanish, Turkish and Arabic PROMIS short forms are linguistically and culturally appropriate for use in the Dutch healthcare setting.

### Methods

The following eight PROMIS short forms were assessed: Ability to Participate, Anxiety, Cognitive Function, Depression, Fatigue, Pain Interference, Physical Function, and Sleep Disturbance, totaling 81 items. Cognitive interviews are conducted with Dutch residents whose first language is English, German, French, Spanish, Turkish, or Arabic (regardless of country of origin; n=5/language) to assess linguistic and cultural appropriateness. Interviews are held in English or Dutch, but to involve a more vulnerable subgroup – those with no proficiency in either Dutch or English – additional interviews will be conducted with interpreter assistance (n=2/language).

### Results

Cognitive interviews have been completed with English (n=5), Spanish (n=2), German (n=1) and Arabic (n=1) speakers. No major linguistic or cultural issues were identified thus far. Additional interviews are ongoing to complete the full target sample per language. Final results will be presented at the ISOQOL conference.

### Conclusion

This study will evaluate the linguistic and cultural appropriateness of translated PROMIS short forms for application in the Dutch healthcare context. Next, measurement properties will be evaluated through testing in a large sample (n=200/language) to ensure score comparability with existing PROMIS measures and other populations. The use of PROMIS short forms in other languages will ensure that Dutch residents with a primary language other than Dutch can benefit from the use of PROMs in clinical practice and research, ultimately contributing to reduced health inequities.

## 1060 Characterizing Variability in Patient Global Impression of Change (PGIC) and Severity (PGIS) Measures: Towards Standardization of PRO Design to Improve Development, Comparability, Translatability, and the Patient Experience

Tim Poepsel^1^, Rebecca Israel^2^, Allyson Nolde^3^, Rachael Browning^4^, Chryso Hadjidemtriou^5^

^1^RWS, St. Louis, Missouri, USA, ^2^RWS, Philadelphia, Pennsylvania, USA, ^3^RWS, Chicago, Illinois, USA, ^4^RWS, Oxfordshire, UK, ^5^RWS, London, UK

*Journal of Patient-Reported Outcomes 2026*, **10(Suppl 1)**:1060

### Aims

Previous research reveals significant levels of variability in the design of common and critical features of PROs, such as domain-specific response sets (e.g., assessing ‘frequency’ or ‘severity’), and suggest that such variability can impact the patient experience, translatability, data quality, and comparability. Here, we extend this investigation of variability to an entire class of PROs; specifically analyzing PGIs, which though common instruments in clinical research, are frequently developed without rigorous validation and can vary extensively in design depending on intended modality (paper; eCOA). We review all PGI features (e.g., item stems, response sets, instructions) and wording choices, to characterize the range of existing variability and develop a framework for standardization and optimization of each feature, which researchers can use to facilitate instrument development or selection.

### Methods

To characterize variability in PGI features, we analyzed a convenience sample (32 PGICs; 32 PGISs) from translation projects completed in the last 5 years.

### Results

Across 32 PGICs, we found 13 unique response sets, and 17 recall periods. 50% of items were phrased as questions, with 28% in the first person. 44% included instructional text; 40% included additional descriptive text. Items started with recall periods 65% of the time. Response sets contained 5 to 8 response options (Mode: 7). There were 12 general item stems, where variables were recall period and symptom.Across 32 PGISs, we found 12 unique response sets, and 13 recall periods. 44% of items were phrased as questions; 28% contained instructional text; 16% contained additional descriptive text. Recall periods appeared in final position in 87% of items, and 6% each for medial and initial position. Response sets had 4 to 7 response options (Mode: 5). There were 9 general item stems, where variables again were recall period and indication/treatment.

### Conclusion

We provide comprehensive data on variation in PGI features and wording to support standardization efforts. Researchers can use this data to optimize COA design or selection regardless of modality, target demographic, or indication, with potential positive effects on comparability, translatability, data quality, and the patient experience, as COAs and expectations about them converge on more straightforward and familiar structures and language.

## 1062 Perceived stress, coping strategies and associated factors among Colombian undergraduate health science students

Martha Rodriguez^1^, Yeny Z. Castellanos-Dominguez^1^, Jhancy R. Aguilar-Jimenez^2^, Sahira G. Franco-Hernández^2^, Tito C. Quintero-Gómez^3^, Paula C. Ramirez^3^

^1^Universidad Santo Tomás, Bucaramanga, Colombia, ^2^Universidad de Santander, Bucaramanga, Colombia, ^3^Universidad Industrial de Santander, Bucaramanga, Colombia

*Journal of Patient-Reported Outcomes 2026*, **10(Suppl 1)**:1062

### Aims

To assess perceived stress, coping strategies and associated factors among Colombian undergraduate health science students.

### Methods

An analytic cross-sectional study was conducted with 783 students, all over 18 years of age, who were selected by simple random probability sampling from thirteen academic programs in the health sciences offered by three universities (two private and one public). The students completed the Spanish versions of the Perceived Stress Scale (PSS-14) and Brief COPE (COPE-28). In addition, sociodemographic variables related to lifestyle and the academic environment were collected. PSS-14 scores higher than 28 were considered indicative of high perceived stress. Frequencies and proportions were calculated for the qualitative variables, and measures of central tendency and dispersion were calculated for the quantitative variables. A backward, stepwise logistic regression was performed including all explanatory variables with p < 0.15 on bivariate logistic regression. A p-value < 0.05 was considered statistically significant.

### Results

The median age of the participants was 21 years (interquartile range [IQR] 19-23), 550 (70.2%) were women, 228 (29.1%) worked in addition to studying, and 193 (24.9%) were in their second year of university. The overall mean (±SD) PSS-14 score was 27.6 (±7.5); high perceived stress was reported by 345 (44,1%) students. Among the sociodemographic, lifestyle, and academic information collected, being male (adjusted OR = 0.44, 95% CI 0.29–0.65), having a medication-taking routine (AOR = 2.04, 95% CI 1.27–3.29), and attending a public university (AOR = 2.37, 95% CI 1.38–4.07) were associated with high perceived stress. On the other hand, self-distraction (AOR = 1.13, 95% CI 1.02–1.27), behavioral disengagement (AOR = 1.16, 95% CI 1.03–1.31), self-blame (AOR = 1.77, 95% CI 1.54–2.03), using informational support (AOR = 0.80, 95% CI 0.70–0.92) and planning (AOR = 0.71, 95% CI 0.62–0.81) as means of coping with stress were also associated with high perceived stress.

### Conclusion

High perceived stress was reported by 44.1% of the participants. Students employed different coping strategies, with active coping showing the highest mean score and substance use the lowest. Three subscales in avoidant coping and two in approach coping were associated with high levels of perceived stress.

## 1064 Quality of Life and Return to Duty and Combat Readiness Among Obese Active-Duty and Retired Military Personnel with Low Back Pain and other Spine Conditions: A Scoping Review

Amy Cizik^1^, Seodam Kwak^1^

^1^University of Utah, Salt Lake City, Utah, USA

*Journal of Patient-Reported Outcomes 2026*, **10(Suppl 1)**:1064

### Aims

Chronic low back pain (cLBP) is a leading cause of lost productivity and disability worldwide, being an especially high burden among veterans and military personnel decreasing combat readiness and return to duty. Among the many risk factors contributing to low back pain and its treatment outcomes, obesity is a critical but often overlooked determinant, particularly as it is prevalent in veteran populations and is increasingly affecting active-duty service members. Additionally, obesity (body mass index (BMI) >30) is associated with chronic low grade systemic inflammation and linked to higher rates of comorbid conditions such as diabetes, osteoarthritis, and cardiovascular disease. A scoping literature review was performed to identify the role of obesity in active-duty and veteran military population with cLBP or other spine conditions with or without surgical interventions.

### Methods

Using the JBI Manual for Evidence Synthesis 2024, a scoping review of existing literature for adults who are active or retired United Stated military with cLBP and other spine conditions, stratified by BMI and treatment interventions (surgical or other) as our cohort that contained any mention of BMI and quality of life outcomes, including validated PROMs, HRQoL domains and single item questions. Exclusion criteria included case studies, biomechanical papers, foreign military, malignancies, spinal cord injury, and congenital malformations. Literature from 2000 –2024 were identified through electronic database searches in PubMed, Embase, Web Science, and SCOPUS.

### Results

A total of 10522 studies were screened. Eligible articles contained key words such as veterans, military, low back pain, spine, degenerative spine, spinal fusion, spine surgery, and obesity. Of the articles that were screened, 736 studies were identified for full text review. Currently, 43 articles have been extracted, PROMs included SF-36, VR-12, ODI, and PROMIS domains.

### Conclusion

The impact of obesity on the treatment of spine pathologies and cLBP in military personnel remains poorly studied. Military members have unique risks due to the physically demanding nature of training and service, and obesity only further exacerbates the risk of cLBP and poor treatment outcomes. This study is the first step to assess gaps in research, areas of intervention for quality-of-life improvement, combat readiness, and return to duty.

## 1066 An “argument-based” approach to validity applied to an endocrine therapy measure of “medication usage”

Kyra Kapsaskis^1^

^1^UNC - Chapel Hill, Chapel Hill, North Carolina, USA

*Journal of Patient-Reported Outcomes 2026*, **10(Suppl 1)**:1066

### Aims

Medication adherence measures have not been widely studied, however, adherence can help improve treatment and Quality of Life (QOL) outcomes. Argument-based approaches are not the most frequently used method in the validation process for PROMs, in favor of more traditional quantitative methods. Dr. Kevin Weinfurt created an argument-based template of assumptions specifically for the assessment of PROMs (Weinfurt, 2021) in an effort to minimize the gap between this method of validity assessment and others that may be used out of habit more so than being the best representation of validity for their measure. I’m focusing on a “medication usage” measure used for Endocrine Therapy adherence (Wheeler et al., 2019) in breast cancer patients and aim to apply Dr. Weinfurt’s template of assumptions to assess the validity of this measure and make recommendations for its improvement.

### Methods

Dr. Weinfurt’s framework progresses through a series of assumptions that he has written as a means of helping PROM researchers apply this theory to their work. My method has been to compare his Assumptions A-F and his recommended evidence needed to support each Assumption with what I could find in the original paper for which this measure was created and used. When further support was necessary, I made recommendations for what this study could specifically do to increase validity.

### Results

Assumptions A and B were the most supported, and Assumptions C.1-C.6 could use further data collection and testing to support. These assumptions mostly address construct validity, reliability, cultural/linguistic differences, and literacy/education levels.

### Conclusion

The argument-based approach has been very effective in generating recommendations to improve this measure’s validity. Among them are to conduct a factor analysis to determine if both concepts of ‘adherence’, ‘usage’ and ‘discontinuation’, are being captured; compare the two questionnaire delivery modes (phone and mail); measure the frequencies in each response option to test for ceiling and floor effects; collect additional data on comorbid health conditions; introduce a free-response option for patients to describe the medication usage experience; measure recall accuracy two weeks later; and implement a logical scoring method. This argument-based approach should be implemented as a standard approach to validity for PROMs.

**Fig. 1 (abstract 1066):**
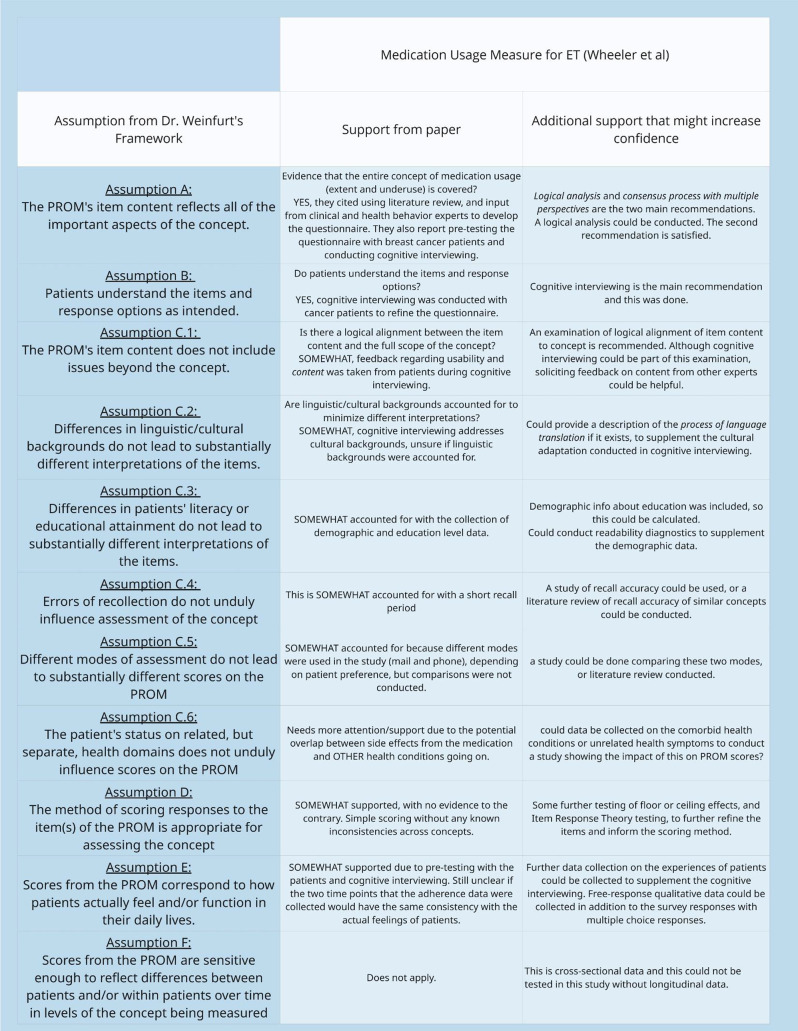
An “argument-based” approach to validity applied to an endocrine therapy measure of “medication usage”

## 1068 Quality of Life in the Early Period After Allogeneic Hematopoietic Stem Cell Transplantation: Dynamics and the Impact of GVHD

Gabriela Lampart^1^, Agnieszka Gniadek^2^, Beata Piątkowska - Jakubas^3^

^1^Jagiellonian University Medical College, Doctoral School of Medical and Health Sciences, Krakow, Poland, ^2^Jagiellonian University Medical College, Faculty of Health Sciences, Institute of Nursing and Midwifery, Krakow, Poland, ^3^Jagiellonian University Medical College, Faculty of Medicine, Chair of Hematology, Krakow, Poland

*Journal of Patient-Reported Outcomes 2026*, **10(Suppl 1)**:1068

### Aims

Quality of life (QoL) is a crucial aspect of recovery following allogeneic hematopoietic stem cell transplantation (allo-HSCT). This complex procedure, involving intensive conditioning regimens and immunosuppressive therapy, often leads to complications such as graft-versus-host disease (GVHD), which can severely impact patients’ overall well-being. Monitoring QoL in the early post-transplant period and understanding the effects of GVHD and its treatment are essential for improving long-term outcomes.

### Methods

This study assessed quality of life in 28 patients during the first six months after allo-HSCT using the validated FACT-BMT questionnaire. Assessments were conducted at three time points: the day after transplantation, one month later, and six months post-transplant. Patients completed the questionnaire independently. Based on GVHD status and response to therapy, participants were categorized into three groups: (1) those with GVHD showing complete or partial response to treatment, (2) those with GVHD without regression, and (3) a control group without GVHD. Statistical analyses were performed to compare QoL scores over time and between groups.

### Results

Patients who developed GVHD reported lower quality of life compared to those without GVHD. QoL was particularly diminished during the early post-transplant period. Differences in outcomes were also observed between patients with GVHD who responded to treatment and those who did not, highlighting the influence of disease control on self-reported well-being.

### Conclusion

Reduced quality of life is a significant concern in the early period following allo-HSCT, especially among patients experiencing active GVHD. Regular monitoring using tools like FACT-BMT and individualized supportive care strategies based on GVHD status may enhance recovery. Integrating QoL-focused interventions into post-transplant protocols can play a vital role in addressing this important, yet often overlooked, dimension of patient care.

## 1070 Design and Implementation of Data Quality Controls in the EQ-DAPHNIE Study: Insights from the Pilot Phase and 15-Country Analysis

Fatima Al Sayah^1^, Hilary Short^1^, Juan M. Ramos-Goñi^2^, Rosalie Viney^3^, Erica I. Lubetkin^4^, Mathieu F. Janssen^5^, Jeffrey A. Johnson^1^

^1^Alberta PROMs & EQ-5D Research & Support Unit (APERSU), School of Public Health, University of Alberta, Edmonton, Alberta, Canada, ^2^Decision Analysis and Support Unit, SGH Warsaw School of Economics, Tenerife, Spain, ^3^University of Technology Sydney, Sydney, Australia, ^4^CUNY School of Medicine, New York, New York, USA, ^5^EuroQol Research Foundation, Rotterdam, Netherlands

*Journal of Patient-Reported Outcomes 2026*, **10(Suppl 1)**:1070

### Aims

The EQ-DAPHNIE study is a large-scale, multi-country initiative designed to collect representative data on self-reported health status from diverse populations through online panel surveys. The goal is to generate population norms, facilitate cross-country comparisons in self-reported health using widely recognized measures such as the EQ-5D-5L, PROMIS-10, and WHO-5. This paper describes the data quality control measures implemented in the EQ-DAPHNIE project, and presents the results of quality metrics from the pilot phase and analysis across 15 countries.

### Methods

The pilot phase was conducted in the United Kingdom, with a target sample of 3000 adults, to evaluate survey design, data quality, and question performance. Results from the pilot informed the final survey design, which was implemented across 15 countries, targeting 4,500 adults per country. Data quality metrics analyzed included completion rates, dropouts, consent rates, duplicate records, ‘bot’ detection, speeders, response consistency, missing data, outliers, and quota achievement.

### Results

The pilot phase (n=3,012) informed several key adjustments in the survey design. Non-mandatory questions were retained after analyzing missing data patterns, and open-text fields were replaced with drop-down lists to minimize outliers. Bot activity and speeders were identified, with minimal exclusions. Repetitive questions were modified to improve consistency checks. In the main data collection phase across 15 countries (n=68,411), response rates ranged from 80.1% to 100%, with most countries achieving rates above 90%. Completion rates ranged from 22.9% to 60.8%, with an average of 42.4%. Non-completion rates varied from 34.2% to 70.1%, averaging 50.5%. Bot detection flagged 0.2% to 11.7% of responses, with an average of 3.0%. Speeding was observed in 0.3% to 0.9% of responses. Missing data varied from 0.0% to 48.7%, and quota achievement ranged from 68.7% to 98.6%. Data collection durations ranged from 32 to 58 days across countries.

### Conclusion

The quality control measures implemented throughout the EQ-DAPHNIE project effectively addressed common issues such as bot responses, speeding, and missing data, resulting in generally high-quality datasets. However, variations across countries in engagement, missing data, and quota achievement point to areas for improvement in future iterations.

## 1072 Development and Content Validation of a Survey Instrument to Assess Physician Attitudes Toward Real-World Evidence in U.S. Cancer Care Using Modified COSMIN Methodology

Thomas Porter^1^, Sabrina Figueiredo^1^

^1^George Washington University School of Medicine and Health Sciences, Washington, District of Columbia, USA

*Journal of Patient-Reported Outcomes 2026*, **10(Suppl 1)**:1072

### Aims

Real-world evidence (RWE) is increasingly relevant in oncology, yet robust tools for measuring cancer physicians’ attitudes toward RWE are lacking. This study aimed to develop and refine a survey instrument to assess U.S. cancer physicians’ knowledge, perceptions, and practices regarding RWE, using a modified COnsensus-based Standards for the selection of health Measurement INstruments (COSMIN) approach to support content and face validity.

### Methods

Qualitative study, approved by the George Washington University School of Medicine and Health Sciences IRB. An initial draft of the survey (nine standalone items, nine Likert-scale items, eight barriers, eight facilitators, and four demographic questions) was adapted from published instruments. Eight actively practicing, licensed U.S. cancer physicians were invited to participate in a cognitive interview to assess the survey’s instrument comprehensiveness, relevance, and comprehensibility between September 6, 2024 and October 14, 2024. A semi-structured guide was used for concept elicitation and cognitive interview.

### Results

Eight practicing U.S. oncology physicians each reviewed the survey once and provided feedback on clarity, relevance, and comprehensiveness. Expert input prompted the addition of items reflecting constructs from the Theory of Planned Behavior (e.g., subjective norms, perceived behavioral control) and the separation of data accuracy and completeness into distinct barriers. This process yielded a revised survey with 18 standalone items, 9 Likert-scale items, 10 barriers, 6 facilitators, and 4 demographic questions. New elements included a visual sliding scale comparing reliance on RWE with reliance on randomized trial evidence, plus questions on prior research experience with RWE. Informal piloting with a small subset of cancer physicians indicated the instrument was comprehensible and easy to navigate, with minimal need for further changes.

### Conclusion

Through iterative expert review and a modified COSMIN-based approach, we developed a refined survey instrument that assesses cancer physicians’ attitudes toward and utilization of RWE. This refined tool can guide future research on physician use of RWE in oncology clinical care.

## 2013 Reliability of “baseline” scores for traumatically injured patients: advantages of using the Patient-Reported Measurement Information System (PROMIS) domains

Amy Cizik^1^, Joshua Horns^1^, Tyler Thorne^1^, Justin Haller^1^, Lucas Marchand^1^, Conor Kleweno^2^

^1^University of Utah, Salt Lake City, Utah, USA, ^2^University of Utah, Seattle, Washington, USA

*Journal of Patient-Reported Outcomes 2026*, **10(Suppl 1)**:2013

### Aims

For patients with traumatic injuries it is difficult to collect a pre-injury or “baseline” assessment of patient-reported outcome measures (PROMs). Proxy measurement through family members or other caregivers has been described as a solution to collect information on prior function for the traumatically injured patient. This study aimed to measure pre-injury function and compare it to population norms by asking patients at their 2-week follow-up visit to estimate their pre-injury function using 21 items from the PROMIS physical function (PF) bank, and Short Forms for Pain Interference (PINT), Anxiety (ANX), Depression (DEP), Sleep (SLP), and Sexual Function (SEX).

### Methods

This was a 3-year prospective multi-center observational study of adult patients with isolated pelvic and/or acetabular fractures undergoing operative and non-operative treatment. Data collection included 2-week post-injury “baseline” for pre-injury recall, 3 month, and 6 month timepoints. Participants at “baseline” were prompted with the following instruction: “Please answer the following questions based on how you felt and acted before your injury.”

### Results

A total of 236 participants with pelvis and acetabular fractures were enrolled in the study, of those only 48% completed all three timepoints for the primary measure (PROMIS PF) of the study 114 patients are included with an average age of 54 years (SD=15), 58% identified as male, and a mean Social Deprivation Index score of 33. Participants on average had a pre-injury PROMIS PF t-score = 57.7 (SD=8.7) and a median score of 63.4 (IQR = 54.4 – 63.4). For other domains, the average PROMIS t-score was PINT=45.5 (SD=6.3), ANX=47.5 (SD=8.2), DEP = 46.6 (SD=7.17), SLP = 46.0 (SD=8.7), and SEX = 58.2 (SD=6.9).

### Conclusion

In this sample of people with traumatic fractures of the acetabulum and pelvis, the average pre-injury PROMIS PF t-score was >1 standard deviation higher than that of the general population. Additionally, sexual function was also to being 1 SD higher (≥ 60) than a representative sample with chronic conditions. All other domains were estimated to be “within normal limits”. Using measures that are standardized to a reference population can help guide clinicians in counseling patients for whom their baseline score is not individually known.

**Fig. 1 (abstract 2013):**
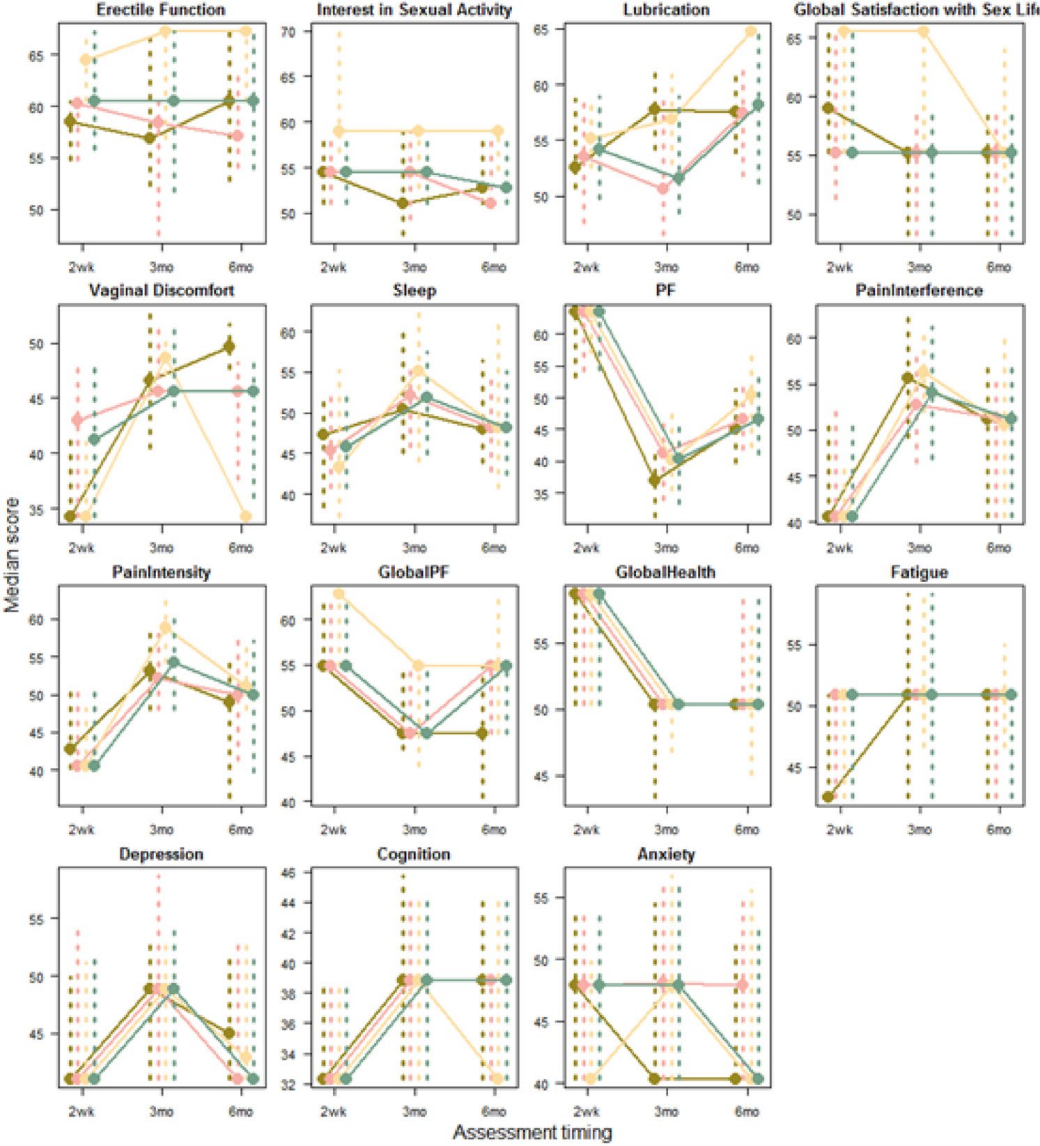
Reliability of “baseline” scores for traumatically injured patients: advantages of using the Patient-Reported Measurement Information System (PROMIS) domains

## 2014 A Qualitative Study to Understand the Supportive Care Needs of Adolescents with X-Linked Hypophosphatemia during the Transition to Adulthood

Angela Rylands^1^, Vrinda Saraff^2^, Pedro Arango Sancho^3^, Justine Bacchetta^4^, Annemieke Boot^5^, Christine Burren^6^, Amish Chinoy^7^, Poonam Dharmaraj^8^, Adele Barlassina^9^, Agnès Linglart^10^, Maria Amelia Gómez Llorente^11^, Juan David González Rodríguez^12^, Iva Gueorguieva^13^, Wesley Hayes^14^, Héctor Ríos Duro^15^, Emily Hardie^16^, Dirk Schnabel^17^, Santhani Selveindran^18^

^1^Kyowa Kirin Ltd, Marlow, UK, ^2^Birmingham Women’s and Children’s Hospital, Birmingham, UK, ^3^Sant Joan de Déu Barcelona Hospital, Barcelona, Spain, ^4^Hospices Civils de Lyon, INSERM1033 Research Unit, Lyon, France, ^5^University Medical Center Groningen, University of Groningen, Groningen, Netherlands, ^6^University Hospitals Bristol and Weston NHS Foundation Trust, Briston, UK, ^7^Royal Manchester Children’s Hospital, Manchester, UK, ^8^Alder Hey Children’s Hospital, Liverpool, UK, ^9^Open Health Ltd, Rotterdam, Netherlands, ^10^AP-HP, Paris Saclay University, Paris, France, ^11^Hospital Virgen de Las Nieves, Granada, Spain, ^12^Department of Pediatric Nephrology, Santa Lucia General University Hospital, Cartagena, Spain, ^13^Centre Hospitalier Universitaire de Lille, Lille, France, ^14^Great Ormond Street Hospital, London, UK, ^15^Pediatric Nephrology, Vall d´Hebron Universitary Hospital, Barcelona, Spain, ^16^Kyowa Kirin International, Marlow, UK, ^17^Center for Chronic Sick Children, Pediatric Endocrinology, Charité – University Medicine Berlin, Berlin, Germany, ^18^Open Health Ltd, London, UK

*Journal of Patient-Reported Outcomes 2026*, **10(Suppl 1)**:2014

### Aims

X-linked hypophosphatemia (XLH) is a rare, genetic disorder caused by pathogenic variants in the phosphate-regulating endopeptidase homologue X-linked (PHEX) gene. It leads to progressive musculoskeletal manifestations, including limb deformities, short stature, and pain, beginning in early childhood and persisting into adulthood. Data on the lived experiences of adolescents with XLH and the perspectives of their carers during transition to adulthood (i.e. at the end of skeletal growth, EoSG) are limited. During this critical time treatment options vary and as such this study aimed to explore the supportive care needs of adolescents with XLH and the associated carer burden.

### Methods

A prospective, observational, multicenter, mixed-methods study was conducted across 14 specialist pediatric centers in the UK, France, Spain, the Netherlands, and Germany (NCT05181839). Semi-structured telephone interviews with carers of adolescents with XLH at EoSG were conducted to explore socio-demographic and clinical backgrounds, caregiving impact on health, daily life, and family dynamics, treatment satisfaction, and information and support needs. Transcriptions were analysed using the Framework method, including data familiarisation, codebook creation, indexing, charting, and interpretation.

### Results

Each of the twelve carers interviewed described providing both emotional and practical support to adolescents with XLH. Some carers (n=5) reported an impact to their physical health including reports of exhaustion and headache, as well as their mental and emotional health including feelings of worry, anger and guilt. The impact of XLH on their lives diminished as their children aged and began treatment with burosumab, with minimal change noted at EoSG. Most (n=9) carers expressed satisfaction with the care their children received and demonstrated knowledge of XLH management. Support from healthcare professionals, family, and patient groups was reported as adequate. Carers voiced concerns regarding the long-term impact of XLH on their children’s future work and social lives, alongside hopes for continued access to burosumab.

### Conclusion

The study underscores the importance of understanding the experiences of carers caring for adolescents with XLH at EoSG. While support systems appear effective, ongoing concerns about future challenges remain, indicating a need for continued dialogue and support for families affected by XLH.

## 2015 Comparison of the EQ-5D-5L cognition bolt-on and EQ-HWB cognition items: Results from EQ-DAPHNIE data in China and Japan

Hilary Short^1^, Fanni Rencz^2^, Mathieu F. (Bas) Janssen^3^, Jeffrey A. Johnson^1^, Fatima Al Sayah^1^

^1^University of Alberta, Edmonton, Alberta, Canada, ^2^Corvinus University of Budapest, Budapest, Hungary, ^3^ EuroQol Research Foundation, Rotterdam, Netherlands, ^1^University of Alberta, Edmonton, Alberta, Canada, ^1^University of Alberta, Edmonton, Alberta, Canada

*Journal of Patient-Reported Outcomes 2026*, **10(Suppl 1)**:2015

### Aims

Cognition is a key domain of health-related quality of life measured in two experimental EuroQol instruments–the EQ-HWB and the EQ-5D Bolt-on Toolbox–yet comparative evidence between them is lacking. This study compares the measurement of cognitive problems by the EQ-5D-5L cognition bolt-on and EQ-HWB cognition items in general adult populations of China and Japan.

### Methods

Cross-sectional data from the EQ-DAPHNIE project were used. Data were collected July 8 - August 8, 2024 in Japan (n=4502), and September 5 - October 14, 2024 in China (n=4519). Three cognitive items were examined: the EQ-5D-5L cognition bolt-on (cognition); and two EQ-HWB items – “trouble remembering” (remembering) and “trouble concentrating/thinking clearly” (concentrating). Descriptive statistics and Spearman’s correlation coefficients were computed.

### Results

Mean age (SD) was 46.8 (15.1) in China and 55.2 (14.4) in Japan; 47.4% and 50.2% were female, respectively. Compared to China, more Japanese participants were single/divorced (42.9% vs. 13.5%), unemployed (19.5% vs. 1.8%), financially uncomfortable (22.0% vs. 6.6%), and living alone (24.4% vs. 3.8%). Mean (SD) EQ-5D-5L level sum scores (LSS) were 6.4 in both countries; EQ-HWB-9 LSS was 15.1 (5.2) in China and 14.7 (6.0) in Japan. In China, 40.8% reported problems (levels 2-5) in cognition, 52.8% in remembering, and 50.0% in concentrating. In Japan: 12.2%, 40.4%, and 30.1%, respectively. Among Chinese with no cognition problems, 17.3% reported remembering and 18.8% concentrating problems. In Japan: 30.2% and 20.9%, respectively. In China, reported problems increased with age across all items. In contrast, in Japan, problems decreased with age for cognition and concentrating and remained stable for remembering. In both countries, males and females reported similar levels of cognitive problems. Higher education and income groups reported fewer problems on all items in both countries. Participants with history of stroke and/or dementia reported more problems on all items, particularly in China. Cognition correlated more strongly with remembering in China (r=0.62) than Japan (r=0.42); correlations with concentrating were similar (China: r=0.46; Japan: r=0.41).

### Conclusion

EQ-HWB items identified more problems than the EQ-5D-5L bolt-on, especially in Japan. Variations in response scales and recall periods of the instruments may explain these patterns. Findings may inform refinement and localization of cognitive items for China, Japan.

## 2017 Symptom burden after dialysis initiation and associations with hospitalizations

Devin Peipert^1^, Devika Nair^2^, Xuan Cai^3^, Ron Hays^4^, Olalekan Aiyegbusi^5^, Rebecca Frazier^6^, Tamara Isakova^7^

^1^University of Birmingham, Birmingham, Illinois, UK, ^2^Division of Nephrology and Hypertension, Vanderbilt University Medical Center, Nashville, Tennessee, USA, ^3^Center for Translational Metabolism and Health, Institute for Public Health and Medicine, Northwestern University Feinberg School of Medicine, Chicago, Illinois, USA, ^4^Division of General Internal Medicine and Health Services Research, Department of Medicine, University of California, Los Angeles, USA, ^5^Centre for Patient-Reported Outcomes Research (CPROR), Department of Applied Health Sciences, College of Medicine and Health, University of Birmingham, Birmingham, UK, ^6^Department of Medicine, Duke University School of Medicine, Durham, North Carolina, USA, ^7^Division of Nephrology and Hypertension, Feinberg School of Medicine, Northwestern University, Chicago, USA

*Journal of Patient-Reported Outcomes 2026*, **10(Suppl 1)**:2017

### Aims

Symptom burden is distressing for patients living with kidney failure, but there is limited information about the combination of symptoms that best predict healthcare utilization in hemodialysis. We classified and summarized patients’ symptom burden levels and changes over time and estimated associations with hospitalizations among incident in-center hemodialysis patients.

### Methods

We conducted an observational, longitudinal study among dialysis patients in the United States. We employed latent transition analysis (LTA) to identify classes of symptom burden in a Kidney Disease Quality of Life-36 (KDQOL-36) dataset. LTA is a patient-centered approach that focuses on identifying profiles of individual patients instead of relationships between variables. We then applied Cox regression models to assess if symptom burden, in terms of individual symptoms from the KDQOL-36 and symptom burden groups, was associated with hospitalization risk after dialysis initiation, independent of demographics and comorbidities.

### Results

1818 participants were Black (29%), aged >65 years (59%), female (42%), had diabetes (49%), and hypertension (74%). LTA identified three symptom burden groups: low (low severity of all symptoms and kidney disease impacts), moderate (high physical health impact and overall burden of kidney disease), high (high levels of all symptoms and kidney disease impact). KDQOL-36 scale scores were significantly (p<0.001) distinct between symptom burden groups. Though distinct between groups, KDQOL-36 scores were largely stable over the 12 months after dialysis initiation. (Figure 1.) After adjusting for patient characteristics, all KDQOL-36 scales except Symptoms and Problems of Kidney Disease were associated with higher hazard of hospitalization. Using the symptom burden groups, high symptom burden was associated with a 20% increase in the hazard of hospitalization. (Table 1.) A 1-category worsening in pain interference and in fatigue was associated, respectively, with 12% and 8% increased hazard of hospitalization.

### Conclusion

A patient-centered approach was able to summarize symptom severity using the KDQOL-36. Assessing individual symptoms and overall symptom burden is useful for risk-stratifying future hospitalizations among in-center hemodialysis patients. Future research must examine how best to implement patient-reported HRQOL assessment into routine clinical dialysis care.

**Fig. 1 (abstract 2017):**
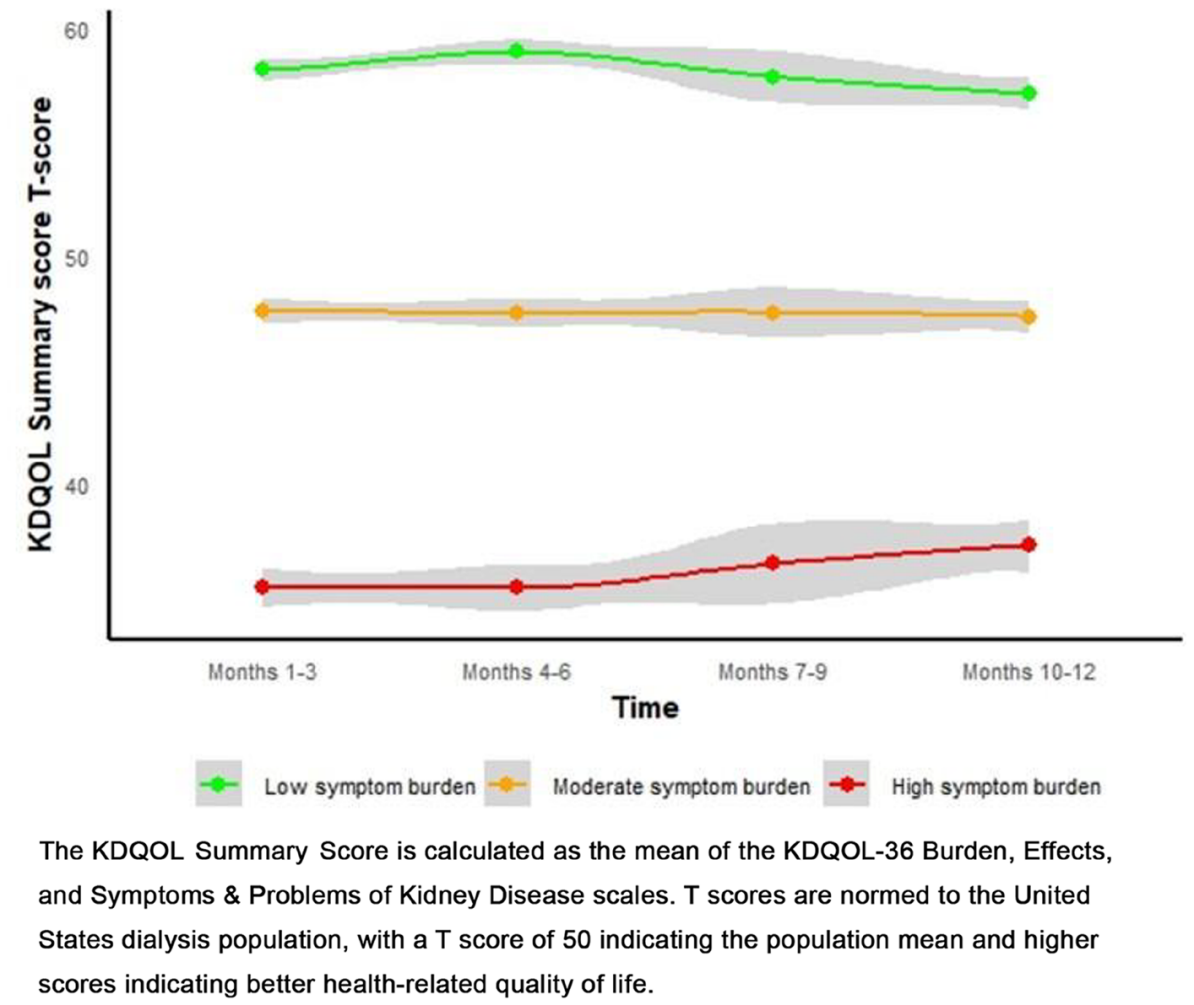
Mean KDQOL Summary Score T scores over time by symptom burden group

**Table 1 (abstract 2017):**
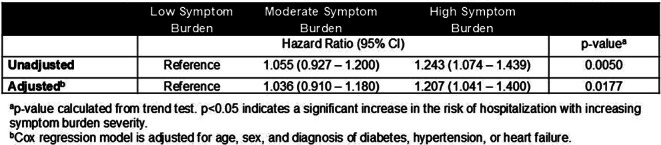
Risk of hospitalization after initializing dialysis associated with latent symptom burden group (N = 1818)

## 2018 Pre-testing items for a new pain-specific patient-reported outcome measure for pelvic floor surgery through patient and clinician feedback

Rasa Ruseckaite^1^, Sheymonti Hoque^2^, Susannah Ahern^2^, Helen O’Connell^3^

^1^Monash University, Melbourne, Australia, ^2^School of Public Health and Preventive Medicine, Monash University, Melbourne, Victoria, Australia, ^3^Department of Surgery, University of Melbourne, Melbourne, Victoria, Australia

*Journal of Patient-Reported Outcomes 2026*, **10(Suppl 1)**:2018

### Aims

Procedures to treat stress urinary incontinence and pelvic organ prolapse often result in post-surgical pain, negatively affecting the health-related quality of life of women. Patient-reported outcome measures (PROMs) that are currently available inadequately capture specific pain attributes and their relationship to pelvic floor disorders (PFDs). This study aimed to pre-test items for a new pain-specific PROM through focus groups, and then produce the first draft of the instrument.

### Methods

This qualitative study utilised seven Zoom focus groups with 15 adult Australian and New Zealand women with PFDs experiencing post-surgical pain and other surgical complications. In addition, consolidation occurred with the Australasian Pelvic Floor Procedure Registry Steering Committee (APFPR SC), comprising 11 clinicians. Women and clinicians provided feedback regarding 35 potential items for the new pain-specific PROM. Data obtained from the discussions were transcribed and thematically analysed using NVivo.

### Results

Women and clinicians agreed that the new measure could effectively address PFDs and pelvic floor surgery pain. Following the discussions, women suggested removing 14 out of the 35 items. Clinicians from the APFPR SC recommended removing five further items. Their feedback contributed to the design of the pain PROM. The preliminary instrument containing 16 items has been produced under seven pain-related domains: (1) region of pain, (2) pain triggers, (3) sensation of pain, (4) intensity and continuity of pain, (5) pain interference, (6) comorbidities and complications, and (7) pain relief and management.

### Conclusion

This qualitative study obtained input directly from women and clinicians regarding the formulation of items for the new pain-specific PROM. A preliminary version of the instrument was developed per the feedback. The pain measure will be the first of its kind for PFDs and pelvic floor surgery. Once it is fully developed and validated, the PROM could assist with shared patient-clinician decision-making and track pain-related health outcomes vital to women post pelvic floor procedure.

## 2019 Administering in-trial interviews using an AI chatbot: results of a proof of principle experiment

Bill Byrom^1^

^1^Signant Health, Nottingham, UK

*Journal of Patient-Reported Outcomes 2026*, **10(Suppl 1)**:2019

### Aims

To evaluate the feasibility of using an AI chatbot to conduct qualitative in-trial interviews, produce redacted transcripts, and generate summary reports in a clinical trial setting.

### Methods

A proof of principle experiment was conducted using Claude 3.5 Sonnet (Anthropic, October 2024) to perform an exit interview by role play with a mock participant from a hypothetical NSCLC trial. The AI was provided with background information on the trial design, patient profile, COAs included, and interview objectives. The chatbot was instructed to conduct the interview following qualitative interview best practices (provided in summary form), generate a redacted transcript removing personal and location identifiers, and produce a summary report of key findings. Interview performance was evaluated on conversation quality, appropriate use of follow-up probing, handling of slang/colloquialisms, expression of empathy, accuracy of redaction, and quality of the summary report.

### Results

The AI successfully conducted a structured interview exploring the participant’s trial experience and symptom assessment. The chatbot was able to: appropriately use probing questions to explore symptom impact, correctly interpret colloquialisms (e.g., “eat like a horse” for good appetite), and show empathy during conversation (e.g., “That sounds like quite a journey for each visit …”). Redaction of names and locations was performed accurately. The summary report effectively organized findings into key themes related to trial participation experience (travel and access, clinic experience, staff interactions, ePRO completion) and symptom assessment (most important and impactful symptoms, less relevant and missing symptoms). The report included relevant quotes with appropriate paraphrasing that maintained the original meaning.

### Conclusion

This experiment demonstrates the potential of AI chatbot technology to efficiently administer and report qualitative in-trial interviews in clinical trials. The approach could enable more extensive use of patient interviews by reducing the time and resources typically required for human-conducted interviews. While promising, further honing of the approach, and evaluation in multilingual settings is recommended given the predominance of English in current large language models. AI-facilitated interviews may ultimately represent a scalable approach to capturing patient input in key areas, including understanding trial participation and study feasibility, and generating evidence to support measure selection and validity in clinical trials.

## 2020 Development of a conceptual model for cognitive impairment in a rare blood disorder, Thrombotic thrombocytopenic purpura

Theresa Coles^1^, Laura Mkumba^1^, Benjamin Tillman^2^, Brian Adkins^3^, Dana Thompson^4^, Elizabeth Cieza^4^, Jamila Minga^1^, Kristin Bryne^2^, Toyosi Onwuemene^1^

^1^Duke University School of Medicine, Durham, North Carolina, USA, ^2^Vanderbilt University, Nashville, Tennessee, USA, ^3^UT Southwestern, Dallas, Texas, USA, ^4^Duke University, Durham, North Carolina, USA

*Journal of Patient-Reported Outcomes 2026*, **10(Suppl 1)**:2020

### Aims

Immune thrombotic thrombocytopenic purpura (iTTP) is a rare blood disorder. Survivors experience health-related quality of life (HRQoL) impacts such as cognitive impairment, depression, and fatigue. Cognitive function was described by patients as one of the most debilitating and important HRQoL impacts. Specificity concerning the types of cognitive domains affected remains insufficient. Without this specific knowledge, appropriate selection of clinical outcome assessments (COAs) for clinical trials is not possible. Therefore, our study aimed to develop a conceptual model of cognitive impairment in iTTP aligning with FDA’s Patient-Focused Drug Development (PFDD) guidance.

### Methods

We conducted qualitative interviews with 24 patient-observer pairs (48 total interviews) across three sites. Inclusion criteria for patients: ≥16 years of age with a confirmed diagnosis of iTTP, and English-speaking. Observers were ≥18 years of age and spend at least 3 hours a day, 4 days a week with the iTTP patient. Purposive sampling ensured diverse experiences were captured, primarily stratified by time since most recent TTP episode. Both patients and observers participated in video recorded, remote administration of semi-structured concept elicitation interviews. Clinical data (medications, hematological parameters, ADAMTS13 activity) from most recent hematology visits was collected to describe the sample clinically. Each video recording was transcribed by professional transcriptionists and then later analyzed using NVIVO software for coding and descriptive analysis. Patient and observer reports were compared. Analysis documented types of cognitive dysfunction from participants’ perspectives and the team collaboratively developed a draft conceptual model.

### Results

With IRB approval obtained, interviews will be completed by July 2025 and results by September 2025. Preliminary findings suggest cognitive difficulties emerge with the first iTTP episode, with fluctuating cognitive impairment during survivorship. A notable methodological challenge involved determining optimal approaches for successful recruitment with consideration of the cognitive capabilities of iTTP patients, including reminder protocols to ensure participant attendance at scheduled interviews.

### Conclusion

Results of this study will be used to match COAs to the domains identified in the conceptual model. This study advances understanding of iTTP-related cognitive impairment, and ultimately enhances patient-centered research in this rare disease.

## 2021 Psychometric Validation of the Weight and Emotions Scale (WES) in Adults with Obesity or with Overweight and Obesity-related Complications

Chisom Kanu^1^, Hayley Karn^2^, Claudine Clucas^2^, Iris Goetz^3^, Lisa M. Neff^4^, Kristina Boye^5^, Miriam Kimel^6^

^1^Eli Lilly & Company, Indianapolis, Indiana, USA, ^2^Evidera, Ltd, London, UK, ^3^Eli Lilly and Company, London, Indiana, UK, ^4^Eli Lilly and Company, Chicago, Illinois, USA, ^5^Eli Lilly and Company, Greenwood, Indiana, USA, ^6^Evidera, Inc., Wilmington, USA

*Journal of Patient-Reported Outcomes 2026*, **10(Suppl 1)**:2021

### Aims

This study evaluated the psychometric properties of the Weight and Emotions Scale (WES), a new patient-reported outcome (PRO) measure that assesses weight-related emotional function.

### Methods

Adults with obesity or with overweight and an obesity-related complication completed two web-based surveys approximately two weeks apart. The first survey included the 16-item WES and other PRO measures: Impact of Weight on Self-Perceptions Questionnaire (IW-SP), Control of Eating Questionnaire (CoEQ), Impact of Weight on Quality of Life-Lite Clinical Trials Version (IWQOL-Lite-CT) Version, and Patient Global Impression of Severity (PGIS) for Overall Emotional Impact of Weight. The WES and PGIS were completed again in the second survey. Exploratory factor analysis (EFA) was used to determine the factor structure. Reliability (internal consistency and test-retest) and validity (convergent and known groups) were assessed.

### Results

The mean age (SD) of 120 study participants was 53.0 (11.8) years. The majority were female (66.7%), White (62.5%) and living with overweight or obesity for ≥11 years (63.4%). EFA supported a two-factor structure. The 16 items had strong loadings (≥0.59) on either of the two factors corresponding to positive and negative feelings about weight (inter-factor correlation 0.61). The WES subscale and total scores showed good internal consistency (Cronbach’s alpha 0.90 to 0.93), as well as good test-retest reliability from Baseline to 2-weeks (ICCs 0.87 to 0.88). The WES demonstrated convergent validity by generally showing strong correlations with IW-SP scores, IWQOL-Lite-CT Psychosocial score, and PGIS Overall Emotional Impact of weight, having smaller correlations with less similar PRO scores. Known-groups validity was supported by significantly higher WES subscale and total scores for participants that more frequently reported feeling good about their current weight overall on the PGIS Overall Emotional Impact of Weight compared to those that less frequently felt good about their current weight.

### Conclusion

These results support that the WES is a reliable and valid measure that can be used in observational studies and clinical trials for obesity.

## 2022 Adaptation of the Pediatric Quality of Life Inventory 3.0 (PedsQL) Neuromuscular Module: Lessons from Kazakhstan

Ainur Bekitayeva^1^, Shalkar Adambekov^1^, Bakhytkul Myrzaliyeva^2^

^1^al-Farabi Kazakh National University, Almaty, Kazakhstan, ^2^Expert Commission on Child Neurology at the Republican Center for Orphan Diseases, Almaty, Kazakhstan

*Journal of Patient-Reported Outcomes 2026*, **10(Suppl 1)**:2022

### Aims

The quality of life (QOL) of children with hereditary neuromuscular diseases (HNMD) in Kazakhstan has not been studied, despite approximately 600 children with these conditions living in Kazakhstan. The main objective is to translate and adapt PedsQL Neuromuscular Module for conducting a study of QOL of children with NHMD in Kazakhstan.

### Methods

The study of the QoL of children with HNMD is carried using neuromuscular module of the PedsQL 3.0 questionnaire developed by Dr. James W.Varni. After receiving permission by Mapi Research Trust to use the questionnaire, it was translated using forward translation, backward translation, cognitive interviews, and proofreading.The questionnaire was administered to parents of children of 2–18 age identified though database of registered patients with NHMD. The responses were collected through video call using WhatsApp platform given its ubiquity in Kazakhstan.

### Results

The very first method, direct translation to Kazakh required significant amount of time to ensure high quality of translation. During the implementation of the next method - reverse translation, discrepancies in the meaning from the original were identified in some issues that had to be corrected. Opinions differ in different sources of literature about the requirements in a cognitive interviewing (CI) in terms of sample size and its relationship to the topic being studied. In this case, 9 Kazakh-speaking respondents from different regions of Kazakhstan participated in the CI, since there are three main types of dialect in the Kazakh language: western, northeastern, and southern. In the CI, basic techniques were used, such as “think aloud”, “paraphrasing”, “confidence judgment”, “probing”. After receiving a positive response to the corrected and cognitively interviewed questionnaire, the next stage was of a technical nature, which required that the questionnaire be designed identically to the original English version, preserving the color scheme, font, footers, copyright links, etc.

### Conclusion

Each stage was completed, the original version of the direct translation was transformed and evolved, getting closer to the correct final version. As a result of correction after the cognitive interview, the questionnaire acquired a less formal picture and became more understandable for everyday speech.

## 2023 Psychometric properties of Dutch-Flemish PROMIS Sleep measures in Dutch childhood cancer patients receiving follow-up care

Anne Westerweel^1^, Shosha H.M. Peersmann^1^, Michiel A.J. Luijten^2^, Caroline B. Terwee^3^, Martha A. Grootenhuis^1^, Raphaële R.L. van Litsenburg^1^

^1^Princess Máxima Center for Pediatric Oncology, Utrecht, The Netherlands, ^2^Amsterdam UMC Location University of Amsterdam, Emma Children’s Hospital, Child and Adolescent Psychiatry & Psychosocial Care, Amsterdam, The Netherlands, ^3^Amsterdam Public Health Research Institute, Methodology, Amsterdam, The Netherlands

*Journal of Patient-Reported Outcomes 2026*, **10(Suppl 1)**:2023

### Aims

Sleep problems are prevalent both during and after childhood cancer, with prevalence estimates ranging from 25% to 59%. Insomnia is the most frequently reported sleep disorder, typically assessed using the Insomnia Severity Index (ISI). The Patient-Reported Outcomes Measurement Information System (PROMIS®) offers standardized, cross-population measures. This study evaluates the psychometric properties of PROMIS Pediatric Sleep Disturbance (SD) and Sleep-Related Impairment (SRI) measures in Dutch childhood cancer patients receiving follow-up care and examines the relationship between ISI-scores and PROMIS T-scores.

### Methods

Patients (n=574) completed the Dutch-Flemish PROMIS Pediatric SD v1.0 and SRI v1.0 full item banks, along with the ISI. Structural validity was evaluated using a Graded Response Model (GRM), assessing item fit statistics (S-X2, p<0.001 indicated misfit). Measurement invariance for sex was assessed through Differential Item Functioning (DIF) analysis (McFadden’s pseudo R2>0.02 indicated DIF). Reliability of item banks, extracted short forms, and simulated Computerized Adaptive Testing (CATs) was estimated based on participants with a standard error (SE) of measurement ≤0.32, indicating reliability ≥0.90. Efficiency ((1-SE(θ)2)/nitems) was computed to evaluate the performance of each measure in relation to the number of items administered. Construct validity was assessed by correlating PROMIS T-scores with ISI raw summed scores. Known-group validity was tested by comparing PROMIS T-scores across ISI-defined insomnia severity groups using ANOVA.

### Results

PROMIS SD did not meet GRM assumptions, indicating insufficient structural validity. PROMIS SRI demonstrated acceptable psychometric properties, with none of the items showing misfit or DIF. Moreover, reliability of PROMIS SRI was high (>0.90) at the mean of the sample and extended 2SD in the clinical direction. SF-4a was most efficient, followed by CATs. The mean T-score was 50.7 (SD=10.0). Correlation between PROMIS SRI and ISI was high (r=0.79). Mean PROMIS SRI T-scores increased with insomnia severity: no insomnia (M=45.6, SD=7.1), subthreshold insomnia (M=59.2, SD=5.7), clinical insomnia moderate (M=66.5, SD=4.8), and clinical insomnia severe (M=74.0, SD=3.4). T-scores differed significantly across groups (p<0.001).

### Conclusion

PROMIS Pediatric SD showed insufficient structural validity. PROMIS Pediatric SRI showed sufficient psychometric properties in Dutch childhood cancer patients receiving follow-up care. PROMIS SRI correlated well with ISI-scores and distinguished between ISI-defined insomnia severity groups.

## 2024 Real-world use of Vitaccess Real™ platform to assess quality of life impact with long-term use of Mucinex® in stable chronic bronchitis

Samuel Llewellyn^1^, Octavia Borecka^2^, Connie Divel^3^

^1^Vitaccess, Charlotte, North Carolina, UK, ^2^Vitaccess, London, North Carolina, UK, ^3^American Health Research, Charlotte, North Carolina, USA

*Journal of Patient-Reported Outcomes 2026*, **10(Suppl 1)**:2024

### Aims

Chronic bronchitis is a respiratory condition characterized by long-term, persistent, productive cough. Guaifenesin is an over-the-counter expectorant used to manage respiratory symptoms and thin mucus, though its potential long-term benefits for stable chronic bronchitis (SCB) are not well studied. The aim of this study was to describe real-world use and effectiveness of Mucinex® (extended-release guaifenesin) in patients with SCB. This analysis comprises engagement data and key patient-reported outcomes.

### Methods

This was an open-label, multicenter, single-cohort study of US adults 40 years or older with SCB. Following a two-week run-in, participants took extended-release Mucinex® (1,200 mg twice daily; 2,400 mg/day) for 12 weeks.Participants used the Vitaccess Real™ platform to complete the Cough and Sputum Assessment Questionnaire (CASA-Q) weekly from baseline, and a selection of survey questions about treatment compliance, satisfaction, and symptoms weekly during the treatment period. Concurrently, healthcare professionals completed an electronic case report form.Several measures were employed to encourage survey completion. Participants could complete surveys on their own devices at their convenience. Questions were selected to balance data relevance and completeness against participant burden. Participants could opt in to email and SMS notifications about survey availability and outstanding survey completions. Participants received a voucher for each weekly survey completed, plus an end-of-study voucher for 100% completion.

### Results

The study enrolled 82 participants, of which 75 were included in the final analysis. Mean age was 66 years. 55% were female and 91% were White.Each week, >92% of participants completed surveys, with 61% completing all surveys.Over 90% of participants reported compliance with Mucinex® each treatment week. At all treatment assessment points, >90% reported that Mucinex® was convenient to take, and >70% reported being extremely or somewhat satisfied with its ability to relieve SCB symptoms. During each treatment week, >85% reported no worsening of SCB symptoms.

### Conclusion

This analysis demonstrates strong engagement with the Vitaccess Real™ platform, plus high satisfaction and compliance with Mucinex® for alleviation of SCB symptoms, supporting the role of long-term expectorant therapy in SCB and the ability of the Vitaccess Real™ platform to measure patient-reported outcomes. Analyses of CASA-Q data are ongoing.

## 2025 Impact of transfusion dependent alpha- and beta-thalassemia on adult patients’ health-related quality of life and work productivity: A multi-region real-world survey

Janet Kwiatkowski^1^, Khaled Musallam^2^, Maria Domenica Cappellini^3^, Christina Chamberlain^4^, Amey Rane^4^, Keely Gilroy^4^, Susan Morris^4^, Emma Chatterton^5^, Brianne Kerr^5^, Katie Lewis^5^, Ali Taher^6^

^1^Children’s Hospital of Philadelphia, Philadelphia, Pennsylvania, USA, ^2^Burjeel Medical City, Abu Dhabi, United Arab Emirates, ^3^Fondazione IRCCS Ca’ Granda Ospedale Maggiore Policlinico, Milan, Italy, ^4^Agios Pharmaceuticals, Cambridge, Massachusetts, USA, ^5^Adelphi Real World, Bollington, UK, ^6^American University of Beirut Medical Centre, Beirut, Lebanon

*Journal of Patient-Reported Outcomes 2026*, **10(Suppl 1)**:2025

### Aims

Research on the impact of transfusion-dependent thalassemia (TDT) on health-related quality of life (HRQoL) and work productivity is limited, particularly in α-TDT. The aim of this analysis was to investigate HRQoL and work productivity of patients with α- or β-TDT globally.

### Methods

Data were drawn from the Adelphi Real World Thalassemia Disease Specific Programme™, a cross-sectional survey of physicians and their patients (≥18 years) with α- or β-TDT. Data were collected from February ─ November 2024 in Brazil, Egypt, France, Germany, Greece, Italy, Malaysia, Saudi Arabia, Spain, Thailand, Turkey, the United Arab Emirates, and the United States.Physicians reported clinical characteristics, and a subset of corresponding patients completed a voluntary survey that captured demographics and data from the Functional Assessment of Chronic Illness Therapy (FACIT)-Fatigue, Patient-Reported Outcomes Measurement Information System (PROMIS) Physical Function (PF) and Work Productivity and Activity Impairment (WPAI)-Thalassemia questionnaires. Patients with a history of gene therapy or hematopoietic stem cell transplantation were excluded.Data were summarized descriptively by genotype, age, and gender.

### Results

Data from 223 patients with TDT (32 α-TDT; 191 β-TDT) were analyzed. Mean age (standard deviation [SD]) was 34.5 (13.2) years (α-TDT: 38.7 [17.9] years; β-TDT: 33.8 [12.2] years), 55.2% were female (α-TDT: 53.1%; β-TDT: 55.5%), and 49.7% were working full/part time (α-TDT: 46.9%; β-TDT: 50.3%). Mean (SD) FACIT-Fatigue scores were 26.2 (11.1) for α-TDT and 29.5 (11.7) for β-TDT compared with published US norm of 43.6 (9.4). Mean (SD) PROMIS PF T-scores were 40.3 (6.6) for α-TDT and 43.0 (7.5) for β-TDT compared with published US norm of 59.7 (8.0).Mean (SD) WPAI-Thalassemia overall work impairment scores were 58.8% (20.8%) for α-TDT and 41.3% (26.8%) for β-TDT, and activity impairment scores were 56.7% (26.7%) and 41.2% (26.2%), respectively. Published US norms are 15.01% (25.96%) for overall work impairment and 22.08% (28.33%) for activity impairment.Results relative to US norms were aligned across age and gender subgroups (Table).

### Conclusion

This multi-regional study included real-world data on humanistic burden from patients with α-TDT and β-TDT. Across genotypes, adults with TDT experience worse fatigue and greater physical function, work productivity, and daily activity impairments than the general US population.

**Table 1 (abstract 2025):**
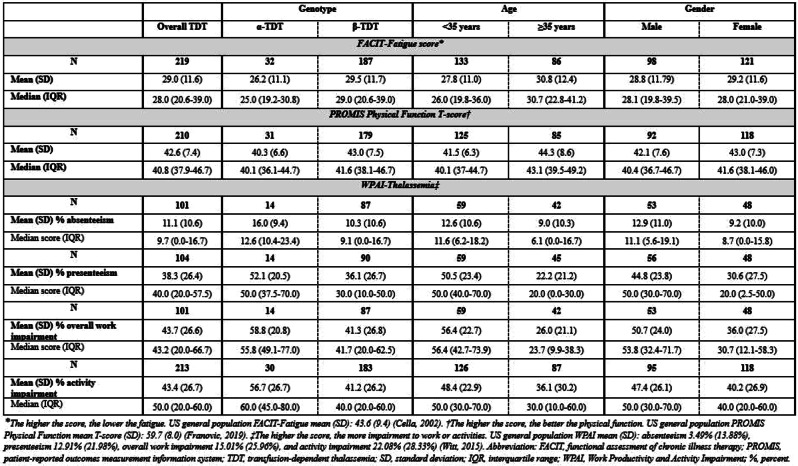
Impact of transfusion dependent alpha- and beta-thalassemia on adult patients’ health-related quality of life and work productivity: A multi-region real-world survey

## 2026 A comprehensive literature review of definitions and methods for assessing saturation of qualitative data

Miranda Lauher-Charest^1^, Kristi Jackson^1^, Cory Saucier^1^

^1^IQVIA Quality Metric, INC., Providence, Rhode Island, USA

*Journal of Patient-Reported Outcomes 2026*, **10(Suppl 1)**:2026

### Aims

Saturation, broadly defined as the point where additional data do not produce new relevant themes, is used as an indicator for stopping qualitative data collection. It originated with grounded theory but has since evolved. The objective of this review was to identify and evaluate existing definitions and methods for assessing saturation of qualitative data.

### Methods

Two PubMed search strings were developed: one focused on saturation in qualitative research, and another more broadly capturing qualitative methodology. The selection criteria were: 1) the article was in English, 2) the article was published between 2013 and 2023, and 3) the primary focus was qualitative methodology. This was supplemented by hand-searching relevant article reference lists and reviewing select FDA, ISPOR, and ISOQOL qualitative research guidance. After screening, full texts were retrieved if the record 1) met selection criteria, 2) presented insufficient information to decide, or 3) was a review article. Articles considered relevant were advanced to the next round for full-text review (Figure 1).

### Results

Twenty-eight records were included. Twenty-five defined the term “saturation,” 5 with original definitions and 20 citing at least one source, most commonly Glaser and Strauss (1967), Guest et al., (2006), and Morse (1995; 2015). Fifteen further identified different versions of saturation: theoretical, thematic, meaning, a priori thematic, code, and data. Of these, 3 were inductive and 3 were deductive. Two versions primarily focused on data novelty, two on data redundancy, and two had no primary focus (Table 1). Sixteen articles described strategies for assessing saturation, each of which aligned to 1 of 6 methodologies—stopping criterion, comparative method, code meaning, code frequency counts, high order grouping, and statistical modeling—under 1 of 3 typologies: sample focused, analysis focused, and frequency focused. Two described saturation alternatives: information power, and theoretical sufficiency (Table 2).

### Conclusion

This review identified and evaluated 6 definitions and 6 methods for assessing saturation of qualitative data. These results offer a central resource for researchers to better understand saturation and its evolution. Future work should offer guidance to researchers on selecting a definition and method of saturation for their research.

**Fig. 1 (abstract 2026):**
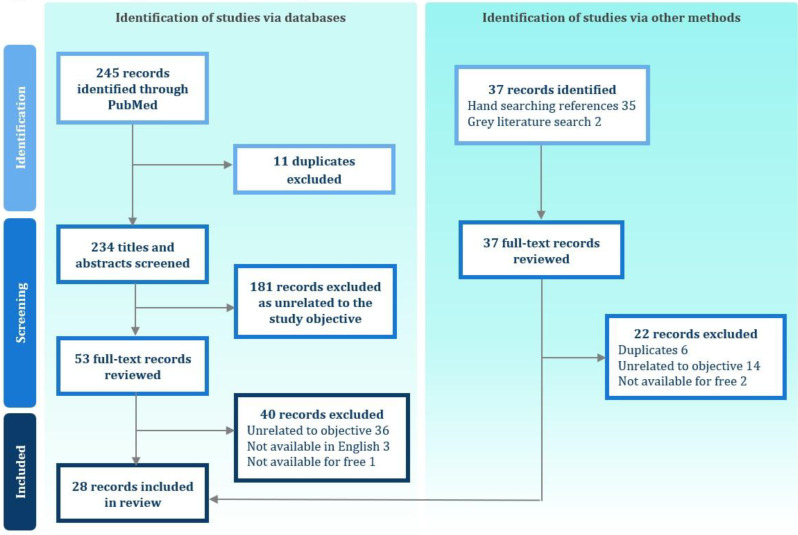
Screening Process PRISMA Diagram

**Table 1 (abstract 2026):**
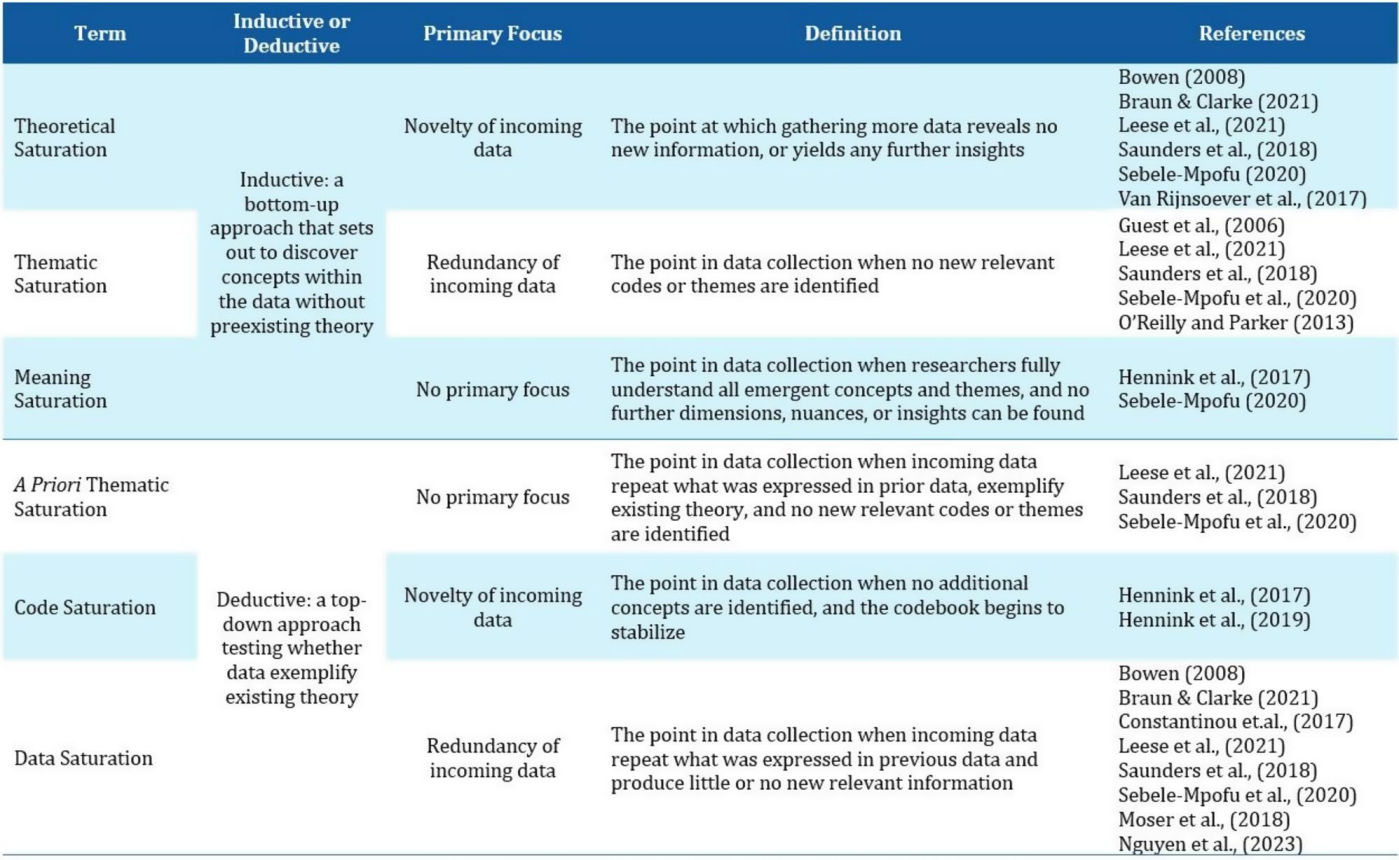
Versions of Saturation

**Table 2 (abstract 2026):**
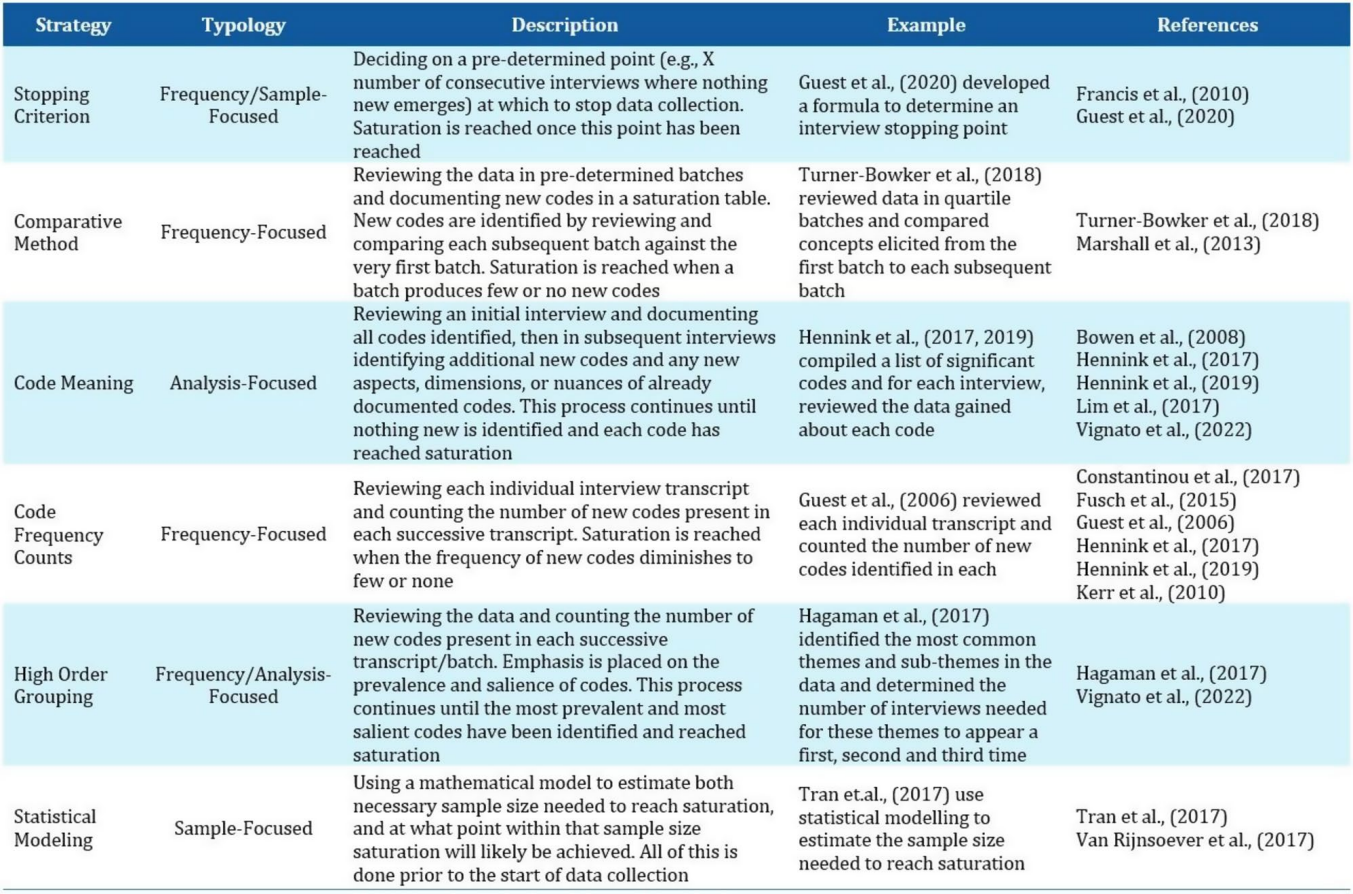
Strategies for Assessing Saturation

## 2027 Capturing the impact of Facioscapulohumeral Muscular Dystrophy (FSHD) on Health-related Quality of Life (HRQoL): a systematic review of the reliability and validity of existing self-report instruments using COSMIN

Jill Carlton^1^, Philip Powell^1^, Sé Frances^1^, Claire Williams^1^, Anthea Sutton^1^, Jonathan Street^1^, Channa Hewamadduma^1^, Jill Carlton^1^

^1^University of Sheffield, Sheffield, UK

*Journal of Patient-Reported Outcomes 2026*, **10(Suppl 1)**:2027

### Aims

Facioscapulohumeral muscular dystrophy (FSHD) is a rare genetic muscle wasting condition causing progressive weakness. Its progression varies between individuals, and typically develops asymmetrically. Studies have explored the impact of FSHD on health-related quality of life (HRQoL) using a plethora of patient reported outcome measures (PROMs). Given that a number of generic and condition-specific questionnaires are available for use in attempting to assess HRQoL in people with FSHD, evidence is desperately needed on the relative validity and psychometric performance of these instruments, when it comes to assessing HRQoL in FSHD. This review aims to: 1) identify which PROMs have been used to assess HRQoL (or an aspect thereof) in FSHD; and 2) evaluate the strength and quality of evidence for the reliability and validity of the PROMs identified.

### Methods

This is a systematic review employing COSMIN methodology. The project follows four stages. Stage 1: Searches are conducted following COSMIN guidance, and will cover multiple databases, including MEDLINE (PudMed), EMBASE, CINAHL, PsycINFO, and The Cochrane Library using a two-stage strategy to identify 1) articles containing PROMs assessing HRQoL in FSHD; and 2) articles reporting the measurement properties of these PROMs. Follow-up searches via citation tracking and Google Scholar will complement the structured search. Stage 2: Following a two-stage sifting strategy against pre-specified criteria, data on reliability and validity are extracted from each included article. Stage 3: COSMIN methodology is applied for each measurement property, as well as the assessment of the methodological quality of each included article. Stage 4: Results are summarised qualitatively.

### Results

The review is ongoing. Preliminary searches have been completed (Oct 2024 and Jan 2025), with follow-up searches ongoing. Initial searching identified 496 articles, with n=193 reviewed at full-text, and n=49 retained. 44 potential PROMs were identified and taken forward to Stage 2. Further results will be presented at the conference.

### Conclusion

FSHD is a complex, devastating condition. There are a number of PROMs that could be used to assess the impact of FSHD on HRQoL. This review will provide a recommendation for the most suitable PROM(s) to consider for use in quantifying the impact of HRQoL on individuals.

## 2028 Translation and linguistic validation of the GENDER-Q into French for use in Canada

Trisia Breitkopf^1^, Sylvie Cornacchi^1^, Ines Ndzana Siani^1^, Julia Sapin-Leduc^2^, Flavie Guibert-Piché^2^, Hanya Obsieh^1^, Charlene Rae^1^, Yi Wang^1^, Alexis Laungani^2^, Pierre Brassard^2^, Manraj Kaur^3^, Andrea Pusic^3^, Anne Klassen^1^

^1^Department of Pediatrics, McMaster University, Hamilton, Ontario, Canada, ^2^GrS Montréal, Montréal, Quebec, Canada, ^3^Patient-Reported Outcomes and Values, & Experience Center (PROVE), Brigham and Women’s Hospital, Harvard Medical School, Boston, USA

*Journal of Patient-Reported Outcomes 2026*, **10(Suppl 1)**:2028

### Aims

The GENDER-Q is a new patient-reported outcome measure (PROM) that measures outcomes in gender-affirming care. The aim of the study was to translate the GENDER-Q into a linguistically validated Canadian French version for use in research and clinical care.

### Methods

We followed methods according to the Professional Society for Health Economics and Outcomes Research (ISPOR) guidelines for PROM translation and cultural adaptation. This involved two forward translations, reconciliation, back-translation and review, and debriefing interviews with transgender and gender diverse (TGD) patients.

### Results

The field-test version of the GENDER-Q, consisting of 55 scales (959 items), was translated by two independent translators whose native language was Canadian French. The scales were back-translated from Canadian French to English and then compared to the original English version to identify discrepancies in conceptual equivalence. The instructions for 13 scales, 7 response options, and 50 phrases (applied to 96 items across scales) required discussion and re-translation. Cognitive debriefing interviews of the French translation version were then conducted with 10 TGD individuals (20 – 62 years of age, n=5 transmale, n=4 transfemale, n=1 gender fluid). Participants found the GENDER-Q scales to be understandable and acceptable. Participant feedback led to further re-wording of 8 instructions to either clarify their meaning or to revise the definitions of various terms (e.g., “donor area” for phalloplasty) and the refinement of 30 phrases (applied to 83 items across scales). The translation was evaluated for consistency in the wording of recurring phrases, instructions, and response options across the different scales. Special attention was paid to ensure that TGD terminology was translated and defined appropriately (e.g., binding, tucking). The translation process led to a linguistically validated and conceptually equivalent Canadian French version of the GENDER-Q. Having a Canadian French version made it possible for us to include an additional 109 participants in the GENDER-Q international field-test study (total sample was 5,497).

### Conclusion

The GENDER-Q was translated and linguistically validated in Canadian French and can be used to inform gender-affirming patient care, clinical research, quality improvement, and regulatory efforts.

## 2030 Validation of a two-item version of the Perceived Health Competence Scale

Jacquelyn Pennings^1^, Devika Nair^1^, Hayden B. Bosworth^2^, Kenneth E. Freedland^3^, Sunil Kripalani^1^, Elisa J. Gordon^1^, Gurjeet S. Birdee^1^, Justin M. Bachmann^1^

^1^Vanderbilt University Medical Center, Nashville, Tennessee, USA, ^2^Duke University Medical Center, Durham, North Carolina, USA, ^3^Washington University School of Medicine, St. Louis, Missouri, USA

*Journal of Patient-Reported Outcomes 2026*, **10(Suppl 1)**:2030

### Aims

Perceived health competence is a construct encompassing individuals’ confidence in managing health-related tasks or behaviors. The original 8-item Perceived Health Competence Scale (PHCS-8) has established psychometric properties. We sought to validate the 2-item Perceived Health Competence Scale (PHCS-2), an abbreviated version of the PHCS-8, for use in time-constrained clinical and research settings.

### Methods

We used pooled data comprising 482 participants from two cohort studies conducted in an integrative medicine clinic at one institution. Participants completed the PHCS-8, sociodemographic information, the Patient-Reported Outcomes Measurement Information System (PROMIS) Global-10 measure of physical and mental health, and a social desirability scale (the SDRS-5). Psychometric evaluation included internal consistency reliability, factorial validity, Bland-Altman analysis of instrument agreement, convergent validity through correlation analysis, and known-groups validity across and health status subgroups.

### Results

The sample had a mean age of 50.8 years (SD=13.2) and was 81% female. Participants were highly educated, with 86% having attained at least a bachelor’s degree. Forty-four percent reported annual incomes of $75,000 or greater. The two PHCS-2 items demonstrated appropriate response distributions across the 5-point Likert scale. The most common response was “disagree” (48.8%) with “It is difficult for me to find effective solutions to the health problems that come my way” and “agree” (48.1%) with “I am able to do things for my health as well as most other people” (Table 1). The PHCS-2 demonstrated strong correlation with PHCS-8 (r=0.798; Table 2). Bland-Altman analysis revealed minimal systematic bias (mean difference=0.032), with 63% of participants having PHCS-2 and PHCS-8 scores within 0.25 points (Figure 1). The PHCS-2 exhibited moderate correlations with PROMIS physical health (r=0.463) and mental health (r=0.391) status (Table 2). The instrument was not significantly correlated with social desirability response bias and there were no significant differences in PHCS-2 scores by sex, education, or income and only a weak correlation with age (r=0.122).

### Conclusion

The PHCS-2 represents a valid, brief measure of perceived health competence that maintains strong concordance with the full PHCS-8 while reducing respondent burden. Clinicians can use the PHCS-2 tool to identify patients who might benefit from interventions designed to improve health self-management skills.

**Table 1 (abstract 2030):**
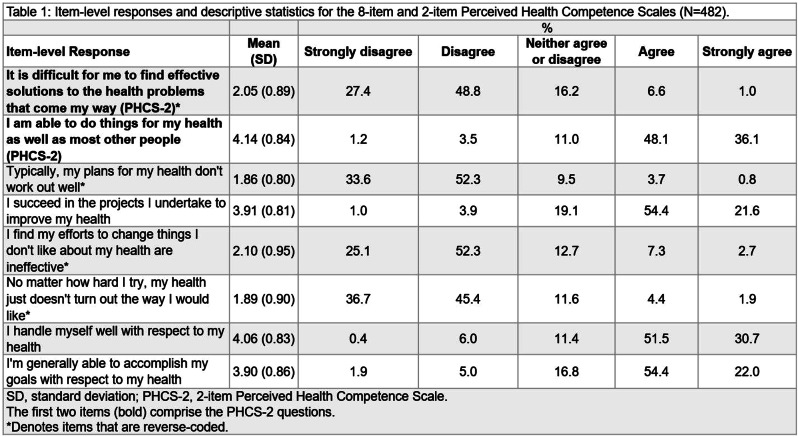
Item-level responses and descriptive statistics for the 8-item and 2-item Perceived Health Competence Scales (N = 482)

**Table 2 (abstract 2030):**
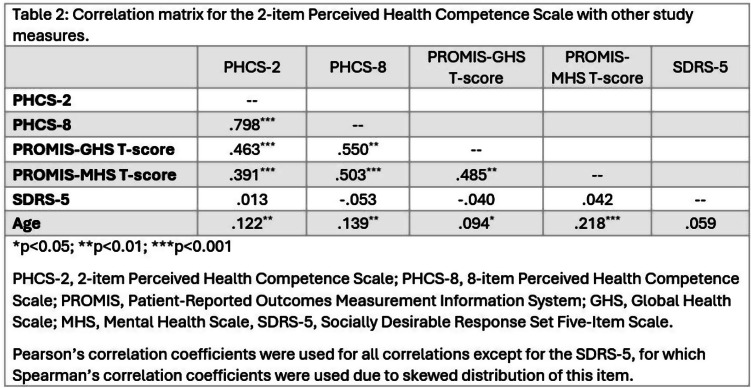
Correlation matrix for the 2-item Perceived Health Competence Scale with other study measures

**Fig. 1 (abstract 2030):**
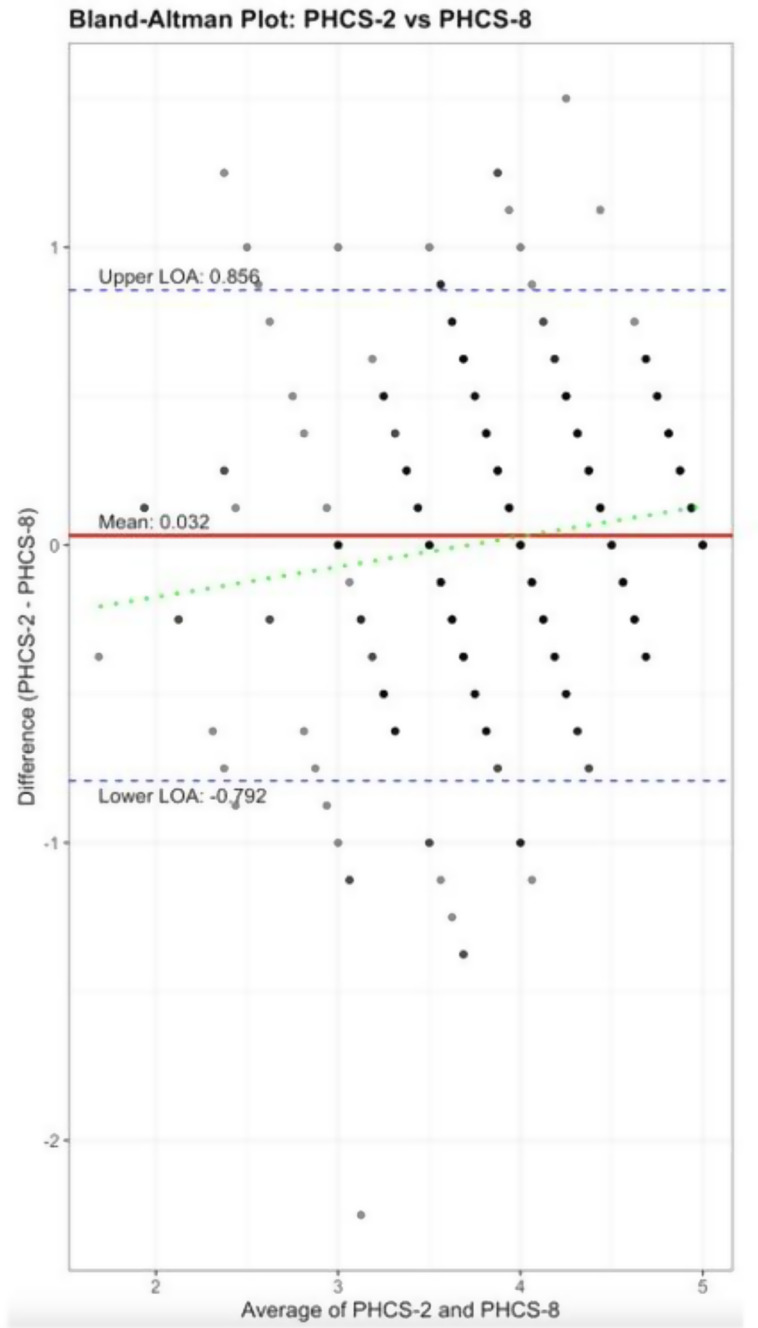
Bland-Altman Plot Comparing Perceived Health Competence Scale 2-item (PHCS-2) and 8-item (PHCS-8) Measurements

## 2031 Cognitive Assessment in Schizophrenia: A Landscape Review of Instruments and Core Domains

Angela Stroupe^1^, Chris Buckley^2^, Alessandra Girardi^3^, Zorana Zupan^4^

^1^Parexel, Boston, Massachusetts, USA, ^2^Parexel, London, UK, ^3^Parexel, Rome, Italy, ^4^Parexel, Belgrade, Serbia

*Journal of Patient-Reported Outcomes 2026*, **10(Suppl 1)**:2031

### Aims

Cognitive impairment, affecting up to 45% of schizophrenia patients, remains the least addressed core symptom. The MATRICS Battery, developed for schizophrenia clinical trials, includes 10 paper-and-pencil tests across various cognitive domains. However, it is lengthy and complex, requiring extensive rater training. Some MATRICS tests contribute less to the composite score, suggesting a need for shorter or computerized alternatives. This review explored alternative cognitive assessment tools in Phase II and III schizophrenia trials and examined which core cognitive domains were included.

### Methods

A systematic search was conducted on ClinicalTrials.gov on January 18, 2024. After excluding one withdrawn study, 27 Phase II and III trials conducted by pharmaceutical companies that assessed cognition in schizophrenia were analyzed. Information regarding instruments and the cognitive domains was identified and extracted.

### Results

The majority of trials (77.8%, N=21) incorporated the MATRICS battery. Of these, 52.4% (N=11) exclusively used MATRICS, while the remainder also included other cognitive batteries or their subdomains both computerized such as Cogstate (22.2%, N=6), and CANTAB (7.4%, N=2) as well as paper and pencil such as BACS (18.5%, N=5), Six trials (22.2%) either used Cogstate (7.4%, N=2), BACS (7.4%, N=2), or a combination of both (7.4%, N=2) instead of MATRICS. Most trials (N = 18/21) assessed all MATRICS domains to establish a composite cognitive function score. Further exploratory analysis beyond the composite score showed that performance on individual domains was often reported for all domains, particularly reasoning/problem solving (N =15) and speed of processing (N = 13). Fewer trials explored performance on the individual social cognition domains (N = 9). A minority of trials included additional non-core such as motor speed and set shifting (both 7.4%, N=2).

### Conclusion

The findings indicate an increasing trend towards using computerized batteries or individual tasks in schizophrenia clinical trials, either as supplementary or in place of the MATRICS battery. Further research is needed to establish clear equivalency standards and improve consistency across alternative cognitive assessments implemented in schizophrenia trials. Additionally, it is important to identify which tests best contribute to the composite cognitive score and relate to daily functioning, as this is relevant for various stakeholders.

## 2032 Assessing the content validity of the Familial Chylomicronemia Syndrome Symptoms and Impacts scale (FCS-SIS) in severe hypertriglyceridemia (sHTG)

Asia Sikora Kessler^1^, T. Michelle Brown^2^, Bonita Basnyat^2^, Jennifer Dine^2^

^1^Ionis Pharmaceuticals, Carlsbad, California, USA, ^2^RTI Health Solutions, Research Triangle Park, North Carolina, USA

*Journal of Patient-Reported Outcomes 2026*, **10(Suppl 1)**:2032

### Aims

Severe hypertriglyceridemia (sHTG) and familial chylomicronemia syndrome (FCS) are characterized by high triglyceride levels associated with negative impacts on physical, emotional, social, and cognitive function. The FCS-Symptom Impact Scale (FCS-SIS) was developed to assess common symptoms and impacts of FCS. This study aimed to evaluate the appropriateness of the FCS-SIS symptom items in adults with sHTG.

### Methods

Two iterative rounds of qualitative interviews were conducted with adults who had clinician-confirmed sHTG and recently experienced ≥ 2 symptoms of sHTG. Interviews included concept elicitation and cognitive debriefing of the FCS-SIS four symptom items. Key themes were identified from participant feedback; item relevance, interpretability, answerability, and meaningful change were assessed.

### Results

Twenty participants were interviewed (n = 10 per round). The most frequently reported symptoms included difficulty thinking, physical fatigue (n = 20, each), diarrhea (n = 19), and abdominal pain (n = 18), which comprise the FCS-SIS symptoms. These symptoms were also most frequently reported as most bothersome and important to improve.All participants reported 1 or more symptoms included in the FCS-SIS within the 7-day recall period, understood the items, and stated that at least one improvement would be meaningful. Many participants (n = 11) found the items comprehensive of their sHTG experience; 9 suggested additional symptoms. Most participants (n = 17) deemed the items relevant with 3 unclear with the relevance of a specific item, and 19 of 20 said the items were representative of their sHTG.

### Conclusion

The study supported the content validity of the FCS-SIS symptom items in adults with sHTG. The symptoms evaluated in the items comprised the most frequently reported, bothersome, and important to treat. Participants understood and answered the items appropriately and noted that improvement in these symptoms would be meaningful. Nearly all participants found the 4 symptoms of the FCS-SIS as representative of their symptom experience with sHTG. Results suggest the FCS-SIS symptom items are suitable for assessing symptoms in adults with sHTG.

## 2033 Subgroup or principal stratum analysis? A comparison and guidance for use of the principal stratum framework for COA endpoints

Konstantina Skaltsa^1^, Paolo Eusebi^2^, Michael Schlichting^3^, Pavol Kral^4^

^1^IQVIA, Barcelona, Spain, ^2^IQVIA, Rome, Italy, ^3^Merck KGaA, Frankfurt, Germany, ^4^IQVIA, Bratislava, Slovakia

*Journal of Patient-Reported Outcomes 2026*, **10(Suppl 1)**:2033

### Aims

It is common for researchers to seek to understand the treatment effect of an intervention in certain subsets of participants of a clinical trial. These subsets may be defined by baseline characteristics, e.g., patients with certain biomarkers, age groups etc., or by post-baseline events, e.g., patients who survived till a certain timepoint, or who responded based on a success criterion. Although it is common to repeat the main analysis on subsets of the randomized population, this is not appropriate when subsets are defined based on post-baseline events. This talk will focus on this case.

### Methods

We will present the principal stratum (PS) framework for estimating the treatment effect in subsets of patients who would potentially experience a post-baseline event under either treatment arm. The PS framework has become more known in drug development since its inclusion as one of the five strategies for handling intercurrent events in the estimand framework (ICH E9(R1) guideline 2020) and the availability of programming macros. We will explain how a PS differs from a subgroup and when each should be considered. We will use an oncology trial dataset and estimate the treatment effect in subsets of the randomized population using the PS and subgroup analysis. Examples of populations of interest may be patients who would achieve clinical response, would survive, would adhere to treatment, would not require a treatment interruption or holiday in case of toxicity or success, respectively, or would/would not go to surgery.

### Results

We will present two examples of post-baseline events for illustration. We will contrast the estimates from PS and subgroup analysis on patients who experienced the post-baseline event in the arm to which they were randomized, and explain why the latter may be biased.

### Conclusion

Although less common, the PS framework is the appropriate method for estimating causal treatment effects when subsets of patients are defined by post-baseline events, while naïve subgroup analysis is, in most cases, biased. Acknowledging the assumptions and potential limitations of the framework, we encourage researchers and statisticians to utilize it and decision makers to be sensitive when subgroup analyses on post-baseline factors are presented for causal inference purposes.

## 2034 Evaluating the impact of missing data on scale reliability: A simulation study of a 10-Item HRQOL COA with varying item properties

Adrian Jewett^1^

^1^Lumanity, Los Angeles, California, USA

*Journal of Patient-Reported Outcomes 2026*, **10(Suppl 1)**:2034

### Aims

Many COAs allow up to 50% of items to be missing, assuming that all items are interchangeable, which is rarely the case. Items in scale development can be classified as parallel, tau-equivalent, or congeneric. Parallel items assume equal factor loadings and error. Tau-equivalent items assume equal factor loadings but with varying errors. Congeneric items assume varied factor loadings and errors, making them the most common.This study uses a simulation-based approach to evaluate missingness in varying item properties and the dangers of accepting item-level missingness without comprehensively assessing the impacts on reliability.

### Methods

A 10-item COA was simulated under parallel, tau-equivalent, and congeneric conditions with an observed Alpha = 0.86 and 10,000 observations in each condition. The parallel condition specified the same factor loading (0.67) and item thresholds. The tau-equivalent condition specified the same factor loading (0.67) but varying thresholds. The congeneric condition specified items with high factor loadings (0.86 to 0.94), low loadings (0.42 to 0.50), and varying thresholds.The Spearman-Brown formula was used to estimate reliability for reductions in scale length, assuming item interchangeability. Alpha was also calculated for every simulated combination of missing items, and descriptive summaries of reliability were provided under different conditions.

### Results

For parallel and tau-equivalent conditions, reliability remained consistent for any combination of missing items at each level. For the congeneric condition, estimates varied depending on the items, influenced by factor loadings. The Spearman-Brown prediction matched the average alpha for all conditions at each level of missingness. The SD was zero for parallel and tau-equivalent conditions, indicating consistent reliability. In the congeneric condition, SD increased with missingness, showing variability and some combinations resulted in Alphas below 0.70. Missing items with high factor loadings resulted in the lowest alpha estimates.

### Conclusion

This study shows that the evaluation of missingness on reliability depends on item properties. For parallel and tau-equivalent items, the Spearman-Brown formula accurately estimates reliability based on reductions to scale length. However, for congeneric items, reliability depends on how many and which items are missing, highlighting the risk of assuming item interchangeability. Comprehensive evaluation of simulated missing item combinations is crucial to ensure reliable measurement.

**Table 1 (abstract 2034):**
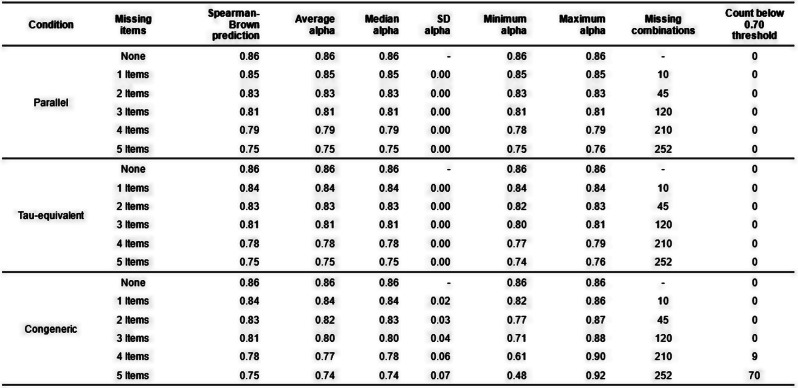
Results of the Spearman-Brown Prediction formula and simulated item missingness across three conditions

**Table 2 (abstract 2034):**
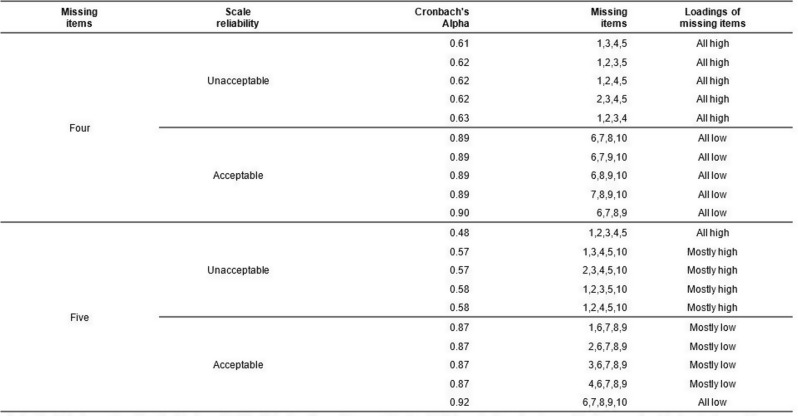
Detailed assessment of the five highest and lowest reliability estimates from the congeneric condition at the four and five missing item level

## 2035 Mapping Treatment-Induced Adverse Events against HRQoL Questionnaires in Non-Small Cell Lung Cancer: Evaluation of the EORTC QLQ-C30 and QLQ-LC29

Lotte Van der Weijst^1^, Cecilia Pompili^2,3^, Maria Teixeira^4^, Hayat Hamzeh^5^, Claire Piccinin^4^, Madeline Pe^4^

^1^EORTC, Brussels, Belgium, ^2^Institute for Clinical & Applied Health Research, University of Hull, UK, ^3^Section of Patient Centred Outcomes Research, University of Leeds, UK, ^4^Quality of Life Department, European Organisation for Research and Treatment of Cancer, Brussels, Belgium, ^5^Leeds Institute of Medical Research, University of Leeds, UK

*Journal of Patient-Reported Outcomes 2026*, **10(Suppl 1)**:2035

### Aims

This study aims to evaluate the relevance of health-related quality of life (HRQoL) questionnaires in capturing treatment-induced adverse events. This study investigates whether the patient-reported European Organisation for Research and Treatment of Cancer (EORTC) quality of life core questionnaire (QLQ-C30) and the associated lung cancer module (QLQ-LC29) comprehensively capture clinician-reported adverse events reported in European Medicines Agency (EMA) associated with standard systemic treatments for non-small cell lung cancer (NSCLC).

### Methods

Inclusion criteria encompassed standard systemic monotherapy for NSCLC, based on the European Society for Medical Oncology (ESMO) guidelines. Data on adverse events were sourced from the product information of the EMA. Treatments that were exclusively documented in the context of combined modalities were excluded. For therapies with multiple EMA authorizations and discrepancies in reported adverse events, a consolidated list of adverse events was compiled. The reported very common and common adverse events were compared against the symptom scales of the EORTC QLQ-C30/LC29 by using the EORTC Common Terminology Criteria for Adverse Events (CTCAE) mapping framework.

### Results

In total, 31 systemic treatments were included in the study (22 targeted therapies, 6 immunotherapies and 3 chemotherapies). Several chemotherapies, particularly platinum-based chemotherapies, were excluded due to insufficient EMA product information. The most frequently reported QLQ-C30/LC29 symptoms scales in the EMA product information included diarrhea (n=31; 100%), pain (n=29; 93.5%), skin problems (n=28; 90.3%), nausea and vomiting (n=28; 90.3%), fatigue (n=23; 74.2%), constipation (n=20; 64.5%), and cough (n=20; 64.5%). The financial difficulties scale of the QLQ-C30 and the surgery-related symptoms and fear of progression QLQ-LC29 scales were not reported as adverse events. See Table 1 for an overview of the mapping of EORTC QLQ-C30/LC29 symptoms to adverse events per treatment modality.

### Conclusion

The results demonstrate that (very) common clinician-reported adverse events associated with systemic treatments for NSCLC are included in the EORTC QLQ-C30/LC29 questionnaires. For symptomatic adverse events not covered in the QLQ-C30/LC29, item lists can be created. Certain scales, such as socio-economic and psychological well-being, are not covered by clinician-reported adverse events but are important HRQoL domains. Future research should consider multi-modality treatment implications, including surgery and radiotherapy.

**Table 1 (abstract 2035):**
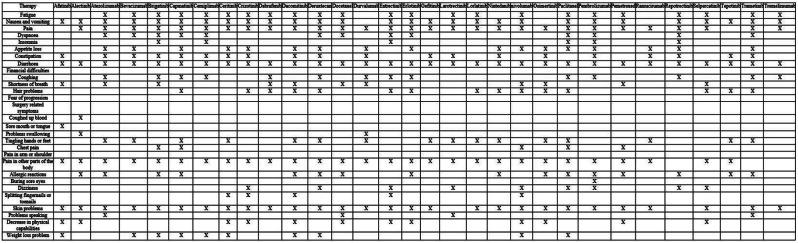
Overview of the mapping of EORTC QLQ-C30 and LC29 symptoms to adverse events per treatment modality

## 2036 Developing a Dutch PROPr value set: study design and lessons learned

Ellen Elsman^1^, Michiel Luijten^2^, Judith Bosmans^3^, Caroline Terwee^2^, Janel Hanmer^4^, Benjamin Schalet^2^

^1^Department of Child and Adolescent Psychiatry and Psychosocial Care, Amsterdam UMC, Emma Children’s Hospital, Amsterdam, Netherlands, ^2^Amsterdam UMC, Department of Epidemiology & Data Science, Amsterdam, Netherlands, ^3^Vrije Universiteit Amsterdam, Department of Health Sciences, Amsterdam, Netherlands, ^4^University of Pittsburgh, Division of General Internal Medicine, Pittsburgh, Pennsylvania, USA

*Journal of Patient-Reported Outcomes 2026*, **10(Suppl 1)**:2036

### Aims

Preference-based quality of life instruments are used to estimate Quality-Adjusted Life-Years. The PROMIS-Preference measure (PROPr) is a preference-based summary score developed in the US. We aim to develop a Dutch PROPr value set reflecting the Dutch population. We present our study design and insights gained from adopting the US blueprint to the Netherlands.

### Methods

We plan to replicate US-based PROPr value set development. We reviewed the literature, PROPr technical reports, and PROMIS item properties. We consulted with US-based PROPr developers to clarify and optimize the procedures. We conducted a pre-pilot with three independent research groups from the Amsterdam UMC to assess the comprehension of the standard gamble valuation method (SG).

### Results

We adopted the seven PROPr health domains based on US research and assumed their relevance for the Dutch population. The SG uses two PROMIS items per domain; we found that one item (“I felt unhappy”) exhibited English-Dutch differential item functioning (DIF) in a previous study. We replaced it with “I felt sad”, which has similar IRT parameters. We plan to administer SG online, as conducted in the US. We obtained pre-pilot feedback on our SG from researchers in Risk Communication (n=1), Epidemiology & Data Science (n=11) and Pediatric Psychology & PROMs (n=15), resulting in adaptations to the instructions. We considered a visual presentation (vs numeric presentation) of the “gamble,” but rejected it due to equivocal findings in the literature, and the risk of incomparability with US-based PROPr. Given the potential for participants to misunderstand SG, we will conduct a pilot study (n=20–30) using think-aloud methods to refine the presentation and instructions. Pilot study results will be presented at ISOQOL.

### Conclusion

Adaptations from the US blueprint include addressing DIF, integrating a pilot study, and modifying instructions. Following pilot testing, we will conduct the SG valuation study using a representative sample of the Dutch general population (n=1300) recruited via an internet panel. Additionally, we will assess the construct validity of PROPr utilities in geriatric long-term home healthcare and compare PROPr scores with the EQ-5D-5L. The Dutch PROPr value set will enhance cost-effectiveness analyses across diverse medical fields using a single PROMIS PROFILE measure.

## 2037 Physical functioning measures by PROs on MDASI-Lung and 6-minute walk test during first year after chemoradiation and immunotherapy for NSCLC

Shu-En Shen^1^, Xin Shelley Wang^1^, Ting Xu^1^, Rachel Maguire^1^, Diana Amaya^1^, Aileen Chen^1^, David Qian^1^, Steven Lin^1^, Anita Deswal^1^, Efstratios Koutroumpakis^1^, Anne Tsao^1^, Mehmet Altan^1^, Mei Chen^1^, Xiaodong Zhang^1^, Ruitao Lin^1^, Jane Pearce^1^, Zhongxing Liao^1^

^1^The University of Texas MD Anderson Cancer Center, Houston, USA

*Journal of Patient-Reported Outcomes 2026*, **10(Suppl 1)**:2037

### Aims

Patients with non-small cell lung cancer (NSCLC) may experience significant physical functioning impairment relevant to both disease and toxicities related to concurrent chemoradiation therapy (CRT) and immunotherapy during the first year of survivorship. The goal of this study is to verify the value of a subjective measure on physical functioning against the 6-minute walk test (6MW) as an objective performance measure for use in future clinical care and trials.

### Methods

Patients were enrolled on a prospective trial (NCT05010109) to perform longitudinal assessment of patient-reported outcomes (PRO) using the lung module of the MD Anderson Symptom Inventory (MDASI-L) and 6MW up to 12 months after CRT. In addition to MDASI-L items, WAW, an activity-related physical functioning interference subscale of work, general activity, and walking, was also used. Mixed modeling was used to examine the longitudinal relationship between the MDASI-L and 6MW distance.

### Results

Sixty-five patients who contributed both MDASI-Lung and 6MW data were included. The mean walking distance (meters) on 6MW decreased from baseline (mean (std): 406.68 (92.98)) until 6–8 weeks post-CRT (391.26 (82.99)), followed by significant recovery within 4–6 months post-CRT (407.35 (94.08)), and another decrease by 12 months post-CRT (394.50 (93.75)) (Fig 1). There was significant correlation between the MDASI-Interference items of general activity (est = -0.0063, p = 0.0051), work (est = -0.0059, p = 0.0140), walking (est = -0.0055, p = 0.0084), and enjoyment of life (est = -0.0058, p = 0.0155) with 6MW distance overtime. More severe shortness of breath (est = -0.0071, p = 0.0003) and coughing (est = -0.0046, p = 0.0228) were also significantly related to shorter distance walked on 6MW overtime. At each timepoint, there was a higher completion rate for MDASI versus 6MW. Patients who didn’t complete 6MW reported higher mean scores for WAW at all timepoints except week 2–3 during CRT.

### Conclusion

Physical functioning and adverse event related PROs on MDASI-L are highly relevant to the physical performance measure 6MW for NSCLC patients during the first year of treatment. Therefore, it represents a good surrogate for an in-person objective measure of physical function when a clinic visit is not possible.

**Fig. 1 (abstract 2037):**
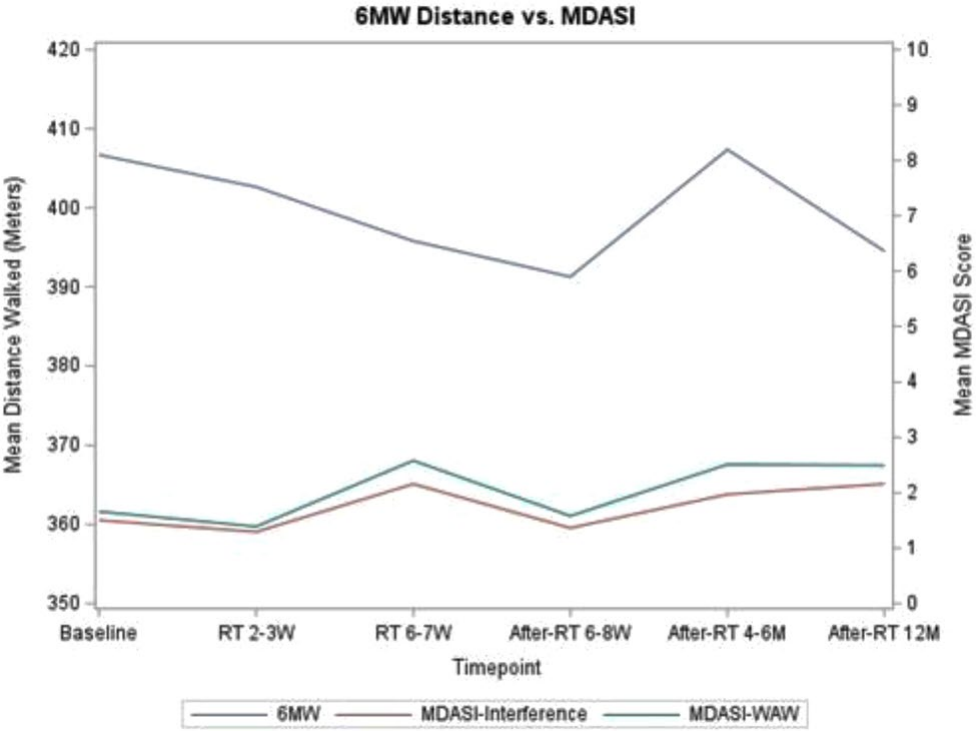
Developing a Dutch PROPr value set: study design and lessons learned

## 2038 How long is too long? Review of screenshots for electronic implementation of EORTC instruments

Dagmara Kulis^1^, Anaïs Simon^1^, Jens Lehmann^2^

^1^EORTC, Brussels, Belgium, ^2^Medical University of Innsbruck, Innsbruck, Austria

*Journal of Patient-Reported Outcomes 2026*, **10(Suppl 1)**:2038

### Aims

With reducing timelines to start of trials being a priority for sponsors, migration of patient-reported outcome (PRO) measures onto electronic platforms as ePROs remains a process perceived as excessively long, especially due to the copyright holder review (CHR) of the screenshots. Emergence of ePRO libraries of pre-migrated measures ready for deployment helps reduce timelines, but not all vendors build such libraries and most ePRO use of the European Organisation for Research and Treatment of Cancer (EORTC) measures requires study-specific migration. To see how much time CHR takes and what types of comments are made, we analysed submissions and turnaround times.

### Methods

Following data was collected for submissions received between 1st November 2024 and 31st March 2025: 1) dates of: receipt of the submission, reply, updated screenshots, final approval; 2) number of business days between each step; 3) number of rounds needed for final approval; 4) types of measures and devices; 5) types of feedback.

### Results

In said period, 41 review requests from 16 different vendors were received. 20 (48%) requests contained one instrument migrated for one or two device types. First round of CHR was done in one business day for 38 (93%) cases and 17 (41%) received final approval after one round (without any comments), with 14 (34%) within one business day.24 (59%) received at least one comment, and 2 (5%) – 7 or more. Most common issues were: no information about branching logic provided (where needed) (n=8); “circle” instead of “select” (n=4); typos and numbering mistakes introduced in migration (n=6). Feedback for 17 required updates solely to aspects covered by the EORTC ePRO Guidelines. Specific requests were made for screenshots review: in Chinese, Japanese and German (instead of English); within 3 hours from receipt.

### Conclusion

With tight timelines, all parties involved in PRO migration face responsibility to reduce delays. CHRs should strive to ensure smooth turnaround which can further be helped by ePRO vendors. Following freely available ePRO guidelines, ensuring quality of builds and providing branching information saves at least one round of review. A library of pre-approved ePROs remains the fastest way to deploy PRO measures in studies.

## 2039 Impact of Diabetes on Patient-Reported Outcomes After Lung Resection: A Prospective Cohort Study

Tingting Zhu^1^, Cheng Lei^2^, Qiuling Shi^3^, Wei Dai^4^

^1^School of Public Health, Chongqing Medical University, Chongqing, People’s Republic of China, China, ^2^Department of Thoracic Surgery, Sichuan Clinical Research Center for Cancer, Sichuan Cancer Hospital & Institute, Sichuan Cancer Center, University of Electronic Science and Technology of China, Chengdu, Sichuan, China, ^3^School of Public Health, Chongqing Medical University; Department of Thoracic Surgery, Sichuan Clinical Research Center for Cancer, Sichuan Cancer Hospital & Institute, Sichuan Cancer Center, Affiliated Cancer Hospital of the University of Electronic Scie, Chongqing, People’s Republic of China, China, ^4^Sichuan Cancer Hospital and Institute, Chengdu, China

*Journal of Patient-Reported Outcomes 2026*, **10(Suppl 1)**:2039

### Aims

Diabetes is a common comorbidity in patients undergoing lung resection, yet its impact on postoperative patient-reported outcomes (PROs) remains unclear. This study aimed to evaluate the influence of preoperative diabetes on PROs following lung resection surgery.

### Methods

A prospective cohort study (CN-PRO-Lung Part3) was conducted, analyzing data from 2774 patients who underwent lung resection at a large specialized cancer hospital between April 2021 and December 2023. Patients were stratified into diabetic (n=180) and non-diabetic (n=2594) groups. Clinical characteristics, postoperative symptoms (e.g., pain, coughing), and functional impairments (e.g., activity limitation) were assessed. Statistical analyses included generalized estimating equation (GEE) models to estimate relative risks (RR) and Kaplan-Meier survival analysis for recovery times.

### Results

Diabetic patients were older (median age 60.0 vs. 54.0 years, P<0.001), had higher BMI (24.0 vs. 22.8 kg/m^2^, P<0.001), and more comorbidities (Charlson Index >0: 98.3% vs. 11.2%, P<0.001). Despite comparable lung function (FEV1% and DLCO%, P>0.05), diabetic patients had a lower proportion of adenocarcinoma (61.7% vs. 75.0%, P<0.001) and higher pathological stages >I (39.4% vs. 24.8%, P=0.001). GEE models adjusted for confounders showed no significant RR of diabetes on moderate-to-severe symptoms or functional impairments (all P>0.05), except a borderline reduction in post-discharge coughing risk (RR=0.733, P=0.189). Diabetic patients recovered slower from drowsiness (median 33 vs. 32 days, P=0.042).

### Conclusion

Preoperative diabetes was associated with distinct clinical profiles but did not significantly worsen most postoperative PROs. However, diabetic patients experienced delayed recovery from drowsiness, highlighting the need for targeted monitoring in this population. These findings support the importance of individualized postoperative care for diabetic patients undergoing lung resection.

## 2040 EORTC item Q168: the psychometric performance of a single-item indicator of side-effect burden for people with cancer

Tamara Jones^1^, Florian Zeman^2^, Michael Koller^2^, Andreia Moura^3^, Claire Piccinin^3^, Sandra Nolte^1^

^1^Person-Centred Research, Eastern Health Clinical School, Monash University, Melbourne, Australia, ^2^Center for Clinical Studies, University Hospital Regensburg, Regensburg, Germany, ^3^Quality of Life Department, European Organisation for Research and Treatment of Cancer (EORTC), Brussels, Belgium

*Journal of Patient-Reported Outcomes 2026*, **10(Suppl 1)**:2040

### Aims

Following the release of the US Food and Drug Administration (FDA) Guidance on core patient-reported outcomes in cancer clinical trials, there has been growing interest in using single items to measure treatment tolerability from the patient’s perspective. The FDA Guidance specifically mentions item Q168 “To what extent have you been troubled with side-effects from your treatment” from the European Organisation for Research and Treatment of Cancer (EORTC) Item Library. However, despite its frequent use, evidence of its psychometric performance is currently lacking. This study aims to evaluate the psychometric properties of item Q168 in a diverse sample of cancer patients.

### Methods

This was a multi-national, online panel study that collected cross-sectional item Q168 and EORTC QLQ-C30 data. Psychometric analyses of item Q168 will include: (1) convergent validity by examining correlations between item Q168 and related dimensions of quality of life, (2) known-groups validity by examining item Q168 responses based on clinical characteristics of participants, and (3) differential item functioning by examining the performance of item Q168 in subgroups of participants.

### Results

A sample of 2,643 cancer patients were recruited from 11 countries. Patients had a mean age of 58 years (range 18 to 92) and equal gender distribution. All major cancer types were represented, with the most common being breast (21%) and prostate cancer (14%). Psychometric analyses are ongoing and results will be ready for presentation at ISOQOL 2025.

### Conclusion

Following the release of the FDA Guidance and the launch of the EORTC Item Library, item Q168 is one of the most frequently requested items by industry users. This study aims to provide an evidence-based rationale for using a single-item measure of side effect burden, such as EORTC item Q168, both within cancer clinical trials and as part of routine care.

## 2041 Dynamic Screening Nutritional Risk Index on Treatment Tolerance and Hospitalization Costs in Cancer Patient: A 3-Year Longitudinal Cohort Study

Hongfan Yu^1^, Li Tang^2^, Qiuling Shi^1^, Xing Wei^3^

^1^State Key Laboratory of Ultrasound in Medicine and Engineering, College of Biomedical Engineering, Chongqing Medical University, Chongqing, China, ^2^Shaoxing Second Hospital, Shaoxing, China, ^3^Department of Thoracic Surgery, Sichuan Clinical Research Center for Cancer, Sichuan Cancer Hospital & Institute, Sichuan Cancer Center, Affiliated Cancer Hospital of the University of Electronic Science and Technology of China, Chengdu, China

*Journal of Patient-Reported Outcomes 2026*, **10(Suppl 1)**:2041

### Aims

Assessment of the advanced cancer patient developing malnutrition has been limited to cross-sectional design; however, the relationships between the dynamic trajectory of Nutritional Risk Index (NRI) and treatment tolerance and healthcare cost are poorly understood. To explores the relationship between nutritional risk, assessed using the NRI, hospitalization costs, and treatment tolerance in cancer patients through a longitudinal cohort study.

### Methods

This retrospective cohort study included 1,490 oncological patients treated at Shaoxing Second Hospital from January 2021 to April 2024. The last follow-up visit was September 2024. Longitudinal data comprising 8,028 hospital records across 26 treatment cycles were analyzed. NRI was calculated using serum albumin and body weight data, and patients were stratified into no/mild or moderate/severe nutritional risk groups. Linear mixed effect models were assessed the association between NRI and hospitalization costs, while Restricted Mean Survival Time (RMST) was used to compare treatment cycles.

### Results

Patients with moderate/severe nutritional risk exhibited significantly higher hospitalization costs than those with no/mild risk, especially during the initial phases of treatment. On average, they had 44% higher costs (Estimate = 1.44; 95% CI: 1.38–1.50; P < 0.001). Sensitivity analysis confirmed this trend across age, gender, and treatment history. Furthermore, metastatic patients with lower nutritional risks received significantly more treatment cycles (RMST: 9.56 vs. 8.15; difference = 1.41; P = 0.004).

### Conclusion

Nutritional risk, as measured by NRI, is a significant predictor of hospitalization costs and treatment duration in cancer patients. Early identification and management of nutritional deficiencies could improve treatment tolerance and reduce economic burden, underscoring the need to incorporate routine nutritional screening into oncological care pathways.

**Fig. 1 (abstract 2041a):**
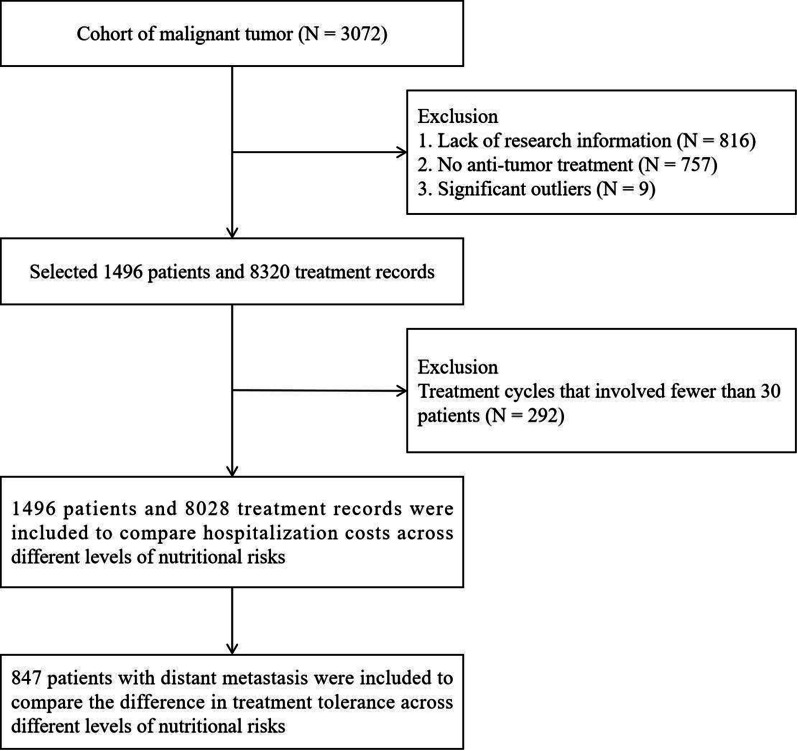
Dynamic Screening Nutritional Risk Index on Treatment Tolerance and Hospitalization Costs in Cancer Patient: A 3-Year Longitudinal Cohort Study

**Fig. 1 (abstract 2041b):**
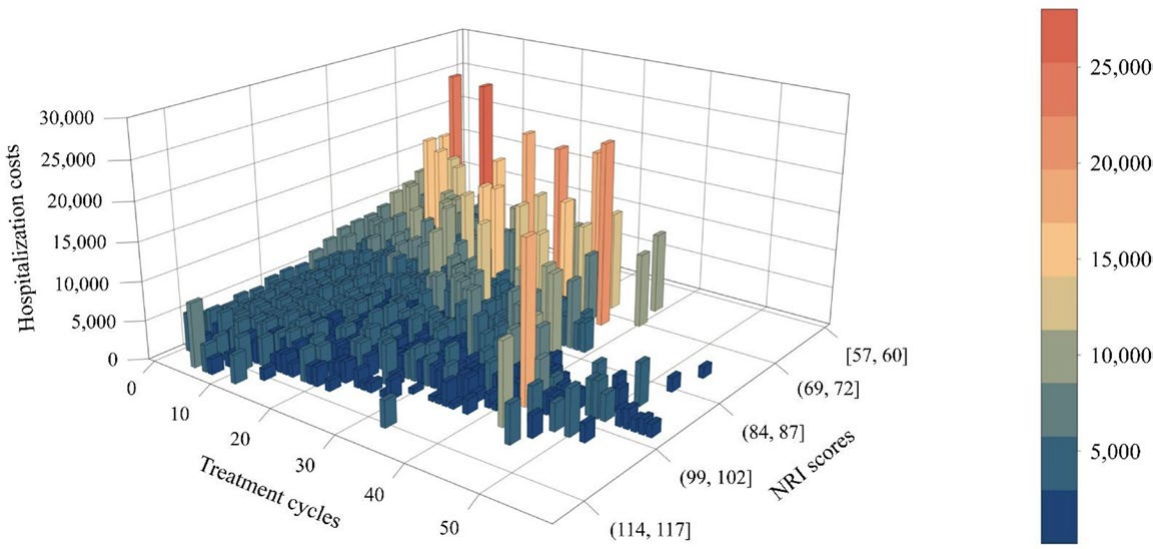
Dynamic Screening Nutritional Risk Index on Treatment Tolerance and Hospitalization Costs in Cancer Patient: A 3-Year Longitudinal Cohort Study

**Table 1 (abstract 2041):**
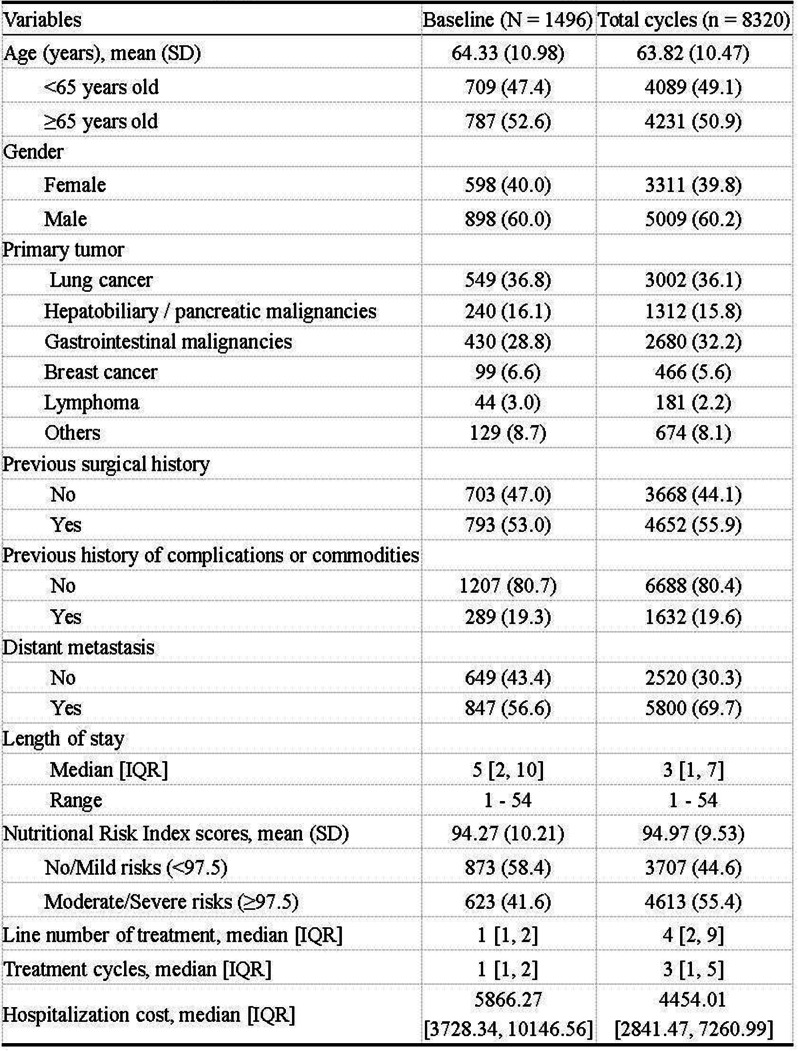
Baseline demographic and clinical characteristics of participants

## 2042 Study protocol for electronic patient-reported outcomes in a decentralised phase IV crossover trial for pulmonary arterial hypertension

Frances Varian^1^, Rebecca Burney^1^, Felicity Hitchcock^2^, Ellis Cerrone^1^, Hamza Zafar^1^, A.A. Roger Thompson^1^, David G. Kiely^2^, Jill Carlton^1^, Alexander M.K. Rothman^1^

^1^University of Sheffield, Sheffield, UK, ^2^Sheffield Teaching Hospitals NHS FT, Sheffield, UK

*Journal of Patient-Reported Outcomes 2026*, **10(Suppl 1)**:2042

### Aims

Pulmonary arterial hypertension (PAH) is a progressive, life-limiting condition that leads to right heart failure and premature death. People living with PAH consistently identify health-related quality of life (HRQoL) as the most important outcome of treatment. However, no clinical trials to date have prioritised HRQoL as an endpoint. We present the first decentralised clinical trial protocol in PAH to be adequately powered to evaluate HRQoL outcomes. We deliver novel digital PROM capture and daily remote haemodynamics to evaluate the relationship of HRQoL to haemodynamics and therapeutic response.

### Methods

PHoenix (NCT05825417) is a phase IV multi-centre 2x2 crossover trial evaluating treatment intensification with two guideline-recommended therapies. The main study protocol is published elsewhere. Seven individuals with lived experience of PAH contributed to PROM selection through patient and public involvement. Clinician-reported endpoints include cardiac biomarkers, cardiac magnetic resonance imaging and walk distance. Daily haemodynamic data (total pulmonary resistance, TPR) is transmitted using a home-reader from an implanted pulmonary artery pressure monitor (CardioMEMSTM) and an insertable cardiac monitor captures heart rate, rhythm, and daily activity. Digital PROMs, captured weekly using a mobile application (Atom5TM), include the emPHasis-10, a side effect questionnaire, and a single-item global anchor. Symptoms of anxiety and depression are assessed fortnightly. Protocol hypotheses were developed following a conceptual framework and systematic review of PROMs used in PAH trials. The trial is powered to evaluate PROMs as a secondary outcome.

### Results

PROM compliance is centrally monitored throughout the study. The primary hypothesis is that improvements in emPHasis-10 score – both at the individual- and group level – will correspond to greater reductions in remotely captured TPR. Secondary hypotheses propose that emPHasis-10 scores are sensitive to side effect burden, and that improvements in HRQoL will be attenuated in participants with elevated anxiety and depression symptoms. Analyses will include total and subscale emPHasis-10 scores, and explore heterogeneity by BMI, age, gender, co-morbidities and time since diagnosis.

### Conclusion

This is the first prospective clinical trial protocol in PAH evaluating HRQoL outcomes. Findings will provide critical insight into HRQOL responsiveness to two PAH therapies, enhance the psychometric evaluation of emPHasis-10 and inform future trial design to better reflect patient priorities.

## 2043 Meaningful changes in physical function and pain in patients with knee osteoarthritis

Xiaodan Tang^1^, Jin-Shei Lai^1^, Jeong Chan Ra^2^, Sung Keun Kang^2^, John Peipert^3^

^1^Northwestern University Feinberg School of Medicine, Chicago, Illinois, USA, ^2^Biostar Stem Cell Research Institute, Seoul, South Korea.^3^University of Birmingham, Birmingham, UK

*Journal of Patient-Reported Outcomes 2026*, **10(Suppl 1)**:2043

### Aims

This study aims to establish meaningful within-person change (MWPC) thresholds for the Total Western Ontario and McMaster Universities Osteoarthritis Index (WOMAC®), its Physical Function (PF) subscale, and the Visual Analog Scale (VAS) for pain in patients with knee osteoarthritis (OA). A secondary objective is to evaluate the effectiveness of a single injection of autologous culture-expanded adipose tissue-derived mesenchymal stem cells (ADMSCs) using these thresholds.

### Methods

The study included 252 patients with knee OA enrolled in a clinical trial. An anchor-based predictive modeling approach was used to determine MWPC thresholds for the WOMAC and VAS Pain scales, using the Knee Injury and Osteoarthritis Outcome Score (KOOS-12), the 36-Item Short Form Survey (SF-36), and the International Knee Documentation Committee (IKDC) scores with literature-based cutoffs as anchors. MWPC thresholds were derived from both the ADMSC injection and control (autoserum) groups at 3- and 6-month follow-ups. Treatment effectiveness was assessed by comparing between-group differences and within-person changes against the MWPC thresholds.

### Results

MWPC thresholds were identified as follows: 5–17 points for WOMAC Total, 4–12 points for WOMAC PF, and 8–14 points for VAS Pain (all on 0–100 scales). As shown in Figures 1 and 2, most patients in the ADMSC group reported meaningful improvements in physical function and pain at 6 months, with between-group differences exceeding the lower bound of MWPC thresholds. Furthermore, as shown in Figure 3, a higher proportion of patients in the ADMSC group achieved meaningful improvement compared to the control group at both 3 and 6 months.

### Conclusion

This study established MWPC thresholds for WOMAC and VAS Pain in patients with knee OA and demonstrated their utility in evaluating meaningful treatment benefits. These findings highlight the potential of ADMSC treatment to provide clinically relevant improvements in physical function and pain.

**Fig. 1 (abstract 2043):**
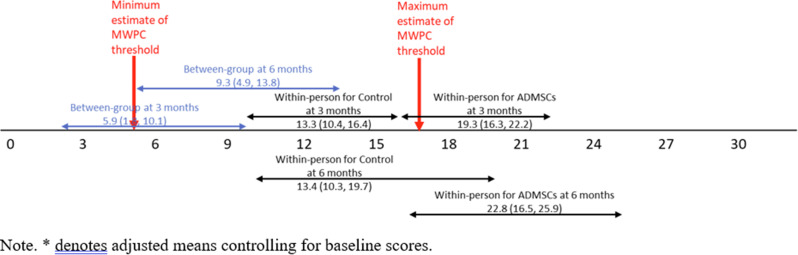
Estimated between-group differences in adjusted* means (with 95% confidence intervals) and adjusted* within-person change scores of each group at each time point for WOMAC Total

**Fig. 2 (abstract 2043):**
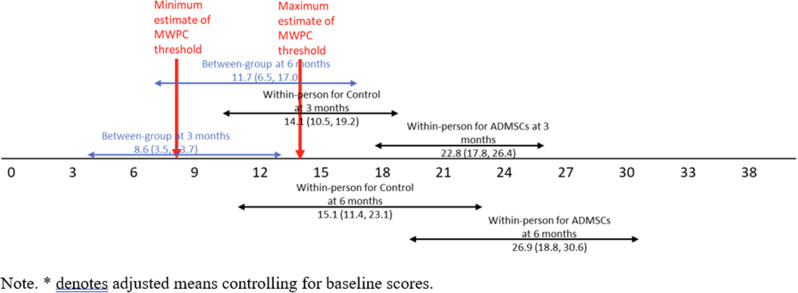
Estimated between-group differences in adjusted* means (with 95% confidence intervals) and adjusted* within-person change scores of each group (with 95% confidence intervals) at each time point for VAS Pain

**Fig. 3 (abstract 2043):**
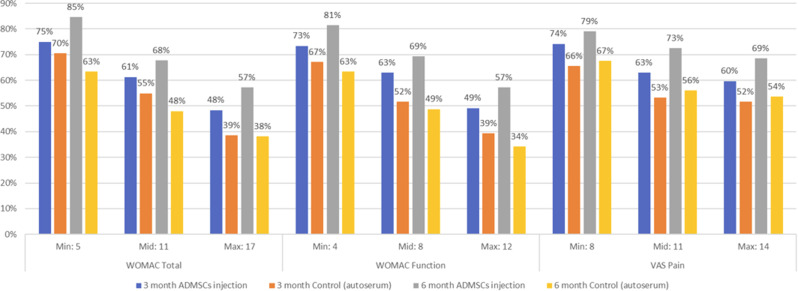
Proportions of participants having improvement reaching the minimum, midpoint, and maximum values of each MWPC range

## 2044 Using Item Response theory for validity evidence using the DASS-21 with a rural population in the highlands of Peru

Fiorella Guerrero^1^, Kevin Weinfurt^1^, Li Lin^1^, Stella Hartinger^2^, Daniel Maeusezahl^3^

^1^Duke University, Durham, North Carolina, USA, ^2^Universidad Peruana Cayetano Heredia, Lima, Peru, ^3^Swiss Tropical Public Health Institute, Allschwill, Switzerland

*Journal of Patient-Reported Outcomes 2026*, **10(Suppl 1)**:2044

### Aims

The DASS-21 (Depression, Anxiety, and Stress Scale-21) is a widely used, standardized psychological tool. The scale has gathered validity evidence across numerous cultural contexts, yet further validity evidence is needed for its use in the highlands of Peru. Item Response Theory (IRT) methods could inform performance of the measure at an item-level while allowing comparisons across samples. Also, a differential item functioning (DIF) analysis may identify possible threats to validity.

### Methods

Using cross-sectional data from a cohort in the highlands of Peru, we assessed structural validity and item performance using classic test theory and IRT methods. We examined item responses, the polychoric correlation matrix, and Cronbach’s alpha for each domain. We conducted a confirmatory factor analysis (CFA). For conducting IRT analyses, we checked for assumptions of unidimensionality, local dependence (LD), and monotonicity. We used the Graded Response Model (GRM) to evaluate item performance. We also conducted a DIF analysis across gender, education levels, and age groups and estimated its magnitude using wABC.

### Results

The sample comprises 3431 participants in rural Andean Peru, with a mean age of 39 years, 67.8% female, and 80.5% with less than 10 years of formal schooling. CFA did support a three-factor structure. Assumptions of monotonicity were met. Analyses to identify LD pairs of items found 19 pairs with LD. IRT analysis showed that item 21 (“life is meaningless”) and item 15 (“close to panic”) were the most informative. No DIF was found between females and males. DIF was found between groups with different education levels and across age groups, but no items had a large DIF magnitude.

### Conclusion

This study provides the first IRT-based evaluation of the DASS-21 in a Spanish-speaking population, offering important validity evidence for its use in a rural Andean setting. The Depression and Anxiety domains provided adequate information, demonstrating high discrimination and measurement invariance across gender, age, and education groups. In contrast, the Stress domain did not meet the assumptions required for IRT modeling. Still, the DASS-21 could be effectively used as a screening tool in resource-limited, high-altitude populations, especially to examine Depression and Anxiety at higher levels of the latent trait.

## 2045 Meaningful within-patient change of PROMIS-Physical Function in patients with cancer cachexia in the phase 2 ponsegromab study

Jarjieh Fang^1^, Joshua Roth^1^, Andrew Bushmakin^2^, Magdalena Harrington^1^, John Groarke^3^, Susie Collins^4^, Jeffrey Crawford^5^, Eric Roeland^6^, Joseph Cappelleri^2^

^1^Pfizer, New York, New York, USA, ^2^Pfizer, Groton, Connecticut, USA, ^3^Pfizer, Cambridge, Massachusetts, USA, ^4^Pfizer, Kent, UK, ^5^Duke Cancer Institute, Durham, USA, ^6^Knight Cancer Institute, Portland, USA

*Journal of Patient-Reported Outcomes 2026*, **10(Suppl 1)**:2045

### Aims

A meaningful within-patient change (MWPC) threshold is the difference between clinical outcomes assessment scores, such as patient-reported outcomes, that is considered meaningful to patients. This study estimated MWPC for the PROMIS-Physical Function 8c instrument in patients with cancer cachexia based on data from a recent randomized trial (NCT05546476; Groarke et al, NEJM, 2024).

### Methods

This prespecified analysis used blinded patient data from the randomized, double-blind, phase 2 trial in adults diagnosed with solid tumors and cachexia (by Fearon criteria). For PROMIS-Physical Function 8c, raw scores range from 8 to 40, with T-scores based on a mean of 50 and standard deviation of 10 (for the general US population). MWPC thresholds were estimated using an anchor-based analysis. Patient Global Impression of Severity (PGI-S) and Patient Global Impression of Change (PGI-C) in limitations in patients’ ability to do daily activities were used as anchors. Effect sizes were calculated by dividing MWPC values by the instrument’s corresponding baseline standard deviation scores.

### Results

Among the overall trial population (n=187), the median age was 67 (IQR 60-74) years, 63% of patients were male, and 40%, 32%, and 29% had non-small-cell lung, pancreatic, and colorectal cancer, respectively. A total of 95% had received systemic anticancer therapy, ranging from 1 to ≥4 lines of treatment. Analyses based on PGI-S and PGI-C included all available longitudinal data from 148 and 151 patients, respectively. Values for MWPC were 5.22 (raw scoring; PGI-S based) and 3.46 (raw scoring; PGI-C based) when using a 2-category change in the anchor; MWPC estimations in terms of T-scores were 6.30 and 4.13, respectively. Regarding the effect sizes, these changes can be interpreted as “large” (PGI-S-based) and “medium” (PGI-C-based). Data are summarized in Table 1. The relationship between change from baseline in PROMIS-Physical Function and change in PGI-S or PGI-C was approximately linear (Figure 1).

### Conclusion

When a 2-point change in the anchor is used to define MWPC in cancer cachexia, values of 5.22 and 3.46, scored in the original units, can be considered MWPCs for PROMIS-Physical Function. Values of 6.30 and 4.13 can be considered MWPCs for PROMIS-Physical Function when represented as T-scores.

**Table 1 (abstract 2045):**
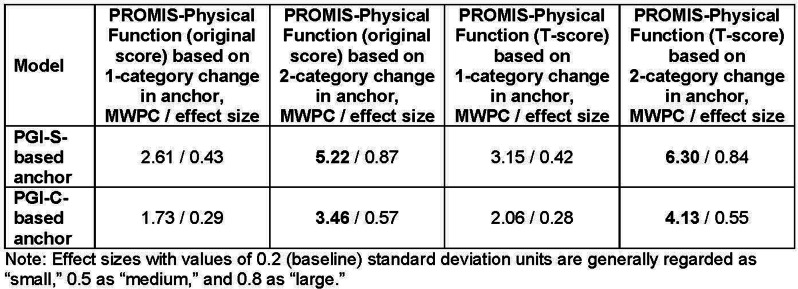
MWPC estimates

**Fig. 1 (abstract 2045):**
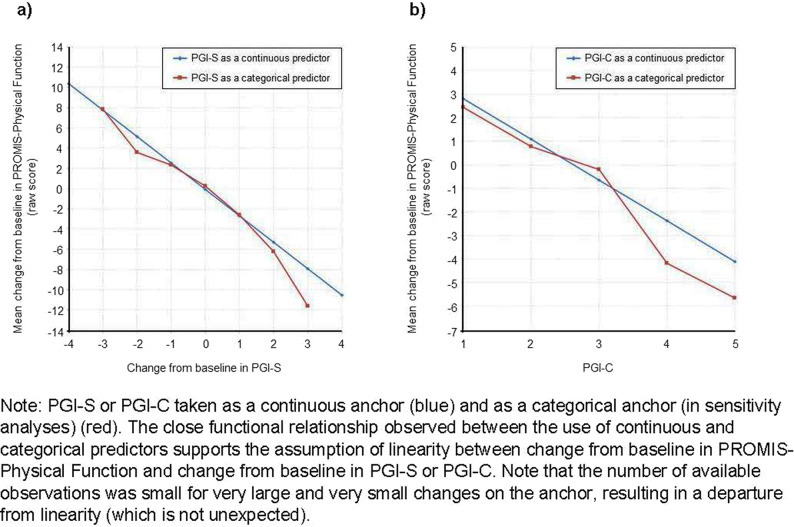
Relationship between changes in PROMIS-Physical Function and changes in PGI-S (**a**) or PGI-C (**b**)

## 2046 Results from a literature review on available methods for defining Meaningful Between-Group Difference (MBGD) to be applied to the Attention-Deficit/Hyperactivity Disorder (ADHD) Investigator Symptom Rating (AISRS) Scale

Angély Loubert^1^, Juliette Meunier^1^, Dorothee Oberdhan^2^

^1^Modus Outcomes, Lyon, France, ^2^Otsuka Pharmaceutical Companies, Alexandria, Virginia, USA

*Journal of Patient-Reported Outcomes 2026*, **10(Suppl 1)**:2046

### Aims

Recent regulatory guidance in the US has focused on defining meaningful within-patient change (MWPC), however clinical trial results are commonly reported as group-level changes in the literature and used for decision-making for a variety of stakeholders. There is growing interest in defining meaningful between-group difference (MBGD) thresholds for interpretation of Clinical Outcomes Assessment (COA) results. MBGD corresponds to the threshold for considering meaningfulness of results from clinical trial endpoints based on continuous difference between treatment groups (e.g., difference in mean scores at a given timepoint), and is different from MWPC, which is meant for interpretation of individual changes. However, and contrary to MWPC, there is no clear guidance on how MBGD should be defined in practice. The objective of this research was thus to conduct a targeted literature review on current scientific and regulatory perspective on MBGD definition, to identify possible approaches in general, and in the perspective of future application to the Attention-Deficit/Hyperactivity Disorder (ADHD) Investigator Symptom Rating (AISRS) Scale.

### Methods

A PubMed search was performed with various search strategies, along with identification of appropriate articles by reviewing key known articles on MBGD. Identified methods were listed within different approaches, along with existing regulatory perspective, when available.

### Results

Seven different approaches were identified. Most approaches were based on anchor-based and distribution-based methods, while others used meaningful score regions (MSRs), consensus-based review of possible scenarios by stakeholders, and pre-defined criterion. The approaches present different advantages and limitations considering their conceptual appropriateness, alignment with regulatory perspective, and complexity of implementation, which were listed. For illustrative purposes, each approach is presented with existing result based on the AISRS scale.

### Conclusion

This research confirms there is no consensus on how to define MBGD, nor even on the terminology to be used to refer to it. While some approaches may be more appealing than others, none is ideal considering all examined perspectives. Meaning of MBGD also appear unclear, which raise the question of relevance of such threshold for interpretation of clinical trial results. Additional work would thus be needed to solidify its conceptual relevance, and eventually achieve the same level of consensus than for MWPC.

## 2047 Evaluation of digital readiness in pulmonary hypertension: a study on health technology implementation and professional insights

Frances Varian^1^, Rebecca Burney^1^, Jenna Ablott^2^, Felicity Hitchcock^2^, Michelle Agboola^1^, Gregg Rawlings^1^, Ellis Cerrone^2^, Ze Ming Goh^1^, Kaushika Rautray^2^, Michael Sharkey^2^, Krit Dwivedi^2^, Samer Alabed^2^, Andy Swift^2^, Hamza Zafar^1^, David G. Kiely^2^, A.A. Roger Thompson^1^, Tim Chico^2^, Jill Carlton^1^, Brian McCullagh^3^, Alexander M.K. Rothman^1^, Ciara McCormack^3^

^1^University of Sheffield, Sheffield, UK, ^2^Sheffield Teaching Hospitals NHS FT, Sheffield, UK, ^3^Misericordiae University Hospital, Dublin, Ireland

*Journal of Patient-Reported Outcomes 2026*, **10(Suppl 1)**:2047

### Aims

Potential advantages offered by digital health technologies (DHTs) in Pulmonary Hypertension (PH) include improved diagnostic detection, increased healthcare efficiency, reduced costs and enhanced health literacy. However, implementation can be challenging. The Medical Research Council (MRC, 2021) complex interventions framework recommends identifying study design and key implementation phases to evaluate integration of DHT into clinical practice. This assessment informs decisions on whether to adopt, adapt, or abandon the DHT. We aim to conduct a systematic review and meta-narrative analysis of digital readiness using the MRC framework and explore healthcare professionals’ perceptions of DHTs in PH.

### Methods

The systematic review and meta-narrative was registered on PROSPERO (CRD42024622031). MRC proposes four research styles – effectiveness, efficacy, system-based, and theory-based – and four implementation phases – developmental/experimental, feasibility, evaluation, and widespread integration – for evaluation. A survey and semi-structured interviews were conducted with professionals representing all nine UK and Ireland specialist PH centres (REC 1/378/2448). Interviews were transcribed and thematically analysed independently by two authors (FV/CM).

### Results

Of 1,106 screened articles, 49 were included. A Sankey diagram (figure 1) represents the literature style and implementation stage of DHTs. Activity and AI represented the most published DHTs. Most evaluations focussed on efficacy in the experimental/developmental implementation stage. AI’s key strengths included reproducibility, demonstration of need, and cost-effectiveness. However, real-world implementation, and impact on decision-making require further research.Fifty healthcare professionals started the survey, with 41 completing it in full (65% were female). While 90% considered themselves ‘digitally ready’ (figure 2), up to 25% reported no discussion of DHT in clinical practice. Additionally, nine interviews were conducted, reaching saturation (i.e. all concepts elicited). The key advantages identified were improved data accuracy, enhanced patient care, and time-savings. Real-world challenges included issues with data management, and supporting education and access for both patients and professionals.

### Conclusion

Applying the MRC framework to DHT implementation in PH highlighted promising technological advances but also revealed gaps in adoption and clinical integration. The survey and interviews followed a similar trend, supporting high digital acceptance among PH professionals, but low uptake in clinical practice. A system- and theory-based approach may support broader DHT adoption for patient benefit.

**Fig. 2 (abstract 2047):**
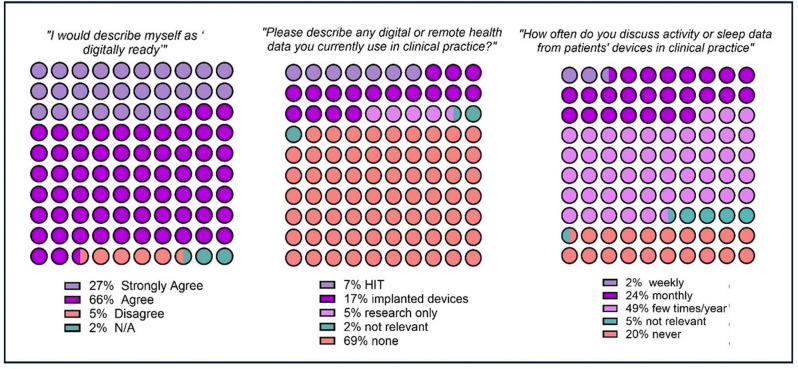
Sample of survey results of professional perceptions and use of digital health technologies in clinical practice. N = 41, 53% from nursing, 37% medical and 10% from an allied healthcare professional background

**Fig. 1 (abstract 2047):**
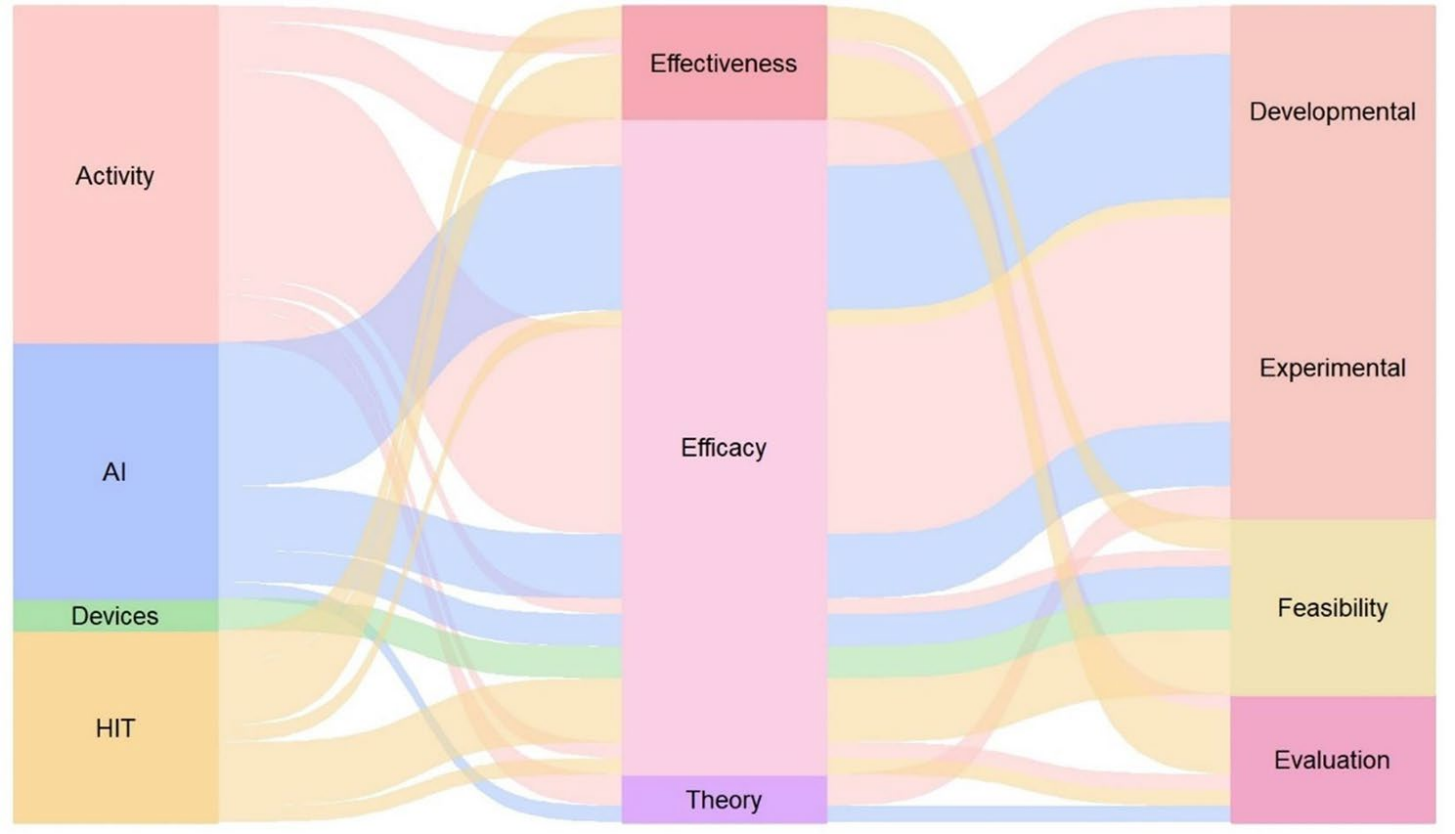
Sankey diagram for digital health technologies in pulmonary hypertension, flow representative of publications (n=) with evaluation according to MRC-research perspective (efficacy, effectiveness, theory and systems) and stage of implementation (developmental/experimental, feasibility, evaluation and integration). DHTs were grouped into: Al artificial intelligence, Activity (including wearables), Devices = implanted devices and HIT Health Intervention Technology (to include behavioural change and electronic patient-reported outcome measures). Total n = 42* (*7 review articles not included in this evaluation)

## 2048 Measuring Health-Related Quality of Life using the EQ-5D-5L in the Lebanese Adult Population

Fatima Al Sayah^1^, Lea Ghajar^2^, Rouba Ballout^3^, Samer A. Kharroubi^3^

^1^Alberta PROMs & EQ-5D Research & Support Unit (APERSU), School of Public Health, University of Alberta, Edmonton, Alberta, Canada, ^2^Center for Clinical, Health Economics and Outcomes Research (CCHO), Beirut, Lebanon, ^3^Department of Nutrition and Food Sciences, Faculty of Agricultural and Food Sciences, American University of Beirut, Beirut, Lebanon

*Journal of Patient-Reported Outcomes 2026*, **10(Suppl 1)**:2048

### Aims

Lebanon has endured compounding crises since 2019, including economic collapse, the COVID-19 pandemic, and the devastating 2020 Beirut Port Explosion, potentially impacting the health-related quality of life (HRQoL) and psychological well-being of its population. This study aimed to assess HRQoL using the EQ-5D-5L and examine its measurement properties and performance in a representative sample of Lebanese adults.

### Methods

A cross-sectional online survey was conducted with 519 adults (mean age 38.2 ± 16.5 years, 55.5% female) between March and September 2024. Quota sampling based on age, sex and geographic distribution was used to ensure a representative sample. Participants completed questionnaires on socio-demographics, the EQ-5D-5L, the SF-12v1, and the Beirut Distress Scale (BDS). Descriptive statistics summarized sample characteristics and EQ-5D-5L responses. Construct validity of the EQ-5D-5L was assessed using convergent and divergent validity, and known-groups validity.

### Results

While most reported no problems in mobility (77.3%), self-care (91.4%), and usual activities (74.1%), substantial proportions reported pain/discomfort (55.2% Levels 2-5) and anxiety/depression (43.1% Levels 2-5). Ceiling effect was 21.8% (health state 11111), and floor effect was 0.2% (health state 55555). The mean (SD) EQ VAS was 74.2 (19.0) and total sum score was 7.8 (3.2). Individuals with fair/poor self-reported health reported significantly more problems across all EQ-5D-5L dimensions and lower EQ VAS scores compared to those with better health. The EQ-5D-5L TSS effectively distinguished between disease groups with mostly moderate-large effect sizes (bowel disease 1.1, depression 1.0, hypertension 0.9, anxiety 0.8, sleep disorder 0.7, migraine 0.5, obesity 0.4). Similarly, EQ VAS differentiated between disease groups with moderate-large effect sizes (bowel disease 1.1, depression 0.9, hypertension 0.8, sleep disorder 0.7, anxiety 0.5, migraine 0.5, obesity 0.3). Dimensions of the EQ-5D-5L showed high correlation with conceptually similar SF-12 domains and BDS items and low correlation with dissimilar domains, providing evidence of convergent and divergent validity, respectively.

### Conclusion

This study provides critical insights into the HRQoL profile of the Lebanese adult population amidst ongoing crises, highlighting a significant burden of pain/discomfort and anxiety/depression. The EQ-5D-5L demonstrated good measurement properties, including construct and known-groups validity, in this context.

## 2049 Study on Perioperative Symptom Trajectories in HPV-Infected Patients Based on Patient-Reported Outcomes

Dengfeng Chen^1^, Wenlin Wu^1^, Qiuling Shi^1^

^1^Chongqing Medical University, Chongqing, China

*Journal of Patient-Reported Outcomes 2026*, **10(Suppl 1)**:2049

### Aims

High-risk HPV infection is the primary cause of cervical precancerous lesions and malignancies. However, systematic research on perioperative symptom trajectories and patient-reported outcomes (PROs) remains limited. This study aims to characterize the dynamic trajectories of perioperative symptoms and explore associations between symptom persistence and demographic/clinical factors.

### Methods

1.Study Design: Prospective observational cohort study.2.Participants: 184 patients with high-risk HPV infection (age ≥18 years) undergoing cervical surgery, including 3 cases requiring secondary treatment.3. Data Collection: Time Points: Preoperative to 3 months postoperative (13 follow-up nodes). Tools: Perioperative Symptom Recovery Assessment Scale (evaluating vaginal discharge, vaginal bleeding, abnormal secretions, lower abdominal pain, pelvic heaviness, vulvar pruritus, anxiety, and activity limitations).4. Analysis:Descriptive statistics for demographics (age, BMI, socioeconomic status) and clinical characteristics (HPV subtypes, cytology, surgical modalities). Symptom trajectory visualization using GraphPad Prism 10.

### Results

1.Population Characteristics: Predominantly young/middle-aged (68.1% aged ≤49 years), low-income (94.8% with monthly income <¥5,000), and low HPV vaccination rate (25%). Clinical Features: HPV 16/18 subtypes accounted for 47.5%; histopathology revealed 50.9% with high-grade squamous intraepithelial lesion (HSIL) or worse; focused ultrasound therapy was the primary surgical approach (58.0%). 2.Symptom Trajectories: Peak Symptoms: - Vaginal discharge peaked on postoperative day 1 (mean score: 2.42). Anxiety was highest preoperatively (mean score: 2.79) and resurged at 3 months (mean score: 1.07). Vaginal bleeding resurged at postoperative week 3 (mean score: 1.81), likely linked to scab detachment. Recovery Patterns: Lower abdominal pain/pelvic heaviness resolved within 1 month. Vaginal discharge persisted for 3 months. 3. Compliance: Preoperative completion rate: 82.1%. Postoperative compliance fluctuated between 50%-60% over 3 months.

### Conclusion

1.Key Findings: Perioperative symptoms exhibit unique dynamic trajectories, necessitating targeted interventions for preoperative anxiety and persistent vaginal discharge. Compliance declines sharply after 1 month postoperatively, correlating with symptom alleviation, highlighting the need for optimized follow-up strategies. 2. Clinical Implications: Stage-Specific Management: Postoperative scab-related bleeding and persistent discharge require tailored interventions during weeks 2-3. Dual-Pathway Approach: Preoperative anxiety and postoperative symptom-triggered anxiety demand early psychological intervention combined with symptom control.

**Table (abstract 2049):**
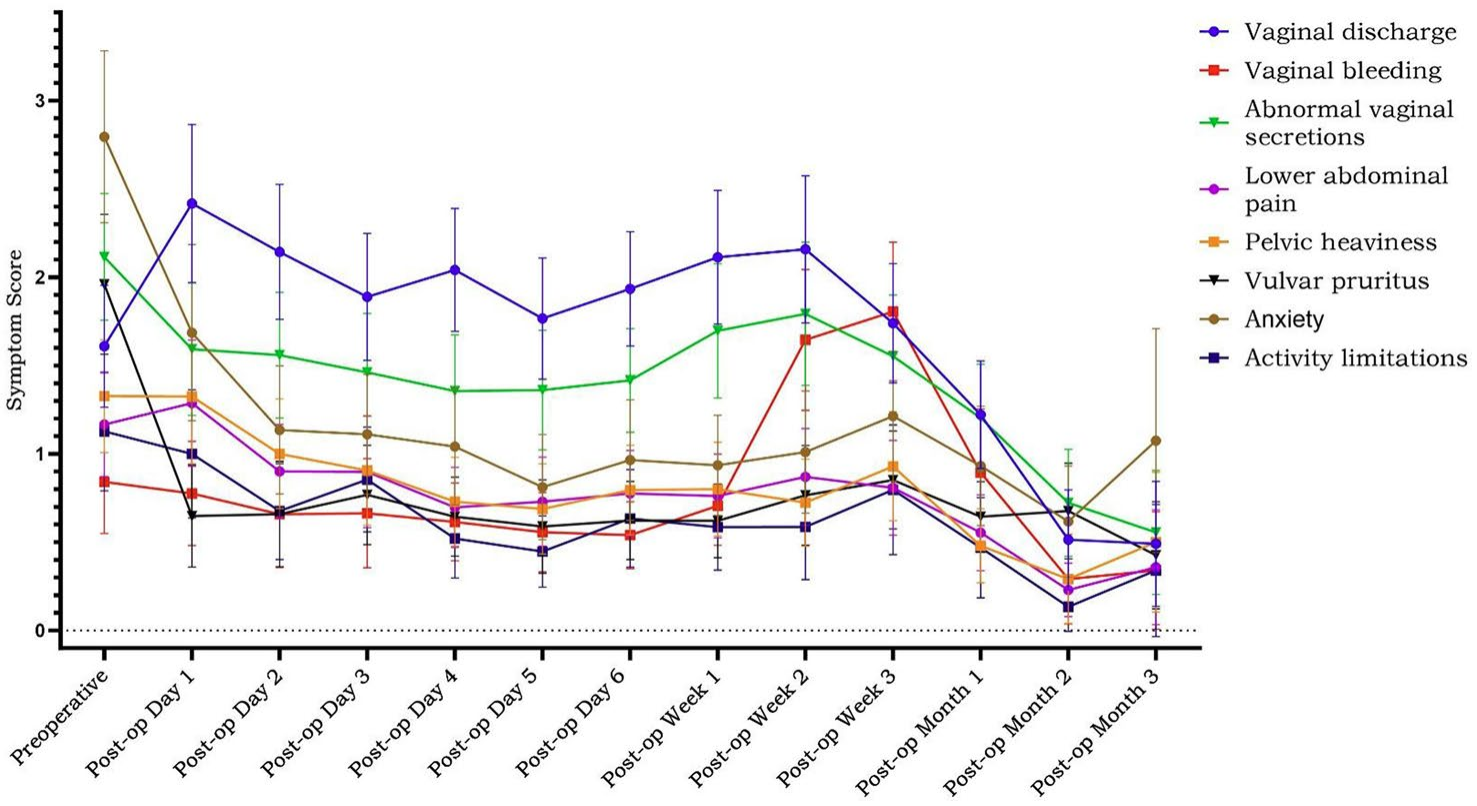
Perioperative Symptom Score Trajectories in HPV-Infected Patients

## 2050 Reliability, Longitudinal Measurement Invariance, and Responsiveness of PROMs in a Japanese Web-Based Cognitive Behavioral Healthcare Intervention for Premenstrual Syndrome: A Secondary Analysis of a Randomized Controlled Trial

Yoshitake Takebayashi^1^, Hideki Sato^2^, Noriko Numata^3^, Miho Egawa^4^, Yumie Ikeda^5^, Kaori Tsuyuki^6^, Takuma Ohsuga^6^

^1^Department Health Risk Communication, Fukushima Medical University School of Medicine, Fukushima, Japan, ^2^Department of Disaster Psychiatry, Fukushima Medical University School of Medicine, Fukushima, Japan, ^3^Department Cognitive Behavioral Physiology, Chiba University Graduate School of Medicine, Chiba, Japan, ^4^Department of Gynecology and Obstetrics, Kyoto University Graduate School of Medicine, Kyoto, Japan, ^5^UMISORA Clinic Department of Gynecology, Kyoto, Japan, Kyoto, Japan, ^6^Department of Gynecology and Obstetrics, Kyoto University Graduate School of Medicine, Kyoto, Japan

*Journal of Patient-Reported Outcomes 2026*, **10(Suppl 1)**:2050

### Aims

This study evaluated the test-retest reliability, longitudinal measurement invariance (LMI), and responsiveness of three patient-reported outcome measures (PROMs) in the context of a web-based cognitive behavioral healthcare intervention for adult women experiencing premenstrual syndrome (PMS) symptoms.

### Methods

This secondary analysis used data from a randomized controlled trial (RCT) in Japan. A total of 245 adult women (mean age = 35.94 ± 6.41 years) with PMS symptoms participated in a structured web-based intervention that incorporated psychoeducation and stress management strategies. The intervention’s impact was assessed using three PROMs administered at two-time points: pre- and post-intervention. These included the PMS-8 (PMS symptoms), PHQ-9 (depressive symptoms), and WHO-5 (subjective well-being). LMI was tested using a series of confirmatory factor analysis (CFA) models evaluating configural, metric, and scalar invariance. Test-retest reliability was assessed using intraclass correlation coefficients (ICCs). Responsiveness was examined using change scores, inter-scale correlations, and receiver operating characteristic (ROC) curve analysis.

### Results

All three PROMs demonstrated full longitudinal measurement invariance, supporting score comparability over time. ICCs indicated good test-retest reliability for PHQ-9 (0.80 [95% CI: 0.75–0.84]) and PMS-8 (0.73 [95% CI: 0.68–0.77]), while WHO-5 showed moderate reliability (0.63 [95% CI: 0.54–0.89]), suggesting greater variability in subjective well-being reporting. Change scores showed moderate correlations: PHQ-9 and WHO-5 (r = –.47), PHQ-9 and PMS-8 (r = .22), and PMS-8 and WHO-5 (r = –.31), reflecting differing responsiveness. ROC analysis revealed good responsiveness to symptom worsening (AUC = .64–.89) but limited responsiveness to improvement (AUC = .46–.56).

### Conclusion

This secondary analysis of a Japanese RCT supports the PMS-8, PHQ-9, and WHO-5 as psychometrically sound PROMs for monitoring changes in PMS-related symptoms and well-being. While the measures demonstrated robust invariance and reliability, the moderate test-retest reliability of WHO-5 and limited responsiveness to improvement across PROMs suggest that caution is warranted when interpreting positive changes following web-based cognitive behavioral interventions.

## 2051 Linguistic and cross-cultural validation of the BODY-Q modules included in the Obesity ICHOM Set for their use in the Spanish and Catalan population

Clara Amat^1^, Marc Beisani^2^, Ana Portillo^1^, Aurea Martin^1^, Yolanda Pardo^3^, Olatz Garin^4^, Montserrat Ferrer^4^

^1^Hospital del Mar Research Institute, CIBER de Epidemiología y Salud Pública (CIBERESP), Universitat Pompeu Fabra, Barcelona, Spain, ^2^Hospital del Mar, Barcelona, Spain, ^3^Universitat Autònoma de Barcelona, Hospital del Mar Research Institute, CIBER de Epidemiología y Salud Pública (CIBERESP), Barcelona, Spain, ^4^Hospital del Mar Research Institute, CIBER de Epidemiología y Salud Pública (CIBERESP),Universitat Pompeu Fabra, Barcelona, Spain

*Journal of Patient-Reported Outcomes 2026*, **10(Suppl 1)**:2051

### Aims

The International Consortium for Health Outcomes Measurement (ICHOM) has recently published the Set of Patient-Centered Outcome Measures for Adults living with Obesity, in which there are 6 modules of the BODY-Q questionnaire (social, physical, psychological function and sexual function, body image and eating behavior). The aim of the present study was to cross-culturally adapt and validate these modules of the BODY-Q for their use in the Spanish and Catalan population.

### Methods

The 6 modules of the BODY-Q questionnaire selected by ICHOM include 48 items (10 items in the social functioning, 7 in the physical functioning, 10 in the psychological functioning, 5 in the sexual functioning, 7 in the body image, and 9 in the eating behaviour). The Q-Portfolio and ISPOR guidelines for translation and cultural adaptation for Patient-Reported Measures were followed both for the both versions: 1) Preparation of materials; 2) Direct dual translation; 3) Reconciliation of direct translations; 4) Back-translation; 5) Review of back-translation and development of consensus. Cognitive debriefing interviews with patients to assess comprehensibility (6) was conducted with bilingual patients (Spanish and Catalan) from the Bariatric Unit of Hospital del Mar in Barcelona. The last step (7) was the incorporation of patients’ suggestions and final approval by the original authors. The process was supervised by an expert committee consisting of two specialists in development and validation of PROMs and four clinicians with diverse profiles in the field.

### Results

Throughout all stages of the adaptation, no major linguistic, conceptual, or cultural discrepancies were identified. The translated versions were found to be semantically equivalent to the original instrument and easily understandable by participants during cognitive interviews. Minor adjustments were made to enhance clarity and fluency, but no content modifications were necessary.

### Conclusion

The Spanish and Catalan versions 6 modules of the BODY-Q questionnaire included in the ICHOM Set of Patient-Centered Outcome Measures for Adults living with Obesity maintain conceptual equivalence with the original instrument and are considered appropriate for use in clinical and research settings. Their structure, reliability, validity and responsiveness will be assessed in a multicentre ah-hoc study. Preliminary results are expected to be available in October 2025.

## 2052 Development and validation of a biliary tract cancer symptom scale for mortality prediction: A Longitudinal Cohort Study

Jian Li^1^

^1^Chongqing Medical Univerisity, Chongqing, China

*Journal of Patient-Reported Outcomes 2026*, **10(Suppl 1)**:2052

### Aims

Patients with biliary tract cancer have poor survival outcomes and may experience high symptom burden. Currently, there is almost no assessment tool specifically designed to monitor biliary tract cancer symptoms. There is an urgent need for a symptom monitoring tool to quantify symptoms in biliary cancer patients before death effectively.

### Methods

The biliary tract cancer symptom item bank is constructed through a systematic literature review. The list is then reduced through the Delphi method and discussions within the research team, resulting in the draft Symptom Assessment for Patients with Biliary Tract Cancer（Bi-SA）. Its psychological characteristics and clinical applicability are tested in cohort 1 using classical test theory and item response theory. Patients in Cohort 2 were monitored with Bi-SA for symptoms 6 months closer to death.

### Results

In total,175 biliary tract cancer patients and 32 clinicians participated in the study. A total of 23 symptoms were identified through a literature review, with 10 symptoms being retained after two rounds of the Delphi Expert Opinion Method and open-ended group discussions. By combining classical measurement theory and item response theory, Bi-SA exhibited strong measurement properties, demonstrating unidimensionality, high internal consistency (with Cronbach’s alphas all exceeding 0.7), and solid test-retest reliability (intraclass correlation coefficients ranging from 0.77 to 0.95). The scale also demonstrated good comprehensibility, sensitivity, and specificity. In cohort 2 (n=69), the tool symptoms showed that mean scores for taste changes, nausea, sleep disturbances, drowsiness, and itching remained relatively stable over 6 months. In contrast, symptoms such as pain, bloating, fatigue, oil aversion, and lack of appetite became more severe over time, particularly in the month closer to death.

### Conclusion

The Bi-SA is a reliable patient-reported outcomes tool for monitoring symptoms in patients with biliary tract cancer. It helps identify the need for regular, comprehensive care in end-of-life patients and captures significant changes that may otherwise go unnoticed in those who are overlooked.

**Table. 1 (abstract 2052a):**
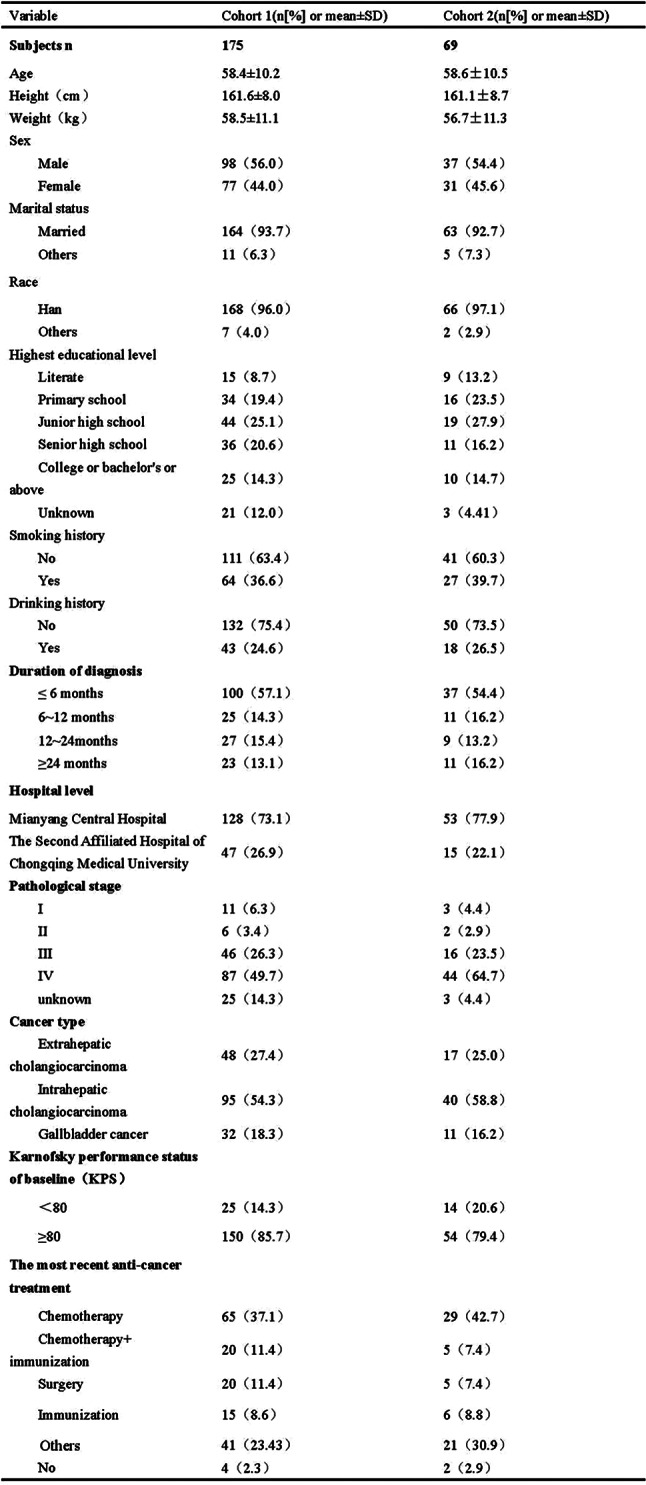
Development and validation of a biliary tract cancer symptom scale for mortality prediction: A Longitudinal Cohort Study

**Table. 1 (abstract 2052b):**
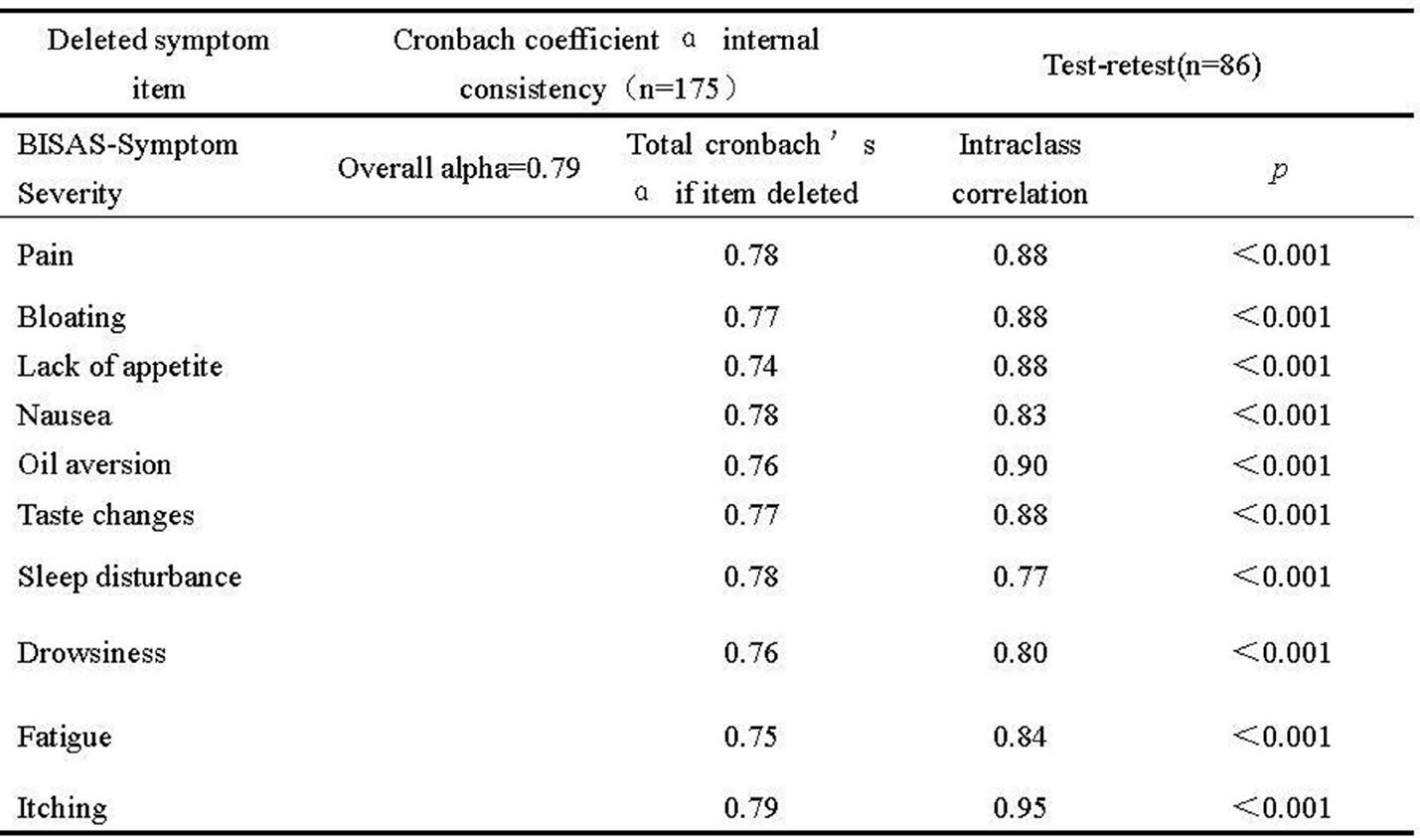
Development and validation of a biliary tract cancer symptom scale for mortality prediction: A Longitudinal Cohort Study

**Fig. 1 (abstract 2052c):**
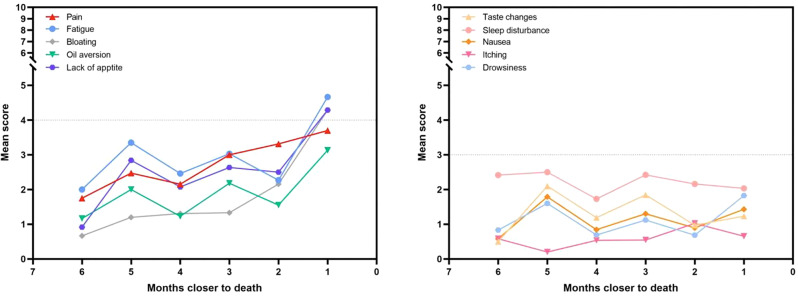
Development and validation of a biliary tract cancer symptom scale for mortality prediction: A Longitudinal Cohort Study

## 2053 Meaningful within-patient change assessment of the Quality of Life-Bronchiectasis Questionnaire Respiratory Symptom score (QOL-B RSS)

John Fastenau^1^, Vivian H. Shih^1^, Chunpeng Fan^1^, Nicholas Rockwood^2^, Lori McLeod^2^, Anne E. O’Donnell^3^

^1^Insmed Incorporated, Bridgewater, New Jersey, USA, ^2^RTI-Health Solutions, Research Triangle Park, North Carolina, USA, ^3^Georgetown University Medical Center, Washington, District Of Columbia, USA

*Journal of Patient-Reported Outcomes 2026*, **10(Suppl 1)**:2053

### Aims

Patients with non-cystic fibrosis bronchiectasis (hereafter bronchiectasis) have high symptom burden impacting their health-related quality of life. Prior analyses of the Quality of Life-Bronchiectasis Questionnaire (QOL-B) defined the minimal important difference in Respiratory Symptom score (QOL-B RSS) as 8 points. To further support clinical interpretation of changes in QOL-B RSS, an assessment of meaningful within-patient change (MWPC) of QOL-B RSS in patients with bronchiectasis was undertaken.

### Methods

From the ASPEN study (NCT04594369), which included adolescents and adults with bronchiectasis, we conducted treatment-agnostic analyses of baseline and week 52 data in adults aged ≥18 to ≤85 years with nonmissing QOL-B RSS baseline scores. Test-retest reliability was assessed using intraclass correlation coefficients and responsiveness to change using Spearman correlations. MWPC for QOL-B RSS was estimated using anchor-based and distribution-based methods, with change from baseline at week 52 in Patient Global Impression of Severity (PGIS) as the primary, and Patient Global Impression of Change (PGIC) at week 52 as the secondary, anchor measures.

### Results

Descriptive statistics are shown in the Table. QOL-B RSS test-retest reliability (baseline to week 2) was 0.74 (95% CI, 0.72-0.77), indicating sufficient reliability. Change from baseline at week 52 in QOL-B RSS was adequately associated with both change in PGIS (r = −0.48) and PGIC (r = −0.42), providing evidence that both are suitable anchor measures. The anchor-based primary MWPC threshold estimate (QOL-B RSS mean change from baseline at week 52 in patients with a 1-point improvement in PGIS) was 12.6 and median 13.0. Supportive threshold estimates (QOL-B RSS mean change in patients with “much improved” PGIC) were mean 10.0 and median 11.1. Distribution-based values for half-standard deviation (8.5) and standard error of measurement (5.8) were also supportive. A MWPC threshold range of 11–14 is proposed for improvement. PGIS and PGIC cumulative distribution plots demonstrated clear separation between adjacent groups at the proposed threshold range (Figures).

### Conclusion

MWPC threshold analyses suggest that improvements in QOL-B RSS in the range of 11–14 are meaningful in patients with bronchiectasis. These thresholds are consistent with prior definitions and may support interpretation of within-patient change in QOL-B RSS and treatment evaluation in this population.

**Table (abstract 2053):**
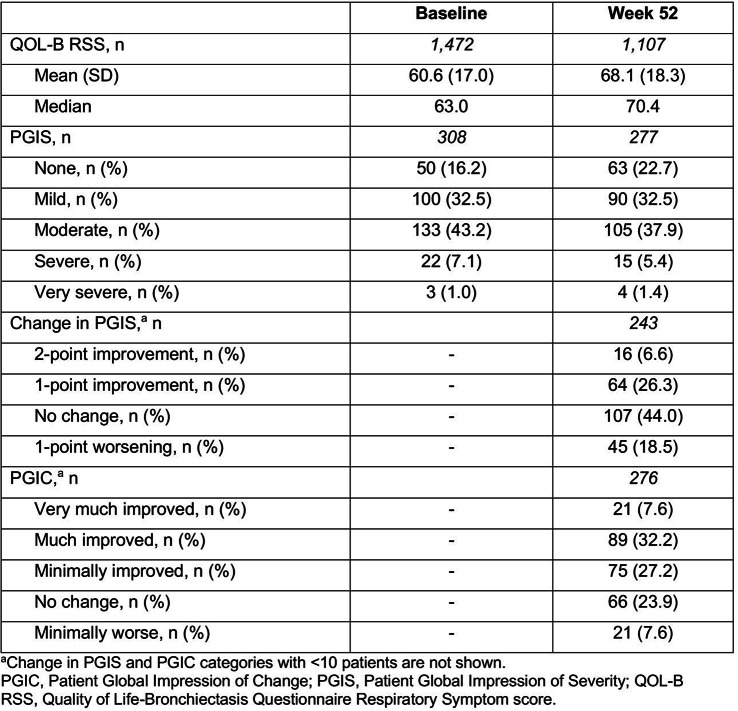
QOL-B RSS, PGIS, and PGIC

**Fig. 1 (abstract 2053):**
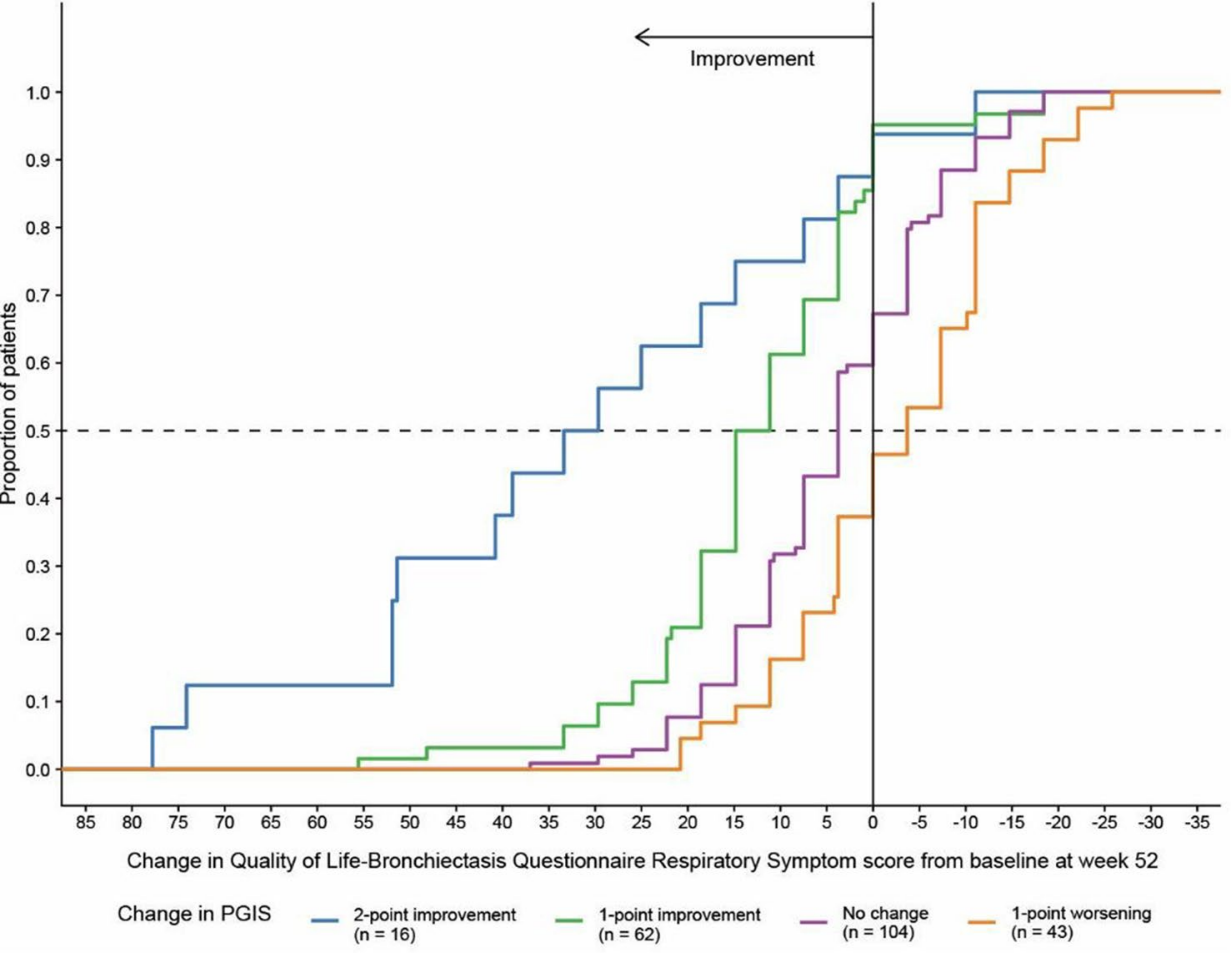
Empirical cumulative distribution function plot using change in PGIS

**Fig. 2 (abstract 2053):**
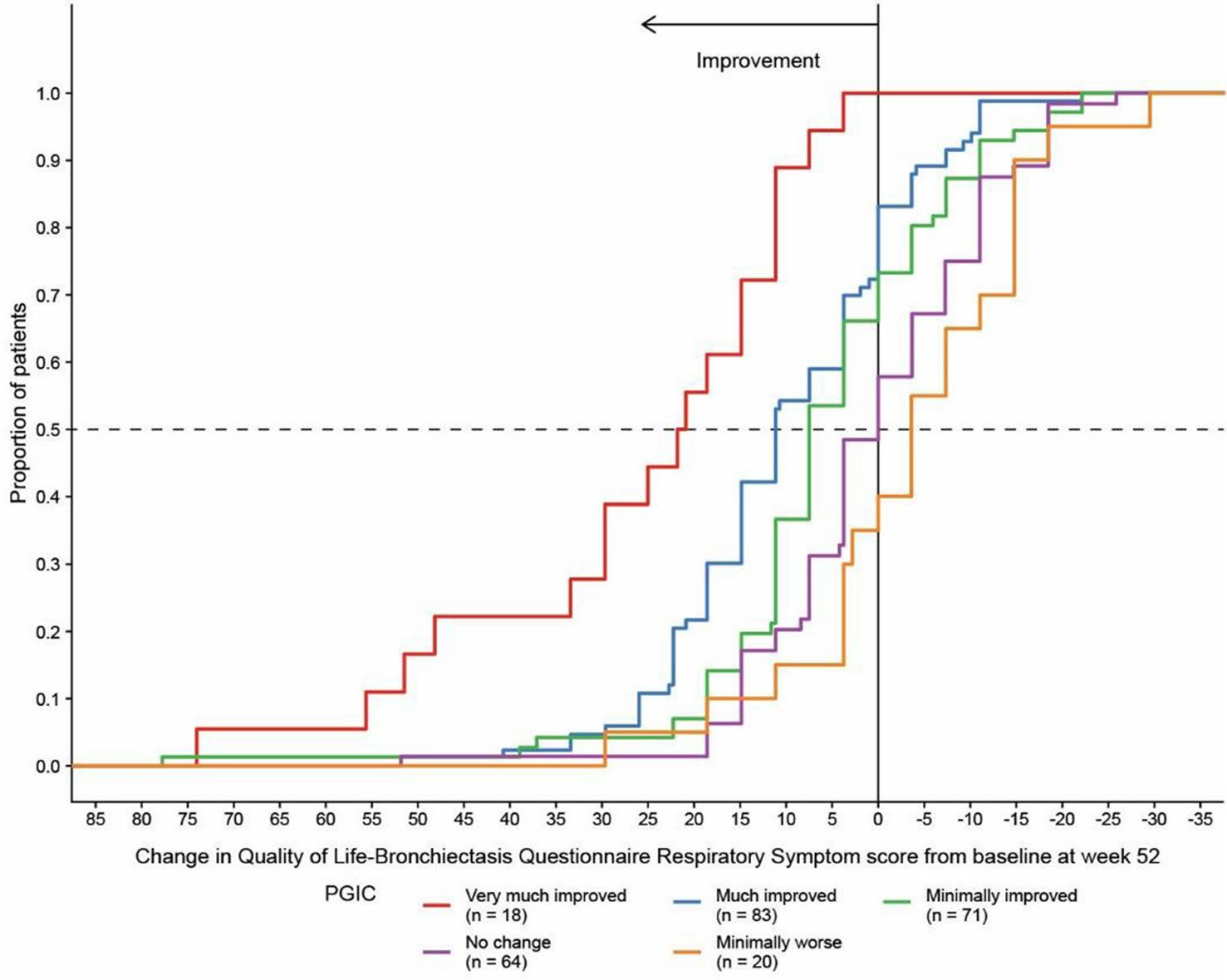
Empirical cumulative distribution function plot using PGIC

## 2054 Change over Time in PROMIS Global Health across Neurological Populations

Brittany Lapin^1^, Yadi Li^1^, Deborah Miller^1^, Irene Katzan^1^

^1^Cleveland Clinic, Cleveland, Ohio, USA

*Journal of Patient-Reported Outcomes 2026*, **10(Suppl 1)**:2054

### Aims

Our study leveraged real world data collected through the electronic health record to evaluate trajectories of health-related quality of life (HRQL) across chronic neurological conditions, and to explore factors associated with change over time.

### Methods

This is a retrospective longitudinal cohort study of adult patients with one of 10 conditions (amyotrophic lateral sclerosis (ALS), back pain, brain tumor, dementia, major depression, epilepsy, migraine, multiple sclerosis, Parkinson’s disease, stroke) who completed patient-reported outcomes as standard care between 10/2015-3/2023 at a large Neurological Institute. Patients completed PROMIS Global Health(GH), from which physical and mental summary scores (GPH, GMH) were calculated. GPH and GMH scores within 265–465 days after baseline were identified as 1-year follow-up. Changes in scores from baseline to 1-year follow-up were classified as 1) stable (within 5T-score points), 2) improved (change ≥5T-score points, 3) worsened (change ≤-5T-score points). Generalized estimating equations (GEEs) were constructed to evaluate the trajectory of PROMIS-GH from baseline to 1-year. Independent variables included timepoint and condition, adjusting for condition-specific severity categories, age, sex, race, marital status, ADI national rank quartile and Charlson comorbidity index.

### Results

There were 21,781 patients with PROMIS-GH scores at baseline and 1-year follow-up (mean age 50.5 (SD ±17) years, 67.5% female, 85.7% white race). Overall, 55.5% and 58.3% of patients remained stable on GMH and GPH, while 24.4% and 23.2% improved, respectively. The proportion of patients who worsened on GMH ranged from 16.3% with depression to 29.5% with ALS, and from 16.5% with headache to 38.5% with ALS for GPH. Significant predictors of worsening change over 1-year differed by condition, but overall included condition-specific severity, black race, unmarried, higher area deprivation, and more comorbidities. Increased age was associated with improvement on GMH and worsening on GPH, while female gender was associated with stability on GMH and worsening on GPH.

### Conclusion

Trajectories of HRQL using PROMIS-GH differed by neurological condition and severity of condition. Identified predictors of change can be targeted to tailor domain-specific treatment strategies. Additionally, through providing robust indicators of change in health status across neurologic conditions, this study provides support for the use of PROMIS-GH as a performance metric in value-based payment models.

## 2055 Methodological considerations for developing an Investigator Global Assessment (IGA) for use in dermatological studies

Helen Kendal^1^, Sophie Whyman^1^, Rob Arbuckle^1^, Laura Grant^1^, Elizabeth Gargon^1^, Sarah Bentley^1^

^1^Adelphi Values Ltd, Bollington, UK

*Journal of Patient-Reported Outcomes 2026*, **10(Suppl 1)**:2055

### Aims

The United States Food and Drug Administration (FDA) commonly recommend including Investigator Global Assessments (IGAs) as primary endpoints for pivotal trials in dermatological conditions. An IGA is a single-item, clinician-reported outcome (ClinRO) measure of overall disease severity at a given timepoint, using observable clinical signs, usually with response options ‘Clear’, ‘Almost Clear’, ‘Mild’, ‘Moderate’ and ‘Severe’; and descriptions of signs for each level.General guidance exists for development and validation of clinical outcome assessments (COAs); however guidance specific to IGA development is limited. While IGAs are often developed specifically for a single study, standardization of methods and best practice could facilitate consistency of IGAs across studies and conditions, reducing research waste. Here we provide recommendations for ensuring IGAs include the most relevant/important signs, have content validity, and are valid, reliable and responsive.

### Methods

Learnings from development of IGAs and related FDA feedback in several different dermatological conditions have been used to inform best practice recommendations.

### Results

The following methodological considerations are recommended for development of robust IGAs: To ensure content validity, dermatologists with expertise in the condition of interest should be actively involved in drafting and modifying the IGA. Selection of signs should focus on those which are observable at a single time point and can realistically change with treatment (i.e. avoid scarring or architectural changes unlikely to improve).In line with regulatory guidance, IGA response option descriptors should be clear, distinct and not overlapping.To generate evidence of relevance of signs included and consistent interpretation, the draft IGA should be debriefed with clinicians with similar backgrounds/expertise to those who will complete the measure during clinical trialsTraining materials and ideally an accompanying photo guide should be developed and also tested to support consistency of interpretation/completionEvaluation of inter- and intra-rater reliability is recommended where feasibleA comprehensive Item Tracking Matrix should be developed and updated iteratively throughout to document modifications and the origin of different components.

### Conclusion

These learnings demonstrate considerations for the development of IGAs for use in dermatological studies and highlight methods that can be adopted to ensure development of robust and appropriate IGAs.

## 2056 Evaluating the Effectiveness of an Electronic Data Collection Platform to Improve Patient Response Rates in a Canadian Orthopaedic Arthroscopic Surgery Registry

Sarah Harris^1^, Sheila Mcrae^1^, Marc Morisette^2^, Peter MacDonald^1^, Jarret Woodmass^1^

^1^Pan Am Clinic Foundation, Winnipeg, Manitoba, Canada, ^2^Shared Health Manitoba, Winnipeg, Manitoba, Canada

*Journal of Patient-Reported Outcomes 2026*, **10(Suppl 1)**:2056

### Aims

Registry programs to collect clinical and patient-reported outcome measures (PROMs) data are emerging in the field of soft-tissue sports surgery. Lack of response to PROMs questionnaires results in inefficient programming and there is little guidance on how to improve engagement in PROMs registries. Three data collection procedures were trialed to improve the effectiveness of an institutional knee and shoulder registry: emailed PROMs (EPROMs), EPROMs with a reminder phone call, and traditional paper-based PROMs.

### Methods

An electronic medical records (EMR) integrated software was selected. Surgeries from June 1, 2023 to January 31, 2024 were included. Preoperatively patients selected preference for email or paper questionnaires. All patients received questionnaires 1-year postoperatively and half who selected email were randomly assigned to receive a reminder phone call if the questionnaire was not complete. Logistic regression assessed the effect of age, sex, contact method, and reminder phone call on response status; odds ratios (ORs) and 95% confidence intervals (CIs) were estimates. Tasks related to PROMs collection and maintenance were categorized as data entry, data collection, patient records maintenance, and reminder phone calls. The number of hours committed to each task was diarized by research coordinators on a daily basis.

### Results

There were 509 procedures included (knee N=245; shoulder N=264) and 79% of patients preferred email. The EPROMs response was 30% (65/217) without a reminder and 45% (84/187) in the reminder group. Sixty-one percent (64/105) of paper questionnaires were returned. After adjusting for age and sex, patients who received EPROMs without a reminder had lower odds of response compared to paper questionnaires (OR=0.30; p<0.001; [95%CI 0.18 to 0.50]). Out of patients who received EPROMs, those with a reminder had higher odds of response (OR=1.92; p=0.002; [95%CI 1.26 to 2.93]). The mean data collection time per questionnaire was highest in the paper group (paper=16mins; email=6mins; email with call=10mins).

### Conclusion

The registry was successfully able to improve the efficiency of PROMs data collection. The majority of patients elected to participate in EPROMs. When combined with reminder phone calls, EPROMs had an equivalent rate of response and reduced resources compared to paper.

## 2057 Future of Linguistic Validation: Will Machine Translation replace or augment LingVal?

Willie Muehlhausen^1^, Himanshu Vashisht^2^, Tomas Ward^3^

^1^SAFIRA, Cloughjordan, Ireland, ^2^Zazu Systems Ltd, Dublin, Ireland, ^3^Dublin City University, Dublin, Ireland

*Journal of Patient-Reported Outcomes 2026*, **10(Suppl 1)**:2057

### Aims

This abstract explores whether traditional linguistic validation of patient-reported outcome (PRO) measures faces obsolescence or transformation in the era of machine translation and artificial intelligence (AI). The objective is to assess the current and future roles of human-led linguistic validation versus technology-augmented approaches in ensuring conceptual equivalence, cultural appropriateness, and regulatory compliance in PRO translations.

### Methods

We used one generic and another therapeutic area specific PROM and their linguistically validated translations for this project. We used multiple AI systems to translate the US-English version into several languages. The quality of these AI translations was then programmatically compared with the LV version. Experienced translators were then in a blinded setup asked to evaluate the quality of both versions.

### Results

Often the machine translations are qualitatively comparable with the translations from a full linguistic validation process. There were differences in the wording, but often these differences did not matter. Not all machine translation systems deliver equal qualities and there were differences in the quality amongst the languages. However, machine translations of simple concepts from the generic PROM were of a high quality needed for regulatory approval.A process including an orchestration of multiple machines and a human in the loop resulted in the highest quality translations.

### Conclusion

The results showed that Machine Translations can produce acceptable quality translations when the process includes multiple AI systems and a human in the loop for final approval. More work is needed to look at more complex languages and instruments for specific therapeutic PROMS.Linguistic validation is not heading for extinction (yet) but can be fundamentally augmented by machine translation and AI. The future of PRO translation lies in harmonising technological advancements with human oversight, ensuring both efficiency and the preservation of conceptual and cultural integrity.The mindful combination of the traditional LV process with AI capabilities will improve the timelines and cost of PRO translations.

## 2058 The Future of Linguistic Validation is Now: Enhancing Reconciliation Process Compliance with GenAI

Melinda Johnson^1^, Elisabet Sas Olesa^2^, Karolina Elizondo Jimenez^3^

^1^Lionbridge, Cary, North Carolina, USA, ^2^Translating and Conference Interpreting, Lionbridge Technologies, Ashford, Ireland, ^3^Lionbridge Technologies, Minneapolis, Minnesota, USA

*Journal of Patient-Reported Outcomes 2026*, **10(Suppl 1)**:2058

### Aims

This study explores the potential of Generative AI in streamlining the dual forward translation (FT) Reconciliation process, with a focus on enhancing efficiency and accuracy. Our primary objective is to evaluate AI’s capability in detecting inconsistencies across target translations and selecting the ‘best-of-both’ options, while leveraging any available Concept Elaborations, thus optimizing reconciliation practices within Linguistic Validation (LV) projects.

### Methods

Following an initial literature review on the topic, we conducted an in-depth analysis of the GenAI-created Dual FT Reconciliation outputs from various Clinical Outcome Assessment instruments, comparing them with those performed by human experts. AI prompts were customized to generate outputs suitable for integration into our existing tools, as well as in line with the latest COA industry standards and established practices.

### Results

Preliminary findings indicate that AI-assisted Reconciliation significantly reduces the time required for the process while maintaining a comparable level of accuracy to traditional human-led reconciliation. The results presented focused on both efficiency (time saved) and accuracy (quality) metrics. Additionally, our research highlighted the importance of maintaining a human-in-the-loop approach to ensure the quality and reliability of AI-generated outputs.

### Conclusion

The integration of GenAI in dual FT reconciliation demonstrates promising potential to enhance efficiency without compromising accuracy. Best practices for balancing AI automation with human oversight have been identified, underscoring the necessity of human intervention in critical stages to ensure the highest quality of outputs. These findings support the feasibility of using GenAI in real LV projects, with significant implications for improving workflow efficiency in clinical research settings.

## 2059 GenAI in Action: A Study on the Efficacy of AI Comparative Review Output

Melinda Johnson^1^, Stephanie Casale^2^, Karolina Elizondo Jimenez^3^, Elisabet Sas Olesa^4^

^1^Lionbridge, Cary, North Carolina, USA, ^2^Lionbridge Technologies, Alanson, Michigan, USA, ^3^Lionbridge Technologies, Minneapolis, Minnesota, USA, ^4^Translating and Conference Interpreting, Lionbridge Technologies, Ashdown, Ireland

*Journal of Patient-Reported Outcomes 2026*, **10(Suppl 1)**:2059

### Aims

This case study explores the efficacy of GenAI Comparative Review output in ongoing Linguistic Validation (LV) Projects. This study is a continuation of research conducted to further test and quantify the impact of a GenAI prompt designed to compare original assessment items with back translated items in order to determine conceptual equivalence. The initial study contained a sample size of five (5) Indo-European languages with a word count of 1000 words (~100 items). This further study expands to longer-tail languages to ensure that less commonly localized languages fair as well as the original set.

### Methods

A prompt was developed for input into a secure GenAI engine with the goal of identifying discrepancies between original assessment and correlated back translation segments. The prompt requests that GenAI define that discrepancy in a way that would help the linguists involved to update and refine the existing forward translation to best reflect the original concepts. The outputs are compared to determine how often discrepancies are found by the GenAI engine versus the human resources and assessed for clarity of the explanations provided.

### Results

The initial results are promising, with clear, concise descriptions of original assessment and back translation discrepancies at an overall preliminary accuracy rate of 97%, matching 84% of feedback when compared to human output, and in 12% of segments, finding discrepancies not noted in the human output. In 3%, human output found potential discrepancies that AI noted as equivalent due to the usage of synonyms or localization changes.

### Conclusion

GenAI shows great promise in detecting and describing discrepancies brought to light through the process of Comparative Review, even for more rarely localized languages and locales. In a time-restricted process, utilizing AI for this intermediate step could make the difference in timely, cost-effective documentation in the clinical trial process.

## 2060 Patient activation is associated with positive attitudes toward pain medications

Richard Skolasky^1^, Ji Won Lee^2^, Janiece Taylor^3^

^1^Johns Hopkins University, Baltimore, Maryland, USA, ^2^Columbia University School of Nursing, New York, New York, USA, ^3^Johns Hopkins University School of Nursing, Baltimore, Maryland, USA

*Journal of Patient-Reported Outcomes 2026*, **10(Suppl 1)**:2060

### Aims

This study determined the association between patient activation and attitudes toward pain medication among patients with chronic low back pain (cLBP). We sought to understand whether those with high versus low activation demonstrated differences in opioid use within 30 days, pain-related disability, and beliefs about pain and pain medication. We hypothesized that patients with low activation would have maladaptive beliefs about pain medications and how to self-manage their pain.

### Methods

This was a cross-sectional study of 1055 patients evaluated at an academic orthopaedic department for degenerative spine disease or adult spinal deformity from Aug 2019 to Dec 2023. Patients were eligible if they: were 18 years or older or met the definition of cLBP and were excluded if they had recent spinal surgery, trauma, tumor, or infections since this would acutely exacerbate their existing spinal disease states. We evaluated patient activation using the Patient Activation Measure. We examined opioid medication use by asking patients if they were taking no, daily, or some medications in the last 30 days. We assessed disability using the Oswestry Disability Index. We evaluated attitudes toward pain medication using the Short Barriers Questionnaire-Taiwan (S-BQT).We compared opioid use and disability scores by patient activation and performed multivariate logistic regression to obtain the odds of experiencing a barrier related to each S-BQT subscale depending on activation status.

### Results

Mean activation score was 69.9 ± 19.1. Patients with low activation were more likely to report using opioid medication (35% versus 29%) and to endorse greater disability (48 ± 16 versus 42 ± 16). Patients with high activation were more likely to disagree with the belief that 1) pain medication cannot control their pain (OR 1.50, 95% CI 1.17–1.94), 2) good patients avoid talking about pain (OR 1.61, CI 1.17–2.21), and 3) pain medication should be saved (OR 1.28, CI 1.08–1.89). P<.05 for all.

### Conclusion

This study demonstrates that high activation was associated with lower odds of 30-day opioid use and disability and higher odds of having adaptive pain medication beliefs. Interventions targeting patient activation may improve pain medication beliefs and pain among patients with cLBP.

**Table 1 (abstract 2060):**
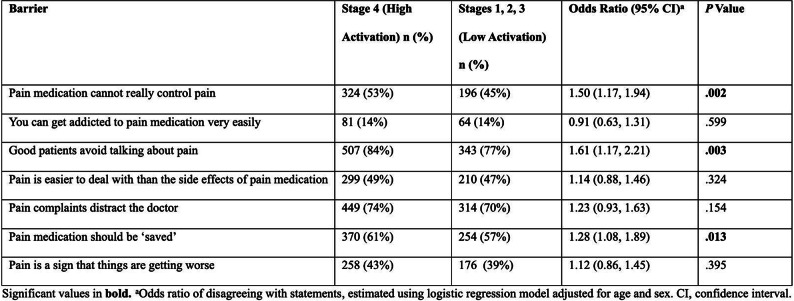
Multivariate Logistic Regression Results Showing the Odds of Agreement With Each of the S-BQT Subscales Depending on the Level of Patient Activation (High Versus Low)

## 2061 Searching Smarter: Using Linguistics to Improve Symptom Reporting for Children with Cancer

Hannah Wani^1^, Bin Jeong^1^, Stacey Crane^1^

^1^University of Texas Health Science Center at Houston, Houston, Texas, USA

*Journal of Patient-Reported Outcomes 2026*, **10(Suppl 1)**:2061

### Aims

Children with cancer often experience symptoms that are overlooked or inadequately managed. To improve symptom assessment during clinical trials, the National Cancer Institute sponsored development of the Pediatric PRO-CTCAE (Patient-Reported Outcomes version of the Common Terminology Criteria for Adverse Events). This tool captures 62 different symptomatic toxicities through 130 questions, but clinicians typically implement the Pediatric PRO-CTCAE by pre-selecting which symptoms to assess, limiting children’s ability to report every symptom experienced and resulting in incomplete data. The Smart Pediatric Oncology Tracker of Symptoms (SPOTS) is a web-based interface for the Pediatric PRO-CTCAE, designed to overcome this limitation. With SPOTS, children and/or their parents can easily identify which symptoms the child is experiencing, ensuring all relevant Pediatric PRO-CTCAE questions are completed and allowing for comprehensive symptom reporting. This project aimed to develop a search feature for SPOTS that enables children as young as seven (and their parents) to search for, identify, and report symptoms using colloquial and child-friendly language.

### Methods

To identify the full range of symptom-related terms included in the Pediatric PRO-CTCAE, sources such as adult and child thesauruses, children’s health websites, child and adolescent patient-reported outcome tools, websites devoted to slang language, and artificial intelligence tools were consulted. This process resulted in the creation of a comprehensive, colloquial, and child-friendly linguistic taxonomy, with terms mapped to each Pediatric PRO-CTCAE symptom. This taxonomy was then incorporated into SPOTS through a search (i.e. lookup) feature.

### Results

The resulting linguistic taxonomy comprises 5,425 terms mapped to 62 symptoms in the Pediatric PRO-CTCAE, averaging 87 terms per symptom. Pain had the highest number of associated terms, with a total of 230.

### Conclusion

Preliminary pilot testing was conducted at three local sites with pediatric cancer patients and their parents and supported the usability of the search feature and the linguistic taxonomy. Ongoing evaluation of symptom searches that did not yield satisfactory matches with a Pediatric PRO-CTCAE term will inform future refinement and expansion of the taxonomy.

## 2062 Functional constipation RCT: MIC/MCID application bias

Shaofen Xu^1^, Zhengkun Hou^1^, Feng-bin Liu^1^

^1^Guangzhou University of Chinese Medicine, No. 12, Ji Chang Road, Baiyun District, Guangzhou, China

*Journal of Patient-Reported Outcomes 2026*, **10(Suppl 1)**:2062

### Aims

In the clinical interpretation of Patient-Reported Outcomes (PROs) data, differentiating statistical significance from clinical relevance remains a key challenge. Current studies often exhibit terminological confusion, especially regarding “minimal important change” (MIC) and “minimal clinically important difference” (MCID). This study aimed to evaluate the inclusion and interpretation of MIC/MCID values in randomized controlled trials (RCTs) of functional constipation interventions.

### Methods

A cross-sectional study was conducted, searching PubMed, EMBASE, and Cochrane for RCTs on functional constipation interventions, with no restrictions on interventions or outcome measures.

### Results

Among 130 eligible studies, only 11 explicitly applied MIC/MCID, with most focusing on MIC. Although 83.3% of implemented MIC values aligned with references, 16.7% of thresholds differed. Notably, 3 studies that reported “clinical important differences” were actually about MIC.

### Conclusion

Terminological mix-ups and the use of unvalidated metrics without proper validation violate FDA’s PRO guidelines. Such issues lead to clinical interpretation differences, potentially affecting treatment decisions, highlighting the urgent need for standardized MIC/MCID application in functional constipation RCTs.

## 2064 Financial Insecurity and Discrimination Are Associated with Patient-Reported Quality of Life in Patients with Lupus

Heather Gold^1^, Yi Li^1^, Peter Izmirly^1^, Jill Buyon^1^, Mala Masson^1^, Amit Saxena^1^, H. Michael Belmont^1^, Chung-E Tseng^1^

^1^NYU Langone Health, New York, New York, USA

*Journal of Patient-Reported Outcomes 2026*, **10(Suppl 1)**:2064

### Aims

Lupus manifestations affect patient-reported outcomes (PROs) such as physical function, fatigue, and pain. Other work suggests economic insecurity is associated with worse PRO scores, while discrimination can have a chronic effect on health. This study evaluated the association of PROs with individual and area-based socioeconomic factors and lupus-related clinical variables.

### Methods

Patients from our cohort (n=272) diagnosed with systemic lupus erythematosus using standard international classification criteria were surveyed using measures from the NIH PROMIS for physical and cognitive function, pain interference, fatigue, anxiety, sleep disturbance, and depression. Analyses included demographics, financial/material insecurity (e.g., housing, finances, healthcare, job), Area Deprivation Index, and clinical data on disease activity (cutaneous, nephritis, arthritis) and immunosuppressive medications. Everyday Discrimination Scale quantified the potential impact of discrimination (never vs ever experienced 9 discrimination types, then summed across types). Multivariable linear regression models of PROs included clinical and socioeconomic factors.

### Results

The cohort was 90% female, 28% African American/Black, 17% Asian, and 39% white, with an average age of 40 years. Multivariable models showed that experiencing financial insecurity was associated with significantly worse scores on all PROs, from 4–7 points worse (p<0.01). Each additional point on the Everyday Discrimination Scale was associated significantly with 1 point worse on physical and cognitive function scores, 1 point higher on sleep disturbance, depression, and anxiety, and 2 points higher on fatigue on average (p<0.05). Finally, experiencing disease activity was significantly associated with lower physical function and higher sleep disturbance, depression, and anxiety (p<0.05). (Table).

### Conclusion

Experiencing financial insecurity was associated with clinically important negative impacts in function and symptom PROs; everyday discrimination consistently significantly negatively affected PRO scores; and disease activity was negatively associated with 4 of the 7 measures. Understanding the full experience of patients with lupus, beyond their clinical manifestations, may help identify approaches to support patients for improved function and reduced symtpoms.

**Table (abstract 2064):**
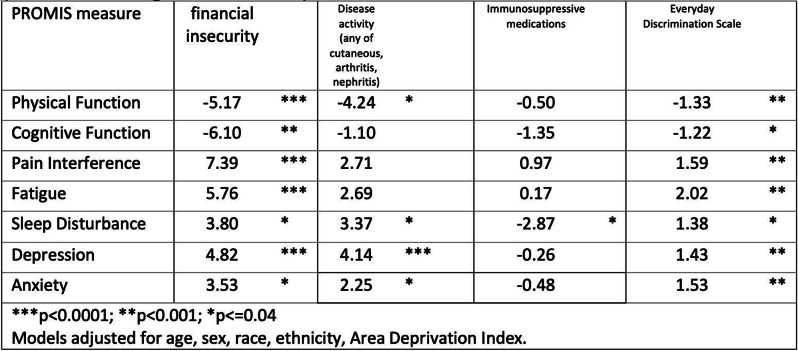
Multivariable linear regression analysis of patient-reported outcome measures (coefficient & significance level), n = 272

## 2066 Traditional Chinese Medicine Injections for Pain Relief in Cancer Patients:A Secondary Analysis of a Randomized Clinical Trial

Mingyan Zhang^1^, Shizhu Li^2^, Qiuling Shi^2^

^1^Master of Public Health, Chongqing Medical University, Chongqing, China, ^2^Chongqing Medical University, Chongqing, China

*Journal of Patient-Reported Outcomes 2026*, **10(Suppl 1)**:2066

### Aims

Pain is one of the most common symptoms of malignant tumors and a frequent adverse effect during radiotherapy and chemotherapy,severely affecting patients’ quality of life.This study aims to evaluate the potential efficacy of Traditional Chinese Medicine (TCM) injection for mitigating pain,with the goal of improving HRQoL in cancer patients.

### Methods

This secondary analysis derives from five multicenter,parallel-group randomized controlled trials conducted from 2014 to 2017,wherein participants completed follow-up assessments for a maximum duration of 6 months. A total of 1,285 patients (mean age: 58.3 years) with one of five major cancer types (lung, liver, cervical, head and neck, and gastrointestinal cancers) were enrolled. Participants underwent 1:1 randomization to either: (1) control group receiving standard-of-care (SOC) regimen for the target malignancy, or (2) intervention group receiving SOC with adjunct Traditional Chinese Medicine (TCM) injection therapy. The final analysis included 640 participants in the control group and 645 in the intervention group.Pain was assessed repeatedly throughout treatment and follow-up using the MD Anderson Symptom Inventory—Traditional Chinese Medicine module (MDASI-TCM).Generalized Estimating Equations (GEE) were used to analyze the main effects of treatment and the interaction between treatment and time.

### Results

The main effects model showed significantly lower pain scores in the intervention group compared to the control group(β = -0.179, 95% CI [-0.294, -0.064], p = 0.002).Pain severity also showed a significant decline over time across both groups (β = -0.084, 95% CI [-0.137, -0.031], p = 0.002). Notably, a significant time-by-treatment interaction was observed (β = -0.166, 95% CI [-0.272, -0.060], p = 0.002).

### Conclusion

This study demonstrates that TCM injection,when used as an adjunctive therapy in cancer patients receiving chemoradiotherapy,can significantly improve pain control. The observed time-dependent analgesic effect suggests a potential cumulative benefit, providing evidence to support the integration of TCM injections into supportive care strategies for cancer-related pain management.

**Table 1 (Abstract 2066a):**
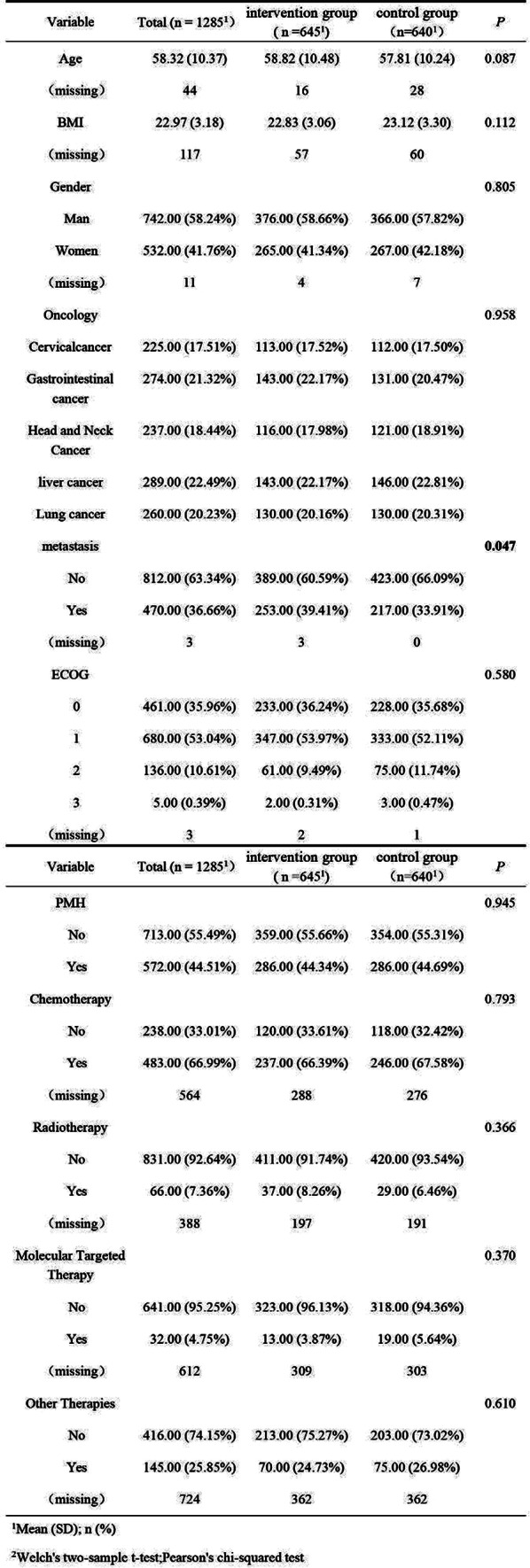
Traditional Chinese Medicine Injections for Pain Relief in Cancer Patients: A Secondary Analysis of a Randomized Clinical Trial

**Fig. 1 (abstract 2066b):**
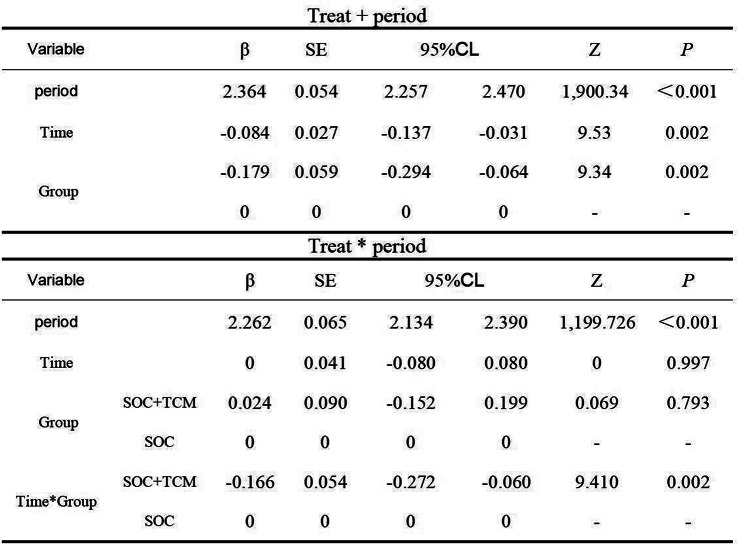
Traditional Chinese Medicine Injections for Pain Relief in Cancer Patients: A Secondary Analysis of a Randomized Clinical Trial

**Fig. 1 (abstract 2066c):**
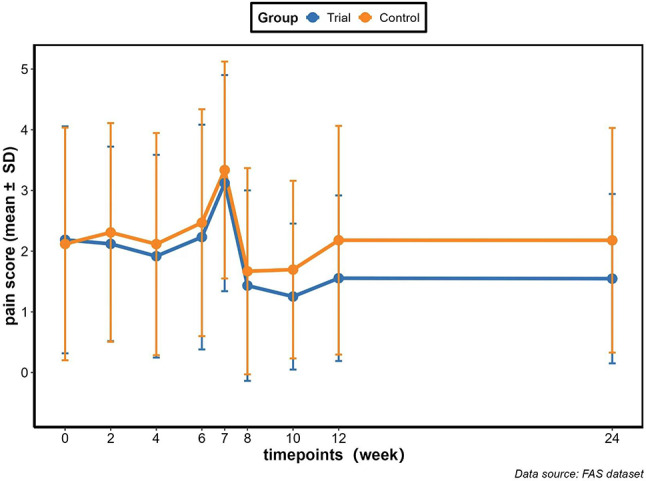
Traditional Chinese Medicine Injections for Pain Relief in Cancer Patients: A Secondary Analysis of a Randomized Clinical Trial

## 3013 Review and Analysis of Patient-Reported Fatigue Label Claims

Iyar Mazar^1^, Tori Brooks^2^, Jarjieh Fang^3^, Jillian Lusk^4^, Magdalena Harrington^5^

^1^Pfizer, Boston, Massachusetts, USA, ^2^Mapi Research Trust, Athens, Georgia, USA, ^3^Pfizer, New York, New York, USA, ^4^Pfizer, Pittsburgh, Pennsylvania, USA, ^5^Pfizer, Milford, New Hampshire, USA

*Journal of Patient-Reported Outcomes 2026*, **10(Suppl 1)**:3013

### Aims

1) Identify patient-reported outcome (PRO)-based fatigue claims in Food Drug Administration (FDA) and European Medicines Agency (EMA) product labeling, and 2) evaluate the interpretability of FDA fatigue labeling claims from a Clinical Outcome Assessment (COA) Scientist perspective.

### Methods

Three searches were conducted: 1) PROLABELS database, to identify the number of PRO-based fatigue labeling claims for products approved by FDA or EMA since 2014; 2) Medline, to identify trial publications for FDA products with fatigue labelling claims; 3) publicly-available FDA submission documents, typically only available for a product’s first approval, to identify additional sponsor evidence supporting a fatigue labeling claim.Each FDA label claim was evaluated against interpretability criteria aligning with the FDA Patient-Focused Drug Development Guidance, i.e., whether the claim reported: 1) statistical significance; 2) point estimate results; 3) meaningful within-patient change (MWPC). Publications were reviewed to identify any additional information supporting the interpretability of identified claims. FDA submission packages were reviewed to identify any additional sponsor-submitted evidence supporting the PRO labeling claims.

### Results

60 PRO-based fatigue labeling claims were identified for 34 products (27 EMA and 7 FDA). Based on the data available in FDA product labeling, none of the fatigue claims met all three of the pre-specified interpretability criteria. Eleven of the 40 trial publications reviewed provided additional information meeting all three of the pre-specified interpretability criteria. Thirteen publications met 2 of the criteria, 2 publications met 1 of the criteria, and 14 met none of the criteria. Three of the 7 FDA-approved products included a PRO-based fatigue labeling claim in their initial approval: Ultomiris, Rinvoq, and Pyrukynd. Information in both the submission packages and trial publications for these products included additional evidence beyond what is reflected in labeling claims.

### Conclusion

Although fatigue is a common symptom experienced across health conditions and reported as important to patients, fatigue labeling claims are infrequent and not interpretable from a COA Scientist perspective. Future research is needed to evaluate if and how other stakeholders (e.g., patients, healthcare providers) utilize and interpret these claims. Efforts to replicate these methods for the concept of pain are ongoing.

## 3015 Evaluating agreement metrics used in analytical validation: A simulation study for a digital measure of step count

Gerasimos Dumi^1^, Paolo Eusebi^2^, Dara O’Neill^3^, Aleksandra Sjöström-Bujacz^4^

^1^Patient-Centered Solutions, IQVIA, Athens, Greece, ^2^Patient-Centered Solutions, IQVIA, Milan, Italy, ^3^Patient-Centered Solutions, IQVIA, Barcelona, Spain, ^4^Patient-Centered Solutions, IQVIA, Stockholm, Sweden

*Journal of Patient-Reported Outcomes 2026*, **10(Suppl 1)**:3015

### Aims

Performance of algorithms for transforming raw sensor data into physiological/behavioral metrics is assessed via analytical validation. This consists of establishing agreement between the digital measure and a reference measure representing a “gold standard”. Several mobility-related parameters are count-based, thus commonly used continuous-based agreement metrics may be inappropriate. This study is aimed at (i) evaluating the performance of concordance correlation coefficient (CCC) based on a Poisson generalized linear mixed model (GLMM) versus a linear mixed model (LMM) as an agreement metric for count data and (ii) comparing CCC with alternative approaches originally designed for continuous data such as Pearson correlation and t-test and with gold standard metrics such as mean absolute percentage error (MAPE) to assess inference consistency under different levels of agreement (<0.7 and >0.7).

### Methods

A GLMM (Poisson) with random subject and fixed measurement method effects was used to simulate step counts (500 datasets) assuming varying sample sizes (80, 200, 1000), and CCCs (0.999, 0.879, 0.785, 0.687, 0.594). Performance of CCCGLMM and CCCLMM was assessed using absolute bias (AB) and mean square error (MSE). Pearson correlation, paired t-test, and MAPE were compared with CCCLMM and CCCGLMM based on the following estimate: percentage of times (denoted as P1) that each metric provided supporting evidence of agreement (correlation>0.7, p-valuet-test>0.05, MAPE<0.05, CCC>0.7) across 500 datasets.

### Results

(i) CCCGLMM had a better performance (i.e., lower AB and MSE) than CCCLMM, especially when true CCC<0.7. (ii) When true CCC>0.7, CCCGLMM, CCCLMM, Pearson correlation, MAPE provided supportive evidence of agreement in most datasets (P1: 94.0%-100.0%, 91.6%-100.0%, 93.0%-100.0%, 100.0%, respectively), while t-test provided variable results (P1 range: 42.2%-93.4%). As expected, when true CCC <0.7, all the metrics provided supportive evidence of agreement less frequently (P1: 0.0%-30.4% for CCCGLMM, 0.0%-42.4% for CCCLMM, 0.0%-48.2% for Pearson correlation) except for MAPE and t-test.

### Conclusion

Pearson correlation or t-test should be used only as a descriptive assessment of two measures. CCCGLMM is an appropriate metric for count-based parameters, accounting for the variability between subjects and measurement methods. It should be complemented with MAPE and preferred over CCCLMM, unless data are near normal or expected agreement is >0.9.

**Fig. 1 (abstract 3015):**
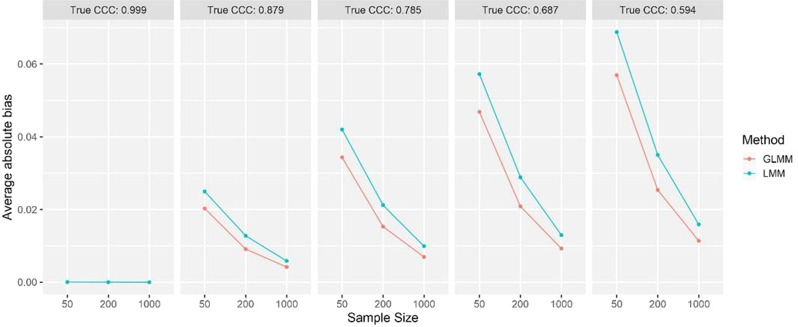
Average Absolute bias for CCCG_LMM_ and CCC_LMM_ under different scenarios

**Fig. 2 (abstract 3015):**
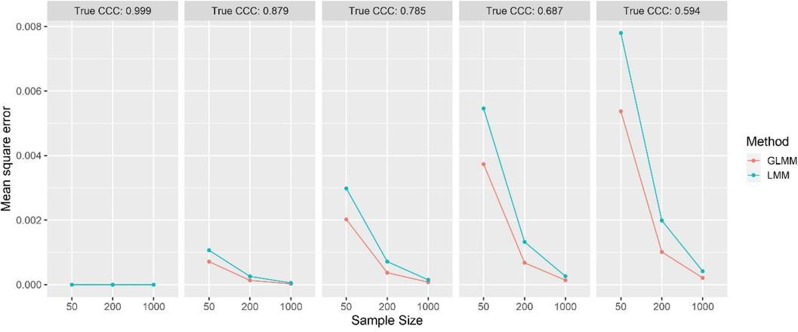
Mean Square Error for CCCG_LMM_ and CCC_LMM_ under different scenarios

**Fig. 3 (abstract 3015):**
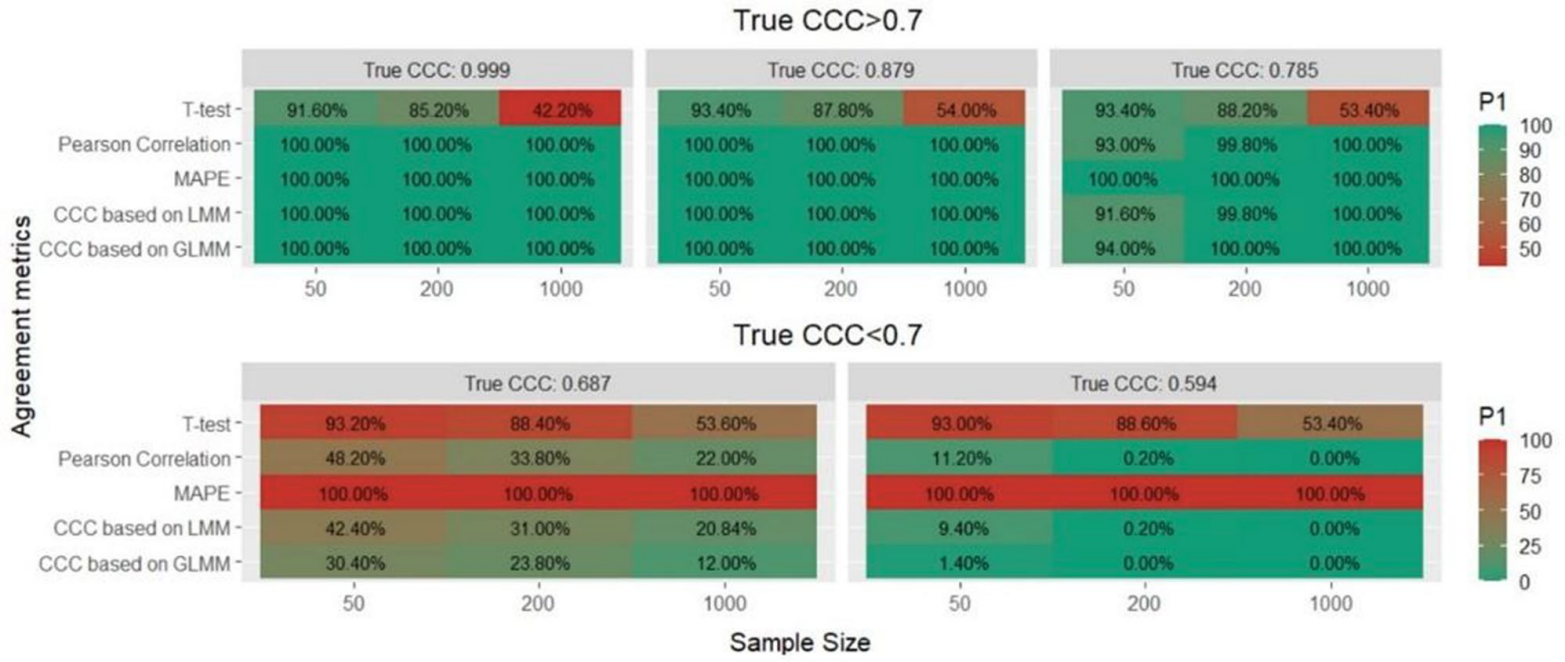
Heat map for P1 for each agreement metric under different scenarios

## 3017 Predictors in infancy of disease-specific quality of life in adolescents born with esophageal atresia - a nationwide prospective observational study in Sweden

Michaela Dellenmark-Blom^1,2^, Sara Persson^1,2^, Elisabet Gustafsson^3,4^, Erik Omling^5^, Colin Reilly^2^, Helene Engstrand Lilja^6^, Jan F. Svensson^6,7^, Linus Jönsson^1,2^, Vladimir Gatzinsky^1,2^, Niclas Högberg^3,4^, Kate Abrahamsson^1,2^, Tomas Wester^6,7^, Elin Öst^6,7^

^1^Department of Pediatric Surgery, Queen Silvia Children’s Hospital, Sahlgrenska University Hospital, Gothenburg, Sweden, ^2^Institute of Clinical Sciences, Department of Pediatrics, Queen Silvia Children’s Hospital, Gothenburg, Sweden, ^3^Department of Pediatric Surgery, University Children’s Hospital, Uppsala, Sweden, ^4^Department of Women’s and Children’s Health, Uppsala University, Uppsala, Sweden, ^5^Department of Pediatric surgery, Skane University Hospital, Lund University, Lund, Sweden, ^6^Department of Women’s and Children’s Health, Karolinska Institutet, Stockholm, Sweden, ^7^Department of Pediatric Surgery, Karolinska University Hospital, Stockholm, Sweden

*Journal of Patient-Reported Outcomes 2026*, **10(Suppl 1)**:3017

### Aims

Esophageal atresia (EA) is a rare congenital malformation characterized by discontinuity of the esophagus. Today, survival rates of children with repair of EA reach 90% in high income countries. However, children with EA face long-term aerodigestive morbidity and are recommended life-long follow-up care. In 2018, a set of age-specific disease-specific Quality of Life (QOL) questionnaires for children with EA was developed in Sweden and Germany according to current standards of patient-reported outcome measurements (the EA-QOL questionnaires). These are currently being psychometrically evaluated in 23 countries/languages. Following its early establishment in Sweden, this study aimed to apply the EA-QOL questionnaire in adolescents with EA, to determine if and which characteristics in infancy predict disease-specific QOL around 15 years of age.

### Methods

In an ongoing nationwide Swedish prospective study of 3 years (Ethical approval 2021-04051), adolescents with EA aged 15–16 years and one parent completed the EA-QOL questionnaire consisting of four subscales: Eating, Body perception, Social Relationships and Health & Emotional Well-being at follow-up. Responses were transformed to a linear scale of 0 (worst) to 100 (best) QOL-scores. Clinical data were extracted from patient medical records. Data were analyzed using descriptive statistics and linear regression. Significance level was p<0.05.

### Results

Eighty-eight participants, 42 adolescents and 46 parents, completed the EA-QOL questionnaire. Female sex predicted lower scores of Body perception (child-report, p=0.041) and Health & Emotional Well-being (child-report,p=0.042). Low birth weight was a predictor of reduced scores of total EA-QOL (parent-report, p=0.036), Body perception (child-report, p=0.031) and Eating (parent-report, p=0.045). Gestational age at birth was associated with lower scores on three subscales, p<0.05 (see Figure). “No primary anastomosis” (parent-report, p=0.0034) and a gastrostomy at age 1 were associated with reduced Social relationships scores (parent-report, p=0.0083). More anesthesias at age 1 predicted lower total scores and scores on three subscales (p<0.05).

### Conclusion

In a nationwide Swedish study, preliminary results suggest that it is possible to identify predictors within infancy of disease-specific QOL in adolescents aged 15–16 years with EA. Female sex, prematurity, low birth weight, a gastrostomy and more anesthesias at age 1 predict reduced levels of disease-specific QOL. This information can help develop preventative care strategies.

**Fig. 1 (abstract 3017):**
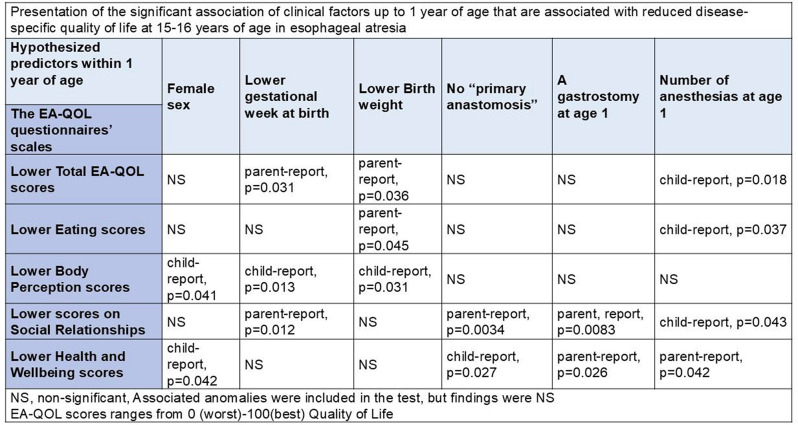
Predictors in infancy of disease-specific quality of life in adolescents born with esophageal atresia - a nationwide prospective observational study in Sweden

## 3019 Psychometric Validation of the Eating Behavior and Appetite Questionnaire (EBAQ) in Adults with Obesity or with Overweight and Obesity-related Complications

Chisom Kanu^1^, Miriam Kimel^2^, Claudine Clucas^3^, Iris Goetz^4^, Lisa M. Neff^5^, Kristina Boye^6^, Hayley Karn^3^

^1^Eli Lilly & Company, Indianapolis, Indiana, USA, ^2^Evidera, Inc., Wilmington, North Carolina, USA, ^3^Evidera, Ltd, London, UK, ^4^Eli Lilly and Company, London, UK, ^5^Eli Lilly and Company, Chicago, Illinois, USA, ^6^Eli Lilly and Company, Greenwood, Indiana, USA

*Journal of Patient-Reported Outcomes 2026*, **10(Suppl 1)**:3019

### Aims

This study evaluated the psychometric properties of a new patient-reported outcome (PRO) measure, the Eating Behavior and Appetite Questionnaire (EBAQ) which assesses concepts important to patients with obesity or overweight.

### Methods

Adults with obesity or with overweight and an obesity-related complication completed two web-based surveys. The first survey included the 21-item EBAQ, Control of Eating Questionnaire (CoEQ), Food Cravings Questionnaire-Trait-reduced (FCQ-T-r), Impact of Weight on Quality of Life-Lite Clinical Trials Version (IWQOL-Lite-CT), and Patient Global Impression of Severity (PGIS) items for appetite, eating control, cravings, and overall eating behavior. The EBAQ and PGIS items were completed in the second survey two weeks later. Exploratory factor analysis (EFA) was used to determine the factor structure. Reliability (internal consistency, test-retest) and validity (convergent and known groups) were assessed.

### Results

The mean age (SD) of 120 study participants was 53.0 (11.8) years. Majority were female (66.7%), White (62.5%) and had lived with obesity/overweight for ≥11 years (63.4%). EFA results supported a two-factor structure, with items loading strongly (≥0.5) on the factors corresponding to appetite or eating behavior (inter-factor correlation 0.57). The remaining four items were dropped because they had poor loadings or did not load as hypothesized. The subscale and total scores of the 17-item EBAQ showed good internal consistency (Cronbach’s alpha 0.84 to 0.92), and good test-retest reliability from Baseline to the 2-week follow up (ICCs: 0.84 to 0.89). The EBAQ demonstrated convergent validity by generally showing large correlations with the CoEQ craving subscales, FCQ-T-r score, and PGIS items relating to appetite, eating control, cravings and overall eating behavior, while having smaller correlations with less similar PROs. Known-groups validity was supported as participants that frequently had a well-controlled appetite (PGIS Appetite), frequently felt in control of their eating (PGIS Eating Control), had fewer food cravings (PGIS Cravings) and better eating habits (PGIS Overall Eating Behavior) had significantly higher EBAQ subscale and total scores than those with more appetite, less control, more cravings and poor eating habits.

### Conclusion

Results support that the 17-item EBAQ is a reliable and valid measure that can be used in observational studies and clinical trials for obesity.

## 3021 Quality of life in the rural highlands of Peru

Fiorella Guerrero^1^, Kevin Weinfurt^1^, Jessica Kramer^2^, Stella Maris Hartinger Peña^3^, Daniel Mäusezahl^4^

^1^Duke University, Durham, North Carolina, USA, ^2^OTR/L, University of Florida, Gainesville, Florida, USA, ^3^Universidad Peruana Cayetano Heredia, Lima, Peru, ^4^Swiss Tropical and Public Health Institute, Kreuzstrasse, Switzerland

*Journal of Patient-Reported Outcomes 2026*, **10(Suppl 1)**:3021

### Aims

Cohort studies are essential in public health research, providing insights into disease etiology and the effects of exposures on health outcomes. The Peruvian Andes Multigenerational High Altitude Cohort (ALTO) was established for this purpose. The World Health Organization Quality of Life (WHOQoL-BREF), a quality-of-life instrument, was selected to describe the effects of new diseases from a patient perspective. This study examines the validity evidence of using the WHOQoL-BREF scores with a high-altitude Andean rural population.

### Methods

Using cross-sectional data from the ALTO cohort, we assessed structural validity, internal consistency, and aspects of construct validity. We examined item responses, missing data, the polychoric correlation matrix, and Cronbach’s alpha for each domain. We conducted a confirmatory factor analysis (CFA) for the original three-factor structure and examined factor loadings. To evaluate construct validity, we ran a one-way ANOVA to explain differences in the WHOQoL-BREF score domains with groups of sociodemographic factors (i.e., education, gender, marital status), and indicators of physical health and depression. We also examined the relationship between age and WHOQoL-BREF scores. Effect sizes were reported.

### Results

The sample comprises 3431 participants in rural Andean Peru, with a mean age of 39 years, 67.8% female, 61.2% married or cohabiting, and 80.5% with less than 10 years of formal schooling. Cronbach’s alpha indicated good reliability for the Physical domain and acceptable reliability for all other domains. CFA did not support a three-factor structure, with low factor loadings for items about leisure and negative feelings. Regarding construct validity, lower scores were observed for older versus younger participants, females versus males, and for those with lower education in all domains. The presence of a chronic health condition and depression were associated with lower scores in the Physical and Psychological domains, respectively.

### Conclusion

Findings on the factor structure suggest the WHOQoL-BREF may be better examined using a composite indicator model rather than a reflective indicator one. Still, we confirmed score differences across the groups tested, suggesting meaningful variance in respondents’ Physical and Psychological QoL. This study provides preliminary support for using the WHOQoL-BREF with this sample and potentially for other rural samples in Latin America.

## 3023 Understanding the natural history of cognitive impairment in people living with immune thrombotic thrombocytopenic purpura: qualitative perspectives of patients and their observers

Laura Mkumba^1^, Elizabeth Cieza^1^, Jada Green^1^, Fiorella Yvette Guerrero Calle^1^, Olivia Fernandez^1^, Kristin Byrne^1^, Dana Thompson^1^, Jamila Minga^1^, Brian Adkins^2^, Benjamin Tillman^3^, Toyosi Onwuemene^1^, Theresa Coles^1^

^1^Duke University, Durham, North Carolina, USA, ^2^UT Southwestern Medical Center, Dallas, USA, ^3^Vanderbilt University School of Medicine, Nashville, USA

*Journal of Patient-Reported Outcomes 2026*, **10(Suppl 1)**:3023

### Aims

Patients with the rare blood disorder, immune thrombotic thrombocytopenic purpura (iTTP), report cognitive function as a key health-related quality of life outcome. However, in iTTP clinical trials, validity evidence is lacking for clinical outcome assessments (COAs) of cognitive function. A comprehensive understanding of the natural history or change over time of cognitive function in patients with iTTP is needed to validate patient-centered COAs for iTTP clinical trials. This study focuses on the descriptions of the natural history of cognitive function from the perspectives of patients with iTTP and their observers.

### Methods

Purposive sampling was used to recruit 24 patient-observer dyads (N=48 participants) from three sites. Eligible patients were ≥ 16 years old, diagnosed with acquired iTTP, and able to speak and understand English. Eligible observers were ≥18 years old, close friends or family members who spent at least 12 hours a week with a patient with iTTP, and able to speak and understand English. Patients and observers engaged in individual, remote in-depth qualitative interviews eliciting information about cognitive capabilities and cognitive changes before the iTTP diagnosis, around the time of diagnosis, and during and between iTTP episodes. All participants completed a study -specific modified version of the 20-item Cognitive Change Index (CCI-20). Interviews were video and audio recorded and transcribed verbatim. Descriptive content analysis of the transcripts was conducted using NVivo software.

### Results

Data collection is ongoing and is expected to conclude by July 2025. Preliminary findings indicate that patients experience worsening episodic memory, concentration, and executive function after iTTP diagnosis.

### Conclusion

The findings from this study will enrich current knowledge about functional cognitive changes in patients with iTTP over time. The study will provide foundational evidence for selecting appropriate COAs with suitable recall periods to support iTTP clinical trial study design.

## 3025 Using the EORTC Item Library to assess patient-reported symptomatic adverse events: Development of the EORTC-PRO-AE inventory

Claire Piccinin^1^, Hayat Hamzeh^2^, Shaista Meer^2^, Dagmara Kuliś^3^, Alexandra Gilbert^2^

^1^Quality of Life Department, EORTC, Brussels, Belgium, ^2^Leeds Institute of Medical Research at St. James’s, University of Leeds, Leeds, UK, ^3^Quality of Life Department, EORTC Headquarters, Brussels, Belgium

*Journal of Patient-Reported Outcomes 2026*, **10(Suppl 1)**:3025

### Aims

A previous mapping of 950 European Organisation for Research and Treatment of Cancer (EORTC) Item Library items to the Common Terminology Criteria for Adverse Events (CTCAE) highlighted considerable coverage of adverse events (AEs) with 208 AEs linked to EORTC patient-reported outcome (PRO) items. These findings provided a framework for the current project, which aims to update the initial mapping by including recently developed items and refining the linking to capture symptomatic AEs only, selecting one item per symptomatic AE in case of multiple linked items. This is part of an ongoing project to create the EORTC-PRO-AE inventory, which will facilitate the use of EORTC items for symptomatic AE assessment.

### Methods

All new items (n=109) were mapped to the CTCAE following previously established linking rules and the full group of items (n=1059) was reclassified into symptomatic and non-symptomatic AEs to retain symptomatic AEs only. Pre-defined decision criteria were used to guide the initial selection of items per symptomatic AE based on content validity, wording, and translatability. The remaining symptomatic AEs with multiple linked items were then included in a modified online Delphi survey (consensus level 70%) wherein participants representing different interest holders were asked to vote for one preferred item to best capture each symptomatic AE.

### Results

Following the updated EORTC-CTCAE mapping and removal of non-symptomatic AEs, 177 symptomatic AEs remained, linked to 620 different items. Each AE was mapped to 1–44 items. Using the pre-defined decision criteria, one item was selected for 147/177 AEs. The remaining 30 AEs were included in the modified Delphi survey, with 2–4 pre-selected items for each AE. Consensus was achieved for all 30 AEs after 3 survey rounds (round 1: 12 AEs; round 2: 7 AEs; round 3: 11 AEs).

### Conclusion

These findings highlight the first 2 stages of development of the EORTC-PRO-AE inventory. The final stage (ongoing) is aimed at developing new EORTC items to cover missing symptomatic AEs. The addition of these missing items will finalise the EORTC-PRO-AE, a comprehensive inventory that will provide a recommended EORTC item for each included symptomatic AE, to support the measurement of patient-reported symptomatic AEs within clinical research and care.

## 3027 Smart Pediatric Oncology Tracker of Symptoms (SPOTS): Design and Development of a Web-Based Interface for the Pediatric PRO-CTCAE

Stacey Crane^1^, Jacqueline Castillo^1^, Cecile Nguyen^1^, Hannah Wani^1^, Jessica Wooden^1^, Karen D. Gibbs^1^, Laura Carter^1^, Andrew D. Miller^2^, Deevakar Rogith^1^

^1^University of Texas Health Science Center at Houston, Houston, Texas, USA, ^2^Indiana University Indianapolis, Indianapolis, USA

*Journal of Patient-Reported Outcomes 2026*, **10(Suppl 1)**:3027

### Aims

Toxicities from cancer treatments have a significant impact on the lives of children with cancer and can impact their ability to successfully complete therapy. The Pediatric Patient Reported Outcome Common Terminology Criteria for Adverse Events (Pediatric PRO-CTCAE) is an instrument developed to capture subjective toxicities via direct reports from children and parents. The Pediatric PRO-CTCAE allows for child self-/parent proxy-report of 62 different symptoms using 130 questions, but is currently implemented in research by asking about pre-selected toxicities of interest, limiting the number of symptoms that can be reported.To fully implement the Pediatric PRO-CTCAE, a user-friendly, child-centered interface is needed—one that children and their families can easily use at home or in the hospital. Smart Pediatric Oncology Tracker of Symptoms (SPOTS) is a novel web-based interface for the Pediatric PRO-CTCAE that was developed by this team. SPOTS provides a method for children and parents to naturally and systematically report all the symptomatic toxicities a child experiences. The aim of this presentation is to describe the formative development of SPOTS.

### Methods

SPOTS was developed alongside children with cancer and their parents using a combination of child-/human- computer interaction theory, the Task, User, Representation, and Function (TURF) Framework, and participatory co-design. Co-design sessions were used to establish the challenges experienced in communicating symptoms to pediatric oncology clinicians and imagine an ideal solution. Based on these insights, an initial version of SPOTS was created. Subsequent co-design sessions focused on refining this early version. A wireframe for the SPOTS prototype was then established, and usability testing was conducted with children with cancer and their parents. Lastly, a preliminary SPOTS prototype was developed and pilot tested with child–parent dyads across three pediatric oncology outpatient programs in Houston, TX.

### Results

29 children with cancer and their parents participated in the formative development of SPOTS. An 8-week longitudinal pilot test was conducted with 35 child with cancer/parent dyads, resulting in 896 reported symptoms.

### Conclusion

Pilot testing supported the usability of the preliminary SPOTS interface, but highlighted the need for additional features and refinement. Funding has been secured and further development is underway to enhance the SPOTS interface.

## 3029 Pre-Treatment Readiness Tool for Sarcoma

Marium Husain^1^, Hisham Alsharif^1^, James Chen^1^, Gabriel Tinoco^1^, David Liebner^1^

^1^Ohio State University James Comprehensive Cancer Center, Columbus, Ohio, USA

*Journal of Patient-Reported Outcomes 2026*, **10(Suppl 1)**:3029

### Aims

There is limited data on assessing patients’ social needs prior to starting treatment, specifically in sarcoma. Pre-treatment planning does not formally exist in medical therapy management for patients with sarcoma. This pre-treatment planning concept exists in other fields, like bone marrow transplant, and has led to improved outcomes. Our hypothesis is that implementation of a pre-treatment readiness tool improves outcomes in patients receiving systemic treatment for sarcoma.

### Methods

The Pre-Treatment Readiness Tool is a non-validated tool created in partnership with the OSU sarcoma social work team that assesses four domains of health: practical, family support, emotional and health care with a total of 24 psychosocial fields assessed, utilizing a 3-point Likert scale (no concerns at all, somewhat concerned, greatly concerned). We implemented the tool from January 1, 2018 through December 31, 2019. Clinical demographic data and the number and type of referrals made to ancillary services (e.g, housing, counseling) were collected. Data analyses performed were descriptive and cox hazard ratios: overall survival (OS) and progression-free survival (PFS). There is no control group in this report.

### Results

There were 109 patients that completed the pre-treatment readiness tool prior to the initiation of systemic chemotherapy. Most patients were older than age 40 (72.2%) and half of the cohort had advanced/stage 4 disease (50%). The older the patient, the more concerns were reported and subsequently more referrals were ordered (Figure 1). This was not associated with gender or stage. Patients of any age who reported emotional concerns had a poor OS (p = 0.030) (Figure 2).

### Conclusion

The implementation of a pre-treatment readiness tool identified important concerns amongst patients prior to starting chemotherapy, with emotional concerns associated with poor survival. This data needs to be further validated with a control group and prospectively to improve addressing patient needs before medical treatment for sarcoma.

**Fig. 1 (abstract 3029a, b):**
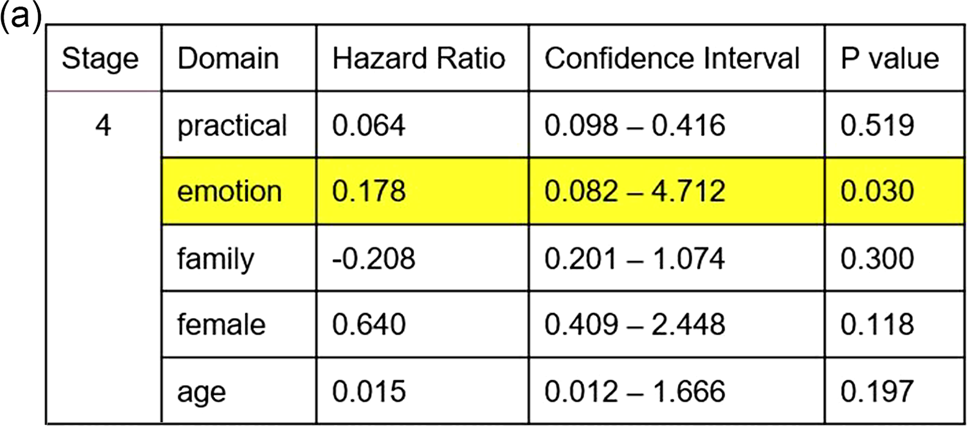

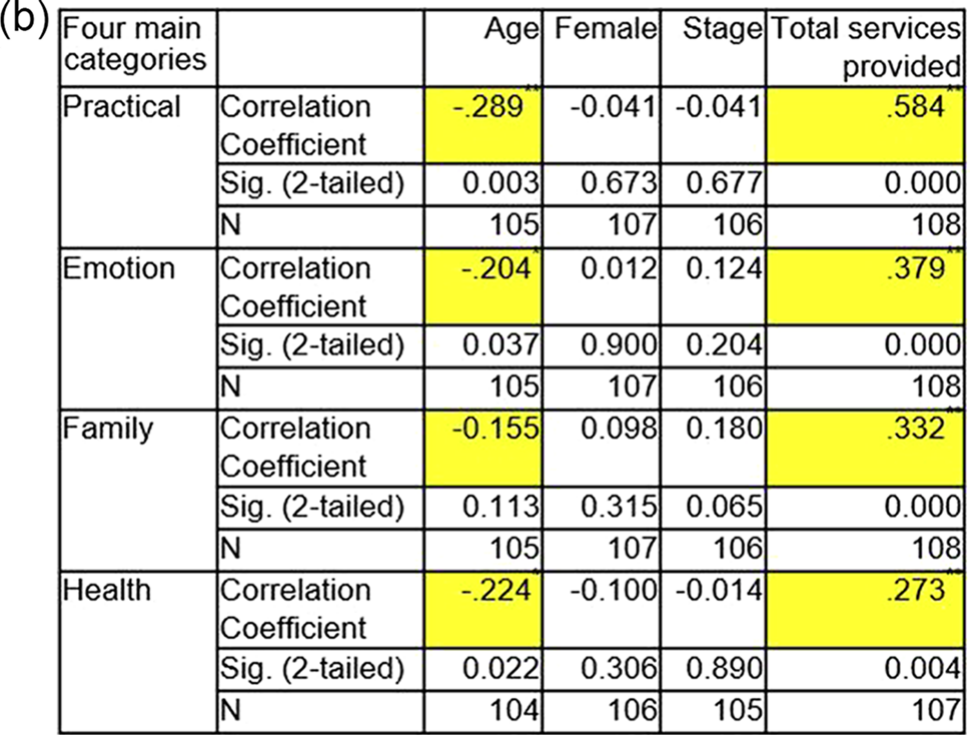
Pre-Treatment Readiness Tool for Sarcoma

## 3031 Test-retest reliability and concurrent validity of performance outcome measures in sarcopenia

Xiaodan Tang^1^, Maja Kuharic^1^, Jin-Shei Lai^1^, John Peipert^2^, Sara Shaunfield^1^, George Greene^1^, Chelsea Perschon^1^, Emilie Jaeger^1^, Mauricio Andrade^1^, Sofia Guzman^1^, Courtney Hurt^1^, Jack Guralnik^3^, David Cella^1^

^1^Northwestern University Feinberg School of Medicine, Chicago, Illinois, USA, ^2^University of Birmingham, Birmingham, UK, ^3^University of Maryland School of Medicine, Baltimore, USA

*Journal of Patient-Reported Outcomes 2026*, **10(Suppl 1)**:3031

### Aims

This study evaluated test-retest reliability and concurrent validity of five performance outcome measures (PerfOs) in older adults with sarcopenia. This work is part of the Northwestern University Clinical Outcome Assessment Team (NUCOAT) project, funded by the FDA, which aims to develop and validate clinical outcome assessments (COAs) of physical function for regulatory use.

### Methods

We assessed 121 participants with sarcopenia at baseline and at a 16-week follow-up using five PerfOs: the Short Physical Performance Battery (SPPB), Grip Strength, Timed Up and Go (TUG), 9-Hole Pegboard Dexterity Test, and 6-Minute Walk Test. A subsample of 45 participants completed repeat assessments (excluding the walk test) 7–21 days after baseline to assess test-retest reliability. Intraclass correlation coefficients (ICCs) were calculated for the full retest sample and the stable subgroup who reported no change in perceived difficulty performing that specific PerfO. To evaluate concurrent validity, we calculated Pearson’s product-moment correlations among the five PerfOs to assess whether they measured similar aspects of physical function at both time points. Grip Strength was analyzed separately by sex.

### Results

All five PerfOs demonstrated acceptable test-retest reliability (ICC > 0.70; see Table 1), except for the dominant-hand Pegboard test in the stable group (ICC = 0.68). ICCs were similar between the full and stable samples. For concurrent validity (Table 2), SPPB scores showed moderate (>0.4, acceptable evidence of validity) to high (>0.6) correlations with all PerfOs, particularly with TUG and the 6-Minute Walk Test, except for Grip Strength in male participants at baseline and female participants at follow-up. The Pegboard test showed moderate correlations with both TUG and the 6-Minute Walk Test. The 6-Minute Walk Test was highly correlated with TUG (-0.71 to -0.73) and moderately correlated with dominant-hand Grip Strength at follow-up (0.45). Grip strength demonstrated relatively low correlations with most other PerfOs, suggesting it may assess different components of physical function. Overall, correlations among the five PerfOs were slightly higher at follow-up than at baseline.

### Conclusion

These five PerfOs demonstrated adequate test-retest reliability and concurrent validity in older adults with sarcopenia. Further analyses using these PerfOs as anchors are planned to evaluate the construct validity of patient-reported outcomes.

**Table 2 (abstract 3031):**
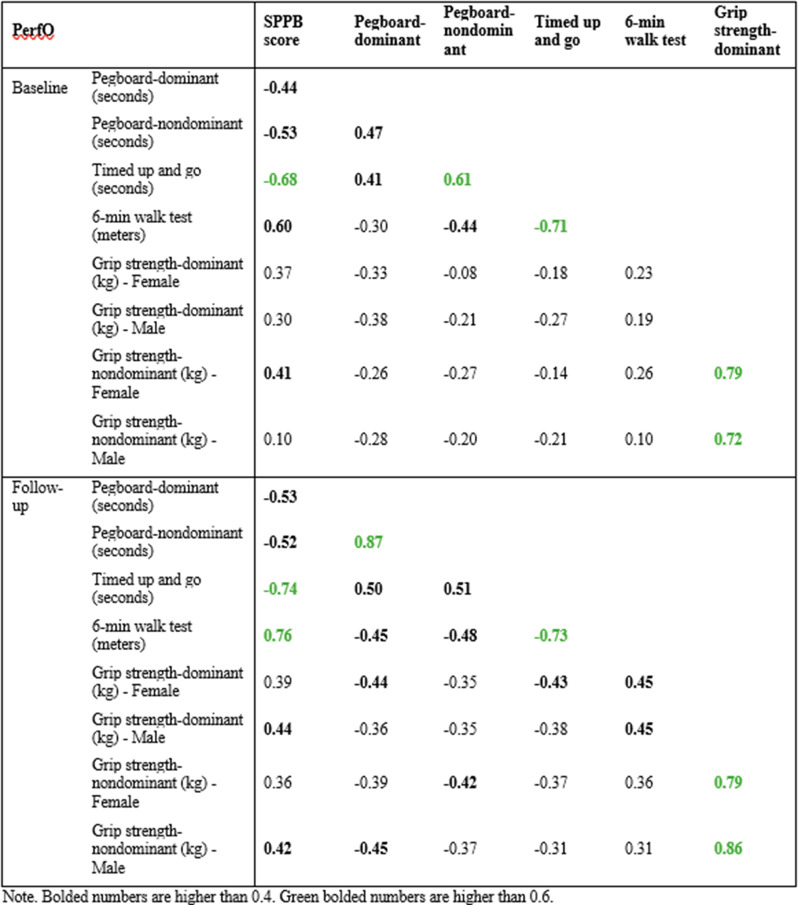
Concurrent validity among five performance outcome measures

**Table 1 (abstract 3031):**
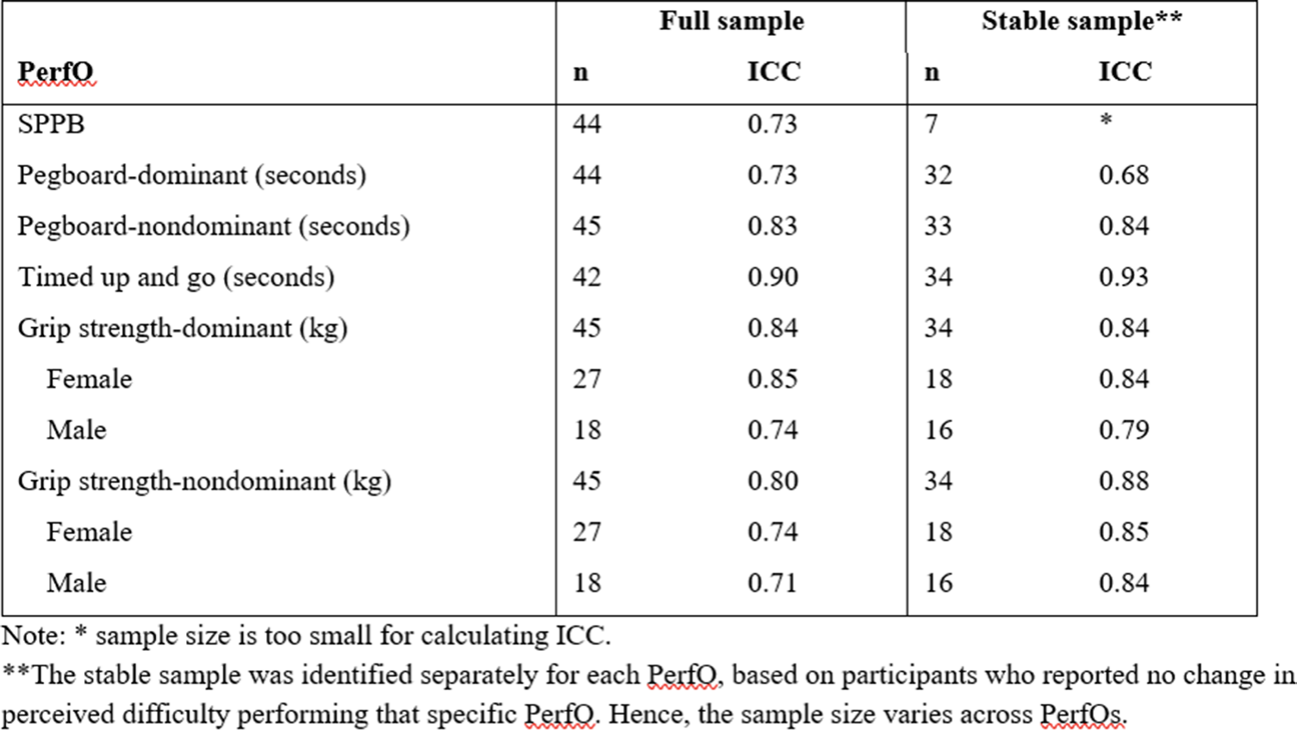
Test-retest reliability for four performance outcome measures

## 3033 Equivalence of Paper-Based and Smartphone-Based Patient-Reported Outcomes Using the Perioperative Symptom Assessment of Lung Surgery (PSA-Lung): A Randomized Crossover Equivalence Study

Hongfan Yu^1^, Qiuling Shi^1^, Xing Wei^2^

^1^State Key Laboratory of Ultrasound in Medicine and Engineering, College of Biomedical Engineering, Chongqing Medical University, Chongqing, China, ^2^Department of Thoracic Surgery, Sichuan Clinical Research Center for Cancer, Sichuan Cancer Hospital & Institute, Sichuan Cancer Center, Affiliated Cancer Hospital of the University of Electronic Science and Technology of China, Chendu, China

*Journal of Patient-Reported Outcomes 2026*, **10(Suppl 1)**:3033

### Aims

Electronic patient-reported outcomes (ePROs) are increasingly adopted for real-time symptom monitoring and deliver early warning to clinicians, yet equivalence validation with paper-based formats remains limited by selection bias. This study compares the equivalence between paper-based (P&P) and smartphone-based PSA-Lung instruments and evaluate patient preferences in perioperative lung surgery.

### Methods

A pragmatic clinical trial (PCT) was employed within crossover RCT using a within-subjects comparison of two administration modes (P&P and ePRO) and cohort study. Participants aged 18 years with scheduling lung surgery were enrolled from April 2021 to July 2021. A 9-item PSA-Lung instrument was evaluated, at per-surgery, postoperation daily until discharge, and twice a week for 4 weeks after discharge. The crossover was conducted pre-surgery and any day of postoperation with a time interval of 30 minutes. Equivalence was evaluated by using intraclass correlation coefficients (ICC) and Bland-Altman plots. Paired t-test was used to confirm carryover effect. Response rates were compared via generalized estimating equations (GEE).

### Results

PCT was divided into 74 patients in the crossover RCT and 339 patients in cohort study. In the crossover RCT, half of those was randomized to P&P and ePRO groups, with average age of 51.93 years and 33.78% male. Two modes showed high agreement (ICC: 0.78–0.95; Bland-Altman limits with 95%CI: -1.21 to 1.88) with no carryover effect (P>0.05). The GEE model demonstrated no significant differences in response rate (P=0.67). The cohort study revealed 71.7% (243/339) preferred ePROs, with an average age of 58.04 years and 40.12% male. Paper users had lower post-discharge compliance (P=0.003) and differed demographically (age, education level, comorbidities and hospitalization duration, P<0.001).

### Conclusion

The PSA-Lung demonstrated excellent equivalence in administration modes, supporting ePRO integration for perioperative symptom monitoring. Despite severe patient preference for smartphone, selection bias highlight the need for tailored ePRO implementation strategies in surgical practice. These findings advocate for ePRO adoption in surgical practice while addressing barriers to equitable engagement.

**Fig. 1 (abstract 3033a):**
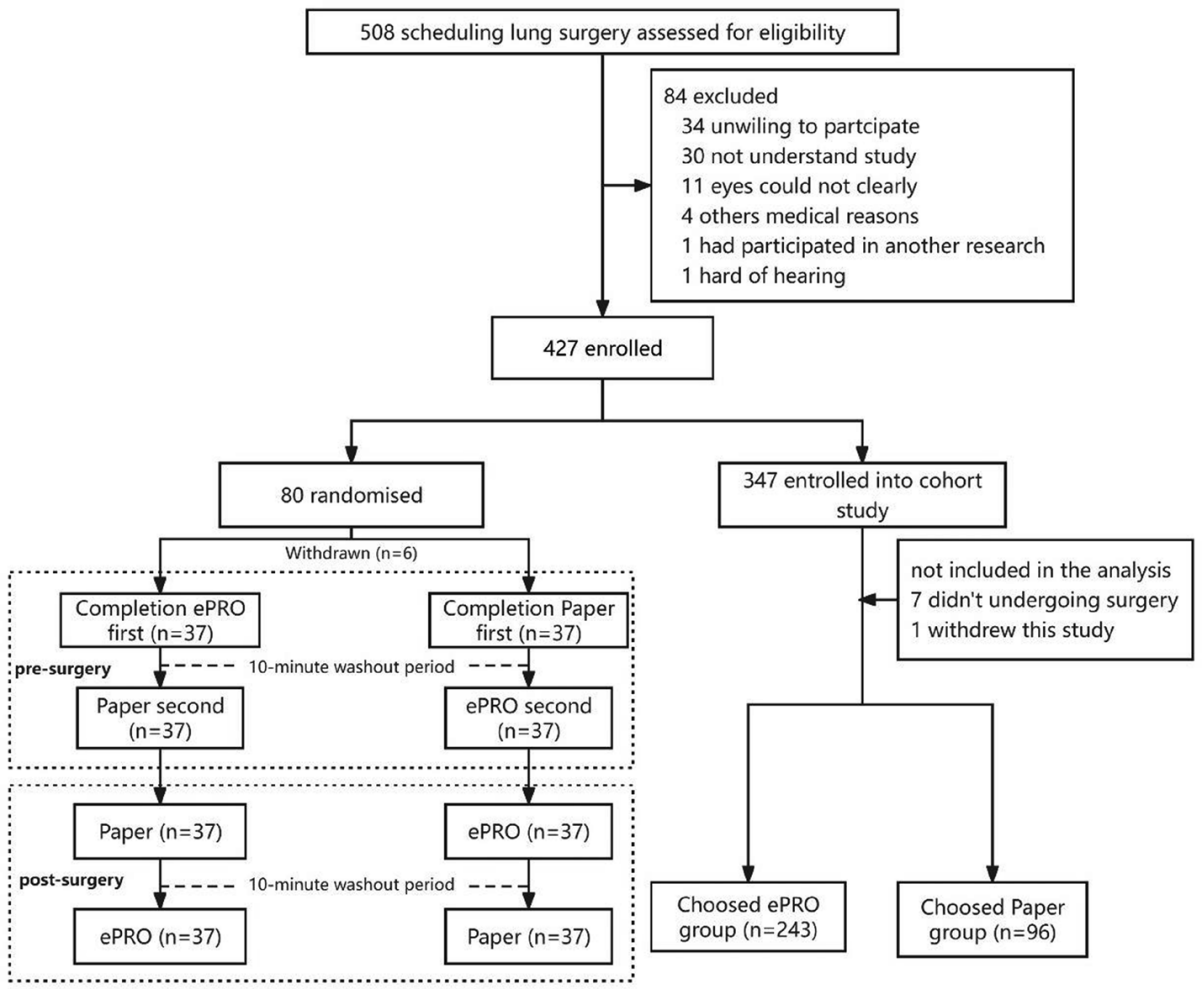
Pre-Treatment Readiness Tool for Sarcoma

**Fig. 1 (abstract 3033b):**
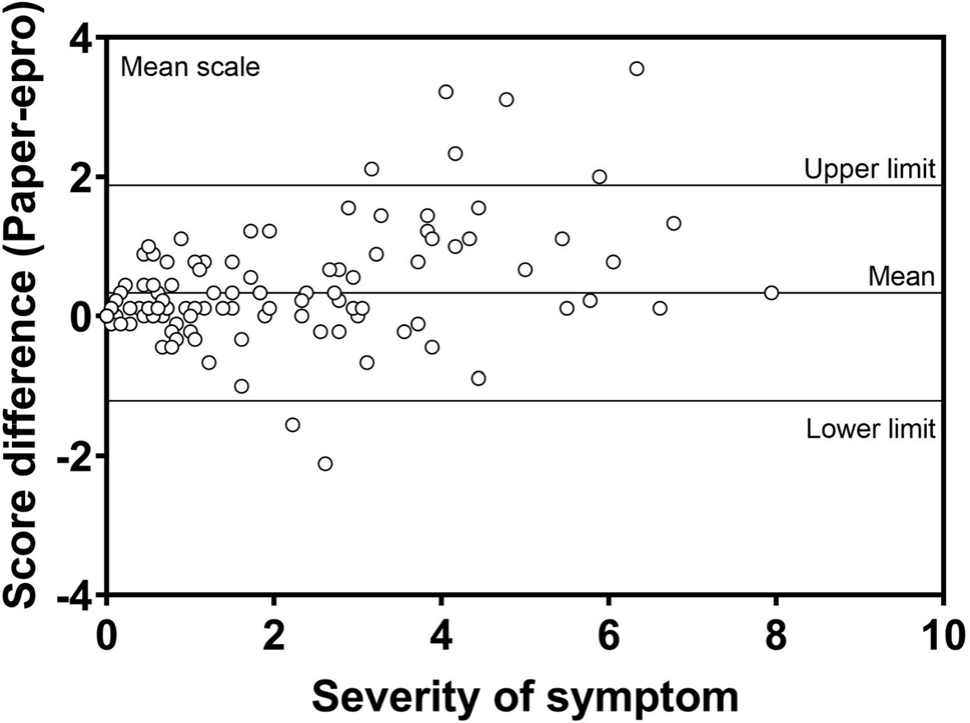
Pre-Treatment Readiness Tool for Sarcoma

**Table 1 (abstract 3033):**
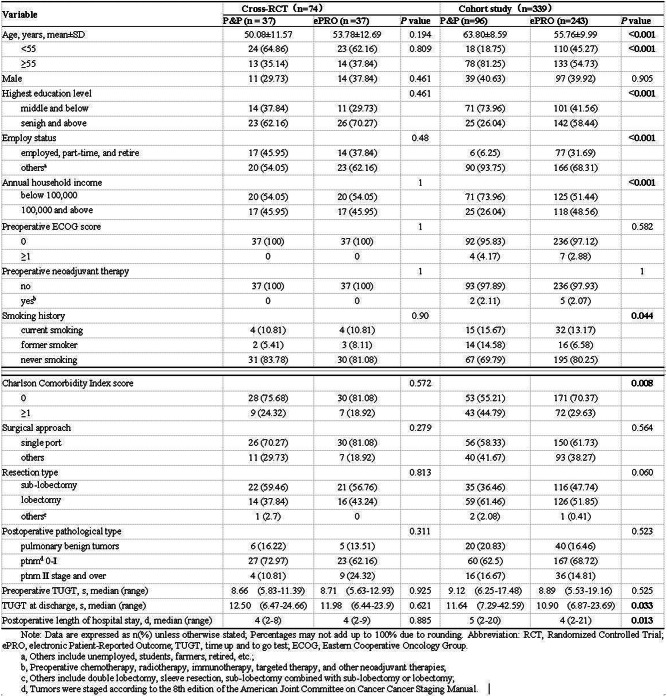
Demographic and Clinical Characteristics

## 3035 Validation and reliability of a Patient-Reported Outcome Tool for Assessing Anxiety in patients with HPV infection

Wenlin Wu^1^, Wei Xu^1^, Shi Wang^1^, Chenxi He^1^,Dengfeng Chen^1^, Li Fan^2^

^1^Chongqing Medical University, Chongqing, China, ^2^Chongqing General Hospital, Chongqing, China

*Journal of Patient-Reported Outcomes 2026*, **10(Suppl 1)**:3035

### Aims

A significant proportion of individuals infected with HPV experience varying degrees of anxiety, which can substantially affect their quality of life. In previous research, we developed a specific anxiety assessment scale for HPV. The primary objective of this study is to assess the anxiety levels of HPV-infected individuals using this self-developed scale, providing scientific evidence to support the prevention, early diagnosis, and psychological care of HPV-related diseases, while also validating the reliability of the scale.

### Methods

Demographic and disease-related data were collected from 660 HPV-infected individuals between July 1, 2024, and April 7, 2025, with 275 participants from Liuzhou City and 385 from Jincheng City. Their anxiety levels were assessed using the HPV anxiety scale. Item scores were analyzed using the mean ± standard deviation, and Cronbach’s alpha was calculated to assess the reliability of the scale. Item content validity indices (CVI), factor analysis results, and Pearson correlation analysis were used to evaluate the scale’s validity, while the differences in scores before and after treatment were used to assess responsiveness.

### Results

The anxiety levels of 660 HPV-infected individuals were assessed in this study, with 536 valid anxiety scales (94.4%) returned. The HPV anxiety scale demonstrated good reliability and validity, showing strong internal consistency (Cronbach’s α = 0.956). The total scale CVI was 0.83, and the item CVIs ranged from 0.75 to 0.85, indicating good content validity. Factor analysis revealed two components, with all items having eigenvalues greater than 0.5 in the main components, indicating strong structural validity. Correlation analysis with the patients’ overall anxiety scores (total score = 10) showed good criterion validity. Statistically significant differences in scores before and after treatment indicated the scale’s strong responsiveness. The survey results showed that respondents generally exhibited high levels of anxiety regarding their condition, particularly concerning the examination results and treatment outcomes.

### Conclusion

The developed HPV anxiety scale has demonstrated good reliability, validity, and responsiveness. HPV-infected individuals often exhibit elevated anxiety levels, which significantly impact various aspects of their lives, including disease examination and treatment, psychological burden, economic stress, and interpersonal relationships.

**Fig. 1 (abstract 3035):**
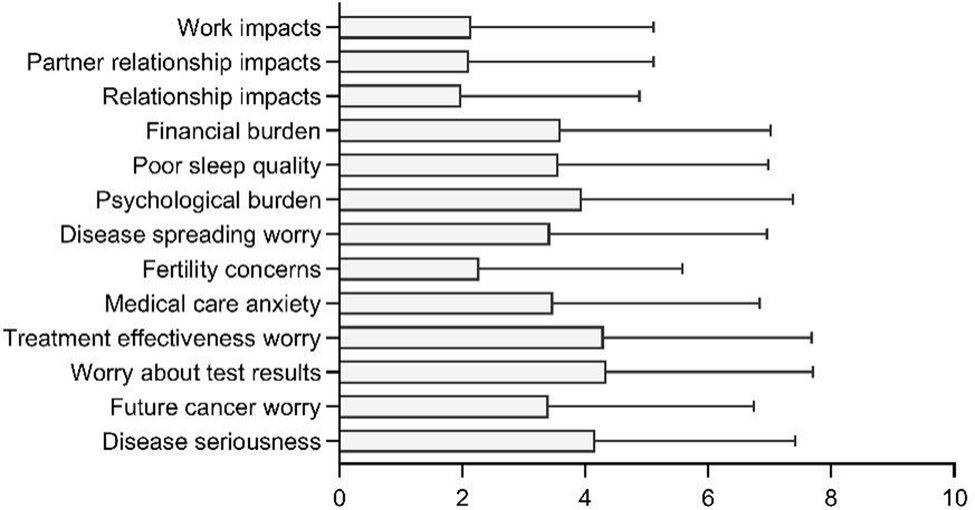
The score for each item of the HPV Anxiety Scale

**Table. 1 (abstract 3035):**
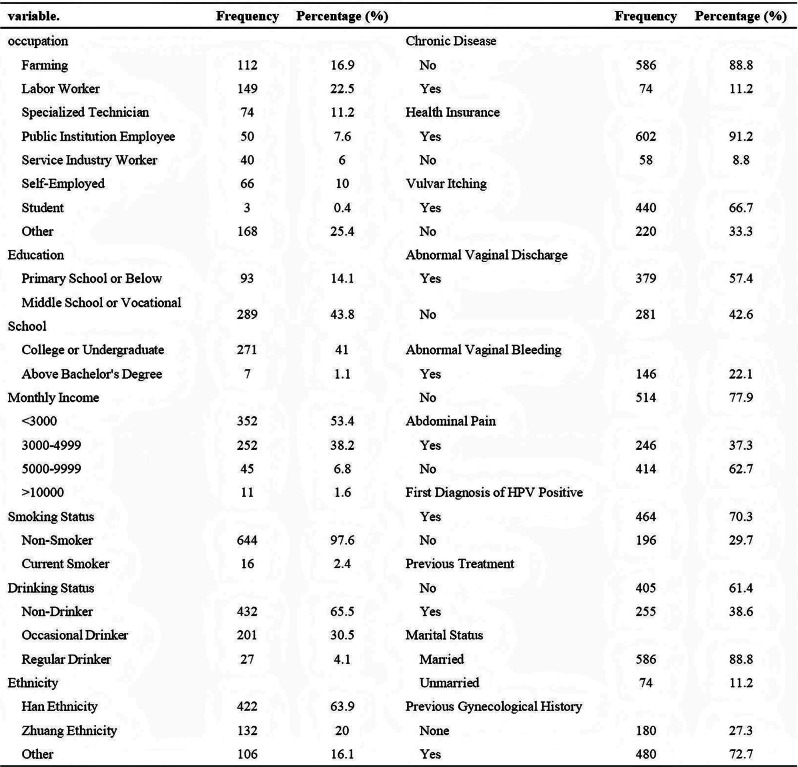
Basic Information of 660 HPV-Infected Individuals

**Table 2 (abstract 3035):**
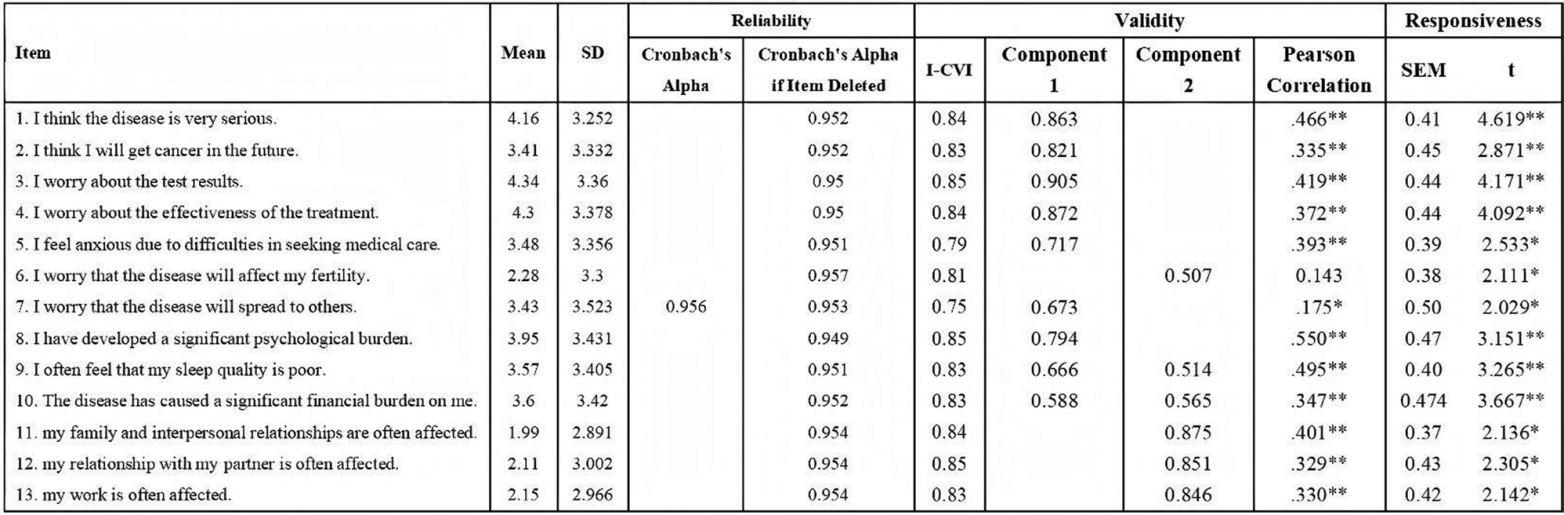
Results of Reliability and Validity Verification

## 3037 A Virtual Focus Group Model for Qualitative Cognitive Debriefing of Patient-Reported Outcome Measures

Sara Shaunfield^1^, Kimberly A Webster^1^, Maja Kuharic^1^, Chelsea R. Perschon^1^, Jissell Torres^1^, Jennifer Mammen^2^, Jamie Adams^3^, Melissa Kostrzebski^3^, Thimaikhue Nguyen^3^, Thao-Uyen Dang^3^, David Cella^1^, Sonya Eremenco^4^

^1^Northwestern University Feinberg School of Medicine, Chicago, Illinois, USA, ^2^University of Massachusetts Dartmouth, Dartmouth, Massachusetts, USA, ^3^University of Rochester, Rochester, USA, ^4^Critical Path Institute, Tucson, USA

*Journal of Patient-Reported Outcomes 2026*, **10(Suppl 1)**:3037

### Aims

While individual cognitive debriefing interviews are traditionally used for patient-reported outcome (PRO) measure evaluation and refinement, focus groups offer unique advantages for interactive discussion and collective feedback - particularly when evaluating multiple or lengthier measures. This study describes a novel panel-based virtual focus group methodology for cognitive debriefing, implemented to evaluate 12 draft PRO measures (51 items) for early Parkinson’s Disease (PD).

### Methods

We adopted a panel-based focus group design involving participants (N=11) from the Wearable Assessments in the Clinic and Home in Parkinson’s Disease (WATCH-PD) qualitative sub-study. Consented participants were divided into two independent groups, each meeting virtually on a weekly basis over four consecutive sessions (February–March 2025). Each session covered the same content, allowing participants to choose their preferred session. Sessions were conducted via Zoom, with a PowerPoint visual display of each measure, its respective items, and discussion topics. Using a structured approach that combined real-time digital polling (via Zoom reactions: yes/no/unsure) and open dialogue, participants evaluated item clarity, relevance to early PD (0–3 years post-diagnosis), response option appropriateness, and overall concept coverage for each draft PRO measure.

### Results

The panel-based approach demonstrated several methodological advantages. Session attendance remained consistently high across the four meetings (95% retention). The focus group format and multi-session design allowed for relationship-building within each group, resulting in progressively richer discussions and nuanced feedback. Digital polling provided immediate quantitative feedback on item clarity and relevance, highlighting problematic items for deeper exploration. This method effectively balanced structured assessment with open dialogue, generating both quantitative ratings and qualitative insights on item interpretation, missing concepts, and response option appropriateness.

### Conclusion

The panel-based virtual focus group model offers a practical and efficient methodology for PRO measure evaluation, with several advantages: 1) enabling the assessment of longer or multiple measures while maintaining participant engagement, 2) incorporating mixed-method data collection (digital polling plus in-depth discussion), and 3) fostering rapport across sessions, which enhanced feedback quality. These findings suggest that carefully structured, sequential focus group panels can effectively complement traditional one-on-one cognitive interviews for PRO measure development and refinement.

## 3039 Evaluating Construct Validity of the Patient-Reported Outcomes Measurement Information System 16-Item Profile in Total Laryngectomy Patients

Maria Edelen^1^, Chengbo Zeng^2^, Manraj Kaur^2^, Anne Klassen^3^, Michael Wu^4^

^1^RAND Corporation, Boston, Massachusetts, USA, ^2^Brigham and Women’s Hospital, Boston, Massachusetts, USA, ^3^McMaster University, Hamilton, Ontario, Canada, ^4^Washington University, St. Louis, Missouri, USA

*Journal of Patient-Reported Outcomes 2026*, **10(Suppl 1)**:3039

### Aims

The Patient-Reported Outcomes Measurement Information System^®^ (PROMIS^®^)-16 Profile assesses eight health-related quality of life domains using two items per domain. The PROMIS-16 was developed using data from a sample of 5775 general population respondents and demonstrated strong psychometric properties in a second cohort of general population respondents. In this study, we evaluated construct validity of the PROMIS-16 domain and summary scores among total laryngectomy patients.

### Methods

A total of 253 total laryngectomy patients were recruited from Massachusetts Eye and Ear Hospital, International Association of Laryngectomees, and two online laryngectomy support groups between July 2023 and March 2024. The PROMIS-16, EQ-5D-5L, and University of Washington Quality of Life Questionnaire (UW-QoL) were administered. We examined hypothesis-based construct validity of the PROMIS-16 domain and summary scores through their associations with UW-QoL scores and EQ-5D-5L index scores using Pearson product-moment correlations. We evaluated hypotheses, generated based on prior evidence, regarding the magnitude of 130 associations, using standard correlation thresholds: low (r<.30), moderate (0.30≤r<0.70), or high (r≥0.7) correlations.

### Results

Among the participants, 65% were male, and the median age was 69 years (IQR: 62 – 74). Most of the patients were White (93%), non-Hispanic (98%), and residing in the United States (88%). The domain scores ranged from 46.6 (SD=11.8) for cognitive function and 53.8 (SD=7.1) for social roles. The physical and mental health summary scores were 49.7 (SD=7.8) and 51.6 (SD=7.8), respectively. Eighty-seven percent of associations conformed with our hypotheses. As expected, except for cognitive function, all PROMIS-16 domain scores were significantly associated with those of UW-QoL, with absolute correlations ranging from 0.23 to 0.69, and with the EQ-5D-5L scores, with absolute correlations ranging from 0.29 to 0.74. The physical and mental health summary scores were significantly associated with all UW-QoL domain scores (range: 0.31 to 0.62), as well as the EQ-5D-5L index scores (range: 0.40 to 0.71).

### Conclusion

Construct validity of the PROMIS-16 domain and summary scores was demonstrated in patients who underwent total laryngectomy providing support for the usefulness of the PROMIS-16 in clinical samples.

**Table 1 (abstract 3039):**
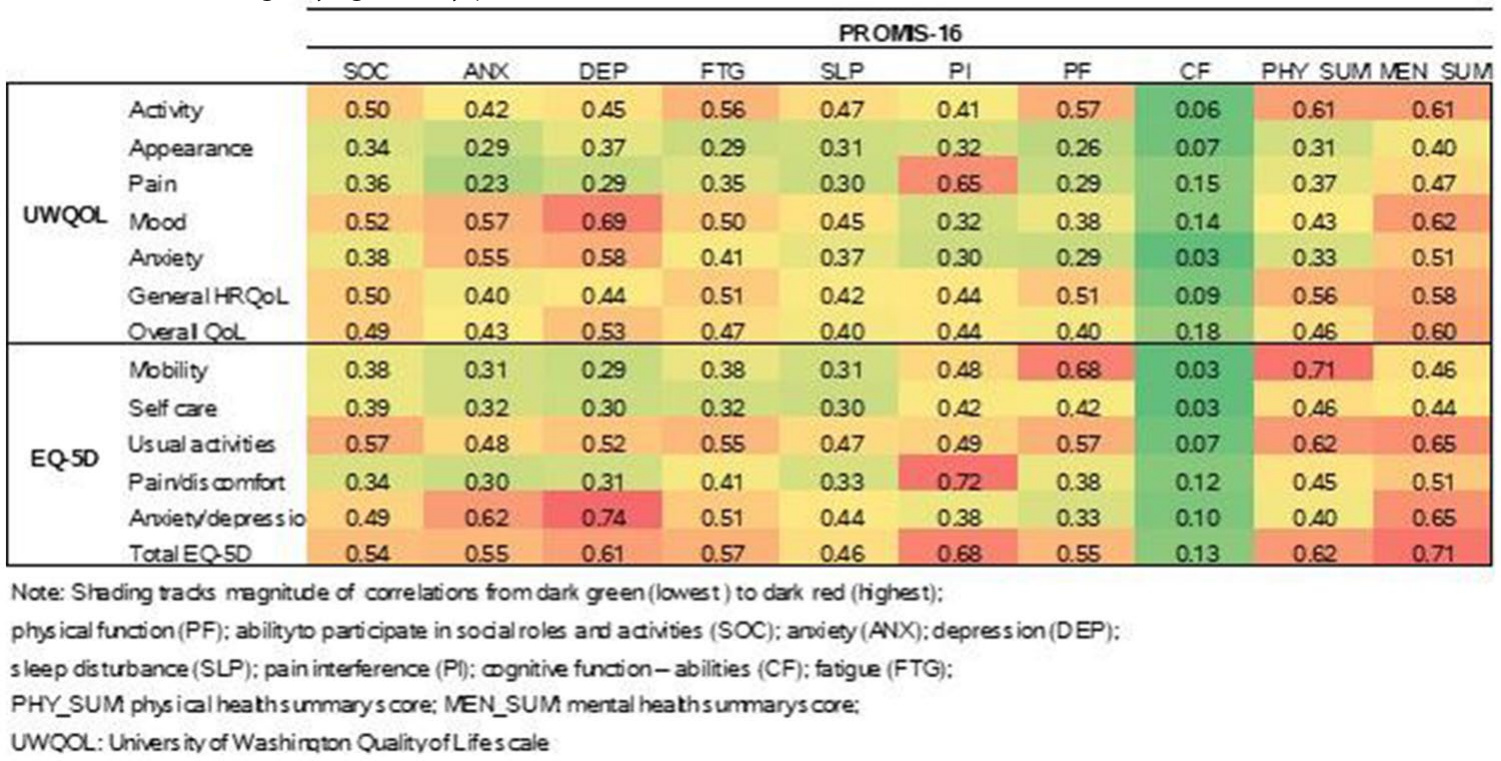
Absolute correlations of PROMS-16 domain and summary scores with UWQOL and EQ-5D scores among laryngectomy patients (n = 253)

## 3041 PRO-CTCAE Composite Average Validation in Prostate Cancer Phase 3 Trial

Minji Lee^1^, Blake Langlais^2^, Gina Mazza^2^, Amylou Dueck^3^, Ethan Basch^4^

^1^Mayo Clinic, Rochester, Minnesota, USA, ^2^Mayo Clinic, Scottsdale, Arizona, USA, ^3^UNC, Scottsdale, Arizona, USA, ^4^UNC, Chapel Hill, North Carolina, USA

*Journal of Patient-Reported Outcomes 2026*, **10(Suppl 1)**:3041

### Aims

The PRO-CTCAE composite average, calculated as the mean of PRO-CTCAE composite scores, summarizes overall symptom and side effect burden. This study investigates the internal structure, known-groups validity, test-retest reliability, and responsiveness to change.

### Methods

We analyzed PRO-CTCAE data from the COMET-2 trial (phase 3, 1:1 randomized) comparing cabozantinib (n=53) and mitoxantrone-prednisone (n=54) in 107 men with previously treated prostate cancer. Known-groups validity of the composite average score, summarizing 10 symptoms (diarrhea, constipation, nausea, vomiting, decreased appetite, shortness of breath, numbness and tingling, insomnia, fatigue, pain), was assessed by comparing symptom burden over time (area under the curve, AUC) between arms using a mixed effects model. Test-retest reliability was evaluated with the intraclass correlation coefficient (ICC) using week 3 and 6 data using the 0.7 as the acceptable threshold. Responsiveness was examined via paired t-tests and standardized mean difference (SMD) of composite averages at baseline and week 6. Confirmatory factor analysis (CFA) tested a one-factor model, randomly selecting one timepoint per patient.

### Results

PRO-CTCAE composite averages (range: 0.2–2.5) were collected at baseline and five follow-up timepoints (median: 4 surveys/patient). CFA supported a one-factor structure (CFI=0.97, TLI=0.96, RMSEA=0.067, 90% CI: 0.01–0.11). Known-groups validity showed higher symptom burden in the cabozantinib arm (AUC=10.2) versus mitoxantrone-prednisone (AUC=8.7; difference: 1.5, 95% CI: 0.2–2.8, p=0.024). A significant arm × timepoint interaction (p=0.016) indicated greater decreases in the mitoxantrone-prednisone arm at weeks 3 (-0.15) and 6 (-0.26) versus cabozantinib (-0.05 and -0.01). Test-retest reliability between weeks 3 and 6 yielded an ICC of 0.71 (95% CI: 0.58–0.80), meeting the 0.7 threshold. Responsiveness showed a decrease in the mitoxantrone-prednisone from baseline to week 6 (-0.26, p<0.001, SMD=-0.64, moderate effect), but not in cabozantinib (-0.01, p=0.86, SMD=-0.02, negligible effect), consistent with its higher symptom burden.

### Conclusion

Known-groups validity, adequate test-retest reliability, and responsiveness to change support PRO-CTCAE composite average for group-level comparisons. Higher symptom burden in cabozantinib, with its lack of responsiveness, suggests that the composite average effectively captures persistent toxicity while detecting improvements in the mitoxantrone-prednisone. These findings support using PRO-CTCAE composite average in phase 3 trials to monitor treatment-related symptom burden.

**Fig. (abstract 3041):**
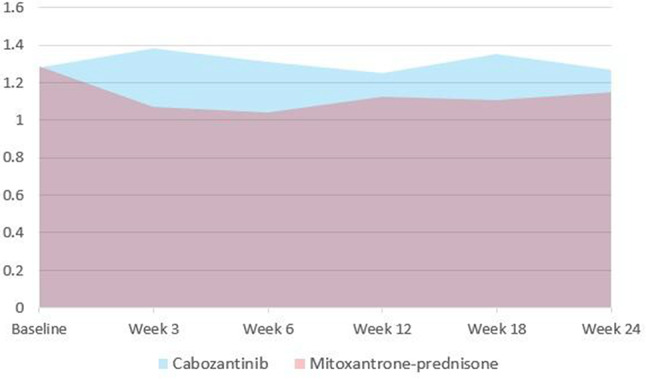
Longitudinal PRO-CTCAE composite average scores by treatment arm supporting AUC analysis

## 3045 Religeous beliefs and health outcomes: results from a population-based study in Japan

Yosuke Yamamoto^1^, Jun Miyashita^2^

^1^Department of Healthcare Epidemiology, Graduate School of Medicine, Kyoto University, Kyoto, Japan, ^2^Department of General Medicine, Shirakawa Satellite for Teaching and Research (STAR), Fukushima Medical University, Shirakawa, Japan

*Journal of Patient-Reported Outcomes 2026*, **10(Suppl 1)**:3045

### Aims

Although religious belief has been reportedly associated with various health conditions, the longitudinal relationship remains unknown. The purpose of this study was to examine the longitudinal association between religious belief and health outcomes, including health-related quality of life, in a general population in Japan.

### Methods

The cross-sectional and longitudinal studies were based on a web-based questionnaire survey (2022-2025), using data from Quality of Life in COVID-19 Era (QoLCoVE) study of adults from the general population in Japan. The relationship between this categorized degree of religious belief and health outcomes was examined using multivaiable regression models. Further, we also used the Scale of Motives for Faith developed by the authors, in order to investigate the detailed aspects of religeous beliefs and health outcomes.

### Results

Of the 3465 subjects who met the inclusion criteria, 4.2% (n=145) had deep religeous beliefs. Mental health is cross-sectionally associated with deep religeous beliefs, but a substantial relationship between the religeous beliefs and change in mental health was not observed in the longitudinal study. Other results will be presented on the day of the conference.

### Conclusion

A cross-sectional study suggested that there may be some association between degree of religious belief and mental health. In contrast, the results of the longitudinal study did not indicate that the degree of religious beliefs may contribute to future improvements in mental quality of life. Further studies based on longer follow-up periods are needed to clarify the relationship.

## 3047 Assessing comprehensibility of Dutch patient-reported outcome measures (PROMs) for people with low literacy skills

Lisa Stikvoort^1^, Ellen Elsman^2^, Olga Damman^3^, Caroline Terwee^2^, Lotte Haverman^4^

^1^Amsterdam UMC, Amsterdam, Netherlands, ^2^Amsterdam UMC, location University of Amsterdam, Epidemiology and Data Science, Amsterdam, The Netherlands, ^3^Amsterdam Public Health Research Institute, Amsterdam, Netherlands. Lotte Haverman^4^Amsterdam UMC, location University of Amsterdam, Emma Children’s Hospital, Child and Adolescent Psychiatry & Psychosocial Care, Amsterdam, The Netherlands

*Journal of Patient-Reported Outcomes 2026*, **10(Suppl 1)**:3047

### Aims

Approximately 2.5 million Dutch adults have low literacy, which may hinder their comprehension of medical information and communication of healthcare needs. Unsurprisingly, this population demonstrates low completion rates of patient-reported outcome measures (PROMs). As a result, they do not benefit from the clinical use of PROMs and are underrepresented in clinical trials and PROM research, further exacerbating health inequalities. While PROMIS items banks have been translated to Dutch and are applied in Dutch healthcare, it is unknown if they are suitable for those with lower literacy. This study therefore aims to assess the comprehensibility of selected PROMIS measures for Dutch individuals with low literacy.

### Methods

Eight PROMIS item banks from a standard PROM set recommended by the Dutch Ministry of Health were assessed: Ability to Participate, Anxiety, Cognitive Function, Depression, Fatigue, Pain Interference, Physical Function, and Sleep Disturbance, totaling 452 items. Item comprehensibility was assessed with the Dutch readability metric Klinkende Taal (resonant language) and by low literacy experts (n=25) in an online survey. Based on these assessments, the research team held a consensus meeting to select items for cognitive interviews with adults with low literacy (n≈40). Each item will be assessed at least four times, guiding further literacy-proof optimalization of the item banks.

### Results

In the consensus meeting (n=10), item inclusion and exclusion criteria were defined. Items were included for the interviews if ≥60% of experts (including Klinkende Taal) considered them comprehensible, and excluded if ≥80% considered them incomprehensible. Remaining items with mixed assessments were reviewed individually by the research team, resulting in 221 items selected for ongoing cognitive interviews (Figure). Final results will be presented at the ISOQOL conference.

### Conclusion

In this study, 221 items, identified as potentially comprehensible, were selected from an initial pool of 452 PROMIS items for evaluation by individuals with low literacy. Next, measurement properties will be evaluated in a large sample of people with low literacy (n=200) to ensure score comparability with existing PROMIS measures and other populations. The adapted measures will support the accurate completion of PROMs by Dutch individuals with low literacy, ultimately contributing to reduced health inequities.

**AQ64 Fig. 1 (abstract 3047):**
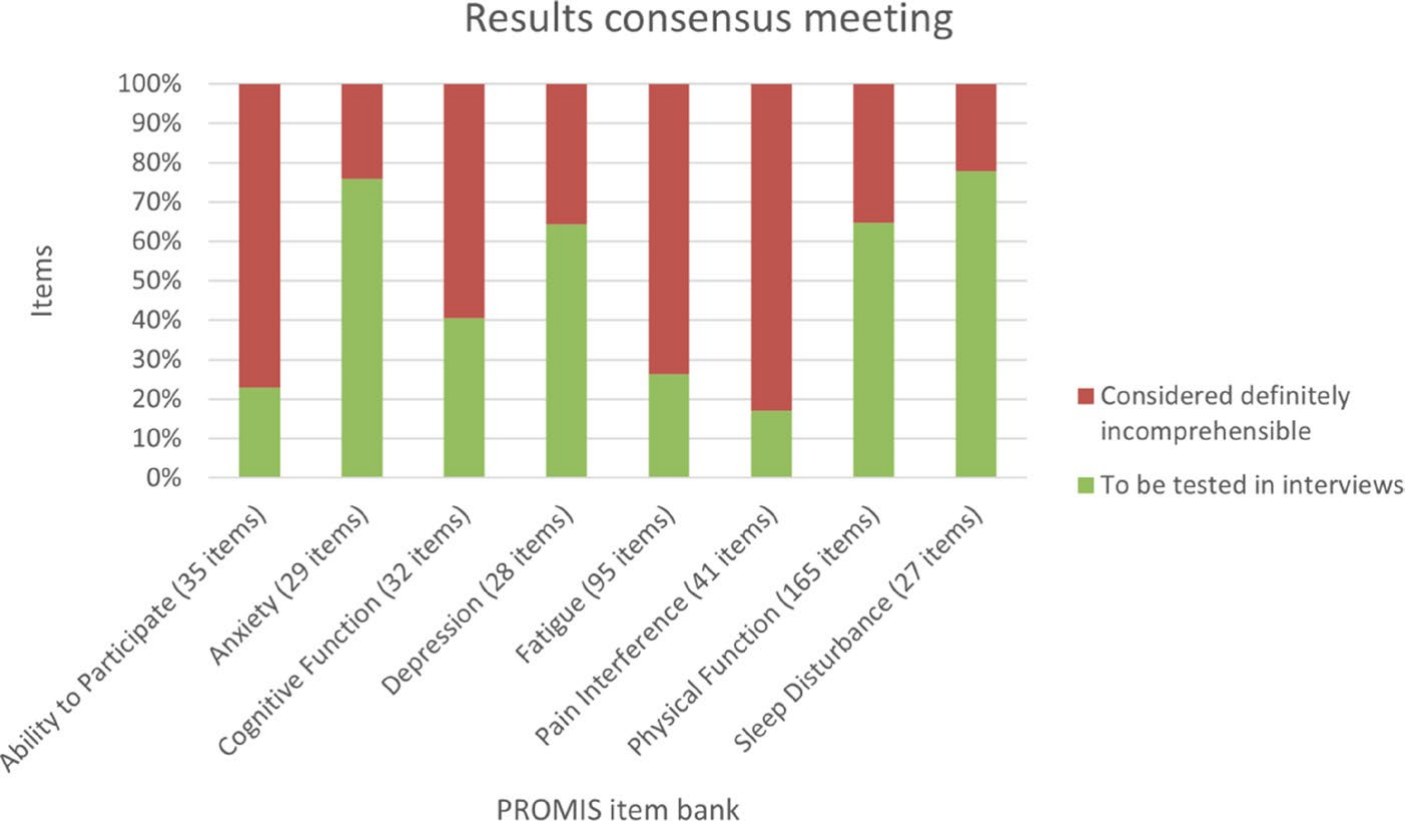
Assessing comprehensibility of Dutch patient-reported outcome measures (PROMs) for people with low literacy skills

## 3049 Development of a Modular Quality of Life Assessment Tool for Breast Cancer Patients Based on Patient-Reported Outcomes (PROs)

Wang Shi^1^, Shi Qiuling^1^, Xu Wei^1^, Wu Wenlin^1^, He Chenxi^1^

^1^Chongqing Medical University, Chongqing, China

*Journal of Patient-Reported Outcomes 2026*, **10(Suppl 1)**:3049

### Aims

Breast cancer is the second most common malignancy globally, and the multidimensional symptoms during treatment significantly affect patients’ physical and psychological well-being. Patient-reported outcomes (PROs) have been established as the most reliable source of subjective experience. However, existing PRO tools for breast cancer are limited by insufficient coverage and cultural differences. Therefore, this study aims to develop a modular PRO scale for breast cancer that is specifically tailored to the Chinese cultural context, ensuring a comprehensive and multidimensional assessment of patients’ quality of life, and better addressing their rehabilitation needs.

### Methods

Using purposive sampling, 38 breast cancer patients from a Grade-A tertiary hospital in Suining City were selected for semi-structured interviews between August and October 2024. Colaizzi’s seven-step analysis method was employed to refine the core dimensions of quality of life and create an item pool. The Delphi expert consultation method was then used to screen and revise items, leading to the development of the first version of the modular quality of life scale for breast cancer patients.

### Results

All 38 participants were female, with tumor stages ranging from I to IV. The interview data were analyzed and categorized into five themes: physical symptoms, psychological symptoms, daily life functions, family functions, and social functions. Physical symptoms were further categorized according to the treatment modality, including surgery, chemotherapy, and endocrine therapy. After two rounds of expert consultation, the initial version of the modular quality of life scale for breast cancer patients was finalized, which includes 27 physical symptoms, 7 psychological symptoms, 4 daily life functions, 2 family functions, 4 social functions, and 1 overall quality of life.

### Conclusion

This study developed a modular quality of life assessment tool for breast cancer patients based on PROs, which can be used to comprehensively evaluate patients’ quality of life throughout their treatment and rehabilitation process. Future steps will involve awareness surveys and reliability and validity testing, leading to the final version of the scale.

**Table 1 (abstract 3049a):**
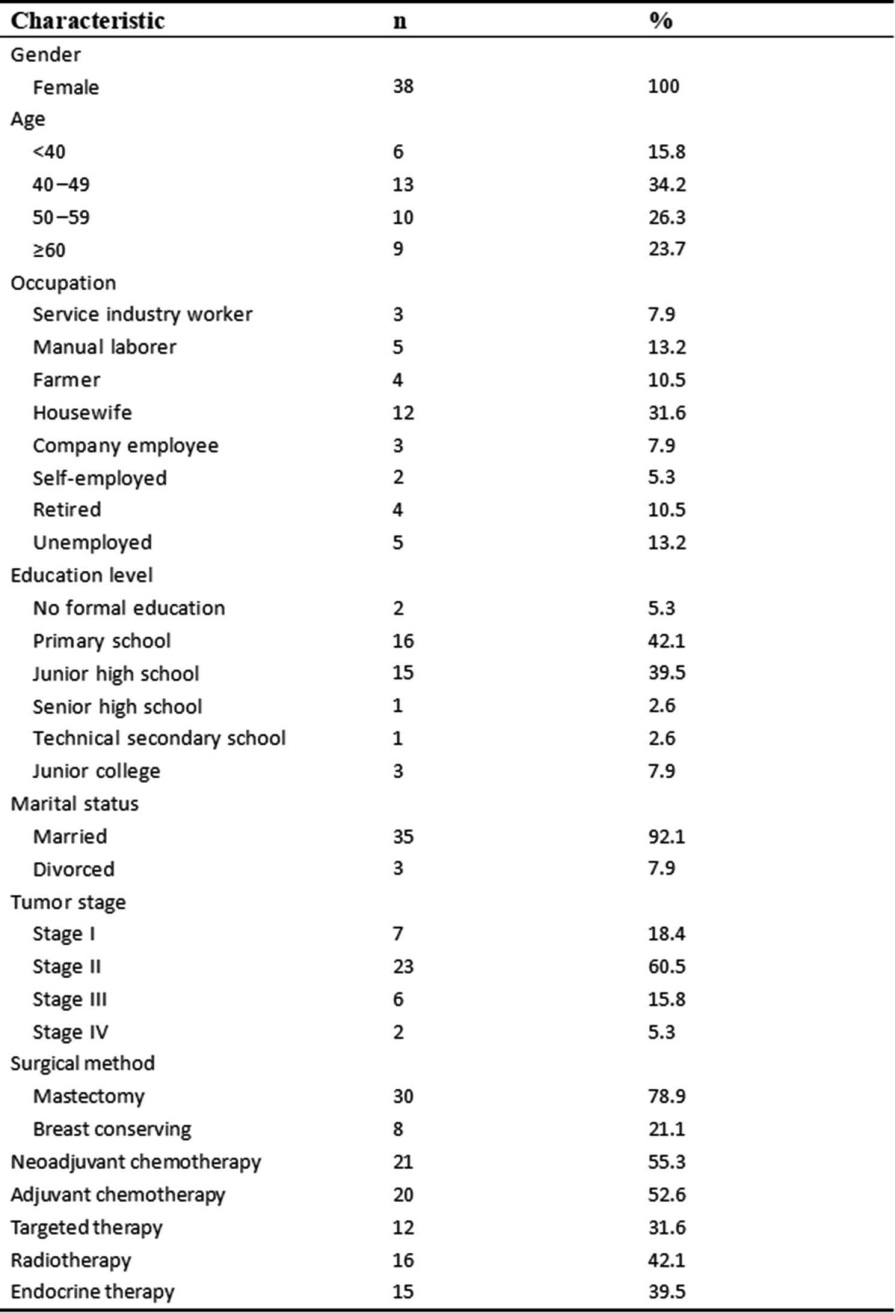
Characteristics of study participants (n = 38)

**Fig. 1 (abstract 3049b):**
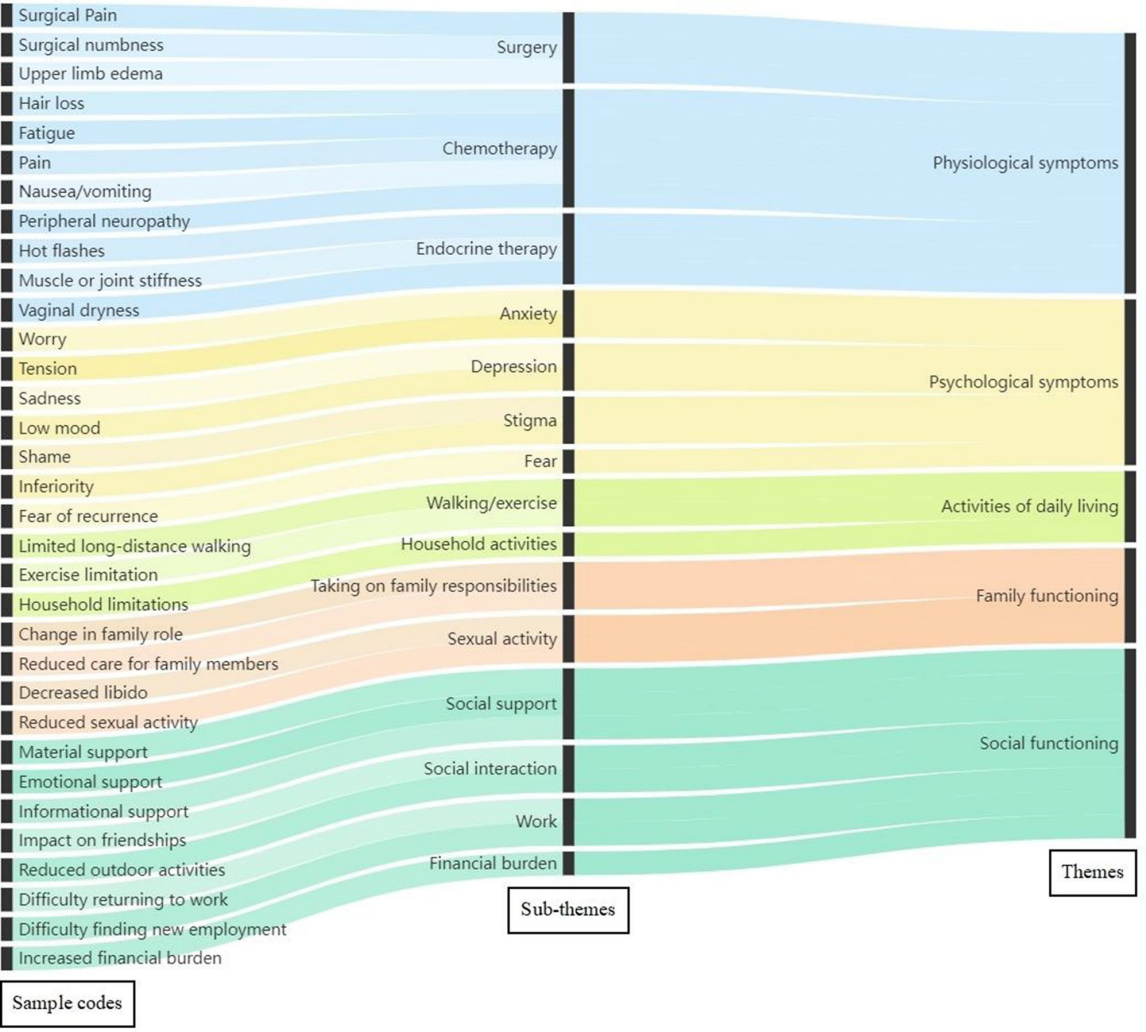
Development of a Modular Quality of Life Assessment Tool for Breast Cancer Patients Based on Patient-Reported Outcomes (PROs)

**Fig. 1 (abstract 3049c):**
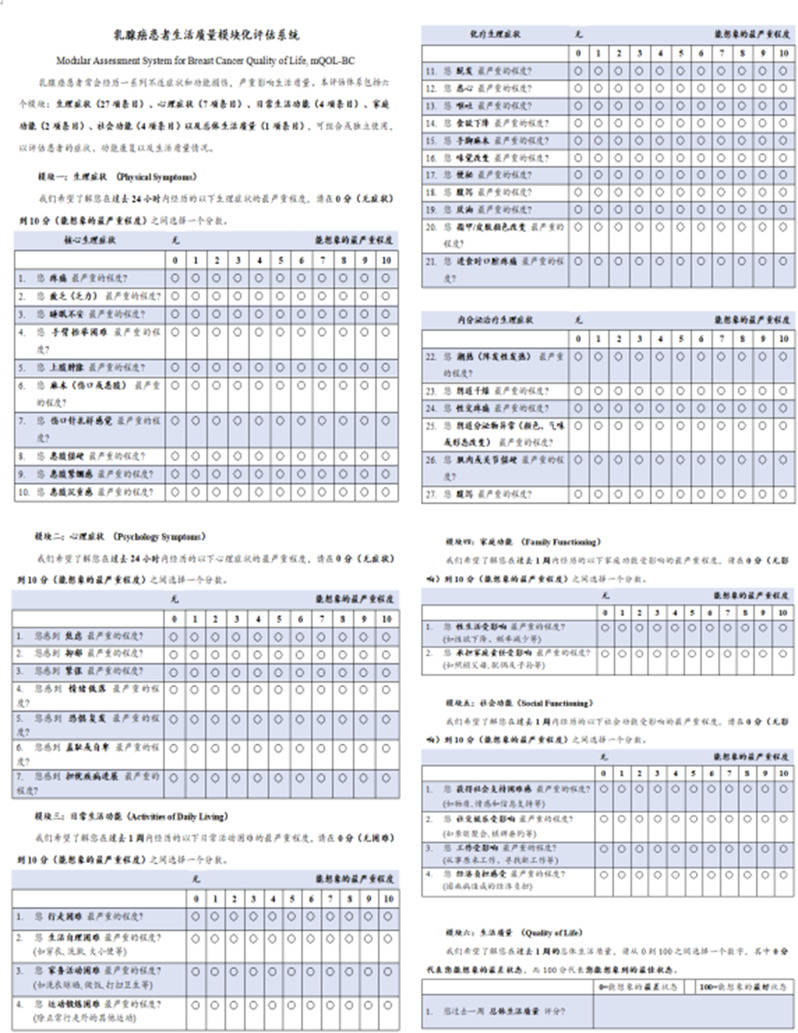
Development of a Modular Quality of Life Assessment Tool for Breast Cancer Patients Based on Patient-Reported Outcomes (PROs)

## 3051 Did The Kids Understand? Translating Key Concepts in Norwegian PROMIS Pediatric and Proxy Measures

Abigail Boucher^1^, Emily Parks-Vernizzi^1^, Paul Cella^1^, Benjamin Arnold^1^, Helena Correia^2^, Ida Sletten^3^, Andrew Garrett^4^, Kristine Risum^5^

^1^FACITtrans, Chicago, Illinois, USA, ^2^Lic, Northwestern University Feinberg School of Medicine, Department of Medical Social Science, Evanston, Illinois, USA, ^3^Oslo University Hospital, Division of Orthopaedic Surgery, Oslo, Norway, ^4^Norwegian Institute of Public Health, Oslo, Norway, ^5^Oslo University Hospital, Oslo, Norway

*Journal of Patient-Reported Outcomes 2026*, **10(Suppl 1)**:3051

### Aims

The purpose of this study was to translate and linguistically validate four PROMIS® Pediatric short forms (Fatigue 10a, Mobility 8a, Physical Activity 8a, Sleep Disturbance 8a) and Parent Proxy counterparts into Norwegian, highlighting linguistic issues encountered during the process.

### Methods

We translated 21 PROMIS Pediatric items and 21 Parent Proxy items using the FACIT translation methodology – a standardized iterative process of forward- and back-translation, expert review, harmonization, and cognitive interviewing. The translation team consisted of native Norwegian-speakers from Norway, and one native English speaking back-translator. During the cognitive interview phase, five Norwegian-speaking parent-child dyads from the general population assessed the relevance, understandability, and appropriateness of the translations. Qualitative analysis of cognitive interviews assessed the relevance and conceptual equivalence of the translations for each population.

### Results

The study sample consisted of 5 native Norwegian-speaking youths (3 female, 2 male) with a mean age of 13 (12-16) and 5 native Norwegian-speaking adults (2 female, 3 male) with a mean age of 37.5 (32-43) in Oslo, Tromsø, Harstad, Bergen and Holmestrand, Norway. Three changes were made to four Pediatric Fatigue 10a items (and four corresponding Parent Proxy items) following discussions with the Norway PROMIS National Center (PNC) and translations director at Northwestern University, namely: “follow” was added to the translation of “keep up”, “hurt” was added to the translation of “muscle burned,” and “trouble” was translated as “problem” (in two items). These revisions were made to facilitate pediatric understanding of the items, and to be consistent with previously validated translations.

### Conclusion

The Norwegian PROMIS Pediatric and Parent Proxy translated items are conceptually equivalent to the English source. Concurrent assessment of children’s and parents’ item interpretation confirmed consistent understanding between both populations. Inclusion of the Norway PROMIS National Center in the translation process was instrumental in fine tuning linguistic nuances and is recommended in future translation work. These Norwegian Pediatric and Parent Proxy items are acceptable for use in international research, clinical trials and clinical practice.

## 3053 Cross-cultural adaptation of the EQ-TIPS into Chichewa (Malawi): preliminary findings

Lucky Gift Ngwira^1^, Sebastian Mboma^1^, Upile Napola^1^, Jennifer Jelsma^2^, Janine Verstraete^2^, Des Scott^2^

^1^Health Economics and Policy Unit (HEPU), Kamuzu University of Health Sciences, Blantyre, Malawi, ^2^University of Cape Town, Cape Town, South Africa

*Journal of Patient-Reported Outcomes 2026*, **10(Suppl 1)**:3053

### Aims

The EuroQol Toddlers and Infant Populations (EQ-TIPS) is a health-related quality of life (HRQoL) instrument designed for children aged 0–3 years. While it has been validated in high-income settings, research in resource-limited contexts has been limited. This study aimed to culturally adapt and validate the EQ-TIPS into Chichewa for Malawi.

### Methods

The translation process involved forward and backward translations by two sets of translators, followed by review by the EuroQol committee to finalize a consensus version. The version was piloted through cognitive debriefing with five caregivers (two at home, three in hospital) to refine the translation. Changes were made accordingly based on their feedback to inform the next version.

### Results

The translation was relatively straightforward although two items: “managing emotions” and “interacting with others” were found to be difficult concepts to translate accurately. Further, pronouns (e.g., he/she) were added to response options for clarity. Cognitive debriefing revealed that “interacting with others” was best translated as a combination of kumasuka(freeness) and kulankhulana (speaking). For “sleep,” some respondents found the item difficult to rate as cited examples refer to different sleep phases. The term for “pain” (kuphwanya m’thupi) was debated, with suggestions to use kupweteka or kuwawa, perceived as more age-appropriate for infants. However, kuphwanya m’thupi was retained for consistency with the EQ-5D-Y, which assumes self-reporting. For “managing emotions” (dongosolo la malingaliro), caregivers recommended expanding examples (e.g., anger, sadness, happiness) to improve understanding.

### Conclusion

These are only preliminary results of the EQ-TIPS Chichewa version for Malawi. The next steps will be to refine the items and come up with a final approved version. The findings highlight that observable items (e.g., mobility) were easier to translate than emotional ones, possibly due to limited vocabulary in Chichewa—a challenge that may extend to other Bantu languages in sub-Saharan Africa.As the first effort to adapt EQ-TIPS into an African language outside South Africa, this work supports EuroQol’s strategic goals and provides insights for similar settings. Although these are only preliminary findings, the study underscores the need for contextual adaptations, such as additional examples for abstract concepts in young children.

## 3055 Bivariate latent change score modeling of pain and physical function in osteoarthritis

Aaron Kaat^1^, James Burns^1^, Martha Burla^1^, Wenjun Li^2^, Patricia Franklin^1^

^1^Northwestern University, Chicago, Illinois, USA, ^2^UMass-Lowell, Lowell, Massachusetts, USA

*Journal of Patient-Reported Outcomes 2026*, **10(Suppl 1)**:3055

### Aims

The A.S.K. (Assessing Shared Knowledge) study was designed to create a web-based tool to help inform osteoarthritis patients about when to have a joint replacement. When developing the shared decision-making tool, and as part of the A.S.K. study, multiple patient-reported outcome measures were collected at baseline, 6-, and 12-months, including the KOOS-12, which has domain scores for pain, function, and quality of life, and custom short forms from the Patient-Reported Outcomes Measurement Information System (PROMIS) Pain Interference and Physical Function item banks. This project is a secondary re-analysis of KOOS-12 and PROMIS data to evaluate the interrelationships among pain and function, including the impact of clinical and sociodemographic covariates.

### Methods

Two bivariate latent change score models—one for KOOS-12 and the other for PROMIS—in the A.S.K. data. External variables were added in blocks representing sociodemographic, emotional health, and physical health (including knee arthroplasty) covariates.

### Results

Most change occurred within 6 months after the index visit, with minimal additional change between 6- and 12-month follow-ups. At baseline, those who went on to have knee arthroplasty were generally in poorer health, with greater differences observed for PROMIS than for KOOS-12 (PROMIS |β| = .32 and.35, KOOS-12 |β| = .13 and .14 for pain and function, respectively). Change was significantly associated with treatment status. Cross-domain coupling was observed for both domains on the KOOS-12 except baseline function predicting 6-month pain, whereas only unidirectional coupling occurred on PROMIS (with Pain Interference associated with earlier Physical Function). The effect of covariates was broadly comparable across the models, with more noticeable effects related to surgical status, age, and baseline emotional health.

### Conclusion

The KOOS-12 and PROMIS measures are frequently used to assess orthopaedic patients considering knee arthroplasty. The A.S.K. study was designed to create a shared decision-making tool useful for supporting the use of patient reported outcomes at the individual level. This secondary analysis of A.S.K. data compared the longitudinal trajectory of pain and function at 6- and 12-months after the index visit. It elucidates the temporal relationship between changes in pain and function over time under both surgical and conservative treatment for osteoarthritis.

## 3057 Using a meaningful change item to support interpretations of meaningful score differences derived using traditional anchors

Alyssa Bamer^1^, Michael DeRosa^2^, Shruti Dave^1^, Helen Doll^4^

^1^Clinical Outcomes Solutions, Tucson, Arizona, USA, ^2^Clinical Outcomes Solutions, Chicago, Illinois, USA, ^3^Clinical Outcomes Solutions, Folkestone, UK

*Journal of Patient-Reported Outcomes 2026*, **10(Suppl 1)**:3057

### Aims

Current draft guidance from the Food and Drug Administration for estimating meaningful score differences (MSD) on a clinical outcome assessment (COA) suggests using anchors, such as Patient or Clinician Global Impact of Change or Severity (PGIC/S, CGIC/S), to identify estimates or ranges of changes in scores that are meaningful to patients. Use of these anchors requires some assumption or knowledge about what level of change on the anchor is considered meaningful to patients, which may not always be clear. One supportive approach to interpreting what level of change is meaningful is to ask patients directly if the change in symptoms experienced was “meaningful” (meaningful change item (MCI)). The use of this item, in conjunction with the anchor, can provide additional evidence in estimating MSD. In this study we aimed to examine how MSD estimates differed when using PGIS/C and CGIS/C versus patient-reported MCI in interpreting change on a clinician reported outcome (ClinRO). We also examined what level of change on the anchor was most likely to be associated with meaningful change on the MCI.

### Methods

A simulated dataset was created from an existing clinical trial dataset. The dataset included the following anchor items: PGIS/CGIS with 5-point Likert scale (No to Severe symptoms), PGIC/CGIC with 7-point Likert scale (Much better to Much worse), and MCI (Yes meaningful, Not meaningful, or No change). The multi-item ClinRO was scored on a 0–100 scale. Descriptive statistics examining COA scores (including percentage change) by anchor change groups were generated. Receiver operating curve (ROC) analyses were completed to identify best cut points between change groups on the anchors and the MCI.

### Results

The study is in progress. Initial findings suggest that the majority of patients reporting a 1-point change on the anchors report experiencing meaningful change, and ROC results from the MCI align with a MSD estimate associated with a 1-point anchor change.

### Conclusion

This study shows how including a MCI alongside a PGIS/CGIS or PGIC/CGIC can support the interpretation of MSD estimates based on traditional anchors. Changes of even 1-point may be meaningful to a majority of patients.

## 3059 Statistical vs. Clinical Significance: Shifting the Focus to Clinical Relevance in Patient - Reported Outcome Measures of Functional Dyspepsia

Shaofen Xu^1^, Zhengkun Hou^1^, Feng-bin Liu^1^

^1^Guangzhou University of Chinese Medicine, No. 12, Ji Chang Road, Baiyun District, Guangzhou, China

*Journal of Patient-Reported Outcomes 2026*, **10(Suppl 1)**:3059

### Aims

Properly defining and utilizing the minimal important change (MIC) and the minimal clinically important difference (MCID) is pivotal for discerning the clinical significance of study outcomes. The objective of this study is to systematically review randomized controlled trials (RCTs) focused on functional dyspepsia (FD) interventions, with the intent of evaluating the incorporation and interpretation of MIC/MCID values within these trials.

### Methods

We conducted a cross-sectional study to investigate the current status of RCTs of insomnia interventions to assess the inclusion and appropriate interpretation of MIC/MClD values. We included RCTs with no restrictions on interventions by searching PubMled, the Excerpta Medica Database (EMBASE), and the Cochrane CentraRegister of Controlled Trials (CENTRAL). The included studies also measured outcomes without restrictions.

### Results

The analysis included 13 studies (sample sizes: 29–224) assessing MIC/MCID across 9 countries and 4 intervention types (primarily pharmacotherapy). Terminology varied, with MIC (10 studies) more common than MCID (2 studies). Reported MIC/MCID values were PROM-specific: 4 (DSS), 0.5 (LPDS), ≥4 to ≤2 (GOS), 1.96 (GDSS/SDSS), and 2 (7-point Likert Scale). Half of studies (53.8%) cited external sources for thresholds, with 38.5% providing interval estimates (e.g., 0.4–0.6). Despite differing labels (e.g., “clinically meaningful difference,” “major improvement”), 11 studies were classified as MIC.

### Conclusion

In conclusion, this review of 13 studies reveals considerable heterogeneity in MIC/MCID terminology and values across gastrointestinal symptom research. While most studies employed MIC thresholds, the variability in definitions and measurement approaches highlights the need for standardized methodology in determining clinically meaningful changes. The findings underscore the importance of using validated, PROM-specific thresholds when interpreting treatment outcomes in both research and clinical practice. Future work should focus on establishing consensus-based standards to improve comparability across studies.

## 3061 The impact of psychological wellness and caregiver quality of life on patient quality of life after CAR T cell therapy

Emma DuMez^1^, Angela Steineck^2^ Amy Pan^1^ Liyun Zhang^1^

^1^Medical College of Wisconsin, Milwaukee, Wisconsin, USA

*Journal of Patient-Reported Outcomes 2026*, **10(Suppl 1)**:3061

### Aims

Investigate the relationship between patient psychological distress and health-related quality of life (HRQOL) among pediatric patients treated with chimeric antigen receptor (CAR) T cell therapy. Investigate the relationship between patient and caregiver HRQOL in this setting.

### Methods

English or Spanish-speaking patients (ages 8–25 years) undergoing CAR T-cell therapy and their primary caregiver were recruited from three large US pediatric cancer centers. Participants completed serial measures of HRQOL (patients; PedsQL, caregivers; CQOLC) and psychological distress (Kessler 6) for the first year following infusion. Summary statistics for each scale were calculated. Pearson correlations’ tests were used to examine each aim. Statistical significance was set at a two-sided p-value of <0.05.

### Results

N=88 families; 59 patients and 69 caregivers completed baseline assessments. Forty-six patients and 60 caregivers completed at least two assessments by three months post-infusion. Patient participants predominantly identified as white (77%) and 51% identified as female. The most prevalent diagnoses were ALL (44%), sarcoma (30%), and central nervous system tumor (14%). PedsQL scores were lowest at baseline (Generic Form [G]: mean: 64.67; standard deviation (SD): 17.43, Cancer module, [C]: mean: 68.27; SD:15.77) and greatest at 4-weeks post-infusion ([G] mean: 75.94; SD: 17.45, [C]: mean: 80.86; SD: 14.04). CQOLC scores were lowest at 3-months post-infusion (mean: 97.03; SD: 13.59) and greatest at baseline (mean: 103.19; SD: 17.38). Patient psychological distress scores were lowest at 9-months post-infusion (mean: 3.23; SD: 5.09) and greatest at baseline (mean: 6.42; SD: 5.78). Psychological distress was negatively correlated with PedsQL at 3-months post-infusion (r = -0.53, p = 0.0023), but the association was otherwise negligible at baseline (r = - 0.18, p = 0.12) and 4-weeks post-infusion (r = -0.22, p = 0.11). PedsQL and CQOLC were not correlated at baseline, 4-weeks, or 3-months (all p > 0.05).

### Conclusion

Although patient and caregiver HRQOL were not correlated at any timepoint, patient distress and PedsQL were significantly negatively correlated at 3-months post-infusion. This suggests a need for psychological support interventions serving patients around this point following therapy.

## 3063 Simulation of screen-to-CAT algorithm for PROMIS anxiety and depression in solid organ transplant recipients

Jad Fadlallah^1^, Wajiha Ghazi^1^, Kristen di Stefano^1^, Ward Hajjar^1^, Istvan Mucsi^1^

^1^University Health Network, Toronto, Ontario, Canada

*Journal of Patient-Reported Outcomes 2026*, **10(Suppl 1)**:3063

### Aims

Computer Adaptive Testing (CAT) offers precise assessment of patient-reported outcomes. The screen-to-CAT (stCAT) algorithm aims to reduce item burden. We evaluated stCAT through a simulation using existing PROMIS anxiety (PROMIS-A) and depression (PROMIS-D) CAT scores.

### Methods

Convenience sample of adult kidney, kidney-pancreas, and liver transplant recipients completing PROMIS-A and PROMIS-D CAT (higher T-score=worse symptoms) via electronic data capture. The CAT algorithm: minimum 4 items, continues until reliability≥0.90 or maximum of 12 items. For stCAT simulation, the first item of each domain serves as screener (“In the past 7 days … “I felt uneasy”[PROMIS-A]; “I felt depressed”[PROMIS-D]. If participants responded “Never,” T-score was estimated using this single item. For the whole sample we computed total number of items completed for CAT and the number of items if we had used stCAT. We assessed accuracy of stCAT compared to CAT for participants who would have completed stCAT using the mean absolute error(MAE), root mean square error(RMSE), agreement in symptom severity categories(<60:none/mild, ≥60:moderate/severe) and Bland–Altman plots. We evaluated group-level agreement with Cohen’s d(<0.2:negligible).

### Results

Of 535 participants, mean(SD) age was 53(14), 329(61%) were males and 322(70%) were white.stCAT scores were simulated for 76(16%) in PROMIS-A and 181(34%) in PROMIS-D.Mean[SD] PROMIS-A scores were similar for CAT and stCAT (42.1[5.6] vs. 41.6[0], Cohen’s d=-0.1[-0.3;0.1]), and for PROMIS-D (42.7[5.7] vs. 42.0[0], Cohen’s d=-0.1[-0.3;0.01]). However, MAE(95%CI) and RMSE(95%CI) were 4.6(4.0;5.3) and 5.6(4.9;6.3) for PROMIS-A, 4.8(4.3;5.2) and 5.8(5.1;6.4) for PROMIS-D, higher than previously reported minimal important differences. Bland-Altman plots showed that stCAT underestimated and overestimated scores for both domains, but only 1 PROMIS-A stCAT showed unacceptable inaccuracy(54.9→41.6) and 3 PROMIS-D stCAT showed unacceptable inaccuracy(78.6,59.6,58.3→42.0). Only 1 participant would have been mis-classified[CAT=78.6] to no depression[stCAT=42].Total item burden was reduced by 43% for PROMIS-D (2950 CAT vs. 1692 stCAT items) and by 21% for PROMIS-A (2558 vs. 2012). With stCAT, 93% and 90% of participants would have completed ≤4 items for PROMIS-D and PROMIS-A, respectively, compared to 69% and 79% with CAT.

### Conclusion

The stCAT algorithm reduces item burden, though some loss of accuracy is observed. This algorithm is very promising for distress screening given observed minimal misclassification.

## 3065 Social communication assessment priorities of adults with traumatic brain injury and speech-language pathologists

Lindsey Byom^1^, Helen Wrenn^2^

^1^Viterbo University, LaCrosse, Wisconsin, USA, ^2^Duke University Hospital, Durham, North Carolina, USA

*Journal of Patient-Reported Outcomes 2026*, **10(Suppl 1)**:3065

### Aims

Social communication encompasses the skills that individuals use to adapt their communication across contexts [1]. Social communication impairments are common after traumatic brain injury (TBI) and negatively affect quality of life [2]. Social communication, however, remains challenging to assess clinically [3]. This study aimed to identify social communication assessment priorities of adults with TBI and speech-language pathologists (SLPs).

### Methods

Adults with moderate-severe TBI [4] and licensed SLPs with adult TBI caseloads were recruited through purposeful sampling. Adults with TBI varied in age, injury cause, and time post-injury. SLPs varied in age, years of clinical experience, and practice setting. All participants completed semi-structured, video-recorded interviews about their social communication assessment goals. Interviews were transcribed verbatim and analyzed using inductive content analysis [5].

### Results

Fourteen adults with TBI (7 women; age range = 26–61 years, M = 42.86 years) and fifteen SLPs (13 women; age range = 26-67; M = 35.73 years) participated. SLPs had an average of 10 years of clinical experience (range = 2–30 years) and worked in diverse clinical settings. Adults with TBI expressed three primary social communication assessment goals: 1) improving communication skills needed to maintain valued social roles (e.g., health coach, parent); 2) identifying personalized supports (e.g., “I need to feel prepared for conversations,” “I always review my emails”; and 3) feeling heard and understood by the SLP. SLPs reported four main assessment goals: 1) inventorying expressive and receptive communication skills; 2) assessing cognitive contributions to communication challenges; 3) getting to know the patient (e.g., communication priorities, linguistic and cultural preferences, and daily communication demands); and 4) identifying contextual factors that could influence rehabilitation (e.g., fatigue, family support).

### Conclusion

Adults with TBI prioritized assessment goals that reflected their identities, roles, and lived experiences. SLPs aimed to align assessment with these individual goals while evaluating factors relevant to treatment planning. These findings support person-centered, goal-oriented assessment practices in social communication rehabilitation after TBI.

## 3067 Using multiple graphical approaches to support estimation of meaningful within patient change (MWPC) and meaningful score regions (MSRs)

Shruti Dave^1^, Helen Doll^2^, Michael DeRosa^3^, Hailin Yu^3^, Swetha Reddy^3^, Stacie Hudgens^4^

^1^Clinical Outcomes Solutions, Seattle, Washington, USA, ^2^Clinical Outcomes Solutions, Folkestone, UK, ^3^Clinical Outcomes Solutions, Chicago, Illinois, USA, ^4^Clinical Outcomes Solutions, Tucson, Arizona, USA

*Journal of Patient-Reported Outcomes 2026*, **10(Suppl 1)**:3067

### Aims

The standardized approach for anchor-based estimation of meaningful within patient change (MWPC) for a clinical outcome assessment (COA) includes tabular presentation of the distributions of COA change scores within anchor response categories. Meaningful score regions (MSR) are determined cross-sectionally and can be used to interpret derived MWPCs. The information presented in these tables, e.g., mean and median change scores and confidence intervals (CIs), can be challenging to apply directly to the derivation of MWPC and MSR, particularly when data are available from many anchors.

### Methods

In this study, multiple graphical methods were used to visualize COA change score distributions. In addition to empirical cumulative distribution function (eCDF) and probability density function (ePDF) curves classically used as supportive evidence when deriving meaningful change estimates, COA scores were also plotted for each anchor response category using box-and-whisker plots and error bars representing CIs and/or distribution percentiles. Formal forest plots were used to visualize group-based point estimates and measures of variability together with an overall weighted estimate (e.g., weighted by anchor correlation). Analyses were performed using a simulated dataset generated from an existing clinical trial dataset. The COA of interest was a patient-reported outcome (PRO) scored and transformed to a 0-to-100-point scale. The dataset also included the following anchor items: Patient Global Impression of Change (PGIC) with a 7-point Likert scale (Very much improved to Very much worse), item scores on an additional PRO measure with a 3-point Likert scale (Not at all, a little bit, and a lot), and EQ-5D-5L items with a 5-point Likert scale (No problems to Extreme problems). Descriptive statistics examining COA scores by anchor change groups were generated, alongside eCDF and ePDF curves, box-and-whisker plots, and forest plots.

### Results

The study findings suggest that graphical methods, in showing COA score variability across anchor categories, can help to derive MWPC and MSR by allowing for visualization and synthesis of data from multiple anchors. Furthermore, application of weighted estimates can allow for formal triangulation of meaningful change estimates.

### Conclusion

This study shows how use of graphical visualization methods can aid in applying anchor-based methods to MWPC and MSR estimation.

## 3069 Reliability and validity of the Colombian version of Kidscreen-10 in school children and adolescents

Martha Rodriguez^1^, Diana M. Camargo^2^, Luis C. Orozc^2^

^1^Universidad Santo Tomás, Bucaramanga, Colombia, ^2^Universidad Industrial de Santander, Bucaramanga, Colombia

*Journal of Patient-Reported Outcomes 2026*, **10(Suppl 1)**:3069

### Aims

To assess the reliability and validity of the Colombian version of the Kidscreen-10 in school children and adolescents aged 8 to 18 in the city of Bucaramanga, Colombia.

### Methods

A diagnostic technology evaluation was performed with 1334 children and adolescents who completed the Colombian version of the Kidscreen-10. Both institutions and children were randomly selected, first by cluster sampling in 30 public and private schools and second, by simple random sampling. The test-retest reliability was assessed in 121 randomly selected students, two separate times, two weeks apart. The application of the Kidscreen-10 was carried out during school hours in a quiet and comfortable place provided by the institutions. The analyses were conducted on the entire sample as well as for age groups (8-11 years and 12–18 years), sex, and socioeconomic conditions (low and high). The internal consistency of the item responses was estimated via Cronbach’s alpha coefficient as a measure of reliability of the Kidscreen scores; the test-retest reproducibility was evaluated calculating the intraclass correlation coefficient (ICC 2.1), values of 0.7 or higher were considered acceptable. The Rasch Rating Scale Model was used to establish the infit and outfit mean square, values between 0.6 and 1.4 indicated a good fit to the Rasch model. Differential Item Functioning (DIF) by age groups, sex and socioeconomic condition were identified. Stata version 18.0 and Winsteps software were used. Informed written consent was obtained from the parents or guardians, and the child/adolescent gave his or her assent to be included in the study.

### Results

The mean age was 12.3±2.7 years; 54.8% were female students, 85.8% lived in low socioeconomic conditions, 22.3% reported a functional limitation, and 88.5% attended public schools. Cronbach’s alpha coefficient was 0.82. Good reproducibility was found for the entire questionnaire (ICC 0.84 95% CI 0.75–0.90). Infit MNSQ values ranged between 0.67 and 1.37, and outfit MNSQ values ranged between 0.66 and 1.39. Uniform DIF was not observed. The variance explained by measures was 34.8%.

### Conclusion

The internal consistency and test–retest reproducibility of the Colombian version of the Kidscreen-10 were good. All items showed good fit to the Rasch model, and no DIF was found.

## 3071 Comprehensive Obesity-specific PRO Short-form Measuring Key Elements of Control and QOL Outcomes: Development and Empirical Evaluation of the Obesity Control Test

John Ware^1^, Sydney Cushing^2^

^1^UMass Medical School, Population and Qualitative Health Sciences, Massachusetts, USA, ^2^John Ware Research Group, Rhode Island, USA

*Journal of Patient-Reported Outcomes 2026*, **10(Suppl 1)**:3071

### Aims

Identifications of five elements common among disease-specific PROs enable construction of comprehensive short form summary measures. This paper documents the construction and psychometric evaluation of a new 5-item PRO survey of common elements specifically for use as a “barometer” for staging and monitoring obesity-specific control and QOL.

### Methods

Online NORC representative US population surveys in early 2025 identified 447 obese (overweight) adults (N=447, 27.4%). For obesity they completed five element measures: (1) Body mass index (BMI), and single-item PROs making obesity-specific attributions for: (2) Symptoms (legacy), (3) QOL Disease-specific Impact Scale (QDIS), (4) Severity and (5) Control. Test-retest reliability was estimated. Confirmatory factor analysis (CFA) tested the fit of a 2-factor higher-order oblique model and fixed factor loadings (see Figure).

### Results

Mean age was 47.8 years; the majority were female (63.3%). Race/ethnic characteristics were White (60%); Black (12.5%), Asian (20.6%), Hispanic (1.2%), mixed race (3.9%), and other (1.8%). Response times were below one minute for most respondents. Reliability was satisfactory. CFA results supporting two higher order factors (correlating r=0.31) explained 70% of the variance. On the basis of the pattern of primary and secondary loadings across the five element measures, factors were interpreted as obesity specific QOL (QDIS global, legacy symptom and severity ratings) and obesity control (BMI, severity). This model had satisfactory goodness of ﬁt (CFI = 0.992, RMSEA = 0.040). The generalizability of this 5-item, 5-element model, currently being evaluated, is supported by results from preliminary analyses across six chronic conditions (asthma, depression, diabetes, low back pain, obesity and osteoarthritis); results not reported.

### Conclusion

A very efficient (1-minute), comprehensive (5 key elements) and psychometrically-sound obesity-specific PRO “barometer” for staging QOL and weight control and for monitoring outcomes seems well within our reach. The underlying model and its construct validity are supported by current-study findings. This new test is worthy of further evaluations, which presently include responsiveness to weight gains and losses and validity in predicting generic QOL outcomes. The Obesity Control Test along with 2025 norm-based scoring instructions is being made available from the non-profit Mapi Research Trust at htps://eprovide.mapi-trust.org.

## VP1 Association of Particulate Matter 2.5 Exposure and Health-related Quality of Life among 38,724 Patients in Massachusetts, United States

Chengbo Zeng^1^, Tianyue Mi^2^, Manraj Kaur^1^, Jason Liu^1^, Mariem Ahmed^1^, Zhenlong Li^3^, Shan Qiao^4^, Xiaoming Li^4^, Andrea Pusic^1^, Maria Edelen^1^

^1^Brigham and Women’s Hospital, Boston, USA, ^2^University of South Carolina, Columbia, South Carolina, USA, ^3^Pennsylvania State University, Centre County, Pennsylvania, USA, ^4^University of South Carolina, Columbia, USA

*Journal of Patient-Reported Outcomes 2026*, **10(Suppl 1)**:VP1

### Aims

Concentration of particulate matter 2.5 (PM2.5) is a key indicator of air quality. Exposure to PM2.5 increases the risk of many health problems, including respiratory disease, heart disease, stroke, and lung cancer. However, limited real-world evidence exists on its impact on health-related quality of life (HRQoL). By linking individual patient data to county-level concentration of PM2.5, we examined the association between PM2.5 exposure and HRQoL among 38,724 patients in Massachusetts, United States.

### Methods

This study included 38,724 adult patients who underwent health-related social needs screening during their medical appointments within the Mass General Brigham healthcare system from March 2018 to January 2023. HRQoL was measured using the Patient-Reported Outcomes Measurement Information System Global-10 measure. We derived the global mental and physical health summary scores. Individual patient data was linked to monthly moving averages of county-level PM2.5 concentrations. We examined the associations of PM2.5 exposure and HRQoL using multilevel linear regression, adjusting for demographic and clinical characteristics and the random effect of county.

### Results

In the 38,724 patients, 61% were female, 34% were older adults, 76% were White, and 30% had comorbidities. In crude models, higher county-level PM2.5 concentrations were significantly associated with lower physical (β = -0.09, 95% CI: -0.15 to -0.02) and mental health scores (β = -0.18, 95% CI: -0.26 to -0.11). After adjustment, the association between PM2.5 and physical health was attenuated and not significant (β = -0.03, 95% CI: -0.10 to 0.03), while the negative association with mental health was significant (β = -0.12, 95% CI: -0.19 to -0.05).

### Conclusion

This study provides real-world evidence of the negative association of PM2.5 exposure and mental health. Future studies are encouraged to investigate the underlying biological and social mechanisms between them. Larger and longer-term studies are needed to further evaluate its impact on physical health.

**Table 1 (abstract VP1):**
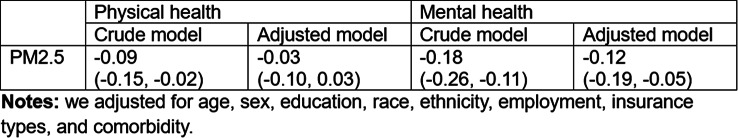
Association of PM2.5 and Health-related Quality of Life in 38,724 patients, 2018-2023

## VP2 Arabic Translation and Adaptation of the CVID_QoL Questionnaire to Measure Health-Related Quality of Life of Adults with Common Variable Immune Deficiency

Salim Zridi^1^, Laila Barakate^2^, Ahmed Aziz Bousfiha^1^

^1^Laboratory of Clinical Immunology, Inflammation and Allergy (LICIA), Faculty of Medicine and Pharmacy, Hassan II University, Casablanca, Benslimane, Morocco, ^2^Faculty of Medicine and Pharmacy, Hassan II University, Casablanca, Morocco

*Journal of Patient-Reported Outcomes 2026*, **10(Suppl 1)**:VP2

### Aims

This study aims to translate, culturally adapt, and validate the CVID_QoL questionnaire in Arabic for use with Arabic-speaking adults with CVID.

### Methods

The study followed a cross-cultural adaptation process, including forward and backward translation, expert review, and pilot testing with 20 Arabic-speaking CVID patients at the National Center for Primary Immunodeficiency, Abderrahim El Harouchi Hospital, Casablanca. The final version of the Arabic CVID_QoL questionnaire underwent psychometric evaluation for internal consistency, test-retest reliability, and construct validity.

### Results

The adapted Arabic version demonstrated good comprehensibility and cultural relevance. Internal consistency was high (Cronbach’s alpha = 0.91), and test-retest reliability was excellent (intraclass correlation coefficient = 0.88). Construct validity was supported by a strong correlation with the General Health 12 items survey (r = 0.67, p < 0.001).

### Conclusion

The Arabic CVID_QoL questionnaire is a reliable and valid instrument for measuring HRQoL in Arabic-speaking adults with CVID. Its availability will enhance patient care and research, offering a culturally appropriate tool for this population. Further studies with larger, multicenter samples are recommended to confirm these findings

## VP3 Physical, mental and social functioning of children in pediatric physical therapy: mental health matters

Dorinde L. Korteling^1^, Michiel A. J. Luijten^1^, Marjolijn Ketelaar^2,^^3^, Hedy A. van Oers^1^, Selina Limmen^1^, Lotte Haverman^1^, Eugene A. A. Rameckers^4,^^5,^^6^, Manon A. T. Bloemen^7^

^1^Amsterdam UMC location University of Amsterdam, Emma Children’s Hospital, Child and Adolescent Psychiatry & Psychosocial Care, Meibergdreef 9, Amsterdam, The Netherlands, ^2^UMC Utrecht Brain Center, University Medical Center Utrecht, The Netherlands, ^3^De Hoogstraat Rehabilitation, Utrecht, Center of Excellence for Rehabilitation Medicine Utrecht, The Netherlands, ^4^CAPHRI, Maastricht University, Maastricht, the Netherlands, ^5^Centre of Expertise, Adelante Rehabilitation centre, Valkenburg, the Netherlands, ^6^Rehabilitation Science and Physiotherapy, REVAL, Hasselt University, Belgium, Maastricht, Netherlands, ^7^Research Group Moving, Growing and Thriving Together, HU University of Applied Sciences, Utrecht, The Netherlands

*Journal of Patient-Reported Outcomes 2026*, **10(Suppl 1)**:VP3

### Aims

Pediatric physical therapy (PPT) focuses on maximizing children and adolescent’s potential for active participation. Patient-reported outcomes (PROs) provide valuable insight in healthcare, but their use in PPT remains limited. To explore the benefits of incorporating PROs into PPT, our goal is to offer an in-depth analysis of the physical, mental and social functioning of children and adolescents in PPT, using nine Patient-Reported Outcomes Measurement Information System (PROMIS®) instruments.

### Methods

Children and adolescents (8-17 years) in PPT completed nine PROMIS® v2.0 instruments in computerized adaptive testing (Anxiety, Depressive Symptoms, Fatigue, Mobility, Pain Interference, Peer Relationships, Sleep Disturbance, Upper Extremity) or shortform format (Anger). The mean T-scores of the PROMIS instruments were compared to reference data of the Dutch general population with an independent t-test. Correlations (Pearson’s r) between T-scores of the PROMIS instruments were explored. Associated variables with PROMIS T-scores have been explored with an ANCOVA analysis.

### Results

The 150 participating children scored worse compared to the general population on all PROMIS® instruments (p<0.005; Cohen’s D’s: 0.24-1.13; mean difference=2.99-10.12), except Peer Relationships (p=0.26; D=0.10; mean difference=0.88), and Anger (p=0.35; D=0.10; mean difference=1.21). Nearly three-quarter (72.0%) of children treated by physical therapists scored above the cut-off for moderate clinical concern (T-score ≤55.9) on the Mobility item bank, and over half (58.3%) of the children did so for the Upper Extremity item bank (T-score ≤52.8). For all other PROMIS instruments, 30-40% of children scored above the cut-off for moderate clinical concern, except for Peer Relations (19.3% above cut-off). No Dutch clinical cut-off scores are currently available for the Sleep Disturbance item bank. Strong positive correlations (r>0.7) were found between the Depression item bank and the Anxiety and Anger item banks. Associations with covariates will be presented at the conference.

### Conclusion

This study provides an overview of physical, mental and social functioning of children and adolescents in treatment with a physical therapists, showing that children in PPT experience reduced functioning in both physical and mental health compared to the general population. This highlights the broader impact of their condition beyond the primary reason for therapy, and emphasizes the importance of incorporating PROs in PPT.

## VP4 Child-caregiver agreement and test-retest reliability of nine PROMIS item banks in pediatric physical therapy

Dorinde L. Korteling^1^, Selina Limmen^1^, Marjolijn Ketelaar^2,^^3^, Eugene A. A. Rameckers^4,^^5,^^6^, Hedy A. van Oers^1^, Manon A. T. Bloemen^7^, Michiel A. J. Luijten^1^, Lotte Haverman^1^

^1^Amsterdam UMC location University of Amsterdam, Emma Children’s Hospital, Child and Adolescent Psychiatry & Psychosocial Care, Meibergdreef 9, Amsterdam, The Netherlands, ^2^UMC Utrecht Brain Center, University Medical Center Utrecht, The Netherlands, ^3^De Hoogstraat Rehabilitation, Utrecht, Center of Excellence for Rehabilitation Medicine Utrecht, The Netherlands, ^4^CAPHRI, Maastricht University, Maastricht, the Netherlands, ^5^Centre of Expertise, Adelante Rehabilitation centre, Valkenburg, the Netherlands, ^6^Rehabilitation Science and Physiotherapy, REVAL, Hasselt University, Belgium, Maastricht, Netherlands, ^7^Research Group Moving, Growing and Thriving Together, HU University of Applied Sciences, Utrecht, The Netherlands

*Journal of Patient-Reported Outcomes 2026*, **10(Suppl 1)**:VP4

### Aims

While self-reported patient-reported outcome measures (PROMs) are preferred, proxies are sometimes necessary. To interpret proxy PROMs scores responsibly and correctly, it is important to know the level of child-caregiver agreement on PROMs. Furthermore, assessing stability of PROMs over time is crucial for ensuring test-retest reliability. Thus, our aim is twofold: (1) examine child-caregiver agreement across nine PROMIS® v2.0 instruments, and (2) to assess the test-retest reliability of three paediatric self and proxy PROMIS® v2.0 instruments in a pediatric physical therapy (PPT) setting.

### Methods

Children (8-17 years) in PPT, and their caregivers (of children aged 5–17 years), completed three pediatric and proxy PROMIS® item banks v2.0 (Pain Interference, Mobility, Upper Extremity) and two legacy instruments (Pediatric Quality of Life Inventory 4.0, NRS Pain Intensity). PROMIS instruments were completed again within three weeks. At this measurement occasion, participants also completed six PROMIS® instruments in computerized adaptive testing format (Anxiety, Depressive Symptoms, Fatigue, Peer Relationships, Sleep Disturbance) or as short-form (Anger). Agreement within child-caregiver dyads for all PROMIS measures and legacy instruments was explored with intraclass correlation coefficients (ICC). Test-retest reliability was assessed for PROMIS Pain Interference, Mobility and Upper Extremity using ICCs. ICC estimates were deemed moderate if ≥0.50 and strong if ≥0.75.

### Results

For child-caregiver agreement (aim 1), 227 child-caregiver dyads completed the PROMIS Pain Interference, Mobility, Upper Extremity item banks and legacy instruments. 146 child-caregiver dyads completed the six additional PROMIS instruments. PROMIS and legacy instruments showed moderate (Anxiety and Fatigue; ICCs=0.73) to strong (all other instruments: 0.75-0.84) ICC estimates.For test-retest reliability (aim 2), 85 children and 127 caregivers participated at first and second administration. Moderate ICC estimates were found for the pediatric and proxy PROMIS Pain Interference (0.71; 0.72), Mobility (0.69; 0.67), Upper Extremity (0.63; 0.66) item banks.

### Conclusion

Child-caregiver agreement on the nine pediatric PROMIS Item Banks v2.0 was generally strong, similar to legacy instruments, suggesting that parent-proxies can be reliably used when a child is unable to self-report. Test-retest reliability of pediatric and proxy PROMIS Pain Interference, Mobility, Upper Extremity item banks v2.0 in PPT showed to be sufficient for use in clinical practice.

## VP5 Responsiveness of pediatric v2.0 and v3.0 PROMIS Pain Interference, Upper Extremity and Mobility

Dorinde L. Korteling^1^, Michiel A. J. Luijten^1^, Hedy A. van Oers^1^, Manon A. T. Bloemen^2^, Lotte Haverman^1^, Selina Limmen^1^, Eugene A. A. Rameckers^3,^^4,^^5^, Marjolijn Ketelaar^6,^^7^

^1^Amsterdam UMC location University of Amsterdam, Emma Children’s Hospital, Child and Adolescent Psychiatry & Psychosocial Care, Meibergdreef 9, Amsterdam, The Netherlands, ^2^Research Group Moving, Growing and Thriving Together, HU University of Applied Sciences, Utrecht, The Netherlands.^3^CAPHRI, Maastricht University, Maastricht, the Netherlands, ^4^Centre of Expertise, Adelante Rehabilitation centre, Valkenburg, the Netherlands, ^5^Rehabilitation Science and Physiotherapy, REVAL, Hasselt University, Belgium, Maastricht, Netherlands, ^6^UMC Utrecht Brain Center, University Medical Center Utrecht, The Netherlands, ^7^De Hoogstraat Rehabilitation, Utrecht, Center of Excellence for Rehabilitation Medicine Utrecht, The Netherlands

*Journal of Patient-Reported Outcomes 2026*, **10(Suppl 1)**:VP5

### Aims

To support clinical decision-making and treatment evaluation, outcome measures need to accurately detect changes in patient health. As such, we aim to assess the responsiveness of the self and proxy PROMIS® Item Banks v2.0 and v3.0 for Pain Interference, Mobility, and Upper Extremity in the context of pediatric physical therapy (PPT).

### Methods

Children (8-17 years) in PPT, and their caregivers (of children 5–17 years), completed three pediatric PROMIS® item banks v2.0 (Upper Extremity, Mobility, Pain Interference). Caregivers indicated the domain (upper extremity, mobility or pain) on which PPT focused the following six months (intervention domain). At follow-up, a self-assessment of change question (SCQ) was administered per domain.Responsiveness was assessed using two construct-based approaches (efficacy-approach and known-groups-approach; based on the intervention domain) and one anchor-based approach (based on SCQ). For construct-based approaches: 1. Efficacy-approach: T-scores on baseline and follow-up were compared using a paired samples t-test to assess responsiveness of PROMIS given the PPT intervention domain. 2. Known-groups-approach: Change in T-scores between baseline and follow-up was compared between participants in different intervention domains, using an independent t-test. For anchor-based approach: 3. Change in T-scores was compared between participants reporting perceived change (SCQ) and those who did not. To compare responsiveness of v2.0 with v3.0, responses were recoded/collapsed and T-scores were recalculated. Subsequently, all above analyses were performed on v3.0 T-scores as well.

### Results

Currently, 76 children and 100 caregivers participated (data collection ends May 2025). For construct-based approaches: 1. Efficacy-approach: Preliminary results show participants improving for all PROMIS v2.0 instruments given PPT intervention domain (p-values<0.05; Cohen’s D’s=0.6-4.4). 2. Known-groups-approach: Participants focussing on upper extremity as intervention domain showed a larger improvement on PROMIS v2.0 Upper Extremity T-scores compared to people not focussing on upper extremity as intervention domain (p=0.006; D=1.4). For mobility (p=0.2; D=0.5) and pain interference (p=0.5; D=0.3), this statistically significant difference was not found. For anchor-based approach: 3. Results will be presented at the conference. Final results, including a comparison between v2.0 and v3.0, will be presented.

### Conclusion

Preliminary results show indications of sufficient responsiveness of pediatric PROMIS item banks for PPT.

## VP6 Bridging the gap: Understanding child and caregiver discrepancies in patient-reported outcome measures

Erin McCabe^1^, Aline Thorkelsson^2^, Whitney Hindmarch^3^

^1^Department of Physical Therapy, University of Alberta, Edmonton, Alberta, Canada, ^2^Department of Psychology, University of Alberta, Edmonton, Alberta, Canada, ^3^Department of Radiology, University of Calgary, Calgary, Alberta, Canada

*Journal of Patient-Reported Outcomes 2026*, **10(Suppl 1)**:VP6

### Aims

Patient-reported outcome measures (PROMs) are often used in mental health care for individual treatment planning and organizational decision-making. In child and adolescent mental health, multiple informants (i.e., child, caregiver, teacher) often complete parallel forms of a PROM. However, research consistently demonstrates low to moderate agreement between their scores, known as “informant discrepancies.” These discrepancies are often attributed to measurement error, but alternative explanations exist. Understanding the source of discrepancies is important to improve the validity of the interpretations made from PROM scores. This study begins to address this challenge by exploring factors that influence child and caregiver discrepancies.

### Methods

This exploratory study uses secondary analysis of data from the Pediatric Quality of Life Inventory (PedsQL) and the Revised Children’s Anxiety and Depression Scale – 25, which were administered during routine clinical care (intake and discharge) to youth (aged 8-18) and their caregivers at a child and adolescent mental health centre. The agreement between caregiver and child self-report was examined using an agreement-type Intraclass Correlation Co-efficient, across the four domains of PedQL and 2 domains of RCADS-25, across child age (8-14 years vs 15–18 years old), and at intake versus discharge from a child mental health service. Based on existing knowledge, we hypothesize that ICCs will be lower for domains focused on internal experiences (e.g., anxiety symptoms) compared with external behaviours (e.g., school functioning), that ICCs will be higher for older children, as caregivers may be less involved in their daily lives and less accurate in reporting symptoms, and ICCs will be higher at discharge compared to intake, as the mental health program from which this data was collected focuses on improving family communication, which may lead to greater alignment of child and caregiver’s perceptions of the child’s mental health experiences.

### Results

Participants include 264 client and caregiver dyads. Mean child age was 14.8 years (SD=1.7), 33.8% were young men, 61.2% young women, 4.6% were trans- or non-binary. This study is in progress.

### Conclusion

This study will contribute to the understanding of the factors influencing PROM score discrepancies, providing clinicians and researchers with information about interpreting PROM score discrepancies in child and adolescent mental health.

## VP7 Using an argument-based approach to validation to evaluate three PROMs for intensive outpatient child and adolescent mental health services

Erin McCabe^1^, Whitney Hindmarch^2^, Bishnu Bajgain^3^, Jennifer Zwicker^4^, Maria Santana^5^

^1^Department of Physical Therapy, University of Alberta, Edmonton, Alberta, Canada, ^2^Department of Radiology, University of Calgary, Calgary, Alberta, Canada, ^3^Community Health Sciences, University of Calgary, Calgary, Alberta, Canada, ^4^School of Public Policy, University of Calgary, Calgary, Alberta, Canada, ^5^Community Health Sciences, University of Calgary, Calgary, Alberta, Canada

*Journal of Patient-Reported Outcomes 2026*, **10(Suppl 1)**:VP7

### Aims

We sought to evaluate the validity of three patient-reported outcome measures (PROMs), the Pediatric Quality of Life Inventory (PedsQL), the Revised Children’s Anxiety and Depression Scale-25 (RCADS-25), and Columbia-Suicide Severity Rating Scale-short form (C-SSRS), for use in the routine clinical care of children and adolescents in intensive outpatient mental health services.

### Methods

We used an argument-based approach to validation, a process where evidence to support a proposed interpretation and use of a measure (in a particular population and context) is collected to provide a sound scientific argument for its use. Formulating a validity argument can be thought of as a three-step process: 1) State the purpose of measurement; 2) State the inferences and assumptions made when the instrument is used; 3) Evaluate the evidence to support those inferences. To evaluate the validity evidence, we used a mixed-methods, secondary analysis of interview and survey data from an MBC implementation evaluation in conjunction with quantitative analysis of PROMs data collected through the routine clinical use of these PROMs. The PROMs data was collected from both children (all three PROMs) and their caregivers (PedsQL and RCADS-25 only) in an intensive outpatient mental health service for children 6–18 years of age.

### Results

The PROMs appear to comprehensively cover key domains relevant to child and adolescent mental health intensive outpatient treatment. There is preliminary evidence that children’s scores accurately reflect their symptoms and functioning and pick up change in these constructs, however, potential issues with response processes were identified with the C-SSRS and the school domain of PedsQL. For caregiver proxy-reports, potential response process issues were identified with the PedsQL and RCADS-25, which warrant further investigation.

### Conclusion

There is evidence to support the use of these PROMs for MBC in child and adolescent mental health. However, further investigation is needed into responses processes, internal structure, and to establish clinically meaningful thresholds to improve interpretability, to ensure the validity of their use.

## VP8 Key informant experiences with patient-reported outcome measures in child and adolescent mental health

Erin McCabe^1^, Marta Ravani^2^, Zahra Laachinani^3^, Bishnu Bajgain^4^, Jennifer Zwicker^5^, Maria Santana^4^

^1^Department of Physical Therapy, University of Alberta, Edmonton, Alberta, Canada, ^2^Queen’s University, Kingston, Ontario, Canada, ^3^Faculty of Rehabilitation Medicine, University of Alberta, Edmonton, Alberta, Canada, ^4^Community Health Sciences, University of Calgary, Calgary, Alberta, Canada, ^5^School of Public Policy, University of Calgary, Edmonton, Alberta, Canada

*Journal of Patient-Reported Outcomes 2026*, **10(Suppl 1)**:VP8

### Aims

Measurement-based care (MBC) is the routine use of patient-reported outcome measures (PROMs) to direct treatment planning in mental health care. It provides clinicians with real-time objective measures of symptoms and functioning, and organizations with outcomes data to drive decision-making. Despite its promises, few studies have investigated clinician and administrator’s perspectives of MBC in child and adolescent mental health services. The objective of this study was to describe clinician and leadership experiences with MBC.

### Methods

This study uses a qualitative description approach. Data were collected as part of a comprehensive implementation evaluation of the MBC at a child and adolescent mental health centre in Calgary, Canada. Semi-structured interviews were conducted with clinicians and administrators. Analysis for this study focused on interview questions related to their attitudes and experiences with using MBC. We used deductive, directed content analysis to analyze interview data, using benefits and drawbacks at the micro- (individual), meso- (service) and macro- (system) levels as the initial coding framework.

### Results

Managers (n=3), clinical supervisors (n=4) and clinicians (n=9) were interviewed (2 men, 14 women). Clinicians consisted of clinical social workers, psychologists, and nurses. Four categories of results were identified: 1) Benefits at micro level – MBC is perceived to provide comprehensive data to guide care, it enhances client experiences of care, and benefits the clinicians’ professional practice; 2) Benefits at the macro-level – provides data for service evaluation and to evaluate client population needs, provides outcomes to demonstrate value to funders; and 3) Drawbacks at micro level – additional burden for clients and families; 4) Drawbacks at macro level – requires additional resources to operate. Benefits were perceived to outweigh drawbacks.

### Conclusion

This study provides insights into clinician and leadership experiences of MBC in child and adolescent mental health. It will inform future decisions to implement MBC in similar settings, and guide the selection of targeted implementation strategies.

## VP9 Psychometric evaluation of the PROMIS SD SF 8b and ISI in women experiencing sleep disturbances associated with menopause: reliability, validity, and responsiveness

Andrew Trigg^1^, Melissa Barclay^2^, Helena Bradley^2^, Rowena Jones^2^, Christian Seitz^3^, Frank Kramer^4^, Huda Shalhoub^3^

^1^Bayer Plc, Reading, UK, ^2^Adelphi Values Ltd., Bollington, UK, ^3^Bayer AG, Berlin, Germany, ^4^Bayer AG, Wuppertal, Germany

*Journal of Patient-Reported Outcomes 2026*, **10(Suppl 1)**:VP9

### Aims

Sleep disturbances are one of the most bothersome and debilitating symptoms that can occur during the peri and postmenopausal transition. This study evaluated the psychometric properties of the PROMIS Sleep Disturbance Short Form 8b (PROMIS SD SF 8b) and Insomnia Severity Index (ISI) in women with sleep disturbances associated with menopause.

### Methods

PROMIS SD SF 8b and ISI data collected at baseline and over 12 weeks was analyzed from a double-blind, randomized, parallel-group, placebo-controlled Phase 2 study investigating the efficacy and safety of elinzanetant for the treatment of sleep disturbances associated with menopause (NIRVANA; NCT06112756). Analyses assessed distributional properties, reliability, validity, responsiveness and minimal detectable change (MDC90).

### Results

Data from 110 trial participants were used for analyses (mean age: 54.8 years, range: 43– 65 years). No problematic floor and ceiling effects were observed across analyzed timepoints. Composite reliability (Cronbach’s alpha coefficients 0.907-0.931 for PROMIS SD SF 8b, 0.858-0.881 for ISI) and unidimensionality (ratio of first to second eigenvalue >4) was supported for each measure. Good to excellent test-retest reliability was found for the PROMIS SD SF 8b T-score between Weeks 6 and 7, and Weeks 8 and 9 (intra-class correlation coefficients (ICCs) 0.910-0.927). Good test-retest reliability was found for the ISI between Week 4 and 12 (ICC 0.819). Convergent and divergent correlations with measures of similar/distinct concepts were mostly consistent with pre-specified hypotheses. Significant differences in scores (p<0.0001, effect sizes ≥0.69) were observed between subgroups considered a priori to be clinically distinct. Responsiveness at Weeks 4 and 12 indicated significant differences in mean changes between ‘improved’, ‘stable’ and ‘worsened’ participants (p<0.05; effect sizes for improvement 1.97-2.58 for PROMIS SD SF 8b T-score, 1.64-2.51 for ISI Total). MDC90 was 3.890 for PROMIS SD SF 8b T-score and 3.991 for ISI Total score.

### Conclusion

Findings provide evidence supporting the reliability, validity, and responsiveness of the PROMIS SD SF 8b T-score and ISI Total score to assess efficacy endpoints in sleep disturbances associated with women experiencing menopause. Interpretative thresholds based on measurement error should be supplemented with anchor-based thresholds in future studies with larger sample sizes.

## VP10 Symptom prevalences and changes among gastrointestinal cancer patients receiving chemotherapy

Chengbo Zeng^1^, Kelsey Lau-Min^2^, Nneka Ufere^2^, Manraj Kaur^1^, Jason Liu^1^, Andrea Pusic^1^, Maria Edelen^1^

^1^Brigham and Women’s Hospital, Boston, Massachusetts, USA, ^2^Massachusetts General Hospital, Boston, Massachusetts, USA

*Journal of Patient-Reported Outcomes 2026*, **10(Suppl 1)**:VP10

### Aims

Chemotherapy as the mainstay of treatment for gastrointestinal (GI) cancers often causes many co-occurring symptoms. Routine symptom assessment using patient-reported outcome measures (PROMs) can inform tailored management strategies, prevent premature treatment discontinuation, and improve overall outcomes. We used routinely collected PROMs to investigate prevalences and changes of symptoms in a cohort of GI cancer patients receiving chemotherapy.

### Methods

Eligible participants were adult patients with GI cancers who (1) received chemotherapy as their primary treatment and (2) completed any symptom assessment within 15 days of chemotherapy initiation (i.e., the start of a new chemotherapy episode), or at one, two, or three months following the chemotherapy. Symptom data were collected using the Patient-Reported Outcome (PRO) version of the Common Terminology Criteria for Adverse Events (PRO-CTCAE®), which evaluates 12 common chemotherapy-related symptoms: constipation, decreased appetite, diarrhea, fatigue, fever, insomnia, nausea, paresthesia, pain, rash, dyspnea, and vomiting. Each symptom was scored on a 0 to 3 scale, with scores ≥1 considered indicative of symptom presence. We summarized the most prevalent symptoms and used repeated measures ANOVA to identify those with significant changes over time.

### Results

The analytic cohort included 1,673 patients. Of these, 80% were non-Hispanic White, and more than half of them were male and/or older adults (aged > 64 years). Colorectal (36%) and pancreatic (29%) cancers were the most common diagnoses. Over 80% reported at least two symptoms, with fatigue, pain, and insomnia being the most prevalent. Among 973 patients with at least two PRO-CTCAEs, diarrhea, fatigue, nausea, and paresthesia significantly increased by three months. Constipation and insomnia scores increased at one month and decreased by three months, while pain scores significantly decreased across all three post-initiation time points (Figure 1). Overall, there were significant changes in existing symptoms (i.e., fatigue, pain, insomnia, constipation), while paresthesia, diarrhea, and nausea emerged as new or worsening symptoms over time.

### Conclusion

Patients living with GI cancers receiving chemotherapy experienced significant symptom burden over time, especially for fatigue, pain, and insomnia. Using PROMs for routine symptom assessment can help monitor these changes and support clinical decision-making.

**Fig. 1:**
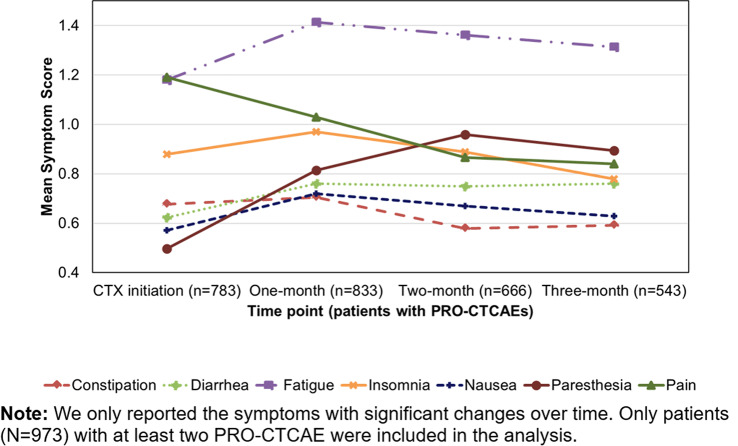
Selected PRO-CTCAE symptom scores across the four time points

## VP11 Bridging Technology and Trust: Leveraging AI and Health Behavior Theory to Combat Misinformation in African American Communities

Akorfa Adobor^1^, Tyson Le^2^, Tatenda Makoni^1^, Philisha Mesidor^1^

^1^MCW, Milwaukee, Wisconsin, USA, ^2^MCW, Wauwatosa, Wisconsin, USA

*Journal of Patient-Reported Outcomes 2026*, **10(Suppl 1)**:VP11

### Aims

African-American communities are disproportionately affected by health misinformation. Artificial intelligence has shown great promise in improving health communication. However, a gap exists between the theoretical application of AI and previously established behavior models. Addressing this gap can help combat misinformation in racially marginalized communities. This project aims to provide a conceptual framework for the integration of AI into health behavior theory in hopes of developing a culturally targeted intervention to address misinformation.

### Methods

This theoretical framework centers around the Health Belief Model (HBM) and Information Diffusion Theory to describe how AI-driven technologies like large language models and recommendation systems can help facilitate health behavior change. The Cultural Mistrust Framework plays a critical role by acknowledging and addressing the well-established history of medical mistrust within the African-American community. Additionally, a comprehensive review of AI-powered health tools and communication strategies will aid the development of our theoretical framework.

### Results

We propose a novel theoretical approach that focuses on AI’s potential to serve as a personalized communication method. AI’s ability to engage individuals using a data-driven approach can help individuals modify health behaviors, while simultaneously increasing one’s ability to confidently engage with medical information. Our framework demonstrates how AI-powered interventions that are created with cultural sensitivity in mind can improve the accessibility of health information within the African-American community.

### Conclusion

This theoretical framework fills the gaps that currently exist in health communication literature by integrating AI into previously established health behavior models, with the ultimate goal of addressing misinformation in African American communities.

## VP12 How AI Can Help Sickle Cell Disease Management in African American Communities

Akorfa Adobor^1^, Stephen Stevanovic^1^

^1^MCW, Milwaukee, Wisconsin, USA

*Journal of Patient-Reported Outcomes 2026*, **10(Suppl 1)**:VP12

### Aims

Sickle Cell Disease (SCD) is a hereditary hematologic disorder that disproportionately affects African Americans in the United States. Despite its prevalence in this population, disparities in diagnosis, treatment access, and long-term outcomes remain profound. Emerging advances in artificial intelligence (AI) may offer novel solutions to these systemic inequities by enhancing disease management, resource allocation, and patient engagement.

### Methods

This project will include a comprehensive review of literature regarding the current landscape of AI-powered applications for SCD management and assess their potential to reduce disparities among African American communities.

### Results

AI innovations demonstrated value in early identification of vaso-occlusive crises through predictive modeling, enhanced diagnostic precision via radiographic and lab-based tools, and improved patient outcomes through mobile health platforms supporting real-time symptom tracking. Notably, AI-driven integration of social determinants of health enabled targeted intervention strategies. However, recurring challenges included algorithmic bias, underrepresentation of African American populations in training datasets, and limited access to AI tools among underserved communities.

### Conclusion

Artificial intelligence holds substantial promise in transforming SCD care through improved prediction, personalization, and equity-driven resource allocation. Realizing this potential requires inclusive data practices, community-centered design, and culturally tailored implementation strategies to mitigate digital and structural disparities. AI must be applied ethically to ensure it serves as a vehicle for equity rather than exacerbating existing gaps in care.

## VP13 Estimating Meaningful Score Differences and Meaningful Score Regions for the PROMIS® Pediatric Asthma Impact Scale

Jing Yuan^1^, Li Lin^2^, Kevin Weinfurt^2^, Nicole Lucas^2^, Allison Burbank^3^, Michelle Hernandez^3^, I-Chan Huang^4^, Bryce Reeve^1^

^1^Duke University, Durham, North Carolina, USA, ^2^Duke University School of Medicine, Durham, North Carolina, USA, ^3^University of North Carolina School of Medicine, Chapel Hill, North Carolina, USA. I-Chan Huang^4^St. Jude Children’s Research Hospital, Memphis, USA

*Journal of Patient-Reported Outcomes 2026*, **10(Suppl 1)**:VP13

### Aims

The Patient-Reported Outcomes Measurement Information System® (PROMIS®) Pediatric Asthma Impact Scale is used in clinical research to assess the impact of asthma on children’s daily activities and quality of life. The purpose of this study is to improve the interpretability of scores from the PROMIS® Pediatric Asthma Impact Scale by estimating meaningful score differences (MSDs) and meaningful score regions (MSRs) using an anchor-based approach.

### Methods

This secondary analysis involved 106 children aged 8–17 years with asthma who completed weekly patient-reported outcome assessments over four weeks. Associations between PROMIS Asthma Impact Scale scores and scores from other anchor measures were examined using repeated measures correlations. MSDs for improvement and deterioration were derived using mixed-effects models anchored to the Global Impact of Change (GIC) items related to changes in their asthma severity and general health. MSRs were established using the receiver operating characteristics (ROC) curve analyses with three anchors: Self-Reported Asthma Symptom Rating (ASR), Global Initiative for Asthma (GINA) control criteria, and Asthma Control Test (ACT) or Childhood Asthma Control Test (cACT).

### Results

PROMIS Asthma Impact Scale scores had moderate associations with the anchor measures. MSDs were 2.3–2.5 points for improvement and 3.5–3.6 points for deterioration. ROC analyses yielded MSRs that differentiated meaningful levels of asthma impact: 38.7 and 49.2 based on GINA categories (between controlled, partly controlled, and uncontrolled asthma); 45.6 based on ACT/cACT (between controlled and uncontrolled); and 47.9, 50.1, and 55.8 based on ASR (between very good, good, a little good, and bad).

### Conclusion

This study enhances score interpretability of the PROMIS® Pediatric Asthma Impact Scale by estimating MSDs and MSRs, aligned with the FDA’s Patient-Focused Drug Development (PFDD) guidance. These estimates support more informed use of PROMIS scores in pediatric asthma research and care, and offer a model for applying MSD and MSR methods to other clinical outcome assessments.

**Fig. 1 (abstract VP13):**
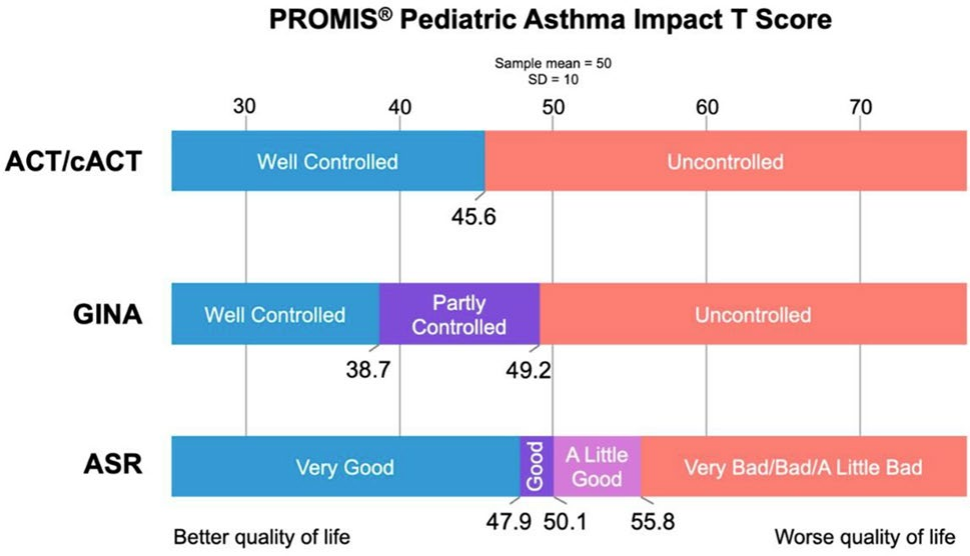
Estimating Meaningful Score Differences and Meaningful Score Regions for the PROMIS® Pediatric Asthma Impact Scale

## VP14 Patient preference information for HTA: A rapid review of HTA community perspectives

Takako Kaneyasu^1^

^1^Kinugasa Research Organization, Ritsumeikan University, Kyoto, Japan

*Journal of Patient-Reported Outcomes 2026*, **10(Suppl 1)**:VP14

### Aims

Patient Preference Information (PPI) has been discussed in the context of patient-centered care for nearly a decade. In recent years, specific considerations have been made in regulatory science. Although various reports have been published on the issues of PPI in health technology assessment (HTA), most have focused on technical issues, and there has been little discussion on how to adopt them. Through a rapid review, this study identified situations in which PPI is useful, useful stages of HTA, and challenges in integrating PPI with HTA.

### Methods

In this study, literature covering three years (2018-2025), that is, before and after the release of the PREFER recommendations, was targeted. Those on “health technology assessment” and health “preferences” were searched using the MEDLINE and Embase databases. Literature related to utility estimation or multicriteria decision analysis was excluded. Predefined information such as “cases and HTA stages where PPI is considered valuable” was extracted from the collected literature.

### Results

Approximately 350 studies were identified from the two databases. After a detailed review, five were selected for data extraction. Most studies were conducted targeting professionals in HTA agencies. HTA professionals considered PPI as an element that was not included in the health economic evaluation. They believe that when there are important non-health benefits of technology or when the treatment is applied to a heterogeneous population, the value of PPI increases. PPI plays an important role in the initial and final stages of HTA, and preference heterogeneity is the most critical issue.

### Conclusion

The use of PPI has expanded from the evaluation of medical devices to regulatory science; however, owing to issues such as heterogeneity, it has been considered difficult to utilize PPI in economic evaluations in HTA. However, PPI is perceived to be useful in the prioritization, scoping and deliberative processes of HTA. Accumulation of information on how PPI contributes to quality improvement in HTA is expected in the future.

## VP15 Exploring health-related quality of life and patient perceptions in persons with above-knee bone-anchored prostheses: a mixed methods study

Mayank Rehani^1^, Christine Guptill^2^, Jeff Round^3^, C. Allyson Jones^4^, Jacqueline Hebert^1^

^1^Division of Physical Medicine & Rehabilitation, Department of Medicine, Faculty of Medicine & Dentistry, University of Alberta, Edmonton, Alberta, Canada, ^2^School of Rehabilitation Sciences, Faculty of Health Sciences, University of Ottawa, Ottawa, Ontario, Canada, ^3^School of Public Health, University of Alberta, Edmonton, Alberta, Canada, ^4^Department of Physical Therapy, Faculty of Rehabilitation Medicine, University of Alberta, Edmonton, Alberta, Canada

*Journal of Patient-Reported Outcomes 2026*, **10(Suppl 1)**:VP15

### Aims

Bone-anchored (osseointegrated) prostheses are an innovative surgical approach for persons with an above-knee amputation who cannot use conventional sockets for their prostheses. Most published studies assessing HRQoL following osseointegration are based on quantitative methods. Lived experiences contributing to the change in perceived HRQoL have not been fully explored as an individual transitions from socket to bone-anchored prosthesis use. This study aims to explore these HRQoL changes using a convergent mixed methods approach.

### Methods

Data is collected at three time points: baseline as a socket user, 6 months after surgery (following intensive rehabilitation), and 12 months after surgery. These time points enable comparisons between an individual’s health states. Quantitative data includes a generic instrument (SF-36) and a condition-specific instrument (Questionnaire for Persons with a Transfemoral Amputation; Q-TFA). Open-ended qualitative interviews are conducted at the three time points using the longitudinal qualitative research (LQR) approach. Quantitative and qualitative data will be analyzed separately and mixed at the integration stage. Metainferences will be drawn about perceptions of HRQoL that change over time.

### Results

Preliminary data from six participants show improvements in the physical component and related subscores of the SF-36 throughout the 12 months; however, no remarkable changes in the mental component score. The Q-TFA prosthetic use, problems, and global scores improved notably from baseline to 6 months, which were maintained at 12 months. The qualitative aspect revealed that before OI, participants experienced varying degrees of discomfort and substantial issues with socket fit, and they had to consistently manage their lives around their prosthetic limb. These issues were resolved over 12 months post-surgery. All six participants reported steady gains in their quality of life over 12 months after surgery, which was driven by an increase in the ability to carry out activities of daily living that were more meaningful to them.

### Conclusion

Bone-anchored prostheses contribute to positive change in an individual’s perception of their HRQoL. The knowledge gained from this study will lead to a more nuanced understanding of change in HRQoL in prosthesis users. It will also advance the use of LQR and mixed methods to evaluate transitions in health states following an intervention.

## VP16 A reconceptualization of Maslow: A chronic illness-informed framework for health-related quality of life

Angel Sheu^1^, Raika Bourmand^1^

^1^Anne Burnett Marion School of Medicine at Texas Christian University, Fort Worth, Texas, USA

*Journal of Patient-Reported Outcomes 2026*, **10(Suppl 1)**:VP16

### Aims

Health-Related Quality of Life (HRQL) is often conceptualized using frameworks such as Maslow’s Hierarchy of Needs, which features a progression from physiological survival to self-actualization. However, in patients with chronic illness, the experience of multimorbidity often leads to a re-prioritization of values and needs. We propose a modified hierarchy of needs that reflects the lives of those with chronic conditions. This modified hierarchy integrates psychological, functional, and social literature to better align with HRQL concepts.

### Methods

Through theory synthesis, we integrate ideas from Maslow’s original theory, Response Shift Theory, and patient-centered literature on chronic illness/multimorbidity. These key themes from quantitative and qualitative studies were then mapped to construct a revised needs hierarchy that emphasizes autonomy, functionality, and adaptation.

### Results

Based on findings from literature from patients who struggle with chronic illness, internal standards and priorities shift towards self agency. The proposed model reorders Maslow’s pyramid, placing autonomy/decision-making control at the top, followed by functional independence, emotional resilience, physical comfort, and social connection. In contrast to the original theory, qualitative and quantitative studies in chronic illness populations demonstrate that autonomy, functional independence, and psychological adaptation often take precedence. Patients often identify these factors as being essential for maintaining dignity and HRQL, even when physical symptoms are severe. Functional status was also found to be more highly correlated with perceived HRQL than symptom burden. Furthermore, many patients report stable or even improved HRQL despite progressing disease over time, indicating a shift in priorities.

### Conclusion

This revised framework offers a more accurate theoretical lens for understanding HRQL within populations that have chronic illnesses. This concept may inform the interpretation of patient-reported outcome measures, as well as inform interventions to ensure alignment with patient priorities.

**Fig. 1 (abstract VP16):**
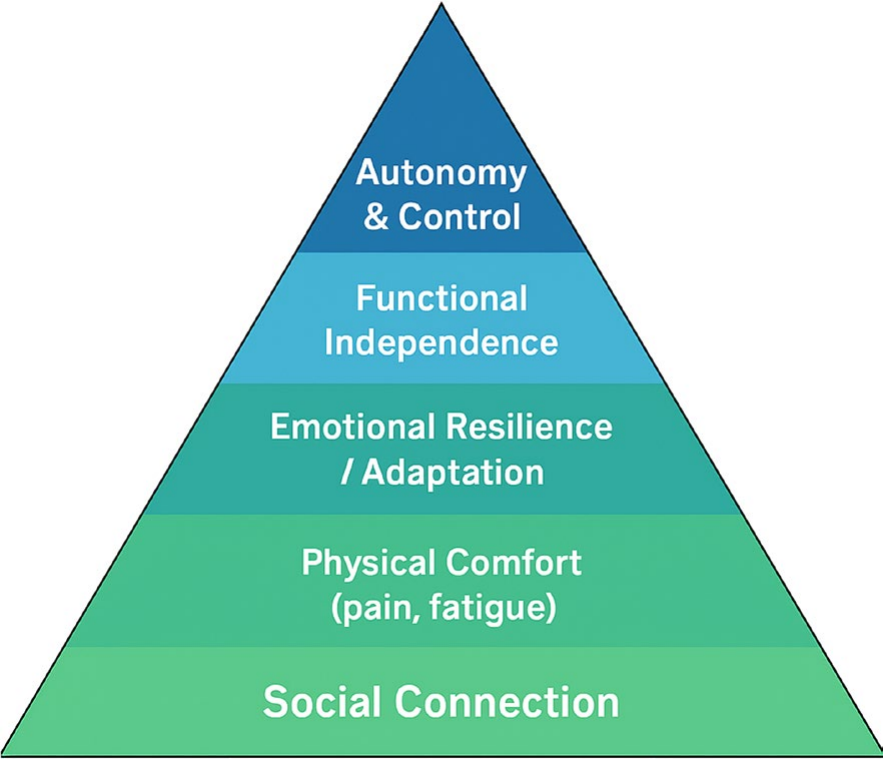
A reconceptualization of Maslow: A chronic illness-informed framework for health-related quality of life

## VP17 How, for Whom, under What Circumstances, and Why is Mental Healthcare for Anxiety Supposed to Improve Well-being for People Receiving Dialysis?

Lori Suet Hang^1^, Richard Sawatzky^2^, Joanne Greenhalgh^3^, Kara Schick-Makaroff^4^

^1^Faculty of Nursing, University of Alberta, Edmonton, Alberta, Canada, ^2^School of Nursing, Trinity Western University, Vancouver, British Columbia, Canada, ^3^School of Sociology and Social Policy, University of Leeds, Leeds, UK, ^4^Faculty of Nursing, University of Alberta, Edmonton, Alberta, Canada

*Journal of Patient-Reported Outcomes 2026*, **10(Suppl 1)**:VP17

### Aims

People receiving dialysis commonly experience anxiety symptoms and low quality of life, yet the problem remains under-addressed. Due to the complex interplay of contextual factors in mental healthcare at individual and institutional levels, a contextually-nuanced initial program theory is needed to guide optimal provision of mental healthcare to improve well-being for people receiving dialysis. This theoretical knowledge can explain how contexts (for whom and under what circumstances) may influence the mechanisms (how and why) and shape outcomes (perceived anxiety symptoms and quality of life). The outcomes include subjective perspectives of people receiving dialysis, measured by patient-reported outcome measures. This project aims to explain how, for whom, under what circumstances, and why mental healthcare for anxiety is supposed to improve well-being for people receiving dialysis.

### Methods

A realist synthesis, through a complexity-informed lens, was undertaken to develop initial program theories. We searched literature via databases and websites using search terms derived from dialysis, anxiety, and care, and recommended documents. Community Advisory Committee members were consulted.

### Results

Theories were identified to explain how features experienced by people may shape responses to care, and influence the mechanisms through which mental healthcare is expected to work. Contextual differences in people’s lives may influence how they receive mental healthcare. Taking a person-centered approach, a person may be more willing to share when feeling heard and understood. Developed rapport and shared decision-making may support continuous conversations and continuity of care. Other contextual factors limiting clinicians’ capacity to provide mental healthcare may be staffing, training, resources, perceived “scope”, and fragmented healthcare systems. A person navigating siloed care may feel confused and frustrated, and lose their trust in the care being provided. Taking up an integrated care model may facilitate communications and support provision of care, and ultimately improve patient-reported outcomes, anxiety symptoms, and well-being for people receiving dialysis.

### Conclusion

The theory may inform policy makers by explaining how specific contexts trigger mechanisms that lead to patient-reported outcomes. The theory will provide a theoretical framework to help identify factors that determine the success of mental healthcare, guide intervention development, and support the implementation of person-centered mental healthcare in the real-world practice.

## VP18 Identifying and explaining variation in emotional well-being measurement bias

Richard Sawatzky^1^, Mathilde Verdam^2^, Ava Mehdipour^1^, Pamela A. Ratner^3^, Karen Courtney^4^, Carl F. Falk^5^, Anne Gadermann^6^, Jeanette Jackson^7^, Jae-Yung Kwon^8^, Lisa M. Lix^9^, Juxin Liu^10^, Joseph J. O’Rourke^11^, Tolulope T. Sajobi^12^, Cathy Son^13^, Kara Schick-Makaroff^14^, Hubert Wong^15^, Bruno D. Zumbo^16^

^1^Trinity Western University, Langley, British Columbia, Canada, ^2^Department of Methodology and Statistics, Institute of Psychology, Leiden University, Leiden, Netherlands, ^3^University of British Columbia, Vancouver, British Columbia, Canada, ^4^University of Victoria - Health Information Science, Victoria, British Columbia, Canada, ^5^McGill University - Psychology, Montreal, Quebec, Canada, ^6^University of British Columbia - School of Population and Public Health, Vancouver, British Columbia, Canada, ^7^Health Quality Council of Alberta, Calgary, Alberta, Canada, ^8^University of Victoria - School of Nursing, Victoria, British Columbia, Canada, ^9^University of Manitoba - Max Rady College of Medicine, Winnipeg, Manitoba, Canada, ^10^University of Saskatchewan - Mathematics and Statistics, Saskatoon, Saskatchewan, Canada. Joseph J. O’Rourke^11^St. Michael’s Hospital, MAP Centre for Urban Health Solutions, Toronto, Ontario, Canada, ^12^University of Calgary - Community Health Sciences, Calgary, Alberta, Canada, ^13^Trinity Western University, School of Nursing, Langley, British Columbia, Canada, ^14^University of Alberta, College of Health Sciences, Faculty of Nursing, Edmonton, Alberta, Canada, ^15^University of British Columbia - School of Population and Public Health, Vancouver, British Columbia, Canada, ^16^University of British Columbia - Educational & Counselling Psychology, and Special Education, Vancouver, British Columbia, Canada

*Journal of Patient-Reported Outcomes 2026*, **10(Suppl 1)**:VP18

### Aims

Ensuring unbiased measurements that represent the perspectives of diverse people is foundational to equitable health care. As part of our research on “Equitable People-Centred Health Measurement” (http://www.healthyqol.com), we examined emotional well-being and the extent to which: a) responses to emotional well-being items are heterogeneous, and b) social determinants of health (SDOH) and health-related variables are associated with measurement bias.

### Methods

Data were obtained via an online survey of 10,076 adults in Canada with diverse SDOH. The questionnaire included: a) the “Emotional Well-Being” item bank (43 items) of the CAT-5D-QOL; b) The “Screening for Poverty and Related social determinants to improve Knowledge of and links to resources” (SPARK) tool to collect information about SDOH, including demographics (e.g., immigration status, gender identity, racial background), social needs (e.g., income, housing status, social isolation) and disability status; and c) health-related variables (e.g., health conditions, healthcare utilization, medications). Latent variable mixture models (LVMMs) were used to examine heterogeneity by varying measurement model parameters across multiple latent classes. Measurement bias was estimated as the differences between standardized emotional well-being scores from a 1-class model (assuming no heterogeneity) and a k-class model (accommodating heterogeneity). Multivariable linear regression models were used to explain variation in the estimated positive and negative measurement bias. The Pratt Index (d) was used to measure variable importance as the percentage of total explained variance.

### Results

The sample was heterogeneous, with optimal results obtained for a 4-class model (class proportions = .09, .08, .41, and .41; entropy = .88; Bayesian Information Criterion = 824437 and 740956 for 1- and 4-class models, respectively). Positive measurement bias ranged from 0.01 to 0.54 for 41% of the sample and was predominantly explained by healthcare utilization, demographics and social needs (d = 30%, 29%, 29%, respectively; total R2 = 30%). Negative measurement bias ranged from -0.01 to -0.31 for 59% of the sample and was predominantly explained by social needs, demographics, and medications (d = 33%, 18%, 16%, respectively; total R2 = 10%).

### Conclusion

Ignoring SDOH and various health-related differences could result in biased measurements of emotional well-being, which may lead to some people’s perspectives of their emotional well-being being misrepresented.

## VP19 Quality-of-life discounting: A behavioral economics framework for understanding the underutilization of preventive health interventions

Angel Sheu^1^, Raika Bourmand^1^

^1^Anne Burnett Marion School of Medicine at Texas Christian University, Fort Worth, Texas, USA

*Journal of Patient-Reported Outcomes 2026*, **10(Suppl 1)**:VP19

### Aims

Although many patients recognize that preventive care can improve long-term health outcomes and health-related quality of time (HRQL), many patients forego screenings, chronic disease monitoring, and appointments for lifestyle modifications. Some explanations for this underutilization docs on access, patient knowledge, and motivation, but these fail to explain patterns observed across diverse populations. By applying principles from behavioral economics, specifically delay discounting, loss aversion, and status quo bias, we explain why patients may under prioritize long-term HRQL benefits in favor of short-term convenience or avoidance.

### Methods

A synthesis of empirical findings from published studies on health behavior, patient-reported quality of life, and preventive care uptake was conducted. This literature was interpreted through a lens of behavioral economics. Patient decisions were reframed through predictable outcomes as a result of cognitive biases rather than noncompliant or irrational behavior.

### Results

Behavioral economic models suggest that patients can discount future health improvements as the immediate costs of discomfort, inconvenience, or fear regarding preventive care appear larger than they are. This phenomenon is what we term the “Quality of Life discount.” Loss aversion may also cause patients to overestimate the possibility of adverse information such as negative test results when compared to long-term benefits. These concepts potentially explain counterintuitive findings such as lower HRQL in patients avoiding care, despite them being asymptomatic.

### Conclusion

By integrating behavioral economics into HRQL theory, we offer a novel framework for the interpretation of patient decision-making. Through recognizing the QOL discount, clinicians can guide intervention design to reduce perceived barriers, reframe benefits, and allow for effective health management. This theoretical model can benefit the communication between patients and their providers while also measuring preventive impact on long-term HRQL and improving health through preventative measures.

**Fig. 1 (abstract VP19):**
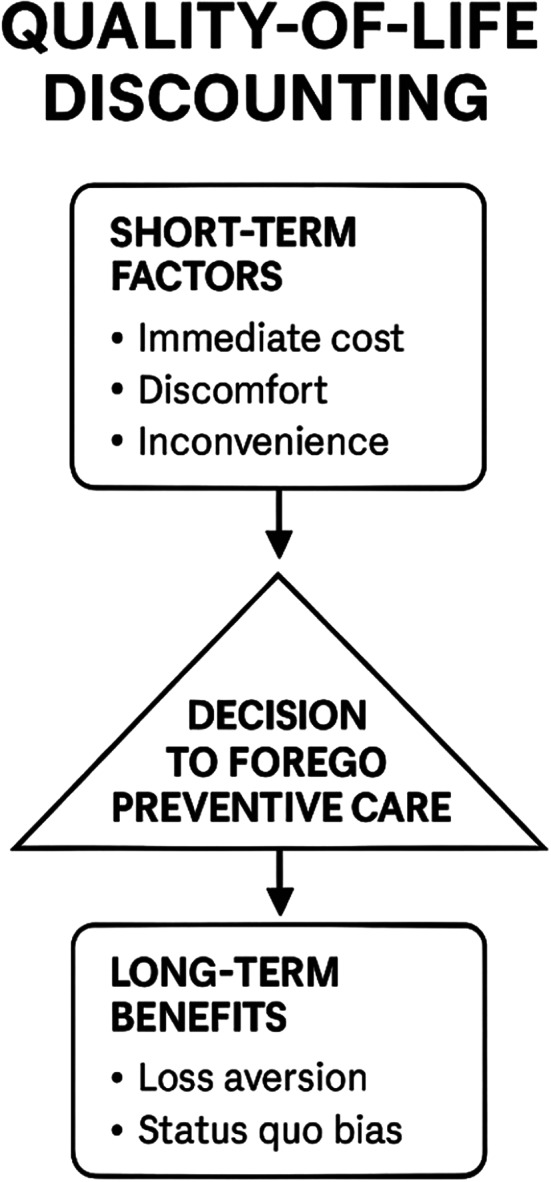
Quality-of-life discounting: A behavioral economics framework for understanding the underutilization of preventive health interventions

## VP20 Beyond the individual: Bronfenbrenner’s ecological systems theory as a proxy for understanding pediatric health-related quality of life

Angel Sheu^1^, Raika Bourmand^1^

^1^Anne Burnett Marion School of Medicine at Texas Christian University, Fort Worth, Texas, USA

*Journal of Patient-Reported Outcomes 2026*, **10(Suppl 1)**:VP20

### Aims

Pediatric Health-Related Quality of Life is typically assessed via tools that focus on physical, emotional, and social functioning. However, the experiences of children are within complex social environments that include family dynamics, school settings, community resources, and healthcare access. These all shape the health and well-being of children in critical ways. Our goal is to apply Bronfenbrenner’s Ecological Systems Theory as a conceptual model for understanding pediatric HRQL to emphasize the multifactorial systems that influence how children experience and report QOL.

### Methods

We synthesize published studies on pediatric HRQL, school functioning, caregiver burden, and the effect of one’s neighborhood. These factors are then mapped onto Bronfenbrenner’s framework, featuring microsystems such as family/school, mesosystems such as parent-provider interactions, exosystems such as caregiver work conditions, macrosystems such as health policies, and chronosystems such as developmental changes.

### Results

This theoretical model reveals how pediatric HRQL is shaped by a child’s context and relationships in addition to individual symptoms. For example, parental/caregiver stress may lower HRQL scores even when the health of a child is relatively stable. In a similar way, school inclusiveness, healthcare navigation complexity, and health policy changes may affect the lived experience of these children and their perceived well-being. Through an application of this ecological model, we can explain disparities that cannot be captured by symptom-based tools alone.

### Conclusion

The Ecological Systems Theory provides a multidimensional framework for advancing our understanding and measurements of pediatric HRQL. By recognizing the influence of broader systems, we are able to encourage the development of instruments that factor in context and construct appropriate interventions. This model holds the potential to improve equity, interpretation of health, and responsiveness within pediatric HRQL research and clinical practice.

**Fig. 1 (abstract VP20):**
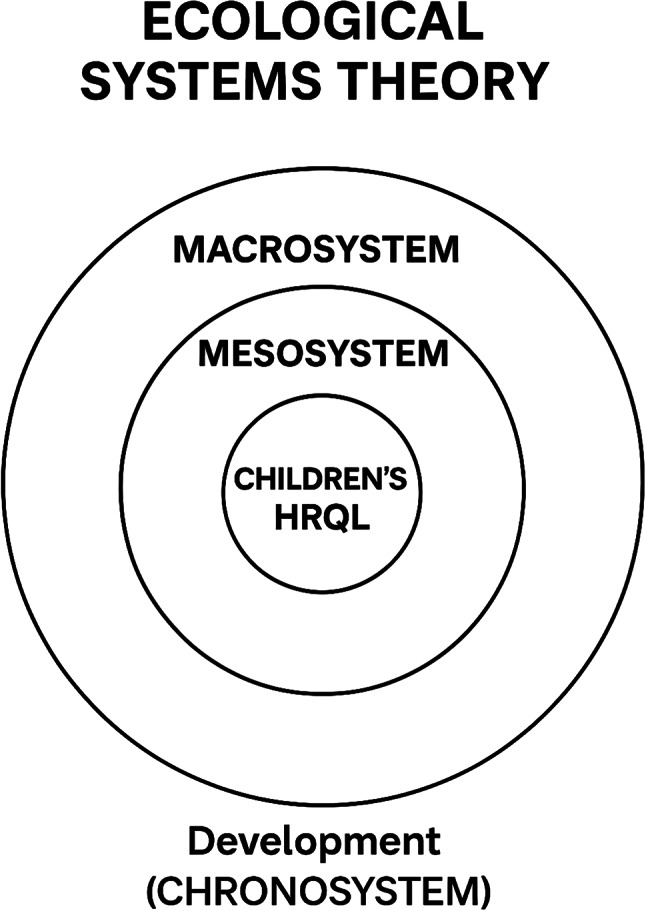
Beyond the individual: Bronfenbrenner’s ecological systems theory as a proxy for understanding pediatric health-related quality of life

## VP21 Mapping the Evidence for Outcomes in Telehealth Across Four Domains: A Scoping Review

Fatemeh Ehteshami^1^, Joy Christian MacDermid^1^

^1^University of Western Ontario, London, Ontario, Canada

*Journal of Patient-Reported Outcomes 2026*, **10(Suppl 1)**:VP21

### Aims: Background and Aim

Telehealth has emerged as a critical component of modern healthcare, offering solutions to improve access, efficiency, and patient satisfaction. However, the rapid expansion of telehealth has outpaced the development of standardized outcome measurement tools, creating challenges for research synthesis and evidence-based practice. This scoping review aimed to identify, map, and evaluate telehealth outcome measurement tools across the four domains defined by the National Quality Forum (NQF): access, cost, experience, and effectiveness. Specifically, it sought to analyze the psychometric properties, development processes, and gaps within the current landscape of telehealth questionnaires.

### Methods

We systematically searched PubMed, Scopus, and Embase using broad terms related to telehealth, outcomes, development, validity, and reliability. A total of 13,067 articles were screened, with 62 studies meeting the inclusion criteria. Tools were categorized based on their focus domains, psychometric rigor, development methodology, and inclusivity features.

### Results

Our review identified considerable heterogeneity in the types, quality, and reporting of telehealth outcome measures. While tools assessing satisfaction and usability were abundant, fewer instruments focused on access, cost, or provider-centered outcomes. Additionally, many questionnaires lacked comprehensive psychometric validation or cross-cultural adaptation, limiting their applicability across diverse populations. Emerging issues such as the integration of AI technologies, ethical considerations, and health equity were rarely addressed in existing tools.

### Conclusion

Despite significant advances, telehealth evaluation remains fragmented, with critical gaps in standardization, inclusivity, and technological responsiveness. There is a pressing need for harmonized frameworks, culturally sensitive instruments, and innovation-adapted measures to advance the field. This review provides a roadmap for researchers and policymakers to guide future development and ensure telehealth systems deliver high-quality, equitable, and evidence-based care.

## VP22 To what extent do older adults differ in how they respond to questions about their physical and mental health?

Ava Mehdipour^1^, Richard Sawatzky^1^

^1^Trinity Western University, Langley, British Columbia, Canada

*Journal of Patient-Reported Outcomes 2026*, **10(Suppl 1)**:VP22

### Aims

Older adults in diverse populations may interpret and respond to patient-reported outcome measures (PROMs) differently, due to their personal and social circumstances, like their social determinants of health (SDOH). Ignoring diversity when analyzing PROMs may lead to inaccurate measurements of health. This study examined 1) heterogeneity in older adults’ responses to a widely used generic PROM, the Veterans Rand 12-item health survey (VR-12), specifically investigating evidence of measurement non-invariance, and 2) potential measurement biases resulting from ignoring heterogeneity.

### Methods

Older adults (≥ 65 years) across Canada participated in an online survey, which included the VR-12, a PROM measuring physical and mental health, and the Screening for Poverty and Related social determinants to improve Knowledge of and links to resources (SPARK) tool, a measure of various SDOH (i.e., demographics, social needs, and disabilities). A 2-factor Item Response Theory (IRT) model was used to reflect both the physical and mental health dimensions of the VR-12. Mixture IRT was applied to examine measurement invariance by allowing measurement model parameters to vary across latent classes (i.e., subpopulations). Measurement bias was calculated as the difference between IRT scores from a 1-class model (assuming invariance) and a k-class model (accommodating non-invariance). Multivariable linear regression models were used to examine associations between SDOH and positive and negative measurement bias.

### Results

Responses on the VR-12 (n=1649) were found to be heterogenous and best represented by a 2-class model (Class proportions = 0.44, 0.56; Bayesian Information Criterion for 1- and 2-class models=36188 and 35990; Loglikelihood Ratio Test = p<0.001). Measurement bias ranged from -1.63 to 2.83 for the physical health dimension (Figure 1) and -0.76 to 1.47 for mental health dimension (Figure 2). SDOH explained 4.9% of variance in positive and 11.6% in negative measurement bias for the physical health dimension, and 14.9% and 7.2%, respectively, for the mental health dimension.

### Conclusion

Older adults were found to respond differently to questions about their health, resulting in measurement biases for people with different SDOH. Researchers and policy analysts can apply mixture IRT models as an approach to enhance equitable measurements of health for older adults.

**Fig. 1 (abstract VP22):**
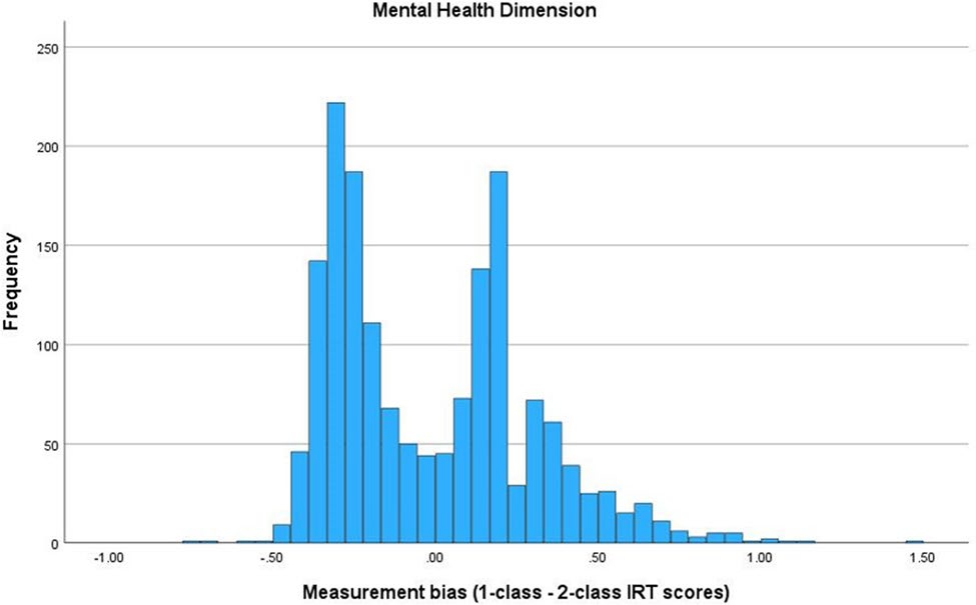
Measurement bias defined as the difference between IRT scores of the 1-class and 2-class model for the physical health dimension of the VR-12

**Fig. 2 (abstract VP22):**
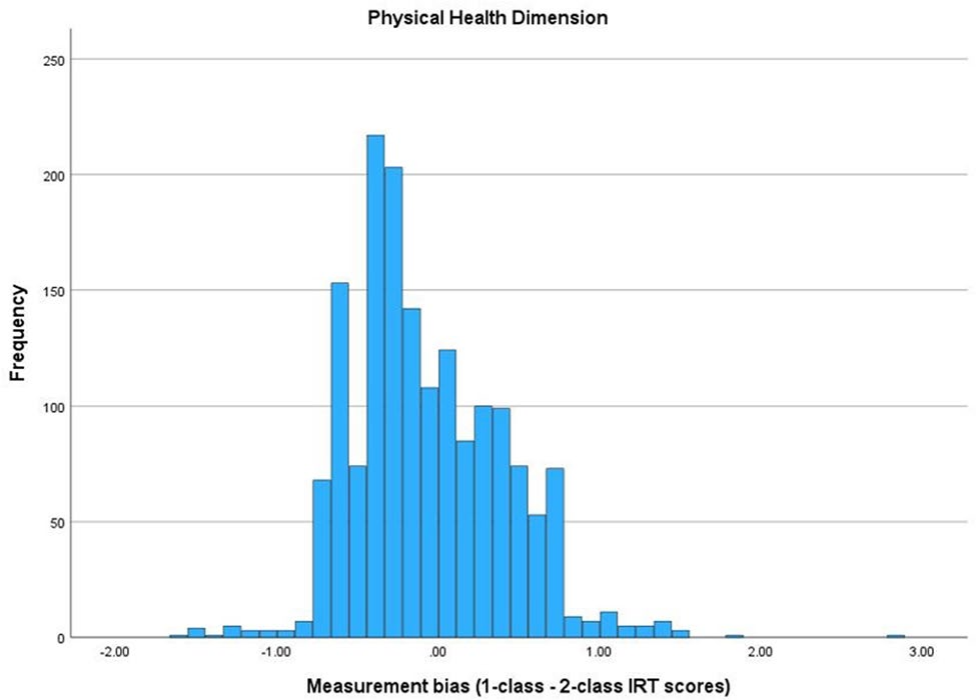
Measurement bias defined as the difference between IRT scores of the 1-class and 2-class model for the mental health dimension of the VR-12

## VP23 Improving communication in primary care using a mixed-methods approach to creating data-driven personas

Jeanette Jackson^1^, Sheliza Ladhani^1^, Ava Mehdipour^2^, Chyloe Healy^3^, Roland Simon^1^, Maz Rahman^4^, Guy DeSantis^1^, Tim Cooke^1^, Jae-Yung Kwon^5^, Richard Sawatzky^6^

^1^Health Quality Council of Alberta, Calgary, Alberta, Canada, ^2^Trinity Western University, Toronto, Ontario, Canada, ^3^Health Quality Council of Alberta, Vancouver, British Columbia, Canada, ^4^Health Quality Council of Alberta, Edmonton, Alberta, Canada, ^5^University of Victoria, Victoria, British Columbia, Canada, ^6^Trinity Western University, Langley, British Columbia, Canada

*Journal of Patient-Reported Outcomes 2026*, **10(Suppl 1)**:VP23

### Aims

The reporting of patient-reported experiences typically emphasizes average experiences. Consequently, experiences of some patients within diverse populations may be rendered invisible or misrepresented. Personas (hypothetical representation of patients) are a tool to help amplify hidden voices by relating responses to patient experience measures to patients’ life stories. A mixed-methods approach to creating data-driven personas (see Figure 1) was used to amplify voices identified through latent class analysis to better understand and attend to unique primary care communication experiences.

### Methods

A latent variable mixture model (LVMM) was applied to six communication-related survey questions from 3,539 people across Alberta to examine heterogeneity (represented as latent classes) of people who interpreted and responded to questions in different ways. Measurement bias was more evident in one of two identified latent classes. Sixteen diverse survey respondents belonging to this class (propensity score greater than 0.8) were invited to participate in an interview, five of whom identified as Indigenous. Transcripts were analyzed according to broad dimensions important in primary care (e.g., health concerns, communication experiences, relationship, preparations). A constellatory approach to qualitative analysis was used for the preliminary mapping of six unique personas. Acknowledging that Indigenous patient experiences are often less attended to, interviews with Indigenous patients were foregrounded as the core constellation of four personas.

### Results

The LVMM resulted in improved fit for a 2-class compared to a 1-class model (improved Bayesian Information criterion and significant Likelihood Ratio Test). Class 2 consisted of 24 per cent of survey respondents. Responses to communication items were more negatively distributed in class 2, relative to class 1. The latent class information together with experiences shared during interviews informed the creation of six unique personas. Figure 2 provides an initial visual of Jesse (persona 1) to better understand experiences of chronic pain management in rural primary care.

### Conclusion

Personas can serve as innovative knowledge translation and communication tools for healthcare practitioners by bringing to the forefront patient stories that have been less attended to. This can ultimately help improve the quality of health services, including enhancing patient-provider communication and tailoring care to individual needs.

**Fig. 1 (abstract VP23):**
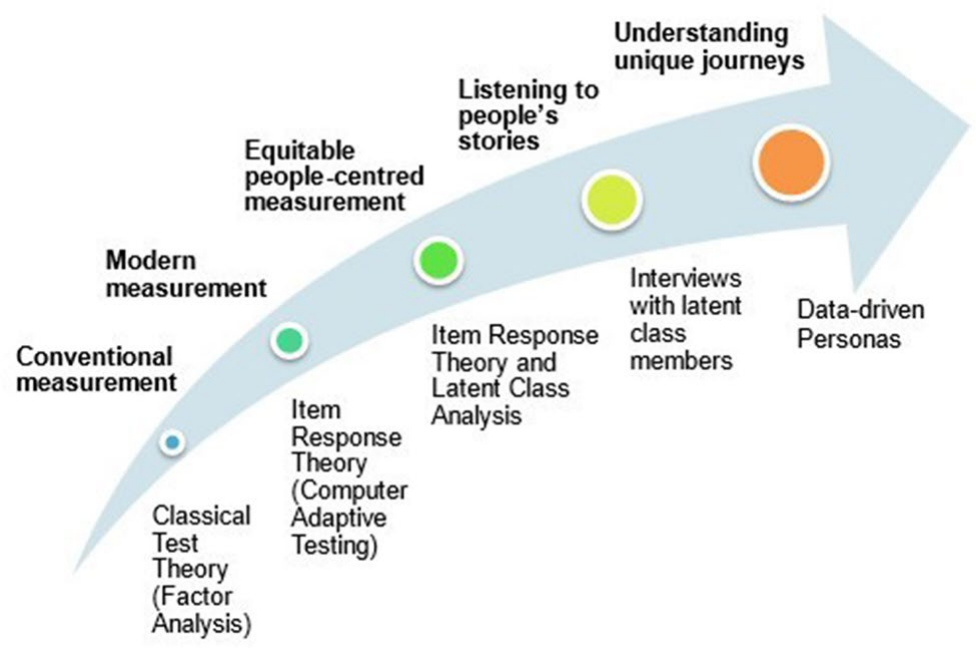
Vision for measuring experience

**Fig. 2 (abstract VP23):**
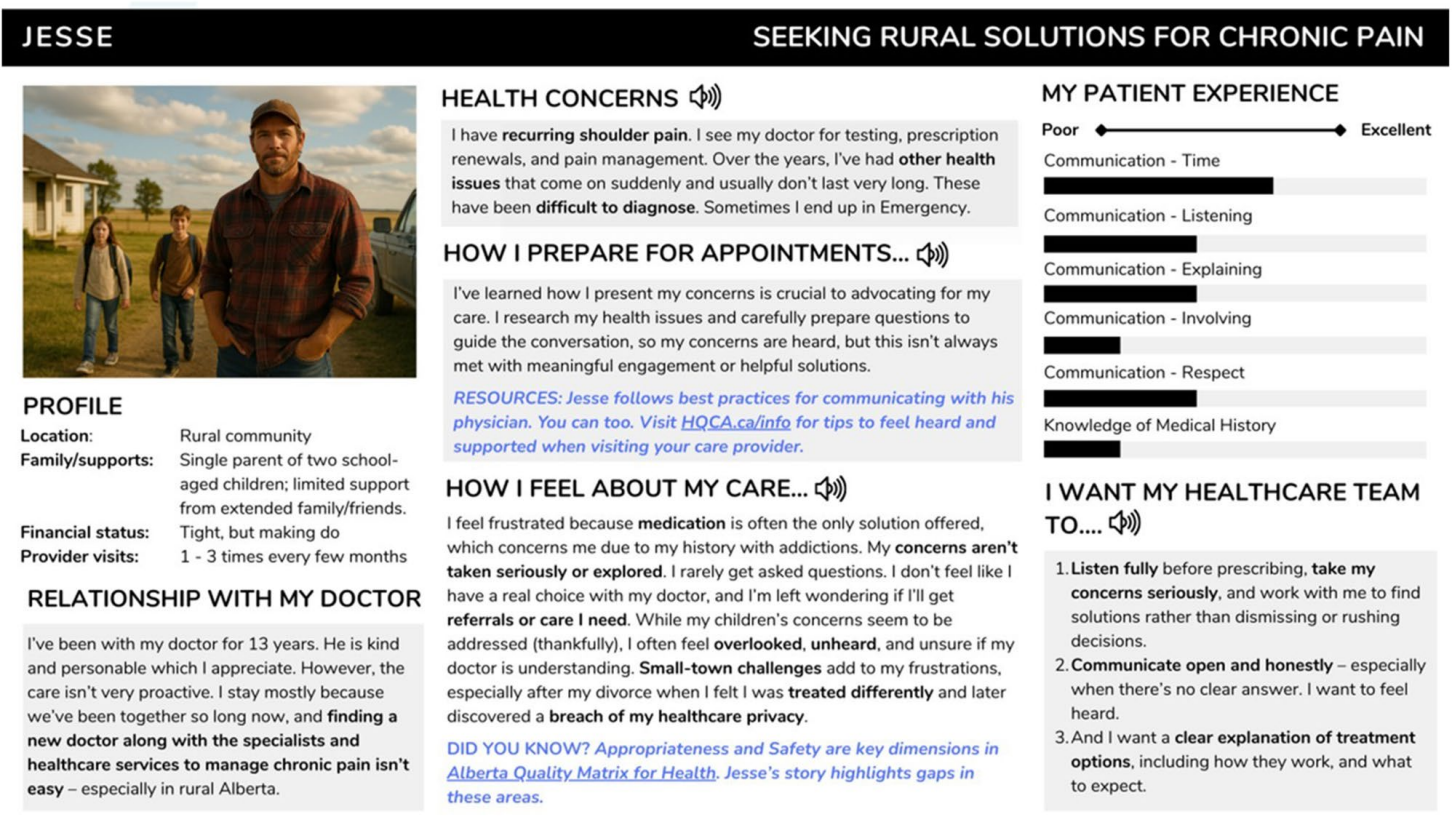
Visual of persona 1

## VP24 The Association Between Self-stigma of Mental Illness and Quality of Life of Adults Living with Depression: A Systematic Review

Refah Alqahtani^1^, Clair Gamble^1^, Jan R. Boehnke^1^

^1^University of Dundee, Dundee, UK

*Journal of Patient-Reported Outcomes 2026*, **10(Suppl 1)**:VP24

### Aims

Self-stigma describes when an individual with a mental illness internalises the negative attitudes that society holds about their condition. Self-stigma is prevalent among people with depression, which can lead to significant harm and negatively impact on their lives. To date, there is no review that examine the association between self-stigma and quality of life (QoL) among patients with depression. The aim of this review was to investigate the association between self-stigma and QoL in adult patients with depressive disorders. Additionally, the review explored which factors are associated with self-stigma.

### Methods

A systematic review was conducted following PRISMA and Joanna Briggs Institute (JBI) guidelines. The review was registered on Prospero (CRD42024500276). Nine databases were searched (CINAHL, PubMed, Scopus, Web of science, APA PsychArticles, Cochrane, Google Scholar, Open Science framework, Figshare). Quantitative or mixed methods studies were included if they sampled adults aged eighteen and above of both genders, who had been clinically diagnosed with depression, and if the exposure ‘self-stigma’ was measured with a validated scale. The COSMIN risk of bias checklist was used to assess the used self-stigma scales. A narrative synthesis was used to aggregate the findings.

### Results

Seven studies were included (N=1,244 patients). Five instruments were used to measure self-stigma, and four instruments to measure QoL (one of these to measure HRQL, three of these to measure QOL). Increased levels of self-stigma were associated with reduced QoL, and the strongest associated QoL domains with self-stigma were the physical health and the psychological domains. Self-stigma was identified to be associated with specific demographic, clinical and psychosocial factors (see Table 1 for details).

### Conclusion

The review showed that the experience of self-stigma was negatively associated with QoL. Importantly, substantially smaller associations were observed in studies that controlled for other variables, pointing to potential confounding factors or mediators. The observed confounding factors include severity of depression, level of family function, sociodemographic and clinical. As a main limitation, all included studies were cross-sectional and none of them explicated a causal model, highlighting a need for further research uncovering the potential impacts of self-stigma on QoL.

**Table 1 (abstract VP24):**
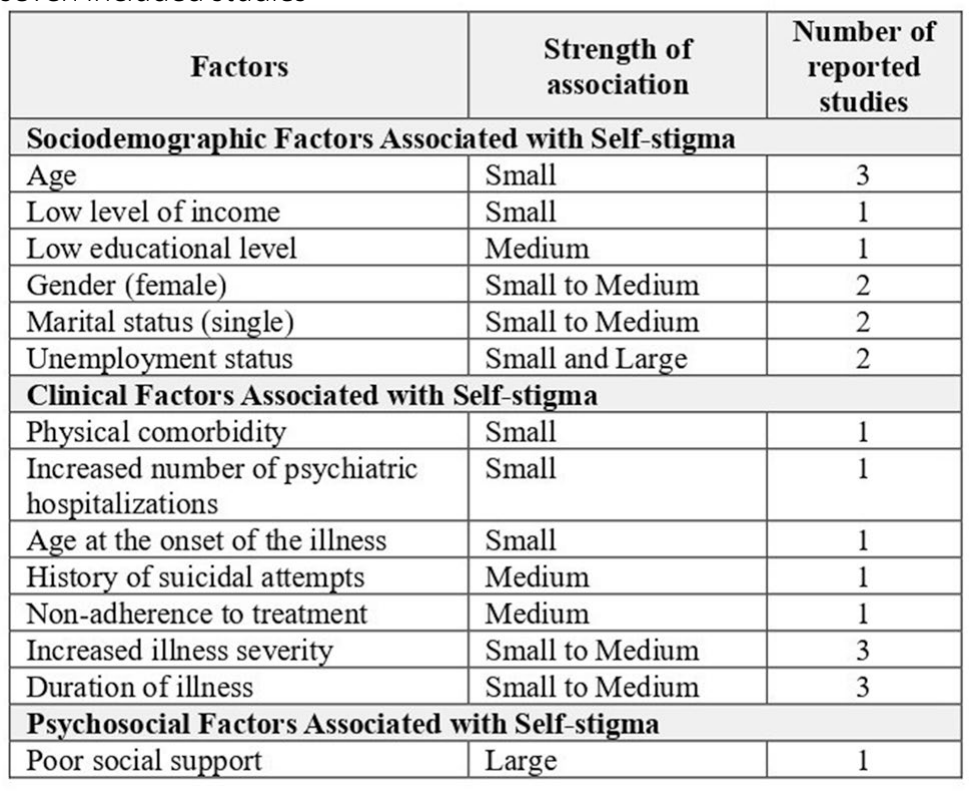
Factors Associated with Self-stigma in the seven included studies

## VP25 Assessing security in telemedical care from the patient’s perspective: Further development of the SeCu questionnaire

Klara Greffin^1^, Annalena Krupskin^1^, Silke Schmidt-Schuchert^1^, Holger Muehlan^2^

^1^University of Greifswald, Greifswald, Germany, ^2^HMU Health and Medical University, Erfurt, Germany

*Journal of Patient-Reported Outcomes 2026*, **10(Suppl 1)**:VP25

### Aims

The SeCu-20 questionnaire is a Patient-Reported Experience Measure (PREM) developed to assess patients’ perceived security in telemedical care. The original version included 16 items covering four facets and two overarching domains. However, findings from previous studies highlighted the need for revision and refinement to improve the instrument’s psychometric properties and practical relevance. The aim of the study was to revise and validate the SeCu-20 questionnaire to enhance its reliability, validity, and applicability in assessing patients’ perceived security within diverse telemedical contexts.

### Methods

A multi-step procedure was employed to revise the SeCu-20, including item refinement, expert evaluations, pilot testing, and subsequent revisions. The resulting version 2.0 of the questionnaire was validated in a sample of 88 adult patients with mental health disorders or heart failure, all of whom were receiving various forms of telemedical care (e.g., telemonitoring). For validation purposes, the SeCu-20 was administered in conjunction with other established instruments, including the Tele-QoL. Data analysis involved descriptive statistics at the item level and psychometric evaluation of the subscales.

### Results

The revised SeCu-20 demonstrated good psychometric properties. Internal consistency was high for three of the four subscales and acceptable for the fourth. Subscale scores showed expected correlations with corresponding scales of the Tele-QoL, providing evidence for convergent validity. Evidence for divergent validity was also found; however, findings regarding discriminant validity were mixed.

### Conclusion

SeCu-20 version 2.0 comprises four subscales and effectively captures patients’ perceived security in telemedical care. The instrument is particularly valuable for use in technology assessment, implementation research, and evaluation studies. Additionally, it may serve as a useful tool in health services research and patient health monitoring when appropriate.

## VP26 Challenges in analysing the effect of treatment on PROs in cancer registry data

Emma Martin^1^, Mike Greenwood^1^, Rachael Lawrance^1^, Kim Cocks^1^

^1^Adelphi Values PCO, Macclesfield, UK

*Journal of Patient-Reported Outcomes 2026*, **10(Suppl 1)**:VP26

### Aims

Patient-reported outcomes (PROs), which reflect how patients feel and function during and after cancer treatment, are increasingly collected in cancer registries to help understand health related quality of life (HRQoL) in real-world settings. While much has been written about the importance of collecting PROs in prospective registries, there is less guidance on how to analyse and share these results for maximum benefit to multiple stakeholders and patients. Unlike clinical trials, which have strict inclusion/exclusion criteria, cancer registries include a broader range of patients, which makes the data more reflective of everyday experiences, improving generalisability, but also introducing challenges. Our aim is to discuss challenges associated with summarising the impact of treatment on PRO data in longitudinal cancer registries and to suggest ways in which these challenges can be overcome.

### Methods

We use visualisations of patient disease pathways to highlight three key challenges faced when estimating the effect of treatment on HRQoL using registry data. 1. Calculation of completion rates; defining expected assessments can be complex but is important when assessing data quality. 2. Definition of baseline; in clinical trials change from baseline is used to assess the effect of treatment, with baseline assessments completed shortly before treatment begins. For registry data, assessment schedules often independent of treatment changes, meaning the “baseline” assessment may have occurred months prior. 3. Visualizations of HRQoL changes over time despite varying PRO assessment times.

### Results

1. We discuss factors to consider when calculating completion rates, and how to separate missing PROs into those which were expected to be collected and those not expected (e.g. due to death or length of follow-up). 2. We suggest analyses to assess the impact of the time between PRO assessment and treatment start date and how that impacts definition of baseline. 3. We present data visualisations to compare a visit windowing approach with a continuous time approach for changes over time.

### Conclusion

Despite the challenges associated with using the PRO data collected in cancer registries they provide a valuable, unique patient perspective, outside of that collected during clinical trials.

## VP27 Key Domains Affected by Lower Limb Amputation: Patient and Healthcare Professional Perspectives

Justin-Pierre Lorange^1^, Virginie Blanchette^2^, Sander L. Hitzig^3^, Audrey Zucker-Levin^4^, Diana Zidarov^5^

^1^Universite de Montreal, Montreal, Quebec, Canada, ^2^Université du Québec à Trois-Rivières, Department of Human Kinetics and Podiatric Medicine and VITAM: Sustainable Health Research Centre, 3351 Bd des Forges, Trois-Rivières, QC G8Z 4M3, Canada, ^3^St. John’s Rehab Research Program, Sunnybrook Research Institute, Sunnybrook Health Sciences Centre, Toronto, Canada, ^4^School of Rehabilitation Science, University of Saskatchewan, Saskatoon, SK, Canada, ^5^Institut universitaire sur la réadaptation en déficience physique de Montréal (IURDPM), Centre for Interdisciplinary Research in Rehabilitation of Greater Montreal (CRIR), Montreal, Quebec, Canada

*Journal of Patient-Reported Outcomes 2026*, **10(Suppl 1)**:VP27

### Aims

Lower limb amputation (LLA) has a profound impact on individuals, affecting physical and mental health, quality of life, and social participation. Major LLA requires comprehensive, multidisciplinary rehabilitation. Standardized outcome measures are essential to assess patient needs, guide prosthetic prescription, monitor progress, and evaluate treatment effectiveness. Importantly, decisions about what to measure in clinical practice must include the perspectives of both patients and healthcare professionals (HCPs) to ensure that selected outcomes reflect what is important to people living with LLA. The aim of this study was to identify the most important domains of health-related quality of life (HRQoL) affected by LLA from the perspectives of people with LLA and HCPs. The goal was to inform the development of a core set of patient-reported outcome (PRO) domains to support holistic, patient-centered rehabilitation.

### Methods

A cross-sectional study was conducted at four Canadian multidisciplinary rehabilitation centers (July 2023-January 2025). Individuals with LLA completed the Patient Generated Index (PGI), an individualized quality of life tool, to identify the most important domains of their lives affected by the amputation. PGI responses were mapped to the International Classification of Functioning, Disability and Health (ICF). An electronic survey was also administered to HCPs (July 2023-April 2024) asking them to rank 19 HRQoL domains from the Patient-Reported Outcomes Measurement Information System (PROMIS) framework. Rankings from both groups were analyzed descriptively, prioritizing the patient perspective.

### Results

A total of 84 HCPs and 96 individuals with LLA participated. The five most important domains identified by HCPs were: physical function, pain interference, satisfaction and ability to participate in social roles and activities, and self-efficacy (Table 1). People with LLA prioritized physical function, self-efficacy, stigma, and satisfaction and ability to participate in social roles and activities (Table 2). Combining both perspectives, ten core PRO domains were identified for routine clinical care (Table 3).

### Conclusion

By integrating patient and clinician perspectives, this study identified key outcome domains encompassing physical function, participation in social roles, pain, and psychosocial well-being. These findings can guide rehabilitation teams in selecting relevant outcome measures to support shared decision-making and individualized care planning, promoting a patient-centered approach to LLA rehabilitation.

**Table 1 (abstract VP27):**
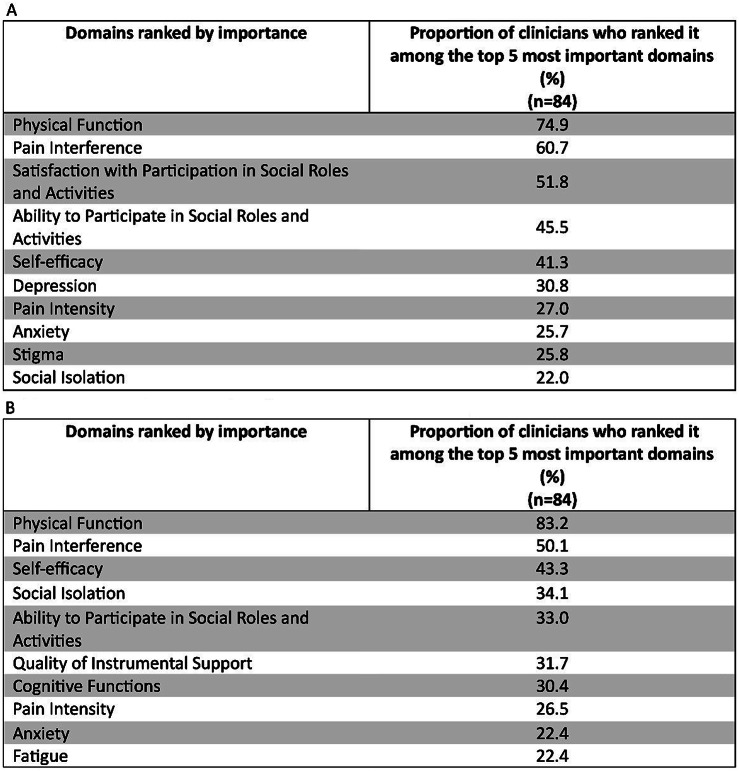
(**A**) 10 most important domains for clinicians (traumatic). (**B**) 10 most important domains for clinicians (vascular)

**Table 2 (abstract VP27):**
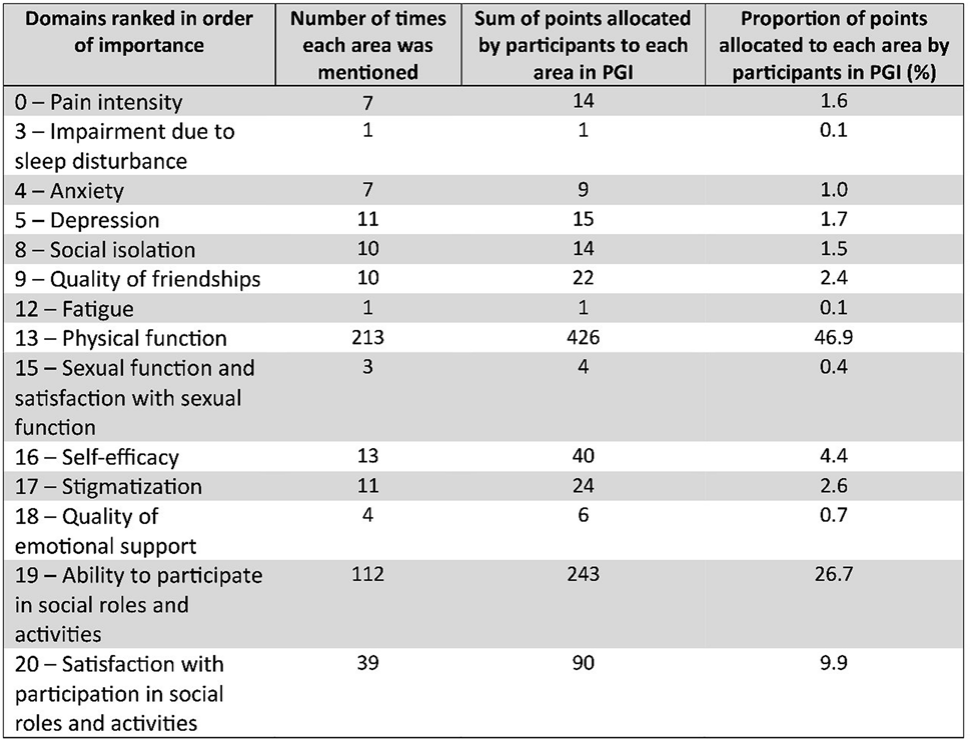
LLA participants (n = 96) perception of the most important areas in their life for which they would like to improve (PGI)

**Table 3 (abstract VP27):**
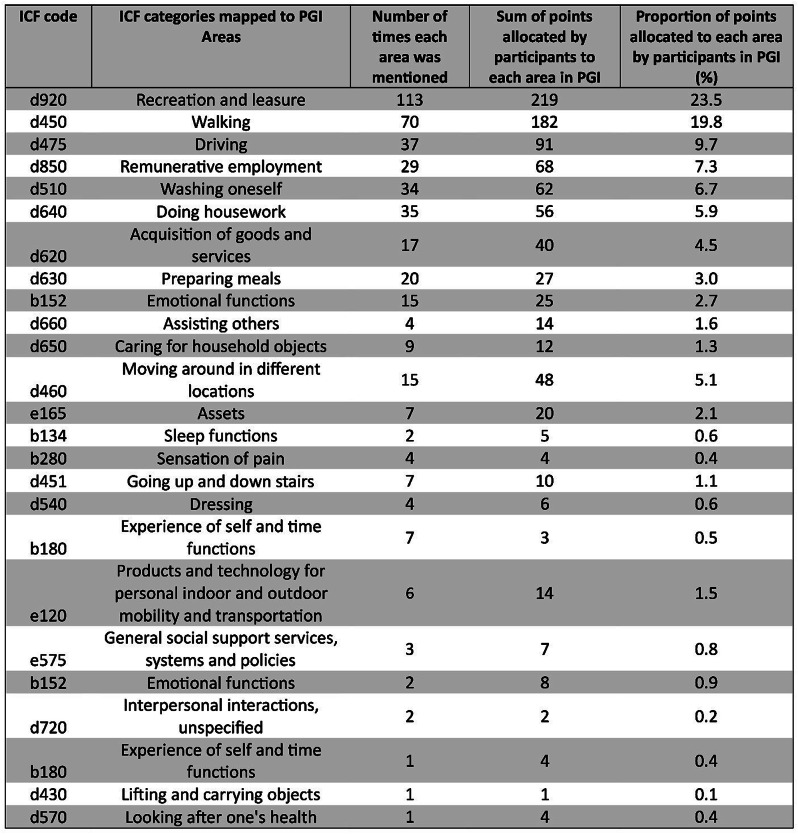
LLA participants (n = 96) perception of the most important domains of HRQOL affected by their LLA for which they would like to have healthcare services (using the PROMIS framework)

## VP28 Grounded in Lived Experience: Community Voices Refine the Obesity-Specific Preference-Based Weight-Related Quality of Life (PB-WRQL) Prototype

Ana Moga^1^, Nancy Mayo^2^, Mary Forhan^3^

^1^Faculty of Medicine, School of Physical Therapy, Rehabilitation Science, McGill University, Montreal, Quebec, Canada, ^2^McGill University, Montreal, Quebec, Canada, ^3^University of Toronto, Toronto, Ontario, Canada

*Journal of Patient-Reported Outcomes 2026*, **10(Suppl 1)**:VP28

### Aims

To refine the dimensions of the prototype PB-WRQL measure and contribute evidence toward its content validity based on input from a community-based working writing group of individuals living with obesity.

### Methods

This participatory, cross-sectional study involved a structured online working group held via Zoom to refine the prototype PB-WRQL descriptive system. Participants were recruited through Obesity Canada’s Connect Community, a moderated online forum, using purposive sampling between July and December 2022. Participants worked in pairs to review PB-WRQL dimensions and response scales, providing structured feedback on clarity, relevance, and comprehensiveness. Paired discussions were followed by a group debrief and submission of suggested revisions.

### Results

All eight participants were white women (mean age = 53.9) with lived experience of obesity. All prototype dimensions underwent revision. Key changes included separating overlapping concepts (e.g., splitting “pain or stiffness” into “back pain” and “joint pain”), removing ambiguous or stigmatizing language (e.g., revising “depressed: because of my weight” to “mood”), and reframing items to emphasize function and capability over interference (e.g., “energy to complete activities” instead of “weight interference with energy”). Social participation was expanded to reflect distinct interpersonal contexts and body-related experiences, including comfort with close relationships, new personal relationships, physical appearance, and sexuality. Response options were revised across items from 3 to 5 levels to enhance granularity. New items proposed to capture difficulty with physical tasks (e.g., bending, walking, stairs), self-perception (e.g., motivation, appearance, body image), and environmental fit (e.g., seating, clothing preferences).

### Conclusion

The revised PB-WRQL prototype incorporates five-level response options and expanded domains rooted in lived experience. It includes physical symptoms, functional limitations, interpersonal relationships, and emotional well-being. These revised items will be evaluated in the next phase of this research to assess response patterns and comparative performance with generic preference-based HRQL measures.

## VP29 Evaluating the PB-WRQL Prototype Against Generic Preference-Based HRQL Measures in People Living with Obesity

Ana Moga^1^, Nancy Mayo^2^, Mary Forhan^3^ Laurie Twells^4^

^1^Faculty of Medicine, School of Physical Therapy, Rehabilitation Science, McGill University, Montreal, Quebec, Canada, ^2^McGill University, Montreal, Quebec, Canada, ^3^University of Toronto, Toronto, Ontario, Canada, ^4^Memorial University, St. John’s, Newfoundland and Labrador, Canada

*Journal of Patient-Reported Outcomes 2026*, **10(Suppl 1)**:VP29

### Aims

This study aimed to identify the distribution patterns and content coverage of the obesity-specific Preference-Based Weight-Related Quality of Life (PB-WRQL) Prototype relative to the EQ-5D-3L, SF-6D, and HUI-3 among a non-clinical, community-based sample of adults living with obesity.

### Methods

A cross-sectional survey study was conducted between July and December 2022. Participants were recruited using purposive sampling through Obesity Canada’s Connect Community, an online forum for individuals living with obesity. Participants completed an online survey that included sociodemographic questions, the PB-WRQL prototype and three preference-based generic indices (EQ-5D-3L, SF-6D, HUI-3). Open-text prompts followed each measure to allow participants to comment on item relevance, identify missing content, and share aspects of HRQL they felt were underrepresented for this population. Two researchers independently analyzed qualitative feedback to identify common themes, and all data were reviewed through team consensus. Descriptive statistics were computed for sociodemographic variables and item-level responses.

### Results

Sixteen participants completed the survey, with most being female (95.5%) and White (90.9%), and a mean age of 53.9 ± 12 years. The mean EQ-5D visual analog scale score was 66.7 ± 21.6. Quantitative analysis showed that the PB-WRQL prototype elicited more differentiated responses across symptom and functional items, such as joint pain, shortness of breath, low energy, swelling, and difficulty with standing or climbing stairs, compared to the generic instruments. In contrast, EQ-5D-3L, SF-6D, and HUI-3 showed clustering at the highest functioning levels, indicating ceiling effects. For example, over 90% of responses on the HUI-3 were at the top level for mobility, emotion, dexterity, and cognition. Similar trends were observed in EQ-5D-3L and SF-6D. Thematic analysis of open-text feedback highlighted the PB-WRQL’s strength in capturing patient-relevant domains. Participants described the generic preference-based measures as overly broad, lacking nuance, and missing key constructs such as stigma, emotional eating, and body image.

### Conclusion

The PB-WRQL prototype demonstrated greater granularity and relevance than generic preference-based HRQL measures in capturing the lived experiences of people with obesity. These findings support its continued development as an obesity-specific, preference-based measure.

## VP30 Measurement properties of PROMIS 29 v2.1 in German, French and Italian for Swiss population in a cross-sectional study

Thanh Elsener^1^, Matthew Kerry-Krause^2^, Felix Fischer^3^, Nikola Biller-Andorno^1^

^1^Institute of Biomedical Ethics and History of Medicine, University of Zurich, Zurich, Switzerland, ^2^Zurich University of Applied Sciences, School of Health Science, Zurich, Switzerland, ^3^Charité – Universitätsmedizin Berlin, Berlin, Germany

*Journal of Patient-Reported Outcomes 2026*, **10(Suppl 1)**:VP30

### Aims

To inspect psychometric properties of the PROMIS-29 v2.1 using a cross-sectional online survey across German, French and Italian-speaking regions of Switzerland. To compare translations across language regions.

### Methods

A prospective cross-sectional design was employed using an online survey methodology, with sampling frames across German, French and Italian language regions of Switzerland. Participants were recruited via postal mailings sent to their home addresses. Up to three reminder letters were subsequently sent to non-respondents to enhance participation rates. Psychometric properties were evaluated according to COSMIN guidelines 2.0 for good measurement properties. Structural validity was evaluated via confirmatory factor analysis (CFI >0.95 OR SRMR <0.08). CFA was conducted for all seven scales combined, as well as separately within each language group. Internal consistency for each scale was evaluated with Chronbach’s alpha > 0.70. Measurement invariance across three languages was assessed with Differential Item Functioning [DIF] with R2McFadden < .02.

### Results

A total of 1,744 participants completed the survey (German: n = 667; French: n = 455; Italian: n = 622). Confirmatory factor analysis (CFA) supported the hypothesized 7-factor model across all language versions, with fit indices meeting established thresholds for acceptable model fit. Unidimensionality was confirmed for each subscale within each language group. All subscales demonstrated sufficient internal consistency (Cronbach’s α ≥ 0.70).Differential item functioning (DIF) analyses revealed no significant DIF between the German and French versions. However, comparisons between the German and Italian versions indicated negligible uniform DIF for items EDDEP06 and SLEEP116, and moderate uniform DIF for items EDANX40, EDANX41, EDDEP27, and SLEEP20.

### Conclusion

The PROMIS-29 v2.1 demonstrated acceptable psychometric properties in the Swiss population, with evidence supporting its structural validity and internal consistency. Measurement invariance was confirmed between the German and French versions, indicating equivalent scale functioning across these linguistic groups.While preliminary analyses revealed no significant DIF between these language groups, further validation should specifically examine potential item-level biases between German and Italian versions and assess longitudinal properties including test-retest reliability, measurement errors and responsiveness.

## VP31 Translation and cultural adaptation of the FACE-Q Paralysis Module from English to Danish

Elena Takasugi Aagaard^1^, Christoffer Bing Ydo^1^, Mette Stueland Wolthers^2^, Trisia Breitkopf^3^, Anne Klassen^3^, Jens Ahm Sørensen^1^

^1^Research Unit of Plastic Surgery, Odense University Hospital, Odense, Denmark, ^2^Department of Plastic Surgery, Breast Surgery, and Burns, Rigshospitalet, Copenhagen University Hospital, Copenhagen, Denmark, ^3^Department of Pediatrics, McMaster University, Hamilton, Ontario, Canada

*Journal of Patient-Reported Outcomes 2026*, **10(Suppl 1)**:VP31

### Aims

Facial nerve paralysis involves loss or weakening of facial muscle movement, impacting physical, social, and mental well-being. The FACE-Q Paralysis Module, developed by McMaster University and the Hospital for Sick Children in Canada, is a Patient Reported Outcome Measure designed for individuals aged 8 and older with congenital or acquired facial nerve paralysis. The FACE-Q Paralysis Module comprises four distinct yet complementary scales that assess appearance (n=46), function (n=45), health-related quality of life (HRQoL; n=48), and adverse effects (n=17). These scales can be used individually or in combination, offering flexibility for clinical or research applications. Additionally, the scales allow clinicians and researchers to assess treatment impact from the patient’s perspective, supporting personalized care and outcome monitoring. While FACE-Q Paralysis has been translated into several languages, a Danish version is not yet available. The aim of this project was to translate and culturally adapt the FACE-Q Paralysis Module from English to Danish following internationally recognized best-practice guidelines.

### Methods

The FACE-Q Paralysis Module was translated using a six-step methodology following guidelines from the World Health Organization and the International Society for Pharmacoeconomics and Outcome Research to ensure conceptual equivalence and cultural relevance. Steps include: (1) preparation and approval, (2) forward translation, (3) back translation, (4) expert panel review, (5) cognitive debriefing with patients, and (6) proofreading.

### Results

The forward translation revealed minor terminological discrepancies, which were discussed to produce a harmonized Danish version 1. The back translation review revealed 12 items requiring re-translation. An expert panel meeting addressed linguistic inconsistencies in 14 items to ensure alignment with the original English meaning. Cognitive interviews are pending to assess patient comprehension.

### Conclusion

The translation process is ongoing. Forward and back translations are complete, and expert panel revisions have been incorporated. Patient recruitment for cognitive interviews is underway. The translation and cultural adaptation will result in a conceptually equivalent and culturally adapted Danish version of the FACE-Q Paralysis Module. This will enable future research using the FACE-Q Paralysis scales to accurately assess and potentially improve HRQoL and treatment for individuals with facial nerve paralysis.

## VP32 A Mixed Methods Analysis Using Trial Data to Describe Patients with Glycogen Storage Disease Type Ia Who Met Baseline Expectations for Meaningful Change in Daily Cornstarch Intake

Diane M. Turner-Bowker^1^, Shayna Egan^1^, Deepali Mitragotri^1^, Blaise Cureg^2^, Martha Gauthier^2^

^1^Ultragenyx Pharmaceutical, Novato, California, USA, ^2^Lumanity, Boston, Massachusetts, USA

*Journal of Patient-Reported Outcomes 2026*, **10(Suppl 1)**:VP32

### Aims

A mixed methods analysis was conducted using qualitative and quantitative data from a phase 3 clinical trial evaluating an investigational treatment for glycogen storage disease type Ia (GSDIa) to determine whether individual patient-level baseline expectations for clinically meaningful change on the primary endpoint were met at the efficacy timepoint.

### Methods

Data were from a phase 3, randomized, double-blind, placebo-controlled trial (NCT05139316) in pediatrics (8 to <18 years) and adults (≥18 years) with GSDIa assessing efficacy/safety of DTX401, an investigational adeno-associated virus serotype 8 vector designed to express the human G6PC1 gene. Patient-level expectation for meaningful percent reduction in daily cornstarch intake collected during phase 3 baseline interviews were compared with patient-level actual percent reduction in daily cornstarch intake at Week 48 and at Week 96. Each patient was then categorized as having not met, met, or exceeded baseline expectation. Results were summarized by treatment group. The average change in daily cornstarch intake was described for the group that met or exceeded baseline expectations. Sample quotes from baseline, Week 48, and Week 96 interviews describe the patient experience.

### Results

A higher proportion of DTX401-treated patients met or exceeded baseline expectations for meaningful reduction in daily cornstarch intake at Week 48 versus placebo patients. Similarly, a high proportion of DTX401-treated patients met or exceeded baseline expectations for meaningful reduction in daily cornstarch intake in the crossover period at Week 96. Adult and pediatric patients treated with DTX401 commonly described positive quality of life benefits associated with reduced daily cornstarch intake.

### Conclusion

Findings from this mixed methods research offer a novel approach for investigating clinically meaningful change in key trial endpoints at the patient level. Results from this research align with patient feedback on what would constitute a meaningful reduction in daily cornstarch intake gathered during a phase 3 protocol planning advisory board and yield impactful patient experience results demonstrating the potential treatment benefit of DTX401.

## VP33 Evaluating Responsive Shift in Total Knee Arthroplasty Patients

Ademola Itiola^1^, Tolulope Sajobi^2^, Deborah Marshall^2^, Jeffrey Johnson^1,2^

^1^School of Public Health, University of Alberta, Edmonton, Alberta, Canada, ^2^Department of Community Health Sciences, Cumming School of Medicine, University of Calgary, Calgary, Alberta, Canada

*Journal of Patient-Reported Outcomes 2026*, **10(Suppl 1)**:VP33

### Aims

After undergoing total knee arthroplasty (TKA), patients may adjust their internal standards (recalibration), reframe how they conceptualize certain questions (reconceptualization) and even shift their priorities (reprioritization) over time. These phenomena, collectively known as response shift (RS), can make patient-reported outcome measures (PROMs) scores taken at different time points—such as before and after surgery— sometimes not directly comparable. Ignoring this shift may lead to inaccurate estimation of intervention effect. As there is limited understanding of RS among total knee arthroplasty (TKA) patients, we explored RS in PROM of TKA patients using extant administrative health data.

### Methods

We identified a cohort of 3,389 individuals who had completed EQ-5D-5L pre-surgery and at 3- and 12-months post-surgery between 2013 and 2023 in Alberta, Canada. RespOnse Shift ALgorithm in Item response theory Rasch Measurement Theory (ROSALI-RMT) was used to test for recalibration RS on EQ-5D-5L item(s). We also examined the association between biological sex, age and presurgical WOMAC physical function (PF) score on response shift effect on EQ-5D-5L items while adjusting for baseline differential item functioning. We assessed partial credit model fit at baseline using infit and outfit indices and visualization of item and test characteristic curves.

### Results

The average age of the cohort was 66.99 years (SD=8.41), 61.61% were female and only 20.15% had no comorbidity. All EQ-5D-5L items, except for pain and discomfort at 3 months and mobility at 12 months, were susceptible to non-uniform recalibration RS. The RS-adjusted intervention effect was larger than the unadjusted intervention effect at 3 months (-1.97 [-1.84, -2.10] vs -1.58 [-1.48, -1.68]) and 12 months (-1.69 [-1.58, -1.81] Vs -1.41 [-1.32, -1.50]) with an attenuation of RS effect on the intervention effect over time. Response shift effect varied with age, sex, and presurgical WOMAC PF score. At 12 months, the unadjusted and RS-adjusted intervention effect were similar for males and females, higher for patients <65 years and those with WOMAC PF score of ≤ 31.

### Conclusion

TKA patients experienced recalibration RS, potentially overestimating presurgical health-related quality of life due to disease adaptation. The impact of total knee arthroplasty on patients’ HRQOL may be underestimated when response shift effect is ignored.

## VP34 Child and family-important outcomes in pediatric randomized controlled trials over a 10-year period

Nora Fayed^1^, Simrin Pardal^1^, Nafiz Sadman^1^, Farhana Zulkernine^1^, Shelley Vanderhout^2^

^1^Queen’s University, Kingston, Ontario, Canada, ^2^Queen’s University, Mississauga, Ontario, Canada

*Journal of Patient-Reported Outcomes 2026*, **10(Suppl 1)**:VP34

### Aims

Pediatric trials have been shown to include less patient-important trial outcomes than adult-focused trials. Our objective was to describe indicators of child, family or caregiver-important outcomes in pediatric randomized controlled trials over the last 10-years.

### Methods

The search was conducted in Ovid MEDLINE from 2014 to June using a strategy to identify pediatric trials. Included trials: i) had clustered or individualized randomization, ii) focused on infants, children, adolescents or youth <25, or ii) were maternal-infant or parental trials including at least one trial endpoint about the infant/child. In phase 1 (2014-2019), all abstracts were screened and data extracted manually by two independent reviewers. Due to an explosion in pediatric trials, in phase 2 (2019-2024), only 25% of abstract and full-text screening of the search was conducted by 2 reviewers.Indicators of patient-importance extracted from included manuscripts of trials were: i) report of patient engagement in trial endpoint selection or development; ii) inclusion of psychosocial, or perceived health or quality of life endpoints coded using standardized procedures, and iii) the tool, agent or respondent of the primary co-primary, and secondary outcomes. Trial characteristics such as: inclusion age at baseline, unit of randomization (individual or clustered), intervention type (pharmacological, surgical, medical-technology, other), trial region, and funding type (industry, NGO, government), were extracted.

### Results

1178 randomized clinical trials demonstrated low rates of cited child, family or caregiver input or selection with trial endpoints, <10%, in each trial year. Specific child or youth engagement, (as opposed to parent/caregiver) increased from 3 to 8% over the 10-year period. Inclusion of psychosocial, perceived health or quality of life primary endpoints ranged from 31-40%, but increased for secondary endpoints from 15 to 30%.

### Conclusion

Despite global initiatives to increase patient engagement in pediatric trials, child, parent, or caregiver engagement in selection of trial endpoint remains low. The doubling of psychosocial, perceived health or quality of life as secondary endpoints demonstrates potential for increasing demand for evidence based on patient-important outcomes.

## VP35 The Impact of Clinical Trial Participation on Quality of Life in Families Affected by Rare Disease

Aidan Leffler^1^, Graham Leffler^2^, Marielle Contesse^3^

^1^University of Washington, Bellevue, Washington, USA. Graham Leffler^2^he International School, Bellevue, Washington, USA, ^3^Red Nucleus, Seattle, Washington, USA

*Journal of Patient-Reported Outcomes 2026*, **10(Suppl 1)**:VP35

### Aims

This study aimed to understand the individual aspects of clinical trial participation that had an impact, whether positive or negative, on both patient and family quality of life for families affected by Duchenne Muscular Dystrophy. It also assessed families’ overall perception of their clinical trial experience and the factors that most impacted their perception. The study calculated agreement between the sum of the individual factors and the overall experience.

### Methods

Qualitative interviews were conducted remotely with 26 caregivers and 7 individuals with DMD using a semi-structured interview guide. The study was approved by the University of Washington IRB. Participants were asked about demographics and their experiences in clinical trials. Descriptive statistics were used to summarize patterns in the data. The mean impact of the individual factors identified as affecting quality of life were categorized as positive, neutral, or negative, and agreement was calculated with net experience using Cohen’s Kappa.

### Results

64.51 percent of respondents [n=15 caregivers, n=4 patients, n=1 joint] assessed their overall clinical trial experience as positive, 12.90 percent as neither [n=3 caregivers, n=1 patient), and 22.58 percent as negative [n=6 caregivers, n=1 patient]. Treatment impact was the most frequently cited reason [n=14 caregivers, n=4 patients]. Other reasons cited included overall study burden (n=3 caregivers), hope (n=2 caregivers), altruism (n=3 caregivers, n=1 patient), missing out on life events (n=1 caregiver), and time with family (n=1 patient). Respondents mentioned 23 individual factors involved in clinical trial participation as having an impact on quality of life, with a mean impact assessment of -.74 [3=very positive impact, -3=very negative impact]. There was poor agreement [Kappa=0.12] between respondents’ assessment of their overall clinical trial experience and the mean impact factor of the individual aspects of trial participation that impacted quality of life.

### Conclusion

When asked about factors about trial participation that impacted quality of life, families affected by Duchenne primarily identified negative factors. However, a majority of families reported their overall experience positively; the impact of treatment was cited as the most frequent reason, indicating that the analysis quality of life data in clinical trials should take the impact of trial participation itself into account.

## CERP1 Concept Extraction from full text publications in qualitative literature reviews using COAScape AI, a Biomedical Large Language Model

Kristian Thorlund^1^, Claire Burbridge^2^, Lucy Lloyd-Price^2^, Stacie Hudgens^3^

^1^McMaster University, Hamilton, British Columbia, Canada, ^2^Clinical Outcomes Solutions, Folkstone UK, ^3^Clinical Outcomes Solutions, Tucson, Arizona, USA

*Journal of Patient-Reported Outcomes 2026*, **10(Suppl 1)**:CERP1

### Aims

COAScape AI is a biomedical large language model (LLM) being developed and trained to facilitate expert researchers conducting qualitative literature reviews (QLR). QLRs involve identifying and extracting key concepts and themes that are discussed by participants and reported within the publications. Manual data extraction by a researcher is resource-intensive, time-consuming, and can be prone to variability. This study aimed to explore the accuracy of COAScape AI in extracting key concepts and themes from full-text publications of qualitative research.

### Methods

Data from 19 qualitative literature reviews previously conducted by expert clinical outcomes assessment (COA) researchers created the dataset used in this evaluation. A proportion of the dataset was used to train COAScape AI and a random sample of the dataset was used to compare the concepts and themes identified and extracted by an expert annotator with those extracted using COAScape AI. This comparison dataset included over 200 full-text articles. The full-text results from these articles were broken down into 5,040 distinct text segments (paragraphs or smaller text units) from which key concepts and themes were extracted. The concepts and themes identified from each text segment by the expert annotator and COAScape AI were independently reviewed to determine level of agreement.

### Results

The expert annotator and COAScape AI both identified approximately 1900 key concepts across all text segments (not distinct concepts; the same concept could be identified across segments). There was substantial or complete agreement in the concepts extracted in over 80% of text segments. COAScape AI also correctly identified more than half of the concepts extracted by the expert annotator in more than 10% of the remaining text segments. Notably, in approximately 4% of cases, COAScape AI accurately captured concepts missed or erroneously annotated by the expert human reviewers. It failed to extract relevant concepts from only 2% of text segments.

### Conclusion

COAScape AI demonstrated high accuracy in concept extraction when compared to the expert annotator, demonstrating its potential to substantially streamline qualitative reviews and assist the expert COA researcher in identifying relevant concepts from published qualitative literature.

## CERP2 GPT-4o Accurately Identifies Patient-Reported Free Text Symptoms After Ambulatory Cancer Surgery Without Need for Direct Human Supervision: A Zero-Shot Learning Task in Investigating AI-Assisted Personalized Oncology Care

Yuelin Li^1,^^2^, Jennifer R. Cracchiolo^3^, Aleksandr Petrov^4^, Thomas M. Atkinson^1^

^1^Department of Psychiatry & Behavioral Sciences, Memorial Sloan Kettering Cancer Center, New York, USA, ^2^Department of Epidemiology and Biostatistics, Memorial Sloan Kettering Cancer Center, New York, USA, ^3^Department of Surgery, Memorial Sloan Kettering Cancer Center, New York, USA, ^4^Department of Digital Informatics & Technology Solutions, Memorial Sloan Kettering Cancer Center, New York, USA

*Journal of Patient-Reported Outcomes 2026*, **10(Suppl 1)**:CERP2

### Aims

Emerging research using large language models (LLMs) showed promise in capturing patient-reported outcomes (PROs) using free-text items. However, LLM’s performance in recognizing fine-grained and highly person-centric symptoms, without supervision, is understudied. We aim to develop LLMs that facilitate comprehensive integration of patient voice into PRO symptom assessment.

### Methods

Leveraging the latest GPT-4o pre-trained by OpenAI licensed to our institution in a secure enterprise cloud-based Microsoft Azure offering, N=1,208 cancer patients undergoing ambulatory surgical procedures were asked to remotely report additional concerns related to recovery via a free text box. Responses from thyroidectomy patients (n=147) were analyzed by a trained coder and verified by a surgeon into 111 unique symptoms (e.g., ‘headache’, ‘dizziness’, ‘swollen incision’) and used as the gold standard. Unsupervised Zero-Shot Learning (ZSL) was applied to identify symptoms and build a symptom taxonomy using word embeddings. Model performance was evaluated by area under the ROC curve (AUC) and other standard metrics. ZSL was iteratively improved to optimize performance; and once validated, summarized symptoms in other surgeries.

### Results

GPT-4o identified 97 symptoms post-thyroidectomy, including (ordered by prevalence) ‘headache’ (AUC=0.91), ‘dizziness’ (AUC=0.97), ‘cough’ (AUC=0.92), ‘sore throat’ (AUC=0.72), ‘swollen incision’ (AUC=0.94), ‘diarrhea’ (AUC=0.99), ‘numbness’ (AUC=0.97), ‘swollen neck’ (AUC=0.89), etc. Sparsely-reported symptoms were unsurprisingly lower in AUC (≥0.70). Symptoms were grouped into a hierarchical taxonomy of 40 clusters (Figure 1), e.g., ‘swallowing difficulty’ and ‘swallow pain’ belonging to Swallow Issues (Figure 2), with 0.77 accuracy, 0.79 precision, and 0.76 f1-score across all symptoms. Optimized ZSL applied to other surgeries identified 486 unique symptoms without supervision, most prevalent in mastectomy being ‘pain’ (18%), ‘swelling’ (12%), ‘drainage issues’ (7%); prostatectomy: ‘swelling’ (16%), ‘pain’ (13%), ‘hematuria’ (9%); and nephrectomy: ‘pain’ (38%), ‘swelling’ (9%), and ‘hematuria’ (7%). High internal consistency was observed (Κ=0.84, 0.81, and 0.90, respectively).

### Conclusion

Optimized ZSL performed exceptionally well and transferred automatically across a broad set of surgical procedures without human supervision, offering feasibility for tailored, individualized, verbatim symptom reporting for patients; emergent post-surgical concerns were identified that are not captured by standard PROs. Utilizing LLM technology to enhance (but not replace) conventional PROs with open-ended items holds significant promise in providing personalized oncology care.

**Fig. 1 (abstract CERP2):**
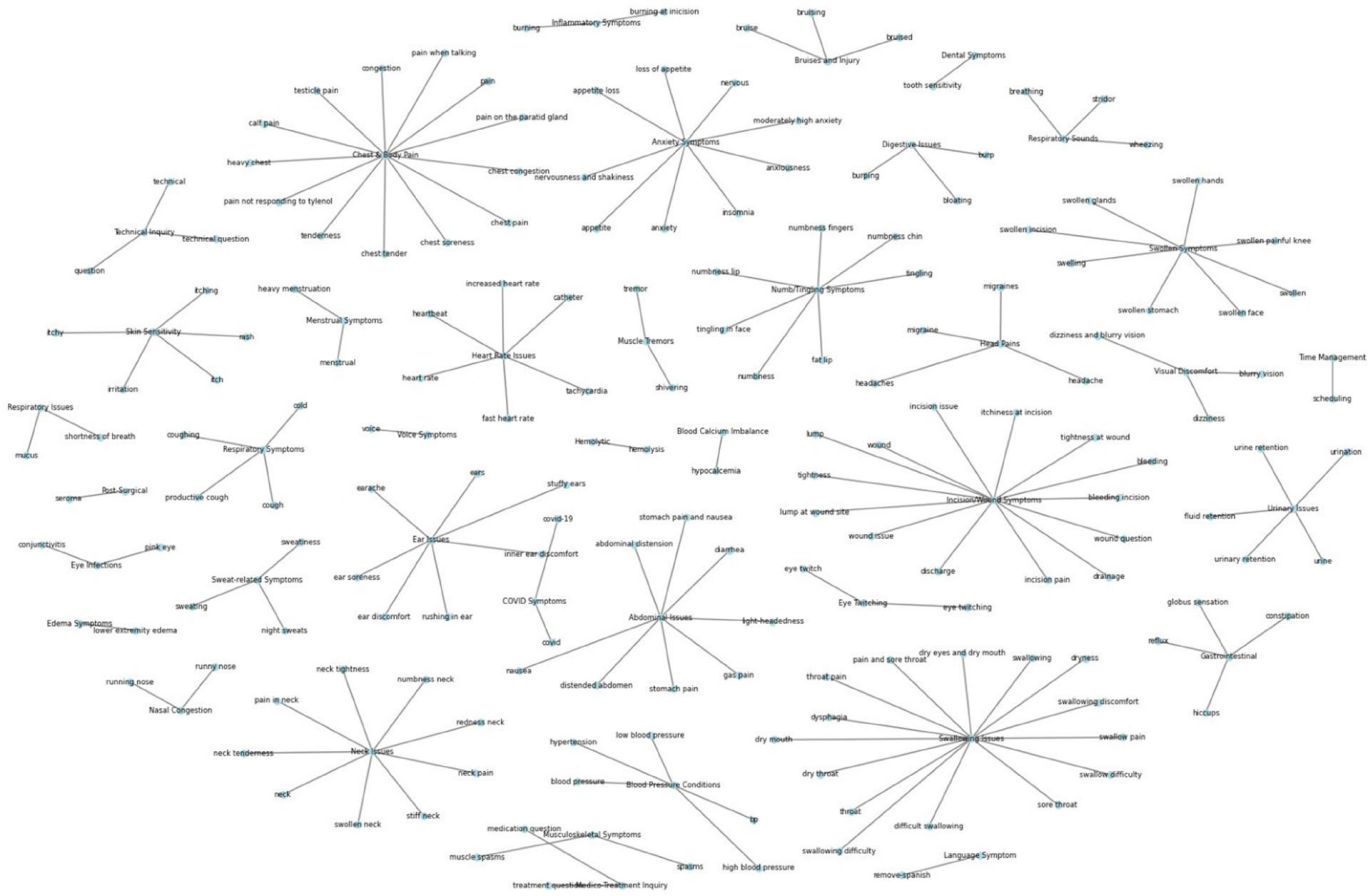
Graph of symptom clusters identified by Louvain method and named by an LLM

**Fig. 2 (abstract CERP2):**
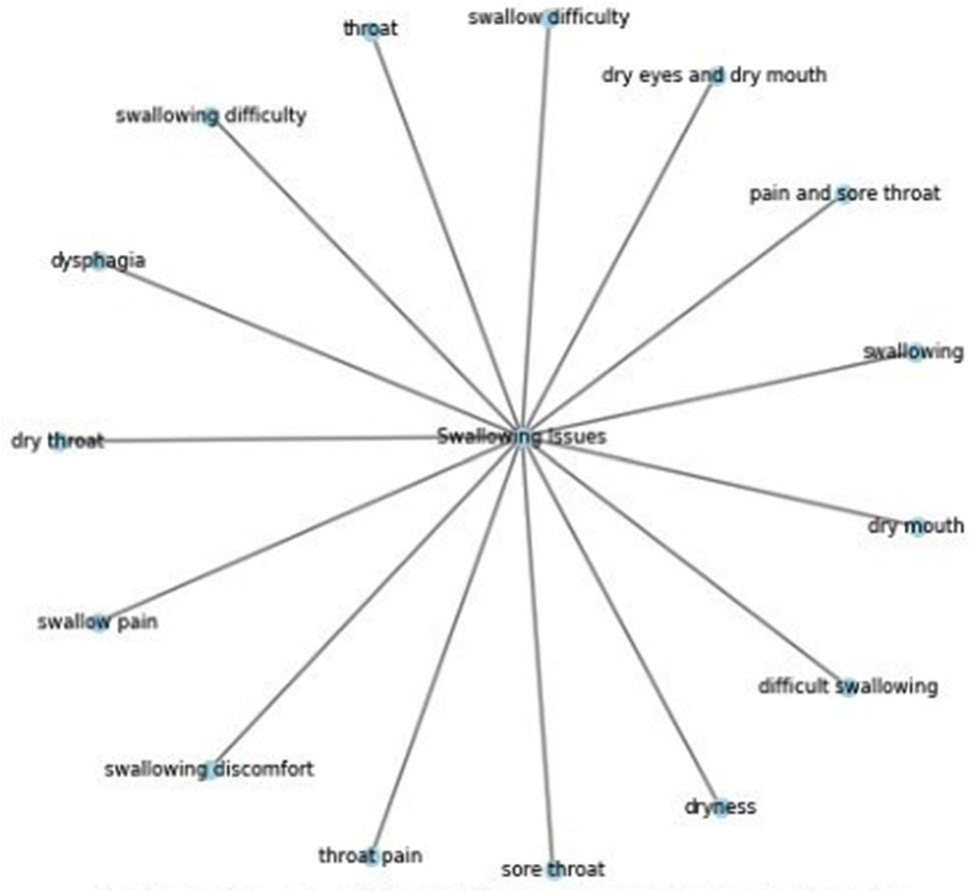
An illustrative symptoms group in an undirected graph for “Swallowing Issues”

## CERP3 Synthetic Patient Generation in Myelodysplastic Syndromes: A Novel Approach Integrating Patient-Reported Outcome Data in Prognostic Models

Fabio Efficace^1^, Giovanni Marsili^1^, Serena Puglia^1^, Francesco Sparano^1^, Paola Fazi^1^, Marco Vignetti^1^, Alfonso Piciocchi^1^

^1^Italian Group for Adult Hematologic Diseases (GIMEMA), Data Center and Health Outcomes Research Unit, Rome Italy

*Journal of Patient-Reported Outcomes 2026*, **10(Suppl 1)**:CERP3

### Aims

Myelodysplastic syndromes (MDS) are heterogeneous hematologic disorders requiring accurate prognostic stratification. While prognostic models are evolving to possibly include patient-reported outcomes (PROs), their validation process remains a major challenge. We present a novel synthetic data generation approach that, for the first time, incorporate PRO data to enable internal validation of the FA-IPSS-R index, an enhanced prognostic model which integrates self-reported fatigue to the purely laboratory-based IPSS-R index.

### Methods

Using clinical, demographic, and PRO data from 927 patients in the PROMYS study (NCT00809575), a synthetic cohort of 5,000 patients was generated with the synthpop package in R. Classification and Regression Trees (CART) were used to model complex variable relationships and retain multivariate structure. Dataset fidelity was evaluated using Kolmogorov-Smirnov, Wilcoxon rank-sum, and Chi-squared tests. Propensity Mean Squared Error (pMSE) and pMSE ratios quantified the utility of the synthesis process. The FA-IPSS-R was validated in the synthetic cohort using Kaplan-Meier survival estimates, log-rank tests and Cox regressions.

### Results

The synthetic dataset accurately reflected the distributions and interrelationships of the original data, including PRO domains. No statistically significant differences were detected across variables. The Kolmogorov-Smirnov tests showed p-values > 0.05 for all PRO domains, indicating comparable distributions. All pMSE ratio values stayed within the recommended threshold of 3 for univariable comparisons, confirming the acceptable utility of the synthetic replicates. The FA-IPSS-R index stratified patients into four prognostic groups with median overall survival of 72, 51, 21 and 16 months and followed the same gradient observed in the real cohort, log-rank p < 0.001. Univariate Cox model in the synthetic data produced estimates for FA-IPSS-R groups comparable to those from the original analysis, with a mean overlap between confidence intervals of 71% (to account for differences in sample size between the synthetic and original datasets, variance estimates were adjusted accordingly).

### Conclusion

This study shows the feasibility of generating high-fidelity synthetic cohorts that preserve PRO data and support robust model validation. The FA-IPSS-R index was successfully validated using synthetic data, reinforcing the importance of integrating patient-reported fatigue in MDS prognostication. This approach offers a scalable, privacy-preserving framework for future research and model validation in other cancer populations.

## CERP4 The potential role of artificial intelligence in the translation of PROMs: a pilot study with the Dutch translation of the PROMIS® Sexual Function and Satisfaction measures

Lorynn Teela^1^, Tessa van Gastel^1^, Athanasios Angelakis^1^, Lotte Haverman^1^, Caroline Terwee^1^

^1^Amsterdam UMC, Amsterdam, Netherlands

*Journal of Patient-Reported Outcomes 2026*, **10(Suppl 1)**:CERP4

### Aims

Translation and linguistic validation of patient-reported outcome measures (PROMs) is a time-consuming and costly process. There is increasing interest in the use of artificial intelligence (AI) for translation, but studies comparing the translation quality of AI supported translations to human translations are scarce. We aimed to explore the potential of AI in the translation of PROMs.

### Methods

A total of 58 items from the PROMIS v2.0 SexFS item banks were translated into Dutch through standard PROMIS methodology. In addition, ChatGPT3.5 and DeepL were used to translate the PROMIS SexFS items. Five PROM experts, four sexuality experts, and five language experts rated the quality of the three translations (human, ChatGPT3.5, and DeepL), presented in a blinded and random order. Experts were given three assignments: (1) Rate the quality of the translation. Each expert received the 58 original English items as well as 1 translation per item (randomly selected from the 3 translations). (2) Which item translation is the best? Each expert received the 58 original English items as well as the 3 translations per item (in random order per item and per expert). (3) Which overall translation is the best? Each expert received the 58 original English items as well as the 3 translations per item (same order for each item, but different among experts).

### Results

Multiple native Dutch and English speakers were involved in the translation process using standard PROMIS methodology. This translation process also included five independent reviews and pretesting with 20 Dutch adults. The results of the comparison of translations using different methodologies, varying in number of people, time, and costs involved, will be presented at the conference.

### Conclusion

 Although this study has some limitations (only one language, highly educated raters), the results will contribute to the currently limited evidence on the appropriateness of AI use in PROM translation. More experimental studies are needed, as an improved translation process could significantly enhance the global adoption and use of PROMs.

